# 2-Arylquinolines as novel anticancer agents with dual EGFR/FAK kinase inhibitory activity: synthesis, biological evaluation, and molecular modelling insights

**DOI:** 10.1080/14756366.2021.2015344

**Published:** 2021-12-20

**Authors:** Mostafa M. Elbadawi, Wagdy M. Eldehna, Amer Ali Abd El-Hafeez, Warda R. Somaa, Amgad Albohy, Sara T. Al-Rashood, Keli K. Agama, Eslam B. Elkaeed, Pradipta Ghosh, Yves Pommier, Manabu Abe

**Affiliations:** a Department of Chemistry, Graduate School of Science, Hiroshima University, Hiroshima, Japan; b Department of Pharmaceutical Chemistry, Faculty of Pharmacy, Kafrelsheikh University, Kafrelsheikh, Egypt; c Pharmacology and Experimental Oncology Unit, Cancer Biology Department, National Cancer Institute, Cairo University, Cairo, Egypt; d Department of Cellular and Molecular Medicine, University of California San Diego, La Jolla, CA, USA; e Faculty of Pharmacy, Kafrelsheikh University, Kafrelsheikh, Egypt; f Department of Pharmaceutical Chemistry, Faculty of Pharmacy, The British University in Egypt (BUE), Cairo, Egypt; g Department of Pharmaceutical Chemistry, College of Pharmacy, King Saud University, Riyadh, Saudi Arabia; h Developmental Therapeutics Branch, Laboratory of Molecular Pharmacology, Center for Cancer Research, National Cancer Institute, NIH, Bethesda, MD, USA; i Department of Pharmaceutical Sciences, College of Pharmacy, AlMaarefa University, Riyadh, Saudi Arabia; j Department of Medicine, University of California San Diego, La Jolla, CA, USA; k Moores Comprehensive Cancer Center, University of California San Diego, La Jolla, CA, USA; l Veterans Affairs Medical Center, La Jolla, CA, USA

**Keywords:** Quinoline, anticancer, EGFR inhibitors, FAK inhibitors, molecular dynamics

## Abstract

In this study, different assortments of 2-arylquinolines and 2,6-diarylquinolines have been developed. Recently, we have developed a new series of 6,7-dimethoxy-4-alkoxy-2-arylquinolines as Topoisomerase I (TOP1) inhibitors with potent anticancer activity. Utilising the SAR outputs from this study, we tried to enhance anticancer and TOP1 inhibitory activities. Though target quinolines demonstrated potent antiproliferative effect, specifically against colorectal cancer DLD-1 and HCT-116, they showed weak TOP1 inhibition which may be attributable to their non-coplanarity. Thereafter, screening against kinase panel revealed their dual inhibitory activity against EGFR and FAK. Quinolines **6f**, **6h**, **6i**, and **20f** were the most potent EGFR inhibitors (IC_50_s = 25.39, 20.15, 22.36, and 24.81 nM, respectively). Meanwhile, quinolines **6f, 6h**, **6i**, **16d**, and **20f** exerted the best FAK inhibition (IC_50_s = 22.68, 14.25, 18.36, 17.36, and 15.36 nM, respectively). Finally, molecular modelling was employed to justify the promising EGFR/FAK inhibition. The study outcomes afforded the first reported quinolines with potent EGFR/FAK dual inhibition.

## Graphical Abstract

**Figure:**
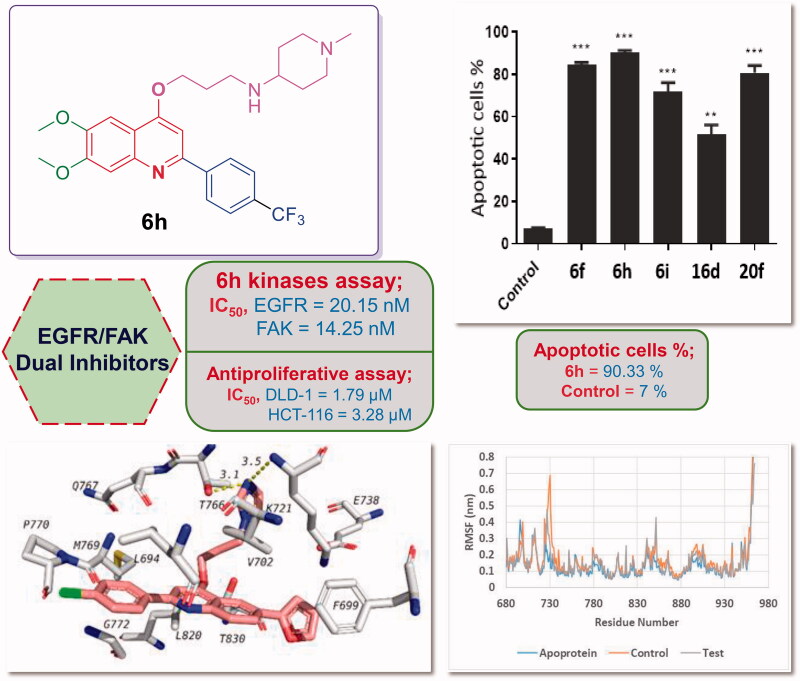

## 1. Introduction

Cancer is a major health obstacle in the world threating the life of millions of people annually^1^^,^^2^. The universal burden of cancer has been expected to rise to 21.6 million in 2023 compared to 14.1 million in 2012 and was predicted to increase to 28.4 million in 2040 with 47% increment relative to 2020^3–5^. In 2020, 19.3 million new cancer cases have been diagnosed and 10 million cancer patients passed away. As established by WHO in 2019, cancer was estimated to be the first or second dominant cause of death for the ages <70 years in 112 countries, while it was projected to be the third or fourth death cause in 23 countries. In general, the incidence and mortality of cancer are growing rapidly worldwide^4^^,^^6^. Subsequently, enormous attempts have been implemented to develop potent anticancer drugs through investigations of diverse scaffolds against numerous potential chemotherapeutic targets^7–9^.

**Figure 1.**
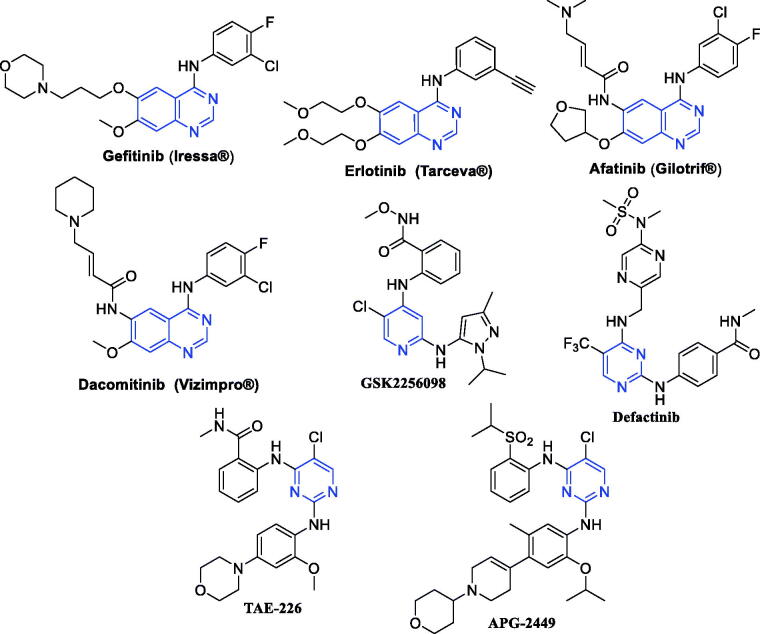
Some reported EGFR and FAK inhibitors.

Epidermal growth factor receptor (EGFR) is a member of tyrosine kinase family in which the endogenous ligand binds to the extracellular domain leading to conformational changes and dimerisation of EGFR resulting in its activation which subsequently stimulates its intrinsic intracellular protein-tyrosine kinase activity^10^. EGFR is over-expressed in many solid tumours and is related to cancer cell proliferation, angiogenesis and metastasis, so it has a critical role in cancer growth. Therefore, EGFR has been validated as an efficient target for anticancer drug discovery. In the last two decades, different 4-anilinoquinazoline-based EFGR inhibitors, such as Gefitinib, Erlotinib, Afatinib, and Dacomitinib (Figure 1), have been FDA-approved for clinical use in treatment of non-small cell lung cancer^11^^,^^12^.

Focal adhesion kinase (FAK) is a cytoplasmic non-receptor tyrosine kinase involved in signal transductions from cell adhesions to regulate different biological cell functions including survival and cell migration^13^^,^^14^. Also, it is activated and overexpressed in diverse cancer types controlling cancer proliferation, survival and metastasis. Thus, FAK has been identified as a promising druggable target for targeted cancer therapy. Currently, several FAK inhibitors, such as 2,4-diaminopyridine derivative GSK2256098 and 2,4-diaminopyrimidine derivative Defactinib (Figure 1), are currently being evaluated in clinical trials for cancer treatment, in addition to the 2,4-diaminopyrimidine derivative TAE-226 (Figure 1) which displayed potent antitumor impact in different cancer types *in vivo* and *in vitro* and usually used as a reference drug^7^^,^^15^^,^^16^. Noteworthy, it was established that the most affected colorectal cancer expressed high levels of EGFR and FAK that particularly correlated with tumour angiogenesis, cancer aggressiveness and poor prognosis^17^^,^^18^.

Thus, dual EGFR/FAK inhibition mechanism is an efficient strategy to fight cancer that could be attributed to a non-overlapping downstream signalling/inhibition^19^^,^^20^. For example, the kinase inhibitor APG-2449 (Figure 1) was reported to improve the antitumor effect of Ibrutinib *via* EGFR/FAK inhibition mechanism in oesophageal squamous cell carcinoma^19^. Also, combined EGFR/FAK inhibition caused higher radiosensitization than either approach alone^21^. Interestingly, few studies have succeeded to develop dual EGFR/FAK small molecule inhibitors. In 2020, Ai et al. has exploited a fragment-based drug design approach to identify novel series of 2,4-diaminopyrimidines as potent dual EGFR/FAK inhibitors with good *in vitro* and *in vivo* antitumor effects^20^.

**Figure 2.**
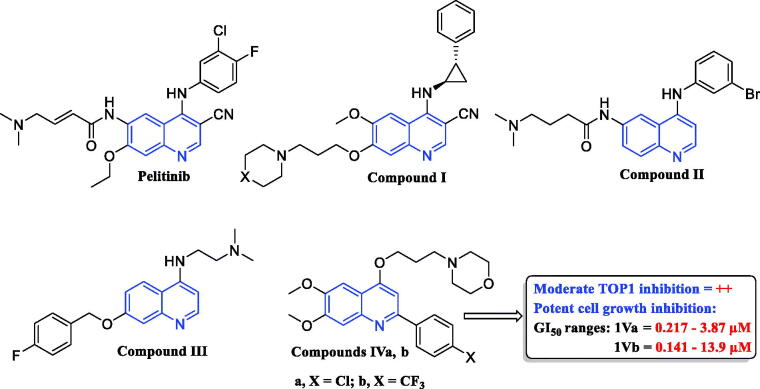
Some reported quinolines with potential anticancer activity.

Quinoline is an outstanding planar heterocyclic motif playing a distinctive role in anticancer drug discovery. So far, assortments of quinoline-based small molecules have been developed and investigated against numerous biological targets for cancer treatment displaying exquisite outcomes^22–25^. It is worth stressing that plenty of quinoline derivatives provoked their anticancer impact through different mechanisms of action, such as inhibition of DNA repair, tubulin polymerisation, and inhibition of various enzymes implicated in critical cancer cell proliferation prominently kinases enzymes (EGFR, VEGFR, pim-1 kinase, c-Met factor, and PI3K) which stood out as one of the most significant targets implemented in cancer therapy due to their functions in cellular signal transduction^26–30^. Of special interest, Pelitinib (EKB-569, Figure 2) is a 4-anilinoquinoline derivative which is a potent irreversible inhibitor of EGFR in the clinical trials as an anticancer candidate^24^. In addition, several 4-aminoquinoline derivatives, such as compounds **I–III** (Figure 2), were reported as promising EGFR inhibitors endowed with effective anticancer activities. Accordingly, quinoline stands out as a significant privileged scaffold in anticancer drug discovery to develop many efficient kinases inhibitors^24^^,^^31–35^.

Recently, we have developed a new series of 6,7-dimethoxy-4-alkoxy-2-arylquinolines as potential Topoisomerase I (TOP1) inhibitors^36^. The TOP1-mediated DNA cleavage assay was utilised to assess the ability of the reported compounds to stabilise TOP1-DNA cleavage complexes (TOP1ccs). The assay outcomes revealed a moderate TOP1 inhibitory activity of compounds **IVa**,**b** (Figure 2). Interestingly, the developed quinolines showed outstanding anti-proliferative profile upon evaluation at the Developmental Therapeutics Program (DTP) of the NCI-USA. Noteworthy, the weightiness of incorporation of *p*-substituted phenyl at *C*-2, as well as propyl linker at *C*-4 of the quinoline scaffold, was highlighted by the SAR study.

**Figure 3.**
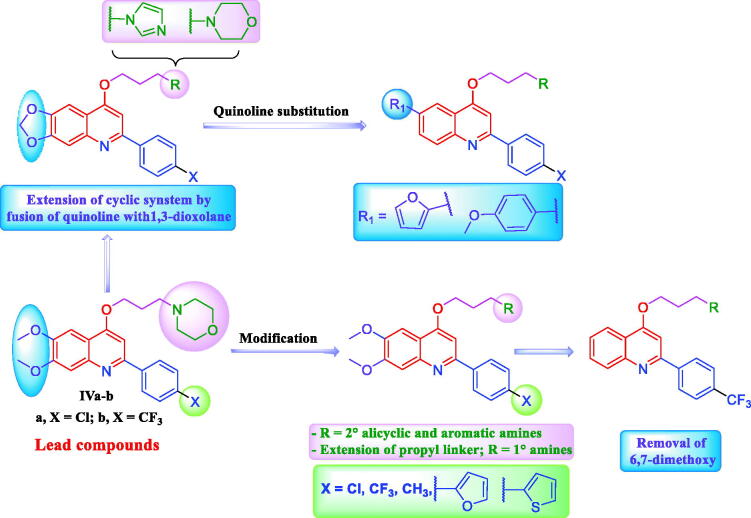
Different structural modification strategies adopted for design of target quinoline derivatives in this study.

As a continuation for our previous study^36^ novel five sets of 4-propoxy-2-arylquinolines (**6a–o**, **8a,b**, **10a,b**, **12a–d**, **16a–d**), and 4-propoxy-2,6-diarylquinolines (**20a–f**) are herein designed and synthesised, exploiting the deduced SARs from the previous study, with the aim to afford more potent anticancer TOP1 inhibitors (Figure 2). Different structural modification strategies were adopted seeking to enhance both anticancer and TOP1 inhibitory activities of the lead compounds **IVa**,**b** (Figure 3).

First, diverse secondary alicyclic and aromatic amines, in addition to different primary amines were appended to the propyl linker to illuminate the influence of these moieties on the activity. Also, we introduced the electron donating methyl group as well as electron withdrawing Cl and CF_3_ for more elucidation of the electronic impact of *p*-substituent of the 2-phenyl. Likewise, the heterocycles: 2-furyl and 2-thienyl were appended to *para* position of the *C2*-phenyl moiety to explore their electronic and size impact on the desired activity. Afterward, the 6,7-dimethoxy groups were removed in some synthesised analogs to confirm their significance. Moreover, the ring system was extended *via* fusion of the quinoline motif with 1,3-dioxolane in attempt to enhance the planar structure requisite for DNA intercalation which may potentiate both anticancer and TOP1 poisoning effects. Finally, a structural extension approach was utilised *via* grafting the HBA-bearing 2-furyl and 4-methoxyphenyl moieties at *C6* of quinoline scaffold, hoping to enhance the hydrophobic interactions (Figure 3). The designed 4-propoxy-2-arylquinolines were prepared employing different synthetic procedures, and then investigated for their anticancer and TOP1 inhibitory activities.

Although the target quinolines demonstrated potent antiproliferative effect against different cancer cell lines, they showed no or weak TOP1 poisoning influence. Accordingly, the promising anticancer activity prompted us to search for the plausible molecular mechanism for herein reported quinolines.

The diverse well-reported kinase inhibitory activities of quinoline-based small molecules, as mentioned above, motivated us to explore the potential inhibitory activity of target 4-propoxy-2-arylquinolines (**6a–o**, **8a,b**, **10a,b**, **12a–d**, and **16a–d**) and 4-propoxy-2,6-diarylquinolines (**20a–f**) against various kinases (EGFR, FAK, FRK, IGF-1R, BTK, c-Src, VEGFR-1, VEGFR-2, HER-2). Strikingly, the investigated quinolines exhibited promising dual inhibitory effect towards EGFR and FAK kinases. Moreover, the apoptotic impact of the most potent anti-proliferative agents in this study was investigated on DLD-1 cells exploiting AV/PI dual staining assay. Finally, *in silico* molecular modelling techniques, including docking and molecular dynamics studies, were exploited to justify and support results obtained from the biological evaluations.

## 2. Results and discussion

### 2.1. Chemistry

**Scheme 1.**
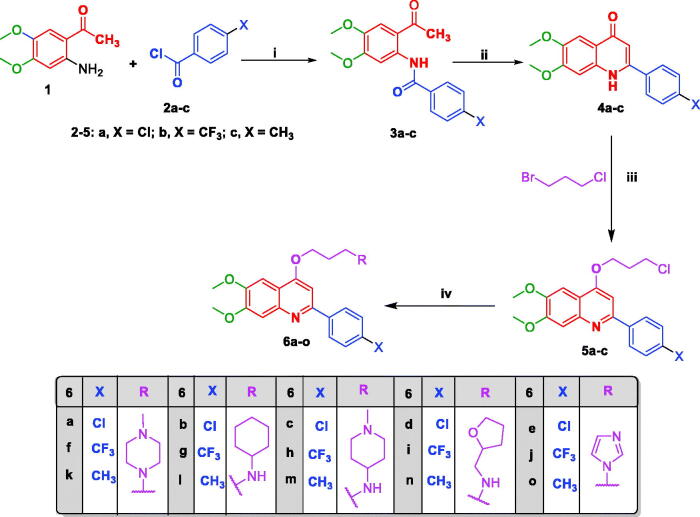
Synthesis of target 4-propoxy-2-arylquinolines **6a–o**; Reagents and conditions: (i) Et_3_N, THF, 0** **°C then rt, overnight; (ii) dry dioxane, NaOH, reflux under N_2_, 110** **°C, 4** **h; (iii) KI, KOH, 1-Bromo-3-chloropropane, dry DMF, rt, 24** **h; (iv) KI, K_2_CO_3_ anhydrous, appropriate amine, dry DMF, reflux, 90** **°C, 12** **h.

**Scheme 2.**
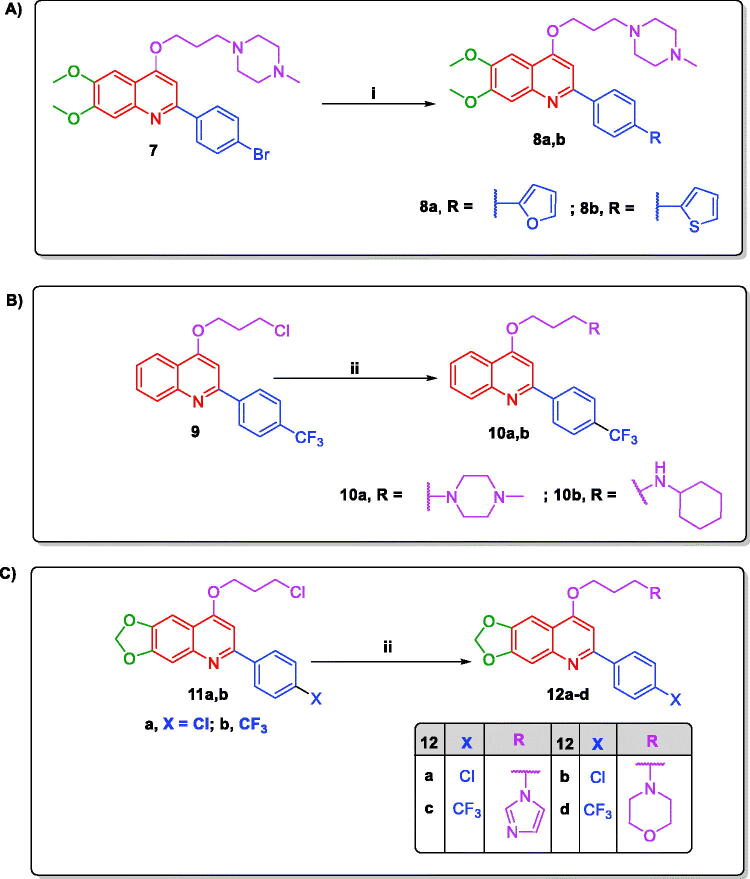
Synthesis of target 4-propoxy-2-arylquinolines (A) **8a,b**; (B) **10a,b**; (C) **12a–d**; Reagents and conditions: (i) Pd(PPh_3_)_4_, 2** **M Na_2_CO_3_, Arylboronic acid, dioxane, 90** **°C under N_2_, 16** **h. (ii) KI, K_2_CO_3_ anhydrous, the respective amine, dry DMF, reflux, 90** **°C, 12** **h.

**Scheme 3.**
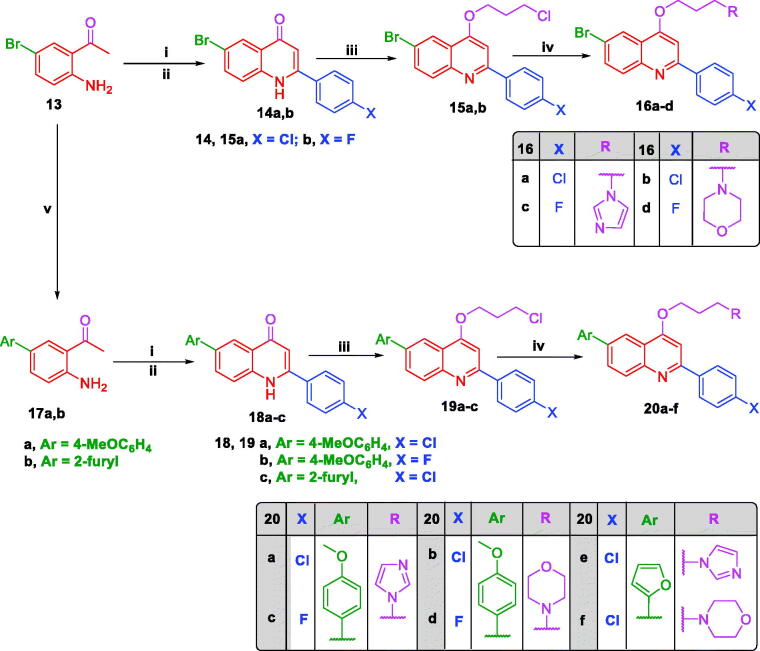
Synthesis of the target 6-bromo-4-propoxy-2-arylquinolines **16a–d** and 4-propoxy-2,6-diarylquinolines **20a–f**; Reagents and conditions: (i) the respective benzoyl chloride, Et_3_N, THF, 0** **°C then rt, overnight; (ii) dry dioxane, NaOH, reflux under N_2_, 110** **°C, 4** **h; (iii) KI, KOH, 1-Bromo-3-chloropropane, dry DMF, rt, 24** **h; (iv) KI, K_2_CO_3_ anhydrous, the respective amine, dry DMF, reflux, 90** **°C, 12** **h; (v) K_2_CO_3_, Pd(PPh_3_)_4_, Arylboronic acid, dioxane/H_2_O (1:1), 100** **°C under N_2_, 4** **h.

The synthetic routes adopted for the synthesis of the target 4-propoxy-2-arylquinolines (**6a–o**, **8a,b**, **10a,b**, **12a–d, 16a–d**) and 4-propoxy-2,6-diarylquinolines **20a–f** are illustrated in Schemes 1–3. Regarding Scheme 1, the benzamides **3a–c** were prepared by reacting 4,5-dimethoxy-2′-aminoacetophenone **1** with *p*-substituted benzoyl derivatives **2a–c** in dry THF and Et_3_N. The latter were cyclized in refluxing dioxane and NaOH to afford the corresponding quinolones **4a–c** in excellent yields which then were subjected to *O*-alkylation with 1-bromo-3-chloropropane using our previously confirmed procedure to yield the respective 4-propoxy key intermediates **5a–c**^36^. Finally, these key intermediates **5a–c** were converted to the target 4-prpoxy-2-arylquinolines **6a–o** in good to excellent yields (70–80%) through nucleophilic substitution with the appropriate amine in dry DMF and potassium carbonate anhydrous using catalytic amount of potassium iodide at 90 °C.

In Scheme 2(A), the bromo analog of 4-propoxy-*N*-methylpiperazine-2-arylquinoline **7** was obtained based on the same pathway for **6a–o** using *p*-bromobenzoyl chloride. Then, this bromo analog **7** has been transformed into the target heterocyclic derivatives **8a,b** in good yields (70–72%) under Suzuki cross coupling condition through its reaction with the appropriate boronic acid derivative in dioxane using tetrakis catalyst and 2 M sodium carbonate under N_2_ at 90 °C. In Scheme 2(B,C), syntheses of the target demethoxylated analogs **10a,b** and 1,3-dioxolo analogs **12a–d** have been accomplished in good to excellent yields (72–87%) adopting the same synthetic routes described for **6a–o** using the respective starting compounds, intermediates, and amines.

Concerning Scheme 3, initiated from 5-bromo-2′-aminoacetophenone **13**, the target 6-bromo analogs **16a–d** have been afforded in good to excellent yields (78–87%) applying the synthetic routes utilised for **6a–o**. Moreover, 5-bromo-2′-aminoacetophenone **13** was transferred to the 6-aryl derivatives **17a,b** *via* Suzuki coupling reaction by its reaction with the appropriate boronic acid derivative in dioxane/water in the presence of tetrakis catalyst and potassium carbonate under N_2_ at 100 °C. Subsequently, these 6-aryl derivatives **17a,b** have been similarly converted to the target 4-propoxy-2,6-diarylquinolnes **20a–f** in good to excellent yields (74–89%) exploiting the same experimental pathways adopted for **6a–o**.

The structures of all synthesised compounds have been authenticated employing one- dimensional (1D) and two-dimensional (2D) NMR (for 2D NMR, see Supplementary Data), in addition to high resolution mass spectrometry (HRMS).

### *2.2. In vitro* anticancer activity

2.2.

#### 2.2.1. Antiproliferative activity against different cancer cell lines

The *in vitro* antiproliferative activity of the synthesised 4-propoxy-2-arylquinolines (**6a–o**, **8a,b**, **10a,b**, **12a–d**, and **16a–d**) and 4-propoxy-2,6-diarylquinolines (**20a–f**) was preliminary investigated by their screening at one dose level (10 µM) against five cancer cell lines representing three cancer types; colorectal cancer (DLD-1 and HCT-116), breast cancer (MDMBA-231 and MCF-7) and cervical cancer (HeLa). After the incubation of the tested compounds for 24 h, the percent growth inhibition (GI%) was calculated. For DLD-1 cell line, twelve compounds exerted good to excellent growth inhibition ranging from 71.94 to 95.36%, from them compounds **6f, 6h, 6i, 16d, 20e**, and **20f** displayed the highest GI%. Similarly, HCT-116 cell line established good to excellent sensitivity to the tested compounds and thirteen compounds exhibited GI% ranging from 64.26 to 97.48%, from them compounds **6d, 6f, 6h, 6i, 12c, 16b, 16d, 20e**, and **20f** demonstrated the best sensitivity (Table 1).

**Table 1.** % Growth inhibition (GI%) of all target compounds **6a–o, 8a,b, 10a,b, 12a–d, 16a–d**, and **20a–f** against different cancer cell lines at 10** **µM dose level.

Compounds	GI%				
	DLD1	HCT-116	MDMBA-231	MCF-7	Hela
**6a**	22.56	31.34	9.38	17.34	20.15
**6b**	36.25	42.81	19.67	23.49	12.81
**6c**	28.22	41.36	19.38	15.76	4.96
**6d**	75.64	82.61	63.39	71.97	26.98
**6e**	15.79	22.03	4.67	5.34	22.36
**6f**	95.36	88.46	75.34	94.36	69.17
**6g**	25.36	22.16	48.15	76.38	46.28
**6h**	92.36	89.34	84.76	90.36	90.78
**6i**	88.76	82.11	81.60	89.49	78.64
**6j**	6.31	2.46	8.42	14.67	5.67
**6k**	18.69	22.15	8.64	17.49	11.32
**6l**	75.34	69.15	38.36	44.71	34.29
**6m**	22.16	24.31	8.16	22.09	30.19
**6n**	81.34	64.26	14.39	58.15	71.12
**6o**	12.36	1.08	8.64	5.36	9.16
**8a**	5.15	1.02	4.40	7.22	11.78
**8b**	74.25	52.10	36.87	74.90	50.81
**10a**	22.51	18.64	37.15	14.26	25.64
**10b**	35.72	24.10	45.35	64.81	55.49
**12a**	21.25	16.25	4.98	11.72	22.79
**12b**	72.95	31.28	14.28	64.38	25.39
**12c**	71.94	80.49	61.94	43.27	68.22
**12d**	22.58	54.37	9.36	26.54	36.67
**16a**	60.08	76.17	51.24	22.97	47.35
**16b**	55.82	87.61	45.31	19.38	35.94
**16c**	12.25	18.14	20.11	4.36	8.67
**16d**	90.11	97.48	80.24	86.38	85.67
**20a**	44.98	36.71	29.08	15.23	22.06
**20b**	53.15	71.05	48.31	29.37	34.02
**20c**	41.34	38.64	32.11	76.82	22.08
**20d**	24.65	36.91	27.05	15.34	11.97
**20e**	90.65	89.64	56.29	73.61	65.73
**20f**	94.18	91.37	82.58	87.36	79.64

Regarding breast cancer cell lines, GI% ranged from 75.34 to 84.76% and 71.97 to 87.36% for MDMBA-231 and MCF-7 cell lines, respectively. While five compounds showed good inhibitory activity towards MDMBA-231, ten compounds possessed good inhibition to the growth of MCF-7 cancer cell line. For HeLa cell line, seven compounds had good GI% from 65.73 to 90.78%. Based on the preceding screening results, it was revealed that colorectal cancer cell lines (DLD-1 and HCT-116) were the most sensitive to the tested compounds, therefore they were selected for further antiproliferative assay at six doses levels (Table 1).

The growth inhibitory activity of the tested 4-propoxy-2-arylquinolines (**6a–o**, **8a,b**, **10a,b**, **12a–d**, and **16a–d**) and 4-propoxy-2,6-diarylquinolines **20a–f** exerted on the colorectal cancer cell lines (DLD-1 and HCT-116) was assessed using MTT assay. DLD-1 and HCT-116 cell lines were incubated for 24 h with increasing concentrations (0.5, 1, 10, 30, 50, and 100 µM) of the tested compounds. Gefitinib (EGFR inhibitor) and TAE226 (FAK inhibitor) were used as reference drugs. The results were presented as half maximal growth inhibitory concentration (IC_50_) which represents the concentration of a drug exhibiting 50% growth inhibition of the cell line compared to the negative control (Table 2).

**Table 2.** The half maximal growth inhibitory concentration (IC_50_) of all target compounds **6a–o, 8a,b, 10a,b, 12a–d, 16a–d**, and **20a–f** against two colorectal cancer cell lines (DLD1 and HCT-116) compared to **Gefitinib** and **TAE226**.

Compounds	IC_50_ (µM)^a^		Compounds	IC_50_ (µM)	
	DLD1	HCT-116		DLD1	HCT-116
**6a**	>100	65.36** **±** **4.37	**10b**	40.28** **±** **2.09	>100
**6b**	19.37** **±** **2.15	16.79** **±** **1.56	**12a**	>100	>100
**6c**	46.95** **±** **2.46	13.70** **±** **1.29	**12b**	8.36** **±** **1.08	14.36** **±** **3.69
**6d**	8.15** **±** **1.05	4.67** **±** **0.85	**12c**	9.12** **±** **2.11	6.37** **±** **1.09
**6e**	>100	>100	**12d**	74.94** **±** **5.46	9.20** **±** **2.49
**6f**	2.25** **±** **0.96	3.09** **±** **1.05	**16a**	8.22** **±** **1.12	6.15** **±** **1.30
**6g**	75.36** **±** **5.46	46.82** **±** **3.16	**16b**	8.69** **±** **2.64	4.22** **±** **1.05
**6h**	1.79** **±** **0.21	3.28** **±** **0.67	**16c**	>100	88.05** **±** **5.24
**6i**	2.48** **±** **0.86	5.68** **±** **1.42	**16d**	2.18** **±** **0.52	2.43** **±** **0.71
**6j**	>100	>100	**20a**	13.05** **±** **3.69	24.11** **±** **2.46
**6k**	>100	>100	**20b**	9.65** **±** **2.15	7.34** **±** **1.82
**6l**	6.34** **±** **0.52	8.11** **±** **1.04	**20c**	15.97** **±** **1.10	12.49** **±** **3.05
**6m**	54.21** **±** **2.11	43.08** **±** **1.05	**20d**	65.30** **±** **5.36	29.47** **±** **4.15
**6n**	6.11** **±** **1.80	8.69** **±** **0.94	**20e**	4.46** **±** **0.65	4.75** **±** **1.02
**6o**	>100	>100	**20f**	2.09** **±** **0.14	2.96** **±** **0.12
**8a**	>100	>100	Gefitinib	10.24** **±** **2.10	6.94** **±** **1.24
**8b**	7.34** **±** **1.22	9.84** **±** **0.68	TAE226	0.12** **±** **0.05	0.17** **±** **0.04
**10a**	>100	>100			

^a^IC_50_ values are the mean of three separate experiments ± *SD*.

The antiproliferative investigations against DLD-1 revealed that compound **6h** emerged as the most potent counterpart showing IC_50_ = 1.79 µM surpassing the activity of Gefitinib by 5-folds which possessed IC_50_ = 10.24 µM. Thereafter, compounds **6f**, **6i**, **16d**, and **20f** displayed 4-folds superior activity compared to Gefitinib with IC_50_ values = 2.25, 2.48, 2.18, and 2.09 µM, respectively. In addition, compounds **6d**, **6l**, **6n**, **8b**, **12b**, **12c**, **16a**, **16b**, **20b**, and **20e** exerted better inhibitory activity than Gefitinib demonstrating IC_50_ values = 8.15, 6.34, 6.11, 7.34, 8.36, 9.12, 8.22, 8.69, 9.65, and 4.46 µM, respectively. The rest of compounds had week to moderate or no activity relative to Gefitinib.

Concerning HCT-116 cell line, it was found that compounds **6f**, **6h**, **16d**, and **20f** established twice inhibitory activity compared to Gefitinib (IC_50_ = 6.94 µM) displaying IC_50_ values = 3.09, 3.28, 2.43, and 2.96 µM, respectively. Furthermore, compounds **6d**, **6i**, **12c**, **16a**, **16b**, and **20e** exhibited higher growth inhibitory activities than Gefitinib with IC_50_ values = 4.67, 5.68, 6.37, 6.15, 4.22, and 4.75 µM, respectively. The remaining compounds possessed week to moderate or no activity compared to Gefitinib.

It is noteworthy that appending *p*-CF_3_ to the 2-phenyl enhanced the antiproliferative activity compared to *p*-Cl and *p*-CH_3_, except for the cyclohexylamine analogs **6b, 6g**, and **6l** in which the *p*-CH_3_ substituent is preferred for antiproliferative activity. Remarkably, the incorporation of *p*-(2-furyl) to the 2-phenyl abolished the growth inhibitory activity, while the introduction of *p*-(2-thienyl) increased the activity compared to *p*-Cl and *p*-CH_3_ analogs. In the context of impact of amine substituents on the antiproliferative activity, it was proved that 4-amino-*N*-methylpiperidine is preferred for anticancer activity, then piperazine, tetrahydrofurfurylamine and cyclohexylamine. Notably, grafting imidazole along with 6,7-dimethoxy substituents abolished the antiproliferative activity for all derivatives.

Besides, the removal of 6,7-dimethoxy groups from the quinoline scaffold decreased or abolished activity. While the fusion of 1,3-dioxolo to the quinoline scaffold along with *p*-CF_3_ substitution on phenyl dramatically potentiated the anticancer activity of the imidazole derivatives, the replacement of imidazole with morpholine decreased the activity of *p*-CF_3_ analogs and markedly increased the activity of *p*-Cl counterparts. On the other hand, appending of electron withdrawing (Br) to position 6 of quinoline parallel with *p*-Cl substitution on 2-phenyl greatly enhanced the activity of the imidazole derivatives while, *p*-F substitution on the 2-phenyl extremely decreased the anticancer activity of imidazole derivatives. Also, the replacement of imidazole with morpholine almost had no impact on the *p*-Cl analogs, but tremendously elevated the anticancer activity of *p*-F derivatives.

Furthermore, the incorporation of 4-methoxyphenyl or 2-furyl to position 6 of quinoline along with *p*-Cl or F on the 2-phenyl enhanced the activity of the imidazole derivatives compared to the dimethoxy analogs, but the 2-furyl derivatives exhibited better activity. Moreover, the replacement of imidazole with morpholine elevated the antiproliferative activity of *p*-Cl analogs while, diminished the activity of *p*-F derivatives.

Finally, the deduced structure activity relationships indicated that the substitution pattern on positions 6 and 7 of quinoline and position 4 of the 2-phenyl, in addition to the amine substitution on the 4-propoxy linker are crucial elements for the anticancer activity. In general, incorporation of dimethoxy groups at positions 6 and 7 of quinoline along with *p*-CF_3_ at 2-phenyl and 4-amino-*N*-methylpiperidine, piperazine or tetrahydrofurfurylamine on the 4-propoxy linker, in addition to grafting of electron withdrawing (Br) or 2-furyl at position 6 of quinoline along with *p*-F or Cl on the 2-phenyl and morpholine on the propoxy linker resulted in the most potent antiproliferative agents in this study.

#### 2.2.2. Annexin V-FITC/propidium iodide apoptosis assay (AV/PI)

**Figure 4.**
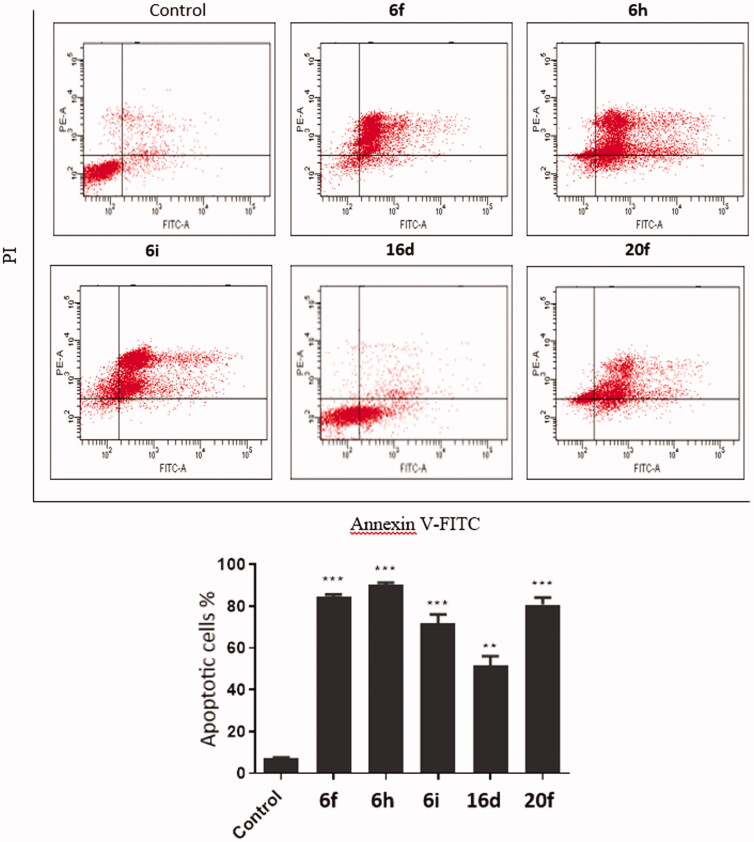
Influence of the promising compounds on the total percentage of AV-FITC positive staining in DLD1 cancer cell line.

The apoptotic impact of the most potent antiproliferative agents **6f, 6h, 6i, 16d**, and **20f** on DLD-1 colorectal cancer cell line was investigated exploiting AV/PI dual staining assay. The assay outcomes proved that the tested compounds elicited apoptosis of such cell line as indicated by significant rise in the total percentage of AV positive apoptotic DLD-1 cells compared to the control (Figure 4). Compound **6h** increased the total percentage of apoptotic cells from 7% for the control to 90.33%. Also, compounds **6f, 6i, 16d**, and **20f** exerted potential apoptotic effect elevating the total percentage of apoptotic cells to 84.33, 71.66, 51.66, and 80.66%, respectively.

### 2.3. Topoisomerase I-mediated DNA cleavage assay

TOP1 poisoning activity of all target compounds has been estimated utilising TOP1-mediated DNA cleavage assay that determines the TOP1 poisoning activity relative to 1 µM Camptothecin (CPT)^37^. The tested compounds were incubated at 0.1, 1, 10, and 100 µM with recombinant human TOP1 enzyme and a 3′-[^32^P]-labeled 117-bp DNA oligonucleotide^38^. TOP1 poisoning agents specifically trap TOP1ccs leading to their stabilisation and DNA cleavage. The drug-induced stabilised TOP1ccs are visualised by gel electrophoresis demonstrating specific DNA cleavage patterns. Then a semiquantitative scoring system by visual comparison between lanes induced by the target compounds and 1 µM CPT was used to score the compounds (Table 3, see Supplementary Data for the results of gel electrophoresis)^39–41^.

**Table 3.** The TOP1 inhibitory activity of all target compounds **6a–o, 8a,b, 10a,b, 12a–d, 16a–d**, and **20a–f** compared to Camptothecin (CPT).

Compounds	TOP1 inhibitory activity^a^	Compounds	TOP1 inhibitory activity
**6a**	−/+	**10a**	0
**6b**	−/+	**10b**	0
**6c**	−/+	**12a**	−/+
**6d**	−/+	**12b**	−/+
**6e**	−/+	**12c**	0
**6f**	0	**12d**	0
**6g**	0	**16a**	0
**6h**	−/+	**16b**	0
**6i**	0	**16c**	+
**6j**	0	**16d**	0
**6k**	0	**20a**	0
**6l**	−/+	**20b**	0
**6m**	−/+	**20c**	0
**6n**	0	**20d**	0
**6o**	0	**20e**	0
**8a**	0	**20f**	0
**8b**	0		

^a^Scoring: 0: no activity; −/+: 0–25% 1** **µM CPT; +: 25–50% 1** **µM CPT.

Compounds **6a–e, 6h, 6l, 6m, 12a**, and **12b** exhibited weak TOP1 poisoning effect (-/+) displaying DNA cleavage activity equal to 0–25% of the activity of 1 µM CPT, while compound **16c** demonstrating activity (+) equals to 25–50% of the activity of 1 µM CPT. The rest of compounds possessed no cleavage activity. Despite the target compounds showing promising antiproliferative activity against cancer cell lines, their TOP1 poisoning activities were not encouraging for further development as potent TOP1 inhibitors. Accordingly, the synthesised compounds have been evaluated for the plausible mechanism by which they provoked the antiproliferative activity.

### 2.4. Kinase inhibitory activities of target quinolines

#### 2.4.1. Kinase profiling

The non-significant TOP1 poisoning activities of target compounds obtained from Topoisomerase I-mediated DNA cleavage assay motivated us to search for the plausible molecular mechanism for herein reported 4-propoxy-2-arylquinolines.

The potential inhibitory activity of the target 4-propoxy-2-arylquinolines (**6a–o**, **8a**,**b**, **10a**,**b**, **12a–d**, **16a–d**) and 4-propoxy-2,6-diarylquinolines (**20a–f**) was explored against a panel of nine kinases representing different signalling pathways; EGFR, FAK, FRK, IGF-1R, BTK, c-Src, VEGFR-1, VEGFR-2 and HER-2 (see Supplementary Data, Table S1). The half maximal inhibitory concentration (IC_50_) values were calculated for each kinase and presented in Table S1 and Table 4. Strikingly, the screening outcomes revealed that the investigated quinolines exhibited promising dual inhibitory effect towards EGFR and FAK kinases.

**Table 4.** The half maximal inhibitory concentration (IC_50_) of all target compounds **6a–o, 8a,b, 10a,b, 12a–d, 16a–d**, and **20a–f** against EGFR and FAK kinase activity compared to Gefitinib and TAE226.

Compounds	IC_50_ (nM)^a^		Compounds	IC_50_ (nM)	
	EGFR	FAK		EGFR	FAK
**6a**	142.64** **±** **2.54	214.36** **±** **1.09	**10b**	179.64** **±** **9.12	164.74** **±** **5.37
**6b**	45.26** **±** **5.36	98.16** **±** **4.67	**12a**	124.97** **±** **7.94	225.46** **±** **14.02
**6c**	85.67** **±** **6.46	45.70** **±** **3.40	**12b**	95.36** **±** **2.05	70.85** **±** **3.16
**6d**	46.37** **±** **4.09	36.97** **±** **2.34	**12c**	246.70** **±** **12.29	111.06** **±** **8.94
**6e**	156.72** **±** **11.36	211.08** **±** **8.96	**12d**	450.16** **±** **4.25	273.16** **±** **2.84
**6f**	25.39** **±** **3.49	22.68** **±** **2.38	**16a**	222.15** **±** **8.25	44.15** **±** **3.26
**6g**	365.49** **±** **14.82	145.71** **±** **10.54	**16b**	313.34** **±** **15.34	50.36** **±** **4.81
**6h**	20.15** **±** **1.07	14.25** **±** **2.72	**16c**	485.46** **±** **11.37	224** **±** **10.46
**6i**	22.36** **±** **2.05	18.36** **±** **3.17	**16d**	35.03** **±** **2.64	17.36** **±** **2.15
**6j**	258.34** **±** **11.94	186.46** **±** **6.22	**20a**	121.74** **±** **9.40	77.25** **±** **4.37
**6k**	410.38** **±** **12.73	157.84** **±** **8.73	**20b**	245.11** **±** **12.34	63.25** **±** **3.25
**6l**	34.91** **±** **3.76	26.37** **±** **2.81	**20c**	362.30** **±** **5.26	91.03** **±** **5.85
**6m**	154.29** **±** **12.80	172.49** **±** **13.67	**20d**	146.95** **±** **8.37	125.38** **±** **3.15
**6n**	41.82** **±** **2.34	44.36** **±** **2.94	**20e**	33.65** **±** **1.02	25.36** **±** **3.48
**6o**	256.19** **±** **6.94	204.84** **±** **8.04	**20f**	24.81** **±** **2.71	15.36** **±** **0.98
**8a**	349.37** **±** **14.05	198.32** **±** **12.32	Gefitinib	48.52** **±** **3.64	–
**8b**	35.48** **±** **1.50	29.79** **±** **2.37	TAE226	–	4.60** **±** **0.94
**10a**	244.30** **±** **8.41	298.74** **±** **1.94			

^a^IC_50_ values are the mean of three separate experiments ± *SD*.

#### 2.4.2. EGFR and FAK kinase inhibitory activity

All the newly prepared 4-propoxy-2-arylquinolines (**6a–o**, **8a**,**b**, **10a**,**b**, **12a–d**, and **16a–d**) and 4-propoxy-2,6-diarylquinolines (**20a–f**) were examined for their potential EGFR and FAK inhibitory activities. Gefitinib and TAE-226 were used as reference EGFR and FAK inhibitors, respectively. The results are reported as half maximal inhibitory concentration values (IC_50_), as determined from triplicate measurements and are presented in Table 4.

Results in Table 4 revealed that the examined quinolines displayed moderate to potent inhibitory activity towards EGFR (IC_50_ values ranging between 20.15 ± 1.07 and 485.46 ± 11.37 nM, Table 4). In particular, trifluoromethyl phenyl-bearing 6,7-dimethoxy-2-arylquinolines **6f**, **6h**, and **6i**, as well as 6-furanyl-2-arylquinoline **20f** emerged as the most efficient EGFR inhibitors with two-digits nanomolar IC_50_s (IC_50_ = 25.39 ± 3.49, 20.15 ± 1.07, 22.36 ± 2.05, and 24.81 ± 2.71 nM, respectively). Notably, these four derivatives displayed 2-fold higher activity than the reference EGFR inhibitor Gefitinib (IC_50_ = 48.52 ± 3.64 nM). In addition, compounds **6b**, **6d**, **6l**, **6n**, **8b**, **16d**, and **20e** exhibited potent EGFR inhibitory activity, as the measured IC_50_ values ranged between 33.65 ± 1.02 and 46.37 ± 4.09 nM, which are slightly improved or comparable to that of the reference drug Gefitinib (Table 4). Moreover, compounds **6c** and **12b** showed 2-fold decreased activity (IC_50_ = 85.67 ± 6.46 and 95.36 ± 2.05 nM, respectively) than Gefitinib against EGFR. The remaining examined quinolines possessed moderate EGFR inhibitory activity (IC_50_ range: 121.74 ± 9.40–485.46 ± 11.37 nM) compared to Gefitinib (Table 4). Strikingly, the inclusion of 4-amino-*N*-methylpiperidine, tetrahydrofurfurylamine and *N*-methylpiperazine along with 2-(*p*-CF_3_ phenyl) and 6,7-dimethoxy substituents (**6f, 6h**, and **6i**), in addition to the grafting of morpholine together with 2-(*p*-Cl phenyl) and 6–(2-furyl) **20f** afforded the most potent EGFR inhibitors in this study displaying IC_50_ range from 20.15 ± 1.07 to 25.39 ± 3.49 nM.

On the other hand, as depicted in Table 4, FAK kinase was efficiently inhibited by all 4-propoxy-2-arylquinolines (**6a–o, 8a,b, 10a,b, 12a–d**, and **16a–d**) and 4-propoxy-2,6-diarylquinolines (**20a–f**) herein reported in the nanomolar range (IC_50_ range: 14.25 ± 2.72–298.74 ± 1.94 nM). Superiorly, *p*-CF_3_-phenyl-bearing 6,7-dimethoxy-2-arylquinolines **6f**, **6h**, and **6i**, as well as, morpholine-bearing 6-bromo-2-arylquinoline **16d** and 6-furanyl-2-arylquinoline **20f** were the most potent FAK inhibitors in this study with IC_50_ values equal 22.68 ± 2.38, 14.25 ± 2.72, 18.36 ± 3.17, 17.36 ± 2.15, and 15.36 ± 0.98 nM, respectively (Table 4). Moreover, compounds **6c**, **6d**, **6l**, **6n**, **8b**, **16a**, **16b**, and **20e** exerted potent FAK inhibitory activity with IC_50_ spanning in the range 25.36 ± 3.48–50.36 ± 4.81 nM.

Further analysis of the obtained results in Table 4 revealed that compounds **6b**, **12b**, and **20a–c** exhibited two-digit nanomolar IC_50_s; 98.16 ± 4.67, 70.85 ± 3.16, 77.25 ± 4.37, 63.25 ± 3.25, and 91.03 ± 5.85 nM, respectively, whereas the remaining derivatives displayed moderate inhibitory activity against FAK kinase (IC_50_ range: 111.06 ± 8.94–298.74 ± 1.94 nM) (Table 4). Interestingly, the incorporation of 4-amino-*N*-methylpiperidine, tetrahydrofurfurylamine and *N*-methylpiperazine along with 2-(*p*-CF_3_ phenyl) and 6,7-dimethoxy substituents (**6f, 6h**, and **6i**), besides the appending of morpholine with 2-(*p*-F phenyl) and 6-Br **16d**, as well as the addition of morpholine in conjunction with 2-(*p*-Cl phenyl) and 6–(2-furyl) **20f** provided the most potent FAK inhibitors in this study demonstrating IC_50_ values ranging from 14.25 ± 2.72 to 22.68 ± 2.38 nM.

It is worth stressing that 4-propoxy-2-arylquinolines **6f**, **6h**, **6i**, and **20f** emerged not only as the most potent dual EGFR/FAK inhibitors in this study, but also as the most efficient anti-proliferative agents towards the examined colorectal cancer (DLD-1 and HCT-116) cell lines.

### 2.5. *In silico* molecular docking

#### 2.5.1. Docking into EGFR binding site

**Figure 5.**
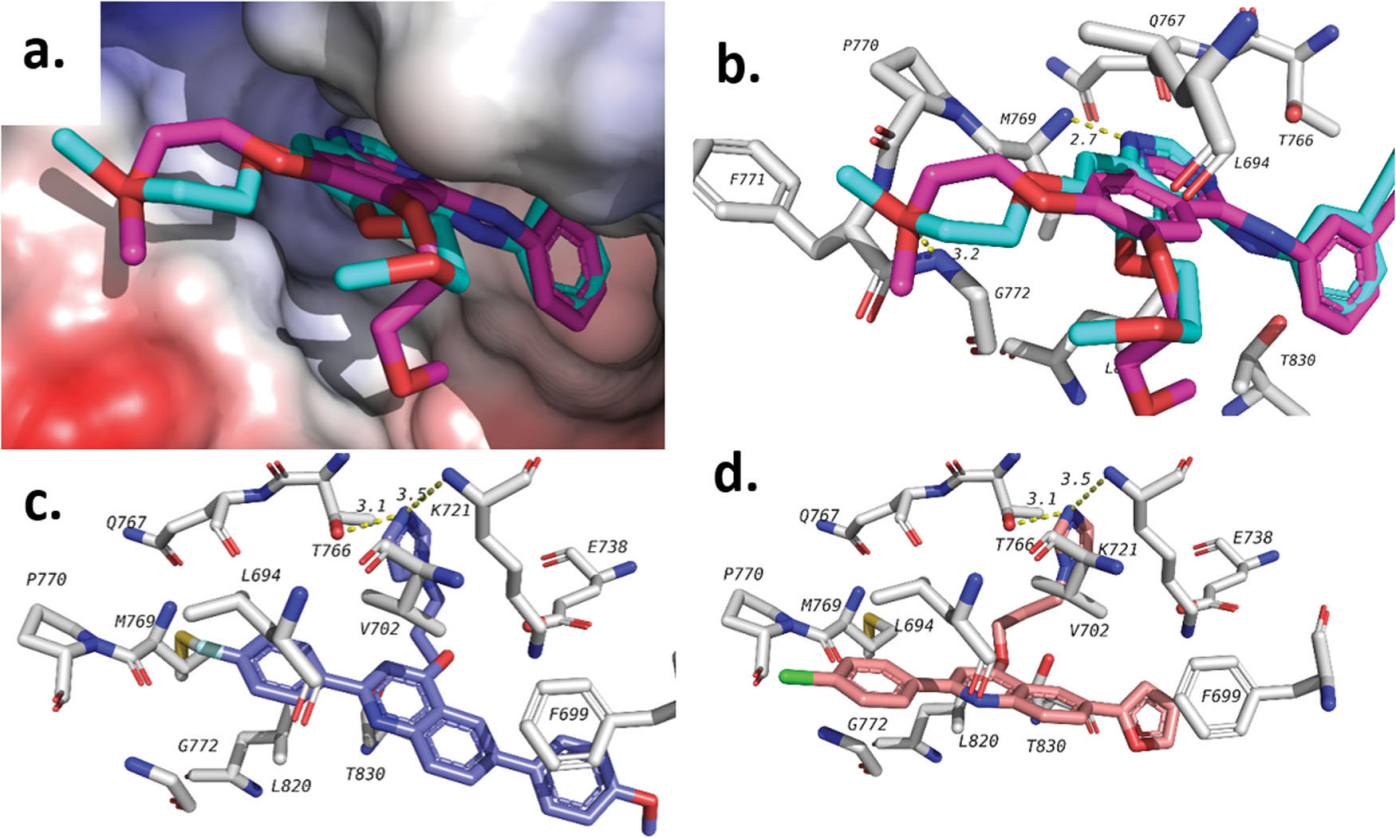
Docking of target quinolines in the active site EGFR. (a) validation of docking procedure showing overlapping of crystalised (blue) and docked (pink) poses; (b) interactions of Erlotinib with EGFR; (c) docking pose of **20c**; (d) docking pose of **20e**.

The molecular docking approach was utilised to investigate the potential binding of herein reported 4-propoxy-2-arylquinolines to EGFR binding site (PDB: 1M17). The docking procedure was validated through the redocking of the co-crystalised ligand. The correct pose was predicted accurately with RMSD of 1.498 between the docked and co-crystalised ligand using DockRMSD server (Figure 5(a))^42^. In addition, docking was able to maintain hydrogen bonding seen in the co-crystalised ligand with NH of M769 (2.7 Å) and with the NH of G772 (3.2 Å). Furthermore, hydrophobic interactions with residues in the active site were also maintained. These include interactions between K721 and ethyne benzene moiety, and L694, L768, and L820 with hydrophobic part of the quinazoline ring (Figure 5(b)).

Docking scores of the tested compounds with EGFR are shown in Table 5. The docking score of the co-crystalised ligand was −7.2 kcal/mol, whereas all the tested quinolines have shown better docking scores than that of the co-crystalised ligand (−7.9 to −9.7 kcal/mol). Best docking scores were seen with quinolines form the series **20**. Compound **20c** showed the best binding energy to EGFR with docking score of −9.7 kcal/mol and its binding pose is shown in Figure 5(c). The compound has formed 2 hydrogen bonds between imidazole ring and both K721 (3.5 Å) and T766 (3.1 Å). Also, several hydrophobic interactions have been seen, which included interactions between quinoline ring and side chains of V702 and T830. The phenyl ring at position 2 formed hydrophobic interactions with L694 and L820 which are also common with the co-crystalised ligand. In addition, π- π stacking between the ring at quinoline position 6 and F699.

**Table 5.** Docking results of target compounds with EGFR and FAK.

Compound	Docking Score (kcal/mol)	
	EGFR (PDB: 1M17)	FAK (PDB: 2JKM)
**6a**	−8.1	−7.7
**6b**	−8.5	−7.3
**6c**	−8.2	−7.5
**6d**	−7.9	−7
**6e**	−8.4	−7.6
**6f**	−8.2	−7.4
**6g**	−8.5	−7.6
**6h**	−8.2	−7.6
**6i**	−8.1	−7.3
**6j**	−8.6	−8.0
**6k**	−8.1	−7.7
**6l**	−8.7	−7.4
**6m**	−8.3	−7.5
**6n**	−8.2	−7.1
**6o**	−8.4	−7.7
**8a**	−8.3	−7.6
**8b**	−8.4	−7.5
**10a**	−8.8	−7.9
**10b**	−8.3	−8.0
**12a**	−8.5	−8.0
**12b**	−8.5	−8.3
**12c**	−8.8	−8.4
**12d**	−8.7	−8.3
**16a**	−8.1	−7.7
**16b**	−8.2	−8.0
**16c**	−8.3	−7.7
**16d**	−8.3	−7.9
**20a**	−9.3	−8.3
**20b**	−9.1	−8.2
**20c**	−9.7	−8.3
**20d**	−9.2	−8.2
**20e**	−9.3	−8.2
**20f**	−8.8	−7.7
Co-crystalised ligand	−7.2 (Erlotinib)	−8.5 (AZW592)

Another compound from this series is compound **20e** which was selected as a representative example because it has shown good biological results with both EGFR and FAK. The docking pose of this compound is shown in Figure 5(d) showing a similar docking pose to **20c**. The same hydrogen bonds with K721 (3.5 Å) and T766 (3.1 Å) were maintained as well as hydrophobic interactions with V702 and T830 as well as with L694 and L820. The π–π stacking was also seen between F699 and the furan ring of **20e**. This compound, in complex with EGFR, was subjected to further investigation using molecular dynamics to study the stability of its complex with EGFR as will be discussed later.

#### 2.5.2. Docking into FAK binding site

**Figure 6.**
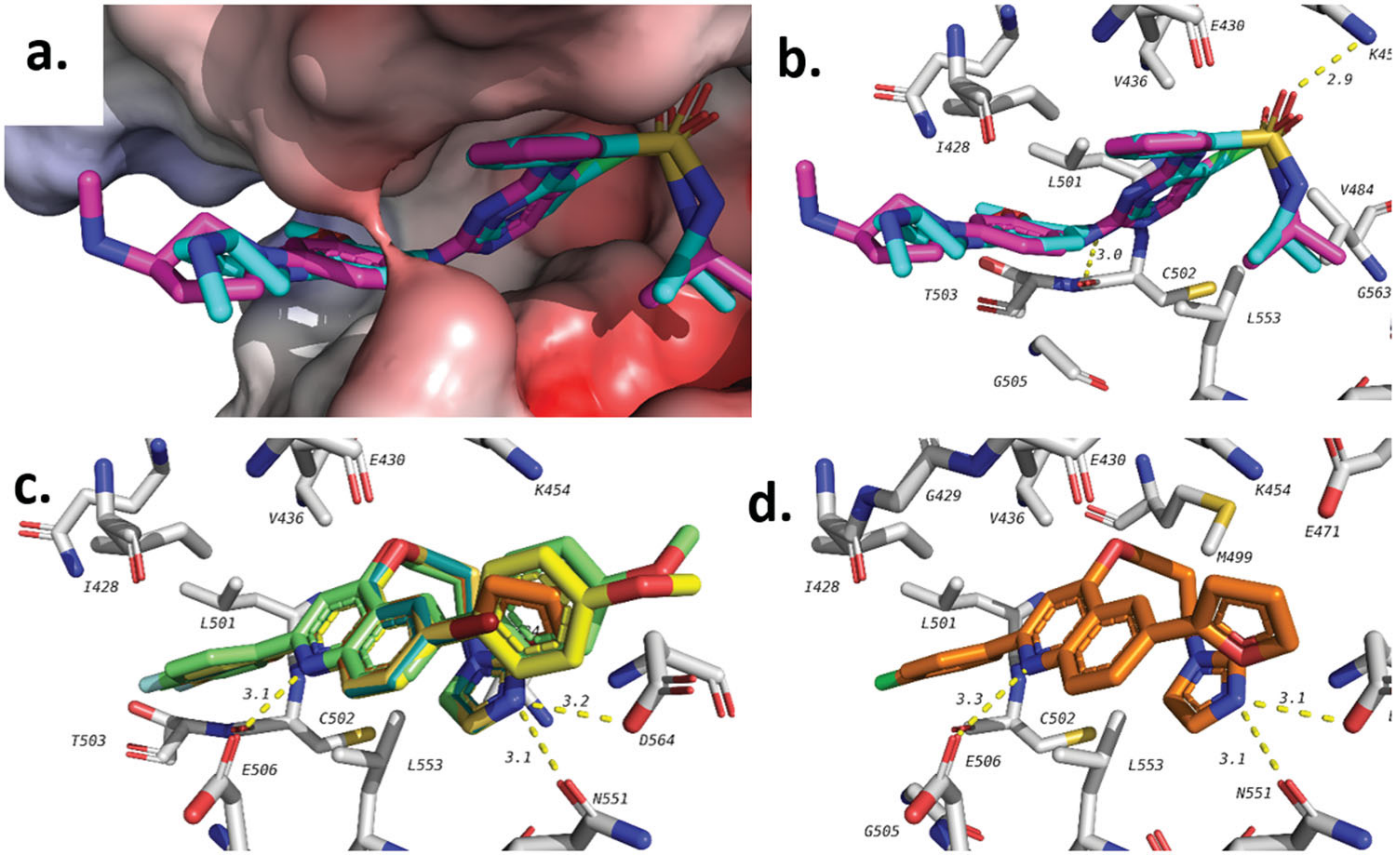
Docking of target compounds in the active site FAK. (a) validation of docking procedure showing overlapping of crystalised (blue) and docked (pink) poses; (b) interactions of AZW592 with FAK; (c) general binding of **16** and **20** compound series; (d) docking pose of **20e**.

Potential binding of target quinolines to FAK was also investigated using docking studies (**PDB**: 2JKM). Initially, the docking procedure was validated through the redocking of the co-crystalised ligand (AZW592). The docking searching algorithm was able to correctly predict the binding pose with acceptable accuracy with RMSD of 1.318 between the docked and co-crystalised ligands as predicted by DockRMSD server (Figure 6(a))^42^. The docked structure was able to maintain same hydrogen bonds that are in the crystal structure including those between the sulphamoyl moiety oxygen and the terminal amino group of K454 (Å) and the hydrogen bond with the α-carbonyl group of *C*502. In addition, several hydrophobic interactions have been also seen with residues in the active site including I428, V436, V484, L501, and L553 (Figure 6(b)).

Next, target synthesised quinolines were docked in the active site of the FAK after their preparation. The docking scores of tested compounds are shown in Table 5. The docking score of co-crystalised ligand (AZW592) was found to be −8.5 kcal/mol. Some of tested compounds have shown scores that are comparable to the co-crystalised ligand. Best results were seen with **12** and **20** and some of the **16** series. Docking poses of these compounds were similar with most of the docked compounds as can be seen in Figure 6(c) which shows docking pose of some compounds from these series.

Docking pose of compound **20e** which was chosen as representative example is shown in Figure 6(d). The compound was able to form 3 hydrogen bonds with N551 (3.1 Å), D564 (3.1 Å), and E506 (3.3 Å). In addition, several hydrophobic interactions were also seen, such as the hydrophobic interaction between quinoline ring and L553 and between phenyl ring at position 2 and I428 which are common with the co-crystallized ligand. This pose was selected for further investigation of the complex stability using molecular dynamics study.

### 2.6. Molecular dynamics (MD) simulation

The stability of compound **20e** complexes with both EGFR and FAK was investigated using 100 ns molecular dynamics studies. With each complex, the results were compared with the co-crystalised ligand complex as a control and with the apoprotein (the protein alone with no ligands). The missing loops in both targets were built using Swiss-Model server^43^ before starting the dynamics to ensure correct results. All complexes were equilibrated under NVT then NPT conditions for 1 ns each and the analysis was done on the production run.

**Figure 7.**
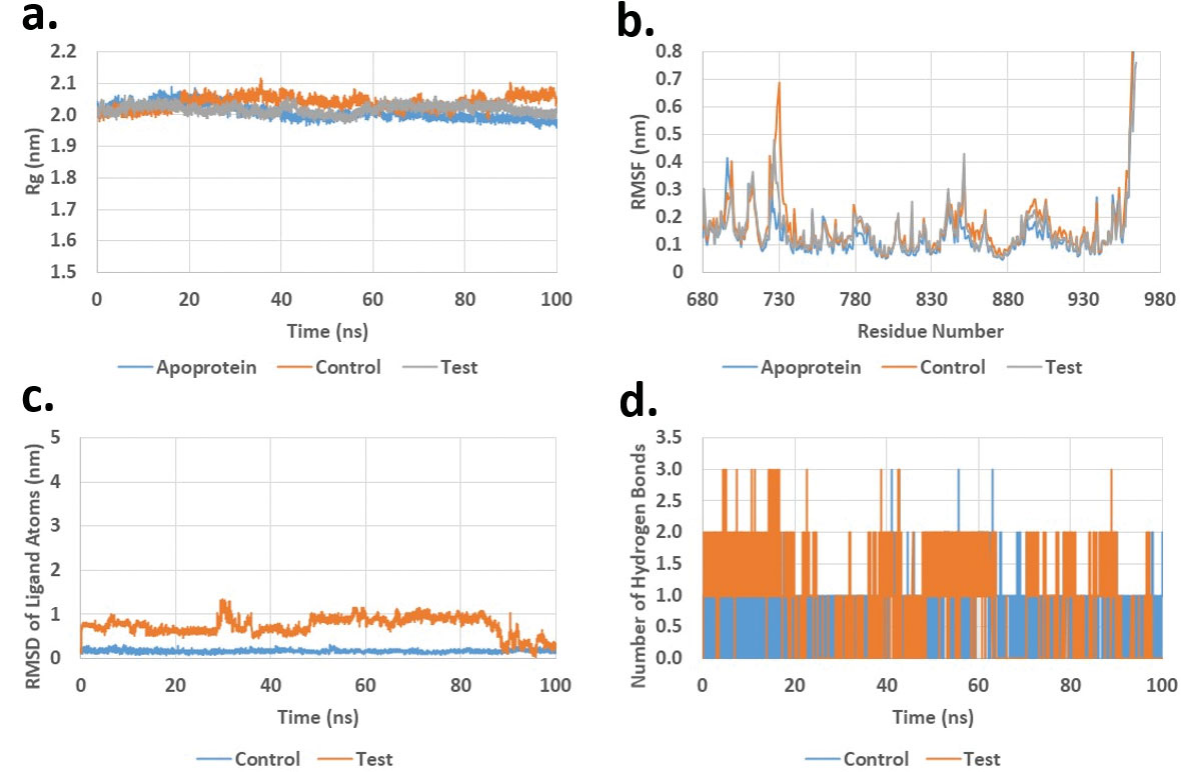
Molecular dynamics analysis of the production run trajectory of **20e** in the active site of EGFR compared to control and apoprotein. (a) Radius of gyration; (b) Root mean square fluctuation of residues; (c) Root mean square deviation of ligand heavy atoms; (d) Number of hydrogen bonds between ligand and protein.

Analysis of the production runs trajectories for **20e** in the active site of EGFR demonstrated stability comparable to the co-crystalised ligand. Radius of gyration (*R_g_*) is a measure of the compactness of the complexes. Stable *R_g_* suggested the stability of the protein or complex under investigation. Figure 7(a) shows a plot of *R_g_* of **20e**, co-crystalised ligand and apoprotein. The average R_g_ was found to be 2.01 ± 0.02, 2.03 ± 0.02, and 2.02 ± 0.01 nm for apoprotein, control, and **20e**, respectively. In addition, Root mean square fluctuation (RMSF) of protein residue (Figure 7(b)) for all the three complexes showing similar patterns. The average RMSF for co-crystalised ligand and **20e** was found to be 0.19 and 0.18 nm, respectively which is slightly higher than that of the apoprotein (0.15 nm). Although root mean square deviation (RMSD) of ligand heavy atoms for **20e** is slightly higher than that of the co-crystalised ligand (Figure 7(c)), the value is <1 nm for most of the trajectory. This value cannot be calculated for the apoprotein as it has no ligand in the system. Finally, the number of hydrogen bonds between ligands and protein are shown in Figure 7(d), which showed that **20e** formed extra hydrogen bonds during at least 50% of the production run time. These results suggested that **20e** complex with EGFR is at least of comparable stability when compared to the complex with the co-crystalised ligand; Erlotinib.

**Figure 8.**
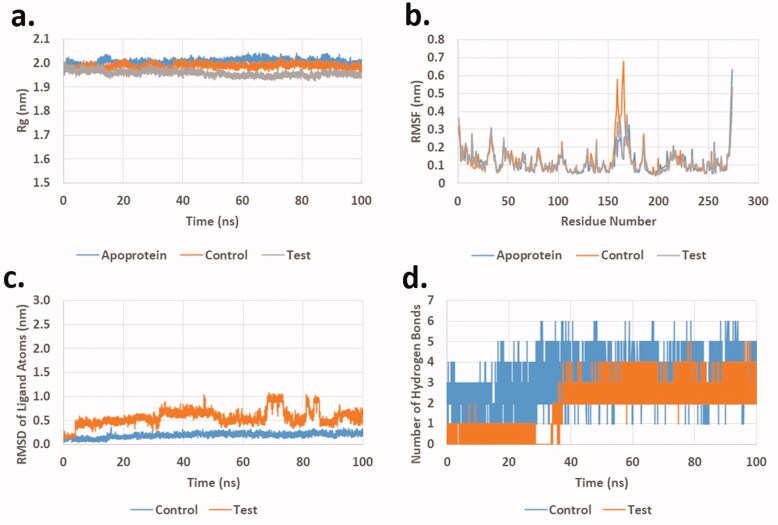
Molecular dynamics analysis of the production run trajectory of **20e** in the active site of FAK compared to control and apoprotein. (a) Radius of gyration; (b) Root mean square fluctuation of residues; (c) Root mean square deviation of ligand heavy atoms; (d) Number of hydrogen bonds between ligand and protein.

Complexes of FAK with **20e**, its co-crystalised ligand and the apoprotein showed similar pattern to that of the EGFR (Figure 8). This includes the radius of gyration (R_g_), which showed an average of 2.00 ± 0.01, 1.99 ± 0.01, and 1.96 ± 0.01 nm for apoprotein, co-crystalised ligand, and **20e**, respectively (Figure 8(a)). Also, RMSF of protein residues was found to follow similar patterns for all the three studied systems (Figure 8(b)). The average RMSF for the three systems was found to be 0.12 ± 0.07, 0.13 ± 0.09, and 0.12 ± 0.07 nm for apoprotein, co-crystalised ligand, and **20e**, respectively. In addition, plotting of RMSD of ligand heavy atoms (Figure 8(c)) showed minimal fluctuation for both with and average RMSD of 0.19 ± 0.05 and 0.57 ± 0.16 nm for co-crystalised ligand and **20e**, respectively. Although, the value for **20e** is higher but it is within acceptable range (<1 nm). Finally, plotting of hydrogen bonds between ligands and target protein (Figure 8(d)) showed that the number of hydrogen bonds is higher in case of the co-crystalised ligand compared to **20e**. Being said, **20e** was still able to maintain an average of 1.93 ± 0.45 hydrogen bonds during the 100 ns production run.

These results collectively suggested the stability of compound **20e** complexes with both EGFR and FAK compared to the corresponding co-crystalised ligands in each target. This in general supported the dual mechanism similar to the enzymatic inhibition data.

## 3. Conclusion

Different series of 4-propoxy-2-arylquinolines (**6a–o, 8a,b, 10a,b, 12a–d**, and **16a–d**) and 4-propoxy-2,6-diarylquinolines (**20a–f**) have been designed and synthesised as potential anticancer agents. The quinolines **6f**, **6h**, **6i**, **16d**, and **20f** demonstrated the most potent antiproliferative effect against DLD-1 colorectal cancer with respective IC_50_ values = 2.25, 1.79, 2.48, 2.18, and 2.09 µM with 4- to 5-folds potency compared to Gefitinib (IC_50_ = 10.24 µM). Additionally, compounds **6f**, **6h**, **16d**, and **20f** possessed twice growth inhibitory impact as Gefitinib (IC_50_ = 6.94 µM) displaying IC_50_ values = 3.09, 3.28, 2.43, and 2.96 µM against HCT-116 cell line, respectively. Moreover, compounds **6f**, **6h**, **6i**, **16d**, and **20f** significantly elevated the total percentage of DLD-1 apoptotic cells. Furthermore, the quinolines **6f**, **6h**, **6i**, and **20f** exerted potent EGFR inhibitory effects with IC_50_ values = 25.39 ± 3.49, 20.15 ± 1.07, 22.36 ± 2.05, and 24.81 ± 2.71 nM, respectively compared to Gefitinib (IC_50_ = 48.52 ± 3.64 nM). In a similar fashion, the quinolines **6f**, **6h**, **6i**, **16d**, and **20f** displayed the best FAK inhibitory actions with IC_50_ values = 22.68 ± 2.38, 14.25 ± 2.72, 18.36 ± 3.17, 17.36 ± 2.15, and 15.36 ± 0.98 nM, respectively. Molecular docking and molecular dynamics simulation rationalised EGFR/FAK dual inhibition providing different quinolines being as the first reported quinolines possessing potential EGFR/FAK dual inhibition. The latter compounds can be used as lead compounds for the development of more potent EGFR/FAK dual inhibitors as potential anticancer agents.

## 4. Experimental

### 4.1. Chemistry

#### 4.1.1. General

Melting points have been measured by Yanaco melting point device and were uncorrected. NMR spectra were measured using Bruker Advance III HD at 400 MHz for ^1^H NMR, 100 MHz for ^13^C NMR and 376 MHz for ^19^F NMR in deuterated CDCl_3_ or DMSO-*d*_6_ using tetramethyl silane (TMS) as an internal standard. Coupling constant values (*J*) were determined in Hertz (Hz) and chemical shifts (*δ*) were expressed in *ppm*. High resolution mass spectrometry (HRMS) have been measured by Thermo Fisher Scientific LTQ Orbitrap XL spectrophotometer using electrospray ionisation (ESI) and the results were expressed as [M + H]^+^ or [M + Na]^+^ at Natural Science Research and Development Centre, Hiroshima University, Japan. The purities of all biologically tested compounds were determined by HPLC and were found to be ≥95%. HPLC analysis was performed utilising JASCO 880-PU HPLC system (Japan spectroscopic Co. Ltd) connected to a diode array detector with detection at a wavelength of 254 nm. The column exploited in the HPLC analysis was Inertsil ODS-3 column with dimensions of 250 × 4.6 mm and 5 µm particle size (GL SCIENCES INC., Japan). The mobile phase employed for HPLC analysis was acetonitrile/water/TFA (29.9/70/0.1, v/v) at a flow rate of 0.5 ml/min. The reactions have been monitored by thin layer chromatography (TLC) using Merck silica gel 60F_254_ aluminium sheets. Column chromatography has been performed utilising silica gel 60 N, 63–210 µm that was purchased from Kanto Chemical Co. Inc., Japan using dichloromethane/methanol (100/0 to 90/10, v/v) and hexane/ethylacetate (100/0 to 90/10, v/v). Unless otherwise stated, all chemicals and solvents were available commercially and have been used without further purification.

#### 4.1.2. Synthesis of 1-(2-amino-4,5-dimethoxyphenyl)ethan-1-one (1)

2′-Aminoacetophenone derivative **1** was prepared using the reported method^44^^,^^45^.

Yellow solid, yield 70%, m.p. 100–102 °C; ^1^H NMR (400 MHz, CDCl_3_) *δ* (*ppm*): 2.53 (s, 3H, CH_3_), 3.85 (s, 3H, OCH_3_), 3.89 (s, 3H, OCH_3_), 6.12 (s, 1H, phenyl CH), 6.27 (br s, 2H, NH_2_), 7.12 (s, 1H, phenyl CH).

#### 4.1.3. General procedure for the synthesis of benzamides (3a–c)

In dry THF (8 ml) and Et_3_N (2 ml), 1–(2-amino-4,5-dimethoxyphenyl)ethan-1-one **1** (0.976 g, 5 mmol) was dissolved and cooled in ice bath. Then, a solution of the respective *p*-substituted benzoyl chloride **2a–c** (5.1 mmol) in dry THF (2 ml) was added dropwise while cooling in ice bath. The reaction mixture was stirred in ice bath for 30 min and then overnight at room temperature. After that, reaction mixture was poured into ice/water and the resulting solid was filtered off and washed excessively with water and methanol to afford the corresponding **3a–c**.

The spectral characterisation of the benzamides **3a,b** were reported in our previous study^36^.

##### 4.1.3.1. N-(2-acetyl-4,5-dimethoxyphenyl)-4-methylbenzamide (3c)

White solid, yield 70%, m.p. 165–167 °C; ^1^H NMR (400 MHz, CDCl_3_) *δ* (*ppm*): 2.42 (s, 3H, CH_3_), 2.65 (s, 3H, CH_3_C=O), 3.91 (s, 3H, OCH_3_), 4.02 (s, 3H, OCH_3_), 7.29 (d, 2H, benzoyl 2CH, *J* = 8.1 Hz), 7.30 (s, 1H, phenyl CH), 7.96 (d, 2H, benzoyl 2CH, *J* = 8.1 Hz), 8.77 (s, 1H, phenyl CH), 12.93 (s, 1H, NH); ^13 ^C NMR (100 MHz, CDCl_3_) *δ* (*ppm*): 21.48, 28.37, 56.23, 56.43, 103.52, 113.90, 114.52, 127.45, 129.48, 132.01, 138.49, 142.49, 143.57, 154.89, 166.19 (C=O), 201 (C=O); HRESIMS (*m/z*): [M + H]^+^ Calcd for C_18_H_20_NO_4_, 314.13868; found, 314.13901.

#### 4.1.4. General procedures for synthesis of the quinolones (4a–c)

Under N_2_ atmosphere, the benzamides **3a–c** and three equivalents NaOH were refluxed in dry dioxane at 110 °C for 4 h and then cooled to room temperature. Small amount of water and excess amount of hexane were added to the reaction mixture. The resulting mixture was subjected to sonication for 2 min and then neutralised using 1 M HCl. The separated solid was filtered off and washed excessively with water to give the corresponding quinolones **4a–c**.

The spectral data of the quinolones **4a,b** have been reported in our previous study^36^.

##### 4.1.4.1. 6,7-dimethoxy-2-(p-tolyl)quinolin-4(1H)-one (4c)

Yellow solid, Yield 92%, m.p. > 250** **°C; ^1^H NMR (400** **MHz, DMSO-*d6*) *δ* (*ppm*): 2.43 (s, 3H, CH_3_), 3.93 (s, 3H, OCH_3_), 3.96 (s, 3H, OCH_3_), 7.03 (s, 1H, vinyl CH), 7.47 (d, 2H, *p*-toluoyl 2CH, *J*** **=** **8.1** **Hz), 7.51 (s, 1H, phenyl CH), 7.62 (s, 1H, phenyl CH), 7.84 (d, 2H, *p*-toluoyl 2CH, *J*** **=** **8.1** **Hz); ^13^** **C NMR (100** **MHz, DMSO-*d4*) *δ* (*ppm*): 21.43, 56.39, 56.62, 100.57, 102.37, 104.13, 115.90, 128.22, 130.19, 130.34, 137.47, 142.04, 149.30, 151.20, 155.22, 170. 10 (C=O); HRESIMS (*m/z*): [M** **+** **H]^+^ Calcd for C_18_H_18_NO_3_, 296.12812; found, 296.12827.

#### 4.1.5. General procedure for synthesis of the key intermediates (5a–c)

The quinolones **4a–c** (3** **mmol), KI (0.498** **g, 3** **mmol) and KOH (1.009** **g, 18** **mmol) were stirred for 2** **h in dry DMF (30** **ml) and then 1-bromo-3-chloropropane (2.361** **g, 15** **mmol) was added to the mixture and stirred at room temperature for 24** **h. Thereafter, the mixture was poured into ice/water and the separated solid was filtered off and washed with water then hexane to furnish the key intermediates **5a–c** which were used without further purification.

The spectral data of **5a,b** have been reported in our previous study^36^.

##### 4.1.5.1. 4-(3-chloropropoxy)-6,7-dimethoxy-2-(p-tolyl)quinoline (5c)

White solid, Yield 90%, m.p. 140–142** **°C; ^1^H NMR (400** **MHz, CDCl_3_) *δ* (*ppm*): 2.42 (s, 3H, CH_3_), 2.43 (p, 2H, CH_2_, *J*** **=** **6.1** **Hz), 3.83 (t, 2H, CH_2_Cl, *J*** **=** **6.2** **Hz), 4.02 (s, 3H, OCH_3_), 4.03 (s, 3H, OCH_3_), 4.42 (t, 2H, CH_2_-O, *J*** **=** **6** **Hz), 7.10 (s, 1H, vinyl CH), 7.30 (d, 2H, *p*-toluoyl 2CH, *J*** **=** **8.1** **Hz), 7.36 (s, 1H, phenyl CH), 7.44 (s, 1H, phenyl CH), 7.96 (d, 2H, *p*-toluoyl 2CH, *J*** **=** **8.1** **Hz); ^13^** **C NMR (100** **MHz, CDCl_3_) *δ* (*ppm*): 21.30, 32.01, 41.40, 56.05, 56.11, 64.81, 97.57, 99.58, 108.37, 114.64, 127.14, 129.43, 137.66, 138.87, 146.28, 148.88, 152.61, 156.97, 160.56; HRESIMS (*m/z*): [M** **+** **H]^+^ Calcd for C_21_H_23_ClNO_3_, 372.13610; found, 372.13623.

#### 4.1.6. General procedure for synthesis of the target 4-propoxy-2-arylquinolines (6a–o)

To a stirred mixture of **5a–c** (1** **mmol), anhydrous K_2_CO_3_ (1.38** **g, 10** **mmol) and KI (0.83** **g, 5** **mmol) in dry DMF (20** **ml), the respective amine (10** **mmol) was added. Then, the mixture was refluxed at 90** **°C for 12** **h and poured into ice/water (50** **ml). The separated solid was filtered off then washed with water and hexane. The products were purified by silica gel column chromatography using DCM/MeOH to furnish the pure target compounds **6a–o**.

##### 4.1.6.1. 2-(4-chlorophenyl)-6,7-dimethoxy-4–(3-(4-methylpiperazin-1-yl)propoxy)quinoline (6a)

White solid, Yield 71%, m.p. 140–142** **°C; ^1^H NMR (400** **MHz, CDCl_3_) *δ* (*ppm*): 2.15 (p, 2H, CH_2_, *J*** **=** **6.8** **Hz), 2.29 (s, 3H, CH_3_-N), 2.36–2.66 (br s, 8H, piperazinyl 4CH_2_), 2.62 (t, 2H, CH_2_-N, *J*** **=** **7.3** **Hz), 4.01 (s, 3H, OCH_3_), 4.02 (s, 3H, OCH_3_), 4.31 (t, 2H, CH_2_-O, *J*** **=** **6.3** **Hz), 7.03 (s, 1H, vinyl CH), 7.37 (s, 1H, phenyl CH), 7.40 (s, 1H, phenyl CH), 7.44 (d, 2H, chlorophenyl 2CH, *J*** **=** **8.6** **Hz), 7.99 (d, 2H, chlorophenyl 2CH, *J*** **=** **8.6** **Hz); ^13^** **C NMR (100** **MHz, CDCl_3_) *δ* (*ppm*): 26.59, 46.02, 53.31, 55.11, 56.04, 56.11, 66.65, 97.38, 99.68, 108.23, 114.93, 128.55, 128.82, 134.90, 139.00, 146.18, 149.07, 152.72, 155.59, 161.08; HRESIMS (*m/z*): [M** **+** **H]^+^ Calcd for C_25_H_31_ClN_3_O_3_, 456.20485; found, 456.20474; HPLC purity: 97.65%.

##### 4.1.6.2. N-(3-((2–(4-chlorophenyl)-6,7-dimethoxyquinolin-4-yl)oxy)propyl)cyclohexanamine (6b)

Grey solid, Yield 80%, m.p. 118–120** **°C; ^1^H NMR (400** **MHz, CDCl_3_) *δ* (*ppm*): 1.02–1.13 (m, 2H, cyclohexyl 2C*H*H′), 1.14–1.19 (m, 1H, cyclohexyl C*H*H′), 1.20–1.30 (m, 2H, cyclohexyl 2C*H*H′), 1.44 (br s, 1H, NH), 1.59–1.63 (m, 1H, cyclohexyl CH*H*′), 1.70–1.74 (m, 2H, cyclohexyl 2CH*H*′), 1.89–1.92 (m, 2H, cyclohexyl 2CH*H*′), 2.14 (p, 2H, CH_2_, *J*** **=** **6.5** **Hz), 2.43–2.50 (m, 1H, cyclohexyl CH-NH), 2.93 (t, 2H, CH_2_-NH, *J*** **=** **6.9** **Hz), 4.02 (s, 3H, OCH_3_), 4.03 (s, 3H, OCH_3_), 4.35 (t, 2H, CH_2_-O, *J*** **=** **6.1** **Hz), 7.06 (s, 1H, vinyl CH), 7.39 (s, 1H, phenyl CH), 7.41 (s, 1H, phenyl CH), 7.45 (d, 2H, chlorophenyl 2CH, *J*** **=** **8.6** **Hz), 8 (d, 2H, chlorophenyl 2CH, *J*** **=** **8.6** **Hz); ^13^** **C NMR (100** **MHz, CDCl_3_) *δ* (*ppm*): 25.06, 26.14, 30.00, 33.68, 43.90, 56.05, 56.12, 56.91, 66.94, 97.42, .99.67, 108.23, 114.94, 128.55, 128.82, 134.90, 138.98, 146.18, 149.07, 152.71, 155.61, 161.07; HRESIMS (*m/z*): [M** **+** **H]^+^ Calcd for C_26_H_32_ClN_2_O_3_, 455.20960; found, 455.20932; HPLC purity: 98.81%.

##### 4.1.6.3. N-(3-((2–(4-chlorophenyl)-6,7-dimethoxyquinolin-4-yl)oxy)propyl)-1-methylpiperidin-4-amine (6c)

White solid, Yield 70%, m.p. 124–126** **°C; ^1^H NMR (400** **MHz, CDCl_3_) *δ* (*ppm*): 1.35–1.45 (m, 3H, piperidinyl 2C*H*H′, NH), 1.89 (d, 2H, piperidinyl 2CH*H*′, *J*** **=** **12.6** **Hz), 1.97 (t, 2H, piperidinyl 2C*H*H′, *J*** **=** **11.7** **Hz), 2.14 (p, 2H, CH_2_, *J*** **=** **6.5** **Hz), 2.25 (s, 3H, N-CH_3_), 2.43–2.51 (m, 1H, piperidinyl CH-NH), 2.80 (d, 2H, piperidinyl 2CH*H*′, *J*** **=** **11.7** **Hz), 2.92 (t, 2H, CH_2_-NH, *J*** **=** **6.9** **Hz), 4.01 (s, 3H, OCH_3_), 4.03 (s, 3H, OCH_3_), 4.35 (t, 2H, CH_2_-O, *J*** **=** **6.1** **Hz), 7.05 (s, 1H, vinyl CH), 7.37 (s, 1H, phenyl CH), 7.41 (s, 1H, phenyl CH), 7.44 (d, 2H, chlorophenyl 2CH, *J*** **=** **8.6** **Hz), 7.99 (d, 2H, chlorophenyl 2CH, *J*** **=** **8.6** **Hz); ^13^** **C NMR (100** **MHz, CDCl_3_) *δ* (*ppm*): 29.96, 32.91, 43.73, 46.23, 54.50, 54.64, 56.06, 56.12, 66.83, 97.39, 99.64, 108.25, 114.92, 128.54, 128.83, 134.91, 138.97, 146.19, 149.08, 152.72, 155.60, 161.05; HRESIMS (*m/z*): [M** **+** **H]^+^ Calcd for C_26_H_333_ClN_3_O_3_, 470.22050; found, 470.21988; HPLC purity: 96.18%.

##### 4.1.6.4. 3-((2–(4-chlorophenyl)-6,7-dimethoxyquinolin-4-yl)oxy)-N-((tetrahydrofuran-2-yl)methy- l)propan-1-amine (6d)

White solid, Yield 74%, m.p. 108–110** **°C; ^1^H NMR (400** **MHz, CDCl_3_) *δ* (*ppm*): 1.48–1.57 (m, 1H, furyl C*H*H′), 1.66 (s, 1H, NH), 1.83–1.92 (m, 2H, furyl CH_2_), 1.93–2 (m, 1H, furyl CH*H*′), 2.16 (p, 2H, CH_2_, *J*** **=** **6.6** **Hz), 2.67 (dd, 1H, furfuryl C*H*H′-NH, *J*** **=** **8, 12** **Hz) , 2.75 (dd, 1H, furfuryl CH*H*′-NH, *J*** **=** **3.6, 12** **Hz) , 2.92 (t, 2H, CH_2_-NH, *J*** **=** **6.9** **Hz), 3.70–3.75 (m, 1H, furyl C*H*H′-O), 3.80–3.85 (m, 1H, furyl CH*H*′-O), 4 (p, 1H, furyl CH-O, *J*** **=** **3.6** **Hz), 4.02 (s, 3H, OCH_3_), 4.03 (s, 3H, OCH_3_), 4.36 (t, 2H, CH_2_-O, *J*** **=** **6.2** **Hz), 7.06 (s, 1H, vinyl CH), 7.39 (s, 1H, phenyl CH), 7.41 (s, 1H, phenyl CH), 7.44 (d, 2H, chlorophenyl 2CH, *J*** **=** **8.6** **Hz), 8 (d, 2H, chlorophenyl 2CH, *J*** **=** **8.6** **Hz); ^13^** **C NMR (100** **MHz, CDCl_3_) *δ* (*ppm*): 25.78, 29.32, 29.67, 46.99, 54.64, 56.08, 66.80, 67.95, 78.31, 97.41, 99.80, 108.25, 114.97, 128.55, 128.80, 134.88, 139.02, 146.20, 149.09, 152.73, 155.58, 161.12; HRESIMS (*m/z*): [M** **+** **H]^+^ Calcd for C_25_H_30_ClN_2_O_4_, 457.18886; found, 457.18936; HPLC purity: 97.34%.

##### 4.1.6.5. 4-(3-(1H-imidazol-1-yl)propoxy)-2–(4-chlorophenyl)-6,7-dimethoxyquinoline (6e)

White solid, Yield 75%, m.p. 209–211** **°C; ^1^H NMR (400** **MHz, CDCl_3_) *δ* (*ppm*): 2.43 (p, 2H, CH_2_, *J*** **=** **6.2** **Hz), 4.03 (s, 3H, OCH_3_), 4.04 (s, 3H, OCH_3_), 4.22 (t, 2H, CH_2_-N, *J*** **=** **5.8** **Hz), 4.28 (t, 2H, CH_2_-O, *J*** **=** **6.6** **Hz), 6.94 (s, 1H, imidazole CH), 6.96 (s, 1H, vinyl CH), 7.07 (s, 1H, imidazole CH), 7.33 (s, 1H, phenyl CH), 7.43 (s, 1H, phenyl CH), 7.44 (d, 2H, chlorophenyl 2CH, *J*** **=** **8.5** **Hz), 7.50 (s, 1H, imidazole CH), 7.96 (d, 2H, chlorophenyl 2CH, *J*** **=** **8.5** **Hz); ^13^** **C NMR (100** **MHz, CDCl_3_) *δ* (*ppm*): 30.51, 43.59, 56.12, 64.48, 97.30, 99.33, 108.44, 114.67, 118.87, 128.51, 128.85, 130.03, 135.05, 137.23, 138.73, 146.34, 149.36, 152.94, 155.55, 160.43; HRESIMS (*m/z*): [M** **+** **H]^+^ Calcd for C_23_H_23_ClN_3_O_3_, 424.14225; found, 424.14264; HPLC purity: 99.50%.

##### 4.1.6.6. 6,7-dimethoxy-4–(3-(4-methylpiperazin-1-yl)propoxy)-2–(4-(trifluoromethyl)phenyl)quin-oline (6f)

White solid, Yield 71%, m.p. 84–86** **°C; ^1^H NMR (400** **MHz, CDCl_3_) *δ* (*ppm*): 2.17 (p, 2H, CH_2_, *J*** **=** **6.8** **Hz), 2.29 (s, 3H, CH_3_-N), 2.36–2.71 (br s, 8H, piperazinyl 4CH_2_), 2.63 (t, 2H, CH_2_-N, *J*** **=** **7.3** **Hz), 4.02 (s, 3H, OCH_3_), 4.03 (s, 3H, OCH_3_), 4.33 (t, 2H, CH_2_-O, *J*** **=** **6.3** **Hz), 7.09 (s, 1H, vinyl CH), 7.39 (s, 1H, phenyl CH), 7.43 (s, 1H, phenyl CH), 7.73 (d, 2H, CF_3_-phenyl 2CH, *J*** **=** **8.2** **Hz), 8.16 (d, 2H, CF_3_-phenyl 2CH, *J*** **=** **8.2** **Hz); ^13^** **C NMR (100** **MHz, CDCl_3_) *δ* (*ppm*): 26.59, 46.03, 53.34, 55.12, 56.07, 56.13, 66.72, 97.71, 99.64, 108.28, 115.18, 124.26 (CF_3_, q, *J*** **=** **271.7** **Hz), 125.59 (CH-C-CF_3_, q, *J*** **=** **3.9** **Hz), 127.59, 130.59 (CH-C-CF_3_, q, *J*** **=** **32.4** **Hz), 143.94, 146.24, 149.32, 152.84, 155.25, 161.18; ^19^** **F NMR (376.46** **MHz, CDCl_3_) *δ* (*ppm*): −62.47 (s); HRESIMS (*m/z*): [M** **+** **H]^+^ Calcd for C_26_H_31_F_3_N_3_O_3_, 490.23120; found, 490.23087; HPLC purity: 98.93%.

##### 4.1.6.7. N-(3-((6,7-dimethoxy-2–(4-(trifluoromethyl)phenyl)quinolin-4-yl)oxy)propyl)cyclohexan- amine (6g)

Grey solid, Yield 75%, m.p. 131–133** **°C; ^1^H NMR (400** **MHz, CDCl_3_) *δ* (*ppm*): 1.02–1.12 (m, 2H, cyclohexyl 2C*H*H′), 1.13–1.19 (m, 1H, cyclohexyl C*H*H′), 1.20–1.30 (m, 2H, cyclohexyl 2C*H*H′), 1.37 (br s, 1H, NH), 1.59–1.63 (m, 1H, cyclohexyl CH*H*′), 1.70–1.74 (m, 2H, cyclohexyl 2CH*H*′), 1.89–1.92 (m, 2H, cyclohexyl 2CH*H*′), 2.15 (p, 2H, CH_2_, *J*** **=** **6.5** **Hz), 2.43–2.50 (m, 1H, cyclohexyl CH-NH), 2.93 (t, 2H, CH_2_-NH, *J*** **=** **6.9** **Hz), 4.03 (s, 3H, OCH_3_), 4.04 (s, 3H, OCH_3_), 4.37 (t, 2H, CH_2_-O, *J*** **=** **6.1** **Hz), 7.11 (s, 1H, vinyl CH), 7.40 (s, 1H, phenyl CH), 7.43 (s, 1H, phenyl CH), 7.73 (d, 2H, CF_3_-phenyl 2CH, *J*** **=** **8.2** **Hz), 8.16 (d, 2H, CF_3_-phenyl 2CH, *J*** **=** **8.2** **Hz); ^13^** **C NMR (100** **MHz, CDCl_3_) *δ* (*ppm*): 25.05, 26.14, 30.03, 33.71, 43.87, 56.07, 56.13, 56.90, 67.01, 97.73, 99.64, 108.30, 115.19, 124.26 (CF_3_, q, *J*** **=** **272** **Hz), 125.59 (CH-C-CF_3_, q, *J*** **=** **3.7** **Hz), 127.57, 130.62 (CH-C-CF_3_, q, *J*** **=** **33.9** **Hz), 143.92, 146.24, 149.33, 152.84, 155.25, 161.17; ^19^** **F NMR (376.46** **MHz, CDCl_3_) *δ* (*ppm*): −62.47 (s); HRESIMS (*m/z*): [M** **+** **H]^+^ Calcd for C_27_H_32_F_3_N_2_O_3_, 489.23595; found489.23578; HPLC purity: 99.37%.

##### 4.1.6.8. N-(3-((6,7-dimethoxy-2–(4-(trifluoromethyl)phenyl)quinolin-4-yl)oxy)propyl)-1-methyl p- iperidin-4-amine (6h)

White solid, Yield 78%, m.p. 137–139** **°C; ^1^H NMR (400** **MHz, CDCl_3_) *δ* (*ppm*): 1.36–1.45 (m, 3H, piperidinyl 2C*H*H′, NH), 1.89 (d, 2H, piperidinyl 2CH*H*′, *J*** **=** **12.5** **Hz), 1.97 (t, 2H, piperidinyl 2C*H*H′, *J*** **=** **11.7** **Hz), 2.14 (p, 2H, CH_2_, *J*** **=** **6.5** **Hz), 2.25 (s, 3H, N-CH_3_), 2.43–2.51 (m, 1H, piperidinyl CH-NH), 2.80 (d, 2H, piperidinyl 2CH*H*′, *J*** **=** **11.7** **Hz), 2.92 (t, 2H, CH_2_-NH, *J*** **=** **6.9** **Hz), 4.02 (s, 3H, OCH_3_), 4.04 (s, 3H, OCH_3_), 4.37 (t, 2H, CH_2_-O, *J*** **=** **6.1** **Hz), 7.10 (s, 1H, vinyl CH), 7.39 (s, 1H, phenyl CH), 7.43 (s, 1H, phenyl CH), 7.73 (d, 2H, CF_3_-phenyl 2CH, *J*** **=** **8.2** **Hz), 8.16 (d, 2H, CF_3_-phenyl 2CH, *J*** **=** **8.2** **Hz); ^13^** **C NMR (100** **MHz, CDCl_3_) *δ* (*ppm*): 29.95, 32.89, 43.68, 46.21, 54.50, 54.63, 56.08, 56.14, 66.89, 97.71, 99.61, 108.31, 115.17, 124.27 (CF_3_, q, *J*** **=** **272.6** **Hz), 125.60 (CH-C-CF_3_, q, *J*** **=** **3.9** **Hz), 127.57, 130.64 (CH-C-CF_3_, q, *J*** **=** **32.7** **Hz), 143.90, 146.25, 149.34, 152.85, 155.26, 161.15; ^19^** **F NMR (376.46** **MHz, CDCl_3_) *δ* (*ppm*): −62.47 (s); HRESIMS (*m/z*): [M** **+** **H]^+^ Calcd for C_27_H_33_F_3_N_3_O_3_, 504.24685; found, 504.24692; HPLC purity: 99.75%.

##### 4.1.6.9. 3-((6,7-dimethoxy-2–(4-(trifluoromethyl)phenyl)quinolin-4-yl)oxy)-N-((tetrahydrofuran-2-yl)methyl)propan-1-amine (6i)

White solid, Yield 76%, m.p. 105–107** **°C; ^1^H NMR (400** **MHz, CDCl_3_) *δ* (*ppm*): 1.49–1.57 (m, 1H, furyl C*H*H′), 1.69 (s, 1H, NH), 1.83–1.92 (m, 2H, furyl CH_2_), 1.93–2 (m, 1H, furyl CH*H*′), 2.18 (p, 2H, CH_2_, *J*** **=** **6.6** **Hz), 2.68 (dd, 1H, furfuryl C*H*H′-NH, *J*** **=** **8, 12** **Hz) , 2.75 (dd, 1H, furfuryl CH*H*′-NH, *J*** **=** **3.5, 12** **Hz), 2.93 (t, 2H, CH_2_-NH, *J*** **=** **6.9** **Hz), 3.70–3.75 (m, 1H, furyl C*H*H′-O), 3.80–3.86 (m, 1H, furyl CH*H*′-O), 4 (p, 1H, furyl CH-O, *J*** **=** **3.5** **Hz), 4.03 (s, 3H, OCH_3_), 4.04 (s, 3H, OCH_3_), 4.38 (t, 2H, CH_2_-O, *J*** **=** **6.2** **Hz), 7.11 (s, 1H, vinyl CH), 7.41 (s, 1H, phenyl CH), 7.43 (s, 1H, phenyl CH), 7.43 (d, 2H, CF_3_-phenyl 2CH, *J*** **=** **8.2** **Hz), 8.16 (d, 2H, chlorophenyl 2CH, *J*** **=** **8.2** **Hz); ^13^** **C NMR (100** **MHz, CDCl_3_) *δ* (*ppm*): 25.78, 29.32, 29.64, 47.00, 54.67, 56.11, 56.13, 66.87, 67.97, 78.28, 97.74, 99.72, 108.26, 115.20, 124.28 (CF_3_, q, *J*** **=** **272** **Hz), 125.58 (CH-C-CF_3_, q, *J*** **=** **3.8** **Hz), 127.58, 130.59 (CH-C-CF_3_, q, *J*** **=** **32.3** **Hz), 143.92, 146.23, 149.31, 152.82, 155.24, 161.20; ^19^** **F NMR (376.46** **MHz, CDCl_3_) *δ* (*ppm*): −62.46 (s); HRESIMS (*m/z*): [M** **+** **H]^+^ Calcd for C_26_H_30_F_3_N_2_O_4_, 491.21522; found, 491.21533; HPLC purity: 99.63%.

##### 4.1.6.10. 4-(3-(1H-imidazol-1-yl)propoxy)-6,7-dimethoxy-2–(4-(trifluoromethyl)phenyl)quinoline (6j)

White solid, Yield 88%, m.p. 217–219** **°C; ^1^H NMR (400** **MHz, CDCl_3_) *δ* (*ppm*): 2.45 (p, 2H, CH_2_, *J*** **=** **6.2** **Hz), 4.05 (s, 3H, OCH_3_), 4.06 (s, 3H, OCH_3_), 4.24 (t, 2H, CH_2_-N, *J*** **=** **5.8** **Hz), 4.29 (t, 2H, CH_2_-O, *J*** **=** **6.6** **Hz), 6.94 (s, 1H, imidazole CH), 7.01 (s, 1H, vinyl CH), 7.08 (s, 1H, imidazole CH), 7.35 (s, 1H, phenyl CH), 7.46 (s, 1H, phenyl CH), 7.51 (s, 1H, imidazole CH), 7.73 (d, 2H, CF_3_-phenyl 2CH, *J*** **=** **8.2** **Hz), 8.13 (d, 2H, CF_3_-phenyl 2CH, *J*** **=** **8.2** **Hz); ^13^** **C NMR (100** **MHz, CDCl_3_) *δ* (*ppm*): 30.49, 43.58, 56.15, 56.18, 64.54, 97.63, 99.24, 108.47, 114.90, 118.88, 124.23 (CF_3_, q, *J*** **=** **272** **Hz), 125.63 (CH-C-CF_3_, q, *J*** **=** **3.7** **Hz), 127.56, 130.05, 130.72 (CH-C-CF_3_, q, *J*** **=** **32.5** **Hz), 137.25, 143.64, 146.38, 149.59, 153.03, 155.22, 160.51; ^19^** **F NMR (376.46** **MHz, CDCl_3_) *δ* (*ppm*): −62.48 (s); HRESIMS (*m/z*): [M** **+** **H]^+^ Calcd for C_24_H_23_F_3_N_3_O_3_, 458.16860; found, 458.16879; HPLC purity: 99.78%.

##### 4.1.6.11. 6,7-dimethoxy-4–(3-(4-methylpiperazin-1-yl)propoxy)-2-(p-tolyl)quinoline (6k)

White solid, Yield 70%, m.p. 128–130** **°C; ^1^H NMR (400** **MHz, CDCl_3_) *δ* (*ppm*): 2.15 (p, 2H, CH_2_, *J*** **=** **6.8** **Hz), 2.29 (s, 3H, CH_3_-N), 2.41 (s, 3H, tolyl CH_3_), 2.41–2.64 (br s, 8H, piperazinyl 4CH_2_), 2.62 (t, 2H, CH_2_-N, *J*** **=** **7.3** **Hz), 4.01 (s, 3H, OCH_3_), 4.02 (s, 3H, OCH_3_), 4.31 (t, 2H, CH_2_-O, *J*** **=** **6.3** **Hz), 7.07 (s, 1H, vinyl CH), 7.44 (d, 2H, tolyl 2CH, *J*** **=** **8** **Hz), 7.38 (s, 1H, phenyl CH), 7.43 (s, 1H, phenyl CH), 7.94 (d, 2H, tolyl 2CH, *J*** **=** **8** **Hz); ^13^C NMR (100** **MHz, CDCl_3_) *δ* (*ppm*): 21.29, 26.62, 46.03, 53.33, 55.12, 55.17, 56.02, 56.09, 66.56, 97.58, 99.72, 108.30, 114.76, 127.16, 129.39, 137.79, 138.77, 146.20, 148.77, 152.52, 157.01, 160.91; HRESIMS (*m/z*): [M** **+** **H]^+^ Calcd for C_26_H_34_N_3_O_3_, 436.25947; found, 436.25943; HPLC purity: 96.05%.

##### 4.1.6.12. N-(3-((6,7-dimethoxy-2-(p-tolyl)quinolin-4-yl)oxy)propyl)cyclohexanamine (6l)

Buff solid, Yield 73%, m.p. 126–128** **°C; ^1^H NMR (400** **MHz, CDCl_3_) *δ* (*ppm*): 1.03–1.14 (m, 2H, cyclohexyl 2C*H*H′), 1.15–1.21 (m, 1H, cyclohexyl C*H*H′), 1.23–1.30 (m, 2H, cyclohexyl 2C*H*H′), 1.59–1.63 (m, 2H, cyclohexyl CH*H*′, NH), 1.70–1.75 (m, 2H, cyclohexyl 2CH*H*′), 1.89–1.93 (m, 2H, cyclohexyl 2CH*H*′), 2.15 (p, 2H, CH_2_, *J*** **=** **6.6** **Hz), 2.41 (s, 3H, tolyl CH_3_), 2.44–2.51 (m, 1H, cyclohexyl CH-NH), 2.93 (t, 2H, CH_2_-NH, *J*** **=** **7** **Hz), 4.01 (s, 3H, OCH_3_), 4.03 (s, 3H, OCH_3_), 4.34 (t, 2H, CH_2_-O, *J*** **=** **6.1** **Hz), 7.08 (s, 1H, vinyl CH), 7.29 (d, 2H, tolyl 2CH, *J*** **=** **8** **Hz), 7.39 (s, 1H, phenyl CH), 7.43 (s, 1H, phenyl CH), 7.95 (d, 2H, tolyl 2CH, *J*** **=** **8** **Hz); ^13^** **C NMR (100** **MHz, CDCl_3_) *δ* (*ppm*): 21.29, 25.05, 26.13, 29.96, 33.62, 43.93, 56.03, 56.09, 56.91, 66.84, 97.60, 99.71, 108.32, 114.77, 127.15, 129.40, 137.77, 138.78, 146.21, 148.79, 152.52, 157.01, 160.88; HRESIMS (*m/z*): [M** **+** **H]^+^ Calcd for C_27_H_35_N_2_O_3_, 435.26422; found, 435.26422; HPLC purity: 97.90%.

##### 4.1.6.13. N-(3-((6,7-dimethoxy-2-(p-tolyl)quinolin-4-yl)oxy)propyl)-1-methylpiperidin-4-amine (6m)

White solid, Yield 76%, m.p. 66–68** **°C; ^1^H NMR (400** **MHz, CDCl_3_) *δ* (*ppm*): 1.35–1.45 (m, 3H, piperidinyl 2C*H*H′, NH), 1.89 (d, 2H, piperidinyl 2CH*H*′, *J*** **=** **12.5** **Hz), 1.96 (t, 2H, piperidinyl 2C*H*H′, *J*** **=** **11.8** **Hz), 2.13 (p, 2H, CH_2_, *J*** **=** **6.5** **Hz), 2.24 (s, 3H, N-CH_3_), 2.41 (s, 3H, tolyl CH_3_), 2.43–2.50 (m, 1H, piperidinyl CH-NH), 2.80 (d, 2H, piperidinyl 2CH*H*′, *J*** **=** **11.8** **Hz), 2.91 (t, 2H, CH_2_-NH, *J*** **=** **6.9** **Hz), 4.01 (s, 3H, OCH_3_), 4.02 (s, 3H, OCH_3_), 4.34 (t, 2H, CH_2_-O, *J*** **=** **6.1** **Hz), 7.08 (s, 1H, vinyl CH), 7.28 (d, 2H, tolyl 2CH, *J*** **=** **8** **Hz), 7.37 (s, 1H, phenyl CH), 7.43 (s, 1H, phenyl CH), 7.94 (d, 2H, tolyl 2CH, *J*** **=** **8** **Hz); ^13^** **C NMR (100** **MHz, CDCl_3_) *δ* (*ppm*): 21.29, 29.97, 32.90, 43.79, 46.22, 54.49, 54.64, 56.03, 56.09, 66.74, 97.58, 99.68, 108.33, 114.75, 127.14, 129.40, 137.76, 138.79, 146.21, 148.79, 152.53, 157.00, 160.87; HRESIMS (*m/z*): [M** **+** **H]^+^ Calcd for C_27_H_36_N_3_O_3_, 450.27512; found, 450.27481; HPLC purity: 95.52%.

##### 4.1.6.14. 3-((6,7-dimethoxy-2-(p-tolyl)quinolin-4-yl)oxy)-N-((tetrahydrofuran-2-yl)methyl)propa- n-1-amine (6n)

Yellow solid, Yield 85%, m.p. 60–62** **°C; ^1^H NMR (400** **MHz, CDCl_3_) *δ* (*ppm*): 1.48–1.57 (m, 1H, furyl C*H*H′), 1.83–2.00 (m, 4H, furyl CH_2_, furyl CH*H*′, NH), 2.16 (p, 2H, CH_2_, *J*** **=** **6.5** **Hz), 2.41 (s, 3H, tolyl CH_3_), 2.67 (dd, 1H, furfuryl C*H*H′-NH, *J*** **=** **8, 12** **Hz), 2.74 (dd, 1H, furfuryl CH*H*′-NH, *J*** **=** **3.6, 12** **Hz), 2.92 (t, 2H, CH_2_-NH, *J*** **=** **6.8** **Hz), 3.69–3.75 (m, 1H, furyl C*H*H′-O), 3.79–3.85 (m, 1H, furyl CH*H*′-O), 4 (p, 1H, furyl CH-O, *J*** **=** **3.6** **Hz), 4.02 (s, 3H, OCH_3_), 4.03 (s, 3H, OCH_3_), 4.35 (t, 2H, CH_2_-O, *J*** **=** **6.2** **Hz), 7.08 (s, 1H, vinyl CH), 7.29 (d, 2H, tolyl 2CH, *J*** **=** **8** **Hz), 7.39 (s, 1H, phenyl CH), 7.43 (s, 1H, phenyl CH), 7.94 (d, 2H, tolyl 2CH, *J*** **=** **8** **Hz); ^13^** **C NMR (100** **MHz, CDCl_3_) *δ* (*ppm*): 21.28, 25.77, 29.33, 29.61, 47.06, 54.61, 56.06, 56.08, 66.70, 67.95, 78.23, 97.64, 99.80, 108.22, 114.78, 127.18, 129.39, 137.76, 138.78, 146.16, 148.78, 152.53, 157.03, 160.93; HRESIMS (*m/z*): [M** **+** **H]^+^ Calcd for C_26_H_33_N_2_O_4_, 437.24348; found, 437.24353; HPLC purity: 97.50%.

##### 4.1.6.15. 4-(3-(1H-imidazol-1-yl)propoxy)-6,7-dimethoxy-2-(p-tolyl)quinoline (6o)

White solid, Yield 83%, m.p. 203–205** **°C; ^1^H NMR (400** **MHz, CDCl_3_) *δ* (*ppm*): 2.41 (s, 3H, tolyl CH_3_), 2.42 (p, 2H, CH_2_, *J*** **=** **6.3** **Hz), 4.03 (s, 6H, 2CH_3_O), 4.22 (t, 2H, CH_2_-N, *J*** **=** **5.8** **Hz), 4.27 (t, 2H, CH_2_-O, *J*** **=** **6.7** **Hz), 6.93 (s, 1H, imidazole CH), 6.99 (s, 1H, vinyl CH), 7.07 (s, 1H, imidazole CH), 7.28 (d, 2H, tolyl 2CH, *J*** **=** **8.1** **Hz), 7.34 (s, 1H, phenyl CH), 7.45 (s, 1H, phenyl CH), 7.51 (s, 1H, imidazole CH), 7.92 (d, 2H, tolyl 2CH, *J*** **=** **8.1** **Hz); ^13^** **C NMR (100** **MHz, CDCl_3_) *δ* (*ppm*): 21.29, 30.55, 43.62, 56.10, 56.13, 64.37, 97.50, 99.35, 108.48, 114.48, 118.92, 127.12, 129.44, 129.97, 137.22, 137.51, 138.96, 146.34, 149.03, 152.71, 156.97, 160.25; HRESIMS (*m/z*): [M** **+** **H]^+^ Calcd for C_24_H_26_N_3_O_3_, 404.19687; found, 404.19724; HPLC purity: 99.42%.

#### 4.1.7. Synthesis of 2-(4-bromophenyl)-6,7-dimethoxy-4-(3-(4-methylpiperazin-1-yl)propoxy)qu-inoline (7)

The bromo analog of 4-propoxy-*N*-methylpiperazine-2-arylquinoline **7** has been prepared using the same synthetic procedure as **6a–o** utilising *p*-bromobenzoyl chloride.

White solid, Yield 77%, m.p. 148–150** **°C; ^1^H NMR (400** **MHz, CDCl_3_) *δ* (*ppm*): 2.15 (p, 2H, CH_2_, *J*** **=** **6.8** **Hz), 2.29 (s, 3H, CH_3_-N), 2.36–2.66 (br s, 8H, piperazinyl 4CH_2_), 2.62 (t, 2H, CH_2_-N, *J*** **=** **7.3** **Hz), 4.01 (s, 3H, OCH_3_), 4.02 (s, 3H, OCH_3_), 4.31 (t, 2H, CH_2_-O, *J*** **=** **6.3** **Hz), 7.03 (s, 1H, vinyl CH), 7.37 (s, 1H, phenyl CH), 7.41 (s, 1H, phenyl CH), 7.60 (d, 2H, bromophenyl 2CH, *J*** **=** **8.6** **Hz), 7.92 (d, 2H, bromophenyl 2CH, *J*** **=** **8.6** **Hz); ^13^** **C NMR (100** **MHz, CDCl_3_) *δ* (*ppm*): 26.57, 45.96, 53.24, 55.07, 56.04, 56.10, 66.65, 97.34, 99.71, 108.23, 114.97, 123.23, 128.85, 131.77, 139.45, 146.20, 149.11, 152.76, 155.62, 161.10; HRESIMS (*m/z*): [M** **+** **H]^+^ Calcd for C_25_H_31_BrN_3_O_3_, 500.15433; found, 500.15466.

#### 4.1.8. General procedure for synthesis of heterocyclic analogs of the bromo derivative (8a,b)

The bromo derivative **7** (125** **mg, 0.25** **mmol) was taken with 2-furylboronic acid or 2-thienylboronic acid (0.5** **mmol) and Pd(PPh_3_)_4_ (0.05 equivalent, 15** **mg), then dioxane (5** **ml) and 2** **M Na_2_CO_3_ (0.3** **ml) were added to the mixture under N_2_ atmosphere. The reaction mixture was refluxed under N_2_ at 90** **°C for 16** **h. Then, the reaction mixture was poured into ice/water (50** **ml), the aqueous layer was extracted with ethyl acetate (50** **×** **3) and the organic layers were washed with water and brine. After evaporation of the organic solvent under vacuum, the residue was purified by silica gel column chromatography using DCM/MeOH to furnish **8a,b** in pure form.

##### 4.1.8.1. 2-(4-(furan-2-yl)phenyl)-6,7-dimethoxy-4-(3-(4-methylpiperazin-1-yl)propoxy)quinoline (8a)

Yellow solid, Yield 70%, m.p. 135–137** **°C; ^1^H NMR (400** **MHz, CDCl_3_) *δ* (*ppm*): 2.17 (p, 2H, CH_2_, *J*** **=** **6.8** **Hz), 2.34 (s, 3H, CH_3_-N), 2.43–2.78 (br s, 8H, piperazinyl 4CH_2_), 2.66 (t, 2H, CH_2_-N, *J*** **=** **7.3** **Hz), 4.02 (s, 3H, OCH_3_), 4.04 (s, 3H, OCH_3_), 4.33 (t, 2H, CH_2_-O, *J*** **=** **6.3** **Hz), 6.50 (dd, 1H, furyl CH, *J*** **=** **1.8, 3.4** **Hz), 6.73 (dd, 1H, furyl CH, *J*** **=** **0.5, 3.4** **Hz), 7.11 (s, 1H, vinyl CH), 7.38 (s, 1H, phenyl CH), 7.44 (s, 1H, phenyl CH), 7.50 (dd, 1H, furyl CH-O, *J*** **=** **0.5, 1.8** **Hz), 7.79 (d, 2H, 2-phenyl 2CH, *J*** **=** **8.5** **Hz), 8.09 (d, 2H, 2-phenyl 2CH, *J*** **=** **8.5** **Hz); ^13^** **C NMR (100** **MHz, CDCl_3_) *δ* (*ppm*): 26.53, 45.73, 52.86, 54.91, 55.02, 56.07, 56.12, 66.51, 97.49, 99.69, 105.63, 108.28, 111.83, 114.89, 124.02, 127.58, 131.21, 139.24, 142.35, 146.23, 148.95, 152.65, 153.71, 156.27, 160.95; HRESIMS (*m/z*): [M** **+** **H]^+^ Calcd for C_29_H_34_N_3_O_4_, 488.25438; found, 488.25446; HPLC purity: 96.06%.

##### 4.1.8.2. 6,7-dimethoxy-4–(3-(4-methylpiperazin-1-yl)propoxy)-2–(4-(thiophen-2-yl)phenyl)quinol-ine (8b)

Buff solid, Yield 72%, m.p. 155–157** **°C; ^1^H NMR (400** **MHz, CDCl_3_) *δ* (*ppm*): 2.17 (p, 2H, CH_2_, *J*** **=** **6.8** **Hz), 2.29 (s, 3H, CH_3_-N), 2.34–2.70 (br s, 8H, piperazinyl 4CH_2_), 2.63 (t, 2H, CH_2_-N, *J*** **=** **7.3** **Hz), 4.02 (s, 3H, OCH_3_), 4.03 (s, 3H, OCH_3_), 4.33 (t, 2H, CH_2_-O, *J*** **=** **6.3** **Hz), 7.09 (dd, 1H, thienyl CH, *J*** **=** **3.7, 5.1** **Hz), 7.10 (s, 1H, vinyl CH), 7.30 (dd, 1H, thienyl CH, *J*** **=** **1.1, 5.1** **Hz), 7.38 (dd, 1H, thienyl CH-S, *J*** **=** **1.1, 3.7** **Hz), 7.39 (s, 1H, phenyl CH), 7.44 (s, 1H, phenyl CH), 7.73 (d, 2H, 2-phenyl 2CH, *J*** **=** **8.4** **Hz), 8.08 (d, 2H, 2-phenyl 2CH, *J*** **=** **8.4** **Hz); ^13^** **C NMR (100** **MHz, CDCl_3_) *δ* (*ppm*): 26.62, 46.05, 53.35, 55.13, 55.17, 56.04, 56.12, 66.62, 97.49, 99.72, 108.30, 114.94, 123.39, 125.12, 126.09, 127.76, 128.13, 134.80, 139.52, 144.01, 146.25, 148.95, 152.64, 156.20, 160.99; HRESIMS (*m/z*): [M** **+** **H]^+^ Calcd for C_29_H_34_N_3_O_3_S, 504.23154; found, 504.23141; HPLC purity: 98.96%.

#### 4.1.9. Synthesis of 4-(3-chloropropoxy)-2-(4-(trifluoromethyl)phenyl)quinoline (9)

The demethoxylated key intermediate **9** has been synthesised using the same synthetic procedure for **5a–c** starting from 2-aminoacetophenone.

White solid, Yield 76%, m.p. 98–100** **°C; ^1^H NMR (400** **MHz, CDCl_3_) *δ* (*ppm*): 2.45 (p, 2H, CH_2_, *J*** **=** **6** **Hz), 2.87 (t, 2H, CH_2_-N, *J*** **=** **6.2** **Hz), 4.46 (t, 2H, CH_2_-O, *J*** **=** **5.8** **Hz), 7.20 (s, 1H, vinyl CH), 7.52 (ddd, 1H, phenyl CH, *J*** **=** **1.2, 6.9, 8.3** **Hz), 7.74 (ddd, 1H, phenyl CH, *J*** **=** **1.3, 6.9, 8.4** **Hz), 7.77 (d, 2H, CF_3_-phenyl 2CH, *J*** **=** **8.2** **Hz), 8.11 (d, 2H, phenyl CH, *J*** **=** **8.4** **Hz), 8.19 (dd, 1H, phenyl CH, *J*** **=** **1.3, 8.3** **Hz), 8.22 (d, 2H, CF_3_-phenyl 2CH, *J*** **=** **8.2** **Hz); ^13^** **C NMR (100** **MHz, CDCl_3_) *δ* (*ppm*): 31.94, 41.23, 64.86, 98.50, 121.56, 124.20 (CF_3_, q, *J*** **=** **272** **Hz), 125.67 (CH-C-CF_3_, q, *J*** **=** **3.7** **Hz), 125.96, 127.87, 129.42, 130.31, 131.08 (CH-C-CF_3_, q, *J*** **=** **32.4** **Hz), 143.56, 149.23, 157.12, 162.07; ^19^** **F NMR (376.46** **MHz, CDCl_3_) *δ* (*ppm*): −62.53 (s); HRESIMS (*m/z*): [M** **+** **H]^+^ Calcd for C_19_H_16_ClF_3_NO, 366.08670; found, 366.08725.

#### 4.1.10. Synthesis of the target demethoxylated 4-propoxy-2-arylquinolines (10a,b)

The demethoxylated 4-propoxy-2-arylquinolines **10a,b** were prepared according to the general synthetic procedure for **6a–o** using the respective key intermediate **9**.

##### 4.1.10.1. 4-(3-(4-methylpiperazin-1-yl)propoxy)-2-(4-(trifluoromethyl)phenyl)quinoline (10a)

White solid, Yield 87%, m.p. 110–112** **°C; ^1^H NMR (400** **MHz, CDCl_3_) *δ* (*ppm*): 2.16 (p, 2H, CH_2_, *J*** **=** **6.7** **Hz), 2.29 (s, 3H, CH_3_-N), 2.37–2.71 (br s, 8H, piperazinyl 4CH_2_), 2.64 (t, 2H, CH_2_-N, *J*** **=** **7.2** **Hz), 4.34 (t, 2H, CH_2_-O, *J*** **=** **6.2** **Hz), 7.16 (s, 1H, vinyl CH), 7.50 (ddd, 1H, phenyl CH, *J*** **=** **1.1, 6.9, 8.2** **Hz), 7.72 (ddd, 1H, phenyl CH, *J*** **=** **1.5 6.9, 8.3** **Hz), 7.75 (d, 2H, CF_3_-phenyl 2CH, *J*** **=** **8.2** **Hz), 8.09 (d, 2H, phenyl CH, *J*** **=** **8.3** **Hz), 8.2 (m, 3H, phenyl CH, CF_3_-phenyl 2CH); ^13^** **C NMR (100** **MHz, CDCl_3_) *δ* (*ppm*): 26.54, 46.04, 53.30, 55.00, 55.13, 66.79, 98.49, 120.63, 121.74, 124.21 (CF_3_, q, *J*** **=** **272** **Hz), 125.63 (CH-C-CF_3_, q, *J*** **=** **3.8** **Hz), 125.82 127.89, 129.32, 130.21, 130.99 (CH-C-CF_3_, q, *J*** **=** **32.6** **Hz), 143.72, 149.19, 157.14, 162.43; ^19^** **F NMR (376.46** **MHz, CDCl_3_) *δ* (*ppm*): −62.53 (s); HRESIMS (*m/z*): [M + Na]^+^ Calcd for C_21_H_33_ClF_3_NONa, 430.20950; found, 430.20993; HPLC purity: 98.63%.

##### 4.1.10.2. N-(3-((2-(4-(trifluoromethyl)phenyl)quinolin-4-yl)oxy)propyl)cyclohexanamine (10b)

Grey solid, Yield 78%, m.p. 68–70** **°C; ^1^H NMR (400** **MHz, CDCl_3_) *δ* (*ppm*): 1.03–1.13 (m, 2H, cyclohexyl 2C*H*H′), 1.14–1.19 (m, 1H, cyclohexyl C*H*H′), 1.20–1.31 (m, 3H, cyclohexyl 2C*H*H′, NH), 1.60–1.63 (m, 1H, cyclohexyl CH*H*′), 1.71–1.75 (m, 2H, cyclohexyl 2CH*H*′), 1.91 (d, 2H, cyclohexyl 2CH*H*′, *J*** **=** **10.2** **Hz), 2.15 (p, 2H, CH_2_, *J*** **=** **6.5** **Hz), 2.43–2.50 (m, 1H, cyclohexyl CH-NH), 2.94 (t, 2H, CH_2_-NH, *J*** **=** **6.9** **Hz), 4.38 (t, 2H, CH_2_-O, *J*** **=** **6.1** **Hz), 7.18 (s, 1H, vinyl CH), 7.51 (ddd, 1H, phenyl CH, *J*** **=** **1.1, 7, 8.1** **Hz), 7.72 (ddd, 1H, phenyl CH, *J*** **=** **1.4 7, 8.3** **Hz), 7.75 (d, 2H, CF_3_-phenyl 2CH, *J*** **=** **8.7** **Hz), 8.09 (d, 2H, phenyl CH, *J*** **=** **8.3** **Hz), 8.21 (d, 3H, phenyl CH, CF_3_-phenyl 2CH, *J*** **=** **8.1** **Hz); ^13^** **C NMR (100** **MHz, CDCl_3_) *δ* (*ppm*): 25.08, 26.16, 30.05, 33.71, 43.77, 56.94, 67.04, 98.51, 120.64, 121.74, 124.25 (CF_3_, q, *J*** **=** **271.7** **Hz), 125.64 (CH-C-CF_3_, q, *J*** **=** **3.7** **Hz), 125.82, 127.88, 129.33, 130.20, 130.99 (CH-C-CF_3_, q, *J*** **=** **32.4** **Hz), 143.70, 149.20, 157.15, 162.42; ^19^** **F NMR (376.46** **MHz, CDCl_3_) *δ* (*ppm*): −62.53 (s); HRESIMS (*m/z*): [M** **+** **H]^+^ Calcd for C_25_H_28_F_3_N_2_O, 429.21482; found, 429.21420; HPLC purity: 99.22%.

#### 4.1.11. Synthesis of 1,3-dioxoloarylquinolines key intermediates (11a,b)

Starting from 6′-amino-3′,4′-(methylenedioxy)acetophenone and the respective *p*-substituted benzoyl chloride, the key intermediates **11a,b** were prepared according to the synthetic route for **5a–c**.

##### 4.1.11.1. 6-(4-chlorophenyl)-8–(3-chloropropoxy)-[1,3]dioxolo[4,5-g]quinoline (11a)

White solid, Yield 90%, m.p. 186–188** **°C; ^1^H NMR (400** **MHz, CDCl_3_) *δ* (*ppm*): 2.40 (p, 2H, CH_2_, *J*** **=** **6** **Hz), 3.83 (t, 2H, CH_2_Cl, *J*** **=** **6.2** **Hz), 4.39 (t, 2H, CH_2_-O, *J*** **=** **5.8** **Hz), 6.09 (s, 2H, dioxolo CH_2_), 7.05 (s, 1H, vinyl CH), 7.37 (s, 1H, phenyl CH), 7.39 (s, 1H, phenyl CH), 7.45 (d, 2H, chlorophenyl 2CH, *J*** **=** **8.6** **Hz), 8 (d, 2H, chlorophenyl 2CH, *J*** **=** **8.6** **Hz); ^13^** **C NMR (100** **MHz, CDCl_3_) *δ* (*ppm*): 31.99, 41.27, 64.68, 97.50, 101.68, 106.01, 116.23, 128.53, 128.84, 135.06, 138.63, 147.30, 147.48, 151.04, 155.48, 161.15; HRESIMS (*m/z*): [M** **+** **H]^+^ Calcd for C_19_H_16_Cl_2_NO_3_, 376.05018; found, 376.05045.

##### 4.1.11.2. 8-(3-chloropropoxy)-6-(4-(trifluoromethyl)phenyl)-[1,3] dioxolo[4,5-g]quinoline (11b)

White solid, Yield 93%, m.p. 145–147** **°C; ^1^H NMR (400** **MHz, CDCl_3_) *δ* (*ppm*): 2.41 (p, 2H, CH_2_, *J*** **=** **6** **Hz), 3.84 (t, 2H, CH_2_Cl, *J*** **=** **6.2** **Hz), 4.41 (t, 2H, CH_2_-O, *J*** **=** **5.8** **Hz), 6.10 (s, 2H, dioxolo CH_2_), 7.10 (s, 1H, vinyl CH), 7.39 (s, 1H, phenyl CH), 7.40 (s, 1H, phenyl CH), 7.73 (d, 2H, CF_3_-phenyl 2CH, *J*** **=** **8.1** **Hz), 8.16 (d, 2H, CF_3_-phenyl 2CH, *J*** **=** **8.1** **Hz); ^13^** **C NMR (100** **MHz, CDCl_3_) *δ* (*ppm*): 31.96, 41.24, 64.74, 97.50, 97.83, 101.75, 106.09, 116.50, 124.25 (CF_3_, q, *J*** **=** **271.8** **Hz), 125.61 (CH-C-CF_3_, q, *J*** **=** **3.7** **Hz), 127.56, 130.72 (CH-C-CF_3_, q, *J*** **=** **32.4** **Hz), 143.55, 147.54, 147.57, 151.16, 155.13, 161.25; ^19^** **F NMR (376.46** **MHz, CDCl_3_) *δ* (*ppm*): −62.50 (s); HRESIMS (*m/z*): [M** **+** **H]^+^ Calcd for C_20_H_16_ClF_3_NO_3_, 410.07653; found, 410.07736.

#### 4.1.12. Synthesis of the target propoxy derivatives of 1,3-dioxoloarylquinolines (12a–d)

The target dioxolo derivatives **12a–d** have been synthesised utilising the synthetic procedures used for **6a–o** using imidazole or morpholine with the appropriate key intermediate **11a,b**.

##### 4.1.12.1. 8-(3-(1H-imidazol-1-yl)propoxy)-6-(4-chlorophenyl)-[1,3] dioxolo[4,5-g]quinoline (12a)

White solid, Yield 77%, m.p. 146–148** **°C; ^1^H NMR (400** **MHz, CDCl_3_) *δ* (*ppm*): 2.39 (p, 2H, CH_2_, *J*** **=** **6.2** **Hz), 4.16 (t, 2H, CH_2_-N, *J*** **=** **5.7** **Hz), 4.28 (t, 2H, CH_2_-O, *J*** **=** **6.7** **Hz), 6.10 (s, 2H, dioxolo CH_2_), 6.93 (s, 1H, imidazole CH), 6.94 (s, 1H, vinyl CH), 7.07 (s, 1H, imidazole CH), 7.38 (s, 1H, phenyl CH), 7.39 (s, 1H, phenyl CH), 7.43 (d, 2H, chlorophenyl 2CH, *J*** **=** **8.7** **Hz), 7.49 (s, 1H, imidazole CH), 7.95 (d, 2H, chlorophenyl 2CH, *J*** **=** **8.7** **Hz); ^13^** **C NMR (100** **MHz, CDCl_3_) *δ* (*ppm*): 30.54, 43.43, 64.18, 97.23, 97.46, 101.76, 106.13, 116.07, 118.90, 128.51, 128.85, 129.98, 135.12, 137.27, 138.49, 147.46, 147.53, 151.13, 155.48, 160.83; HRESIMS (*m/z*): [M** **+** **H]^+^ Calcd for C_22_H_19_ClN_3_O_3_, 408.11095; found, 408.11179; HPLC purity: 99.60%.

##### 4.1.12.2. 6-(4-chlorophenyl)-8-(3-morpholinopropoxy)-[1,3]dioxolo [4,5-g]quinoline (12b)

White solid, Yield 72%, m.p. 153–155** **°C; ^1^H NMR (400** **MHz, CDCl_3_) *δ* (*ppm*): 2.12 (p, 2H, CH_2_, *J*** **=** **6.7** **Hz), 2.49 (t, 4H, morpholinyl 2CH_2_, *J*** **=** **4.6** **Hz), 2.61 (t, 2H, CH_2_-N, *J*** **=** **7.2** **Hz), 3.73 (t, 4H, morpholinyl 2CH_2_, *J*** **=** **4.6** **Hz), 4.29 (t, 2H, CH_2_-O, *J*** **=** **6.2** **Hz), 6.08 (s, 2H, dioxolo CH_2_), 7.03 (s, 1H, vinyl CH), 7.36 (s, 1H, phenyl CH), 7.42 (s, 1H, phenyl CH), 7.44 (d, 2H, chlorophenyl 2CH, *J*** **=** **8.7** **Hz), 7.99 (d, 2H, chlorophenyl 2CH, *J*** **=** **8.7** **Hz); ^13^** **C NMR (100** **MHz, CDCl_3_) *δ* (*ppm*): 26.27, 53.82, 55.47, 66.43, 66.98, 97.50, 97.66, 101.63, 105.97, 116.35, 128.53, 128.82, 135.00, 138.76, 147.21, 147.44, 150.97, 155.51, 161.49; HRESIMS (*m/z*): [M** **+** **H]^+^ Calcd for C_23_H_24_ClN_2_O_4_, 427.14191; found, 427.14221; HPLC purity: 98.94%.

##### 4.1.12.3. 8-(3-(1H-imidazol-1-yl)propoxy)-6-(4-(trifluoromethyl)phenyl)-[1,3]dioxolo[4,5-g]qui-noline (12c)

White solid, Yield 81%, m.p. 125–127** **°C; ^1^H NMR (400** **MHz, CDCl_3_) *δ* (*ppm*): 2.41 (p, 2H, CH_2_, *J*** **=** **6.2** **Hz), 4.19 (t, 2H, CH_2_-N, *J*** **=** **5.7** **Hz), 4.29 (t, 2H, CH_2_-O, *J*** **=** **6.7** **Hz), 6.12 (s, 2H, dioxolo CH_2_), 6.93 (s, 1H, imidazole CH), 7 (s, 1H, vinyl CH), 7.08 (s, 1H, imidazole CH), 7.41 (s, 2H, phenyl 2CH), 7.49 (s, 1H, imidazole CH), 7.72 (d, 2H, CF_3_-phenyl 2CH, *J*** **=** **8.2** **Hz), 8.12 (d, 2H, CF_3_-phenyl 2CH, *J*** **=** **8.2** **Hz); ^13^** **C NMR (100** **MHz, CDCl_3_) *δ* (*ppm*): 30.52, 43.42, 64.26, 97.22, 97.79, 101.83, 106.22, 16.34, 118.88, 124.22 (CF_3_, q, *J*** **=** **272.7** **Hz), 125.61 (CH-C-CF_3_, q, *J*** **=** **3.7** **Hz), 127.55, 130.01, 130.79 (CH-C-CF_3_, q, *J*** **=** **33.2** **Hz), 137.28, 143.41, 147.60, 147.73, 151.26, 155.15, 160.92; ^19^** **F NMR (376.46** **MHz, CDCl_3_) *δ* (*ppm*): −62.52 (s); HRESIMS (*m/z*): [M** **+** **H]^+^ Calcd for C_23_H_19_F_3_N_3_O_3_, 442.13730; found, 442.13754; HPLC purity: 99.35%.

##### 4.1.12.4. 8-(3-morpholinopropoxy)-6-(4-(trifluoromethyl)phenyl)-[1,3]dioxolo[4,5-g]quinoline (12d)

White solid, Yield 76%, m.p. 138–140** **°C; ^1^H NMR (400** **MHz, CDCl_3_) *δ* (*ppm*): 2.14 (p, 2H, CH_2_, *J*** **=** **6.7** **Hz), 2.50 (t, 4H, morpholinyl 2CH_2_, *J*** **=** **4.4** **Hz), 2.62 (t, 2H, CH_2_-N, *J*** **=** **7.2** **Hz), 3.73 (t, 4H, morpholinyl 2CH_2_, *J*** **=** **4.4** **Hz), 4.32 (t, 2H, CH_2_-O, *J*** **=** **6.2** **Hz), 6.10 (s, 2H, dioxolo CH_2_), 7.08 (s, 1H, vinyl CH), 7.39 (s, 1H, phenyl CH), 7.44 (s, 1H, phenyl CH), 7.73 (d, 2H, CF_3_-phenyl 2CH, *J*** **=** **8.2** **Hz), 8.16 (d, 2H, CF_3_-phenyl 2CH, *J*** **=** **8.2** **Hz); ^13^** **C NMR (100** **MHz, CDCl_3_) *δ* (*ppm*): 26.27, 53.82, 55.45, 66.51, 66.98, 97.65, 97.83, 101.70, 106.05, 116.62, 124.23 (CF_3_, q, *J*** **=** **272.3** **Hz), 125.59 (CH-C-CF_3_, q, *J*** **=** **3.9** **Hz), 127.57, 130.70 (CH-C-CF_3_, q, *J*** **=** **32.2** **Hz), 143.70, 147.48, 147.51, 151.10, 155.18, 161.59; ^19^** **F NMR (376.46** **MHz, CDCl_3_) *δ* (*ppm*): −62.50 (s); HRESIMS (*m/z*): [M** **+** **H]^+^ Calcd for C_24_H_24_F_3_N_2_O_4_, 461.16827; found, 461.16837; HPLC purity: 99.62%.

#### 4.1.13. Synthesis of 1-(2-amino-5-bromophenyl)ethan-1-one (13)

The 5-bromo derivative of 2-aminoacetophenone **13** has been synthesised based on the reported procedure^46^^,^^47^.

Yellow solid, Yield 96%, m.p. 77–79** **°C; ^1^H NMR (400** **MHz, CDCl_3_) *δ* (*ppm*): 2.55 (s, 3H, CH_3_), 6.29 (s, 2H, NH_2_), 6.54 (d, 1H, phenyl CH, *J*** **=** **8.8** **Hz), 7.31 (dd, 1H, phenyl CH, *J*** **=** **2.3, 8.8** **Hz), 7.78 (d, 1H, phenyl CH, *J*** **=** **2.3** **Hz); ^13^** **C NMR (100** **MHz, CDCl_3_) *δ* (*ppm*): 27.83, 106.63, 118.99, 119.40, 134.11, 136.99, 149.08, 199.64; HRESIMS (*m/z*): [M** **+** **H]^+^ Calcd for C_8_H_9_CBrNO, 213.98620; found, 213.98599.

#### 4.1.14. Synthesis of 6-bromo-2-arylquinolones (14a,b)

1-(2-amino-5-bromophenyl)ethan-1-one **13** was benzoylated with *p*-chloro or fluorobenzoyl chloride using the same procedure for **3a–c**. Then, the resulted benzoyl derivatives have been subjected to ring closure reaction according to the synthetic route for **4a–c** to afford the corresponding 6-bromo-2-arylquinolones **14a,b**.

##### 4.1.14.1. 6-bromo-2-(4-chlorophenyl)quinolin-4(1H)-one (14a)

Yellow solid, Yield 95%, m.p. > 250** **°C; ^1^H NMR (400** **MHz, CDCl_3_) *δ* (*ppm*): 6.41 (s, 1H, vinyl CH), 7.67 (d, 2H, chlorophenyl 2CH, *J*** **=** **8.5** **Hz), 7.72 (d, 1H, bromophenyl CH, *J*** **=** **8.9** **Hz), 7.84 (dd, 1H, bromophenyl CH, *J*** **=** **1.7, 8.9** **Hz), 7.87 (d, 2H, chlorophenyl 2CH, *J*** **=** **8.5** **Hz), 8.17 (d, 1H, bromophenyl CH, *J*** **=** **1.7** **Hz), 11.91 (s, 1H, NH); ^13^** **C NMR (100** **MHz, CDCl_3_) *δ* (*ppm*): 108.25, 116.52, 121.86, 126.76, 127.35, 129.53, 129.81, 133.14, 135.15, 135.96, 139.85, 149.64, 176.06; HRESIMS (*m/z*): [M** **+** **H]^+^ Calcd for C_15_H_10_BrClNO, 333.96288; found, 333.96295.

##### 4.1.14.2. 6-bromo-2-(4-fluorophenyl)quinolin-4(1H)-one (14b)

Yellow solid, Yield 93%, m.p. > 250** **°C; ^1^H NMR (400** **MHz, CDCl_3_) *δ* (*ppm*): 6.39 (s, 1H, vinyl CH), 7.44 (t, 2H, fluorophenyl 2CH, *J*** **=** **8.7** **Hz), 7.72 (d, 1H, bromophenyl CH, *J*** **=** **8.8** **Hz), 7.84 (dd, 1H, bromophenyl CH, *J*** **=** **2.3, 8.8** **Hz), 7.91 (dd, 2H, fluorophenyl 2CH, *J*** **=** **5.4, 8.7** **Hz), 8.18 (d, 1H, bromophenyl CH, *J*** **=** **2.3** **Hz), 11.88 (s, 1H, NH); ^13^** **C NMR (100** **MHz, CDCl_3_) *δ* (*ppm*): 108.14, 116.44, 116.50 (CH-C-F, d, *J*** **=** **21.8** **Hz), 121.83, 126.72, 127.35, 130.43 (CH-CH-C-F, d, *J*** **=** **8.7** **Hz), 130.85, 135.07, 139.85, 149.88, 163.95 (C-F, d, *J*** **=** **248.3** **Hz), 176.02; ^19^** **F NMR (376.46** **MHz, CDCl_3_) *δ* (*ppm*): −110.21 (s); HRESIMS (*m/z*): [M** **+** **H]^+^ Calcd for C_15_H_10_BrFNO, 317.99243; found, 317.99265.

#### 4.1.15. Synthesis of 6-bromo-2-arylquinolines key intermediates (15a,b)

The key intermediates **15a,b** have been prepared from **14a,b** according to the synthetic route for **5a–c**.

##### 4.1.15.1. 6-bromo-2-(4-chlorophenyl)-4-(3-chloropropoxy)quinoline (15a)

White solid, Yield 96%, m.p. 134–136** **°C; ^1^H NMR (400** **MHz, CDCl_3_) *δ* (*ppm*): 2.44 (p, 2H, CH_2_, *J*** **=** **6** **Hz), 3.86 (t, 2H, CH_2_Cl, *J*** **=** **6.1** **Hz), 4.43 (t, 2H, CH_2_-O, *J*** **=** **5.9** **Hz), 7.15 (s, 1H, vinyl CH), 7.47 (d, 2H, chlorophenyl 2CH, *J*** **=** **8.6** **Hz), 7.76 (dd, 1H, bromophenyl CH, *J*** **=** **2.2, 9** **Hz), 7.92 (d, 1H, bromophenyl CH, *J*** **=** **9** **Hz), 8.04 (d, 2H, chlorophenyl 2CH, *J*** **=** **8.6** **Hz), 8.26 (d, 1H, bromophenyl CH, *J*** **=** **2.2** **Hz); ^13^** **C NMR (100** **MHz, CDCl_3_) *δ* (*ppm*): 31.84, 41.23, 65.02, 98.77, 119.50, 121.47, 124.04, 128.76, 128.99, 131.03, 133.57, 135.78, 138.13, 147.77, 157.72, 160.98; HRESIMS (*m/z*): [M** **+** **H]^+^ Calcd for C_18_H_15_BrCl_2_NO, 409.97088; found, 409.97205.

##### 4.1.15.2. 6-bromo-4-(3-chloropropoxy)-2-(4-fluorophenyl)quinoline (15b)

White solid, Yield 92%, m.p. 163–165** **°C; ^1^H NMR (400** **MHz, CDCl_3_) *δ* (*ppm*): 2.44 (p, 2H, CH_2_, *J*** **=** **6** **Hz), 3.86 (t, 2H, CH_2_Cl, *J*** **=** **6.1** **Hz), 4.43 (t, 2H, CH_2_-O, *J*** **=** **5.9** **Hz), 7.15 (s, 1H, vinyl CH), 7.19 (t, 2H, fluorophenyl 2CH, *J*** **=** **8.8** **Hz), 7.75 (dd, 1H, bromophenyl CH, *J*** **=** **2.3, 9** **Hz), 7.93 (d, 1H, bromophenyl CH, *J*** **=** **9** **Hz), 8.09 (dd, 2H, fluorophenyl 2CH, *J*** **=** **5.4, 8.8** **Hz), 8.27 (d, 1H, bromophenyl CH, *J*** **=** **2.3** **Hz); ^13^** **C NMR (100** **MHz, CDCl_3_) *δ* (*ppm*): 31.85, 41.22, 65.00, 98.84, 115.77 (CH-C-F, d, *J*** **=** **21.7** **Hz), 119.33, 121.37, 124.03, 129.38 (CH-CH-C-F, d, *J*** **=** **8.6** **Hz), 130.98, 133.52, 135.91 (C-CH-CH-C-F, d, *J*** **=** **3** **Hz), 147.78, 157.97, 160.94, 163.89 (C-F, d, *J*** **=** **249.4** **Hz); ^19^** **F NMR (376.46** **MHz, CDCl_3_) *δ* (*ppm*): −111.96 (s); HRESIMS (*m/z*): [M** **+** **H]^+^ Calcd for C_18_H_15_BrClFNO, 394.00041; found, 394.00122.

#### 4.1.16. Synthesis of the target 6-bromo-4-propoxy-2-arylquinolines (16a–d)

The synthesis of the target 6-bromo-4-propoxy-2-arylquinolines **16a–d** has been accomplished based on the general synthetic route for **6a–o** utilising imidazole or morpholine with the respective key intermediate **15a,b**.

##### 4.1.16.1. 4-(3-(1H-imidazol-1-yl)propoxy)-6-bromo-2-(4-chlorophenyl)quinoline (16a)

White solid, Yield 84%, m.p. 149–151** **°C; ^1^H NMR (400** **MHz, CDCl_3_) *δ* (*ppm*): 2.44 (p, 2H, CH_2_, *J*** **=** **6.2** **Hz), 4.19 (t, 2H, CH_2_-N, *J*** **=** **5.7** **Hz), 4.31 (t, 2H, CH_2_-O, *J*** **=** **6.6** **Hz), 6.95 (s, 1H, imidazole CH), 7.04 (s, 1H, vinyl CH), 7.09 (s, 1H, imidazole CH), 7.46 (d, 2H, chlorophenyl 2CH, *J*** **=** **8.7** **Hz), 7.50 (s, 1H, imidazole CH), 7.78 (dd, 1H, bromophenyl CH, *J*** **=** **2.2, 9** **Hz), 7.94 (d, 1H, bromophenyl CH, *J*** **=** **9** **Hz), 8 (d, 2H, chlorophenyl 2CH, *J*** **=** **8.7** **Hz), 8.27 (d, 1H, bromophenyl CH, *J*** **=** **2.2** **Hz); ^13^** **C NMR (100** **MHz, CDCl_3_) *δ* (*ppm*): 30.42, 43.41, 64.59, 98.77, 118.87, 119.69, 121.33, 123.79, 128.75, 129.00, 130.09, 131.15, 133.71, 135.86, 137.28, 137.99, 147.81, 157.74, 160.67; HRESIMS (*m/z*): [M** **+** **H]^+^ Calcd for C_21_H_18_BrClN_3_O, 442.03163; found, 442.03214; HPLC purity: 98.60%.

##### 4.1.16.2. 4-(3-((6-bromo-2-(4-chlorophenyl)quinolin-4-yl)oxy)propyl)morpholine (16b)

White solid, Yield 78%, m.p. 137–139** **°C; ^1^H NMR (400** **MHz, CDCl_3_) *δ* (*ppm*): 2.15 (p, 2H, CH_2_, *J*** **=** **6.7** **Hz), 2.50 (t, 4H, morpholinyl 2CH_2_, *J*** **=** **4.4** **Hz), 2.62 (t, 2H, CH_2_-N, *J*** **=** **7.1** **Hz), 3.73 (t, 4H, morpholinyl 2CH_2_, *J*** **=** **4.4** **Hz), 4.33 (t, 2H, CH_2_-O, *J*** **=** **6.3** **Hz), 7.13 (s, 1H, vinyl CH), 7.47 (d, 2H, chlorophenyl 2CH, *J*** **=** **8.6** **Hz), 7.75 (dd, 1H, bromophenyl CH, *J*** **=** **2.2, 9** **Hz), 7.92 (d, 1H, bromophenyl CH, *J*** **=** **9** **Hz), 8.03 (d, 2H, chlorophenyl 2CH, *J*** **=** **8.6** **Hz), 8.30 (d, 1H, bromophenyl CH, *J*** **=** **2.2** **Hz); ^13^** **C NMR (100** **MHz, CDCl_3_) *δ* (*ppm*): 26.14, 53.81, 55.37, 66.84, 66.98, 98.76, 119.38, 121.63, 124.21, 128.77, 128.97, 130.96, 133.49, 135.71, 138.28, 147.76, 157.75, 161.34; HRESIMS (*m/z*): [M** **+** **H]^+^ Calcd for C_22_H_23_BrClN_2_O_2_, 461.06259; found, 461.06314; HPLC purity: 99.92%.

##### 4.1.16.3. 4-(3-(1H-imidazol-1-yl)propoxy)-6-bromo-2-(4-fluorophenyl)quinoline (16c)

White solid, Yield 80%, m.p. 144–146** **°C; ^1^H NMR (400** **MHz, CDCl_3_) *δ* (*ppm*): 2.43 (p, 2H, CH_2_, *J*** **=** **6.2** **Hz), 4.19 (t, 2H, CH_2_-N, *J*** **=** **5.7** **Hz), 4.31 (t, 2H, CH_2_-O, *J*** **=** **6.6** **Hz), 6.94 (s, 1H, imidazole CH), 7.03 (s, 1H, vinyl CH), 7.09 (s, 1H, imidazole CH), 7.17 (t, 2H, fluorophenyl 2CH, *J*** **=** **8.7** **Hz), 7.50 (s, 1H, imidazole CH), 7.77 (dd, 1H, bromophenyl CH, *J*** **=** **2.3, 9** **Hz), 7.94 (d, 1H, bromophenyl CH, *J*** **=** **9** **Hz), 8.04 (dd, 2H, fluorophenyl 2CH, *J*** **=** **5.4, 8.7** **Hz), 8.27 (d, 1H, bromophenyl CH, *J*** **=** **2.3** **Hz); ^13^** **C NMR (100** **MHz, CDCl_3_) *δ* (*ppm*): 30.42, 43.41, 64.55, 98.83, 115.78 (CH-C-F, d, *J*** **=** **21.6** **Hz), 118.88, 119.51, 121.22, 123.78, 129.37 (CH-CH-C-F, d, *J*** **=** **8.5** **Hz), 130.07, 131.10, 133.65, 135.75 (C-CH-CH-C-F, d, *J*** **=** **3.1** **Hz), 137.28, 147.80, 157.97, 160.61, 163.91 (C-F, d, *J*** **=** **249.5** **Hz); ^19^** **F NMR (376.46** **MHz, CDCl_3_) *δ* (*ppm*): −111.81 (s); HRESIMS (*m/z*): [M** **+** **H]^+^ Calcd for C_21_H_18_BrFN_3_O, 426.06118; found, 426.06125; HPLC purity: 99.65%.

##### 4.1.16.4. 4-(3-((6-bromo-2-(4-fluorophenyl)quinolin-4-yl)oxy)propyl)morpholine (16d)

White solid, Yield 87%, m.p. 132–134** **°C; ^1^H NMR (400** **MHz, CDCl_3_) *δ* (*ppm*): 2.16 (p, 2H, CH_2_, *J*** **=** **6.7** **Hz), 2.51 (t, 4H, morpholinyl 2CH_2_, *J*** **=** **4.4** **Hz), 2.62 (t, 2H, CH_2_-N, *J*** **=** **7.1** **Hz), 3.73 (t, 4H, morpholinyl 2CH_2_, *J*** **=** **4.4** **Hz), 4.33 (t, 2H, CH_2_-O, *J*** **=** **6.2** **Hz), 7.12 (s, 1H, vinyl CH), 7.19 (t, 2H, fluorophenyl 2CH, *J*** **=** **8.7** **Hz), 7.75 (dd, 1H, bromophenyl CH, *J*** **=** **2.3, 9** **Hz), 7.92 (d, 1H, bromophenyl CH, *J*** **=** **9** **Hz), 8.08 (dd, 2H, fluorophenyl 2CH, *J*** **=** **5.4, 8.7** **Hz), 8.17 (d, 1H, bromophenyl CH, *J*** **=** **2.3** **Hz); ^13^** **C NMR (100** **MHz, CDCl_3_) *δ* (*ppm*): 26.14, 53.81, 55.37, 66.81, 66.97, 98.83, 115.74 (CH-C-F, d, *J*** **=** **21.7** **Hz), 119.21, 121.52, 124.19, 129.37 (CH-CH-C-F, d, *J*** **=** **8.5** **Hz), 130.91, 133.44, 136.05 (C-CH-CH-C-F, d, *J*** **=** **2.6** **Hz), 147.76, 158.01, 161.28, 163.85 (C-F, d, *J*** **=** **249.4** **Hz); ^19^** **F NMR (376.46** **MHz, CDCl_3_) *δ* (*ppm*): −112.05 (s); HRESIMS (*m/z*): [M** **+** **H]^+^ Calcd for C_22_H_23_BrFN_2_O_2_, 445.09215; found, 445.09241; HPLC purity: 99.15%.

#### 4.1.17. Synthesis of 5-aryl-2-aminoacetophenones (17a,b)

To a mixture of 1–(2-amino-5-bromophenyl)ethan-1-one **13** (1.07** **g, 5** **mmol), 4-methoxyphenylboronic acid or 2-furylboronic acid (5.5** **mmol), K_2_CO_3_ (2.28** **g, 16.5** **mmol) and Pd(PPh_3_)_4_ (0.02 equivalent, 116** **mg), dioxane (14** **ml) and H_2_O (14** **ml) were added under N_2_ atmosphere. The reaction mixture was refluxed under N_2_ at 100** **°C for 4** **h. Then, the reaction mixture was poured into ethylacetate (50** **ml) and the organic layer was separated. The aqueous layer was extracted with ethylacetate (30** **×** **3) and the organic layers were collected and washed with 1** **M HCl (100** **×** **3) then brine ((100** **×** **3). After evaporation of the organic solvent under vacuum, the residue was purified by silica gel column chromatography using hexane/ethylacetate to afford the corresponding compounds **17a,b**.

##### 4.1.17.1. 1-(4-amino-4′-methoxy-[1,1′-biphenyl]-3-yl)ethan-1-one (17a)

Yellow solid, Yield 86%, m.p. 101–103** **°C; ^1^H NMR (400** **MHz, CDCl_3_) *δ* (*ppm*): 2.63 (s, 3H, CH_3_-C=O), 3.85 (s, 3H, CH_3_-O), 6.29 (s, 2H, NH_2_), 6.71 (d, 1H, phenyl CH, *J*** **=** **8.6** **Hz), 6.97 (d, 2H, methoxyphenyl 2CH, *J*** **=** **8.8** **Hz), 7.45 (d, 2H, methoxyphenyl 2CH, *J*** **=** **8.8** **Hz), 7.48 (dd, 1H, phenyl CH, *J*** **=** **2.2, 8.6** **Hz), 7.76 (d, 1H, phenyl CH, *J*** **=** **2.2** **Hz); ^13^** **C NMR (100** **MHz, CDCl_3_) *δ* (*ppm*): 27.93, 55.39, 114.29, 117.73, 118.44, 127.36, 128.80, 129.83, 133.08, 133.24, 149.15, 158.66, 200.79; HRESIMS (*m/z*): [M** **+** **H]^+^ Calcd for C_15_H_16_NO_2_, 242.11756; found, 242.11717.

##### 4.1.17.2. 1-(2-amino-5-(furan-2-yl)phenyl)ethan-1-one (17b)

Yellow solid, Yield 70%, m.p. 87–89** **°C; ^1^H NMR (400** **MHz, CDCl_3_) *δ* (*ppm*): 2.63 (s, 3H, CH_3_), 6.36 (s, 2H, NH_2_), 6.44–6.46 (m, 2H, furyl 2CH), 6.66 (d, 1H, phenyl CH, *J*** **=** **8.6** **Hz), 7.41 (dd, 1H, furyl CH, *J*** **=** **0.9, 1.6** **Hz), 7.55 (dd, 1H, phenyl CH, *J*** **=** **2, 8.6** **Hz), 8.02 (d, 1H, phenyl CH, *J*** **=** **2** **Hz); ^13^** **C NMR (100** **MHz, CDCl_3_) *δ* (*ppm*): 27.91, 102.61, 111.55, 117.61, 118.02, 119.47, 127.24, 130.41, 141.09, 149.57, 153.73, 200.74; HRESIMS (*m/z*): [M** **+** **H]^+^ Calcd for C_12_H_12_NO_2_, 202.08626; found, 202.08594.

#### 4.1.18. Synthesis of 2,6-diarylquinolones (18a–c)

The synthesis of 2,6-diarylquinolones **18a–c** has been accomplished using 5-aryl-2-aminoacetophenones **17a,b** and the respective *p*-chloro or fluorobenzoyl chloride according to the synthetic route used for 6-bromo-2-arylquinolones **14a,b**. The 2,6-diarylquinolones **18a,b** have poor solubility for NMR spectral analysis, so the NMR spectral analysis of the soluble 6-furyl analog **18c** was used for their structural authentication in addition to HRMS for **18a,b**. Moreover, the corresponding 4-propoxy analogs **19a–c** exhibited good solubility and their spectral characterisation was enough for structural confirmation of 2,6-diarylquinolones **18a–c**.

##### 4.1.18.1. 2-(4-chlorophenyl)-6-(4-methoxyphenyl)quinolin-4(1H)-one (18a)

Yellow solid, Yield 97%, m.p. > 250** **°C; HRESIMS (*m/z*): [M + Na]^+^ Calcd for C_22_H_16_ClNO_2_Na, 384.07618; found, 384.07623.

##### 4.1.18.2. 2-(4-fluorophenyl)-6-(4-methoxyphenyl)quinolin-4(1H)-one (18b)

Yellow solid, Yield 90%, m.p. > 250** **°C; HRESIMS (*m/z*): [M** **+** **H]^+^ Calcd for C_22_H_16_FNO_2_Na, 368.10573; found, 368.10574.

##### 4.1.18.3. 2-(4-chlorophenyl)-6-(furan-2-yl)quinolin-4(1H)-one (18c)

Yellow solid, Yield 93%, m.p. > 250** **°C; ^1^H NMR (400** **MHz, CDCl_3_) *δ* (*ppm*): 6.68 (dd, 1H, furyl CH, *J*** **=** **1.6, 3.4** **Hz), 7.04 (s, 1H, vinyl CH), 7.19 (d, 1H, furyl CH, *J*** **=** **3.4** **Hz), 7.73 (d, 2H, chlorophenyl 2CH, *J*** **=** **8.6** **Hz), 7.86 (d, 1H, furyl CH, *J*** **=** **1.6** **Hz), 7.99 (d, 2H, chlorophenyl 2CH, *J*** **=** **8.6** **Hz), 8.18 (d, 1H, phenyl CH, *J*** **=** **8.9** **Hz), 8.26 (dd, 1H, phenyl CH, *J*** **=** **2. 8.9** **Hz), 8.43 (d, 1H, phenyl CH, *J*** **=** **2** **Hz); ^13^** **C NMR (100** **MHz, CDCl_3_) *δ* (*ppm*): 106.42, 108.09, 112.95, 117.53, 121.27, 122.67, 128.11, 129.59, 129.71, 130.49, 132.12, 136.85, 140.05, 144.39, 151.67, 152.32, 172.86; HRESIMS (*m/z*): [M** **+** **H]^+^ Calcd for C_19_H_13_ClNO_2_, 322.06293; found, 322.06299.

#### 4.1.19. Synthesis of 4-propoxy-2,6-diarylquinolines key intermediates (19a–c)

The key intermediates 4-propoxy-2,6-diarylquinolines **19a–c** were synthesised from 2,6-diarylquinolones **18a–c** utilising the synthetic route used for **5a–c**.

##### 4.1.19.1. 2-(4-chlorophenyl)-4-(3-chloropropoxy)-6-(4-methoxyphenyl)quinoline (19a)

Off-white solid, Yield 93%, m.p. 183–185** **°C; ^1^H NMR (400** **MHz, CDCl_3_) *δ* (*ppm*): 2.45 (p, 2H, CH_2_, *J*** **=** **6** **Hz), 3.86 (t, 2H, CH_2_Cl, *J*** **=** **6.2** **Hz), 3.88 (s, 3H, CH_3_-O), 4.45 (t, 2H, CH_2_-O, *J*** **=** **5.8** **Hz), 7.04 (d, 2H, methoxyphenyl 2CH, *J*** **=** **8.8** **Hz), 7.15 (s, 1H, vinyl CH), 7.48 (d, 2H, chlorophenyl 2CH, *J*** **=** **8.6** **Hz), 7.66 (d, 2H, methoxyphenyl 2CH, *J*** **=** **8.8** **Hz), 7.94 (dd, 1H, phenyl CH, *J*** **=** **2, 8.8** **Hz), 8.07 (d, 2H, chlorophenyl 2CH, *J*** **=** **8.6** **Hz), 8.11 (d, 1H, phenyl CH, *J*** **=** **8.8** **Hz), 8.26 (d, 1H, phenyl CH, *J*** **=** **2** **Hz); ^13^** **C NMR (100** **MHz, CDCl_3_) *δ* (*ppm*): 31.95, 41.34, 55.41, 64.90, 98.43, 114.41, 118.46, 120.52, 128.48, 128.76, 128.93, 129.59, 129.68, 133.16, 135.43, 138.06, 138.60, 148.31, 156.99, 159.47, 161.94; HRESIMS (*m/z*): [M** **+** **H]^+^ Calcd for C_25_H_22_Cl_2_NO_2_, 438.10221; found, 438.10242.

##### 4.1.19.2. 4-(3-chloropropoxy)-2-(4-fluorophenyl)-6-(4-methoxyphenyl)quinoline (19b)

Off-white solid, Yield 86%, m.p. 174–176** **°C; ^1^H NMR (400** **MHz, CDCl_3_) *δ* (*ppm*): 2.46 (p, 2H, CH_2_, *J*** **=** **6** **Hz), 3.86 (t, 2H, CH_2_Cl, *J*** **=** **6.2** **Hz), 3.88 (s, 3H, CH_3_-O), 4.46 (t, 2H, CH_2_-O, *J*** **=** **5.8** **Hz), 7.04 (d, 2H, methoxyphenyl 2CH, *J*** **=** **8.8** **Hz), 7.16 (s, 1H, vinyl CH), 7.20 (t, 2H, fluorophenyl 2CH, *J*** **=** **8.7** **Hz), 7.66 (d, 2H, methoxyphenyl 2CH, *J*** **=** **8.8** **Hz), 7.93 (dd, 1H, phenyl CH, *J*** **=** **2, 8.8** **Hz), 8.09–8.13 (m, 3H, fluorophenyl 2CH, phenyl CH), 8.27 (d, 1H, phenyl CH, *J*** **=** **2** **Hz); ^13^** **C NMR (100** **MHz, CDCl_3_) *δ* (*ppm*): 31.96, 41.35, 55.41, 64.88, 98.51, 114.41, 115.68 (CH-C-F, d, *J*** **=** **21.7** **Hz), 118.47, 120.40, 128.48, 129.34 (CH-CH-C-F, d, *J*** **=** **8.5** **Hz), 129.55, 129.63, 133.22, 136.38 (C-CH-CH-C-F, d, *J*** **=** **3** **Hz), 137.94, 148.32, 157.26, 159.44, 161.90, 163.75 (C-F, d, *J*** **=** **248.8** **Hz); ^19^** **F NMR (376.46** **MHz, CDCl_3_) *δ* (*ppm*): −112.55 (s); HRESIMS (*m/z*): [M** **+** **H]^+^ Calcd for C_25_H_22_ClFNO_2_, 422.13176; found, 422.13123.

##### 4.1.19.3. 2-(4-chlorophenyl)-4-(3-chloropropoxy)-6-(furan-2-yl)quinoline (19c)

Yellow solid, Yield 74%, m.p. 144–146** **°C; ^1^H NMR (400** **MHz, CDCl_3_) *δ* (*ppm*): 2.47 (p, 2H, CH_2_, *J*** **=** **6** **Hz), 3.88 (t, 2H, CH_2_Cl, *J*** **=** **6.2** **Hz), 4.44 (t, 2H, CH_2_-O, *J*** **=** **5.8** **Hz), 6.53 (dd, 1H, furyl CH, *J*** **=** **1.7, 3.4** **Hz), 6.79 (d, 1H, furyl CH, *J*** **=** **3.4** **Hz), 7.14 (s, 1H, vinyl CH), 7.47 (d, 2H, chlorophenyl 2CH, *J*** **=** **8.6** **Hz), 7.55 (d, 1H, furyl CH, *J*** **=** **1.7** **Hz), 7.98 (dd, 1H, phenyl CH, *J*** **=** **1.9, 8.8** **Hz), 8.06 (d, 3H, chlorophenyl 2CH, phenyl CH, *J*** **=** **8.6** **Hz), 8.38 (d, 1H, phenyl CH, *J*** **=** **1.9** **Hz); ^13^** **C NMR (100** **MHz, CDCl_3_) *δ* (*ppm*): 31.94, 41.36, 64.95, 98.60, 106.15, 111.95, 115.65, 120.52, 126.56, 128.02, 128.72, 128.93, 129.71, 135.50, 138.43, 142.63, 148.55, 153.66, 157.06, 161.94; HRESIMS (*m/z*): [M** **+** **H]^+^ Calcd for C_22_H_18_Cl_2_NO_2_, 398.07091; found, 398.07150.

#### 4.1.20. Synthesis of the target 4-propoxy-2,6-diarylquinolines (20a–f)

The synthesis of the target 4-popoxy-2,6-diarylquinolines **20a–f** has been conducted by reaction of imidazole or morpholine with the corresponding key intermediate **19a–c** under the same conditions used for synthesis of **6a–o**.

##### 4.1.20.1. 4-(3-(1H-imidazol-1-yl)propoxy)-2-(4-chlorophenyl)-6-(4-methoxyphenyl)quinoline (20a)

White solid, Yield 80%, m.p. 210–212** **°C; ^1^H NMR (400** **MHz, CDCl_3_) *δ* (*ppm*): 2.45 (p, 2H, CH_2_, *J*** **=** **6.2** **Hz), 3.88 (s, 3H, CH_3_-O), 4.22 (t, 2H, CH_2_-N, *J*** **=** **5.7** **Hz), 4.31 (t, 2H, CH_2_-O, *J*** **=** **6.6** **Hz), 6.94 (s, 1H, imidazole CH), 7.04 (d, 2H, methoxyphenyl 2CH, *J*** **=** **8.8** **Hz), 7.05 (s, 1H, vinyl CH), 7.08 (s, 1H, imidazole CH), 7.47 (d, 2H, chlorophenyl 2CH, *J*** **=** **8.6** **Hz), 7.50 (s, 1H, imidazole CH), 7.67 (d, 2H, methoxyphenyl 2CH, *J*** **=** **8.8** **Hz), 7.95 (dd, 1H, phenyl CH, *J*** **=** **2, 8.8** **Hz), 8.03 (d, 2H, chlorophenyl 2CH, *J*** **=** **8.6** **Hz), 8.13 (d, 1H, phenyl CH, *J*** **=** **8.8** **Hz), 8.27 (d, 1H, phenyl CH, *J*** **=** **2** **Hz); ^13^** **C NMR (100** **MHz, CDCl_3_) *δ* (*ppm*): 30.50, 43.45, 55.43, 64.38, 98.41, 114.47, 118.18, 118.90, 120.40, 128.50, 128.74, 128.94, 129.74, 129.83, 130.05, 133.11, 135.51, 137.30, 138.30, 138.45, 148.35, 156.97, 159.54, 161.61; HRESIMS (*m/z*): [M** **+** **H]^+^ Calcd for C_28_H_25_ClN_3_O_2_, 470.16298; found, 470.16299; HPLC purity: 99.88%.

##### 4.1.20.2. 4-(3-((2-(4-chlorophenyl)-6-(4-methoxyphenyl)quinolin-4-yl)oxy)propyl)morpholine (20b)

White solid, Yield 89%, m.p. 173–175** **°C; ^1^H NMR (400** **MHz, CDCl_3_) *δ* (*ppm*): 2.18 (p, 2H, CH_2_, *J*** **=** **6.7** **Hz), 2.50 (t, 4H, morpholinyl 2CH_2_, *J*** **=** **4.4** **Hz), 2.63 (t, 2H, CH_2_-N, *J*** **=** **7.1** **Hz), 3.73 (t, 4H, morpholinyl 2CH_2_, *J*** **=** **4.4** **Hz), 3.88 (s, 3H, CH_3_-O), 4.35 (t, 2H, CH_2_-O, *J*** **=** **6.3** **Hz), 7.03 (d, 2H, methoxyphenyl 2CH, *J*** **=** **8.8** **Hz), 7.13 (s, 1H, vinyl CH), 7.48 (d, 2H, chlorophenyl 2CH, *J*** **=** **8.6** **Hz), 7.67 (d, 2H, methoxyphenyl 2CH, *J*** **=** **8.8** **Hz), 7.93 (dd, 1H, phenyl CH, *J*** **=** **2.1, 8.8** **Hz), 8.06 (d, 2H, chlorophenyl 2CH, *J*** **=** **8.6** **Hz), 8.10 (d, 1H, phenyl CH, *J*** **=** **8.8** **Hz), 8.30 (d, 1H, phenyl CH, *J*** **=** **2.1** **Hz); ^13^** **C NMR (100** **MHz, CDCl_3_) *δ* (*ppm*): 26.22, 53.83, 55.41, 55.48, 66.64, 66.98, 98.40, 114.40, 118.59, 120.66, 128.44, 128.77, 128.90, 129.48, 129.63, 133.20, 135.37, 137.91, 138.74, 148.30, 157.02, 159.44, 162.28; HRESIMS (*m/z*): [M** **+** **H]^+^ Calcd for C_29_H_30_ClN_2_O_3_, 489.19395; found, 489.19415; HPLC purity: 96.78%.

##### 4.1.20.3. 4-(3-(1H-imidazol-1-yl)propoxy)-2-(4-fluorophenyl)-6-(4-methoxyphenyl)quinoline (20c)

White solid, Yield 79%, m.p. 176–178** **°C; ^1^H NMR (400** **MHz, CDCl_3_) *δ* (*ppm*): 2.44 (p, 2H, CH_2_, *J*** **=** **6.2** **Hz), 3.88 (s, 3H, CH_3_-O), 4.22 (t, 2H, CH_2_-N, *J*** **=** **5.7** **Hz), 4.30 (t, 2H, CH_2_-O, *J*** **=** **6.6** **Hz), 6.94 (s, 1H, imidazole CH), 7.04 (s, 1H, vinyl CH), 7.06 (d, 2H, methoxyphenyl 2CH, *J*** **=** **8.8** **Hz), 7.07 (s, 1H, imidazole CH), 7.18 (t, 2H, fluorophenyl 2CH, *J*** **=** **8.8** **Hz), 7.50 (s, 1H, imidazole CH), 7.67 (d, 2H, methoxyphenyl 2CH, *J*** **=** **8.8** **Hz), 7.95 (dd, 1H, phenyl CH, *J*** **=** **2, 8.8** **Hz), 8.07 (dd, 2H, fluorophenyl 2CH, *J*** **=** **5.4, 8.8** **Hz), 8.13 (d, 1H, phenyl CH, *J*** **=** **8.8** **Hz), 8.27 (d, 1H, phenyl CH, *J*** **=** **2** **Hz); ^13^** **C NMR (100** **MHz, CDCl_3_) *δ* (*ppm*): 30.51, 43.46, 55.42, 64.36, 98.48, 114.46, 115.69 (CH-C-F, d, *J*** **=** **21.7** **Hz), 118.19, 118.91, 120.27, 128.49, 129.33 (CH-CH-C-F, d, *J*** **=** **8.3** **Hz), 129.70, 129.77, 130.02, 133.15, 136.22, (C-CH-CH-C-F, d, *J*** **=** **3.3** **Hz), 137.29, 138.16, 148.34, 157.23, 159.52, 161.57, 163.77 (C-F, d, *J*** **=** **249.2** **Hz); ^19^** **F NMR (376.46** **MHz, CDCl_3_) *δ* (*ppm*): −112.39 (s); HRESIMS (*m/z*): [M** **+** **H]^+^ Calcd for C_28_H_25_FN_3_O_2_, 454.19326; found, 454.19244; HPLC purity: 99.21%.

##### 4.1.20.4. 4-(3-((2-(4-fluorophenyl)-6-(4-methoxyphenyl)quinolin-4-yl)oxy)propyl)morpholine (20d)

White solid, Yield 82%, m.p. 168–170** **°C; ^1^H NMR (400** **MHz, CDCl_3_) *δ* (*ppm*): 2.18 (p, 2H, CH_2_, *J*** **=** **6.7** **Hz), 2.50 (t, 4H, morpholinyl 2CH_2_, *J*** **=** **4.4** **Hz), 2.63 (t, 2H, CH_2_-N, *J*** **=** **7.1** **Hz), 3.73 (t, 4H, morpholinyl 2CH_2_, *J*** **=** **4.4** **Hz), 3.88 (s, 3H, CH_3_-O), 4.36 (t, 2H, CH_2_-O, *J*** **=** **6.3** **Hz), 7.03 (d, 2H, methoxyphenyl 2CH, *J*** **=** **8.8** **Hz), 7.13 (s, 1H, vinyl CH), 7.20 (t, 2H, fluorophenyl 2CH, *J*** **=** **8.7** **Hz), 7.67 (d, 2H, methoxyphenyl 2CH, *J*** **=** **8.8** **Hz), 7.93 (dd, 1H, phenyl CH, *J*** **=** **2, 8.8** **Hz), 8.09–8.12 (m, 3H, fluorophenyl 2CH, phenyl CH), 8.30 (d, 1H, phenyl CH, *J*** **=** **2** **Hz); ^13^** **C NMR (100** **MHz, CDCl_3_) *δ* (*ppm*): 26.23, 53.83, 55.40, 55.48, 66.62, 66.98, 98.48, 114.40, 115.65 (CH-C-F, d, *J*** **=** **21.5** **Hz), 118.59, 120.54, 128.43, 129.34, (CH-CH-C-F, d, *J*** **=** **8.5** **Hz), 129.44, 129.57, 133.25, 136.51 (C-CH-CH-C-F, d, *J*** **=** **3** **Hz), 137.79, 148.30, 157.29, 159.42, 162.24, 163.71 (C-F, d, *J*** **=** **248.8** **Hz); ^19^** **F NMR (376.46** **MHz, CDCl_3_) *δ* (*ppm*): −112.63 (s); HRESIMS (*m/z*): [M** **+** **H]^+^ Calcd for C_29_H_30_FN_2_O_3_, 473.22422; found, 473.22357; HPLC purity: 99.75%.

##### 4.1.20.5. 4-(3-(1H-imidazol-1-yl)propoxy)-2-(4-chlorophenyl)-6-(furan-2-yl)quinoline (20e)

Yellow solid, Yield 79%, m.p. 130–132** **°C; ^1^H NMR (400** **MHz, CDCl_3_) *δ* (*ppm*): 2.47 (p, 2H, CH_2_, *J*** **=** **6.2** **Hz), 4.22 (t, 2H, CH_2_-N, *J*** **=** **5.7** **Hz), 4.34 (t, 2H, CH_2_-O, *J*** **=** **6.6** **Hz), 6.55 (dd, 1H, furyl CH, *J*** **=** **1.6, 3.3** **Hz), 6.82 (d, 1H, furyl CH, *J*** **=** **3.3** **Hz), 6.96 (s, 1H, imidazole CH), 7.04 (s, 1H, vinyl CH), 7.09 (s, 1H, imidazole CH), 7.46 (d, 2H, chlorophenyl 2CH, *J*** **=** **8.5** **Hz), 7.53 (s, 1H, imidazole CH), 7.56 (d, 1H, furyl CH, *J*** **=** **1.6** **Hz), 7.99 (dd, 1H, phenyl CH, *J*** **=** **1.8, 8.7** **Hz), 8.02 (d, 2H, chlorophenyl 2CH, *J*** **=** **8.5** **Hz), 8.07 (d, 1H, phenyl CH, *J*** **=** **8.7** **Hz), 8.41 (d, 1H, phenyl CH, *J*** **=** **1.8** **Hz); ^13^** **C NMR (100** **MHz, CDCl_3_) *δ* (*ppm*): 30.48, 43.47, 64.40, 98.63, 106.32, 112.03, 115.33, 118.94, 120.41, 126.70, 128.19, 128.71, 128.95, 129.87, 129.96, 135.58, 137.29, 138.30, 142.72, 148.59, 153.57, 157.09, 161.62; HRESIMS (*m/z*): [M** **+** **H]^+^ Calcd for C_25_H_21_ClN_3_O_2_, 430.13168; found, 430.13174; HPLC purity: 95.27%.

##### 4.1.20.6. 4-(3-((2-(4-chlorophenyl)-6-(furan-2-yl)quinolin-4-yl)oxy)propyl)morpholine (20f)

Yellow solid, Yield 74%, m.p. 136–138** **°C; ^1^H NMR (400** **MHz, CDCl_3_) *δ* (*ppm*): 2.19 (p, 2H, CH_2_, *J*** **=** **6.7** **Hz), 2.52 (t, 4H, morpholinyl 2CH_2_, *J*** **=** **4.4** **Hz), 2.65 (t, 2H, CH_2_-N, *J*** **=** **7.1** **Hz), 3.74 (t, 4H, morpholinyl 2CH_2_, *J*** **=** **4.4** **Hz), 4.35 (t, 2H, CH_2_-O, *J*** **=** **6.3** **Hz), 6.53 (dd, 1H, furyl CH, *J*** **=** **1.8, 3.4** **Hz), 6.79 (dd, 1H, furyl CH, *J*** **=** **0.5, 3.4** **Hz), 7.12 (s, 1H, vinyl CH), 7.47 (d, 2H, chlorophenyl 2CH, *J*** **=** **8.6** **Hz), 7.54 (dd, 1H, furyl CH, *J*** **=** **0.5, 1.8** **Hz), 7.98 (dd, 1H, phenyl CH, *J*** **=** **1.9, 8.8** **Hz), 8.05 (d, 3H, chlorophenyl 2CH, phenyl CH, *J*** **=** **8.6** **Hz), 8.42 (d, 1H, phenyl CH, *J*** **=** **1.9** **Hz); ^13^** **C NMR (100** **MHz, CDCl_3_) *δ* (*ppm*): 26.19, 53.84, 55.49, 66.71, 67.00, 98.60, 106.04, 111.94, 115.86, 120.67, 126.49, 127.93, 128.73, 128.91, 129.67, 135.44, 138.60, 142.58, 148.56, 153.75, 157.12, 162.29; HRESIMS (*m/z*): [M** **+** **H]^+^ Calcd for C_26_H_26_ClN_2_O_3_, 449.16265; found, 449.16275; HPLC purity: 98.79%.

### 4.2. *In vitro* anticancer activity

#### 4.2.1. *In vitro* antiproliferative assay

The antiproliferative assay against five cancer cell lines representing three different tumour subpanels, including colorectal (DLD-1, HCT-116), breast (MDMBA-231, MCF-7), and cervical (HeLa) cell lines was conducted using MTT assay. Cells were seeded at 1** **×** **10^4^ cells/well and cultured overnight in a 96-well plate. The cells were treated with either 10 µМ of tested quinolines **6a–o, 8a,b, 10a,b, 12a–d, 16a–d**, and **20a–f**, or DMSO as a negative control. After 24** **h, the cells washed with phosphate buffered saline (PBS, Invitrogen Gibco) and incubated with 20** **µl of MTT solution (2** **mg/ml) for 4** **h at 37** **°C. Then, 150** **µl DMSO was used to solubilise MTT formazan crystals. Finally, the plates were shaken, and the optical density was determined at 570** **nm using ELISA plate reader. At least, three independent experiments were performed. Percentage of growth inhibition was determined as (1-[OD of treated cells/OD of control cells]). On the other hand, using the MTT assay, we tested the effect of different concentrations (0.5, 1, 10, 30, 50, and 100** **µM)) of the synthesised compounds on colorectal cancer cell lines (DLD-1 and HCT-116), using DMSO as a negative control, whereas Gefitinib and TAE226 were used as positive controls. The IC_50_ values were calculated using Prism v0.8 software (GraphPad Software Inc., La Jolla, CA).

#### 4.2.2. Apoptosis assay

The apoptotic effect of the most potent antiproliferative agents **6f, 6h, 6i, 16d**, and **20f** on DLD-1 colorectal cancer cell line was investigated using the annexin V/propidium iodide (AV/PI) staining kit (BioLegend, San Diego, CA, USA) according to the manufacturer's instructions. DLD-1 cells were treated with 3** **µM of the most potent antiproliferative compounds **6f, 6h, 6i, 16d**, and **20f** or DMSO as a negative control then incubated for 24** **h. Flow cytometric analysis was conducted using FACS Calibur flow cytometer (BD Biosciences, San Jose, CA, USA).

### 4.3. Topoisomerase I-mediated DNA cleavage assay

A 3′-[^32^P]-labeled 117-bp DNA substrate oligonucleotide was prepared as described previously^39^. Radiolabeled DNA was incubated with recombinant human TOP1 in 20** **µL reaction buffer (10** **mmol/L Tris-HCl, pH 7.5, 50** **mmol/L KCl, 5** **mmol/L MgCl2, 0.1** **mmol/L EDTA, and 15** **µg/mL BSA) at 30** **°C for 20** **min in the presence of the indicated drug concentrations. Reactions were terminated by adding SDS (0.5% final concentration) followed by the addition of two volumes of loading dye (80% formamide, 10** **mmol/L sodium hydroxide, 1** **mmol/L sodium EDTA, 0.1% xylene cyanol, and 0.1% bromophenol blue). Aliquots of reaction mixtures were subjected to 20% denaturing PAGE. Gels were dried and visualised by using PhosphorImager and Image Quant software (Molecular Dynamics).

### 4.4. Kinases inhibitory assay

The IC_50_ values of the tested compounds, Gefitinib and TAE226 on different nine kinases (EGFR, FAK, FRK, IGF-1R, BTK, c-Src, VEGFR-1, VEGFR-2, HER-2) were estimated utilising Z`-LYTE® technology, which is based on FRET (Invitrogen/Life Technologies).

### 4.5. *In silico* molecular docking

Ligands were converted to 3D structures and minimised using Avogadro^48^. Ligands were prepared and converted to pdbqt files using PyRx^49^. Protein targets were downloaded from the protein data bank under the codes 1M17 for EGFR and 2JKM for FAK^50^. Co-crystalised ligands were extracted form pdb files and prepared similar to the tested ligands. Docking was done using Autodock Vina^51^ in a grid box of 25^3^** **Å^3^ centred on the co-crystalised ligand with exhaustiveness of 16. Visualisation and 3D images were prepared using PyMol^52^.

### 4.6. Molecular dynamics (MD) simulation

Missing loops in the 3D structures of the protein were constructed using Swiss-Model^43^ before starting molecular dynamics steps. All atom molecular dynamics simulations were performed using GROMACS 2020.3^53^ for the selected protein-ligand complexes as reported earlier^54^. In brief, SwissParam server^55^ was used for ligands parameterisation while Charmm36 all-atom force field^56^ was used to generate topology files for the protein. Ligand coordinates obtained from docking studies were used to build complexes which were boxed in a dodecahedron box and then solvated with TIP3P^57^ explicit water. Systems were neutralised by the addition of required number of Na^+^ or Cl^−^ ions. Systems energy was minimised with a maximum force of 1000** **kJ mol^−1^** **nm^−1^ using steepest descent algorithm. Equilibration using NVT and NPT ensembles for 1** **ns each was done afterward then production run was done for 50** **ns. Temperature was kept at 300** **K using the V-rescale algorithm^58^ while pressure was controlled using the Parrinello-Rahman barostat^59^ as required. The LINear Constraint Solver (LINCS) algorithm^60^ and Particle mesh Ewald (PME) method^61^ were used for bond’s length constraints and long-range electrostatics calculations, respectively. Two femtosecond timestep was used for all simulations. Van der Waals distance cut-off (rvdw) was set to 1.2** **nm. Trajectories from the production run were used for analysis using trajconv after correction of periodic boundary condition (PBC).

## Supplementary Material

Supplemental Material
