# Supplementary material for: 2-Arylquinolines as novel anticancer agents with dual EGFR/FAK kinase inhibitory activity: synthesis, biological evaluation, and molecular modelling insights
Source: J Enzyme Inhib Med Chem. 2021 Dec 20;37(1):349–72. doi: 10.1080/14756366.2021.2015344 (PMC8725837; doi:10.1080/14756366.2021.2015344)
Supplement: Supplemental Material [file IENZ_A_2015344_SM3876.pdf]

Supplementary Data

Discovery of novel 4-propoxy-2-arylquinolines as dual EGFR and FAK inhibitors with *in vitro* antitumor activity

Mostafa M. Elbadawi [a, b, *], Wagdy M. Eldehna [b], Amer Ali Abd El-Hafeez [c, d], Warda R. Somaa [e], Amgad Albohy [f], Keli K. Agama [g], Pradipta Ghosh [d, h, i, j], Yves Pommier [g], and Manabu Abe [a, **]

[a] *Department of Chemistry, Graduate School of Science, Hiroshima University, 1-3-1 Kagamiyama, Higashi-Hiroshima, Hiroshima 739-8526, Japan*

[b] *Department of Pharmaceutical Chemistry, Faculty of Pharmacy, Kafrelsheikh University, Kafrelsheikh 33516, Egypt*

[c] *Pharmacology and Experimental Oncology Unit, Cancer Biology Department, National Cancer Institute, Cairo University, Cairo, Egypt*

[d] *Department of Cellular and Molecular Medicine, University of California San Diego, La Jolla, CA, USA*

[e] *Faculty of Pharmacy, Kafrelsheikh University, Kafrelsheikh 33516, Egypt*

[f] *Department of Pharmaceutical Chemistry, Faculty of Pharmacy, The British University in Egypt (BUE), El-Sherouk City, Suez Desert Road, Cairo 11837, Egypt*

[g] *Developmental Therapeutics Branch & Laboratory of Molecular Pharmacology, Center for Cancer Research, National Cancer Institute, NIH, Bethesda, Maryland, USA*

[h] *Department of Medicine, University of California San Diego, La Jolla, CA, USA*

[i] *Moores Comprehensive Cancer Center, University of California San Diego, La Jolla, CA, USA*

[j] *Veterans Affairs Medical Center, La Jolla, CA, USA*

* Corresponding author: mostafa_elbadawi@pharm.kfs.edu.eg

** Corresponding author: mabe@hiroshima-u.ac.jp

Table of contents

Content	Page
NMR data………………………………………………………………	S2
Topoisomerase I-mediated DNA cleavage assay………………………	S114
Table S1. Kinase profiling…………………………………………………	S118
Representative HPLC traces………………………………………………	S119

NMR data

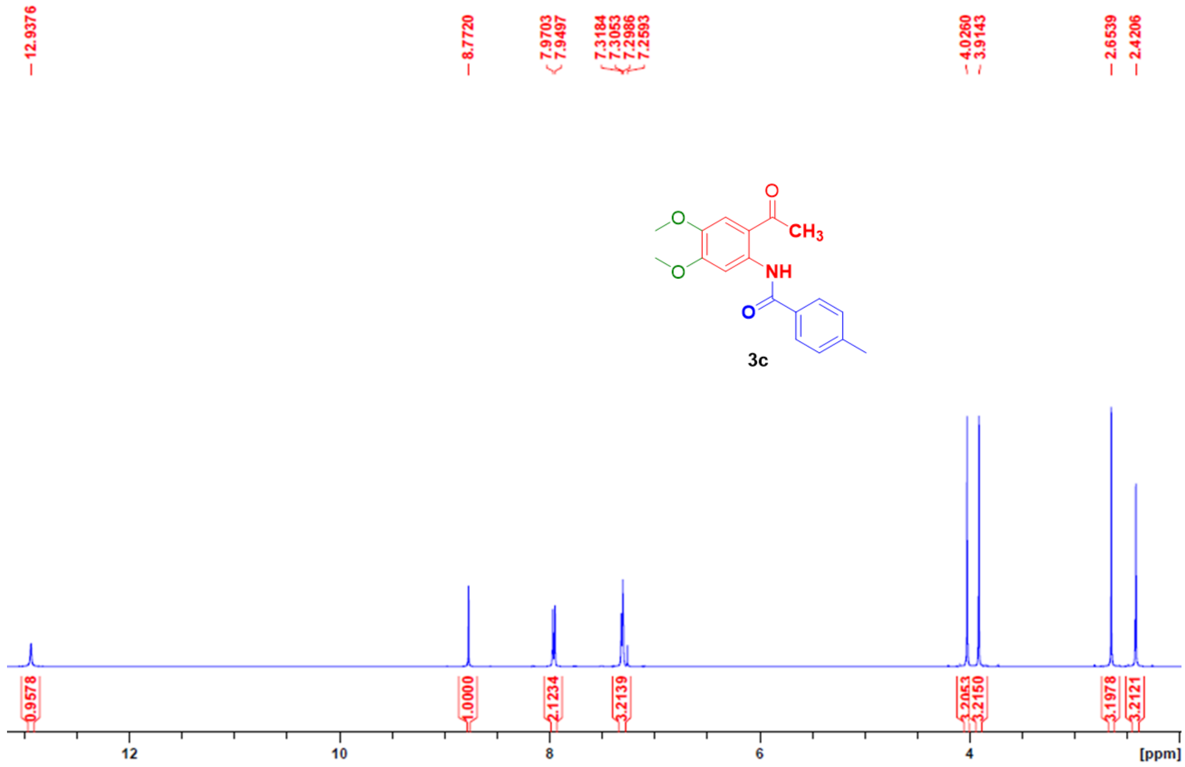
12.9376
8.7720
7.9703
7.9497
7.3184
7.3053
7.2986
7.2593
4.0260
3.9143
2.6539
2.4206
CH3
NH
O
3c
0.9578
1.0000
2.1234
3.2139
3.2053
3.2150
3.1978
3.2121
12
10
8
6
4
[ppm]

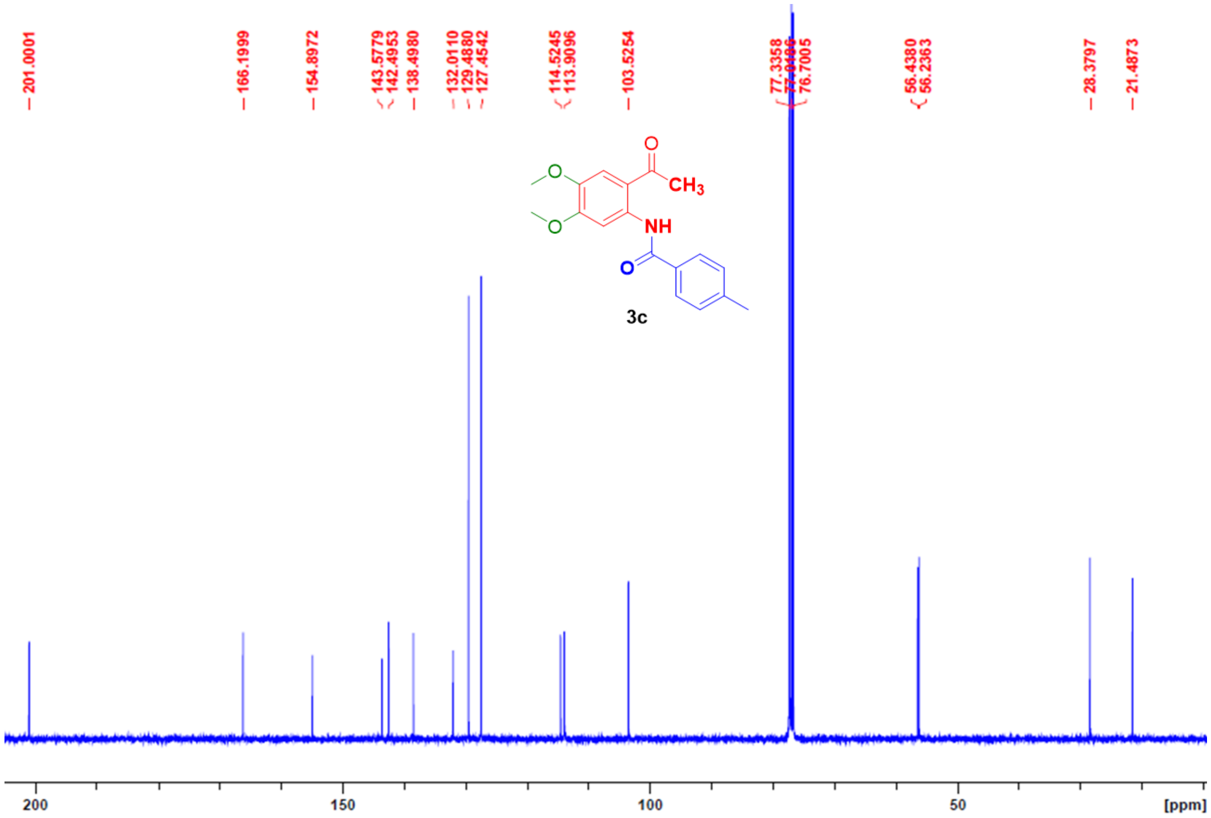
201.0001
166.1999
154.8972
143.5779
142.4953
138.4980
132.0110
129.4880
127.4542
114.5245
113.9096
103.5254
77.3358
76.7005
56.4380
56.2363
28.3797
21.4873
CH3
NH
O
3c
200
150
100
50
[ppm]

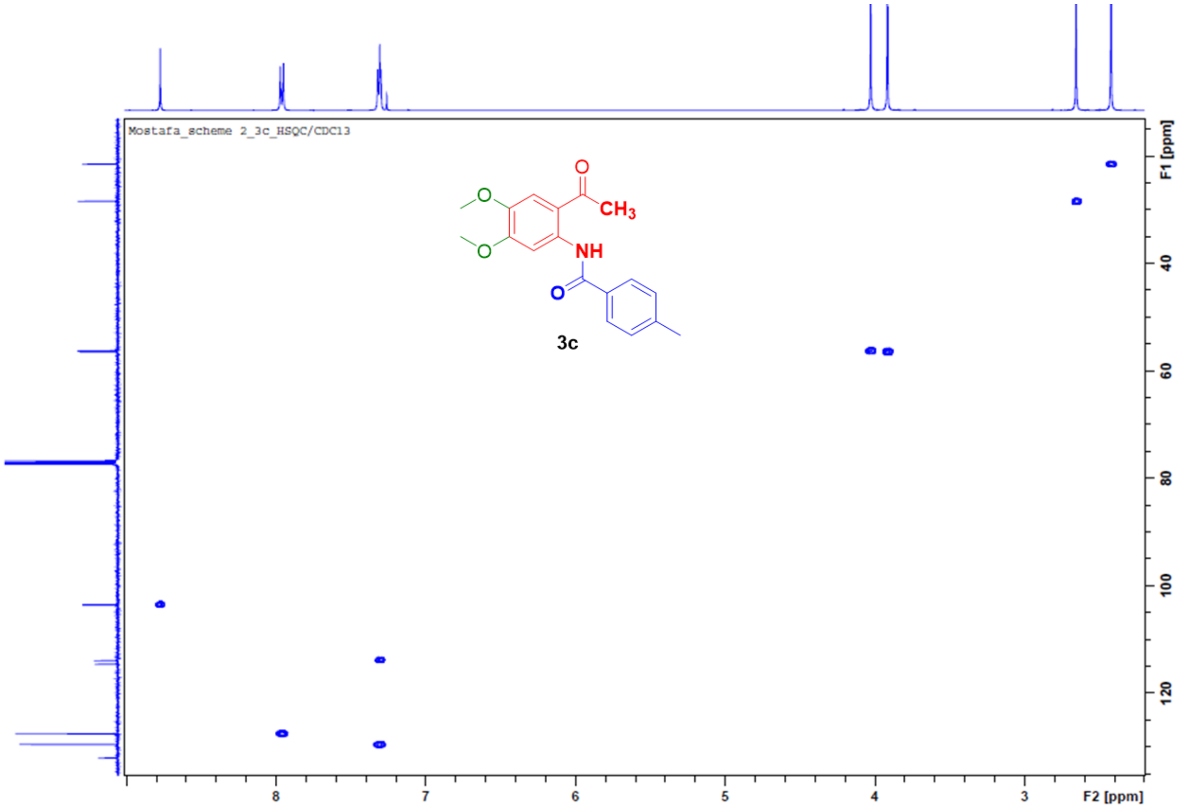
Mostafa_scheme 2_3c_HSQC/CDCl3
O
CH3
O
O
NH
O
3c
F1 [ppm]
40
60
80
100
120
8
7
6
5
4
3
F2 [ppm]

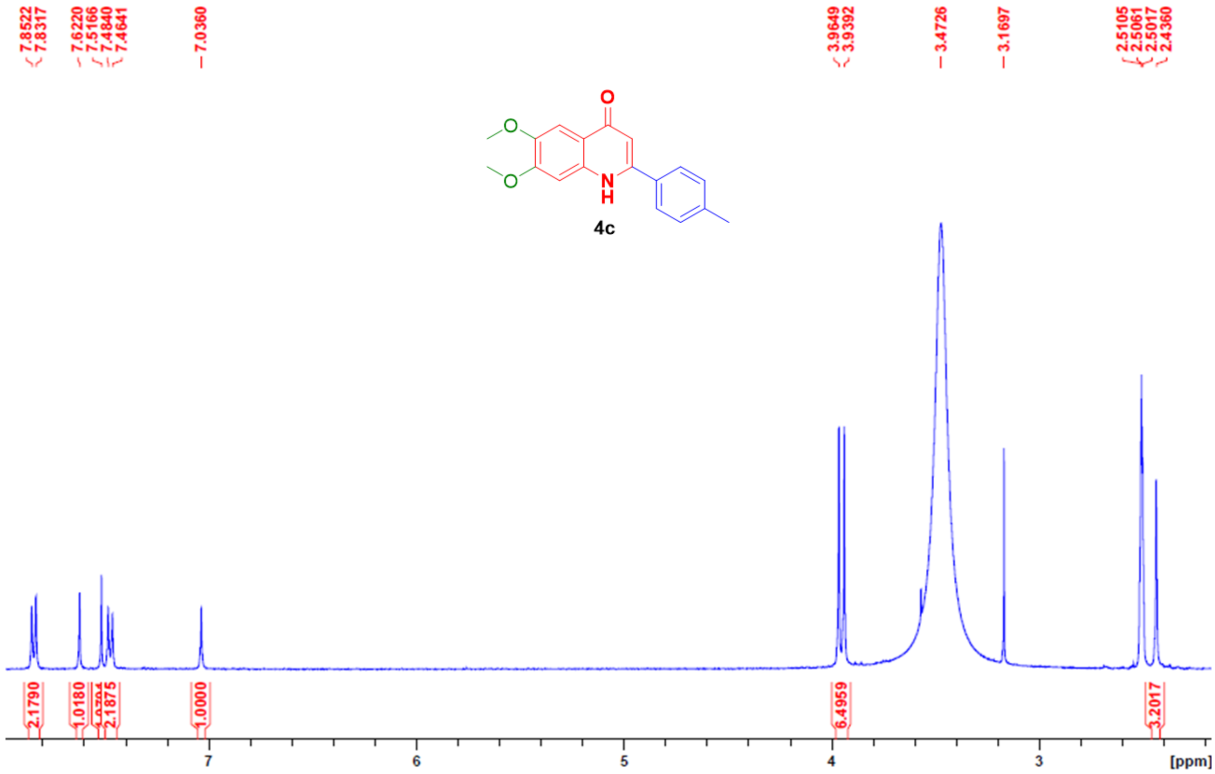
7.8522
7.8317
7.6220
7.5166
7.4840
7.4641
7.0360
3.9649
3.9392
3.4726
3.1697
2.5105
2.5061
2.5017
2.4360
O
O
O
N
H
4c
2.1790
1.0180
2.1875
1.0000
6.4959
3.2017
7
6
5
4
3
[ppm]

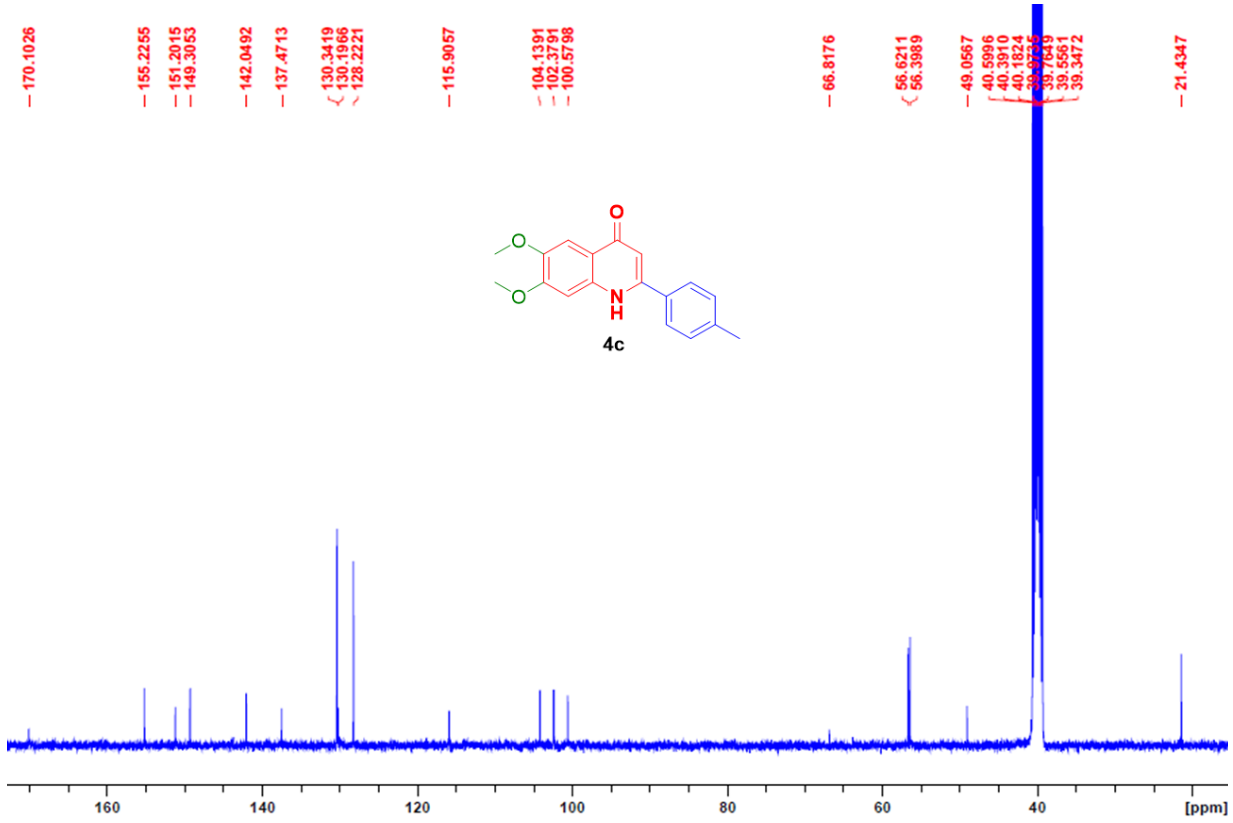
170.1026
155.2255
151.2015
149.3053
142.0492
137.4713
130.3419
130.1966
128.2221
115.9057
104.1391
102.3791
100.5798
66.8176
56.6211
56.3989
49.0567
40.5996
40.3910
40.1824
39.7649
39.5561
39.3472
21.4347
O
N
H
4c
160
140
120
100
80
60
40
[ppm]

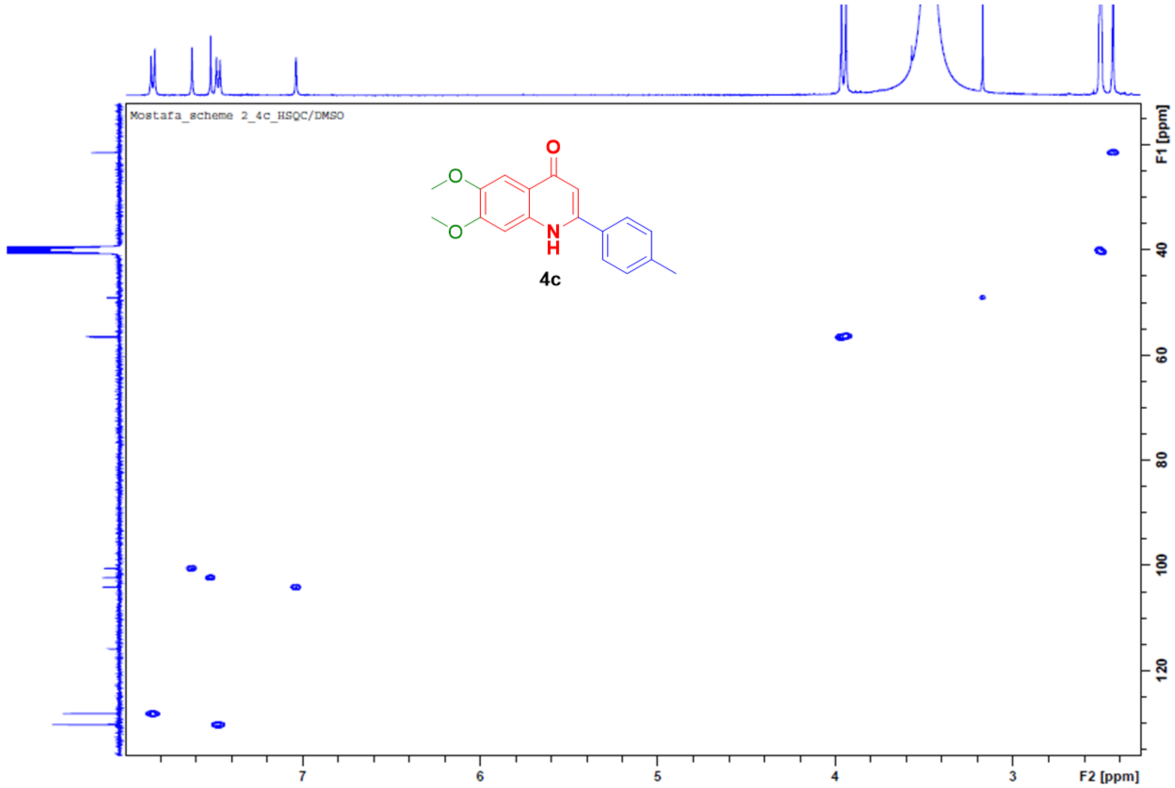
Mostafa_scheme 2_4c_HSQC/DMSO
O
N
H
4c
F1 [ppm]
40
60
80
100
120
7
6
5
4
3
F2 [ppm]

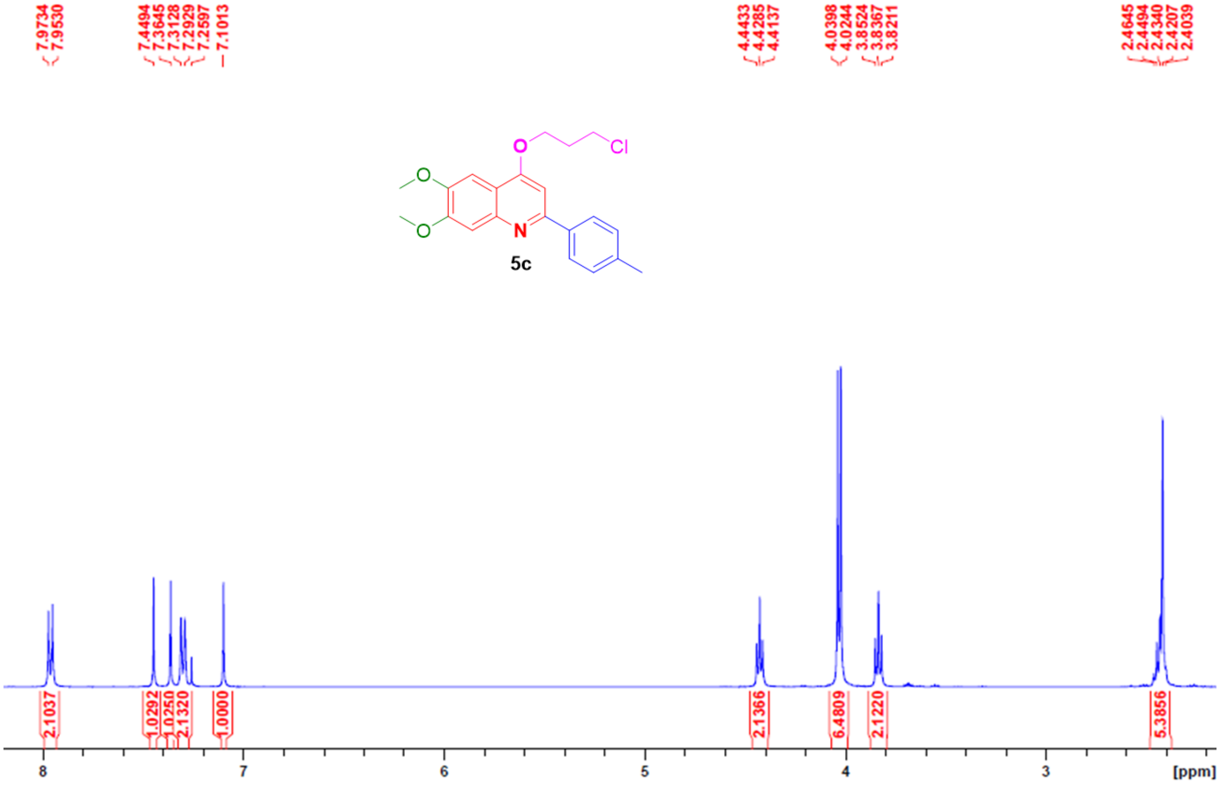
7.9734
7.9530
7.4494
7.3645
7.3128
7.2929
7.2597
7.1013
4.4433
4.4285
4.4137
4.0398
4.0244
3.8524
3.8367
3.8211
2.4645
2.4494
2.4340
2.4207
2.4039
O
Cl
N
5c
2.1037
1.0292
1.0250
2.1320
1.0000
2.1366
6.4809
2.1220
5.3856
8
7
6
5
4
3
[ppm]

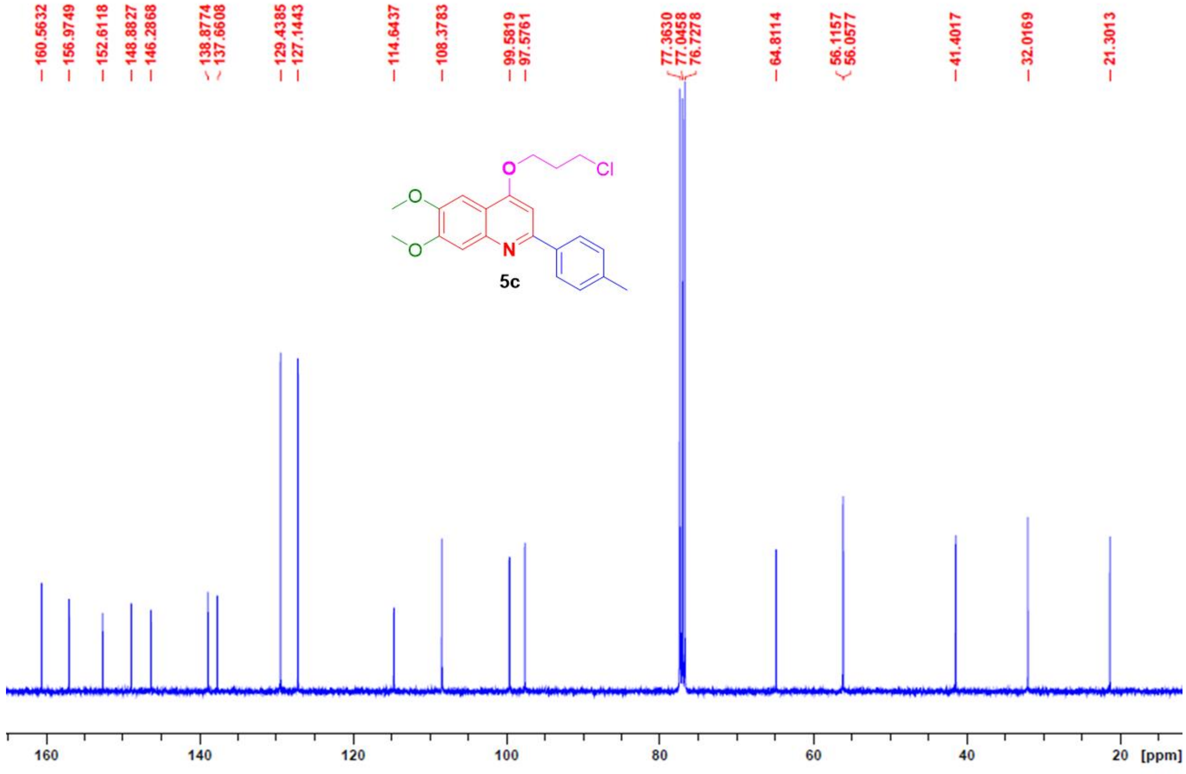
160.5632
156.9749
152.6118
148.8827
146.2868
138.8774
137.6608
129.4385
127.1443
114.6437
108.3783
99.5819
97.5761
77.3630
77.0458
76.7278
64.8114
56.1157
56.0577
41.4017
32.0169
21.3013
O
Cl
N
5c
160
140
120
100
80
60
40
20
[ppm]

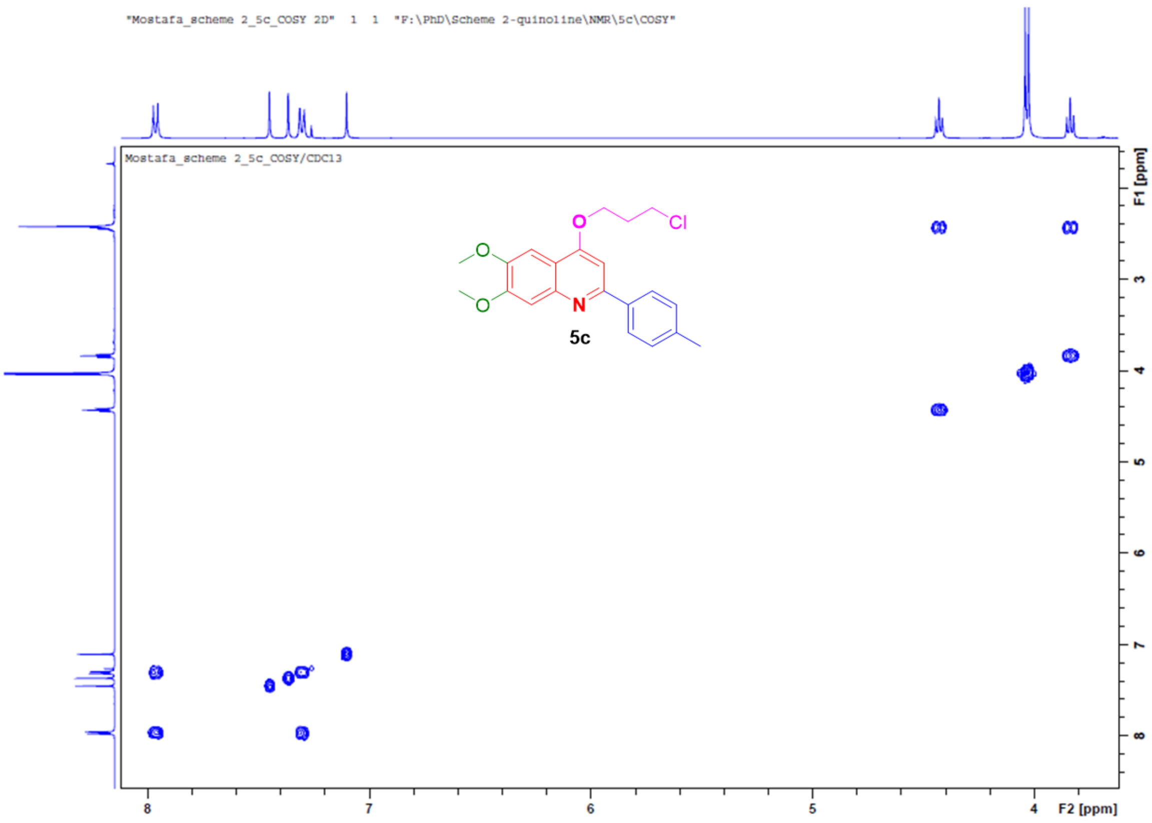
"Mostafa_scheme 2_5c_COSY 2D" 1 1 "F:\PhD\Scheme 2-quinoline\NMR\5c\COSY"
Mostafa_scheme 2_5c_COSY/CDCl3
5c
F1 [ppm]
F2 [ppm]

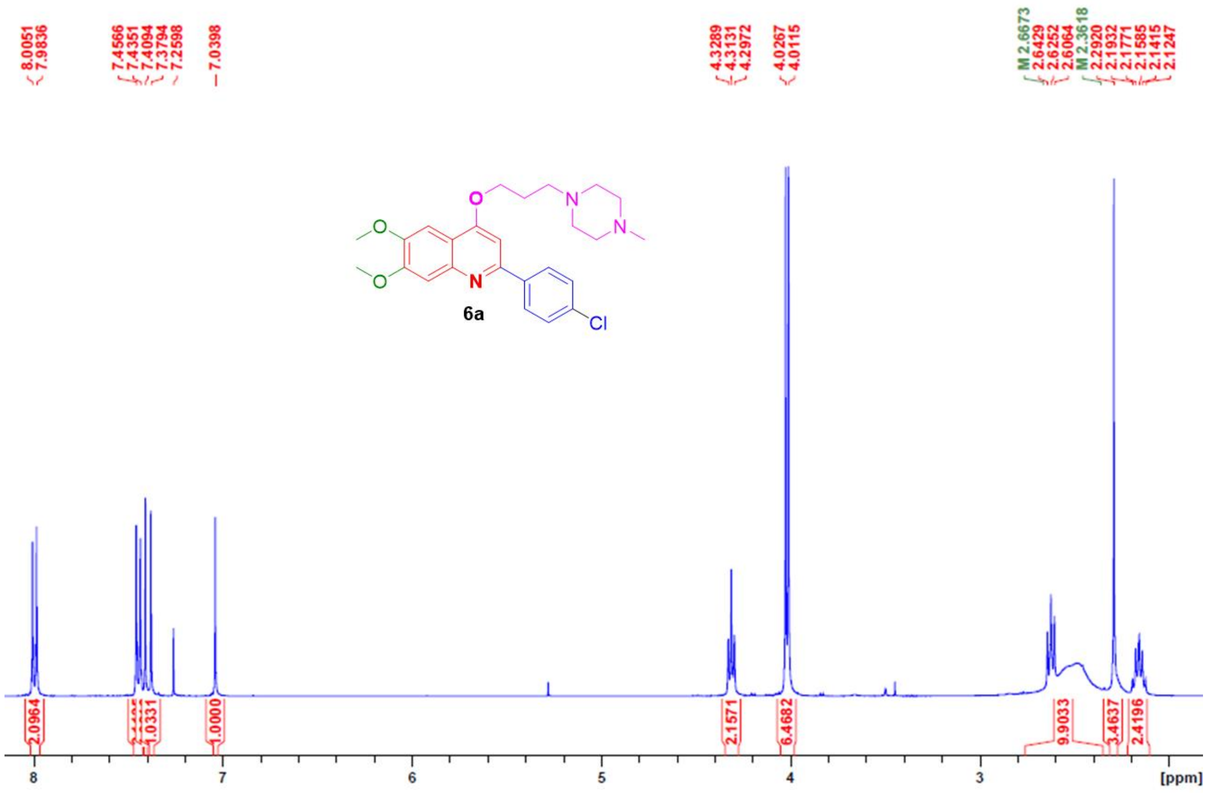
8.0051
7.9836
7.4566
7.4351
7.4094
7.3794
7.2598
7.0398
4.3289
4.3131
4.2972
4.0267
4.0115
M 2.6673
2.6429
2.6252
2.6064
M 2.3618
2.2920
2.1932
2.1771
2.1585
2.1415
2.1247
6a
2.0964
1.0331
1.0000
2.1571
6.4682
9.9033
3.4637
2.4196
[ppm]

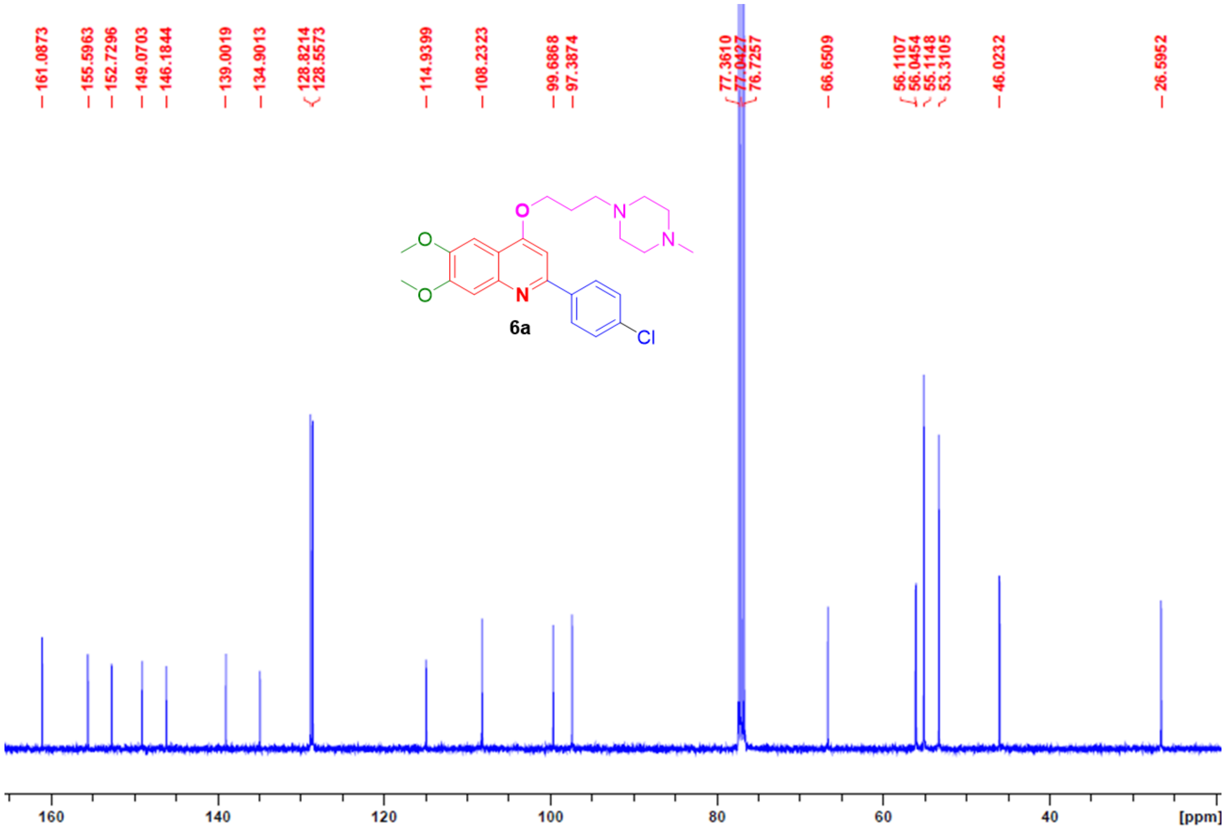
161.0873
155.5963
152.7296
149.0703
146.1844
139.0019
134.9013
128.8214
128.5573
114.9399
108.2323
99.6868
97.3874
77.3610
77.0427
76.7257
66.6509
56.1107
56.0454
55.1148
53.3105
46.0232
26.5952
6a
Cl
[ppm]

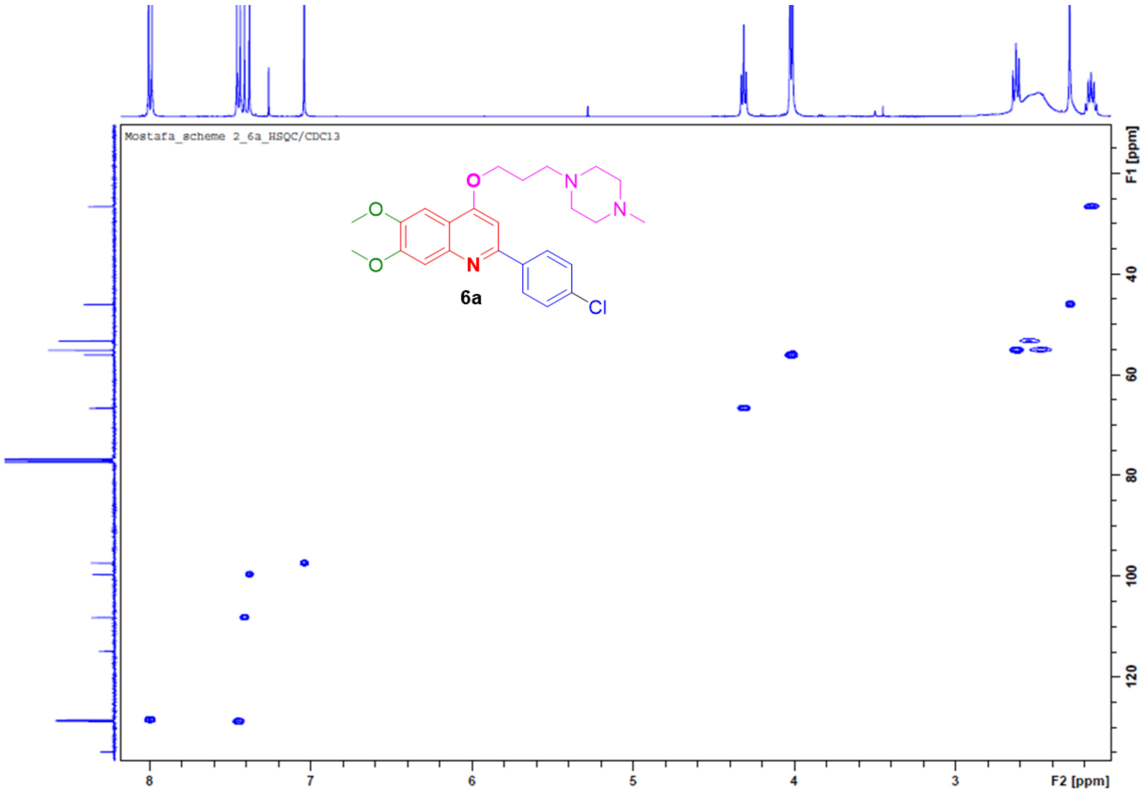
Mostafa_scheme 2_6a_HSQC/CDCl3
6a
Cl
F1 [ppm]
F2 [ppm]

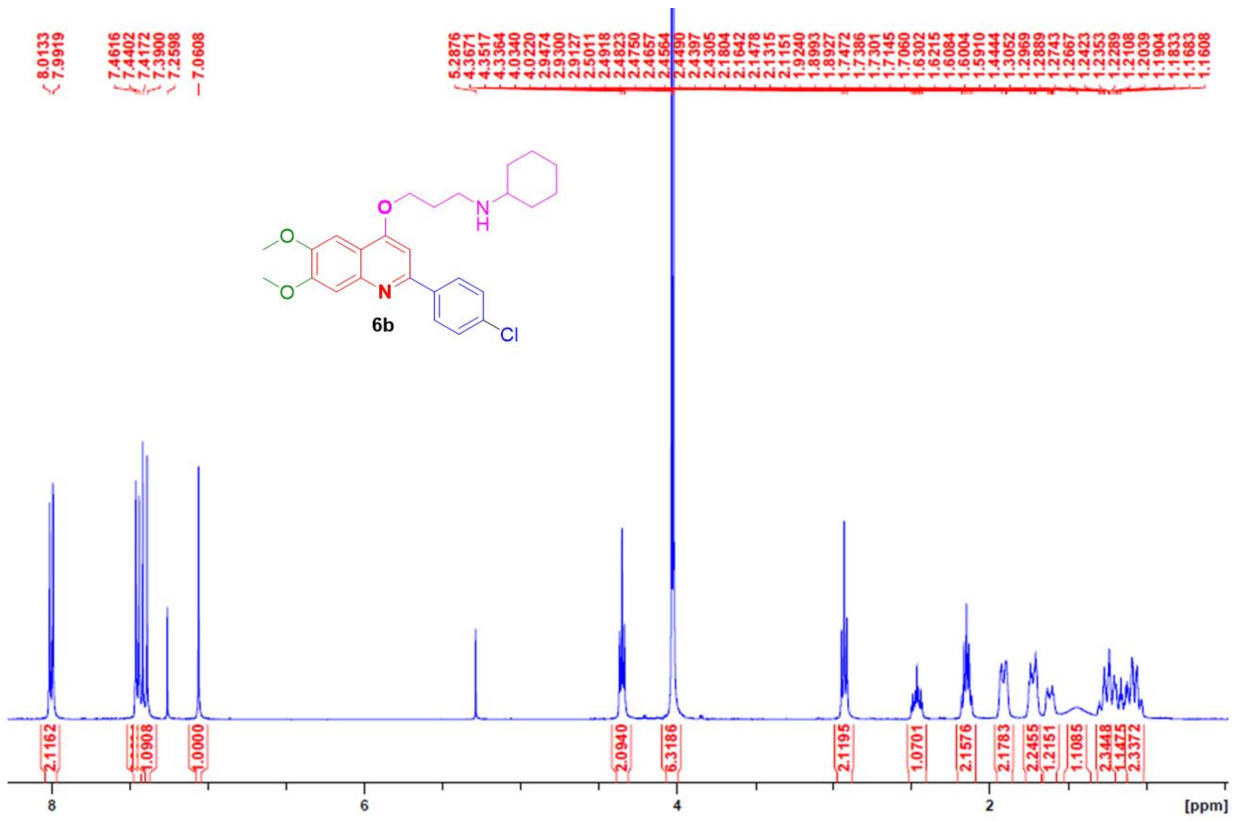

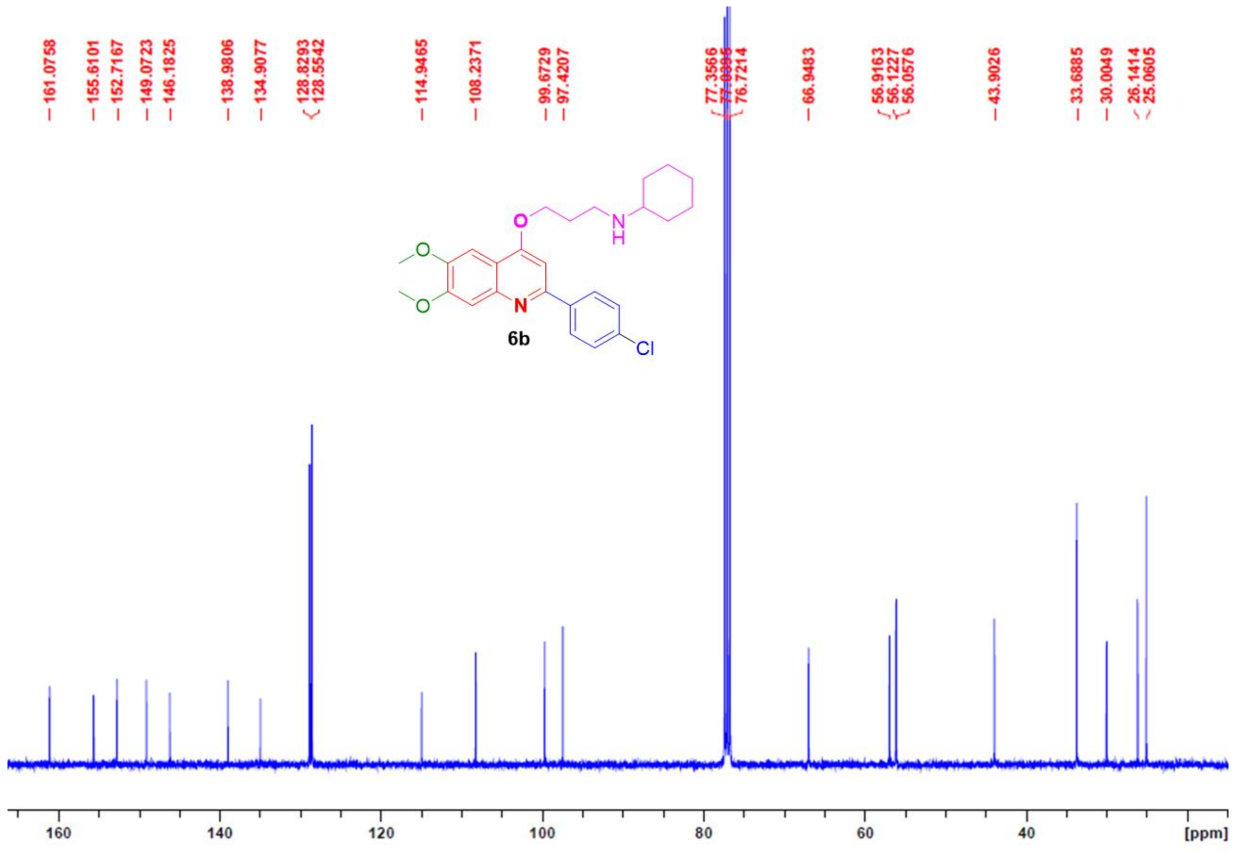

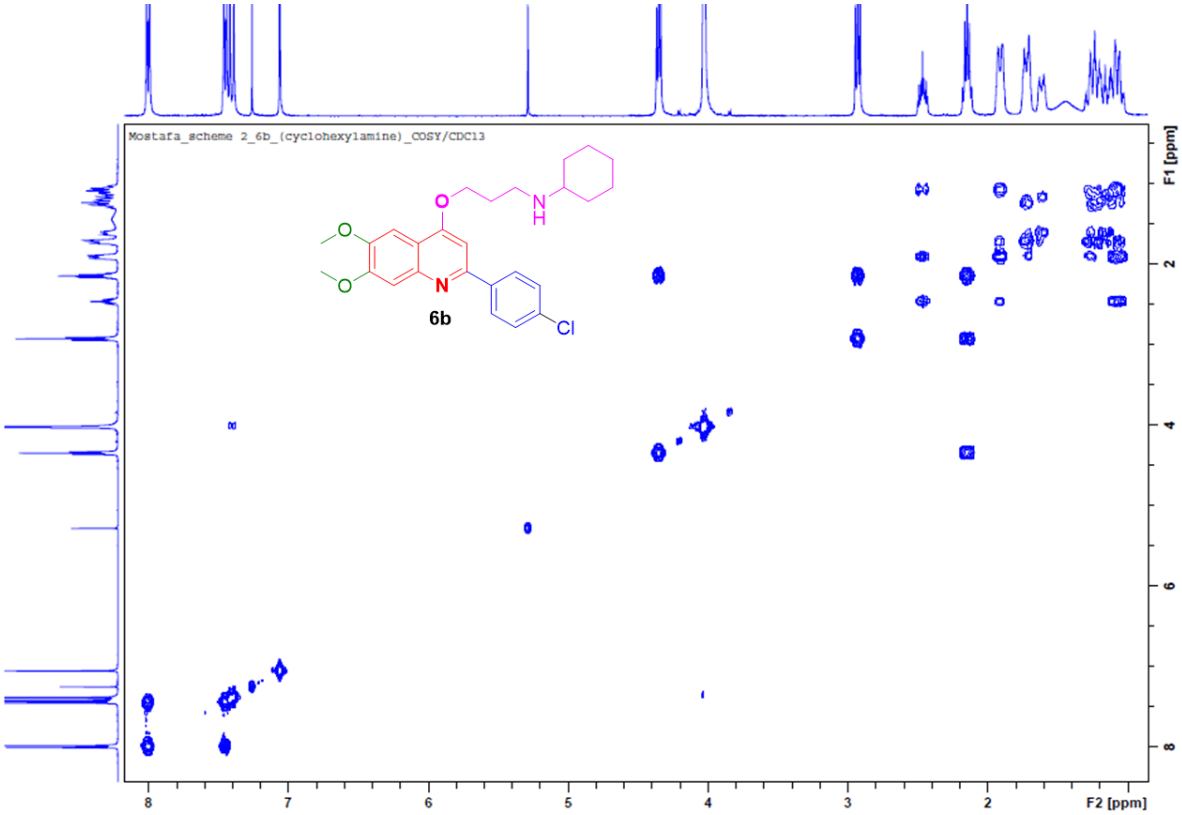
Mostafa_scheme 2_6b_(cyclohexylamine)_COSY/CDCl3
6b
F1 [ppm]
F2 [ppm]

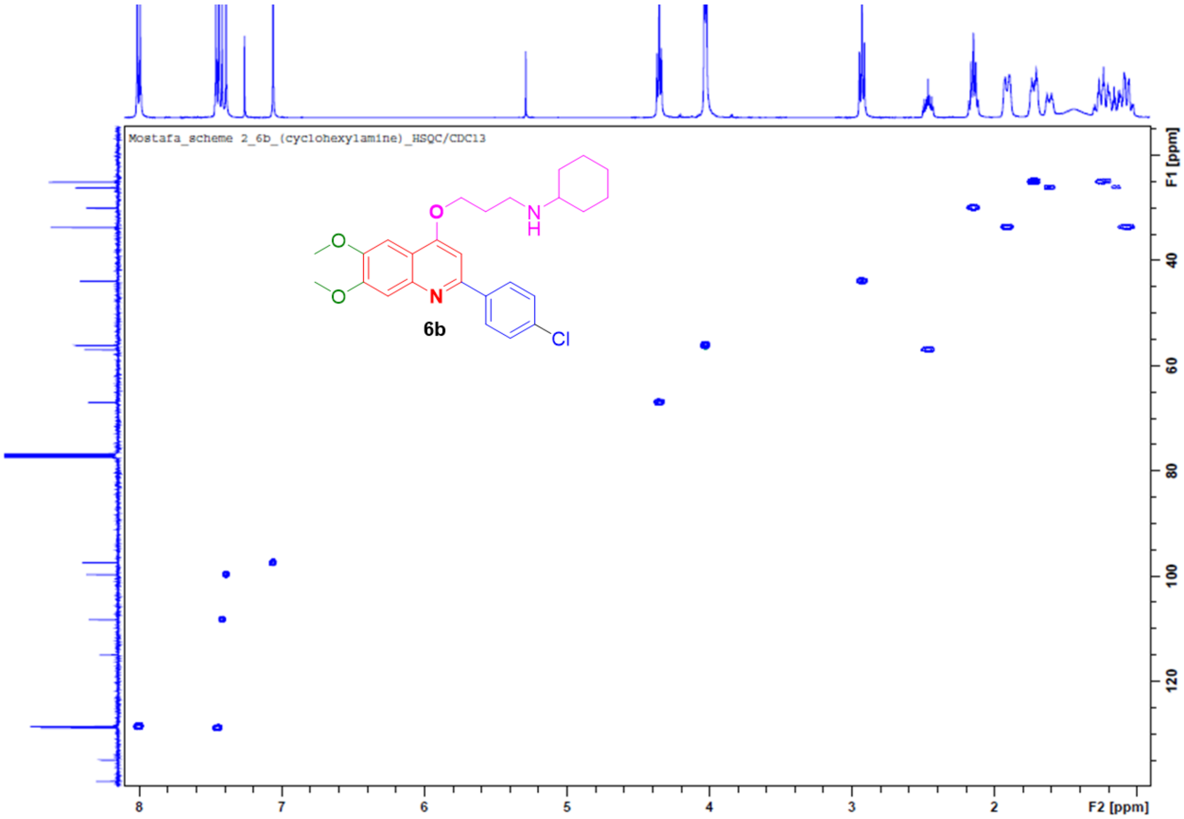
Mostafa_scheme 2_6b_(cyclohexylamine)_HSQC/CDCl3
6b
F1 [ppm]
F2 [ppm]

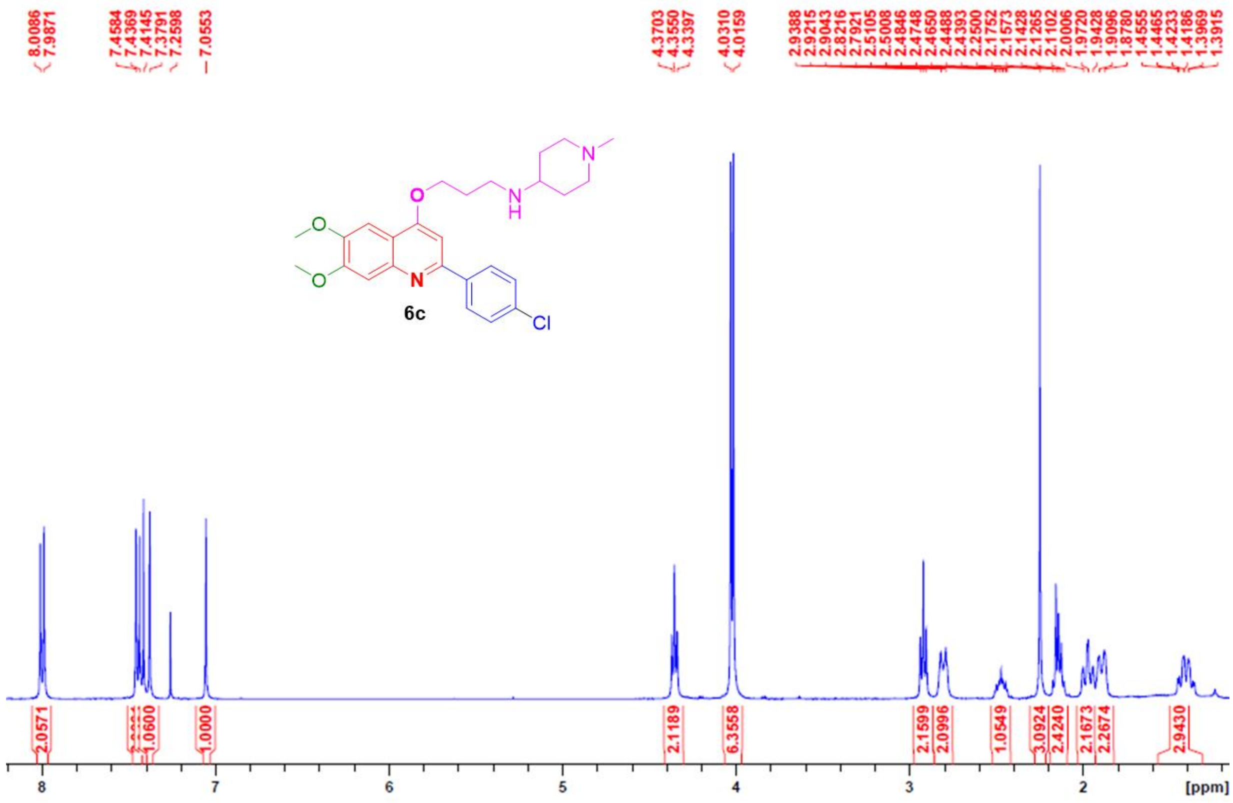
8.0086
7.9871
7.4584
7.4369
7.4145
7.3791
7.2598
7.0553
4.3703
4.3550
4.3397
4.0310
4.0159
2.9388
2.9215
2.9043
2.8216
2.7921
2.5105
2.5008
2.4846
2.4748
2.4650
2.4488
2.4393
2.2500
2.1752
2.1573
2.1428
2.1265
2.1102
2.0006
1.9720
1.9428
1.9096
1.8780
1.4555
1.4465
1.4233
1.4186
1.3969
1.3915
6c
2.0571
1.0600
1.0000
2.1189
6.3558
2.1599
2.0996
1.0549
3.0924
2.4240
2.1673
2.2674
2.9430
[ppm]

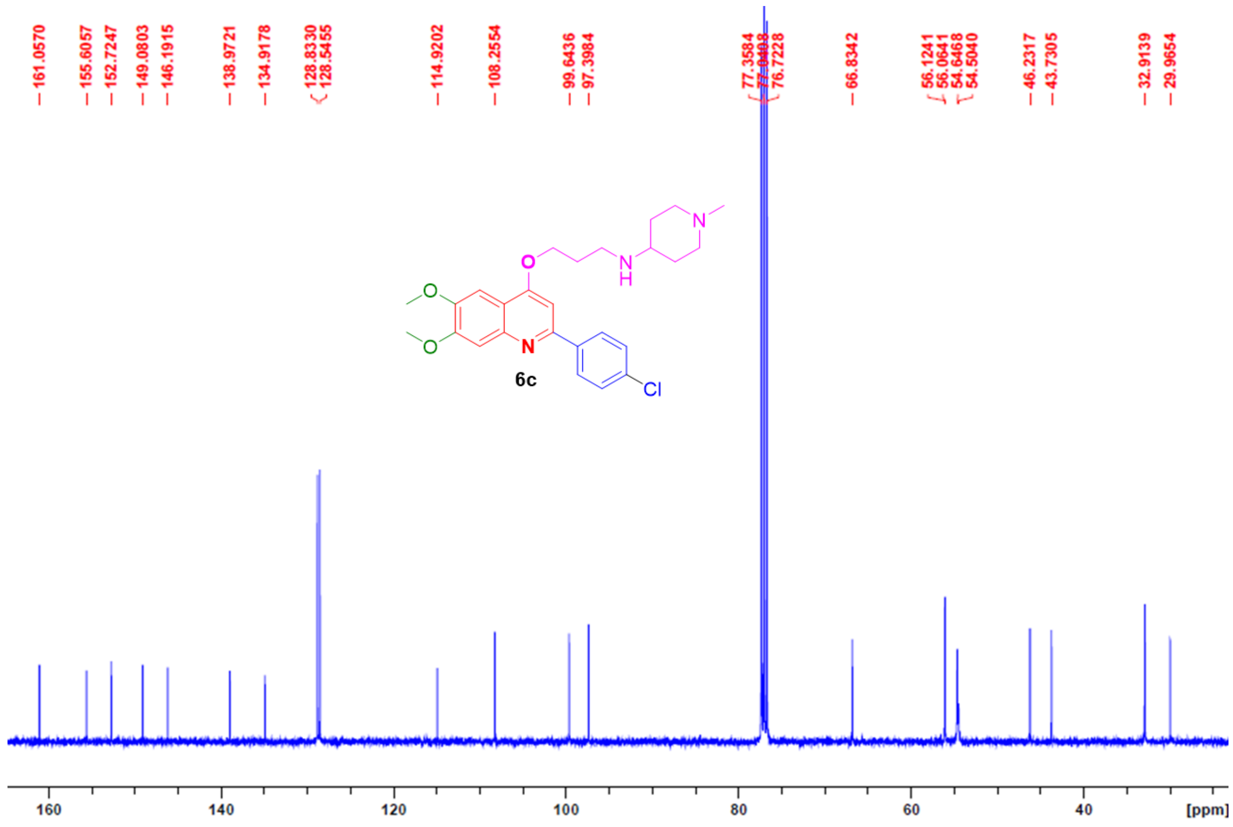
161.0570
155.6057
152.7247
149.0803
146.1915
138.9721
134.9178
128.8330
128.5455
114.9202
108.2554
99.6436
97.3984
77.3584
76.7228
66.8342
56.1241
56.0641
54.6468
54.5040
46.2317
43.7305
32.9139
29.9654
6c
[ppm]

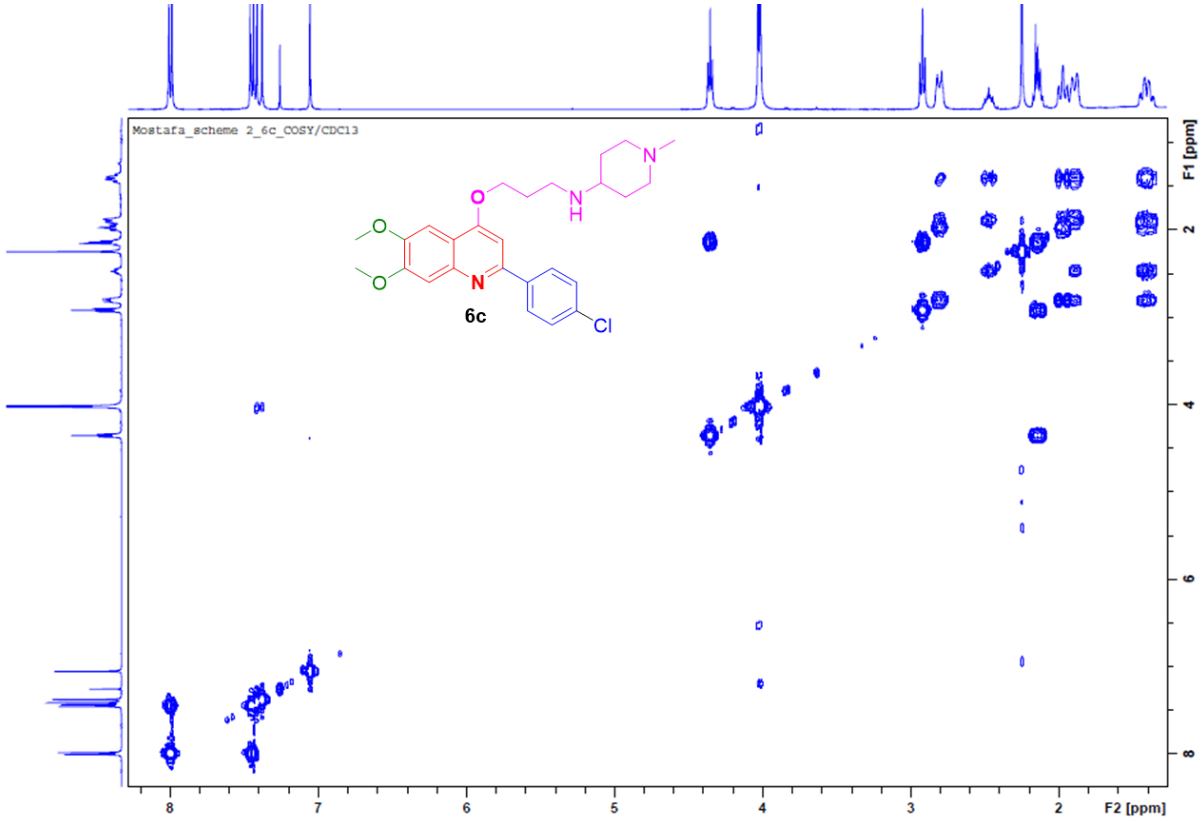
Mostafa_scheme 2_6c_COSY/CDCl3
6c
N
H
O
Cl
F1 [ppm]
F2 [ppm]

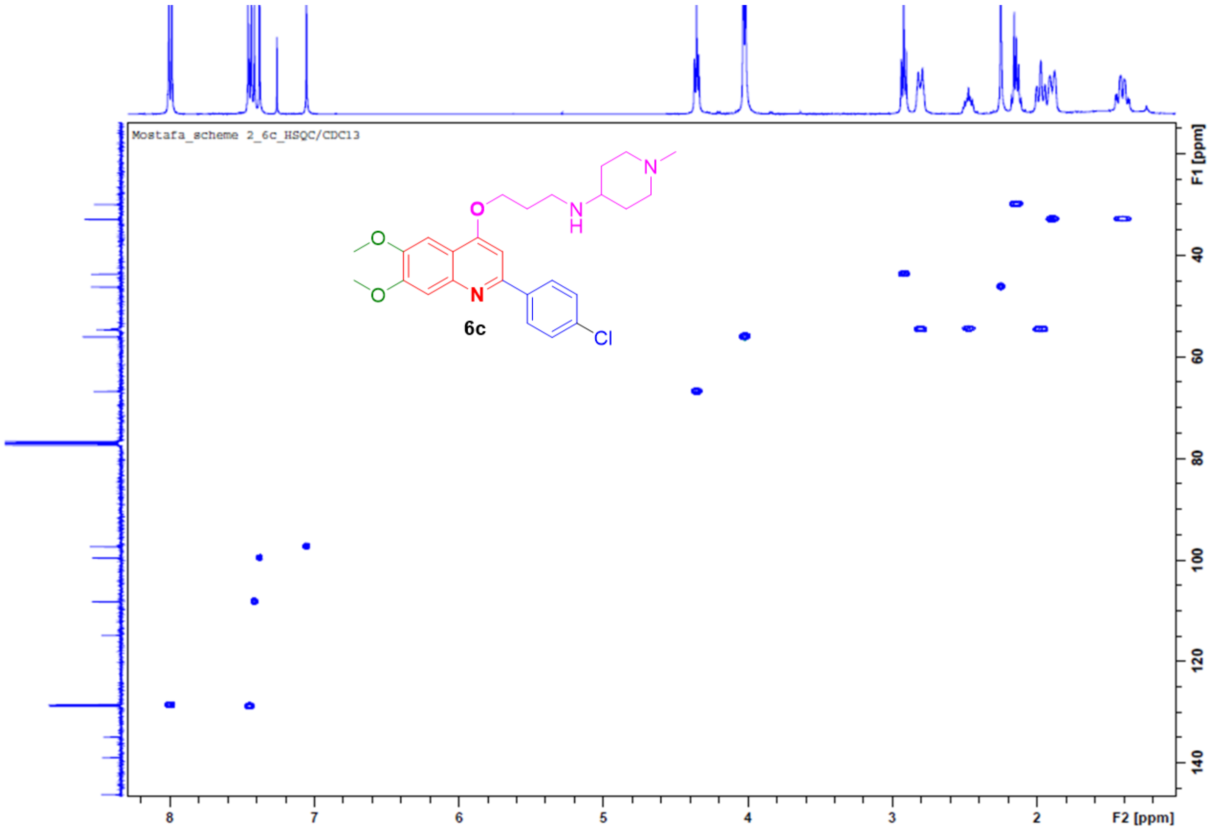
Mostafa_scheme 2_6c_HSQC/CDCl3
6c
N
H
O
Cl
F1 [ppm]
F2 [ppm]

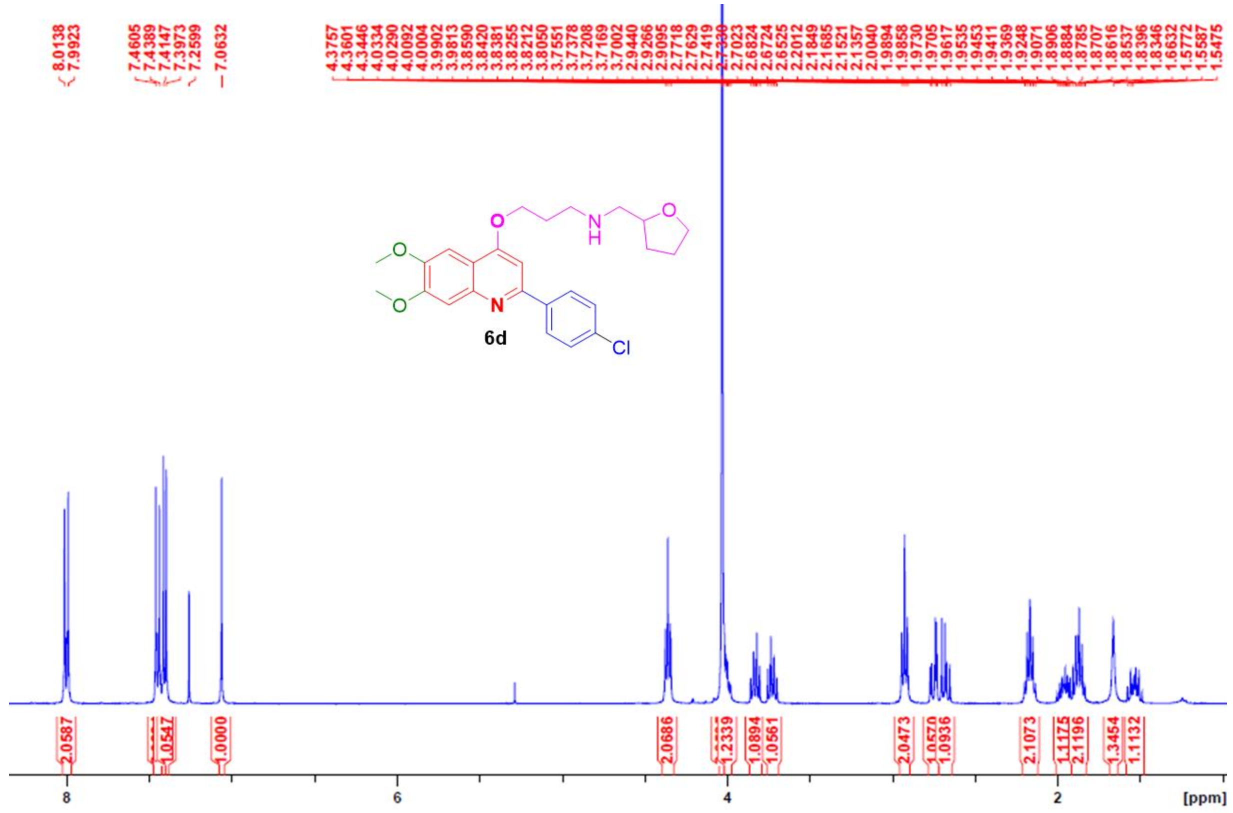

6d
[ppm]

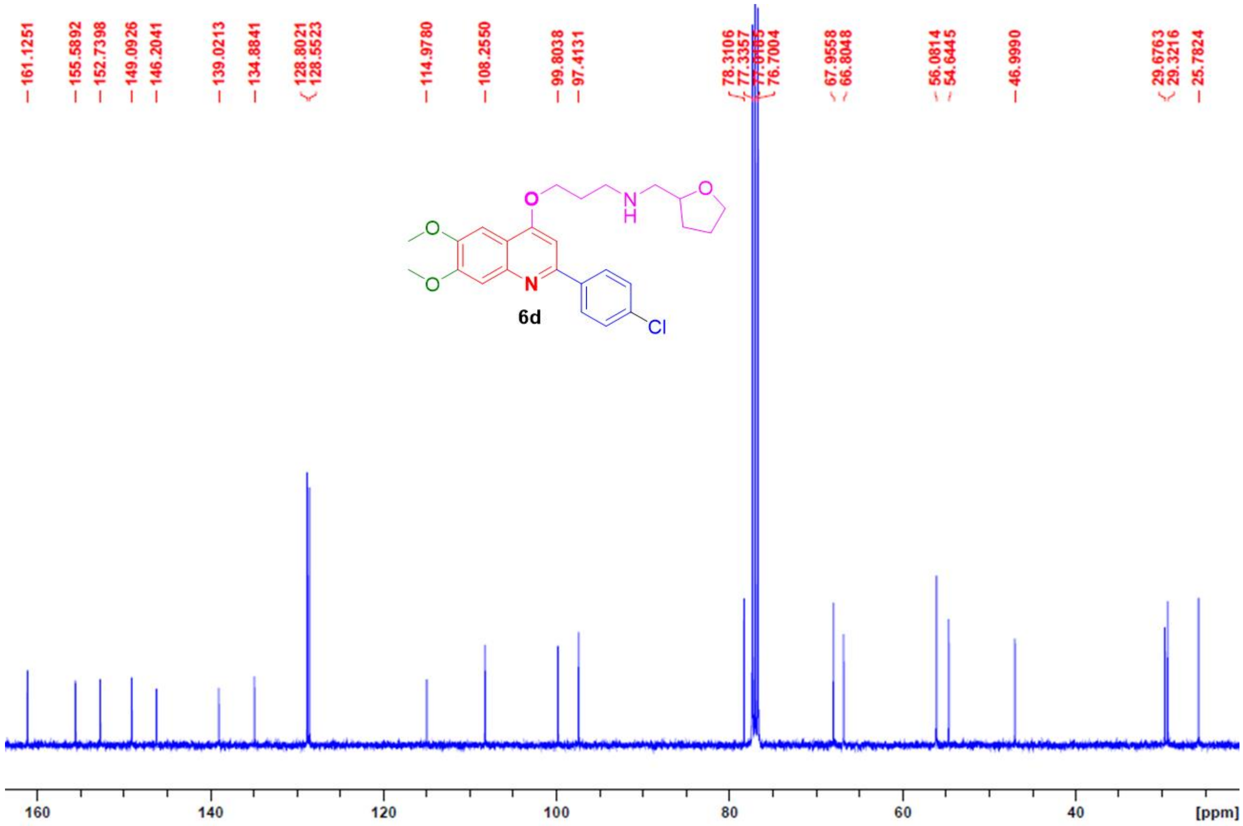

161.1251
155.5892
152.7398
149.0926
146.2041
139.0213
134.8841
128.8021
128.5523
114.9780
108.2550
99.8038
97.4131
78.3106
76.7004
67.9558
66.8048
56.0814
54.6445
46.9990
29.6763
29.3216
25.7824
6d
[ppm]

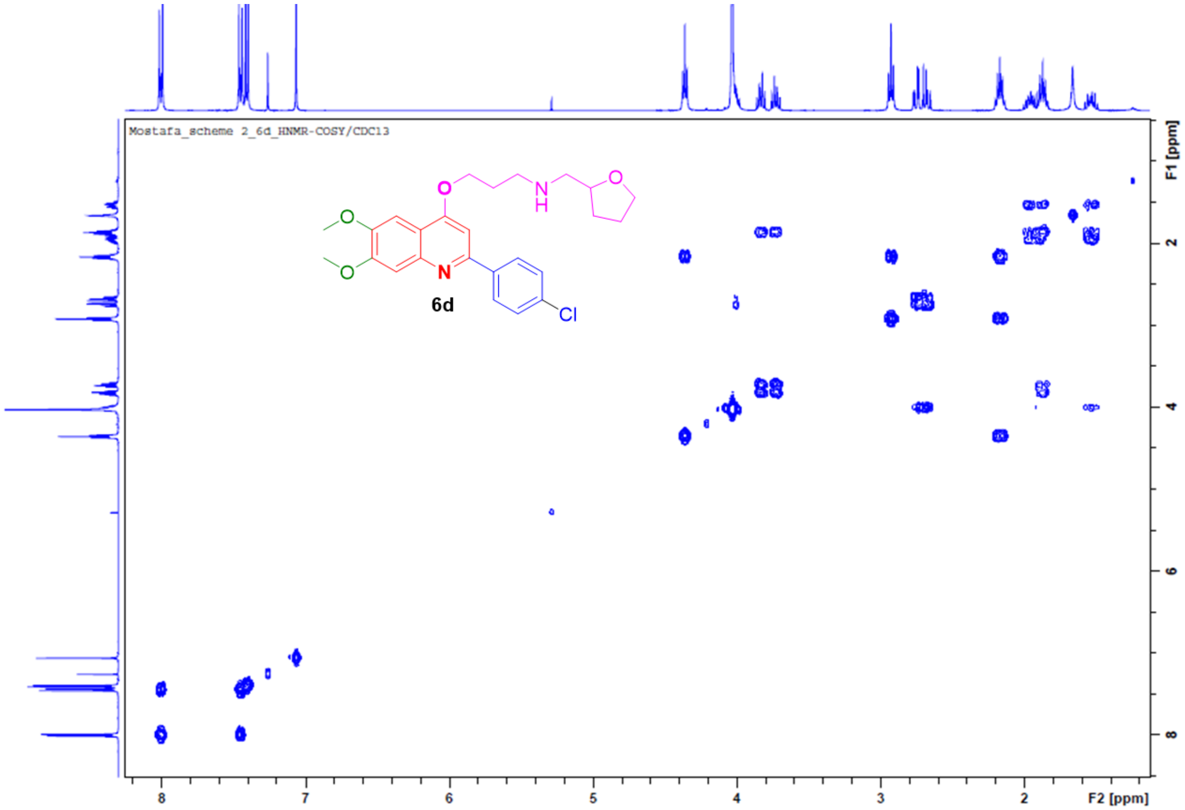
Mostafa_scheme 2_6d_HNMR-COSY/CDCl3
6d
F1 [ppm]
F2 [ppm]

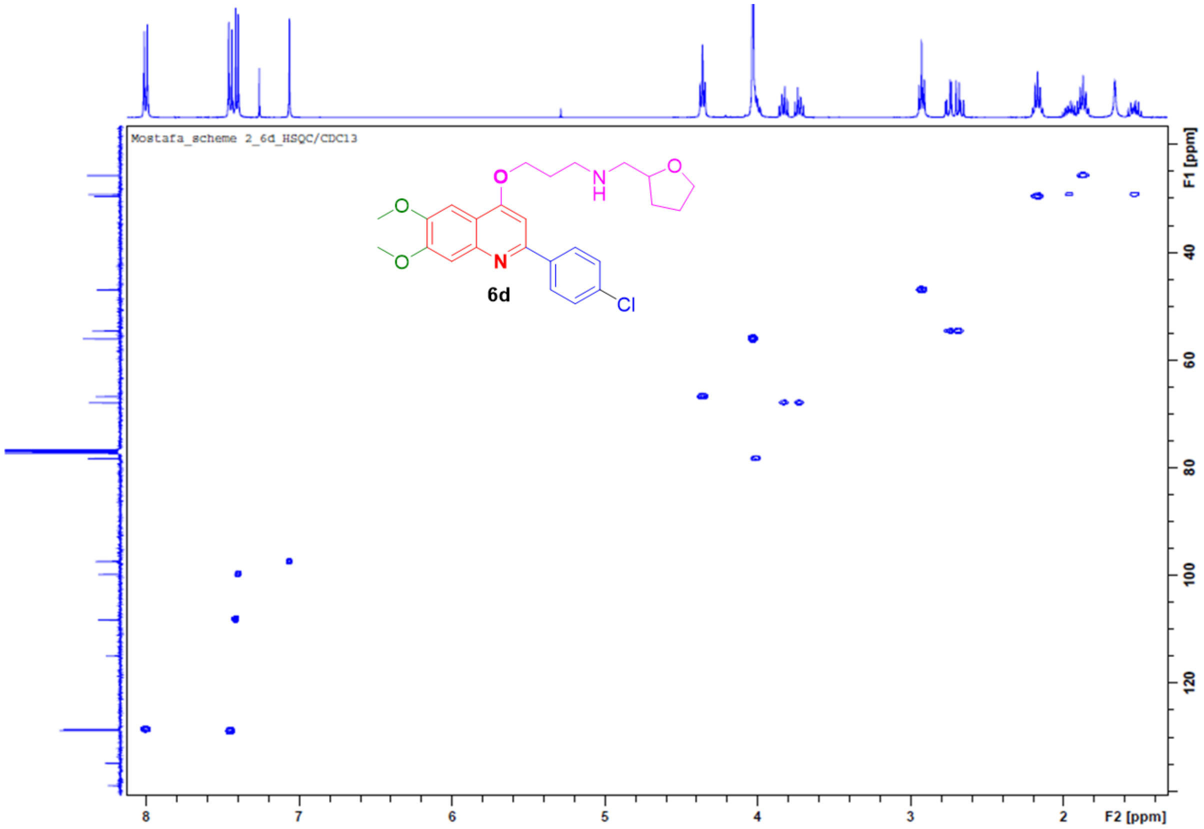
Mostafa_scheme 2_6d_HSQC/CDCl3
6d
F1 [ppm]
F2 [ppm]

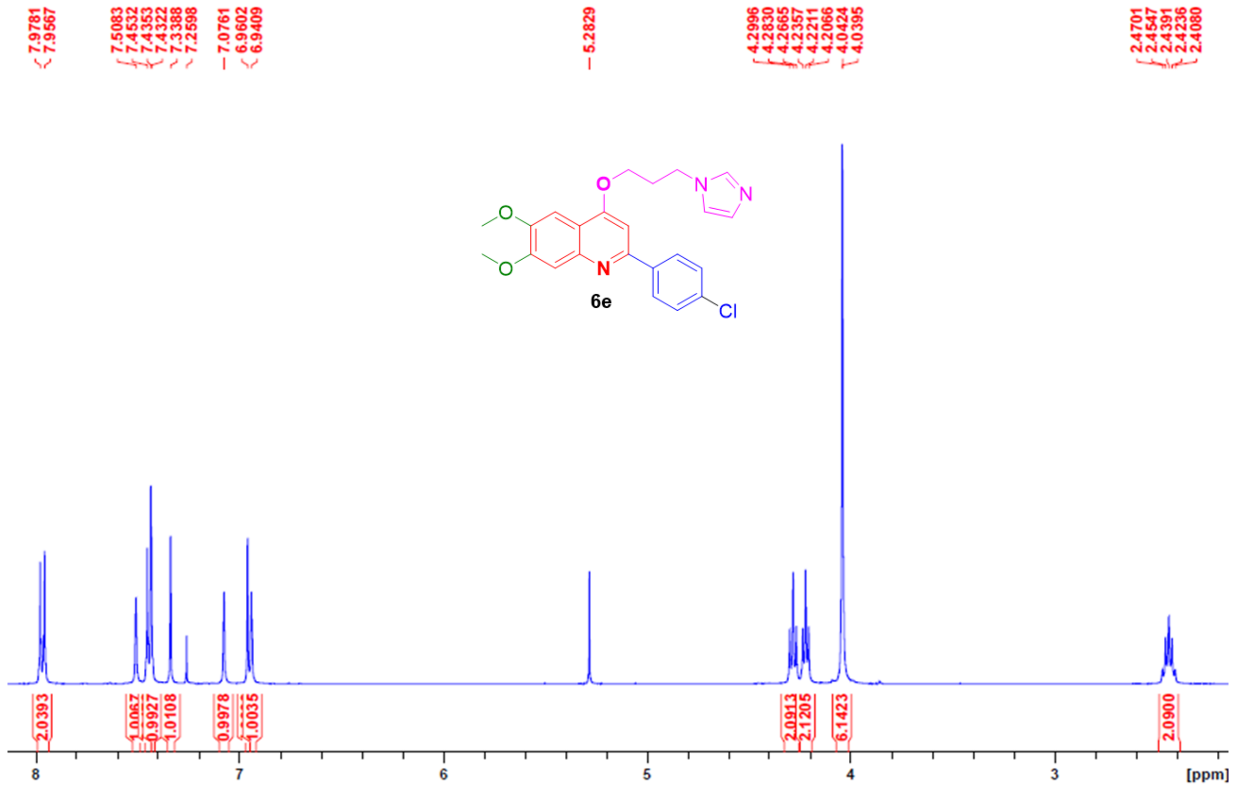

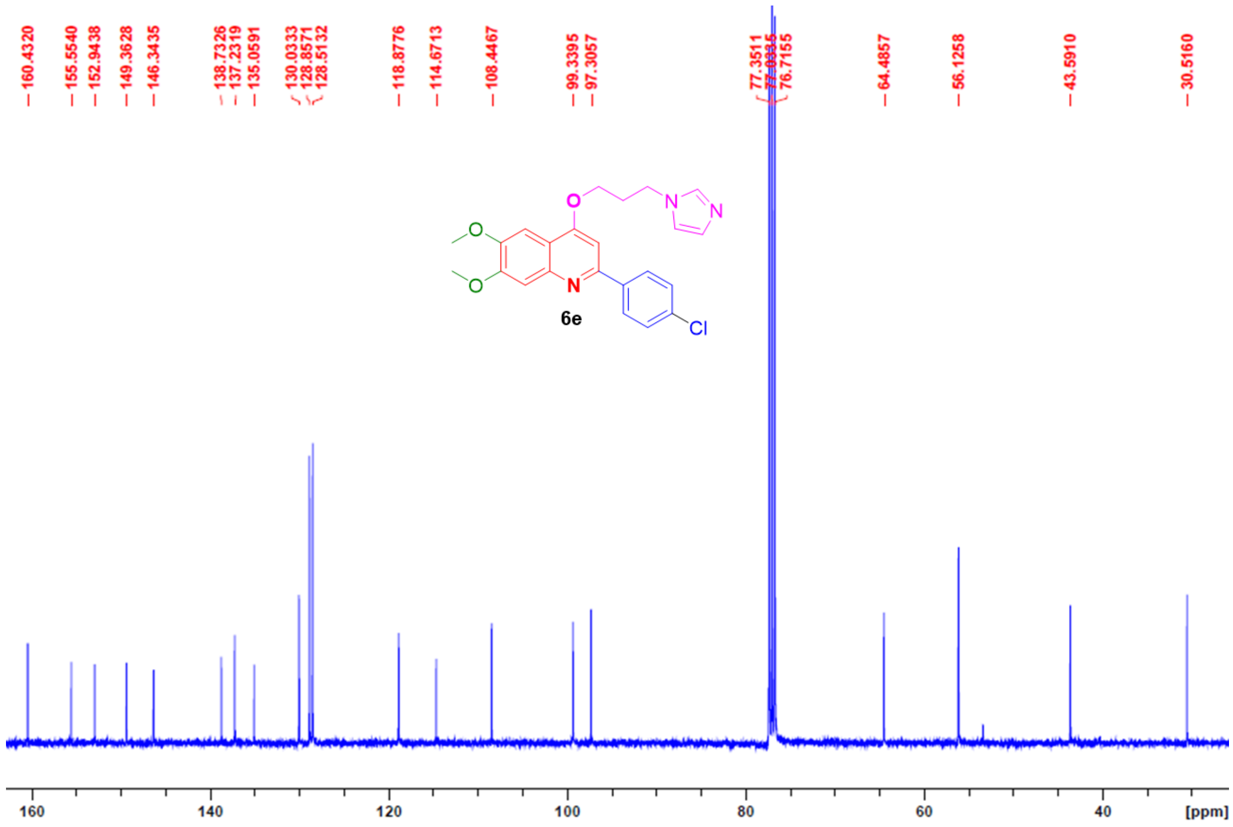

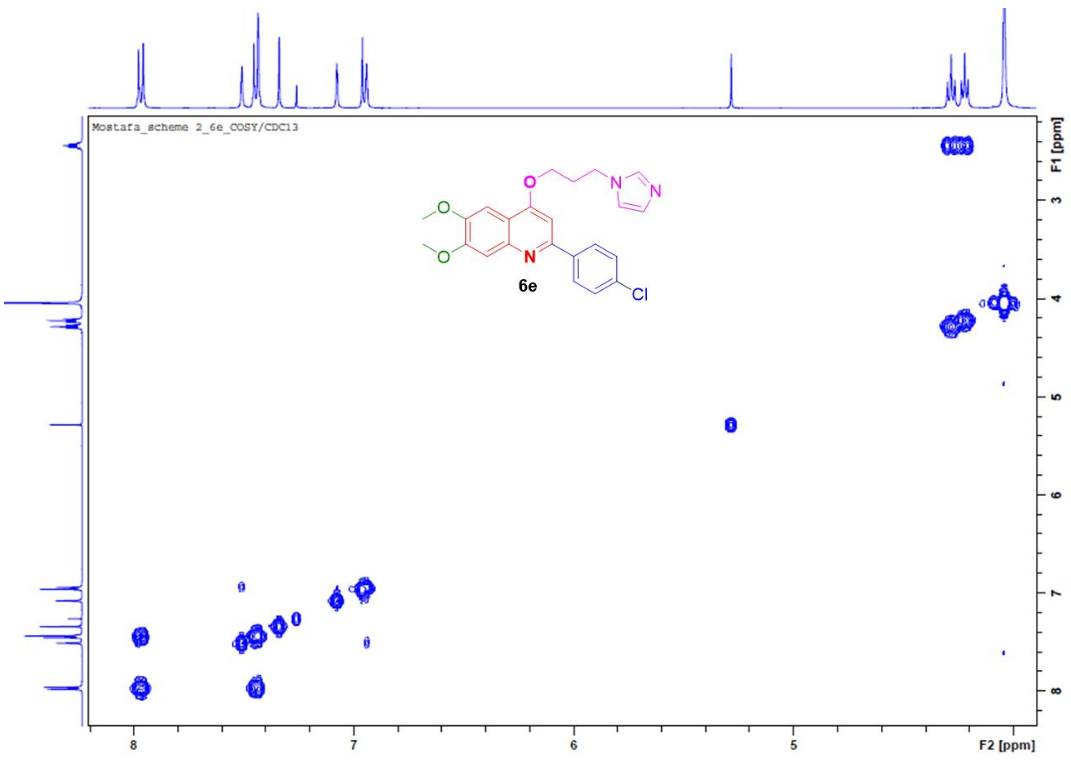
Mostafa_scheme 2_6e_COSY/CDCl3
6e
F1 [ppm]
F2 [ppm]

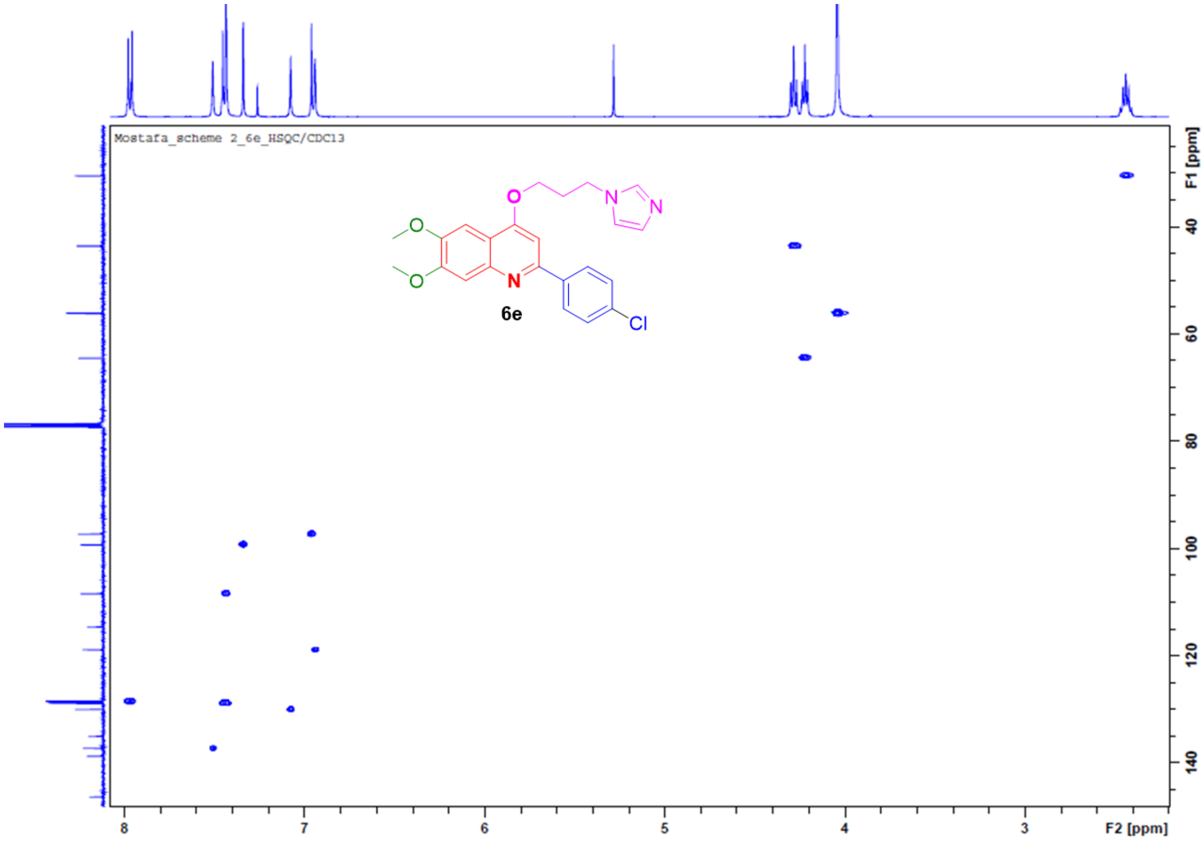
Mostafa_scheme 2_6e_HSQC/CDCl3
6e
F1 [ppm]
F2 [ppm]

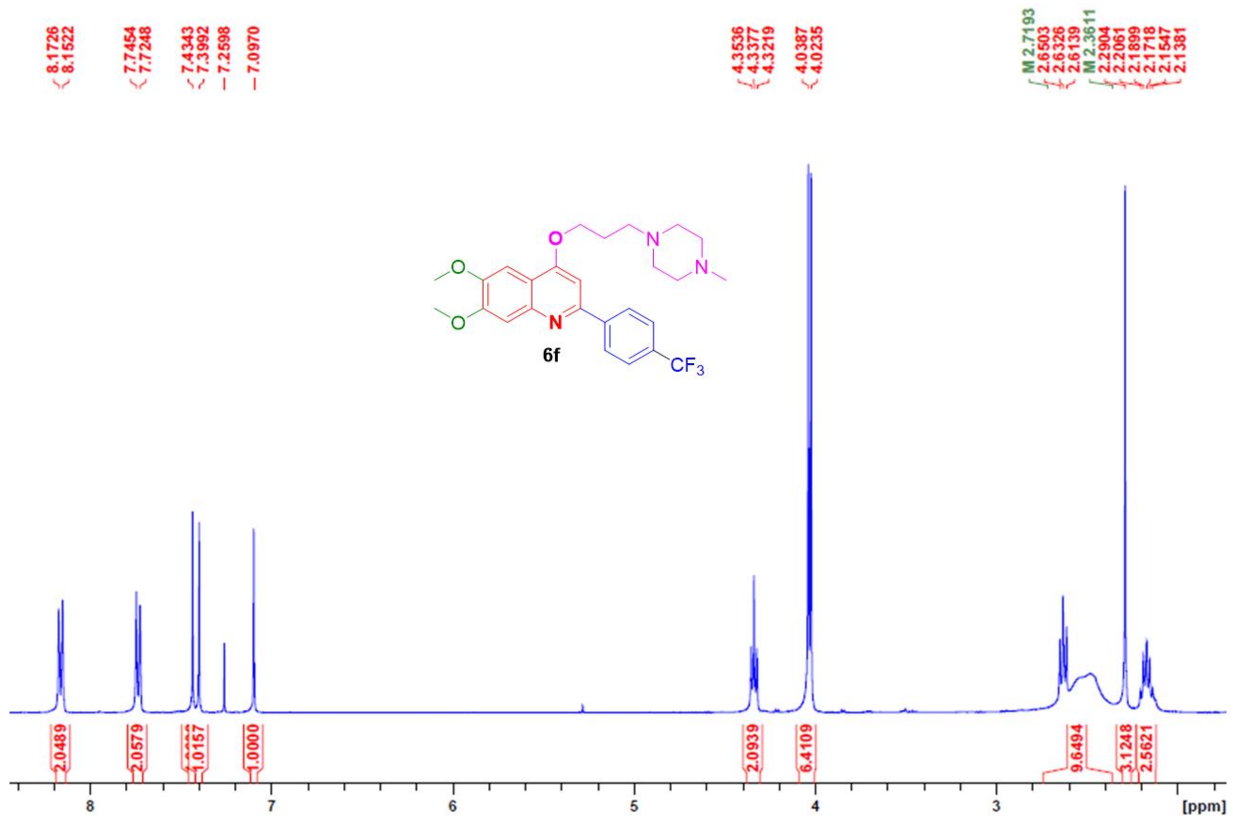
8.1726
8.1522
7.7454
7.7248
7.4343
7.3992
7.2598
7.0970
4.3536
4.3377
4.3219
4.0387
4.0235
M 2.7193
2.6503
2.6326
2.6139
M 2.3611
2.2904
2.2061
2.1899
2.1718
2.1547
2.1381
6f
CF3
2.0489
2.0579
1.0157
1.0000
2.0939
6.4109
9.6494
3.1248
2.5621
[ppm]

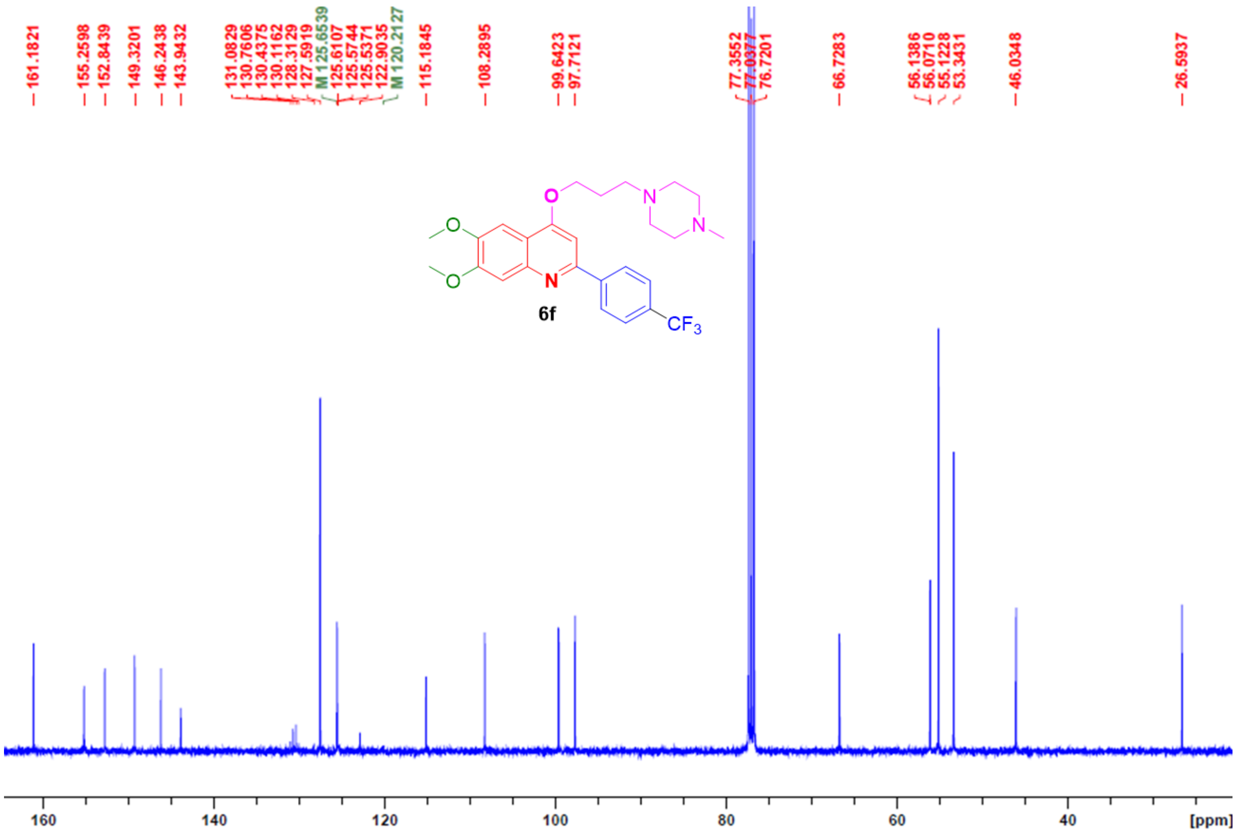
161.1821
155.2598
152.8439
149.3201
146.2438
143.9432
131.0829
130.7606
130.4375
130.1162
128.3129
127.5919
M 125.6539
125.6107
125.5744
125.5371
122.9035
M 120.2127
115.1845
108.2895
99.6423
97.7121
77.3552
77.0377
76.7201
66.7283
56.1386
56.0710
55.1228
53.3431
46.0348
26.5937
6f
CF3
[ppm]

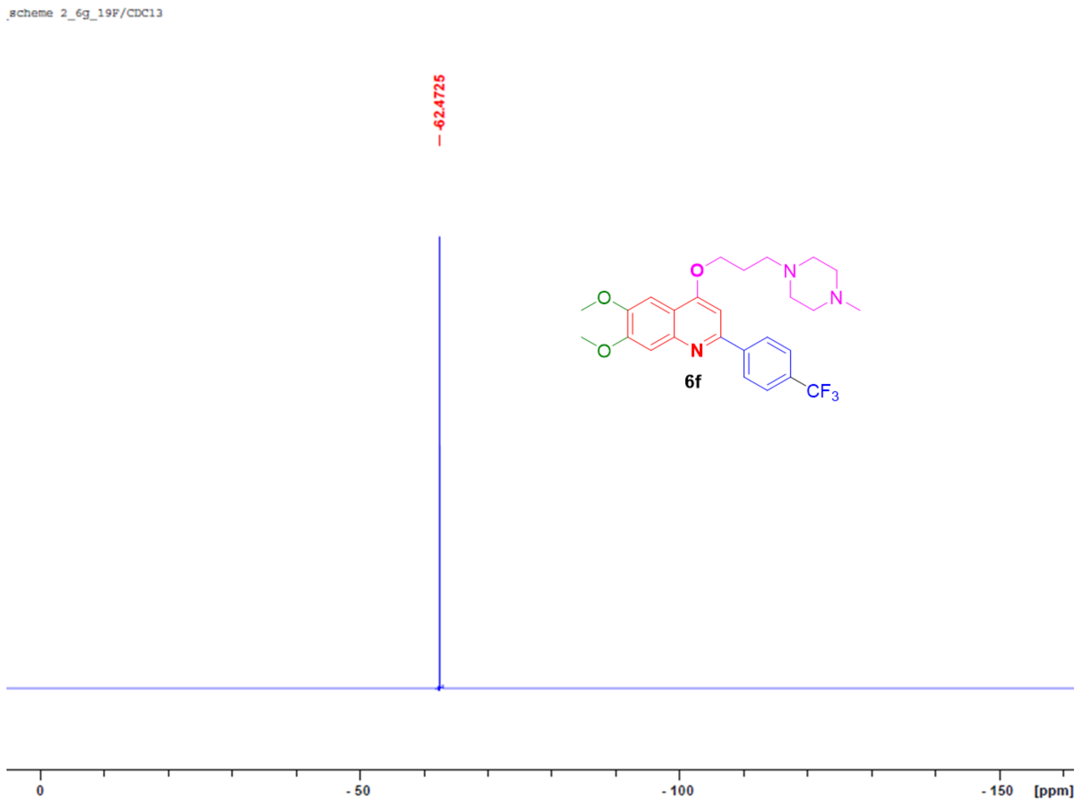
scheme 2_6g_19F/CDCl3
-62.4725
6f
CF3
0
-50
-100
-150
[ppm]

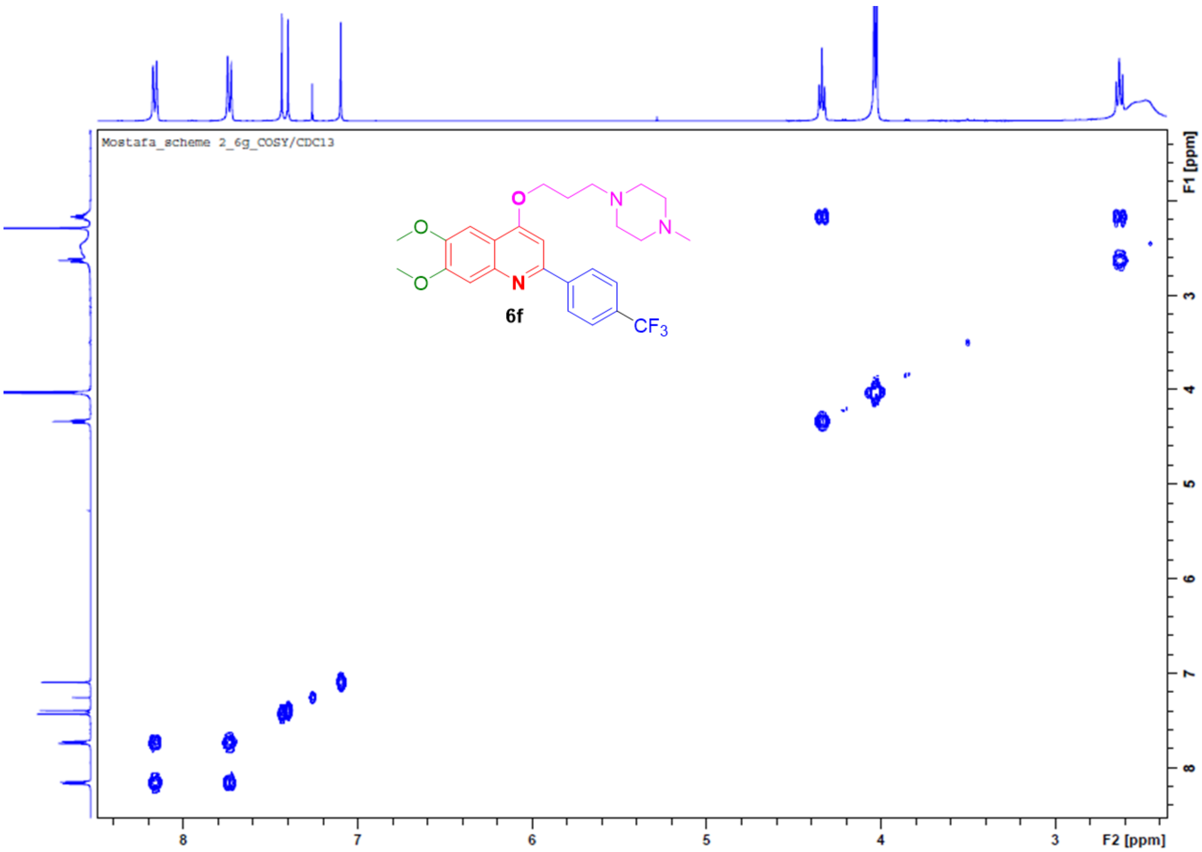
Mostafa_scheme 2_6g_COSY/CDCl3
6f
CF3
F1 [ppm]
F2 [ppm]

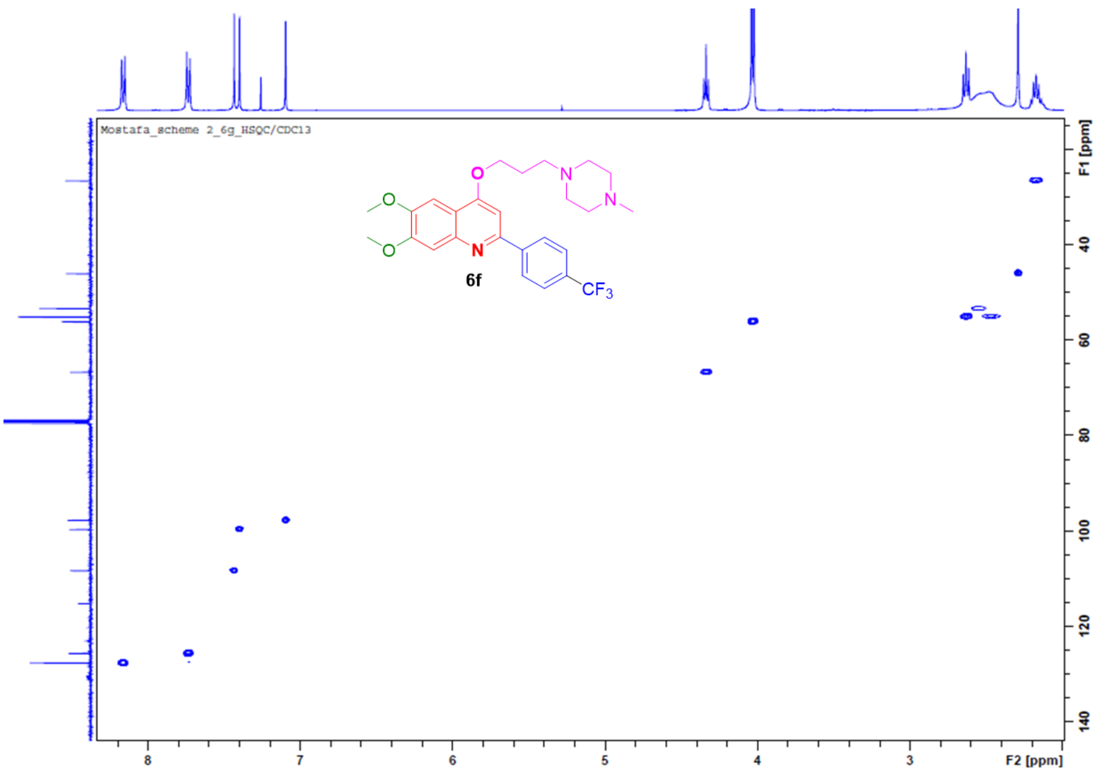
Mostafa_scheme 2_6g_HSQC/CDCl3
6f
F1 [ppm]
F2 [ppm]

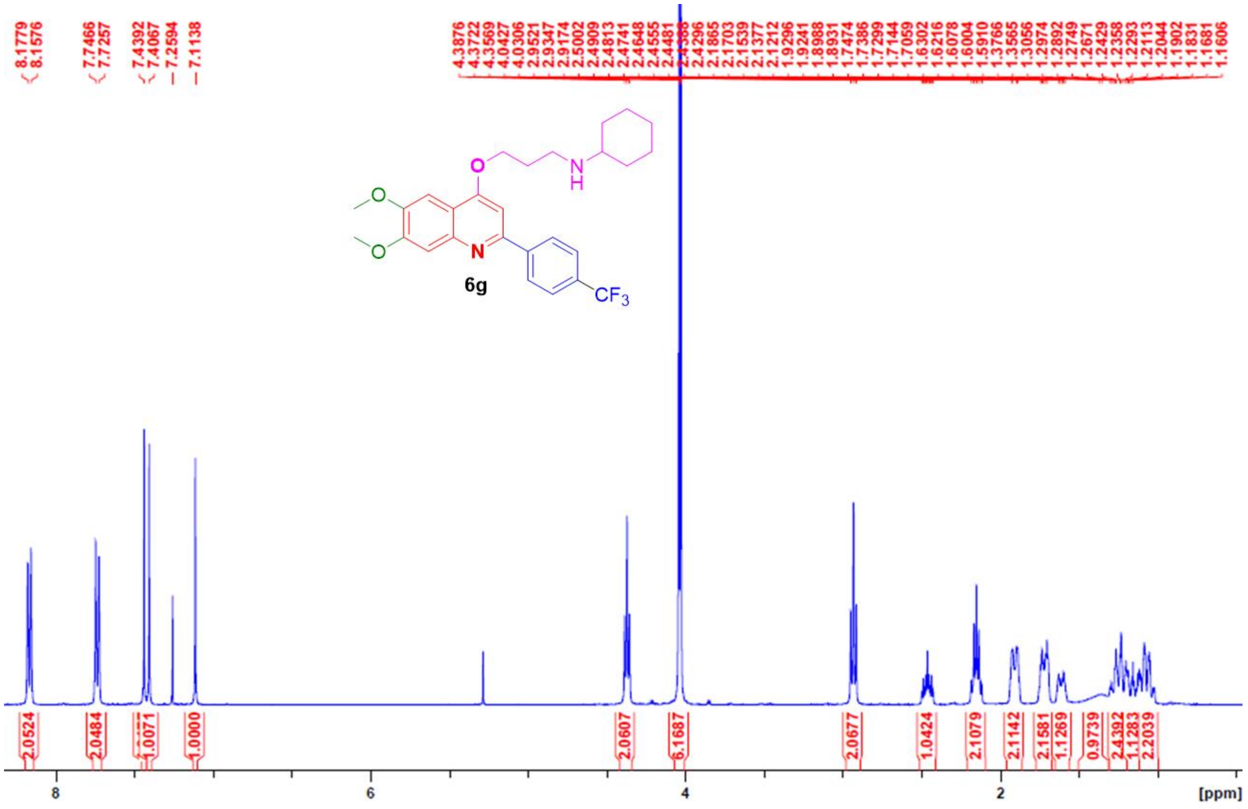
6g
[ppm]

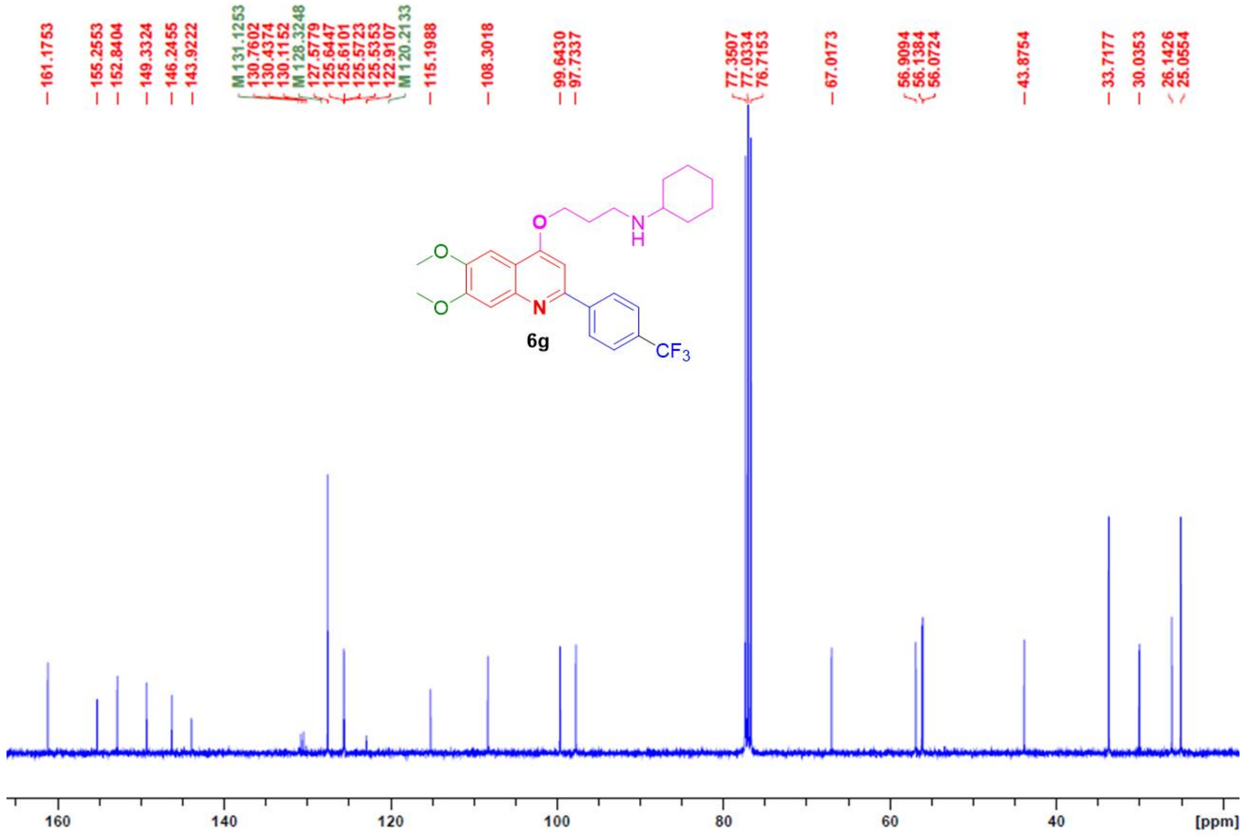

_scheme 2_6h_19F/CDCl3

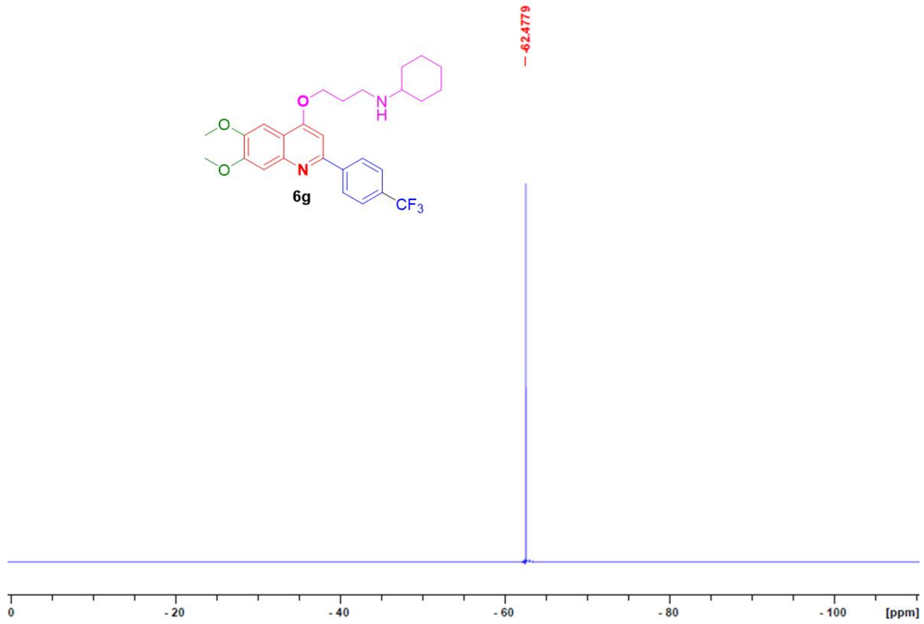

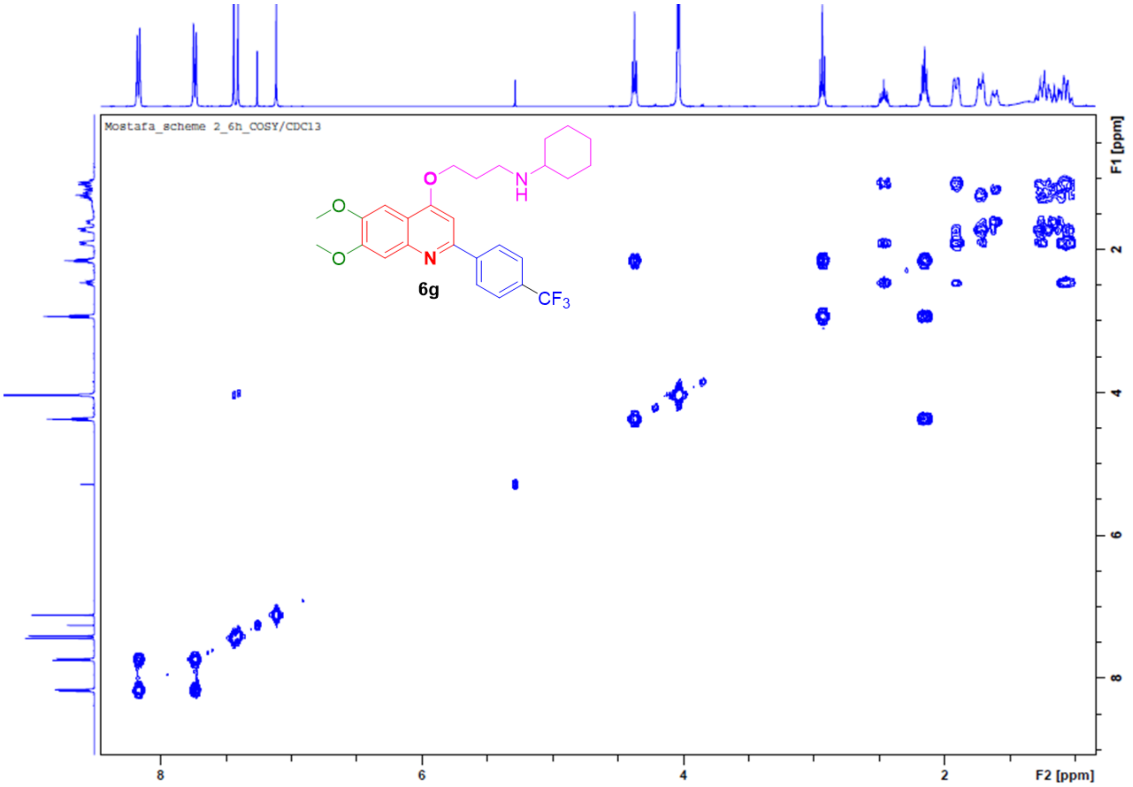
Mostafa_scheme 2_6h_COSY/CDCl3
6g
CF3
F1 [ppm]
F2 [ppm]

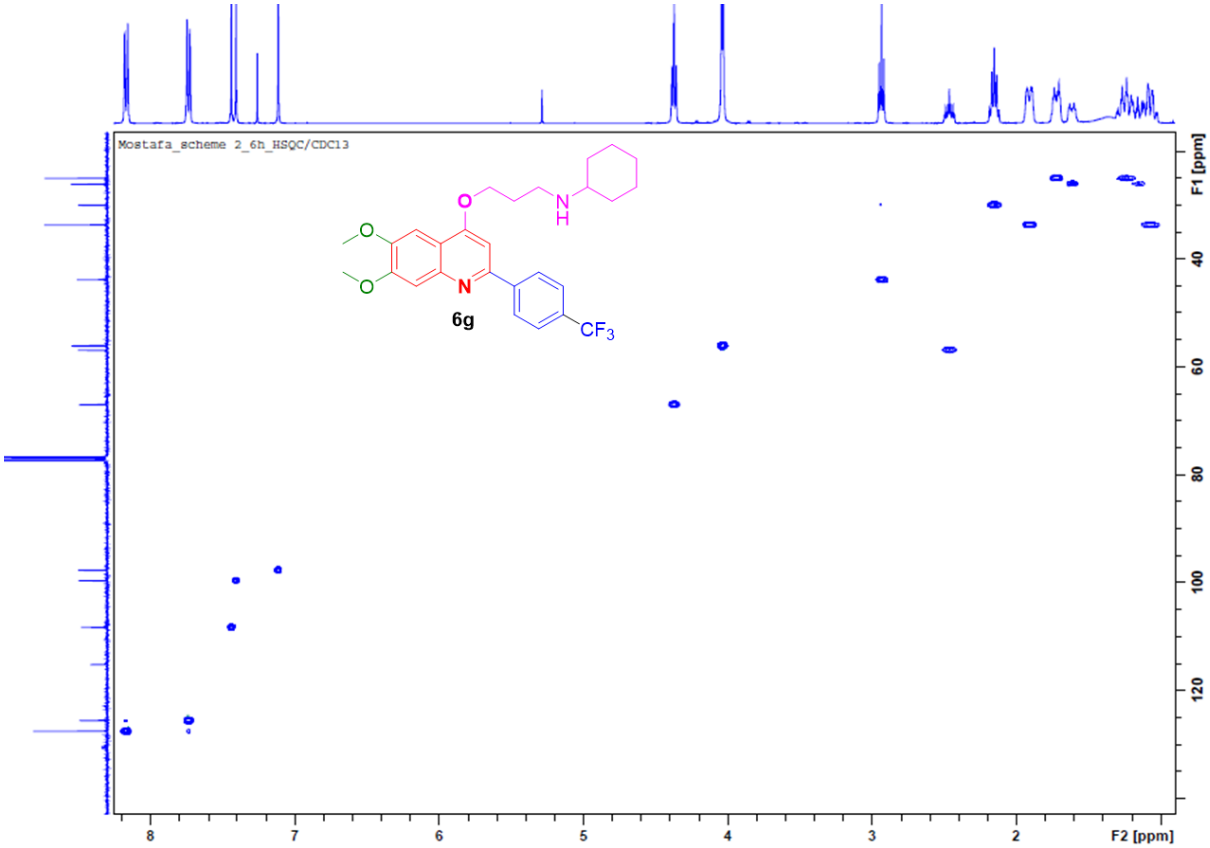
Mostafa_scheme 2_6h_HSQC/CDCl3
6g
CF3
F1 [ppm]
F2 [ppm]

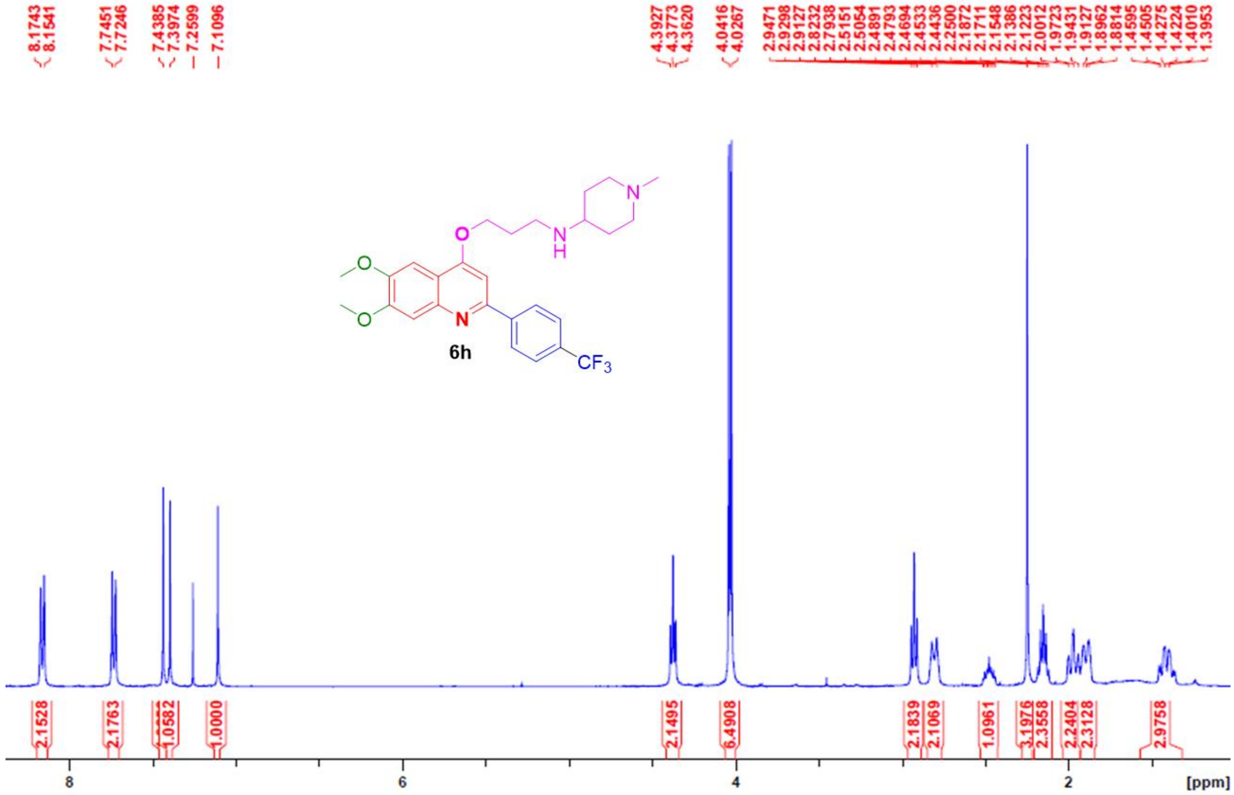
8.1743
8.1541
7.7451
7.7246
7.4385
7.3974
7.2599
7.1096
4.3927
4.3773
4.3620
4.0416
4.0267
2.9471
2.9298
2.9127
2.8232
2.7938
2.5151
2.5054
2.4891
2.4793
2.4694
2.4533
2.4436
2.2500
2.1872
2.1711
2.1548
2.1386
2.1223
2.0012
1.9723
1.9431
1.9127
1.8962
1.8814
1.4595
1.4505
1.4275
1.4224
1.4010
1.3953
6h
2.1528
2.1763
1.0582
1.0000
2.1495
6.4908
2.1839
2.1069
1.0961
3.1976
2.3558
2.2404
2.3128
2.9758
8
6
4
2
[ppm]

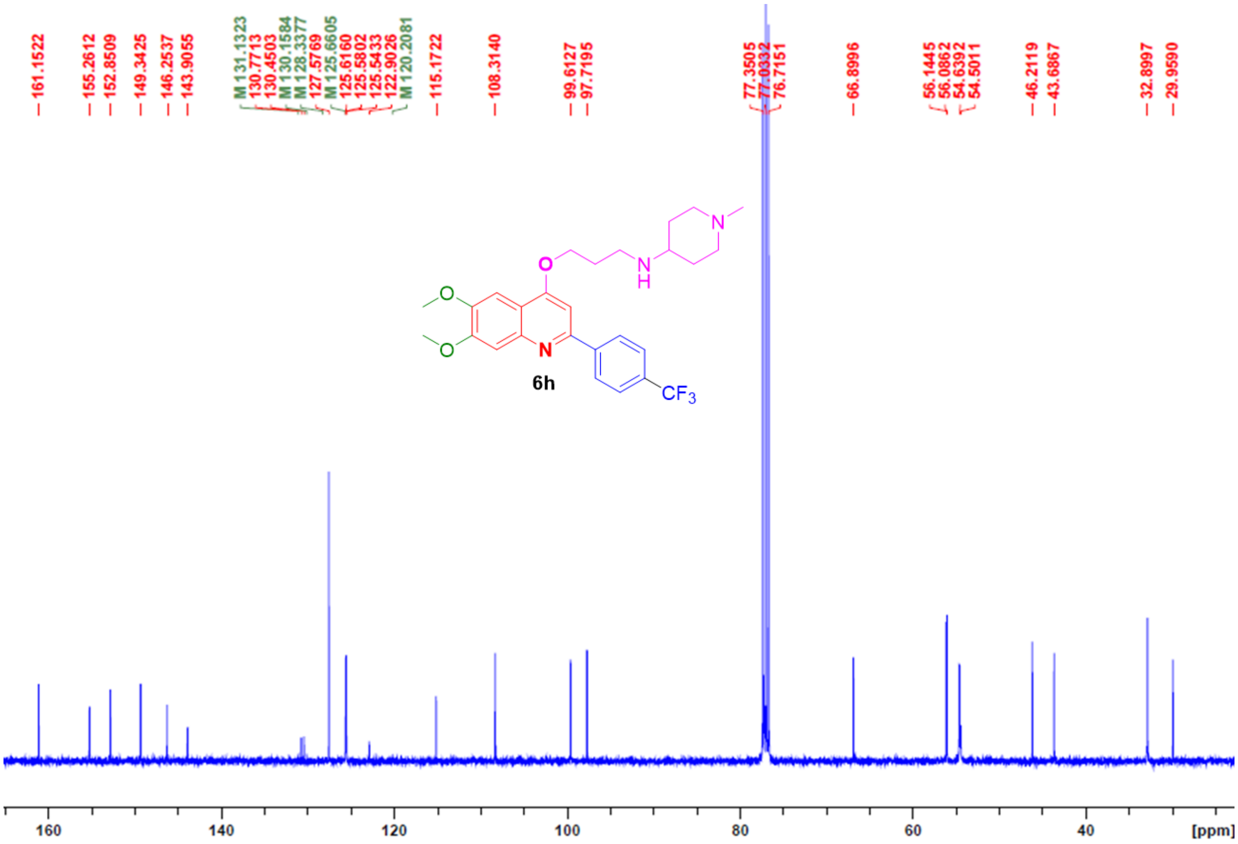
161.1522
155.2612
152.8509
149.3425
146.2537
143.9055
M 131.1323
130.7713
130.4503
M 130.1584
M 128.3377
127.5769
M 125.6605
125.6160
125.5802
125.5433
122.9026
M 120.2081
115.1722
108.3140
99.6127
97.7195
77.3505
77.0332
76.7151
66.8996
56.1445
56.0862
54.6392
54.5011
46.2119
43.6867
32.8997
29.9590
6h
160
140
120
100
80
60
40
[ppm]

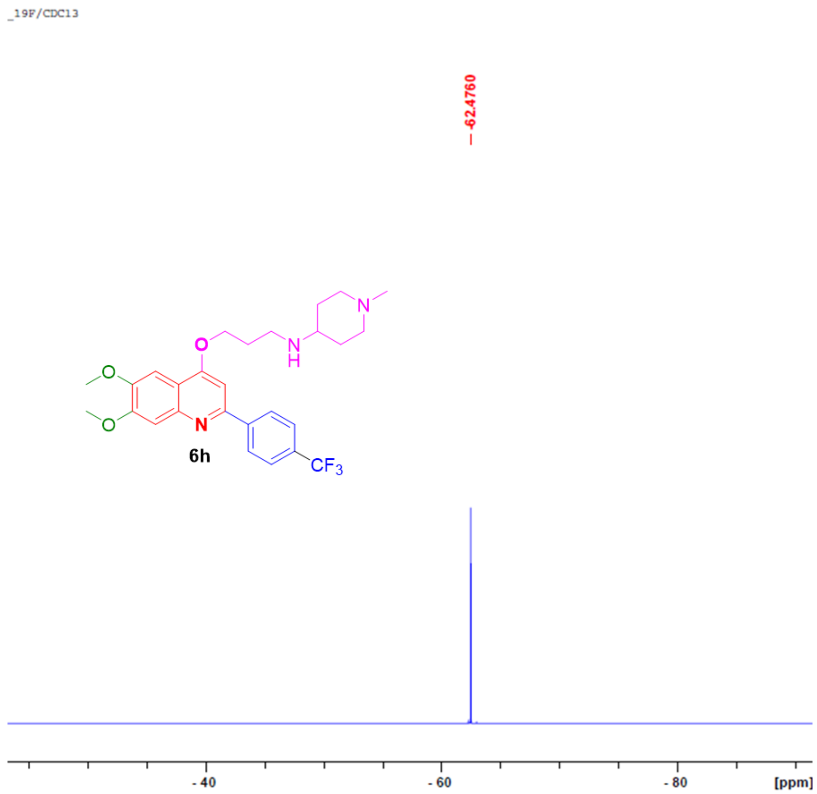
_19F/CDCl3
-62.4760
O
N
H
N
O
O
N
6h
CF3
-40
-60
-80
[ppm]

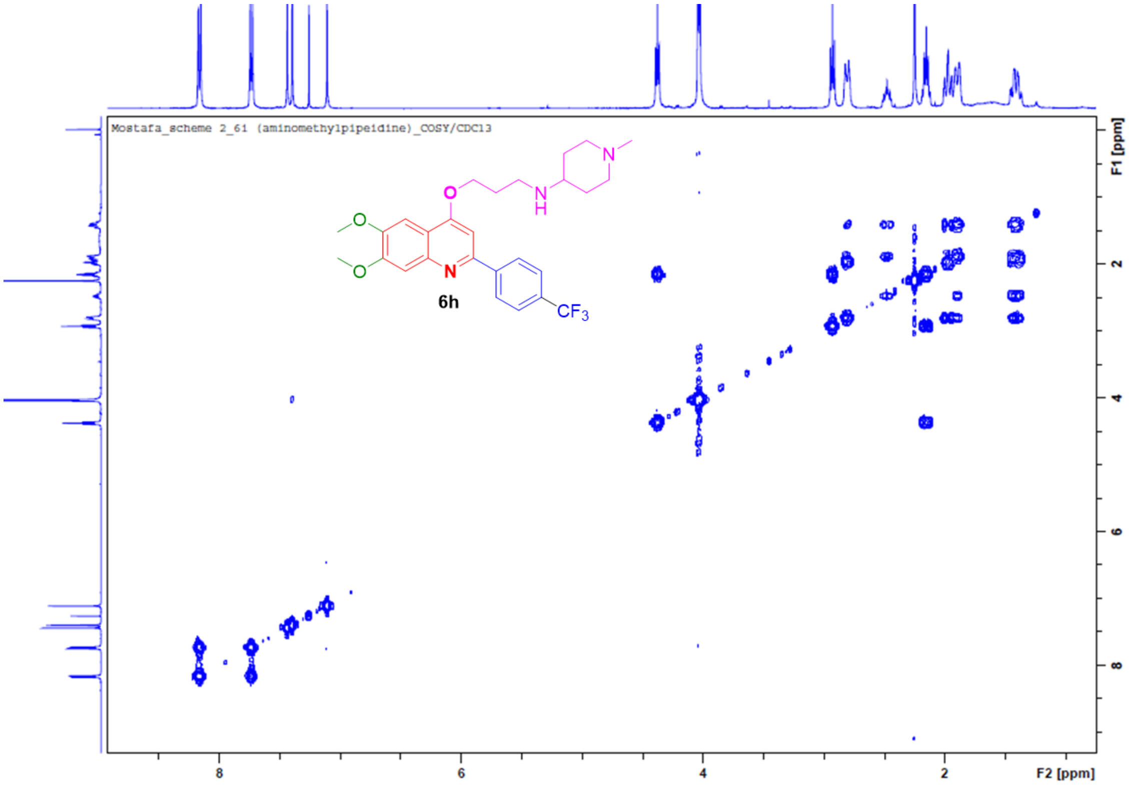
Mostafa_scheme 2_61 (aminomethylpipeidine)_COSY/CDCl3
O
N
H
N
O
O
N
6h
CF3
F1 [ppm]
2
4
6
8
8
6
4
2
F2 [ppm]

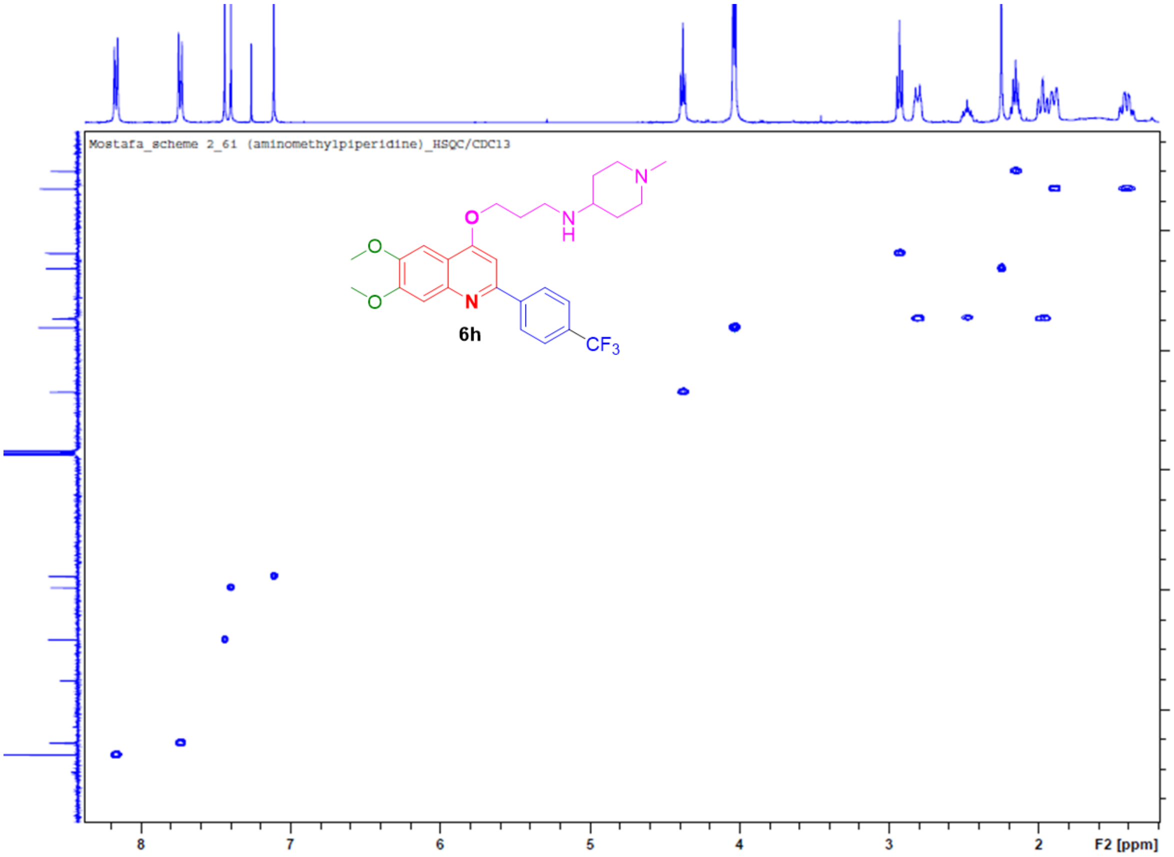

Mostafa_scheme 2_61 (aminomethylpiperidine)_HSQC/CDCl3
6h
F1 [ppm]
F2 [ppm]

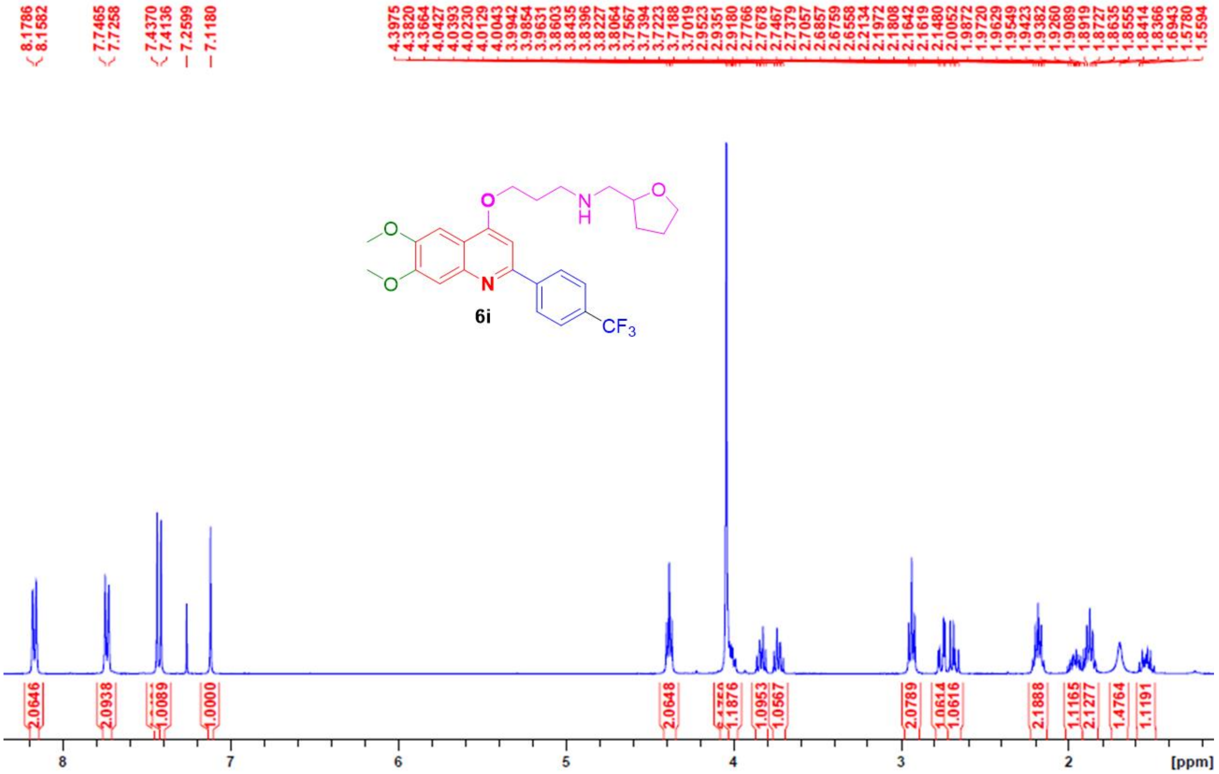

6i
[ppm]

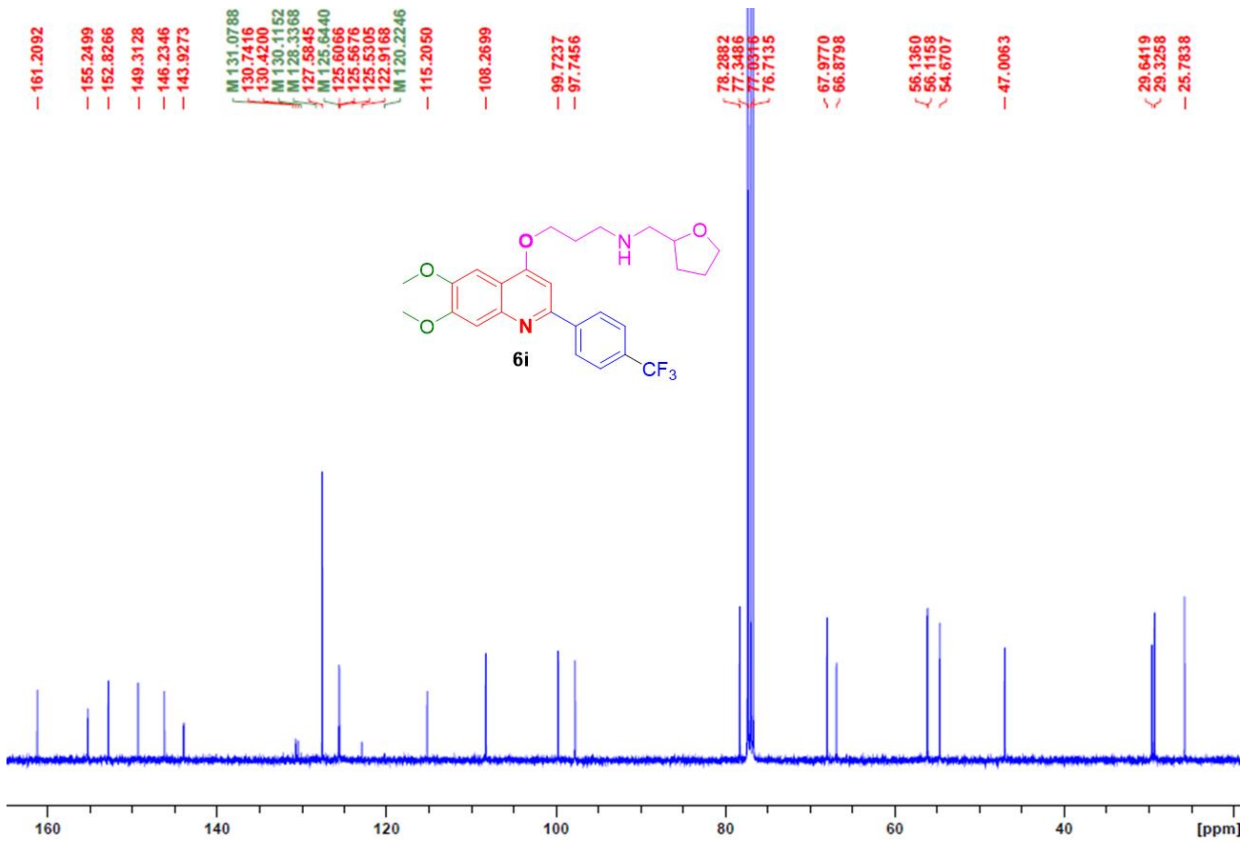

_19F/CDCl3

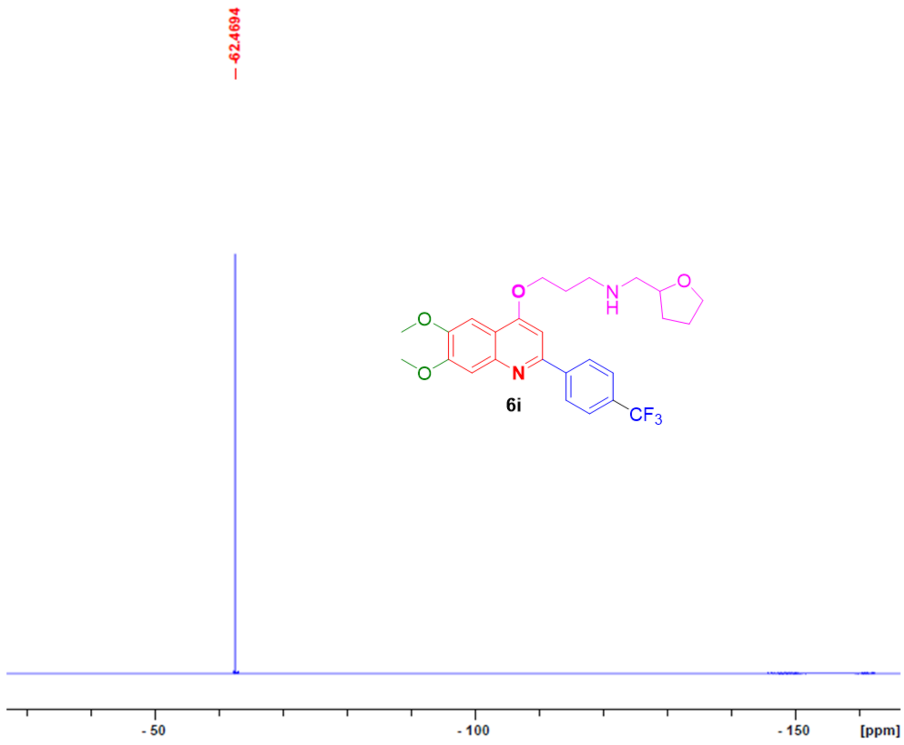

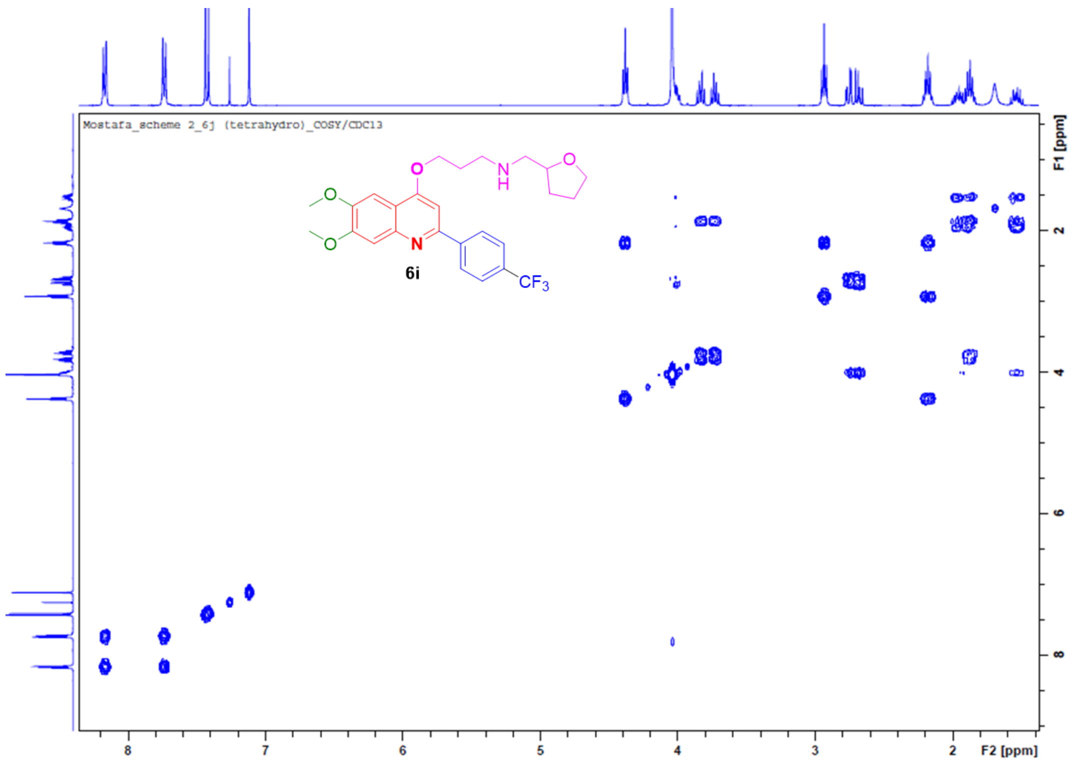
Mostafa_scheme 2_6j (tetrahydro)_COSY/CDCl3
6i
CF3
F1 [ppm]
F2 [ppm]

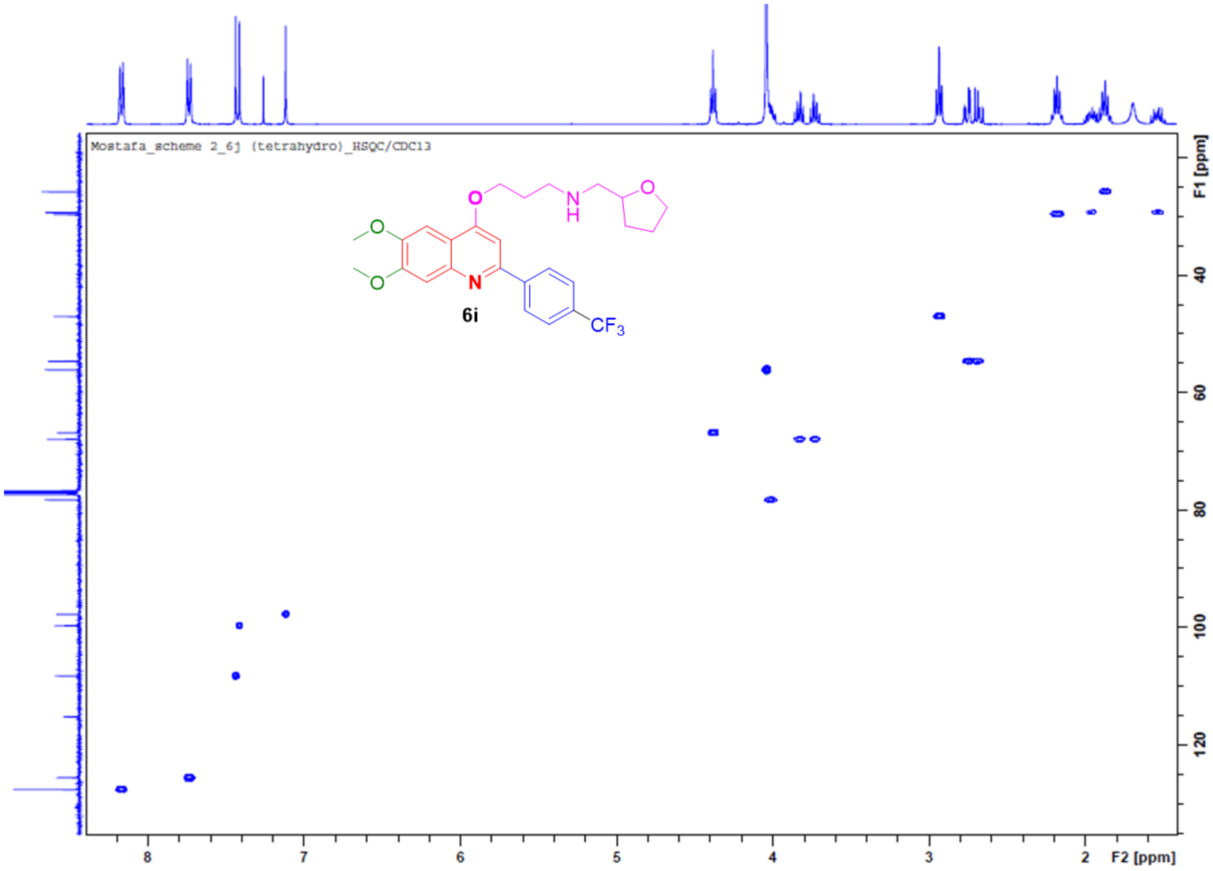
Mostafa_scheme 2_6j (tetrahydro)_HSQC/CDCl3
6i
CF3
F1 [ppm]
F2 [ppm]

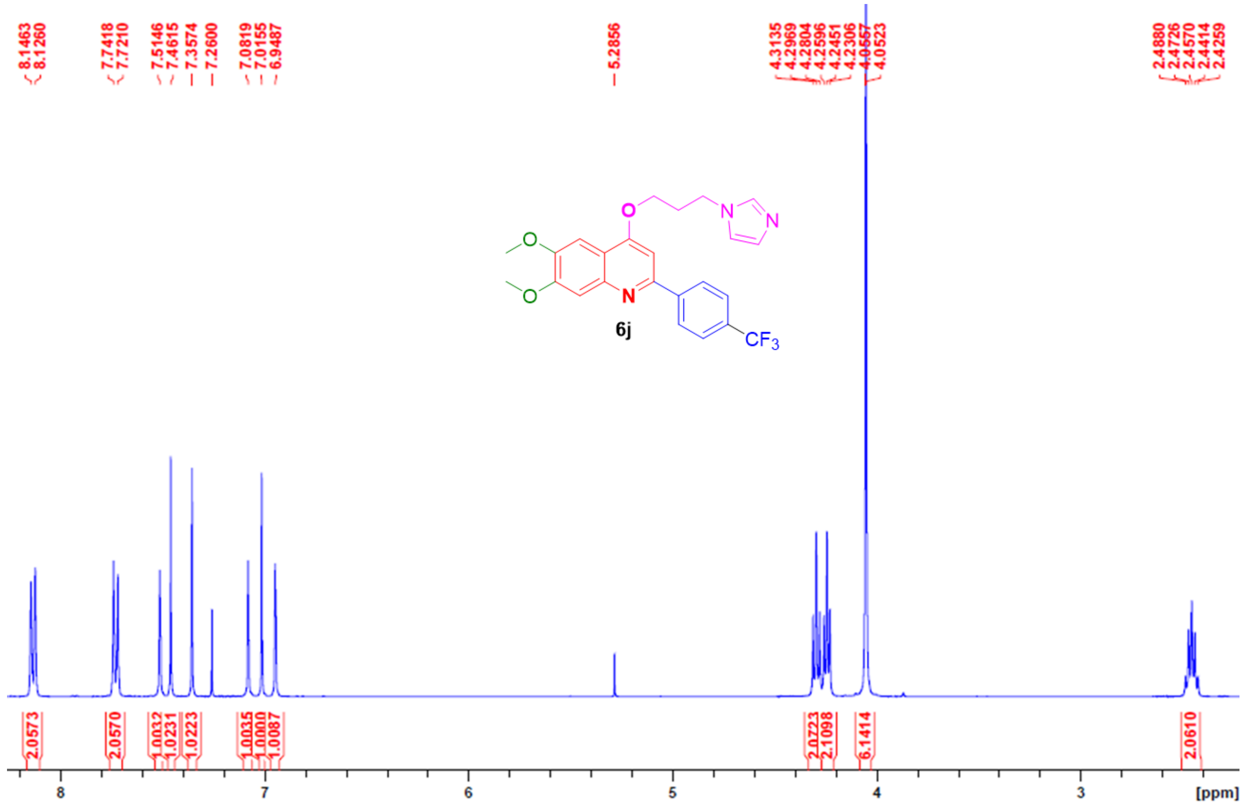
6j
CF3
[ppm]

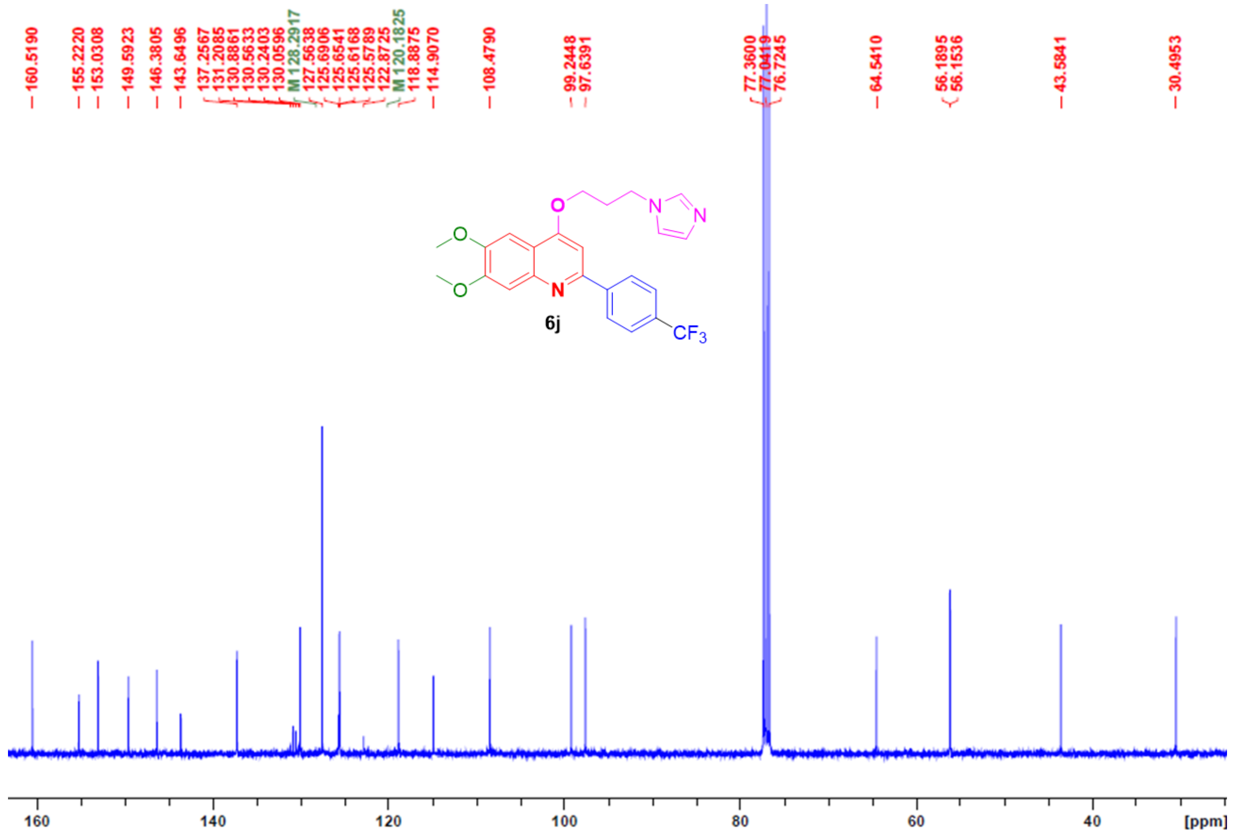
6j
CF3
[ppm]

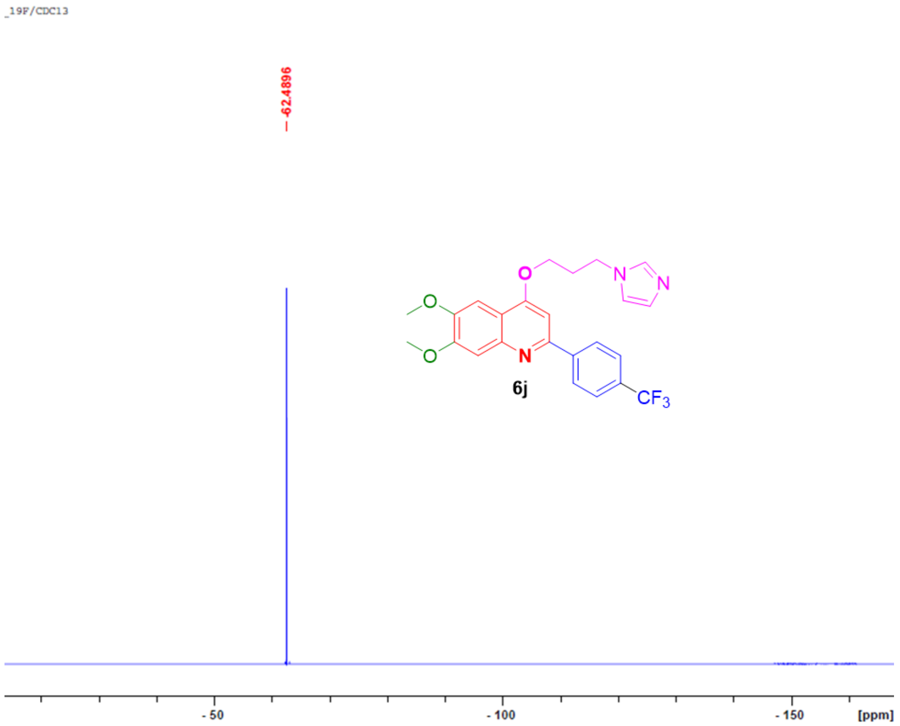
_19F/CDCl3
-62.4896
6j
CF3
- 50
- 100
- 150
[ppm]

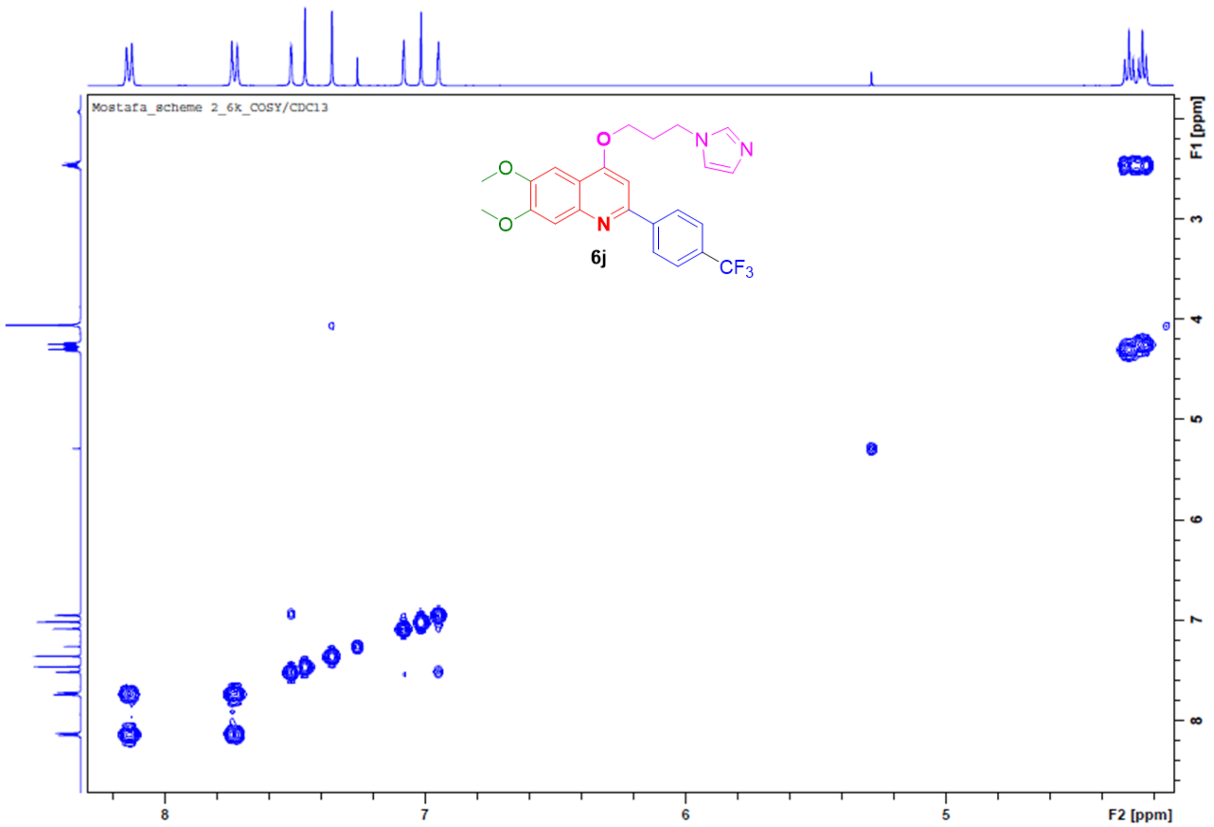
Mostafa_scheme 2_6k_COSY/CDCl3
6j
CF3
F1 [ppm]
F2 [ppm]

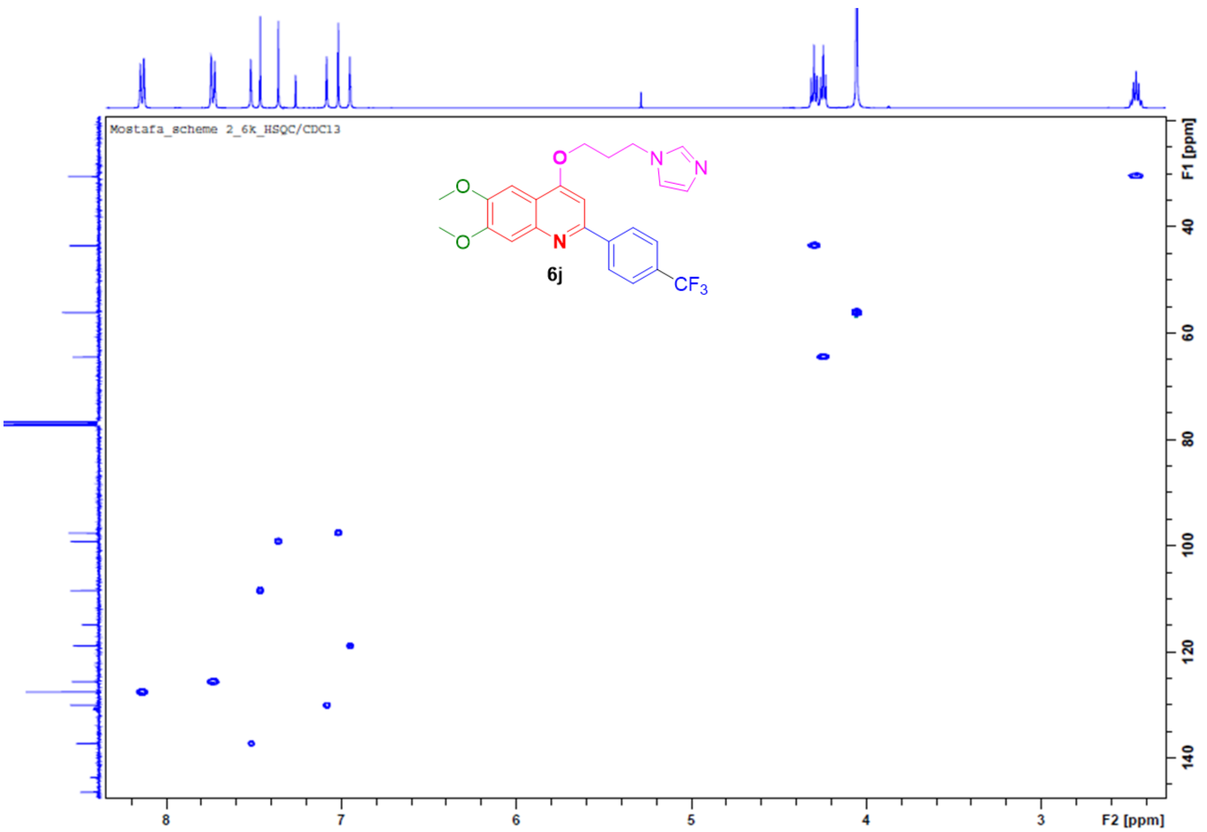
Mostafa_scheme 2_6k_HSQC/CDCl3
6j
CF3
F1 [ppm]
F2 [ppm]

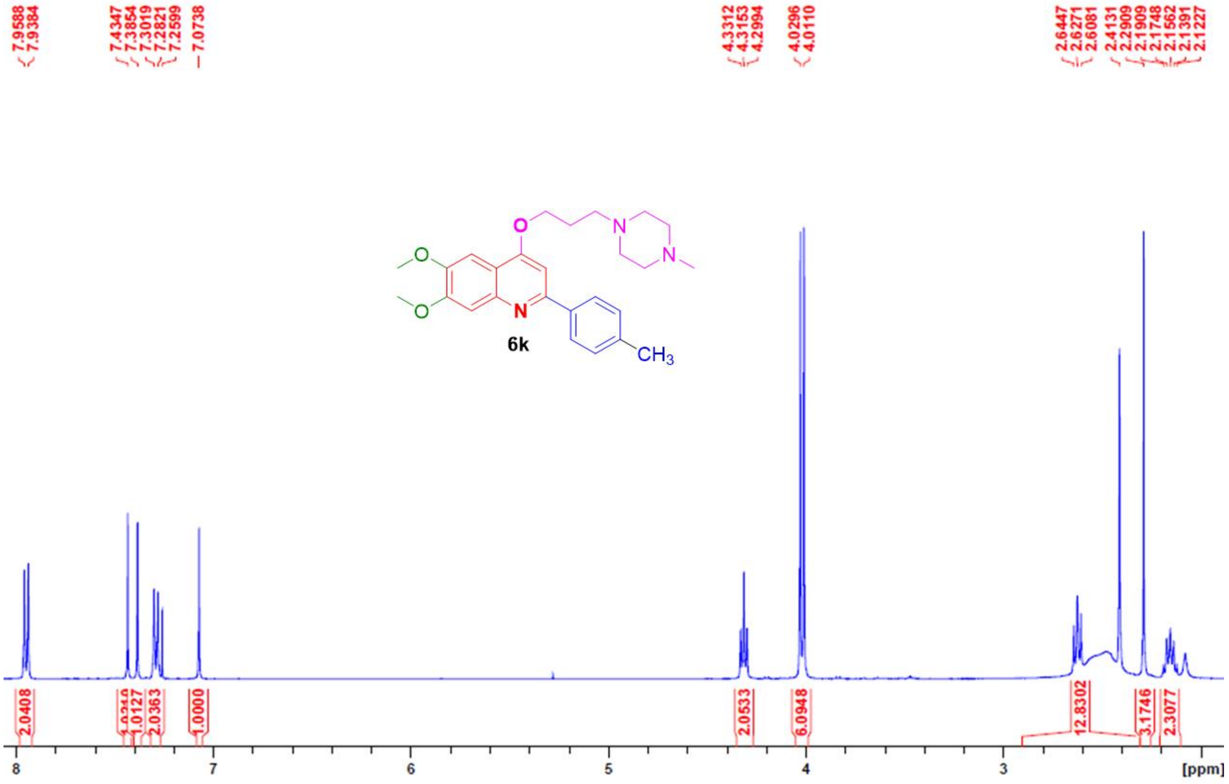
7.9588
7.9384
7.4347
7.3854
7.3019
7.2821
7.2599
7.0738
4.3312
4.3153
4.2994
4.0296
4.0110
2.6447
2.6271
2.6081
2.4131
2.2909
2.1909
2.1748
2.1562
2.1391
2.1227
6k
CH3
2.0408
1.0127
2.0363
1.0000
2.0533
6.0948
12.8302
3.1746
2.3077
[ppm]

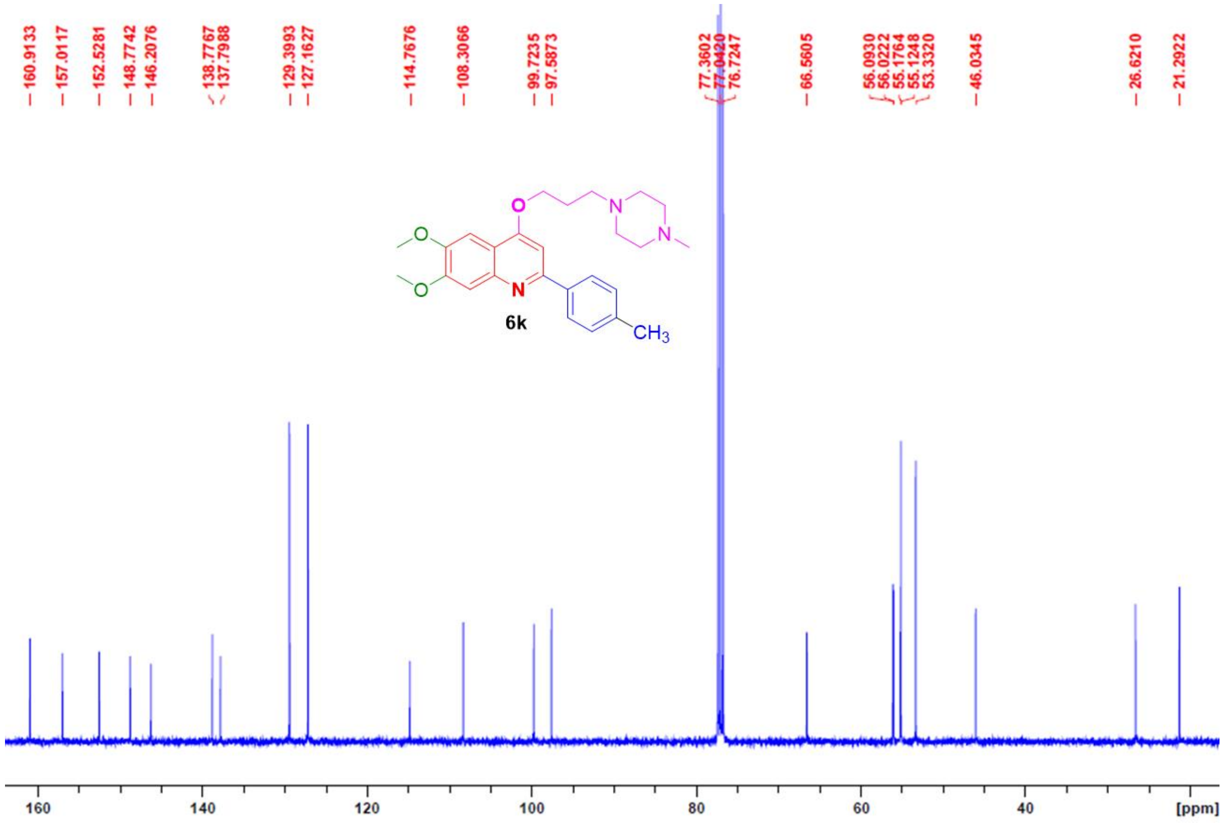
160.9133
157.0117
152.5281
148.7742
146.2076
138.7767
137.7988
129.3993
127.1627
114.7676
108.3066
99.7235
97.5873
77.3602
77.0420
76.7247
66.5605
56.0930
56.0222
55.1764
55.1248
53.3320
46.0345
26.6210
21.2922
6k
[ppm]

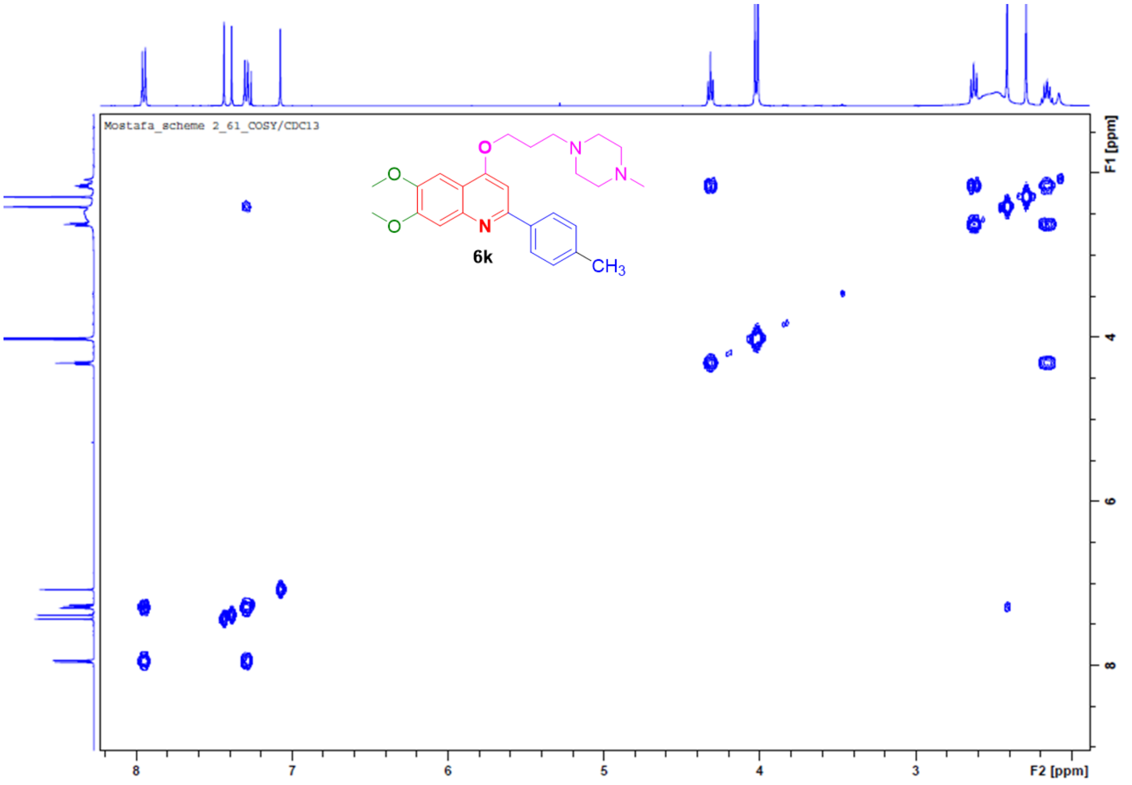
Mostafa_scheme 2_61_COSY/CDCl3
6k
F1 [ppm]
F2 [ppm]

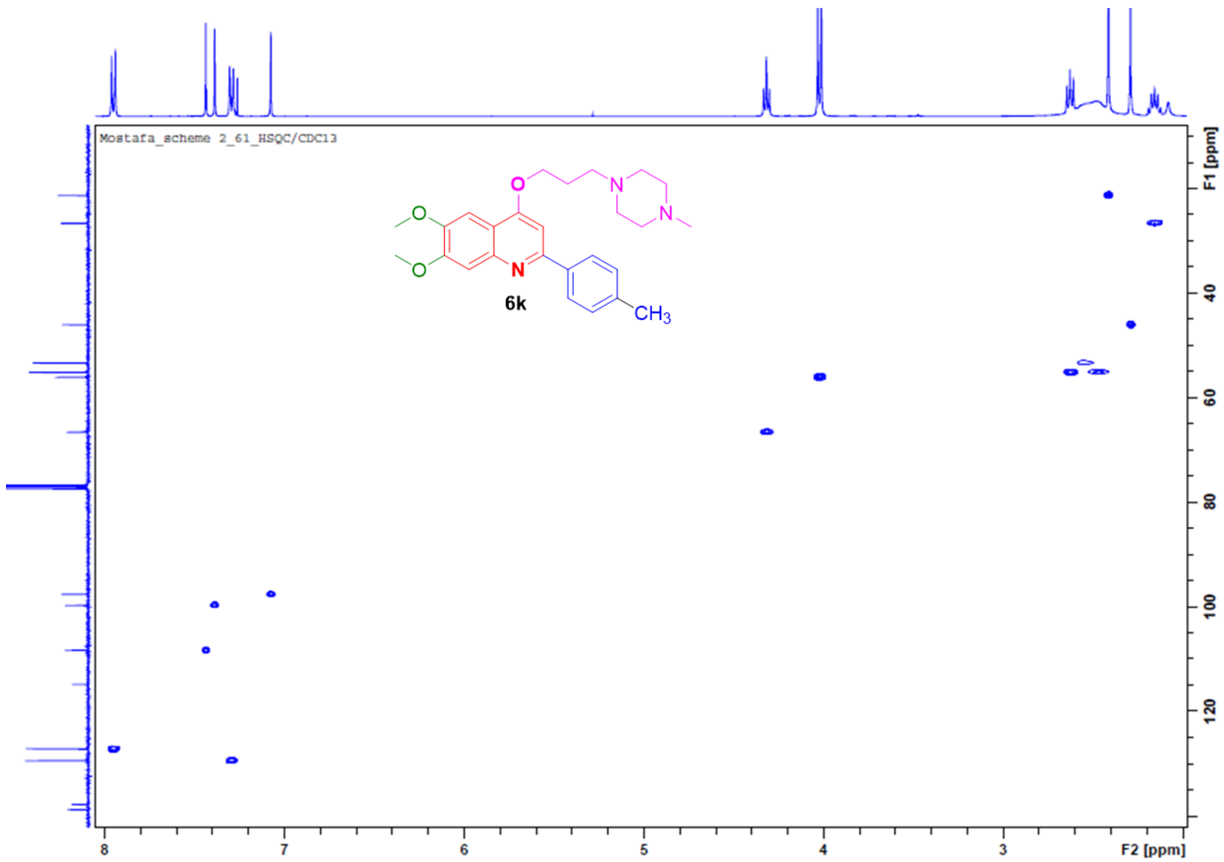
Mostafa_scheme 2_61_HSQC/CDCl3
6k
F1 [ppm]
F2 [ppm]

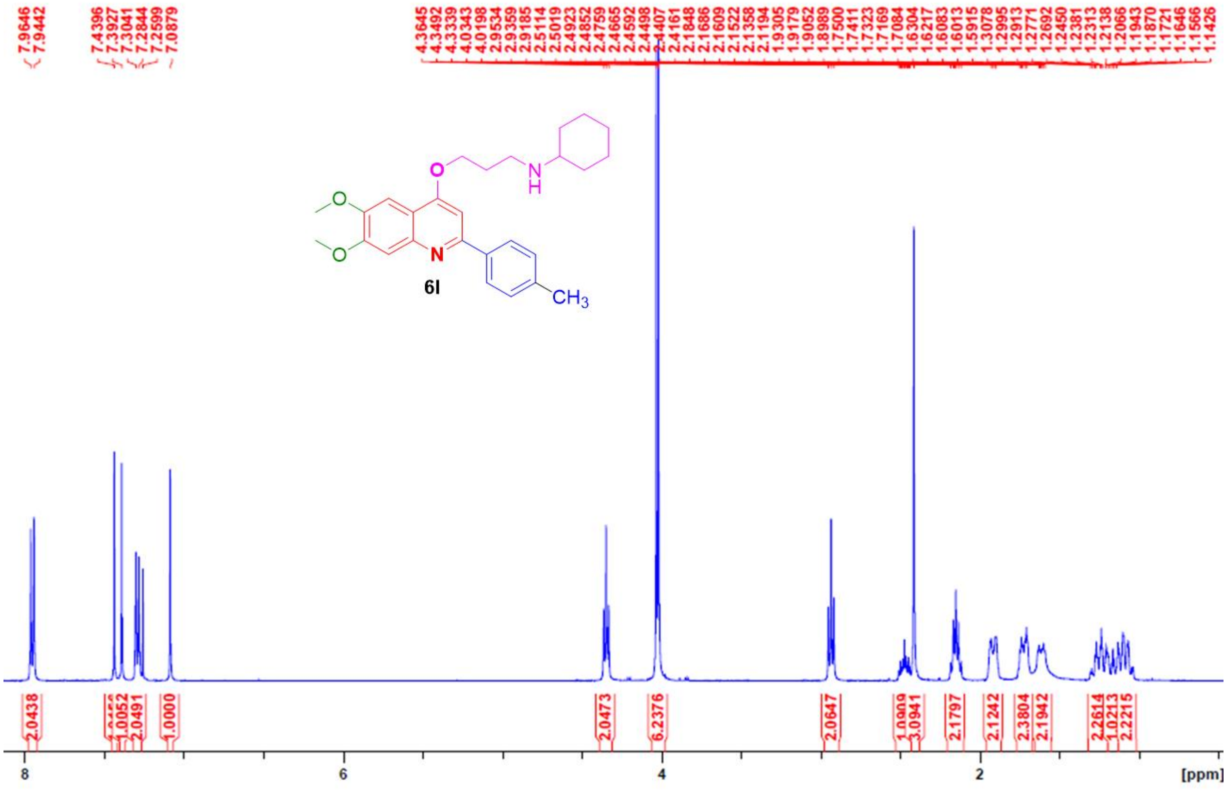
6l
[ppm]

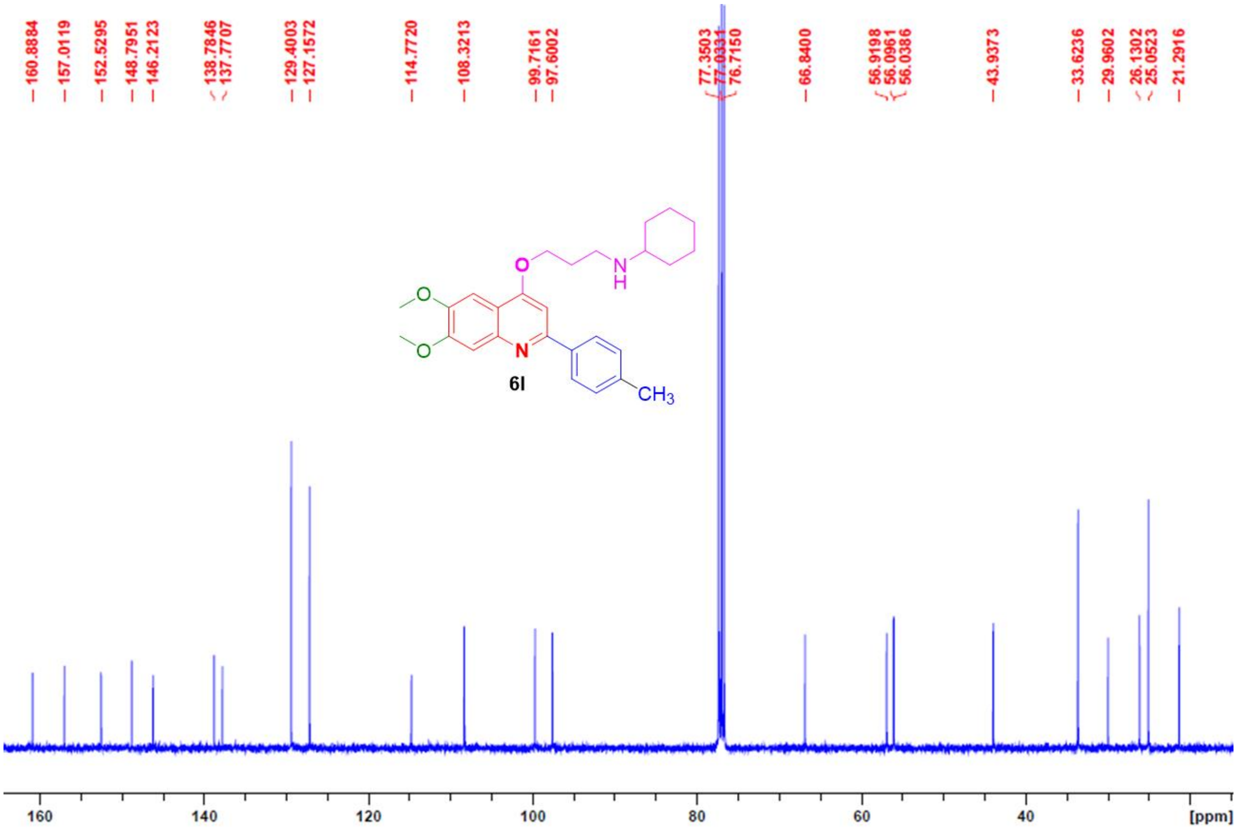
160.8884
157.0119
152.5295
148.7951
146.2123
138.7846
137.7707
129.4003
127.1572
114.7720
108.3213
99.7161
97.6002
77.3503
77.0331
76.7150
66.8400
56.9198
56.0961
56.0386
43.9373
33.6236
29.9602
26.1302
25.0523
21.2916
6l
CH3
160
140
120
100
80
60
40
[ppm]

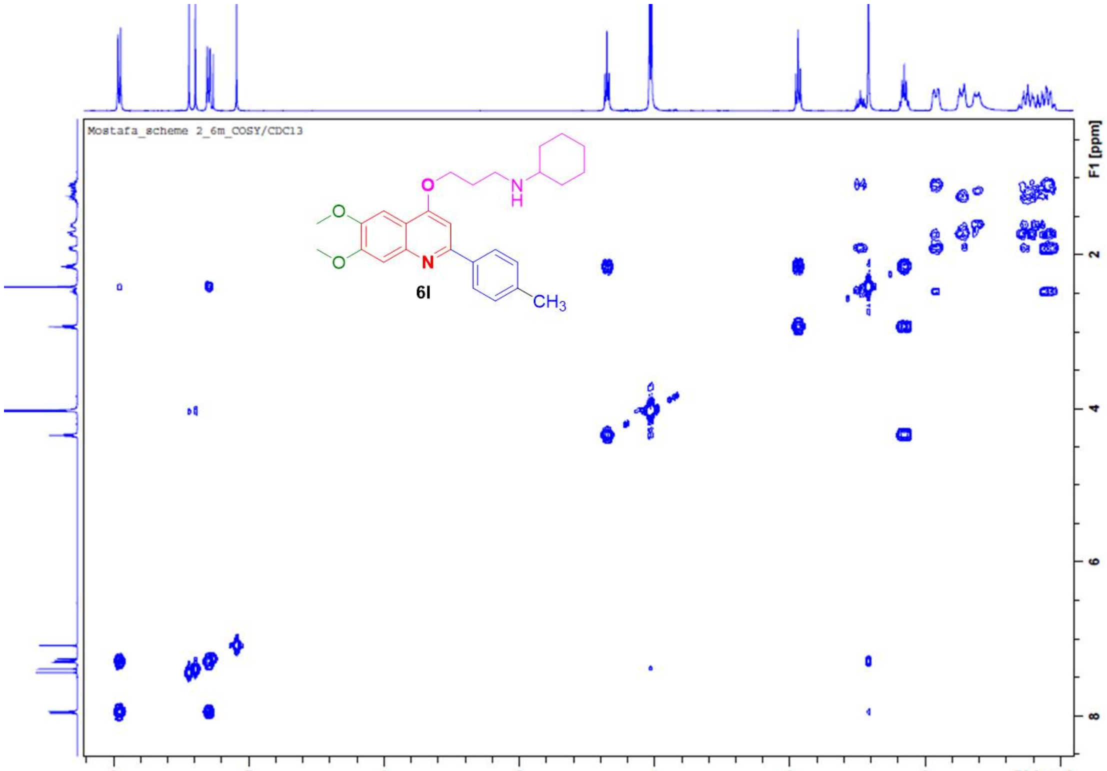
Mostafa_scheme 2_6m_COSY/CDCl3
6l
CH3
F1 [ppm]
F2 [ppm]

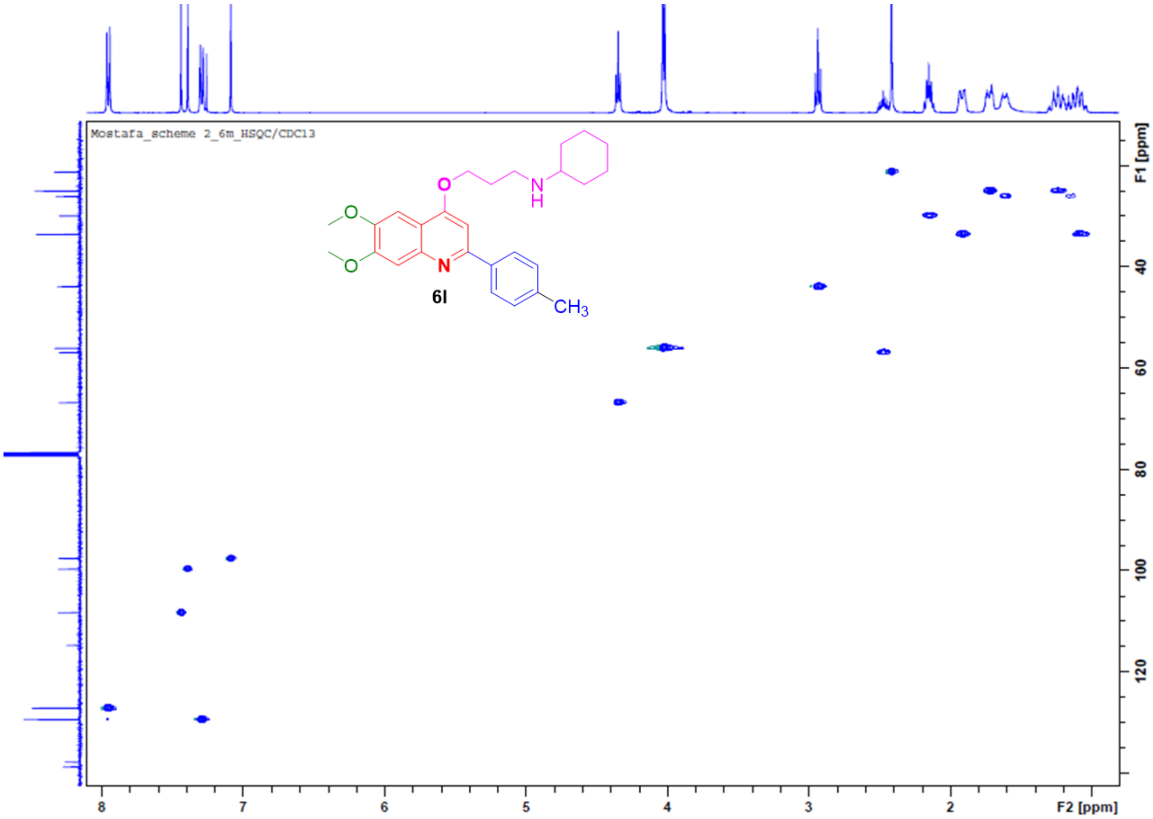
Mostafa_scheme 2_6m_HSQC/CDCl3
6l
F1 [ppm]
F2 [ppm]

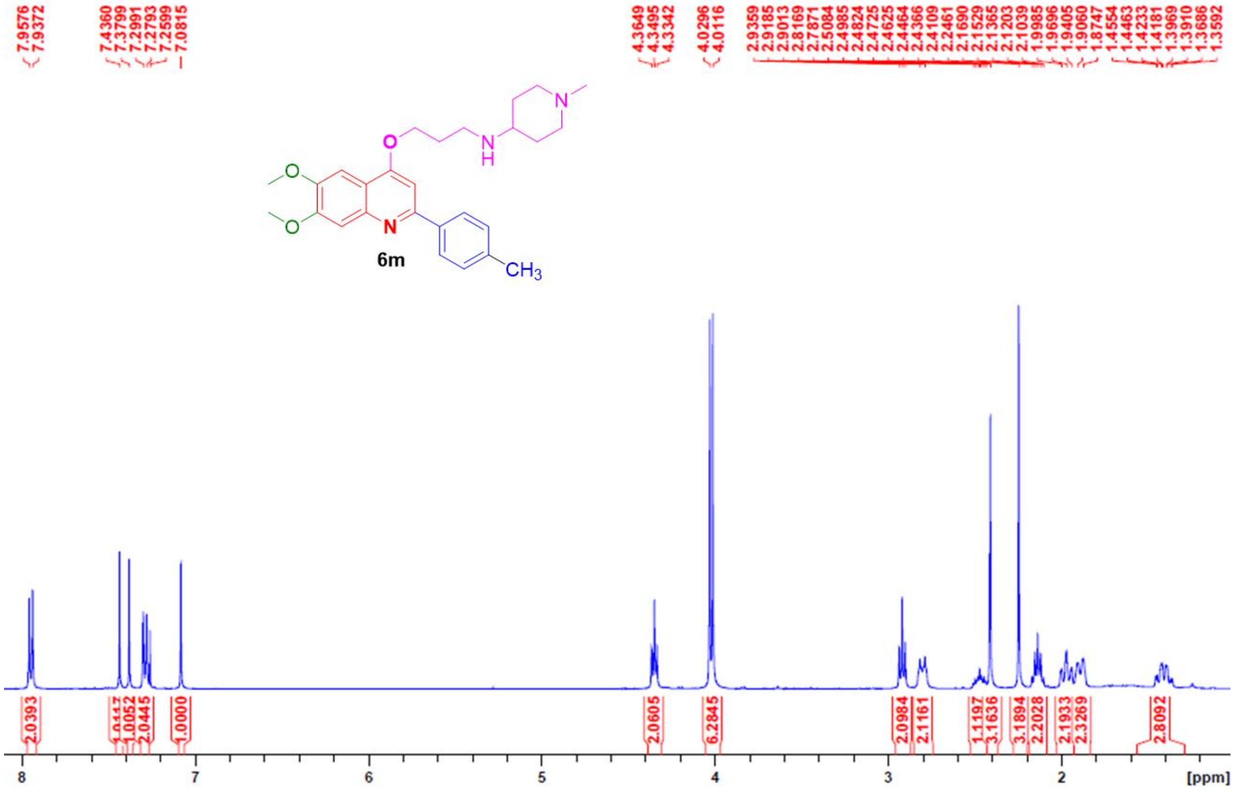
6m
[ppm]

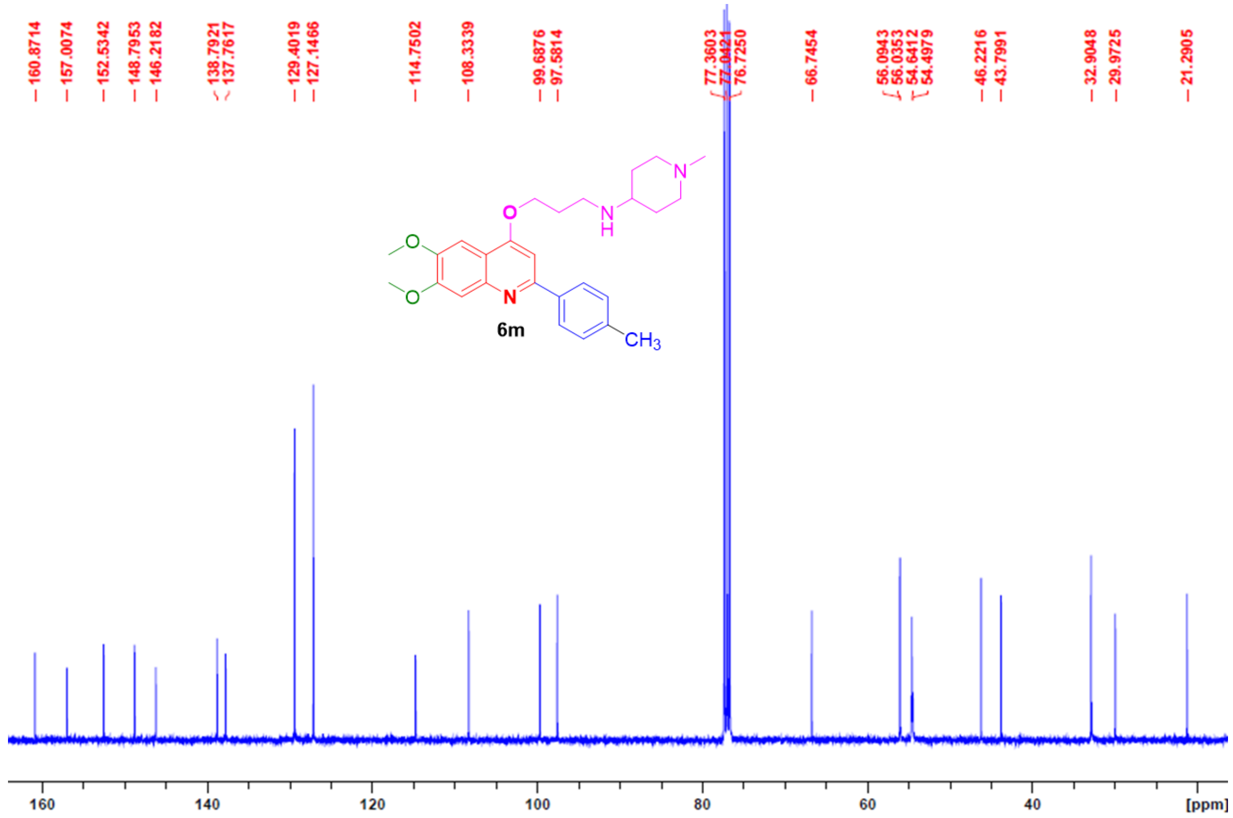

160.8714
157.0074
152.5342
148.7953
146.2182
138.7921
137.7617
129.4019
127.1466
114.7502
108.3339
99.6876
97.5814
77.3603
77.0421
76.7250
66.7454
56.0943
56.0353
54.6412
54.4979
46.2216
43.7991
32.9048
29.9725
21.2905
6m
[ppm]

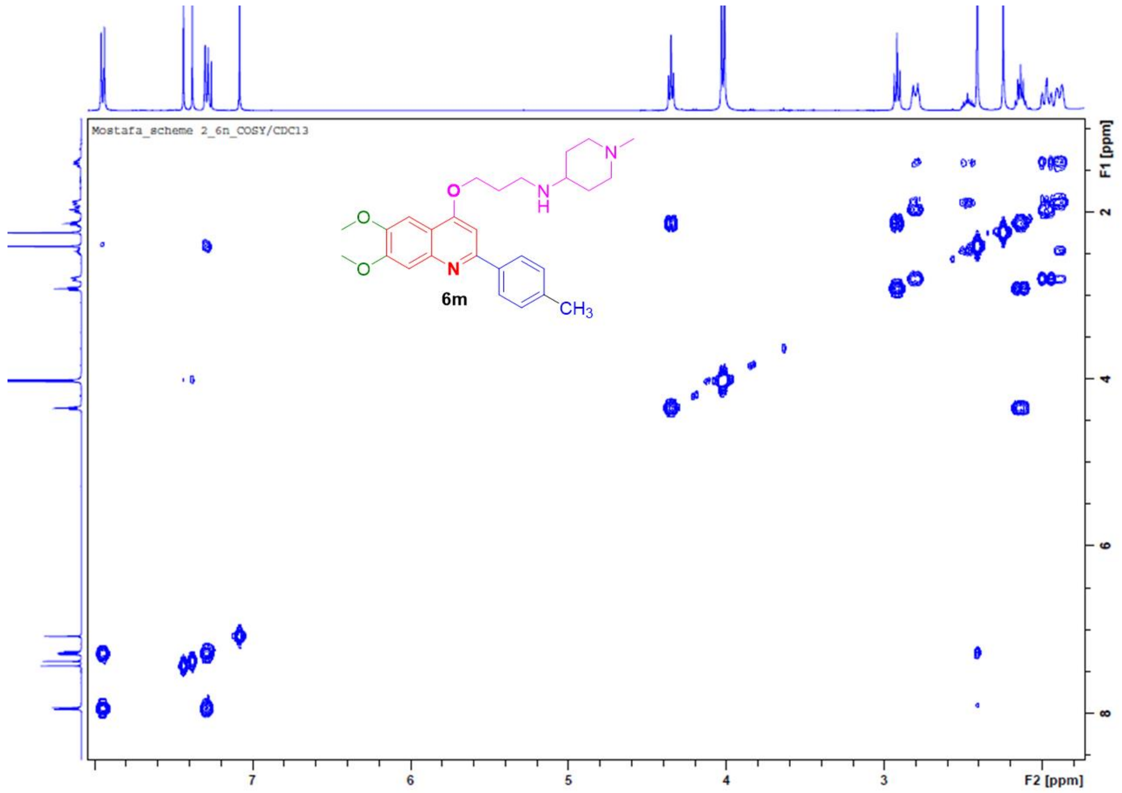

Mostafa_scheme 2_6n_COSY/CDCl3
6m
F1 [ppm]
F2 [ppm]

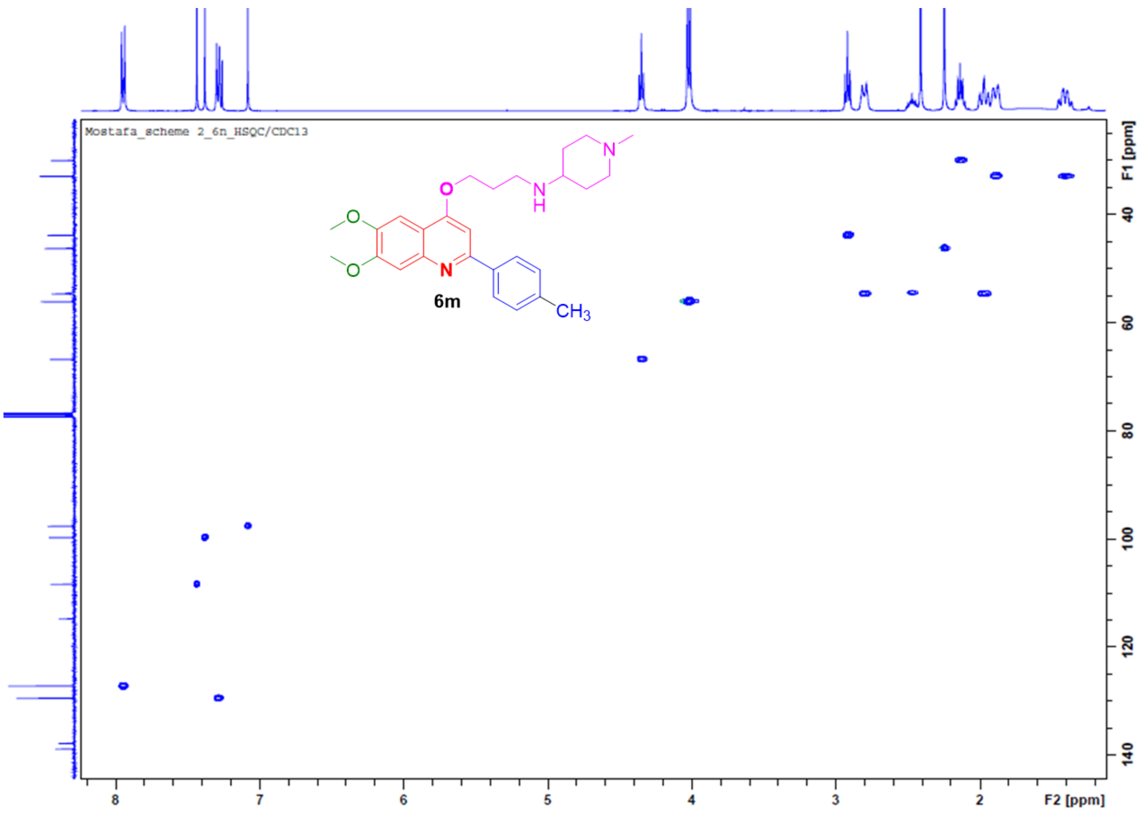

Mostafa_scheme 2_6n_HSQC/CDCl3
6m
CH3
F1 [ppm]
F2 [ppm]

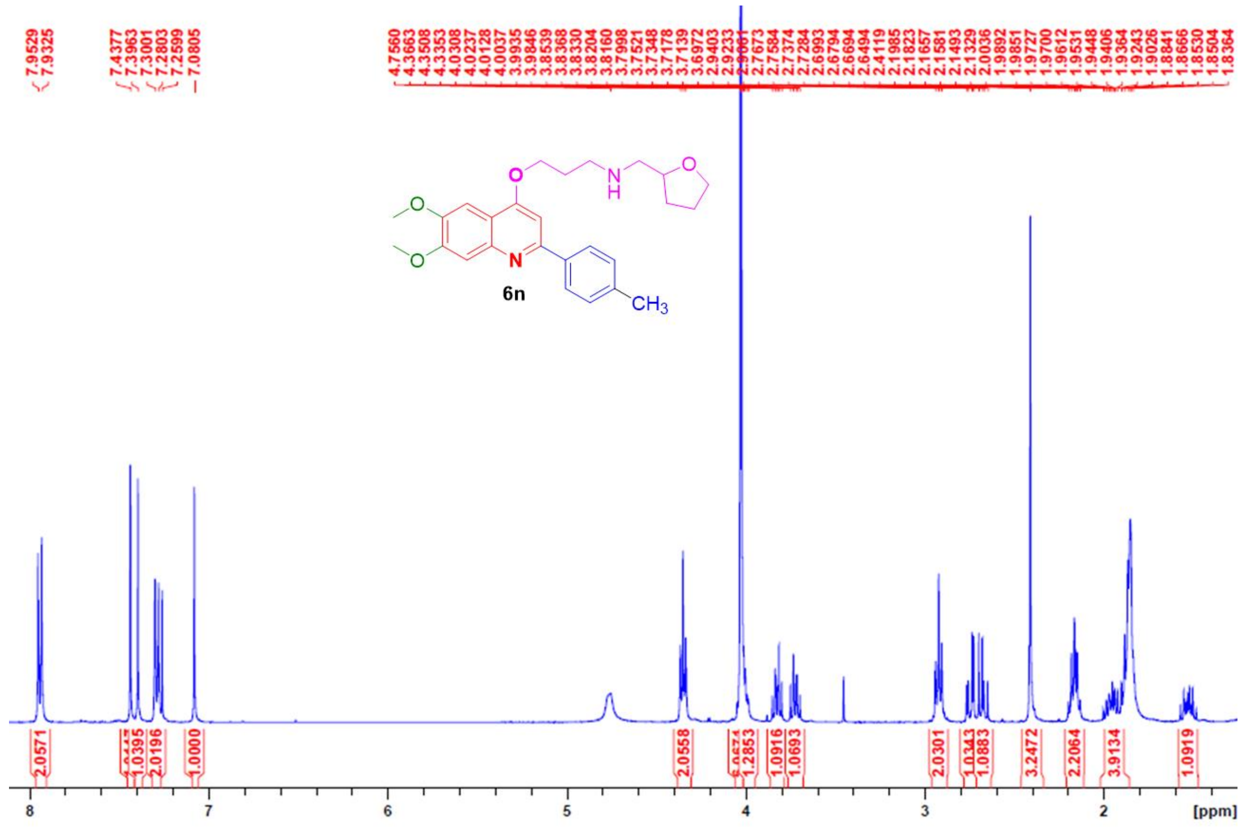

7.9529
7.9325
7.4377
7.3963
7.3001
7.2803
7.2599
7.0805
4.7560
4.3663
4.3508
4.3353
4.0308
4.0237
4.0128
4.0037
3.9935
3.9846
3.8539
3.8368
3.8330
3.8204
3.8160
3.7998
3.7521
3.7348
3.7178
3.7139
3.6972
2.9403
2.9233
2.7673
2.7584
2.7374
2.7284
2.6993
2.6794
2.6694
2.6494
2.4119
2.1985
2.1823
2.1657
2.1581
2.1493
2.1329
2.0036
1.9892
1.9851
1.9727
1.9700
1.9612
1.9531
1.9448
1.9406
1.9364
1.9243
1.9026
1.8841
1.8666
1.8530
1.8504
1.8364
6n
CH3
2.0571
1.0395
2.0196
1.0000
2.0558
1.2853
1.0916
1.0693
2.0301
1.0343
1.0883
3.2472
2.2064
3.9134
1.0919
[ppm]

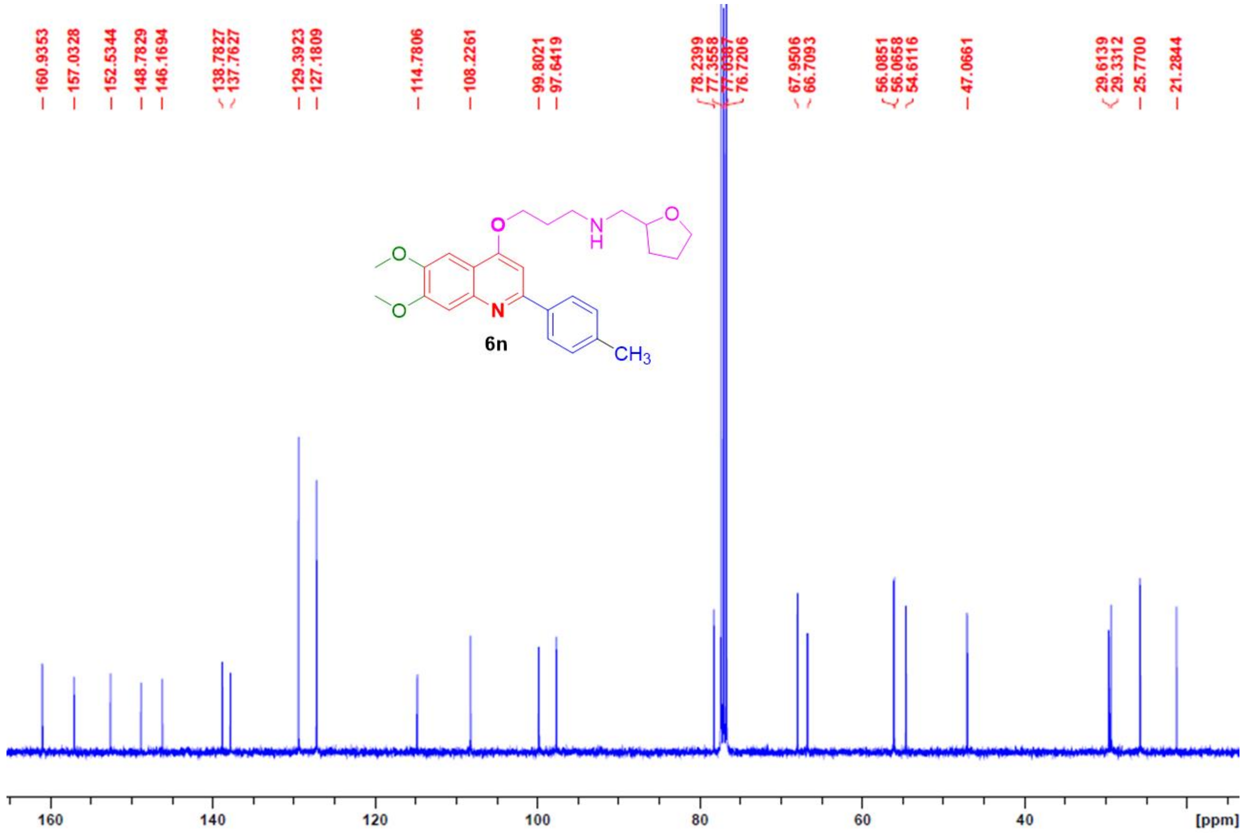
160.9353
157.0328
152.5344
148.7829
146.1694
138.7827
137.7627
129.3923
127.1809
114.7806
108.2261
99.8021
97.6419
78.2399
77.3558
77.0387
76.7206
67.9506
66.7093
56.0851
56.0658
54.6116
47.0661
29.6139
29.3312
25.7700
21.2844
6n
[ppm]

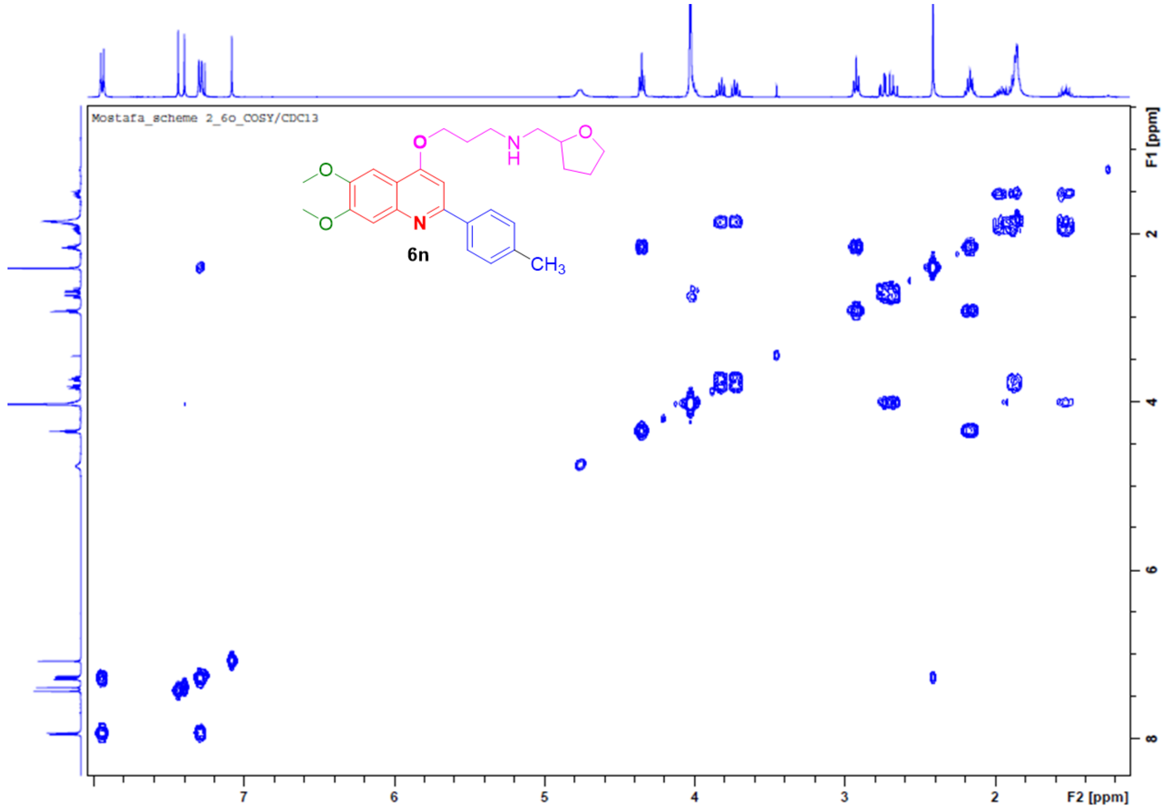
Mostafa_scheme 2_6o_COSY/CDCl3
6n
F1 [ppm]
F2 [ppm]

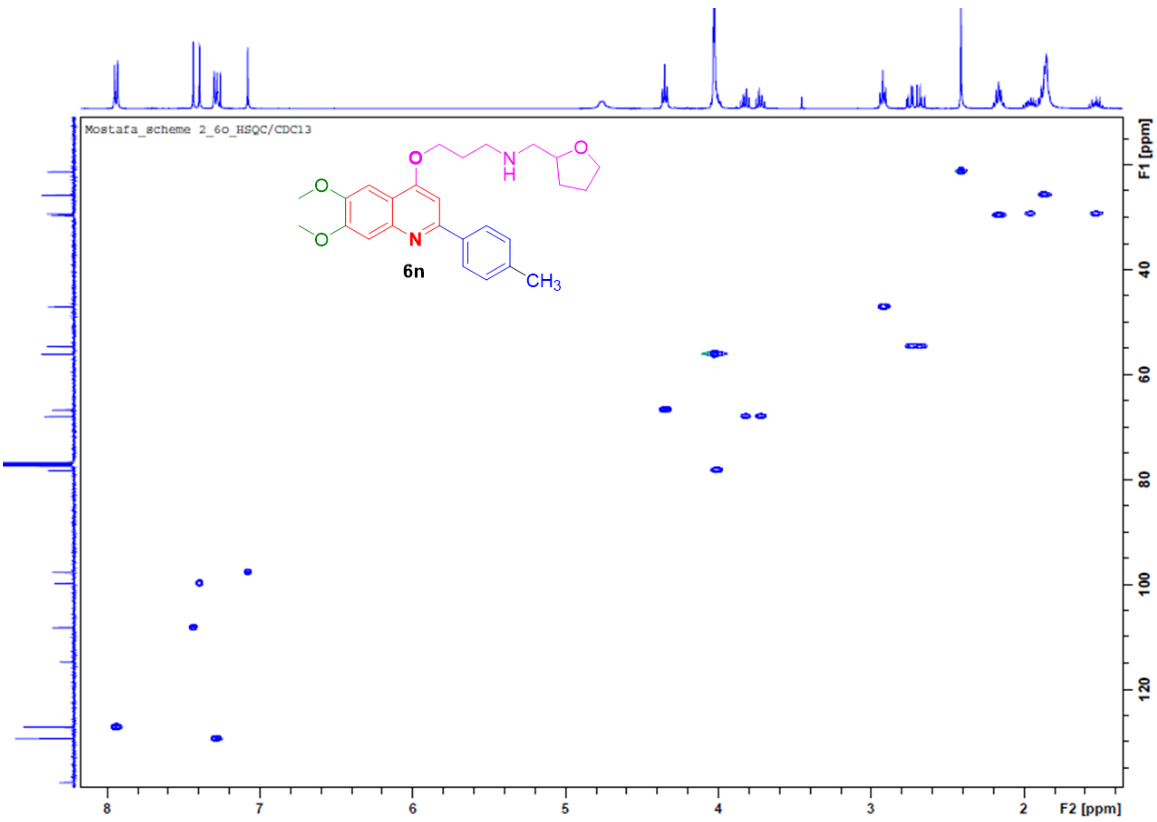
Mostafa_scheme 2_6o_HSQC/CDCl3
6n
CH3
F1 [ppm]
F2 [ppm]

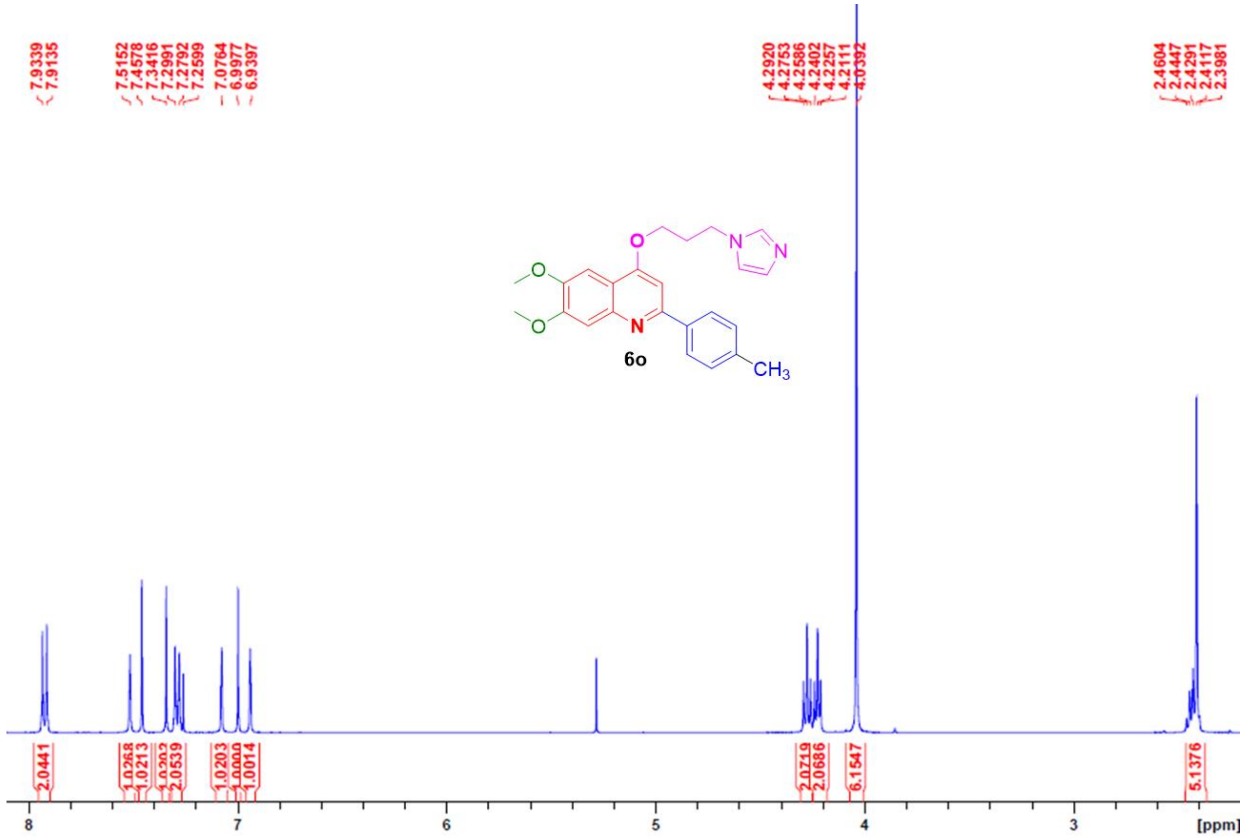
7.9339
7.9135
7.5152
7.4578
7.3416
7.2991
7.2792
7.2599
7.0764
6.9977
6.9397
4.2920
4.2753
4.2586
4.2402
4.2257
4.2111
4.0392
2.4604
2.4447
2.4291
2.4117
2.3981
6o
CH3
2.0441
1.0268
1.0213
2.0539
1.0203
1.0014
2.0719
2.0686
6.1547
5.1376
[ppm]

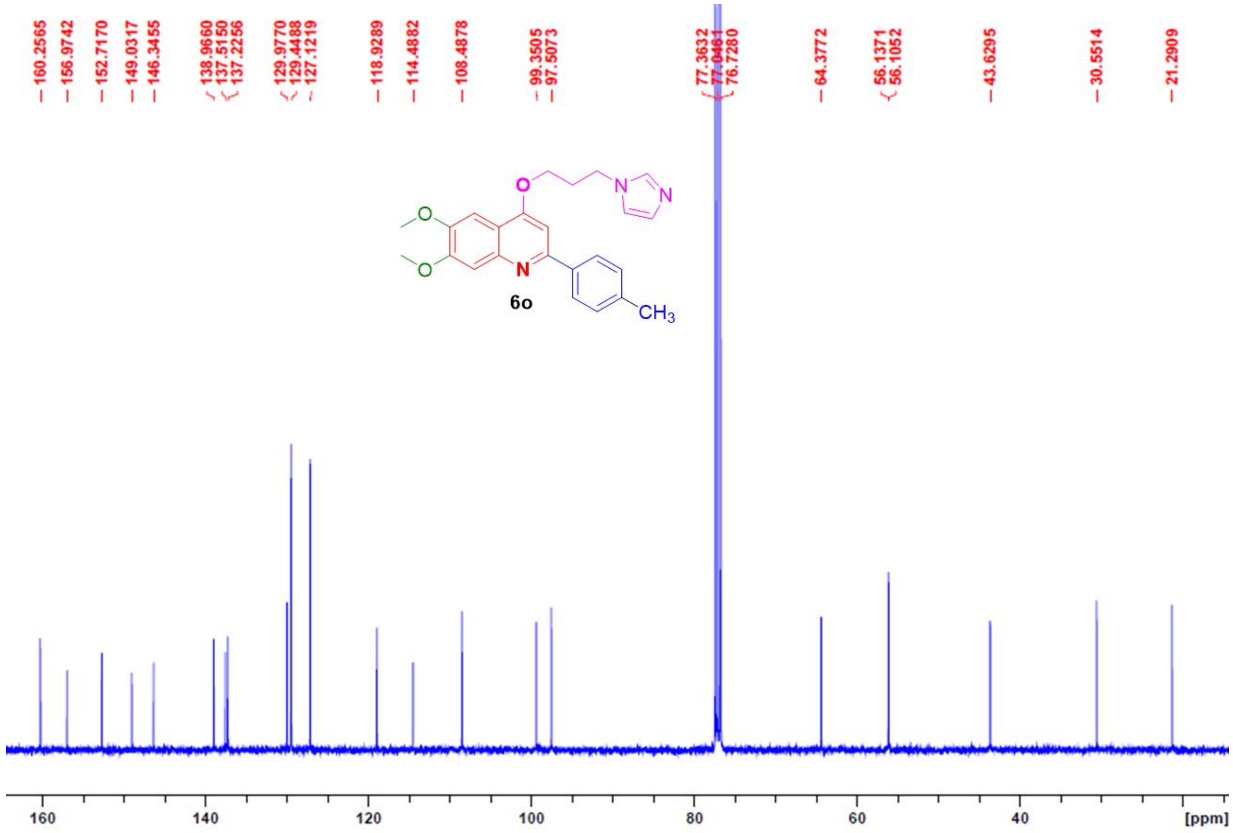

160.2565
156.9742
152.7170
149.0317
146.3455
138.9660
137.5150
137.2256
129.9770
129.4488
127.1219
118.9289
114.4882
108.4878
99.3505
97.5073
77.3632
77.0461
76.7280
64.3772
56.1371
56.1052
43.6295
30.5514
21.2909
6o
[ppm]

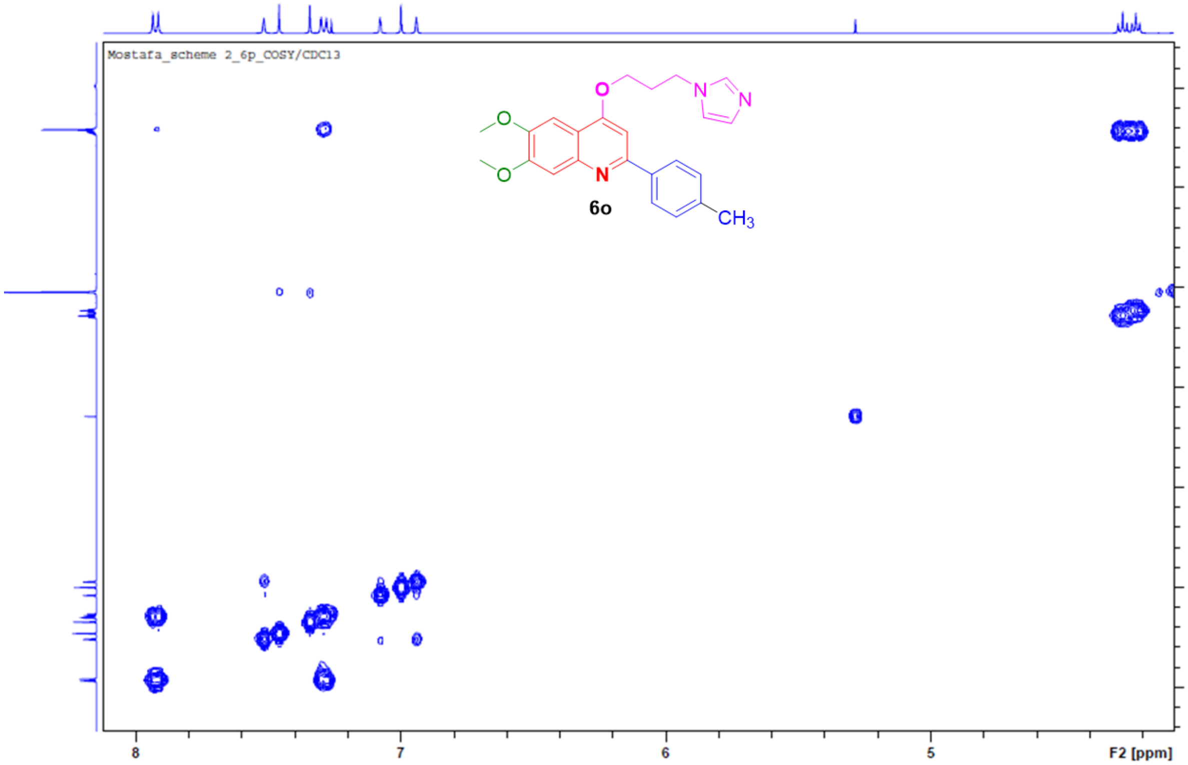

Mostafa_scheme 2_6p_COSY/CDCl3
6o
F1 [ppm]
F2 [ppm]

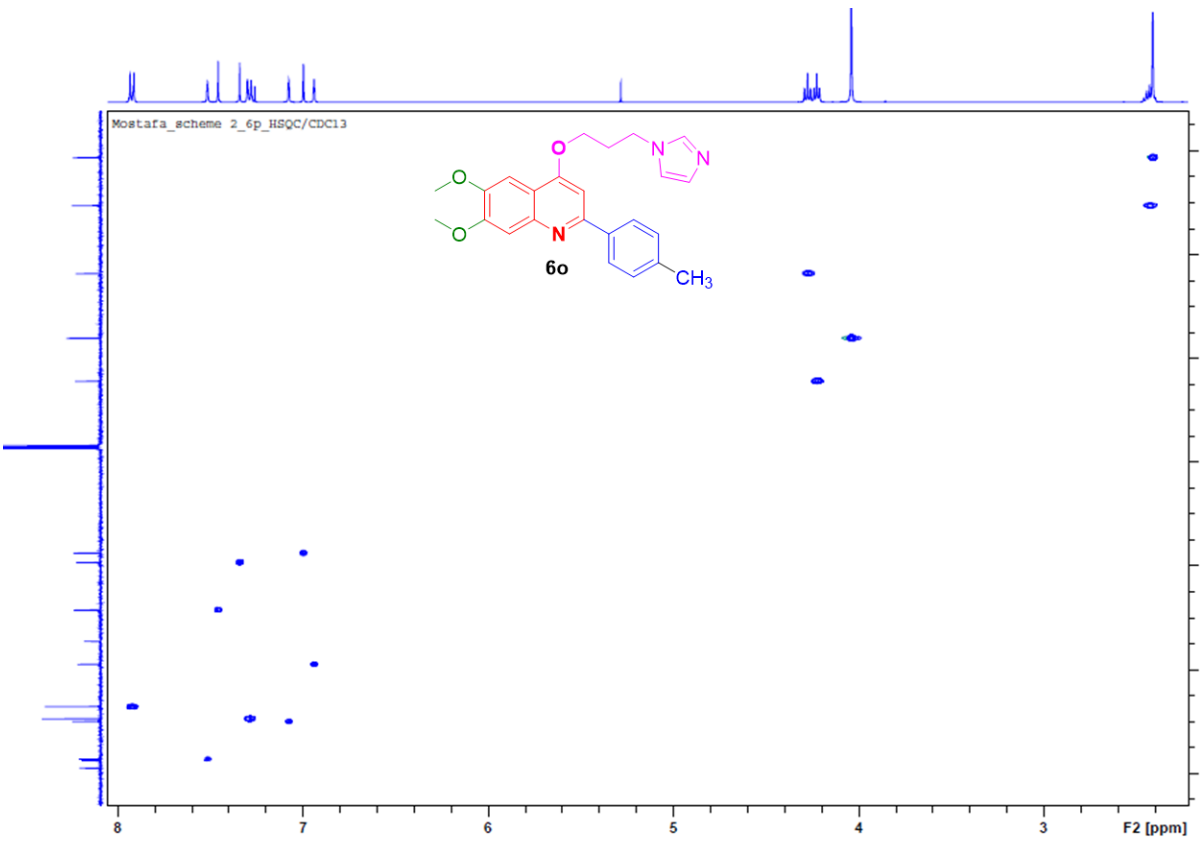

Mostafa_scheme 2_6p_HSQC/CDCl3
6o
CH3
F1 [ppm]
F2 [ppm]

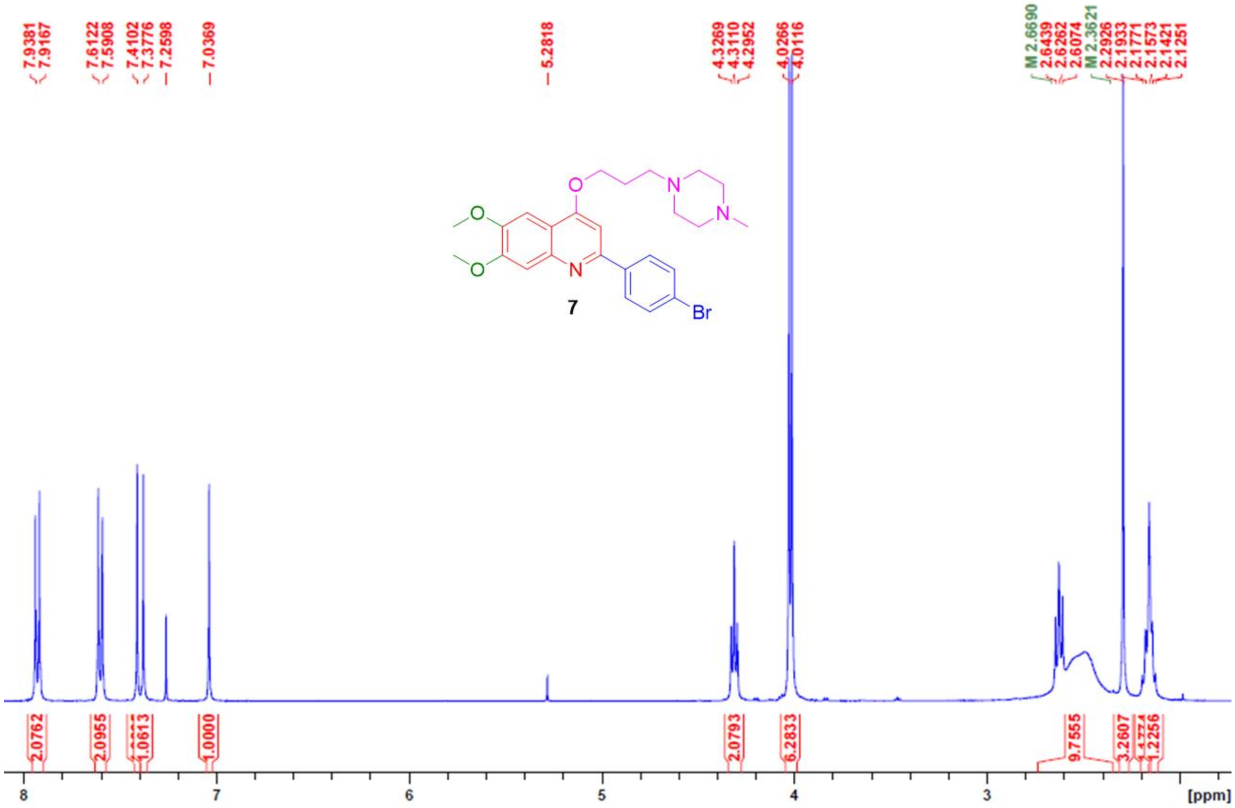

7.9381
7.9167
7.6122
7.5908
7.4102
7.3776
7.2598
7.0369
5.2818
4.3269
4.3110
4.2952
4.0266
4.0116
M 2.6690
2.6439
2.6262
2.6074
M 2.3621
2.2926
2.1933
2.1771
2.1573
2.1421
2.1251
7
Br
2.0762
2.0955
1.0613
1.0000
2.0793
6.2833
9.7555
3.2607
1.2256
[ppm]

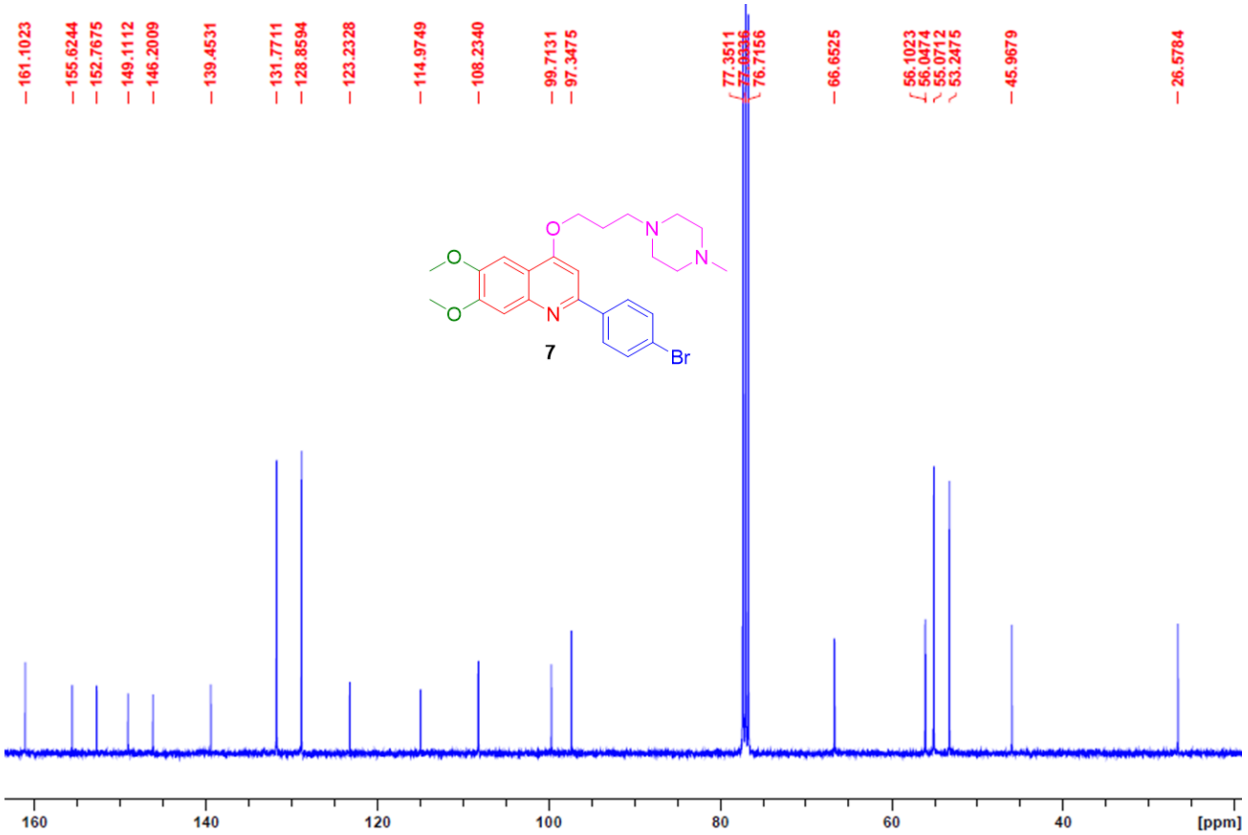
161.1023
155.6244
152.7675
149.1112
146.2009
139.4531
131.7711
128.8594
123.2328
114.9749
108.2340
99.7131
97.3475
77.3511
77.0336
76.7156
66.6525
56.1023
56.0474
55.0712
53.2475
45.9679
26.5784
O
N
N
O
O
N
Br
7
160
140
120
100
80
60
40
[ppm]

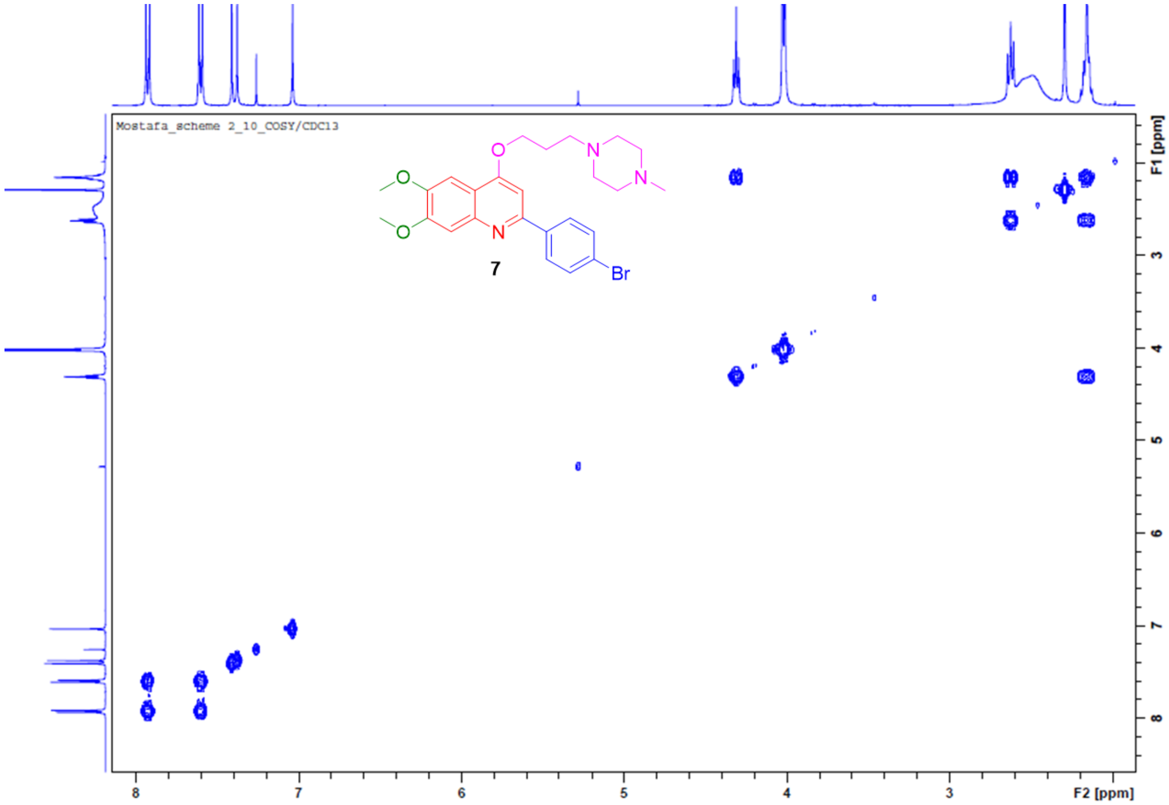
Mostafa_scheme 2_10_COSY/CDCl3
O
N
N
O
O
N
Br
7
F1 [ppm]
3
4
5
6
7
8
8
7
6
5
4
3
F2 [ppm]

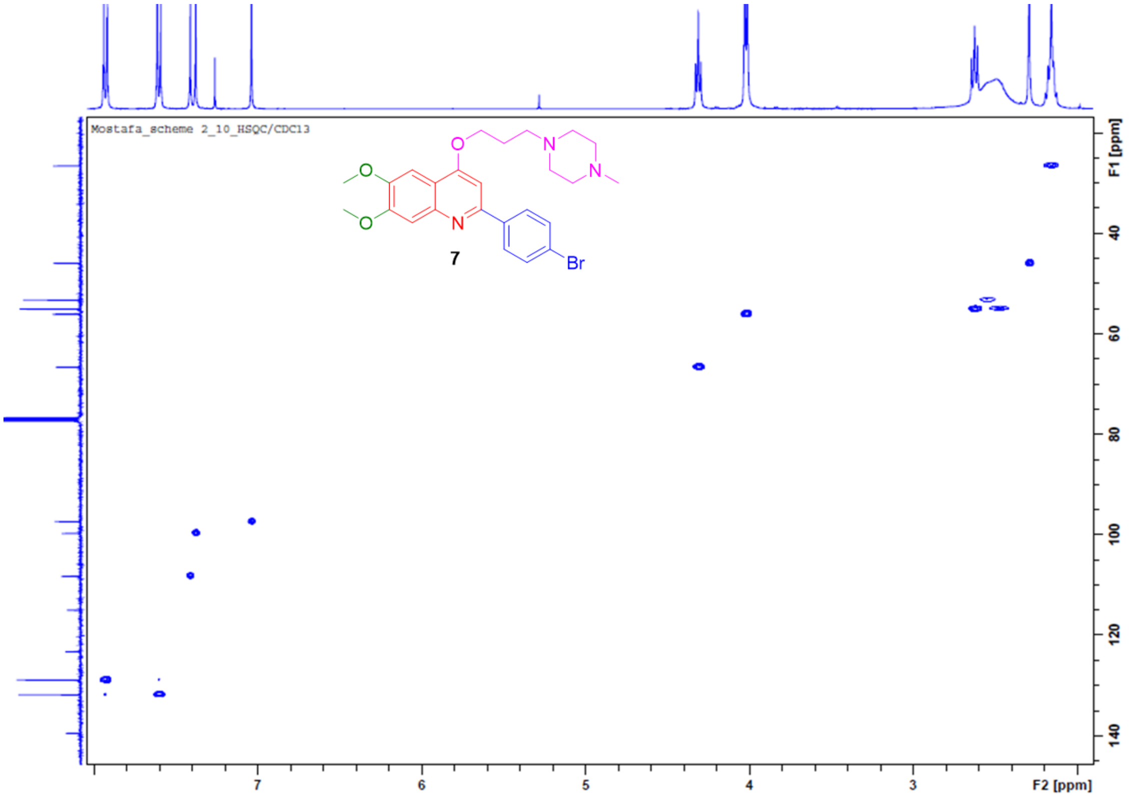
Mostafa_scheme 2_10_HSQC/CDCl3
7
Br
F1 [ppm]
F2 [ppm]

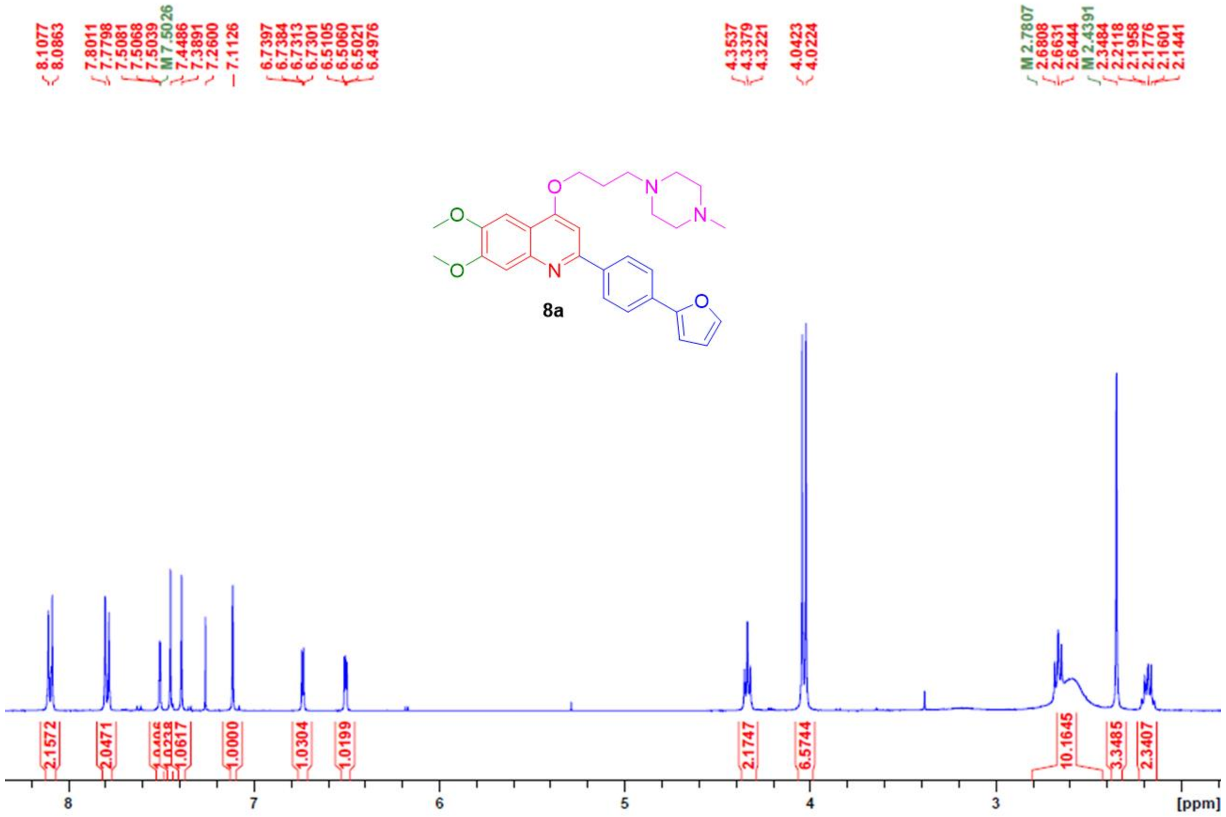
8a
[ppm]

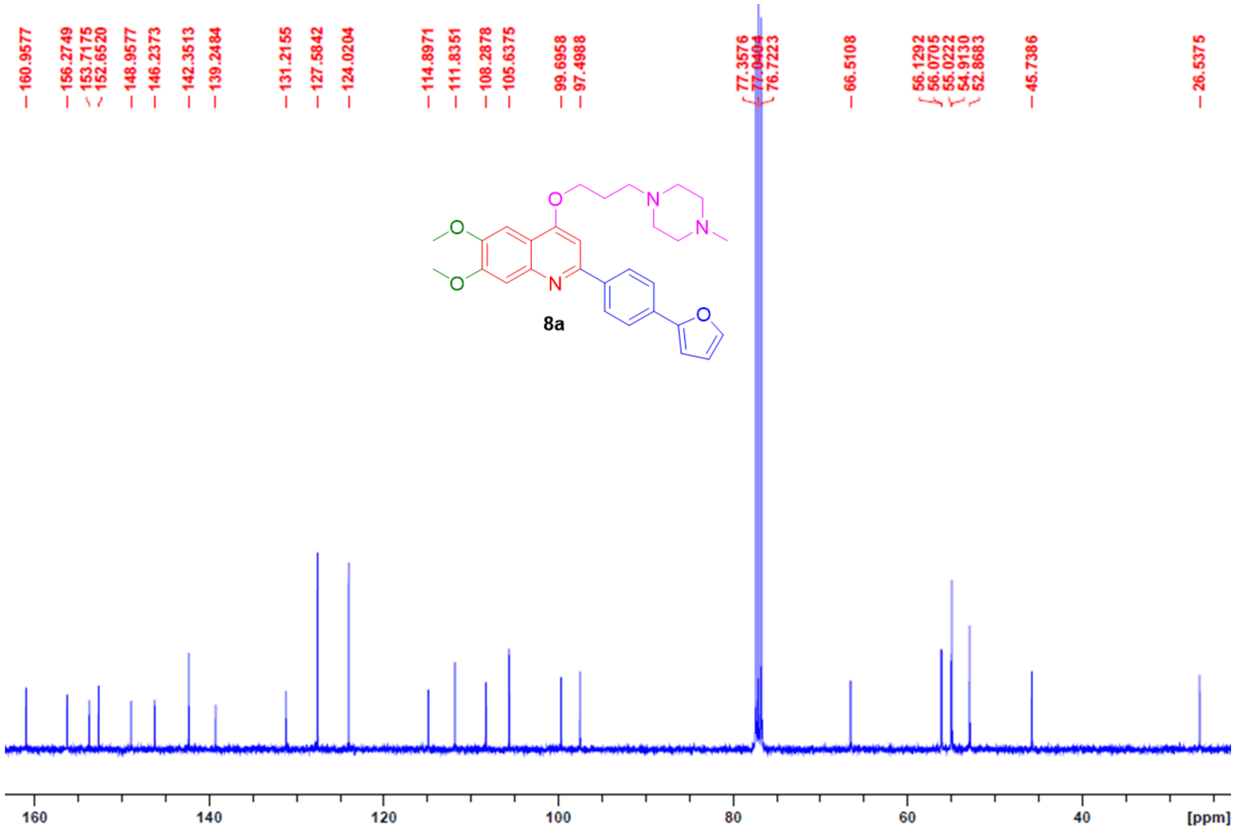

160.9577
156.2749
153.7175
152.6520
148.9577
146.2373
142.3513
139.2484
131.2155
127.5842
124.0204
114.8971
111.8351
108.2878
105.6375
99.6958
97.4988
77.3576
77.0404
76.7223
66.5108
56.1292
56.0705
55.0222
54.9130
52.8683
45.7386
26.5375
8a
[ppm]

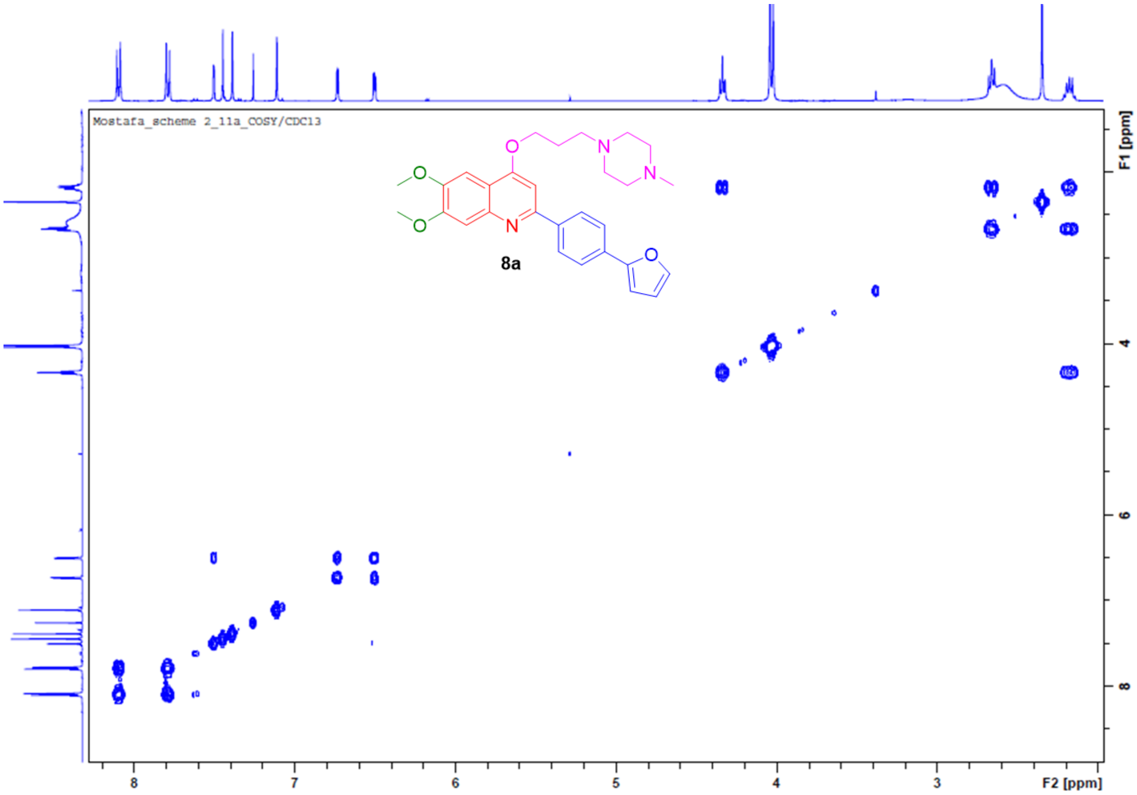

Mostafa_scheme 2_11a_COSY/CDCl3
8a
F1 [ppm]
F2 [ppm]

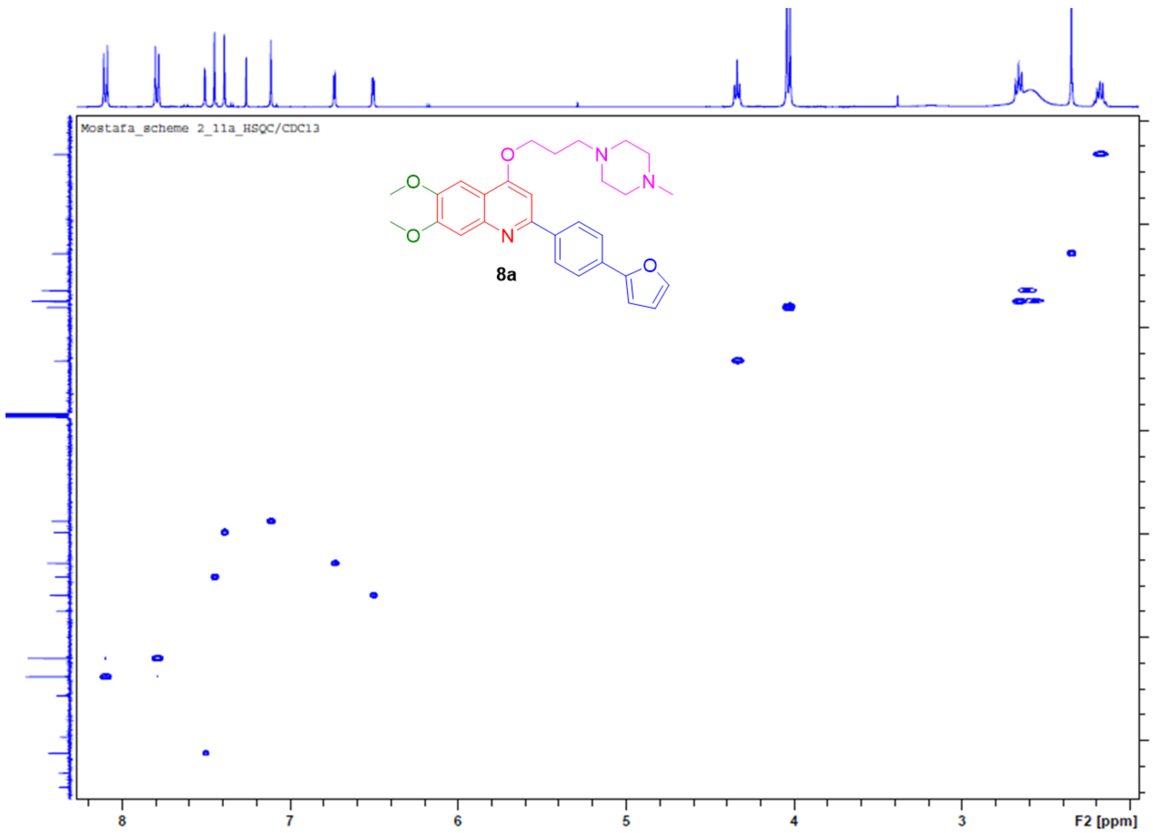
Mostafa_scheme 2_11a_HSQC/CDCl3
8a
F1 [ppm]
F2 [ppm]

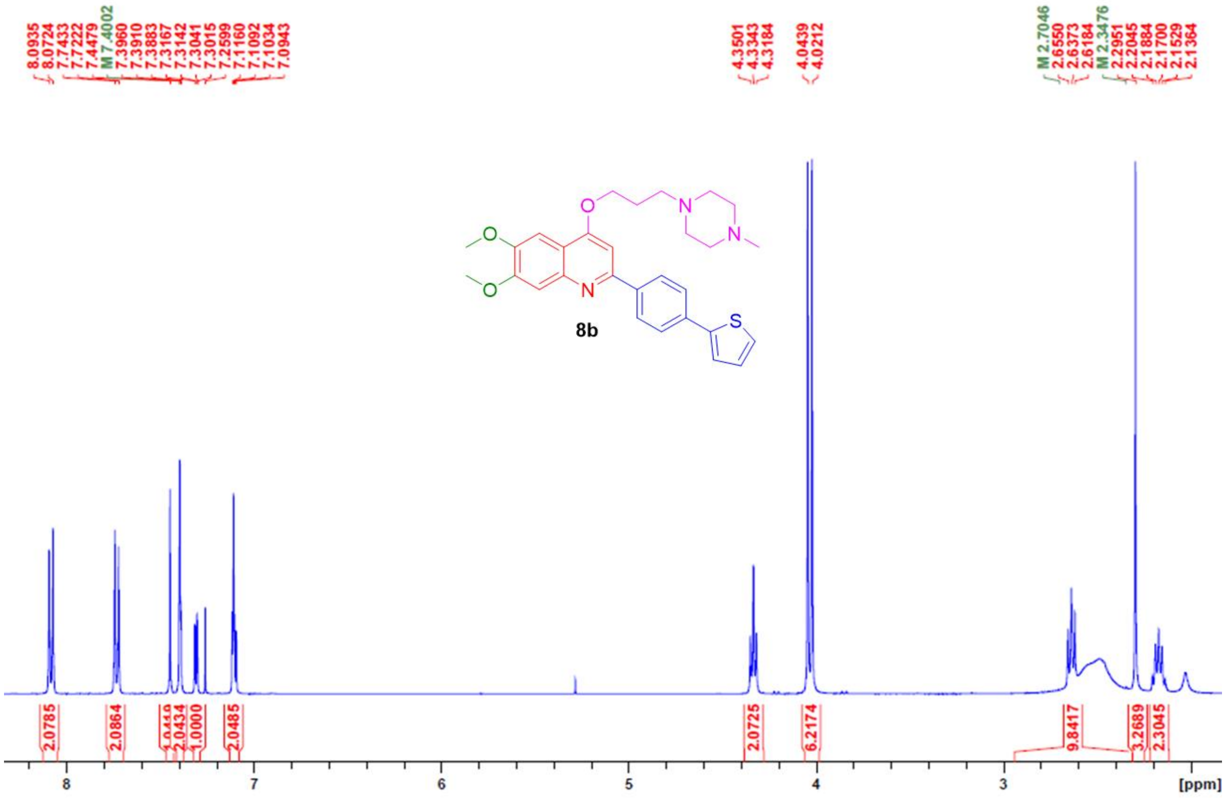
8b
[ppm]

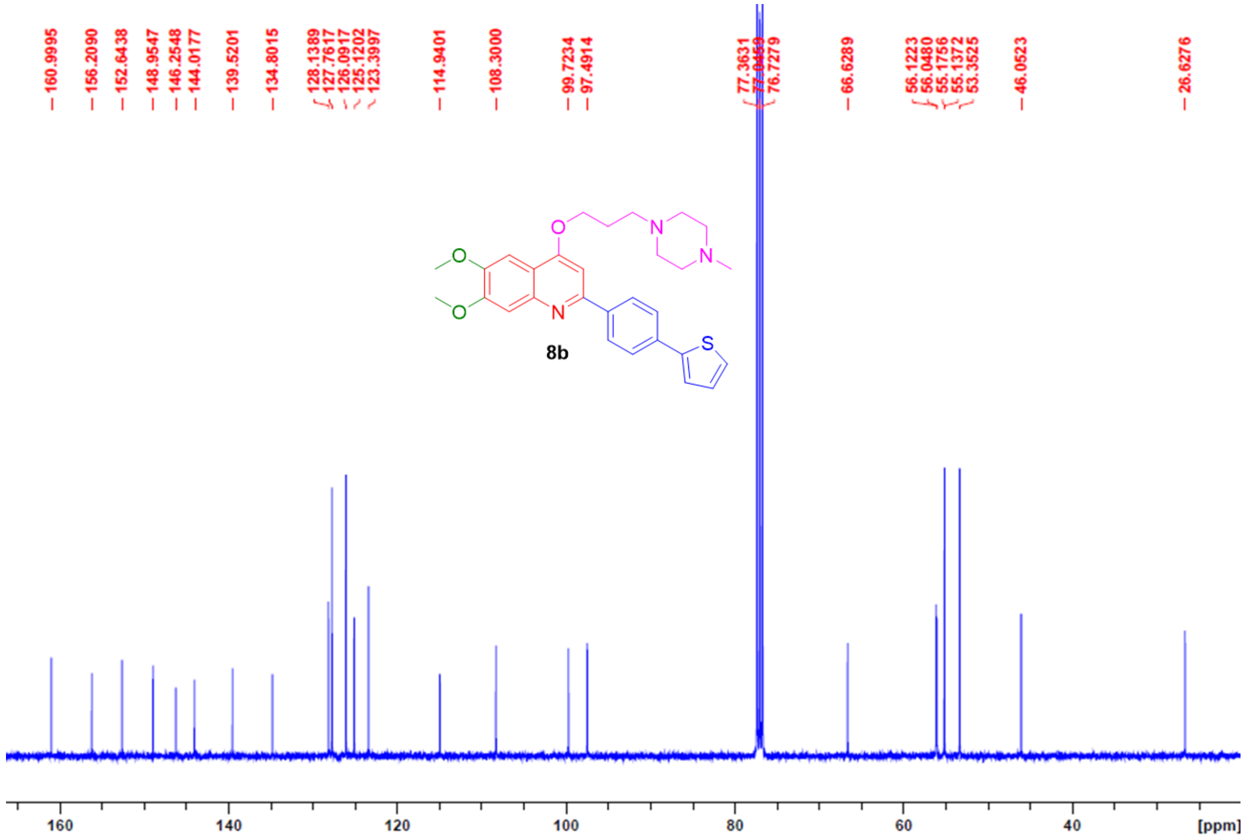
160.9995
156.2090
152.6438
148.9547
146.2548
144.0177
139.5201
134.8015
128.1389
127.7617
126.0917
125.1202
123.3997
114.9401
108.3000
99.7234
97.4914
77.3631
76.7279
66.6289
56.1223
56.0480
55.1756
55.1372
53.3525
46.0523
26.6276
8b
[ppm]

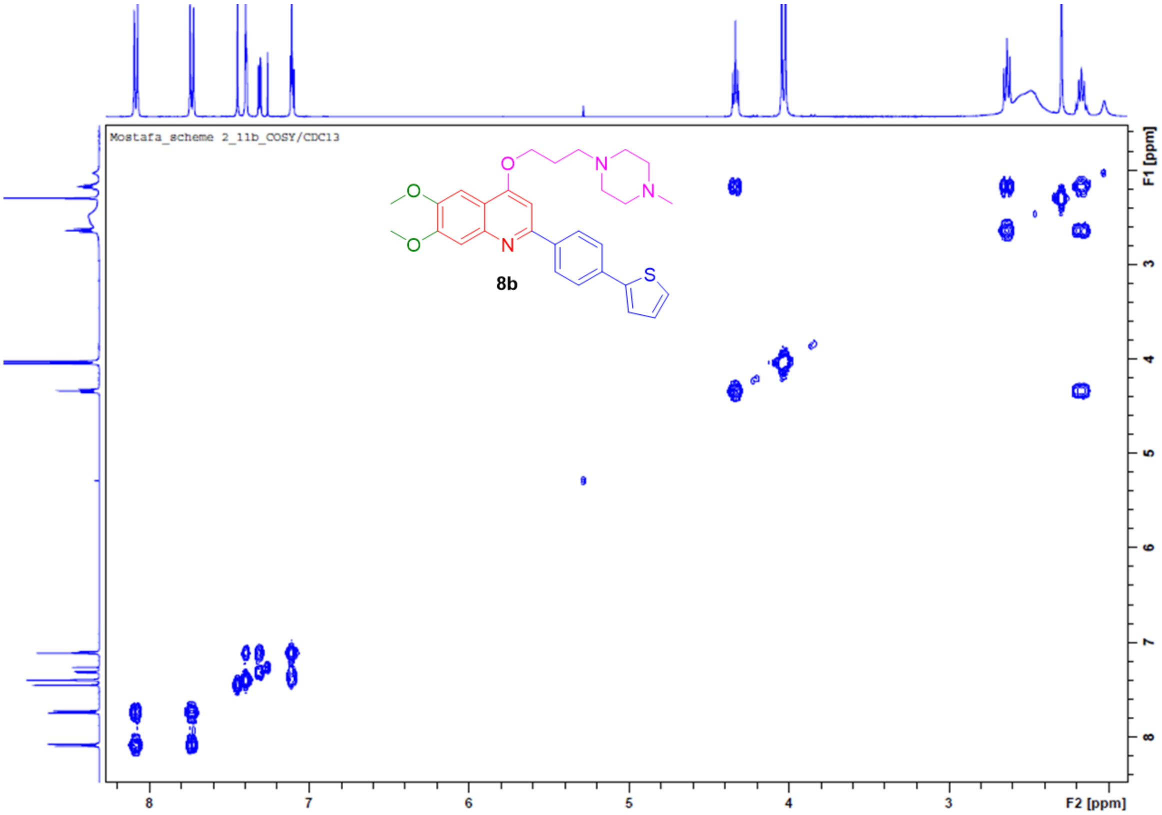
Mostafa_scheme 2_11b_COSY/CDCl3
8b
F1 [ppm]
F2 [ppm]

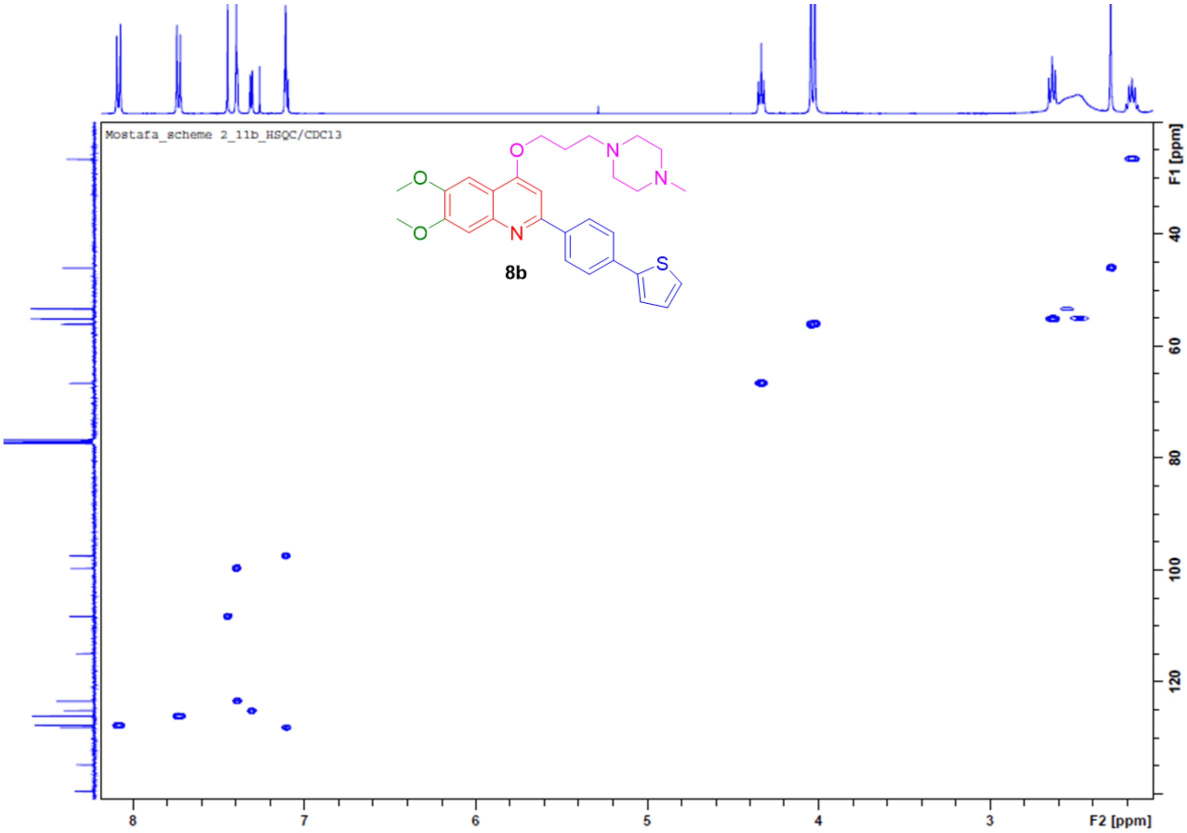
Mostafa_scheme 2_11b_HSQC/CDCl3
8b
F1 [ppm]
F2 [ppm]

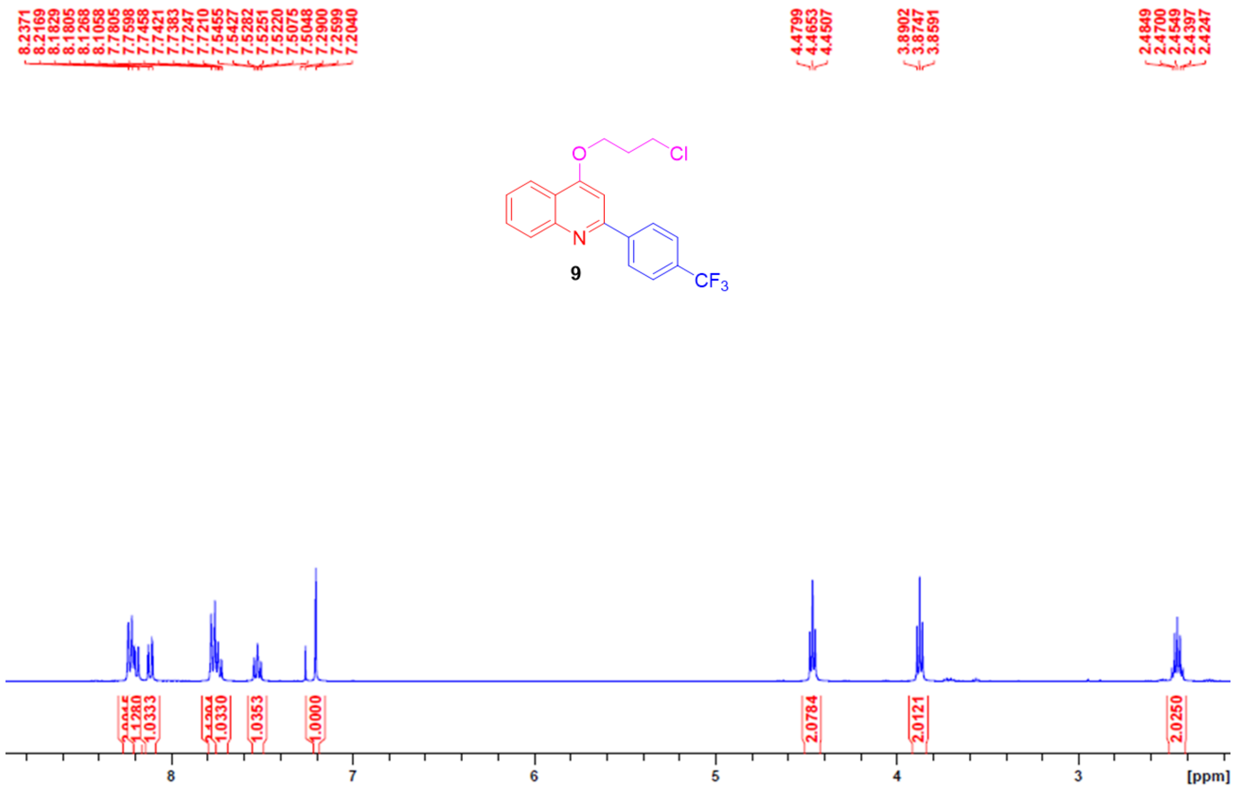
8.2371
8.2169
8.1829
8.1805
8.1268
8.1058
7.7805
7.7598
7.7458
7.7421
7.7383
7.7247
7.7210
7.5455
7.5427
7.5282
7.5251
7.5220
7.5075
7.5048
7.2900
7.2599
7.2040
4.4799
4.4653
4.4507
3.8902
3.8747
3.8591
2.4849
2.4700
2.4549
2.4397
2.4247
9
1.1280
1.0333
1.0330
1.0353
1.0000
2.0784
2.0121
2.0250
[ppm]

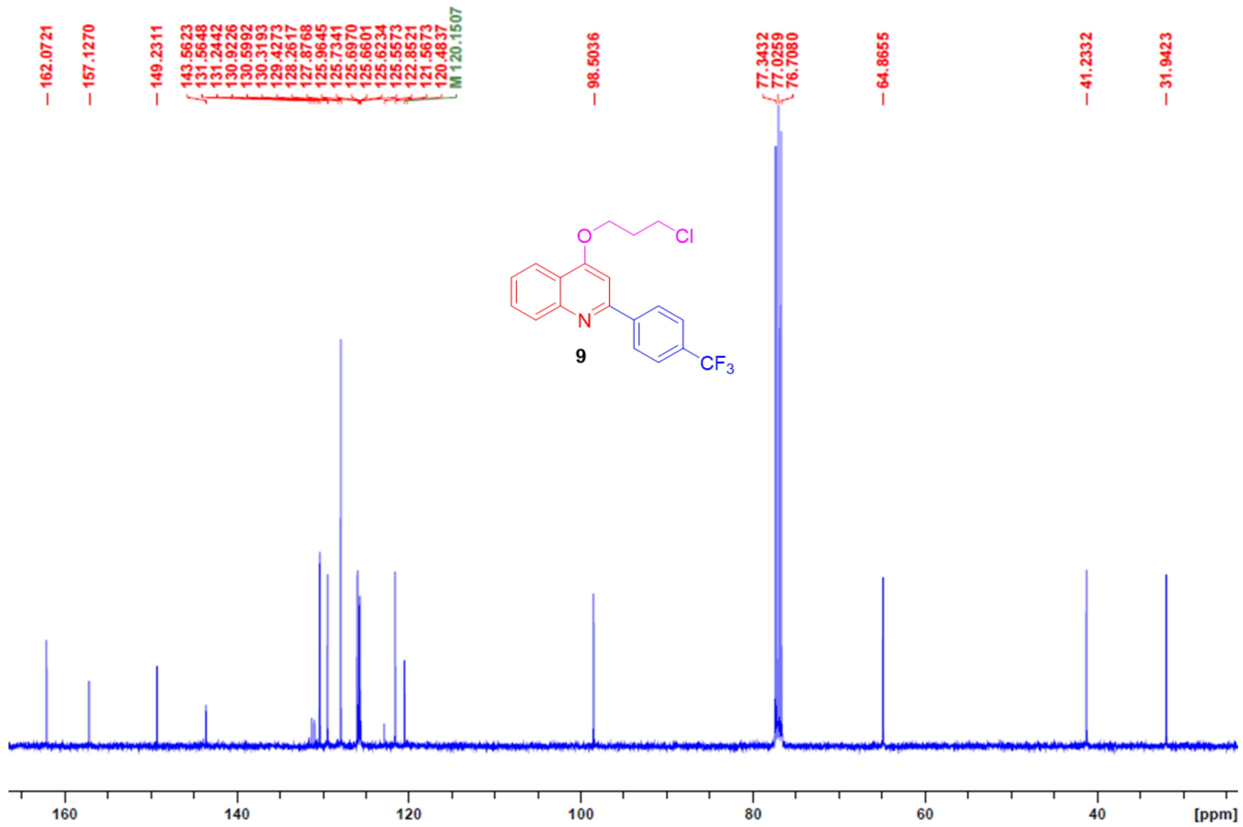
162.0721
157.1270
149.2311
143.5623
131.5648
131.2442
130.9226
130.5992
130.3193
129.4273
128.2617
127.8768
125.9645
125.7341
125.6970
125.6601
125.6234
125.5573
122.8521
121.5673
120.4837
M 120.1507
98.5036
77.3432
77.0259
76.7080
64.8655
41.2332
31.9423
O
Cl
N
9
CF3
160
140
120
100
80
60
40
[ppm]

_19F/CDCl3

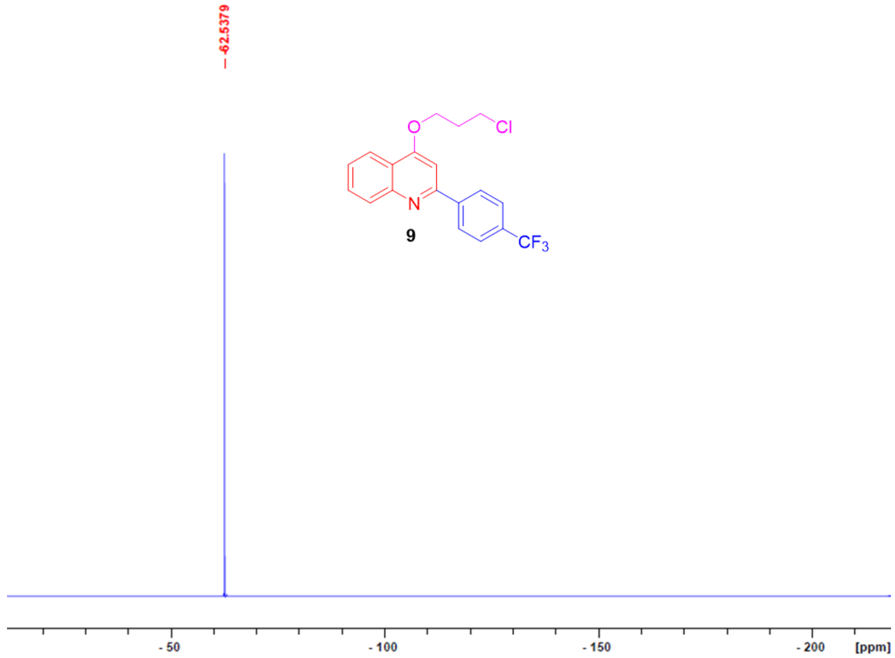
-62.5379
O
Cl
N
9
CF3
- 50
- 100
- 150
- 200
[ppm]

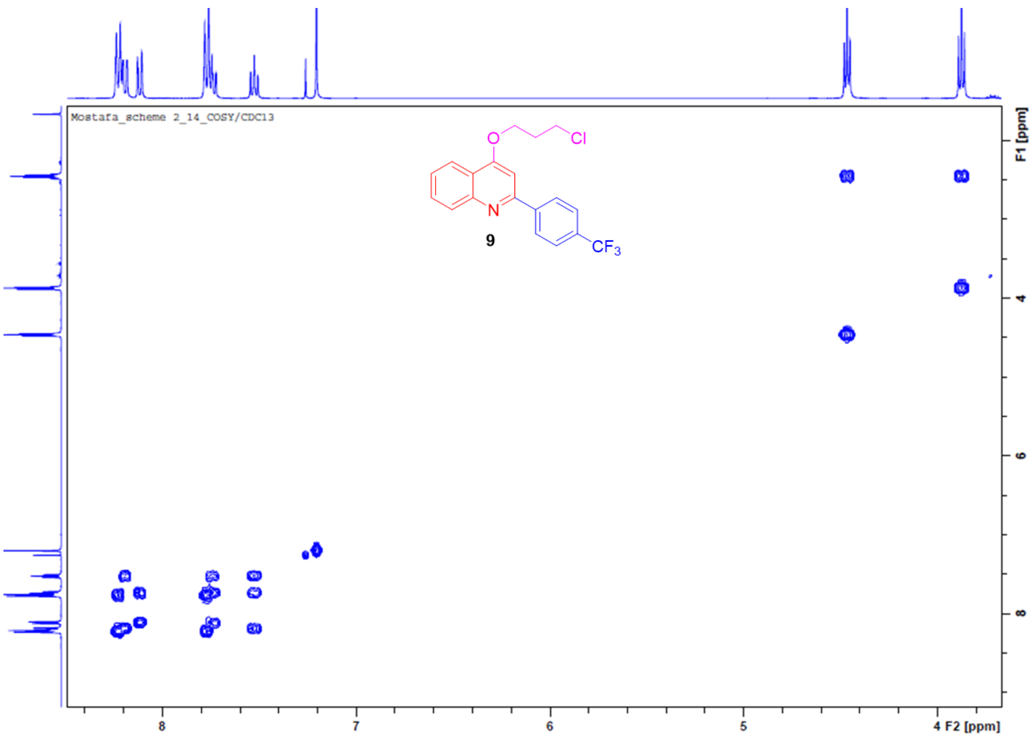
Mostafa_scheme 2_14_COSY/CDCl3
O
Cl
N
9
CF3
F1 [ppm]
4
6
8
8
7
6
5
4 F2 [ppm]

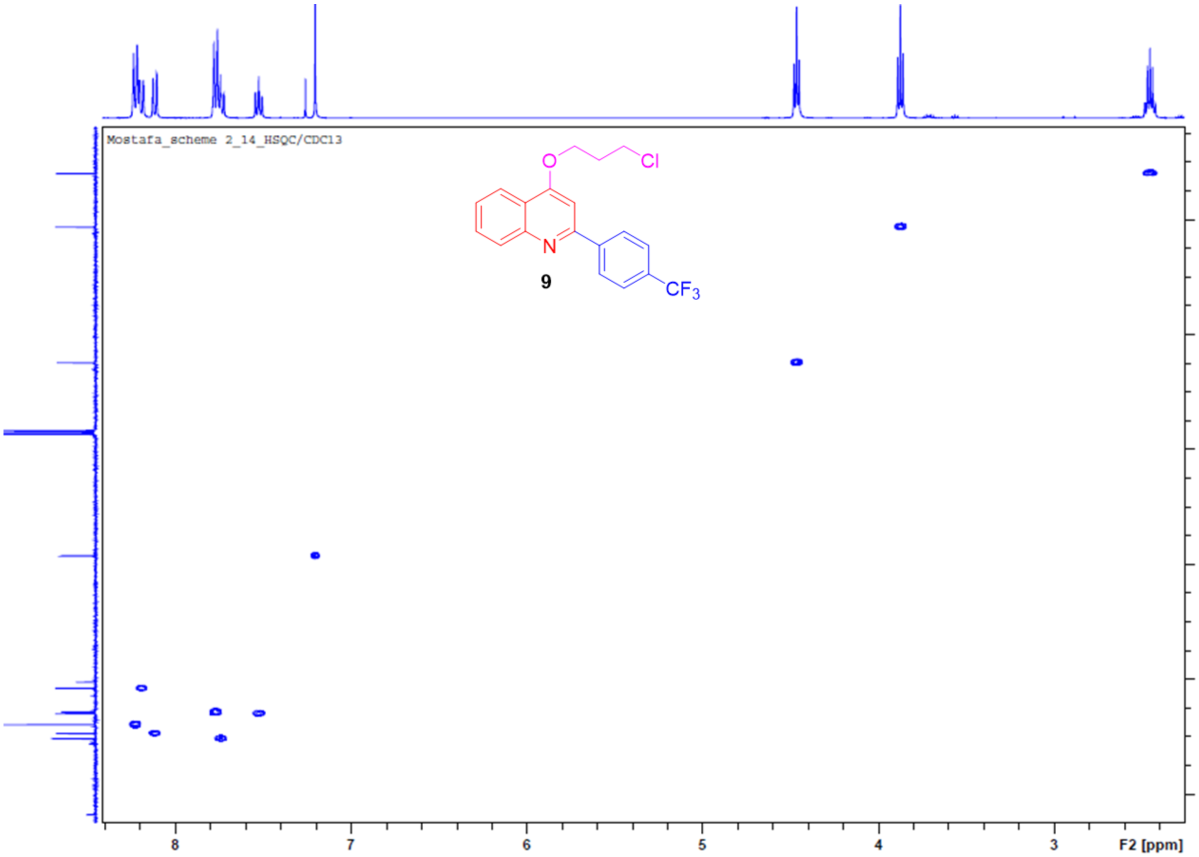
Mostafa_scheme 2_14_HSQC/CDCl3
O
Cl
N
9
CF3
F1 [ppm]
40
60
80
100
120
140
8
7
6
5
4
3
F2 [ppm]

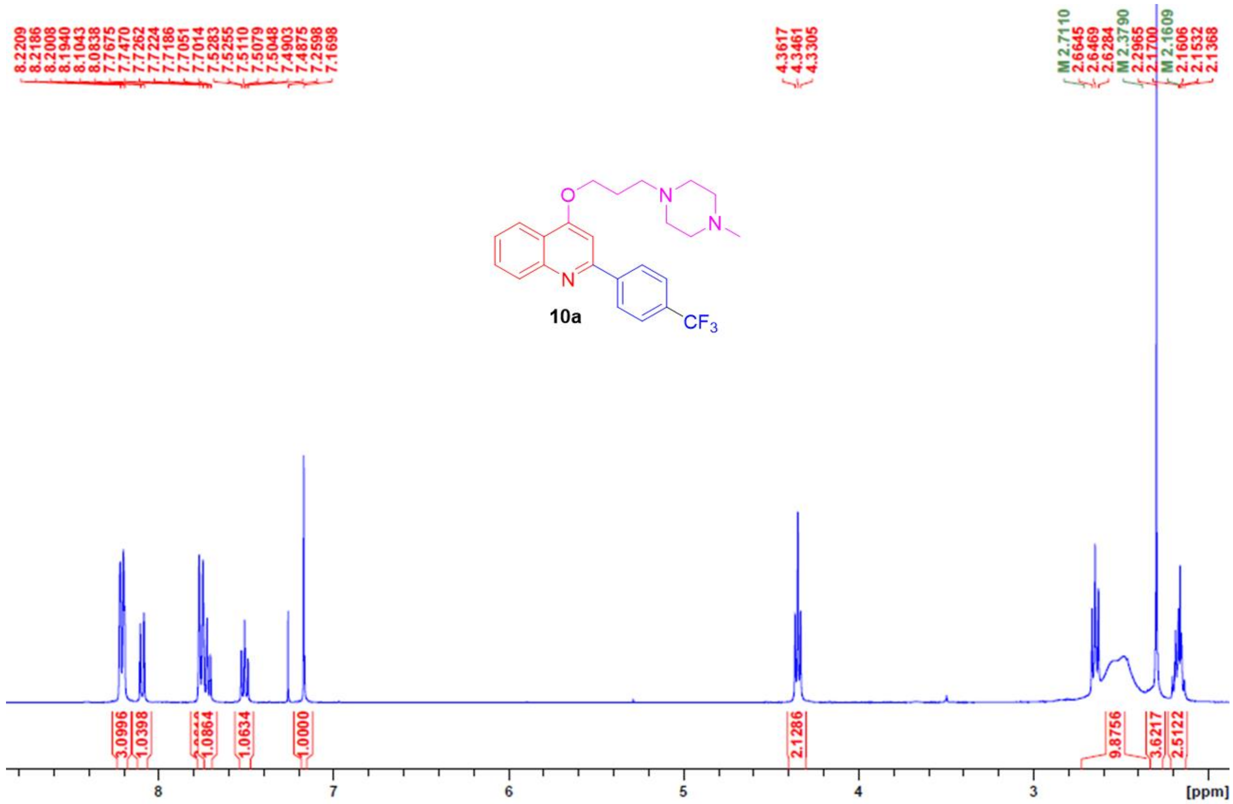

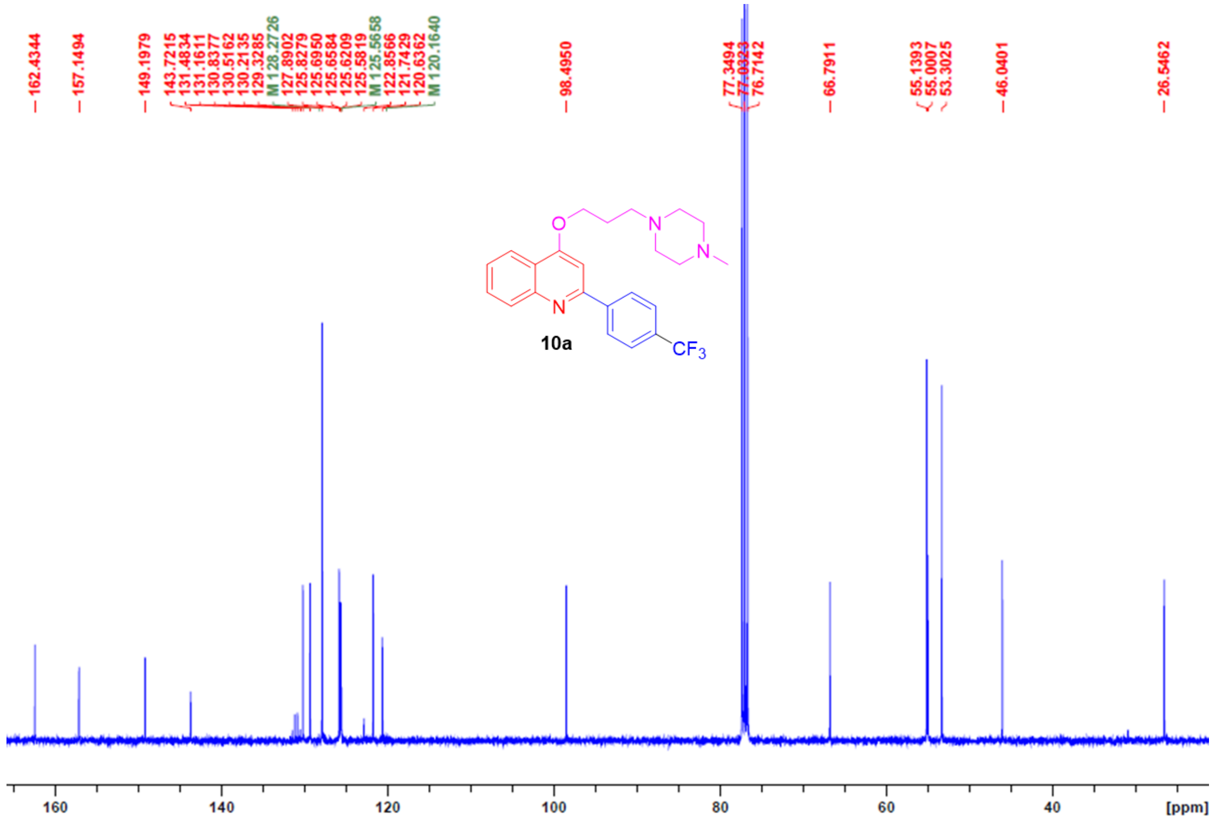

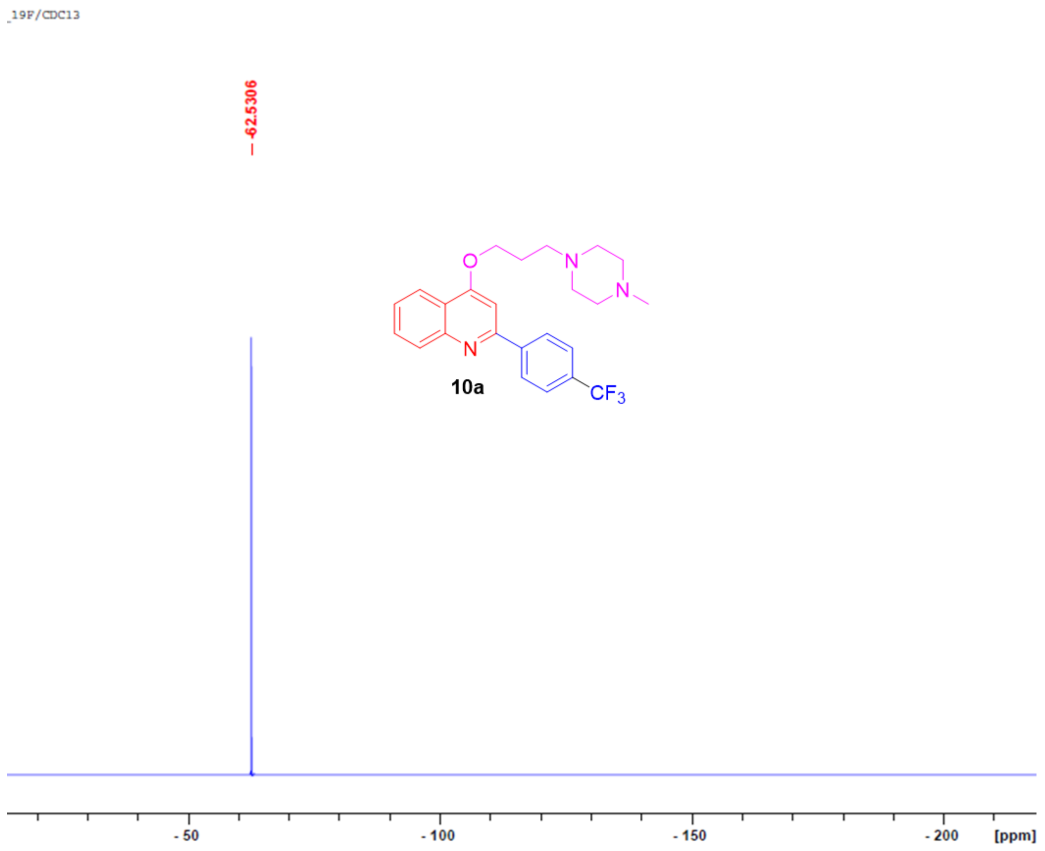
_19F/CDCl3
-62.5306
O
N
N
N
10a
CF3
- 50
- 100
- 150
- 200
[ppm]

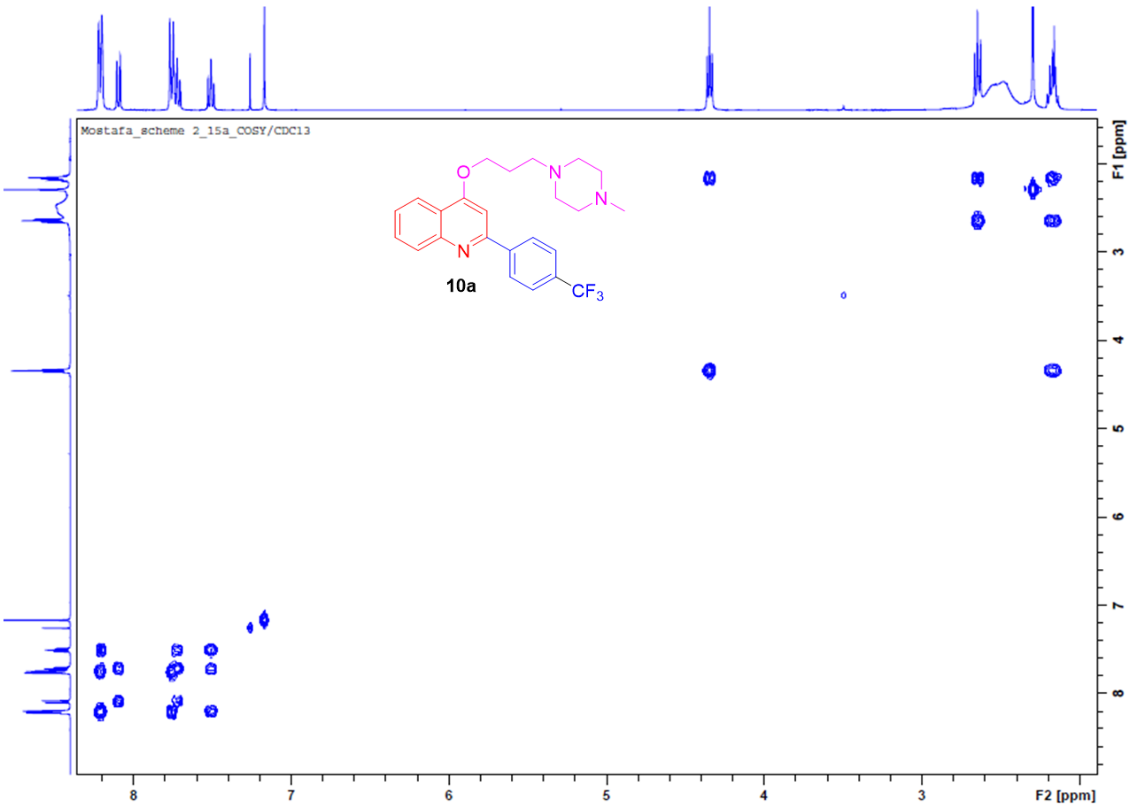
Mostafa_scheme 2_15a_COSY/CDCl3
O
N
N
N
10a
CF3
F1 [ppm]
3
4
5
6
7
8
8
7
6
5
4
3
F2 [ppm]

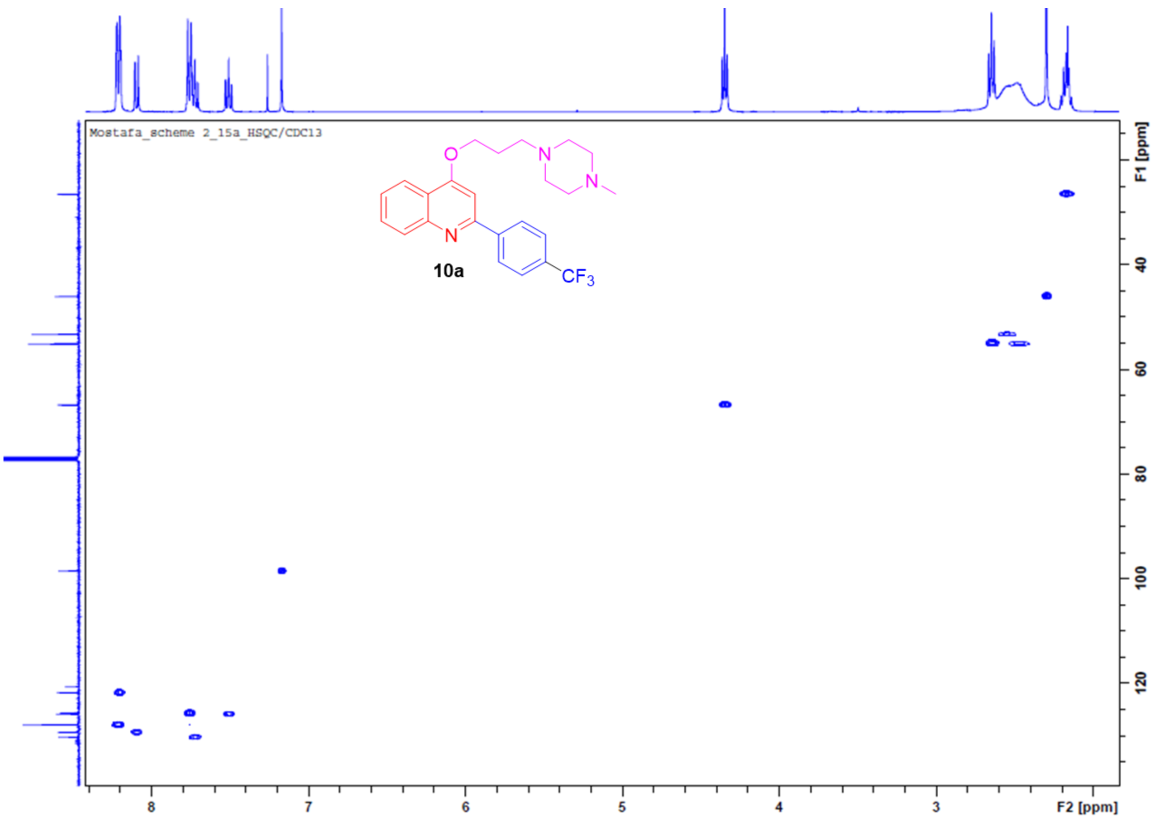

Mostafa_scheme 2_15a_HSQC/CDCl3
10a
CF3
F1 [ppm]
F2 [ppm]

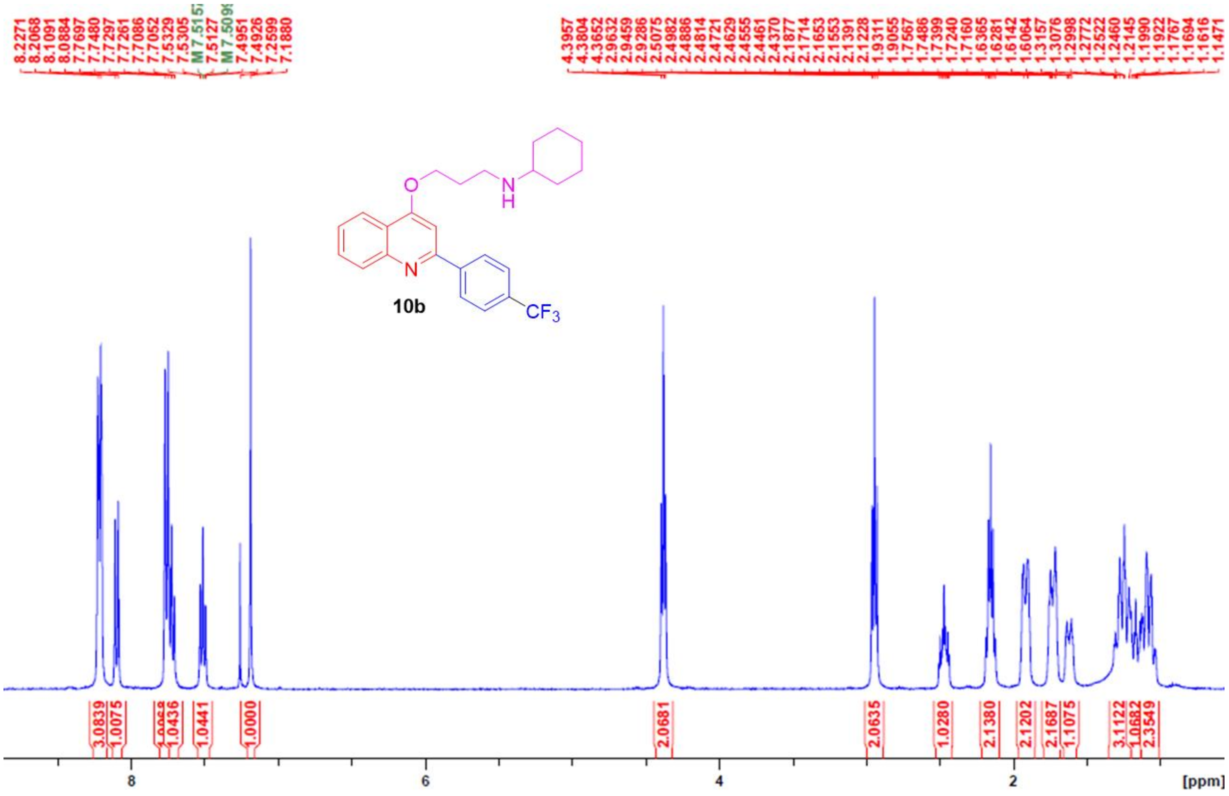

10b
CF3
3.0839
1.0075
1.0436
1.0441
1.0000
2.0681
2.0635
1.0280
2.1380
2.1202
2.1687
1.1075
3.1122
1.0682
2.3549
[ppm]

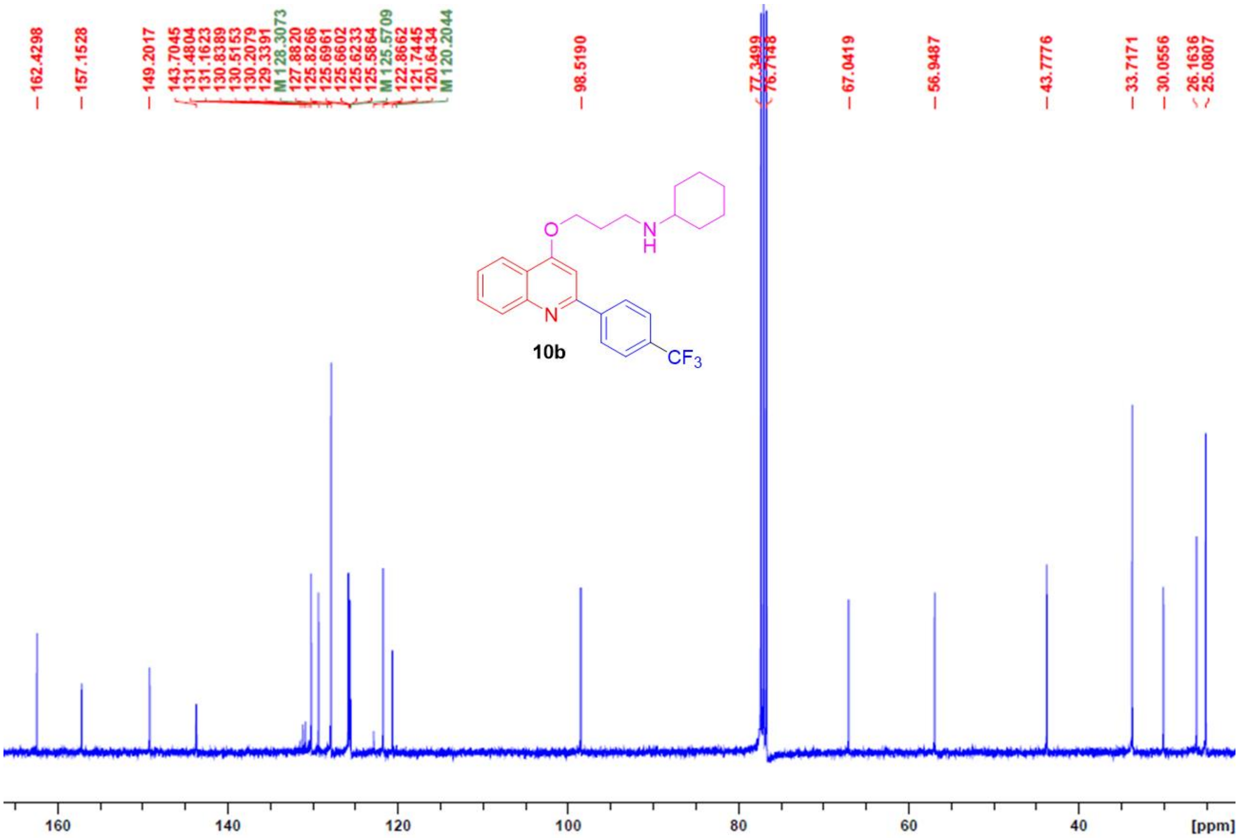
162.4298
157.1528
149.2017
143.7045
131.4804
131.1623
130.8389
130.5153
130.2079
129.3391
M 128.3073
127.8820
125.8266
125.6961
125.6602
125.6233
125.5864
M 125.5709
122.8662
121.7445
120.6434
M 120.2044
98.5190
77.3499
76.7148
67.0419
56.9487
43.7776
33.7171
30.0556
26.1636
25.0807
10b
[ppm]

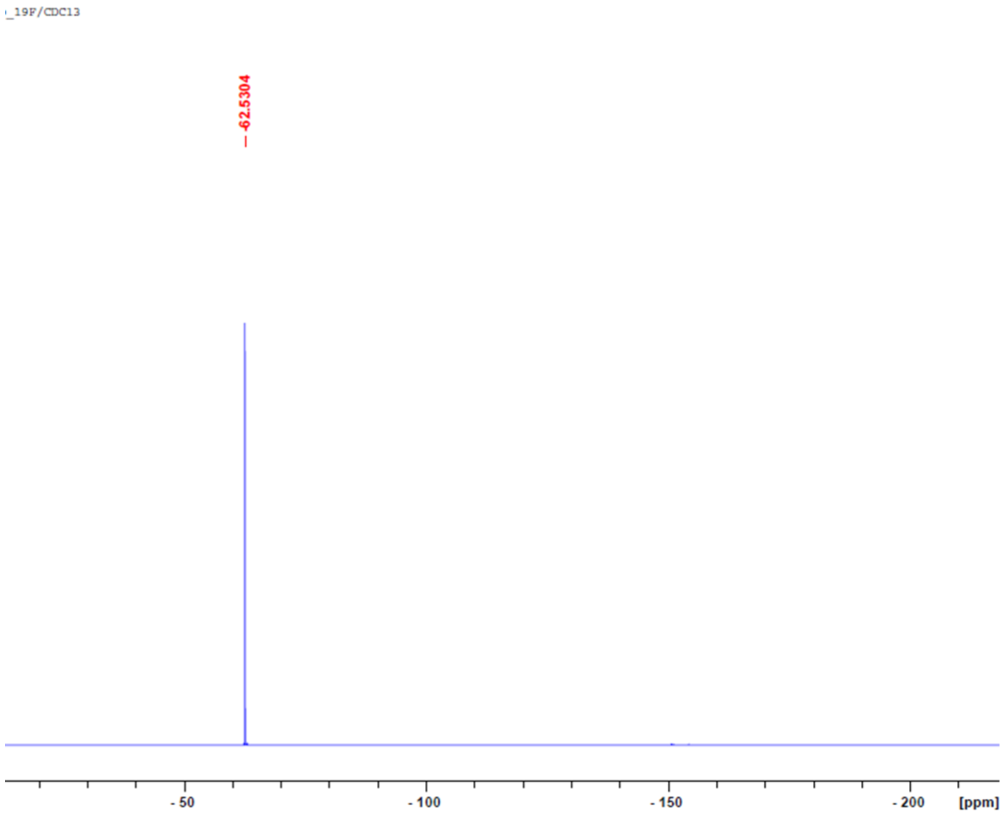
_19F/CDCl3
-62.5304
[ppm]

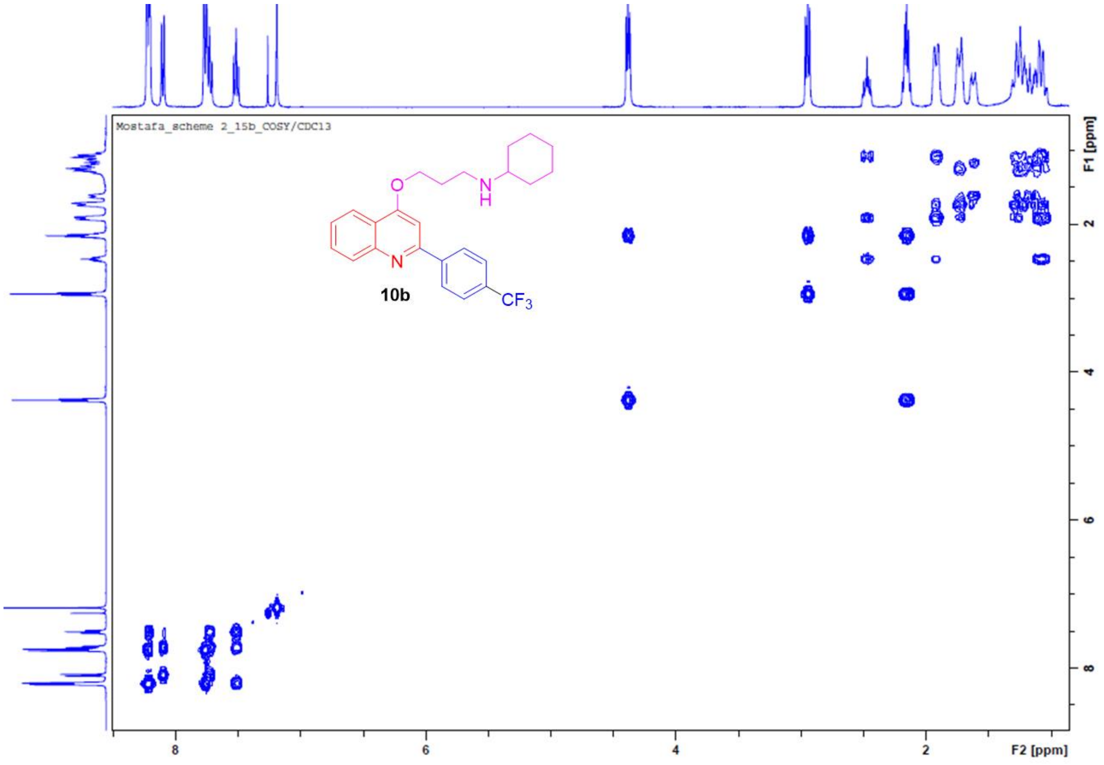
Mostafa_scheme 2_15b_COSY/CDCl3
10b
CF3
F1 [ppm]
F2 [ppm]

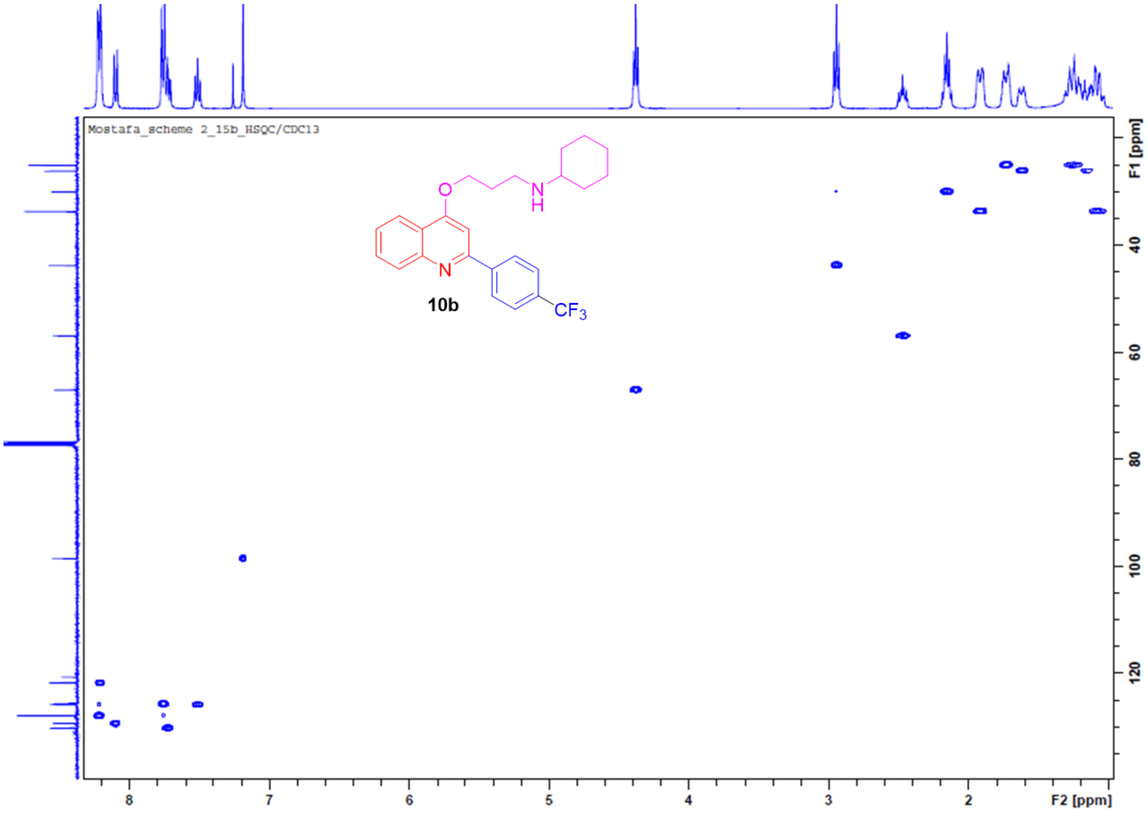
Mostafa_scheme 2_15b_HSQC/CDCl3
10b
CF3
F1 [ppm]
F2 [ppm]

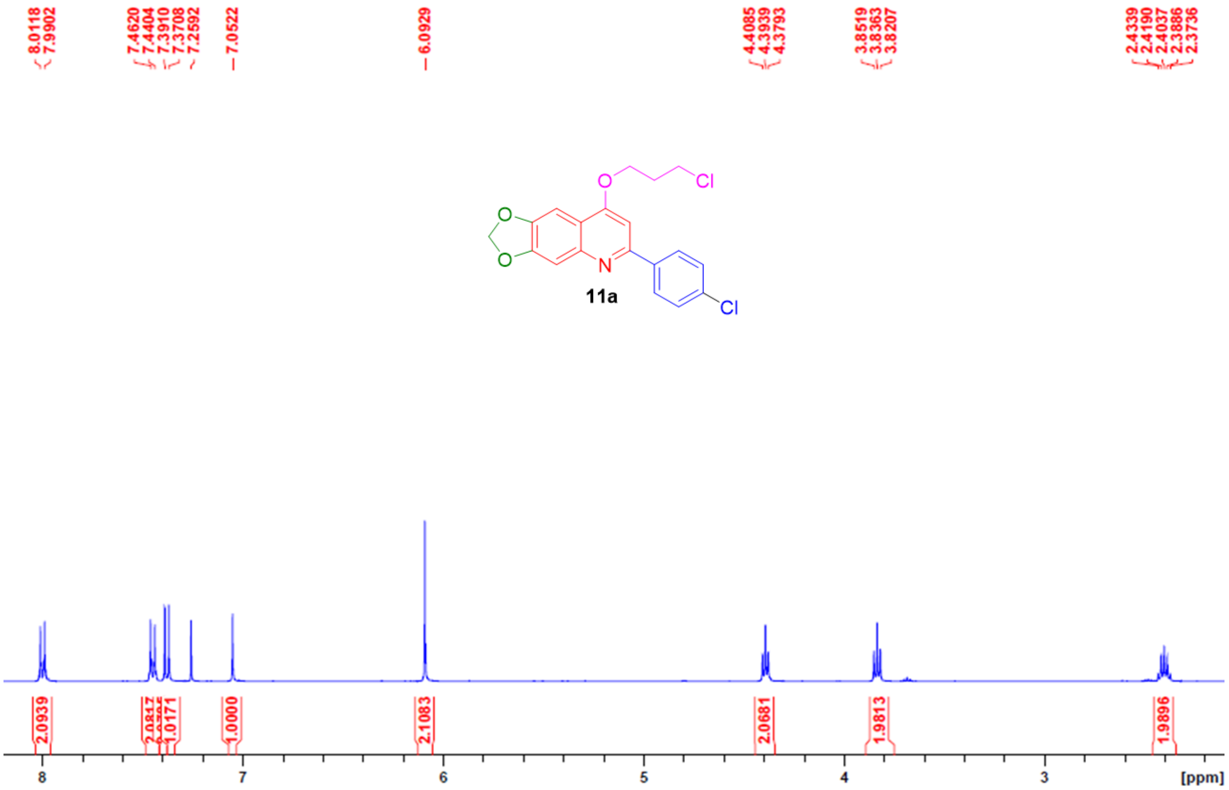
8.0118
7.9902
7.4620
7.4404
7.3910
7.3708
7.2592
7.0522
6.0929
4.4085
4.3939
4.3793
3.8519
3.8363
3.8207
2.4339
2.4190
2.4037
2.3886
2.3736
O
Cl
O
O
N
11a
Cl
2.0939
1.0171
1.0000
2.1083
2.0681
1.9813
1.9896
[ppm]

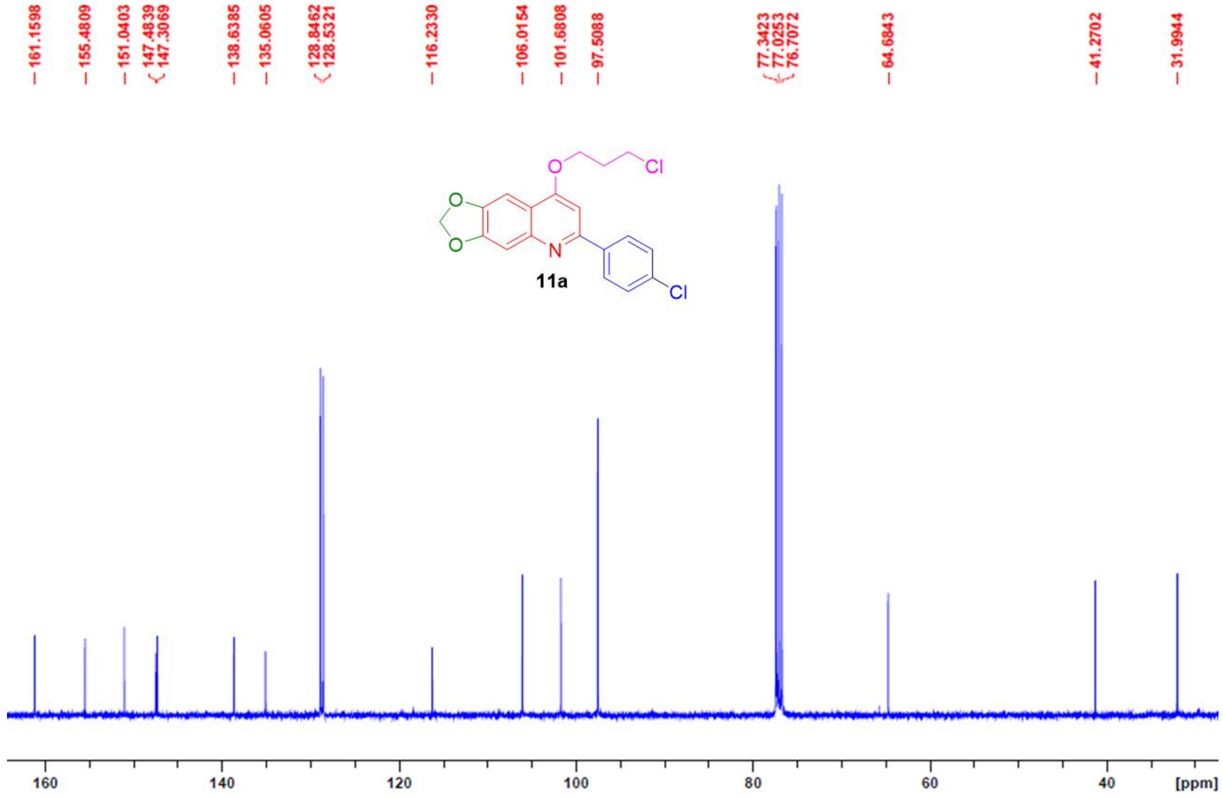
161.1598
155.4809
151.0403
147.4839
147.3069
138.6385
135.0605
128.8462
128.5321
116.2330
106.0154
101.6808
97.5088
77.3423
77.0253
76.7072
64.6843
41.2702
31.9944
O
Cl
O
O
N
11a
Cl
[ppm]

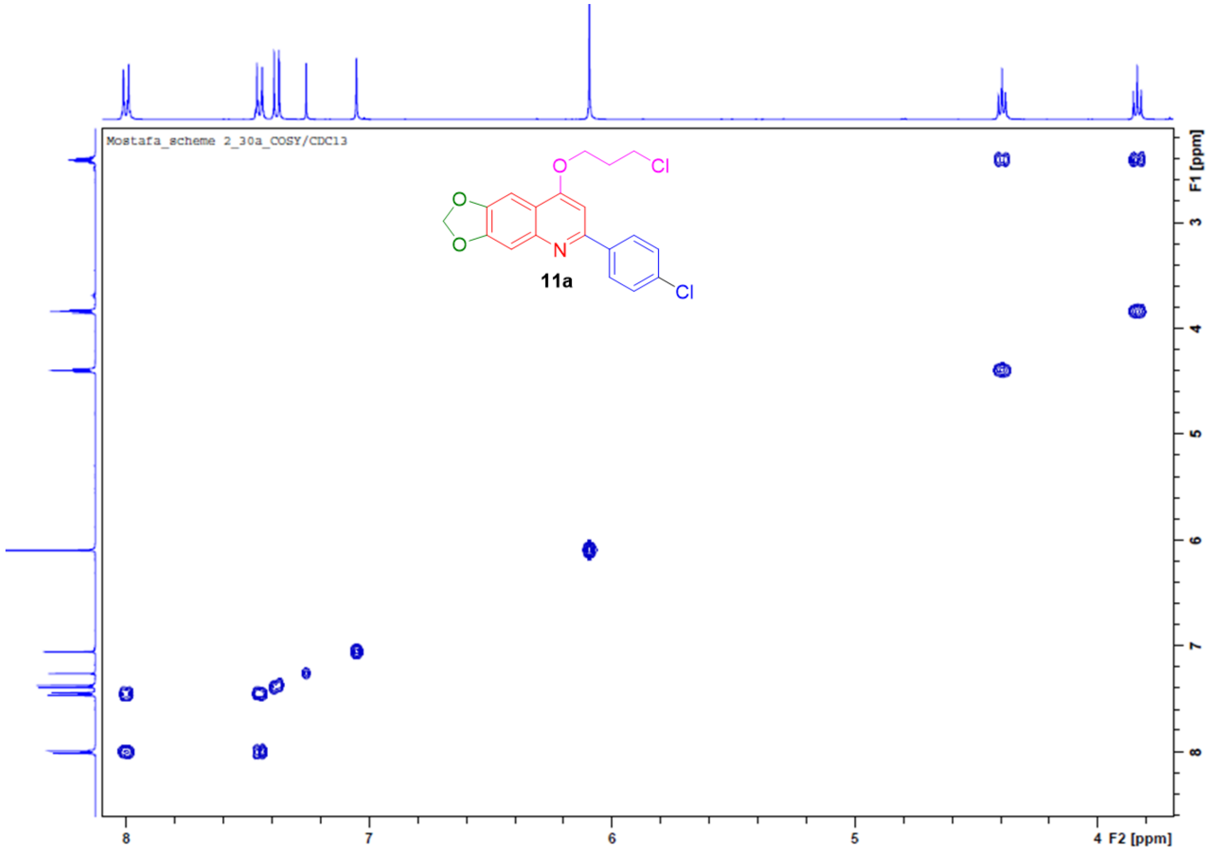
Mostafa_scheme 2_30a_COSY/CDCl3
O
Cl
O
O
N
11a
Cl
F1 [ppm]
3
4
5
6
7
8
8
7
6
5
4 F2 [ppm]

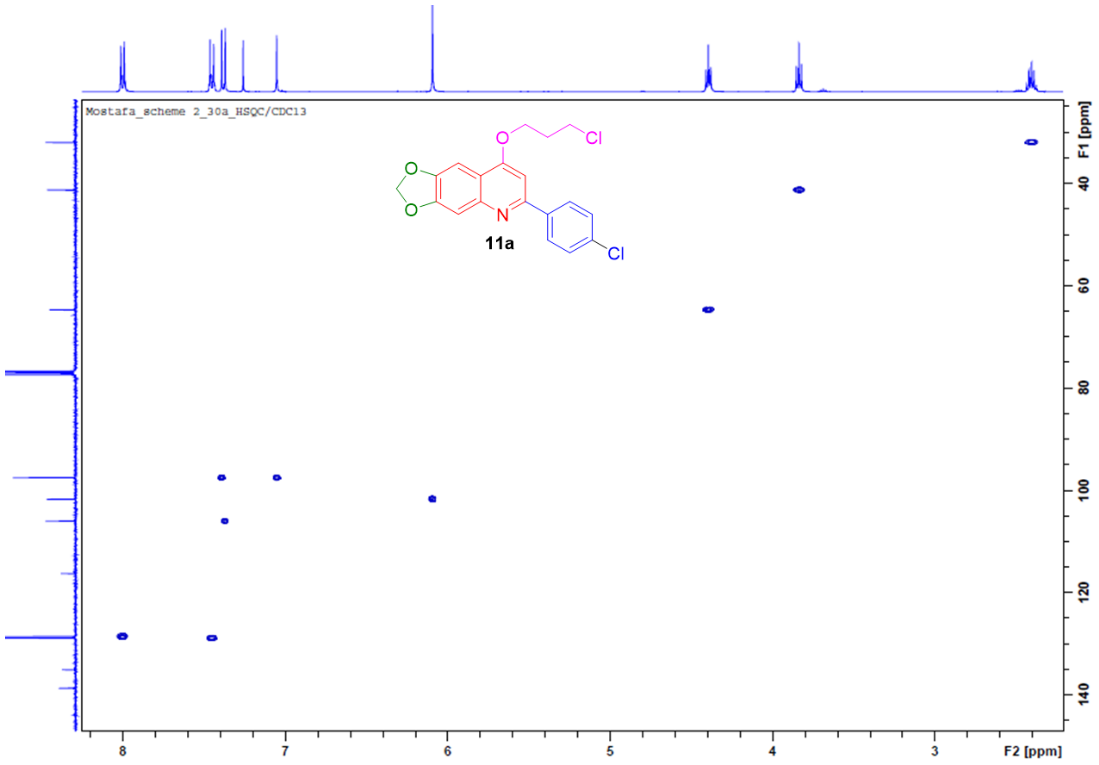
Mostafa_scheme 2_30a_HSQC/CDCl3
O
Cl
O
O
N
11a
Cl
F1 [ppm]
40
60
80
100
120
140
8
7
6
5
4
3
F2 [ppm]

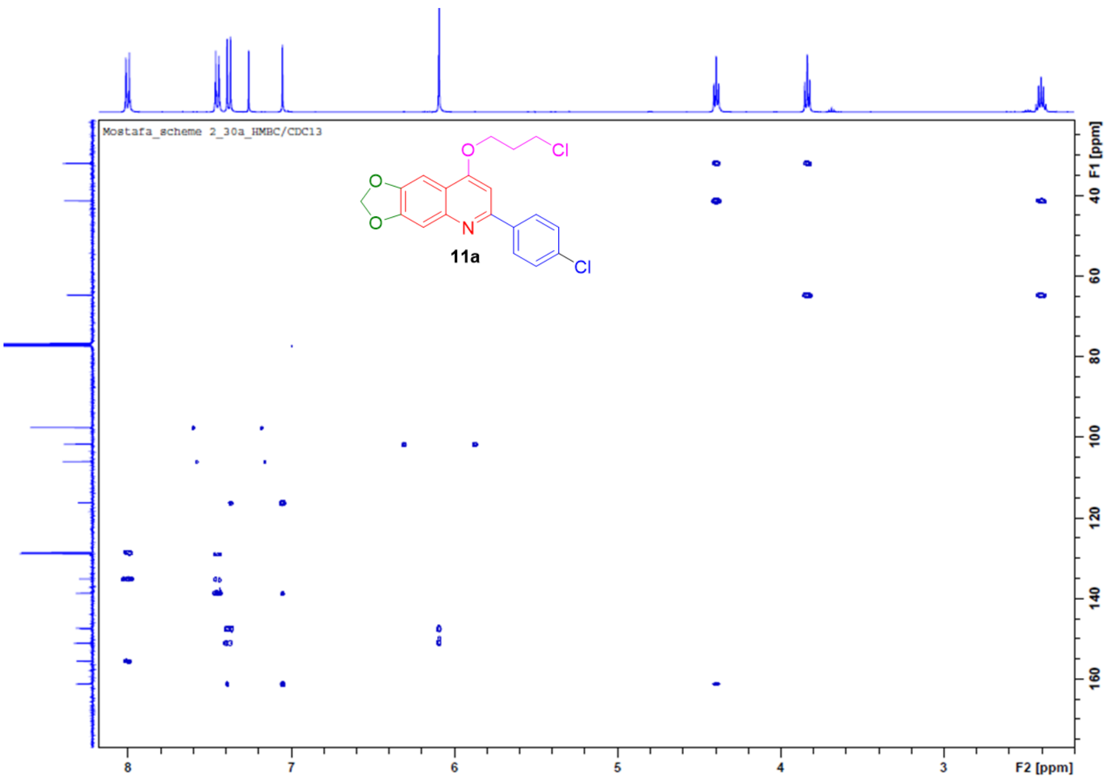
Mostafa_scheme 2_30a_HMBC/CDCl3
O
Cl
O
O
N
11a
Cl
F1 [ppm]
F2 [ppm]

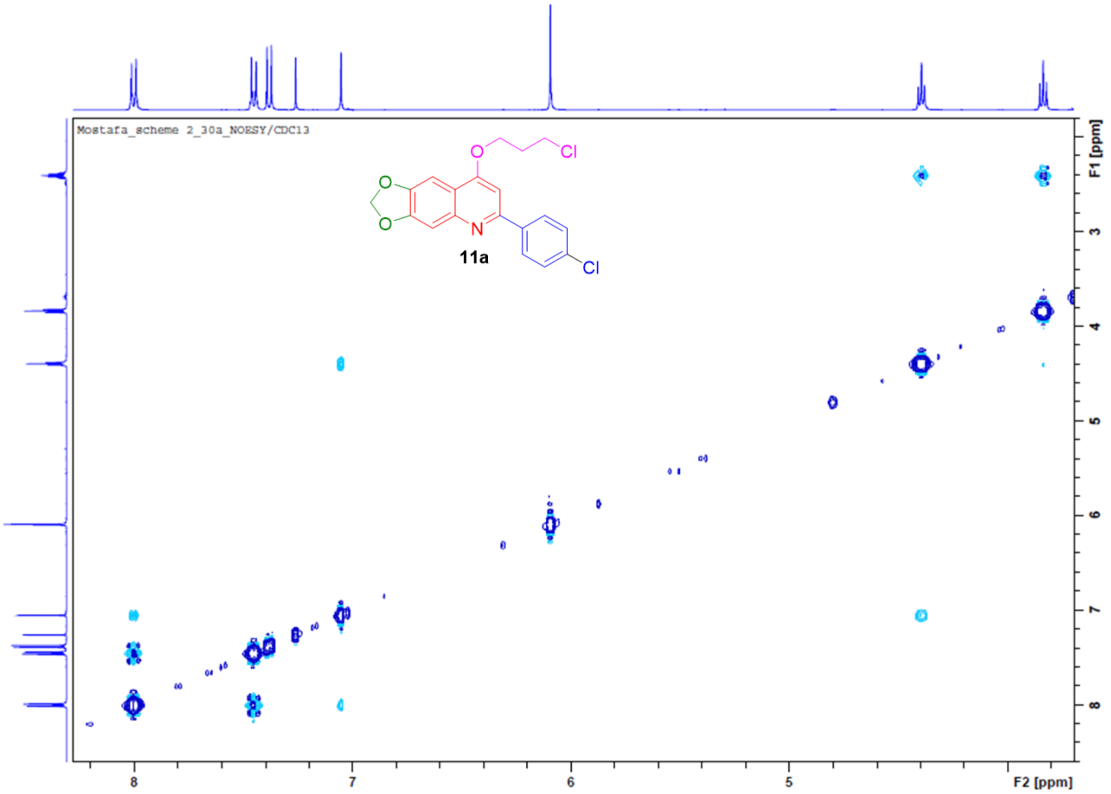
Mostafa_scheme 2_30a_NOESY/CDCl3
O
Cl
O
O
N
11a
Cl
F1 [ppm]
F2 [ppm]

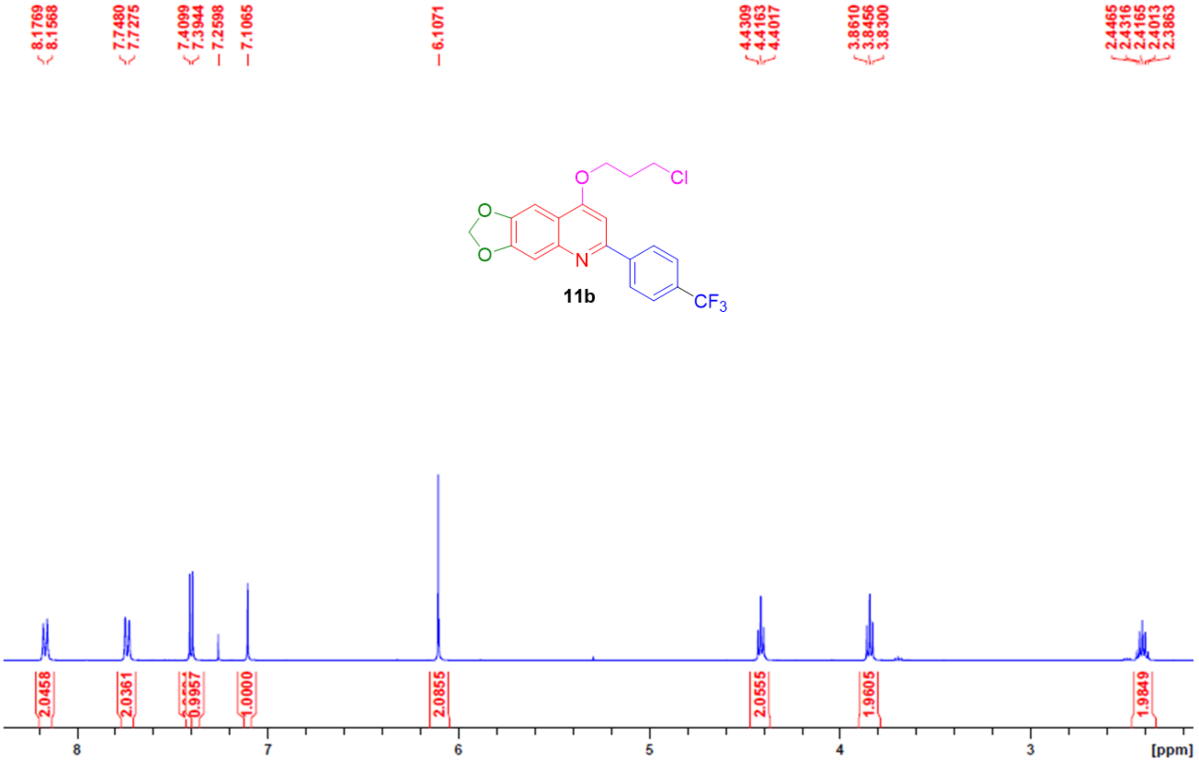
8.1769
8.1568
7.7480
7.7275
7.4099
7.3944
7.2598
7.1065
6.1071
4.4309
4.4163
4.4017
3.8610
3.8456
3.8300
2.4465
2.4316
2.4165
2.4013
2.3863
O
Cl
N
CF3
11b
2.0458
2.0361
[illegible]
0.9957
1.0000
2.0855
2.0555
1.9605
1.9849
8
7
6
5
4
3
[ppm]

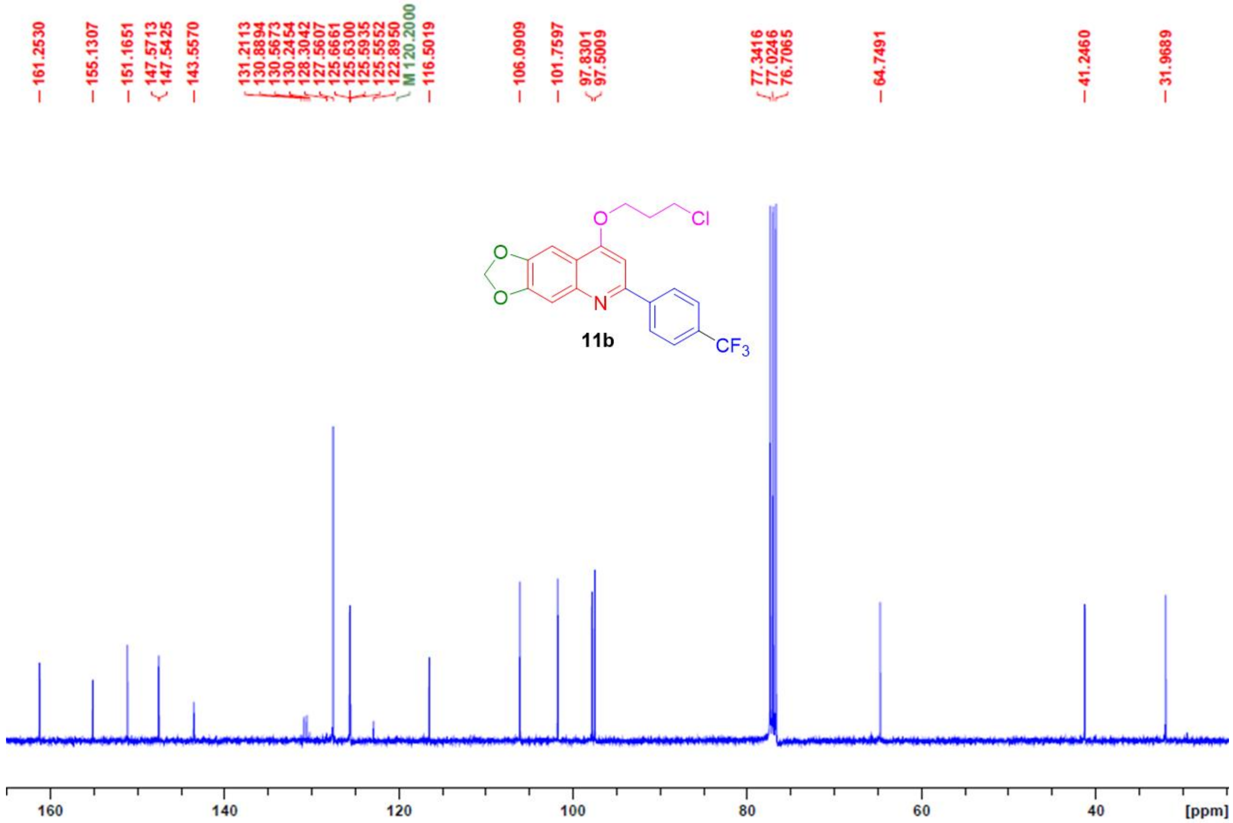
161.2530
155.1307
151.1651
147.5713
147.5425
143.5570
131.2113
130.8894
130.5673
130.2454
128.3042
127.5607
125.6661
125.6300
125.5935
125.5552
122.8950
M1 120.2000
116.5019
106.0909
101.7597
97.8301
97.5009
77.3416
77.0246
76.7065
64.7491
41.2460
31.9689
O
Cl
N
CF3
11b
160
140
120
100
80
60
40
[ppm]

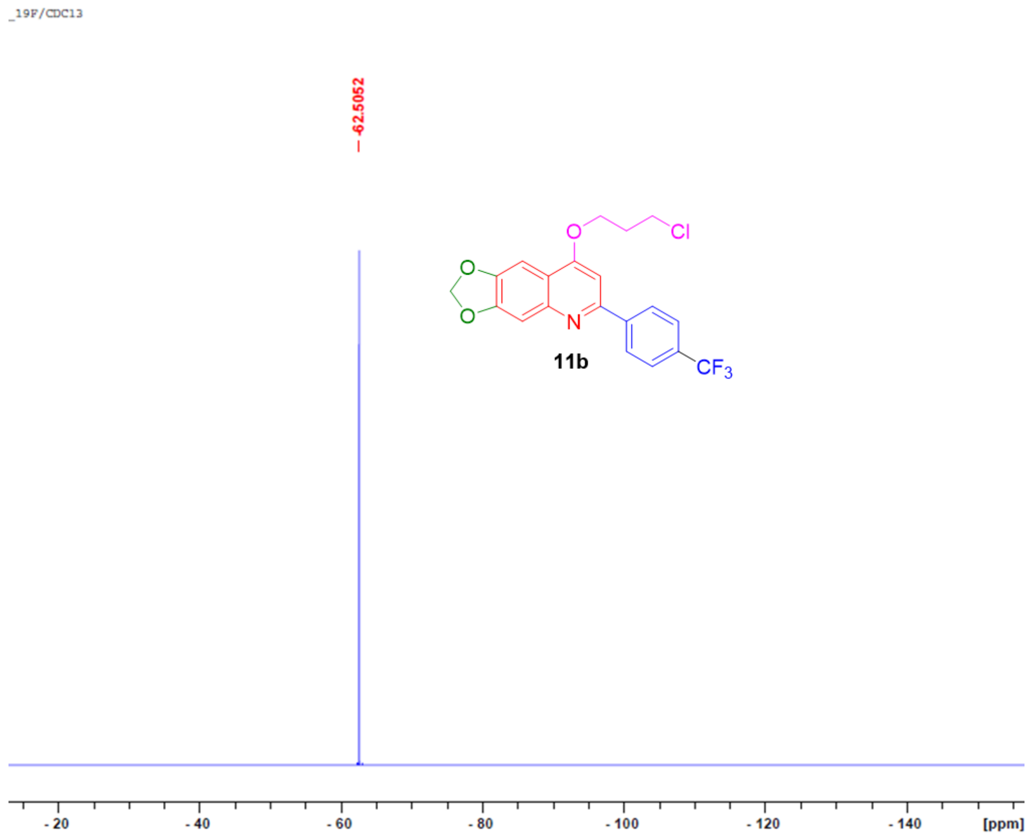
_19F/CDCl3
-62.5052
O
Cl
O
O
N
11b
CF3
-20
-40
-60
-80
-100
-120
-140
[ppm]

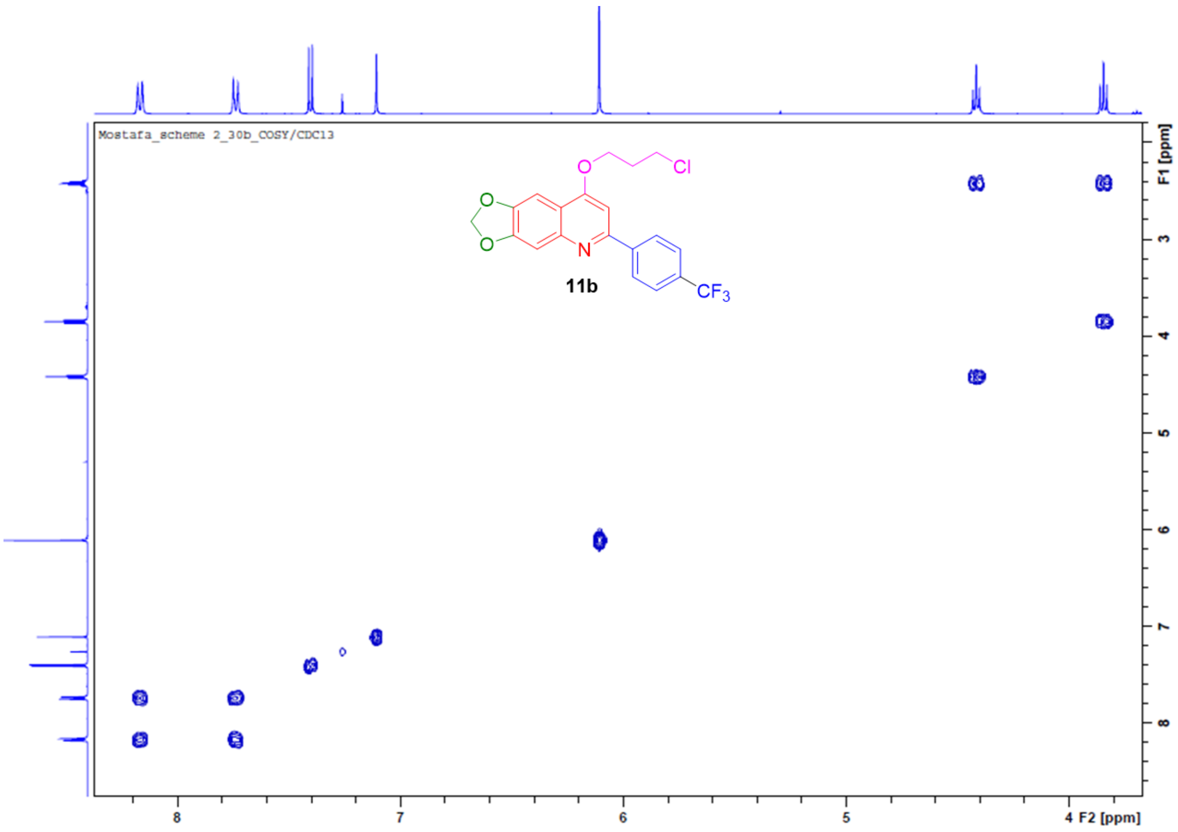
Mostafa_scheme 2_30b_COSY/CDCl3
O
Cl
O
O
N
11b
CF3
F1 [ppm]
3
4
5
6
7
8
8
7
6
5
4 F2 [ppm]

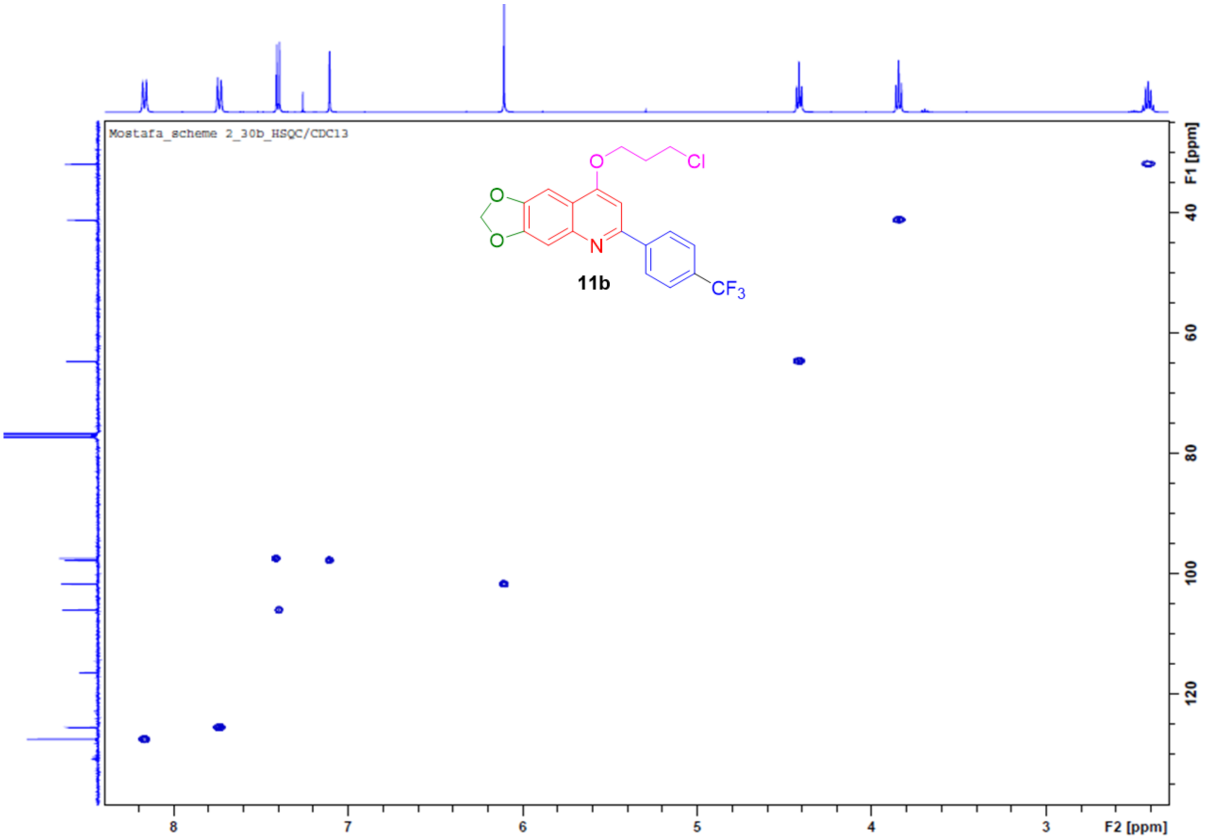
Mostafa_scheme 2_30b_HSQC/CDCl3
O
Cl
O
O
N
11b
CF3
F1 [ppm]
40
60
80
100
120
8
7
6
5
4
3
F2 [ppm]

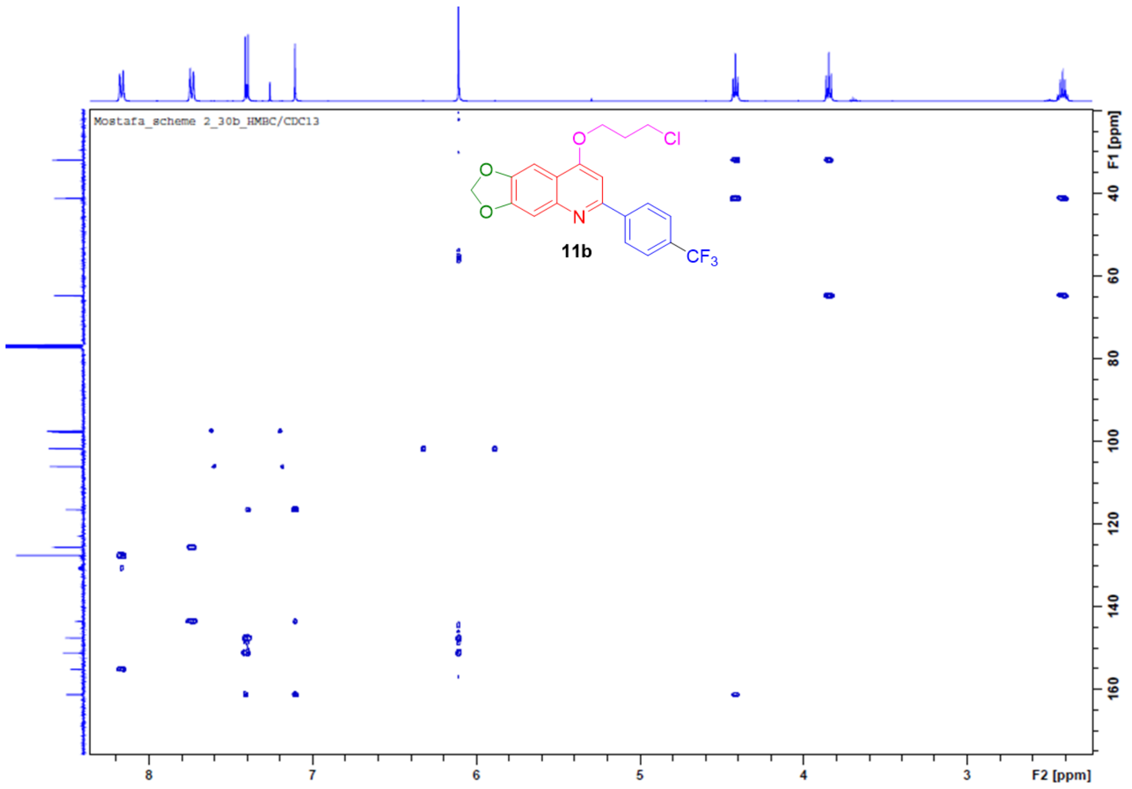
Mostafa_scheme 2_30b_HMBC/CDCl3
O
Cl
O
O
N
11b
CF3
F1 [ppm]
40
60
80
100
120
140
160
8
7
6
5
4
3
F2 [ppm]

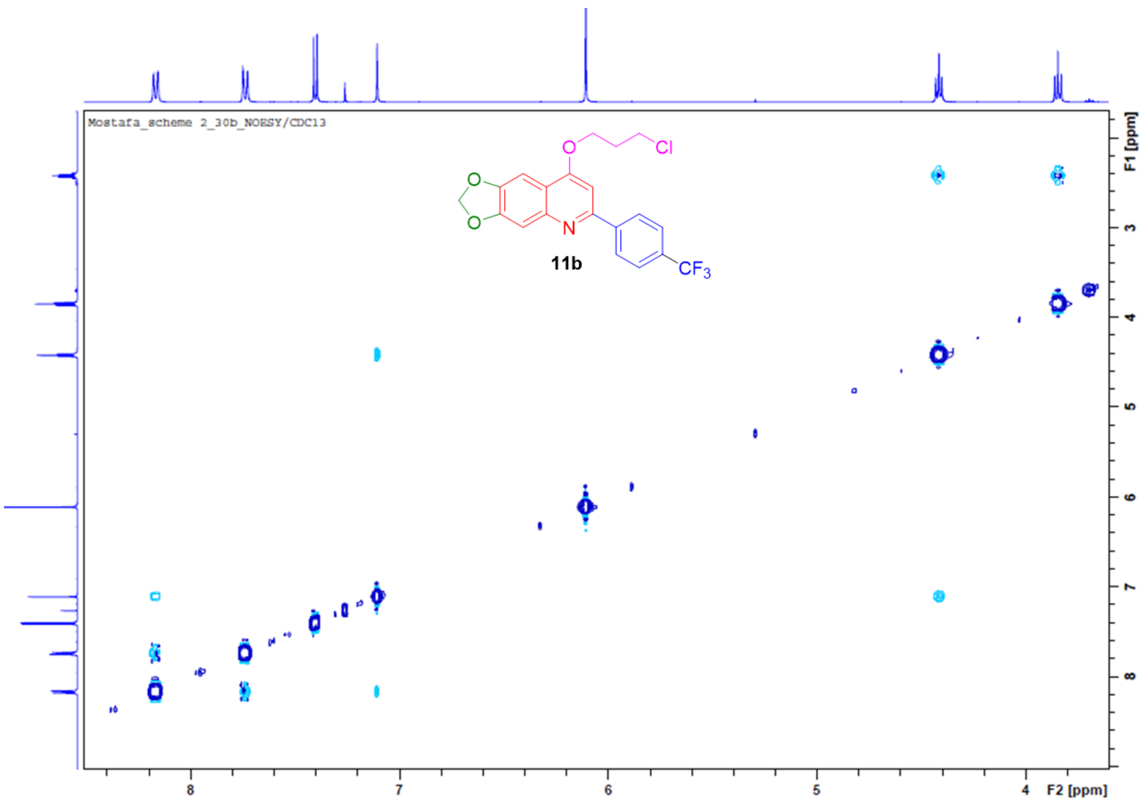
Mostafa_scheme 2_30b_NOESY/CDCl3
O
Cl
O
O
N
11b
CF3
F1 [ppm]
F2 [ppm]

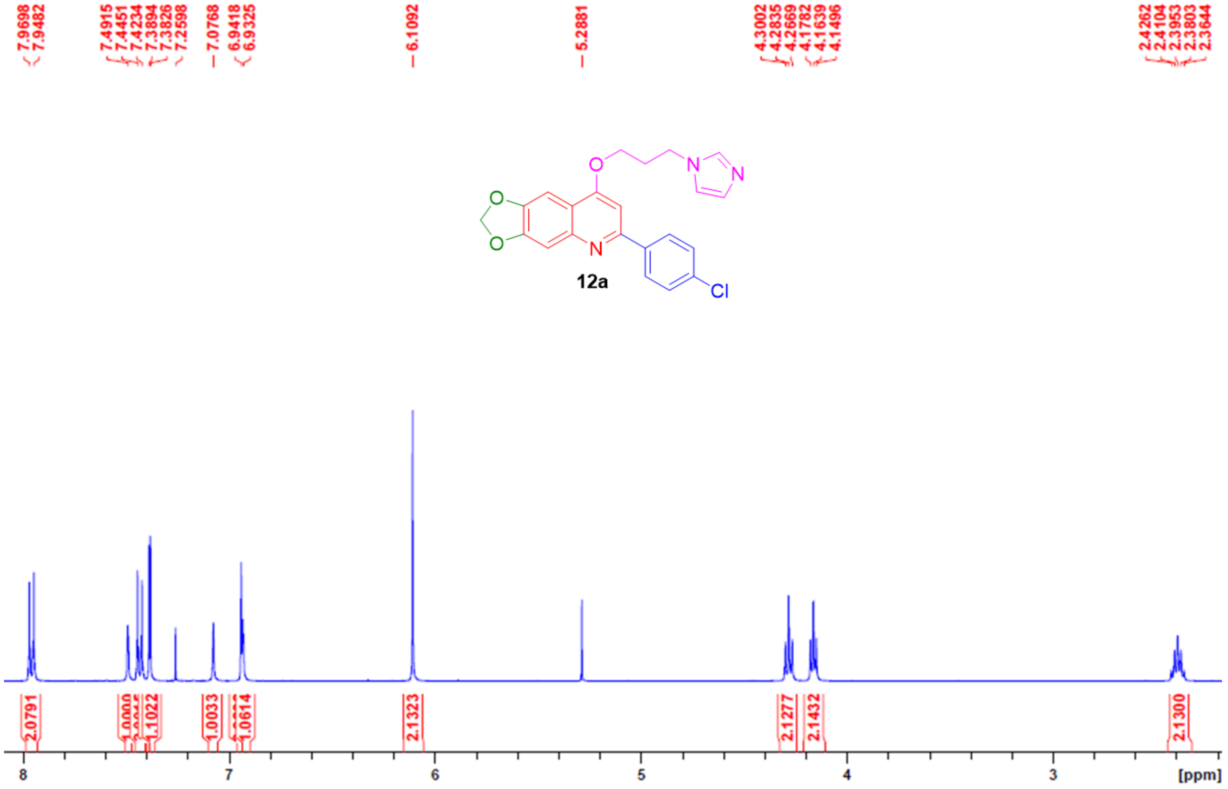
7.9698
7.9482
7.4915
7.4451
7.4234
7.3894
7.3826
7.2598
7.0768
6.9418
6.9325
6.1092
5.2881
4.3002
4.2835
4.2669
4.1782
4.1639
4.1496
2.4262
2.4104
2.3953
2.3803
2.3644
O
N
N
O
O
N
12a
Cl
2.0791
1.0000
1.1022
1.0033
1.0614
2.1323
2.1277
2.1432
2.1300
[ppm]

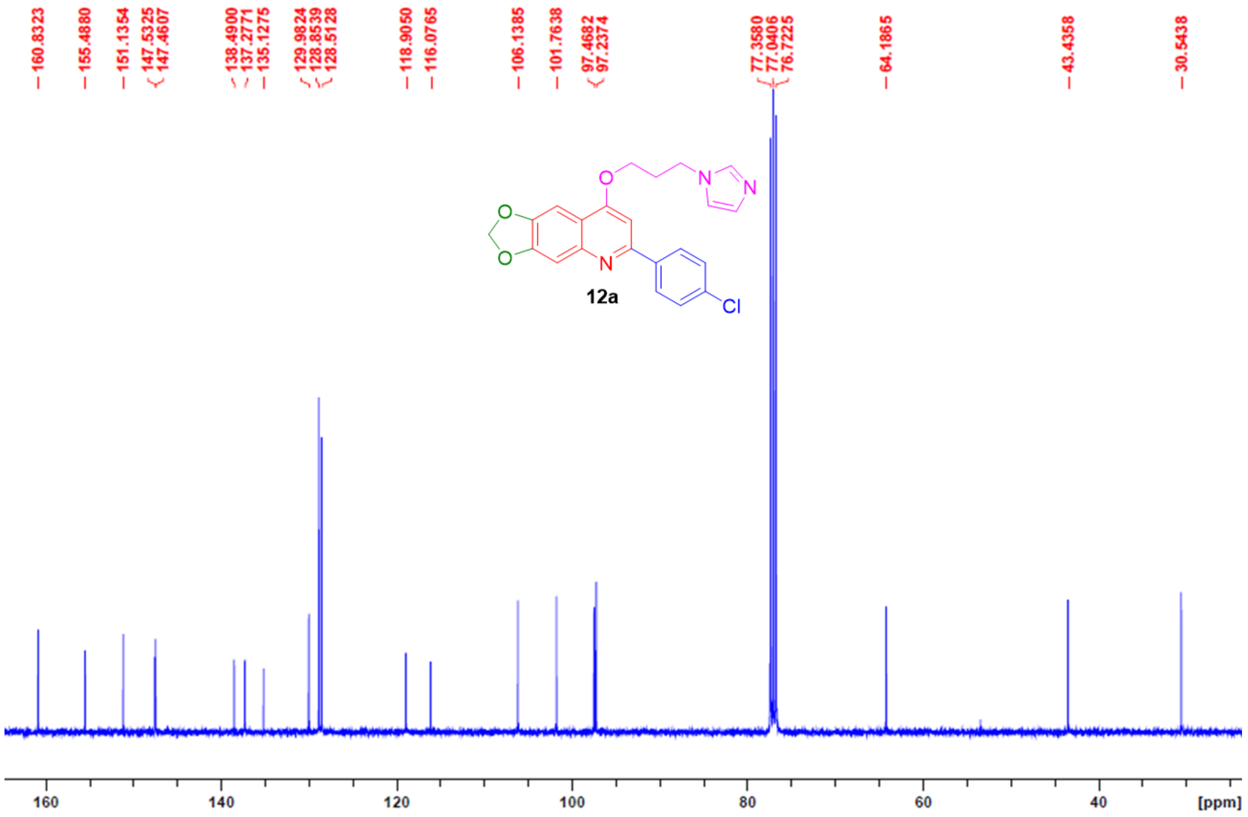
160.8323
155.4880
151.1354
147.5325
147.4607
138.4900
137.2771
135.1275
129.9824
128.8539
128.5128
118.9050
116.0765
106.1385
101.7638
97.4682
97.2374
77.3580
77.0406
76.7225
64.1865
43.4358
30.5438
O
N
N
O
O
N
12a
Cl
160
140
120
100
80
60
40
[ppm]

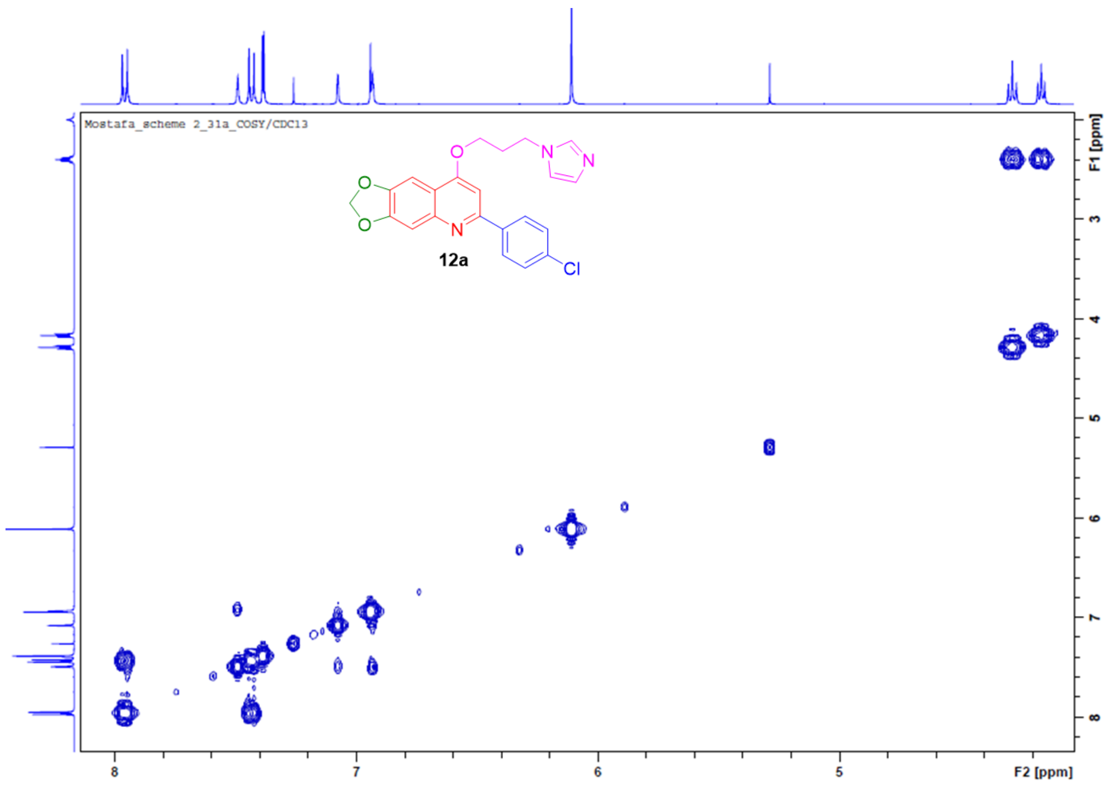
Mostafa_scheme 2_31a_COSY/CDCl3
O
N
N
O
O
N
12a
Cl
F1 [ppm]
3
4
5
6
7
8
8
7
6
5
F2 [ppm]

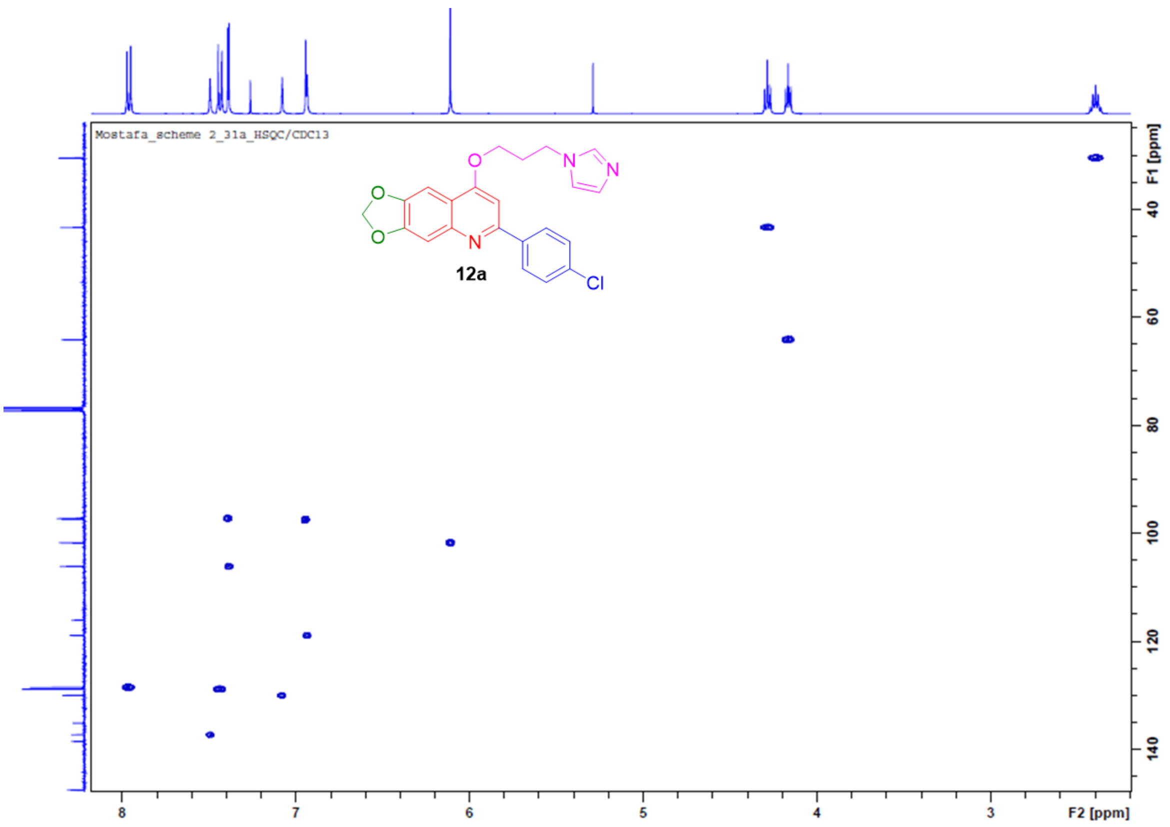
Mostafa_scheme 2_31a_HSQC/CDCl3
12a
F1 [ppm]
F2 [ppm]

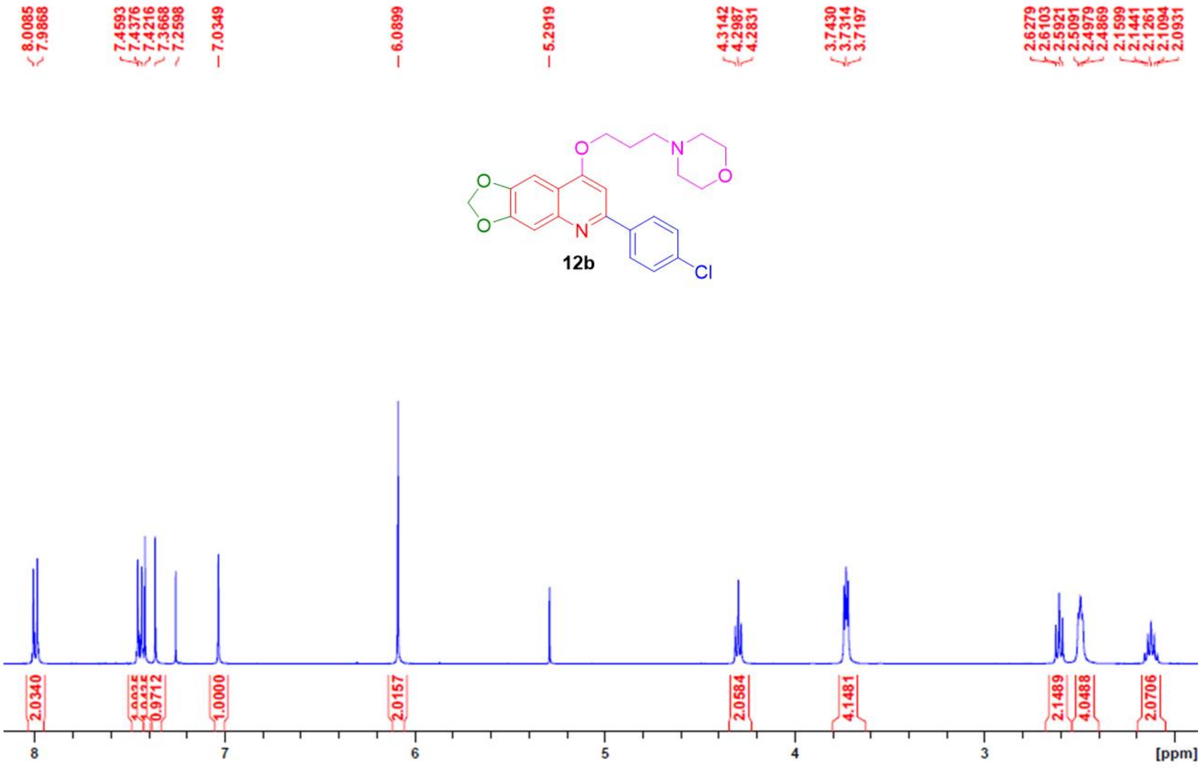
8.0085
7.9868
7.4593
7.4376
7.4216
7.3668
7.2598
7.0349
6.0899
5.2919
4.3142
4.2987
4.2831
3.7430
3.7314
3.7197
2.6279
2.6103
2.5921
2.5091
2.4979
2.4869
2.1599
2.1441
2.1261
2.1094
2.0931
12b
2.0340
0.9712
1.0000
2.0157
2.0584
4.1481
2.1489
4.0488
2.0706
[ppm]

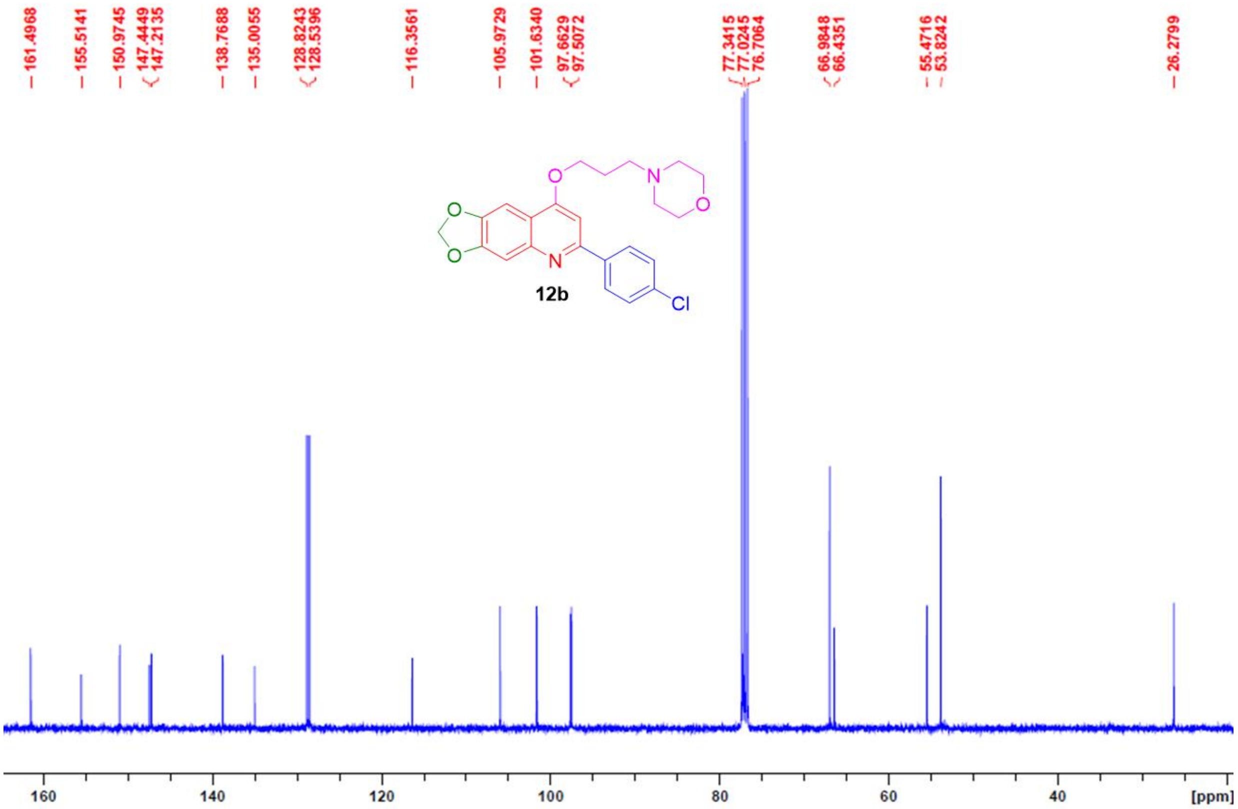
161.4968
155.5141
150.9745
147.4449
147.2135
138.7688
135.0055
128.8243
128.5396
116.3561
105.9729
101.6340
97.6629
97.5072
77.3415
77.0245
76.7064
66.9848
66.4351
55.4716
53.8242
26.2799
12b
[ppm]

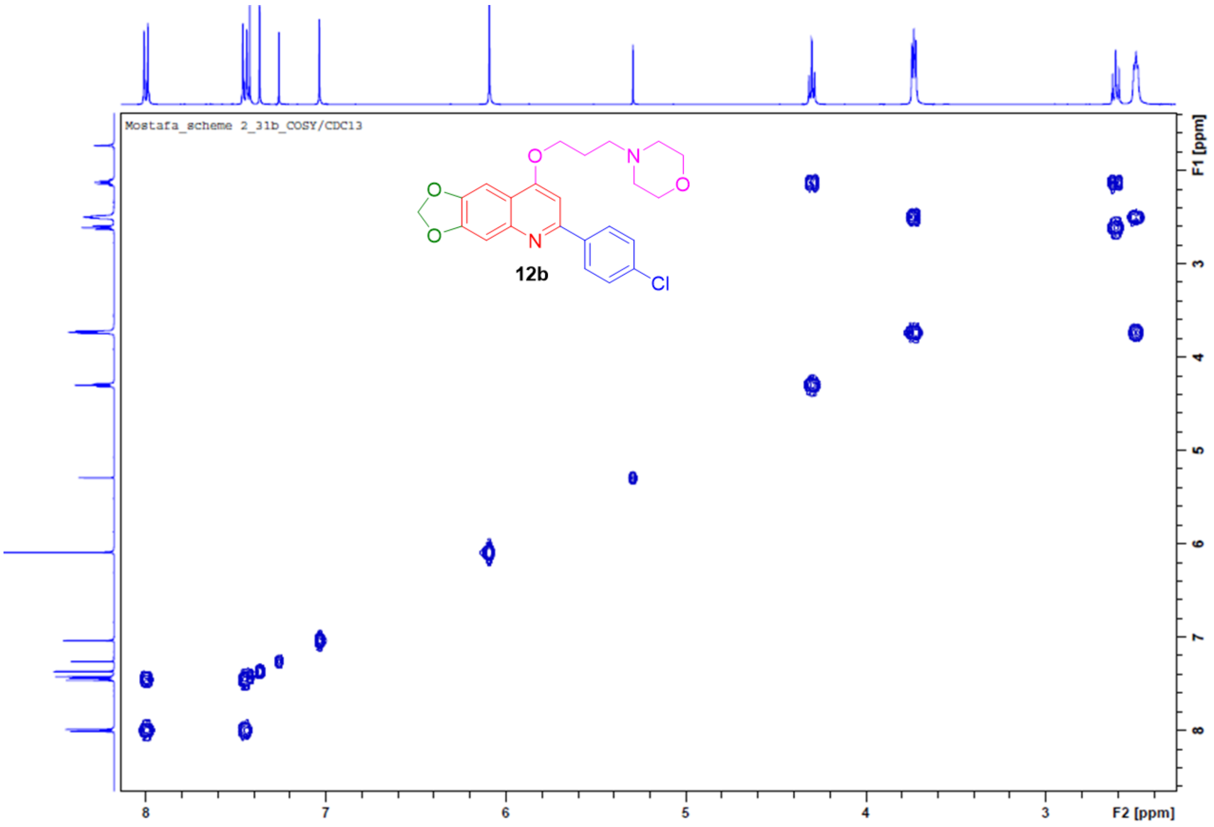
Mostafa_scheme 2_31b_COSY/CDCl3
12b
F1 [ppm]
F2 [ppm]

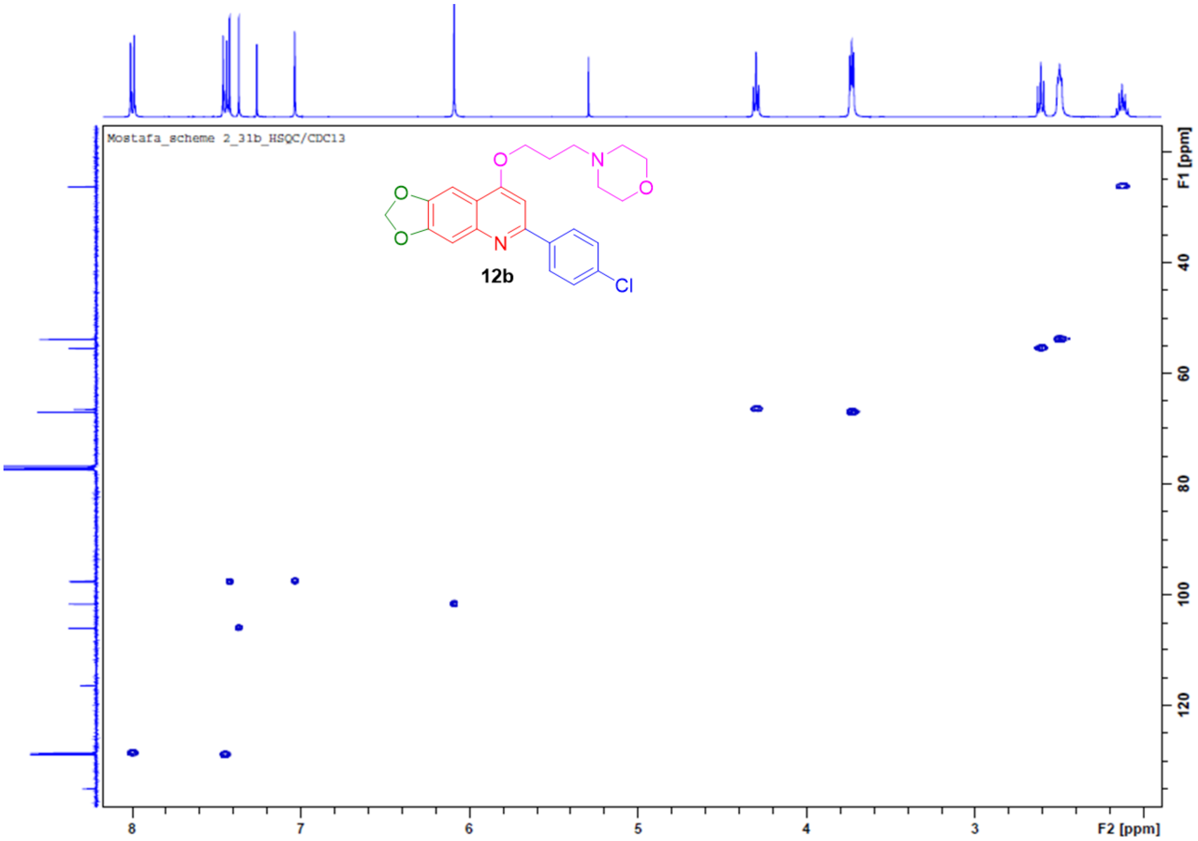
Mostafa_scheme 2_31b_HSQC/CDCl3
12b
F1 [ppm]
F2 [ppm]

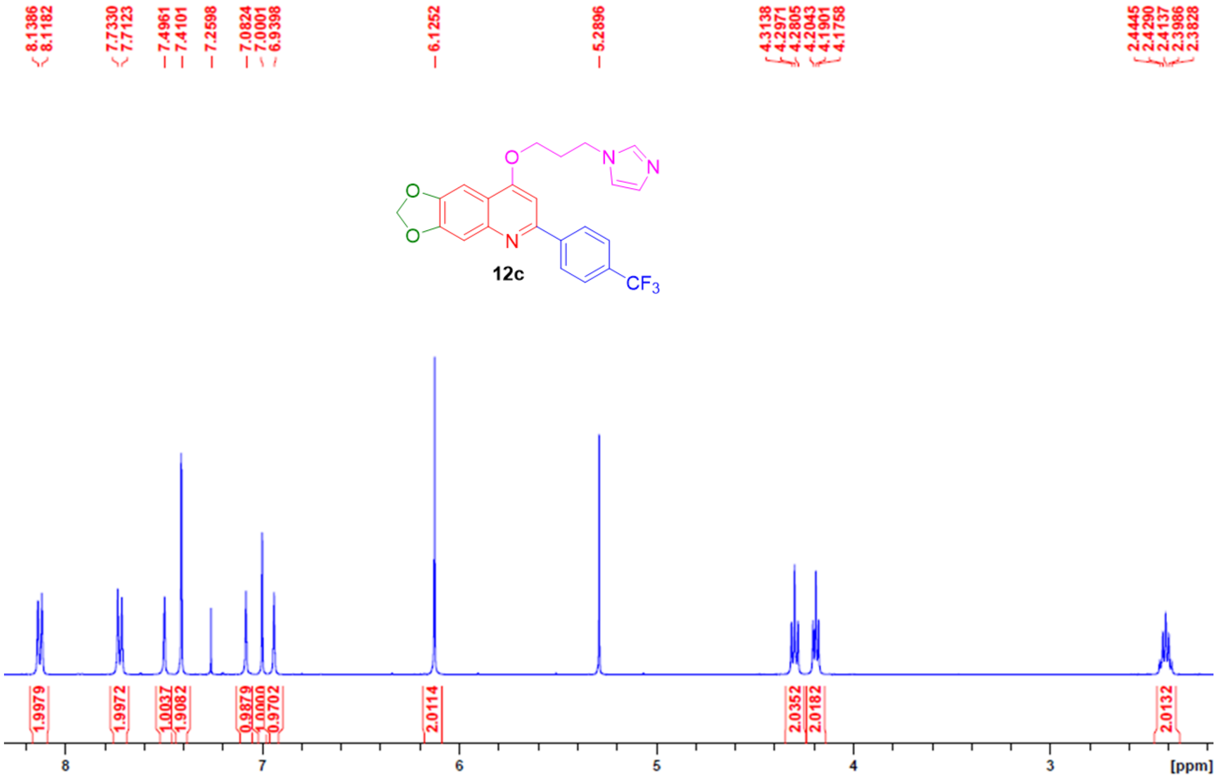
8.1386
8.1182
7.7330
7.7123
7.4961
7.4101
7.2598
7.0824
7.0001
6.9398
6.1252
5.2896
4.3138
4.2971
4.2805
4.2043
4.1901
4.1758
2.4445
2.4290
2.4137
2.3986
2.3828
12c
1.9979
1.9972
1.0037
1.9082
0.9879
1.0000
0.9702
2.0114
2.0352
2.0182
2.0132
[ppm]

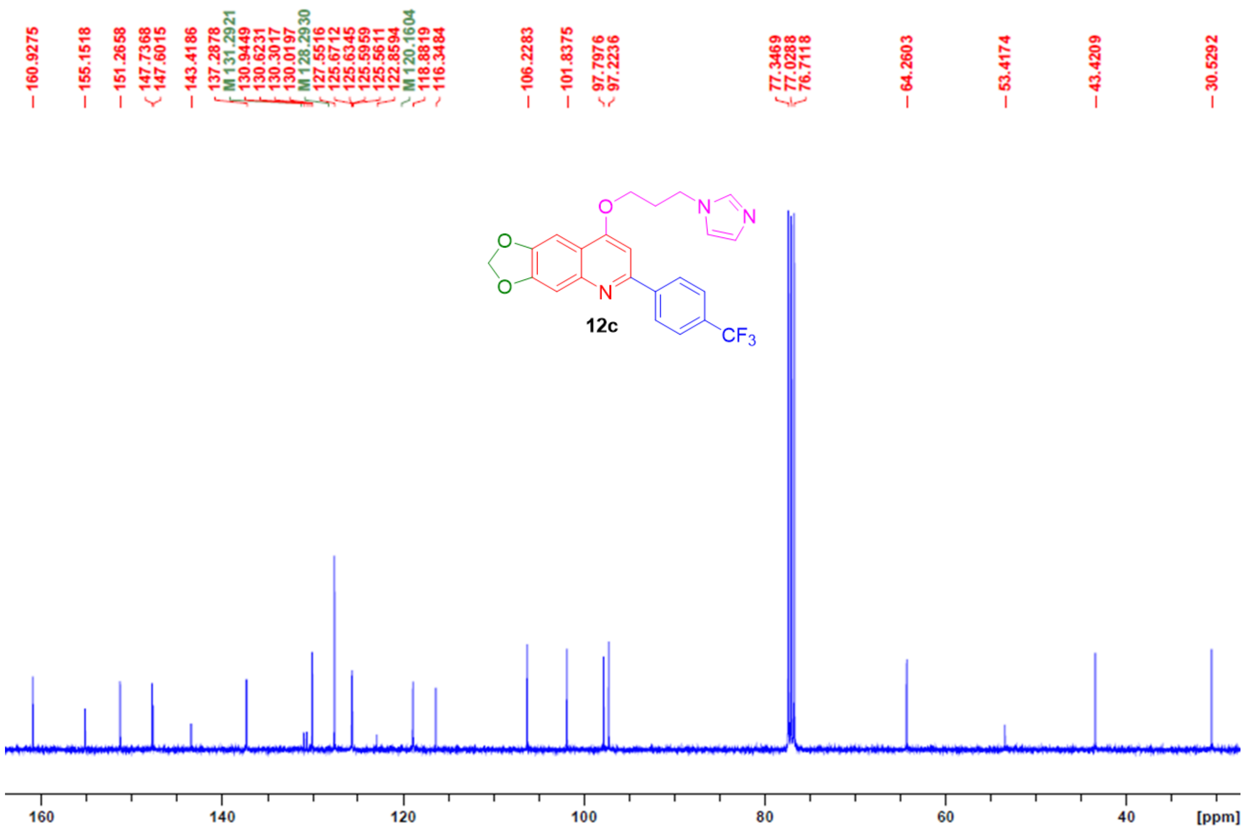
160.9275
155.1518
151.2658
147.7368
147.6015
143.4186
137.2878
M 131.2921
130.9449
130.6231
130.3017
130.0197
M 128.2930
127.5516
125.6712
125.6345
125.5959
125.5611
122.8594
M 120.1604
118.8819
116.3484
106.2283
101.8375
97.7976
97.2236
77.3469
77.0288
76.7118
64.2603
53.4174
43.4209
30.5292
12c
160
140
120
100
80
60
40
[ppm]

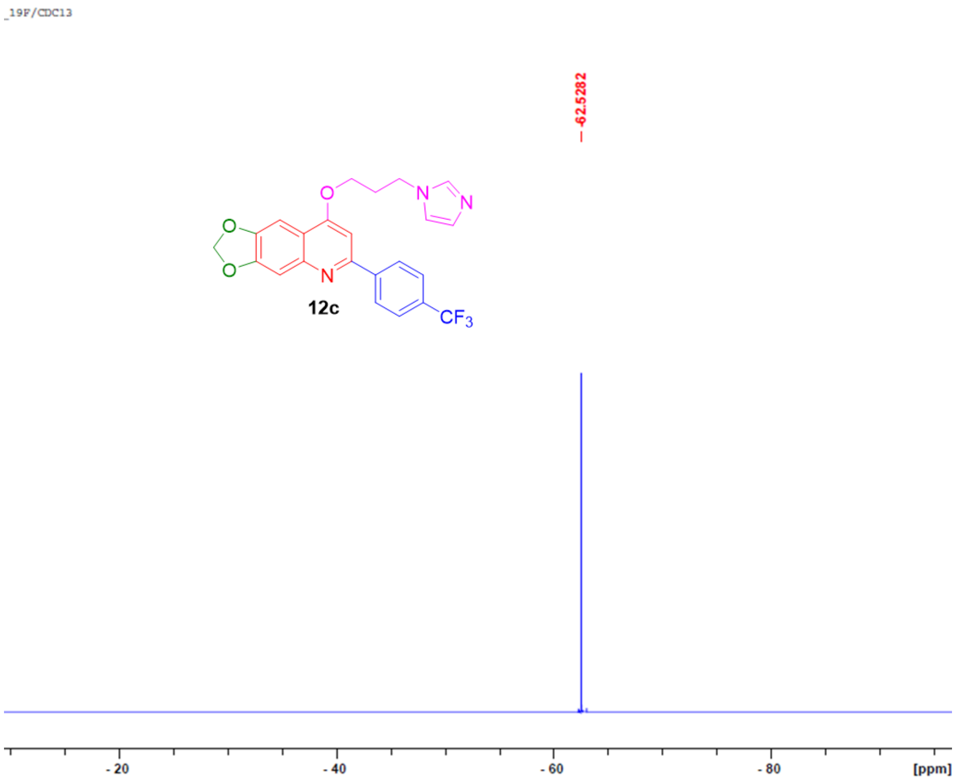
_19F/CDCl3
-62.5282
12c
-20
-40
-60
-80
[ppm]

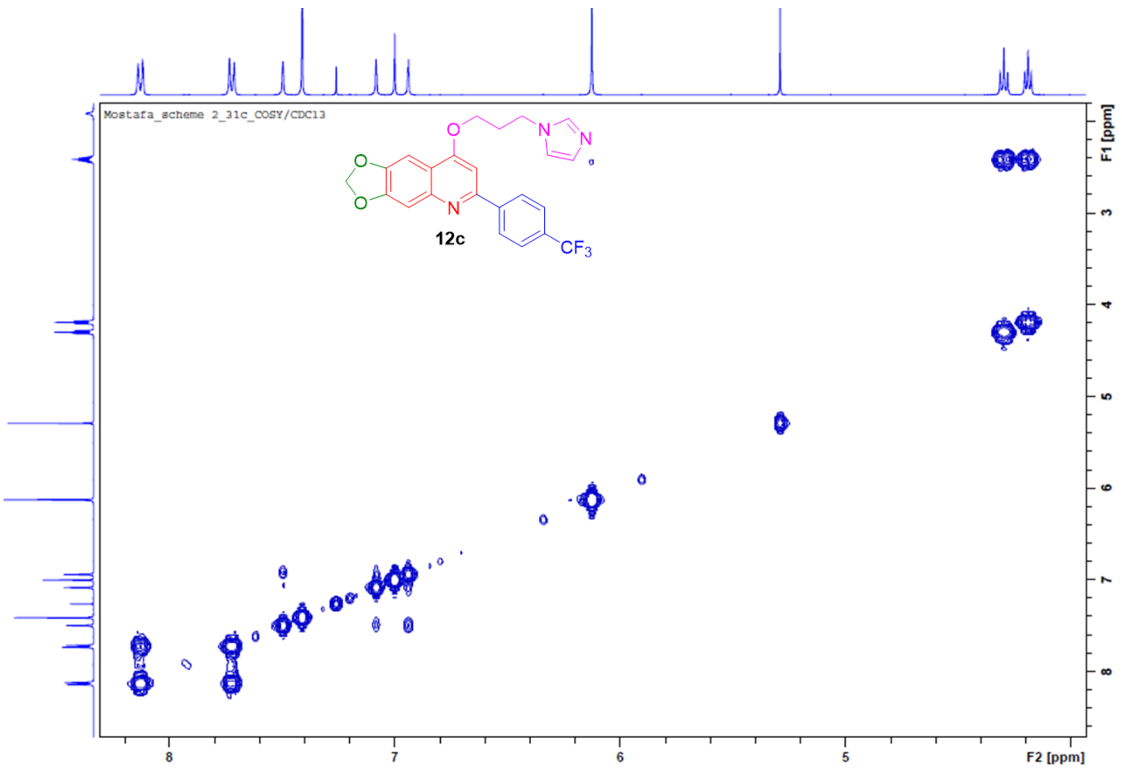
Mostafa_scheme 2_31c_COSY/CDCl3
12c
CF3
F1 [ppm]
F2 [ppm]

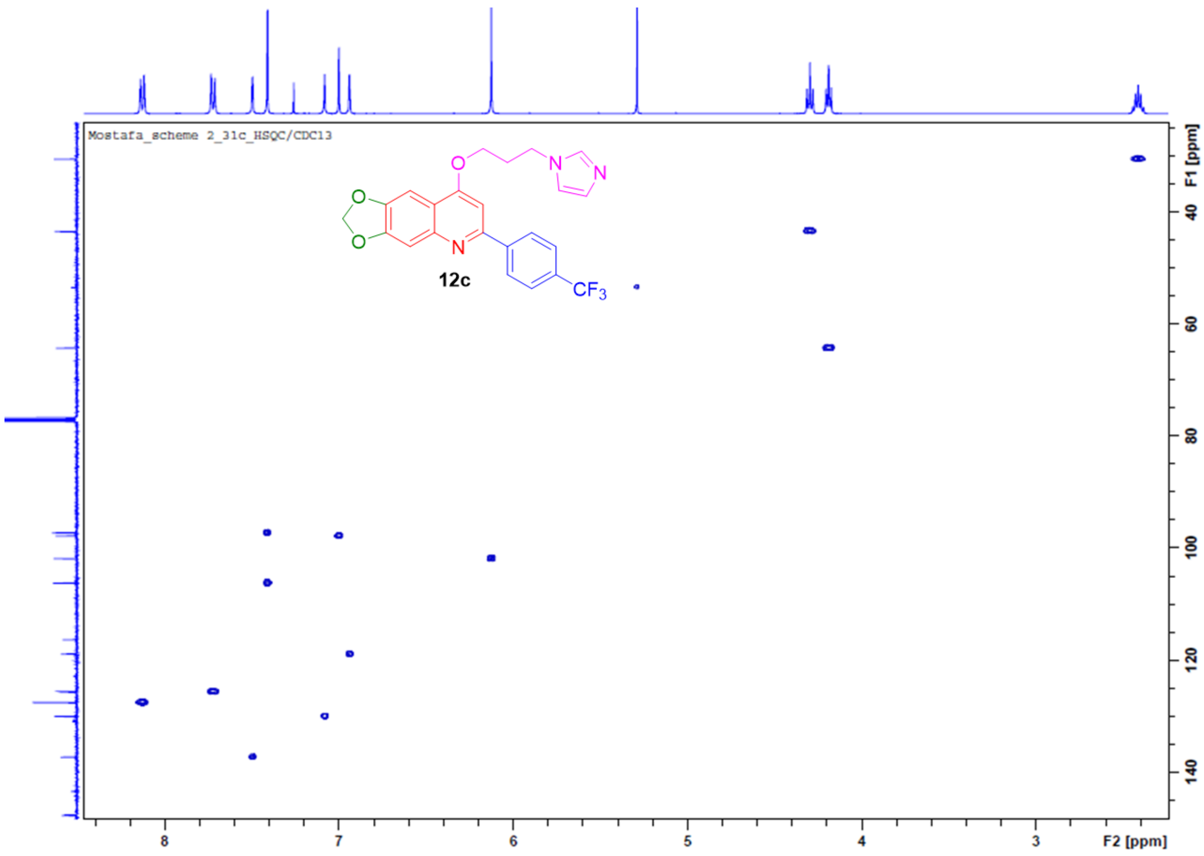
Mostafa_scheme 2_31c_HSQC/CDCl3
12c
CF3
F1 [ppm]
F2 [ppm]

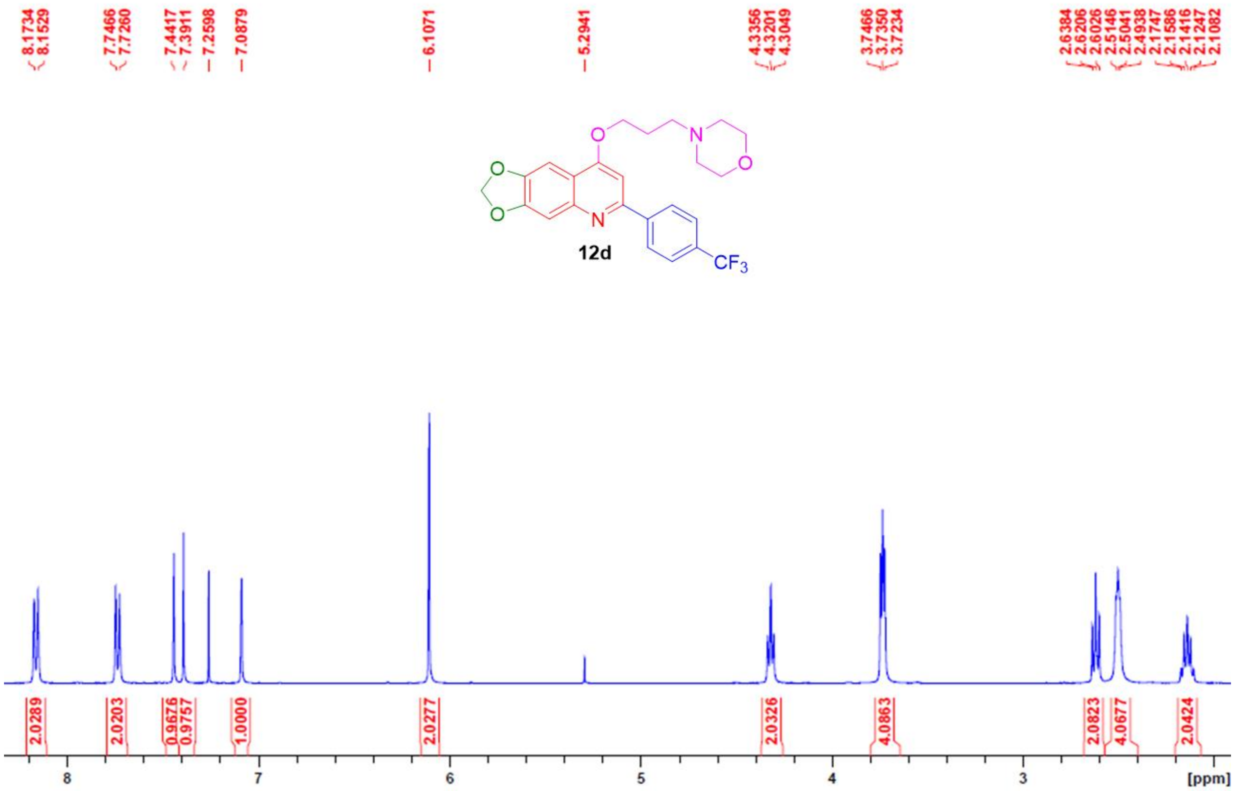
8.1734
8.1529
7.7466
7.7260
7.4417
7.3911
7.2598
7.0879
6.1071
5.2941
4.3356
4.3201
4.3049
3.7466
3.7350
3.7234
2.6384
2.6206
2.6026
2.5146
2.5041
2.4938
2.1747
2.1586
2.1416
2.1247
2.1082
O
N
O
O
N
O
12d
CF3
2.0289
2.0203
0.9676
0.9757
1.0000
2.0277
2.0326
4.0863
2.0823
4.0677
2.0424
8
7
6
5
4
3
[ppm]

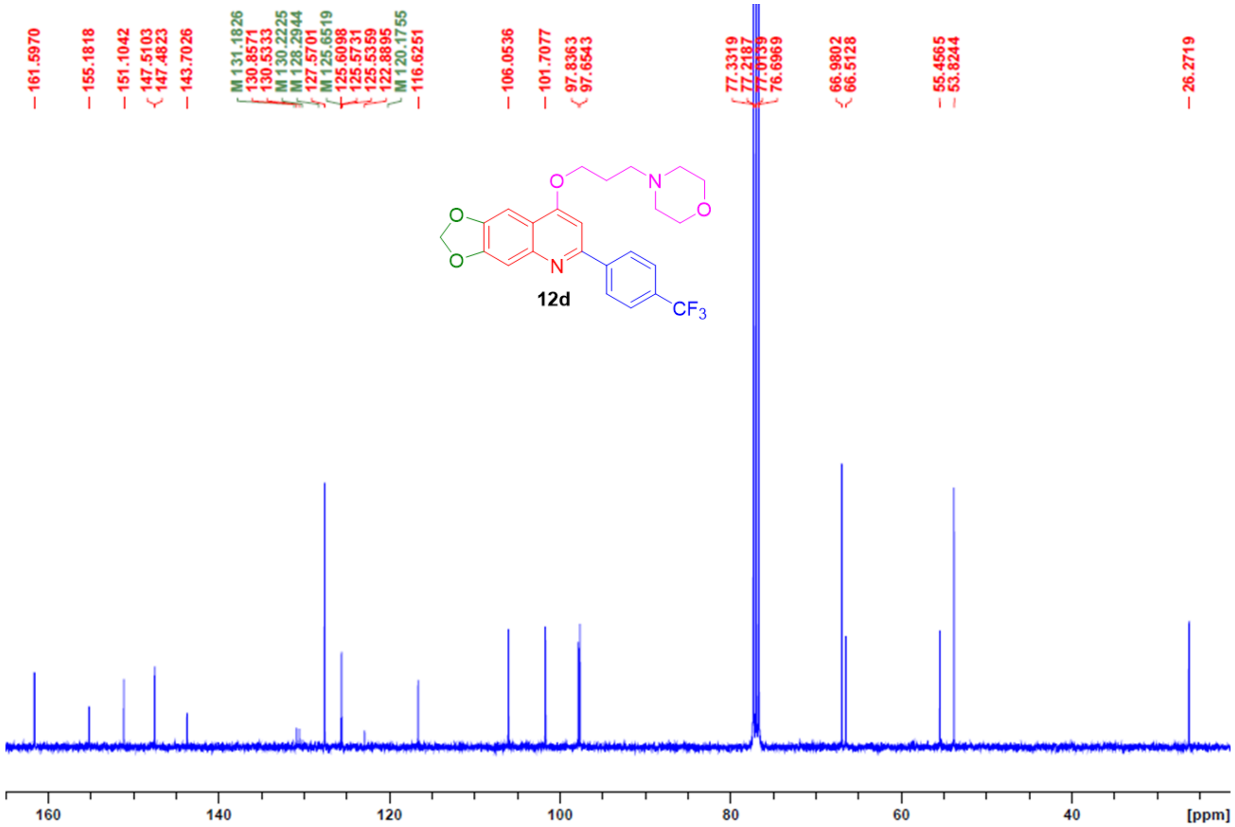
161.5970
155.1818
151.1042
147.5103
147.4823
143.7026
M 131.1826
130.8571
130.5333
M 130.2225
M 128.2944
127.5701
M 125.6519
125.6098
125.5731
125.5359
122.8895
M 120.1755
116.6251
106.0536
101.7077
97.8363
97.6543
77.3319
77.2187
77.0139
76.6969
66.9802
66.5128
55.4565
53.8244
26.2719
12d
CF3
160
140
120
100
80
60
40
[ppm]

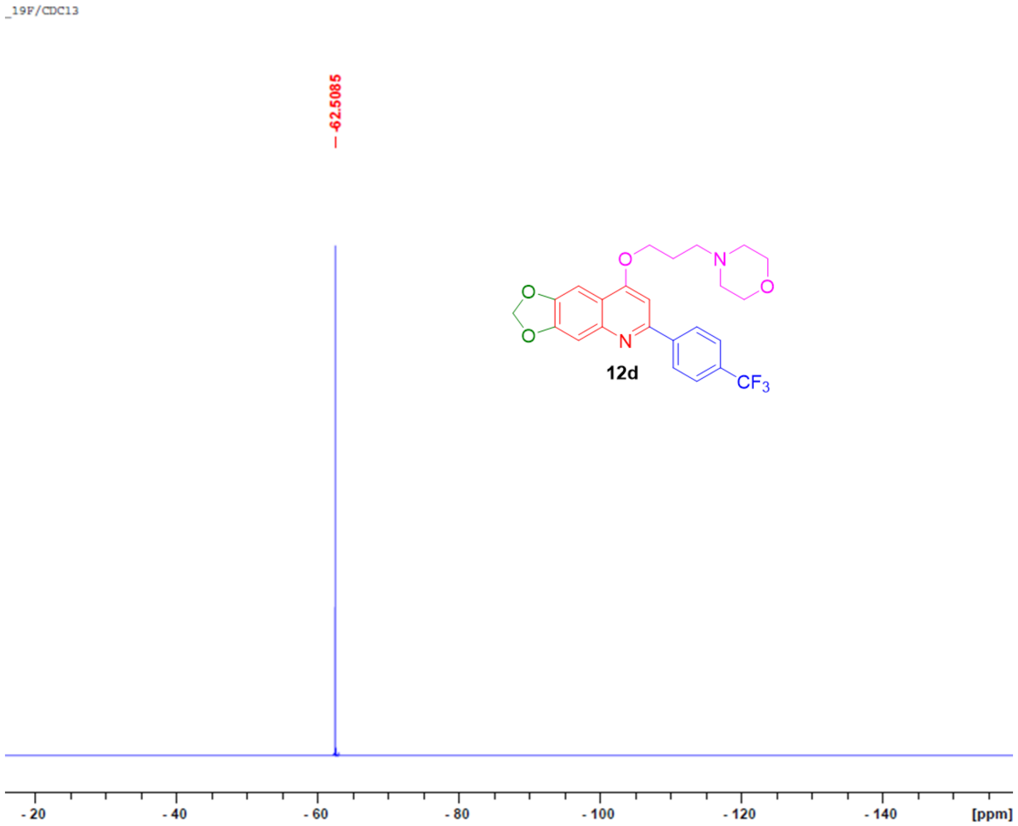
_19F/CDCl3
-62.5085
O
O
O
N
N
O
CF3
12d
-20
-40
-60
-80
-100
-120
-140
[ppm]

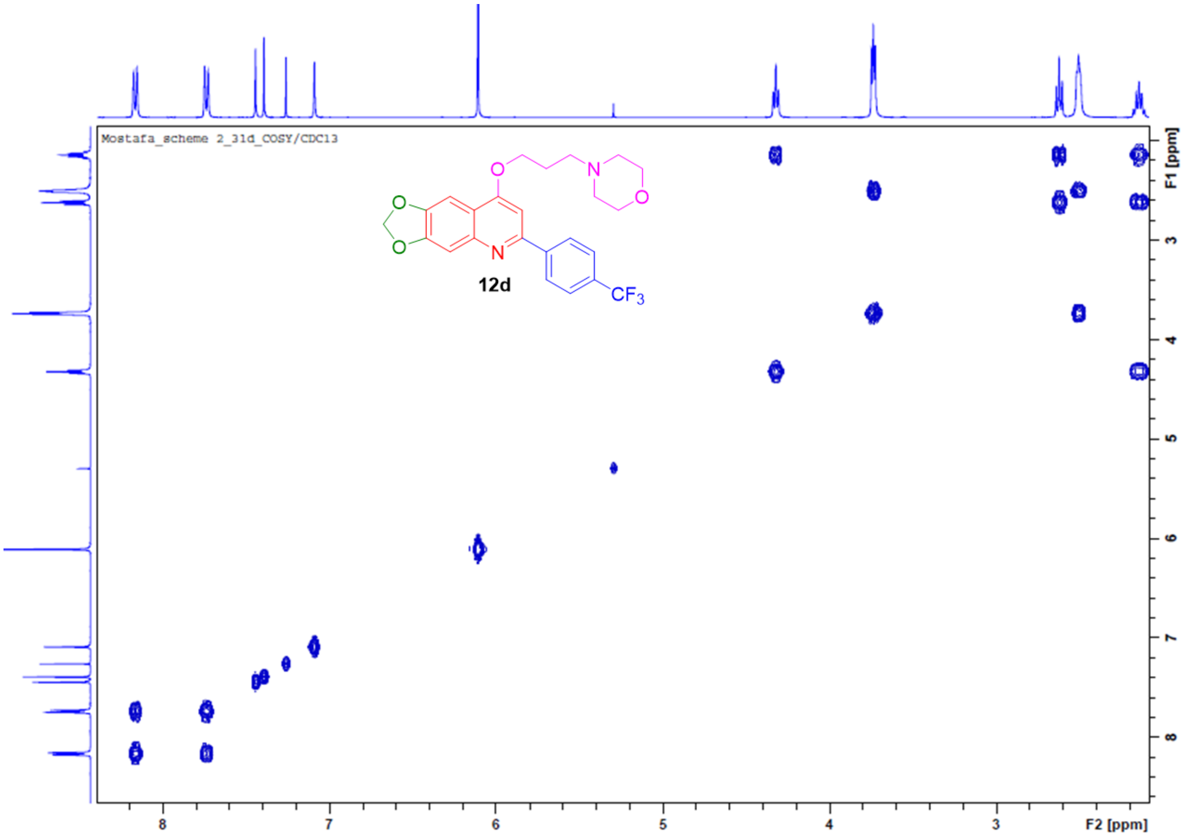
Mostafa_scheme 2_31d_COSY/CDCl3
O
O
O
N
N
O
CF3
12d
F1 [ppm]
F2 [ppm]
8
7
6
5
4
3

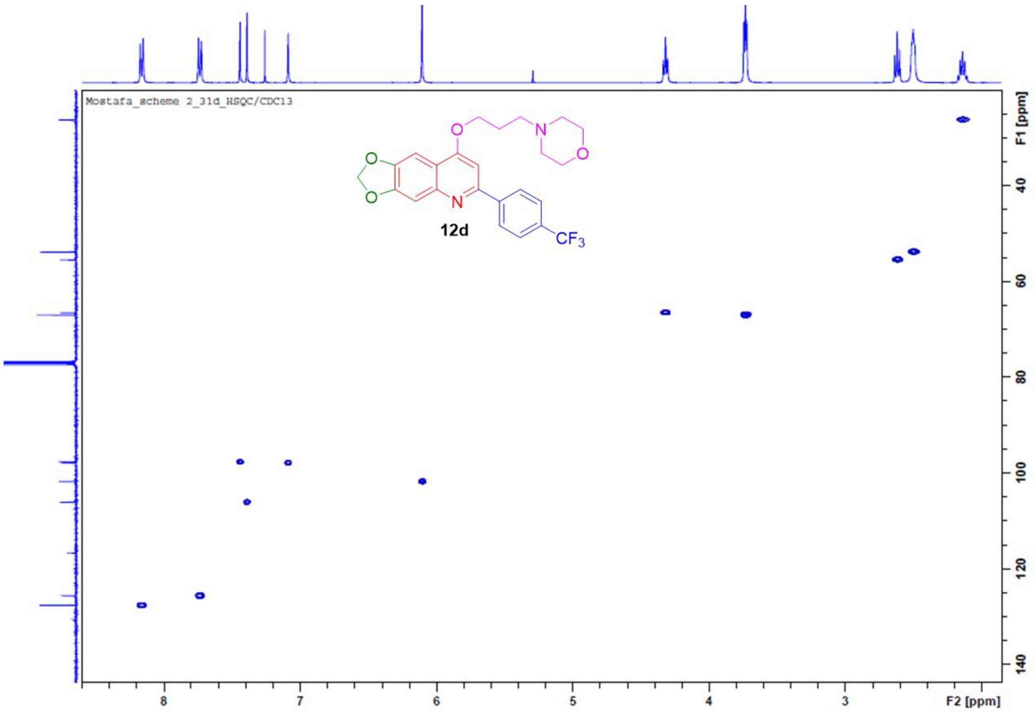
Mostafa_scheme 2_31d_HSQC/CDCl3
12d
CF3
F1 [ppm]
F2 [ppm]

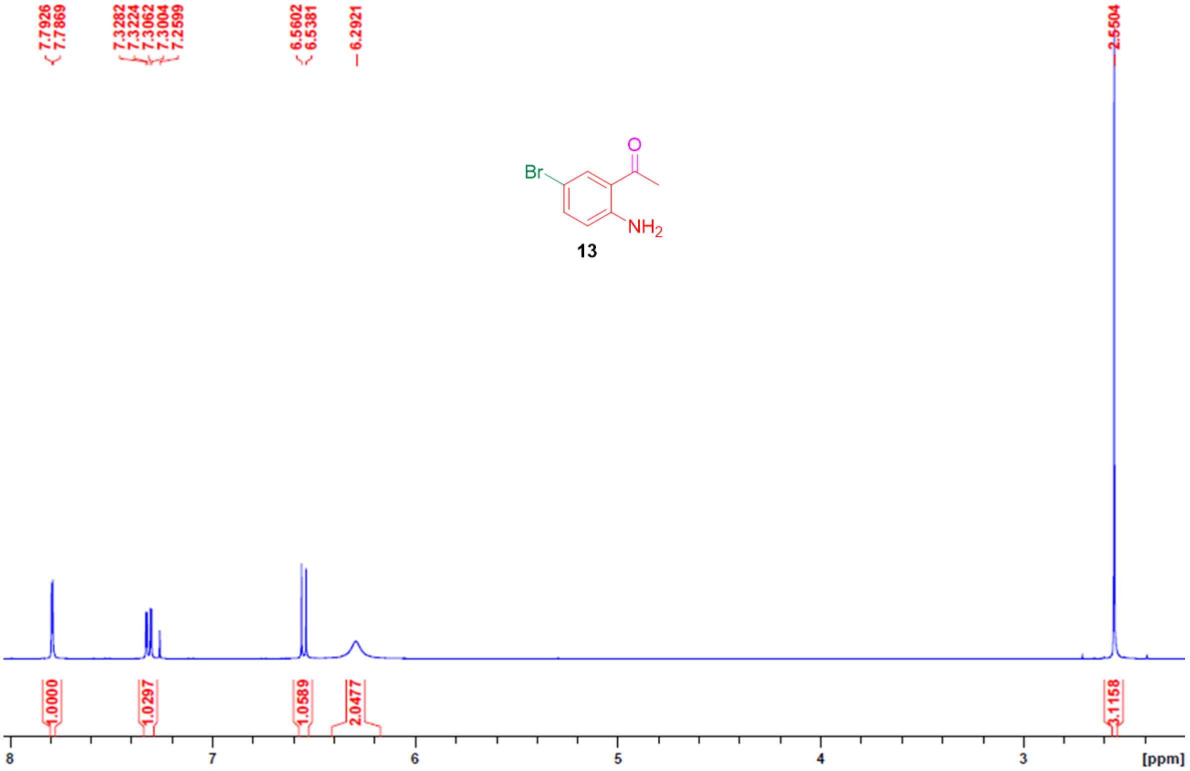
7.7926
7.7869
7.3282
7.3224
7.3062
7.3004
7.2599
6.5602
6.5381
6.2921
2.5504
Br
NH2
13
1.0000
1.0297
1.0589
2.0477
3.1158
[ppm]

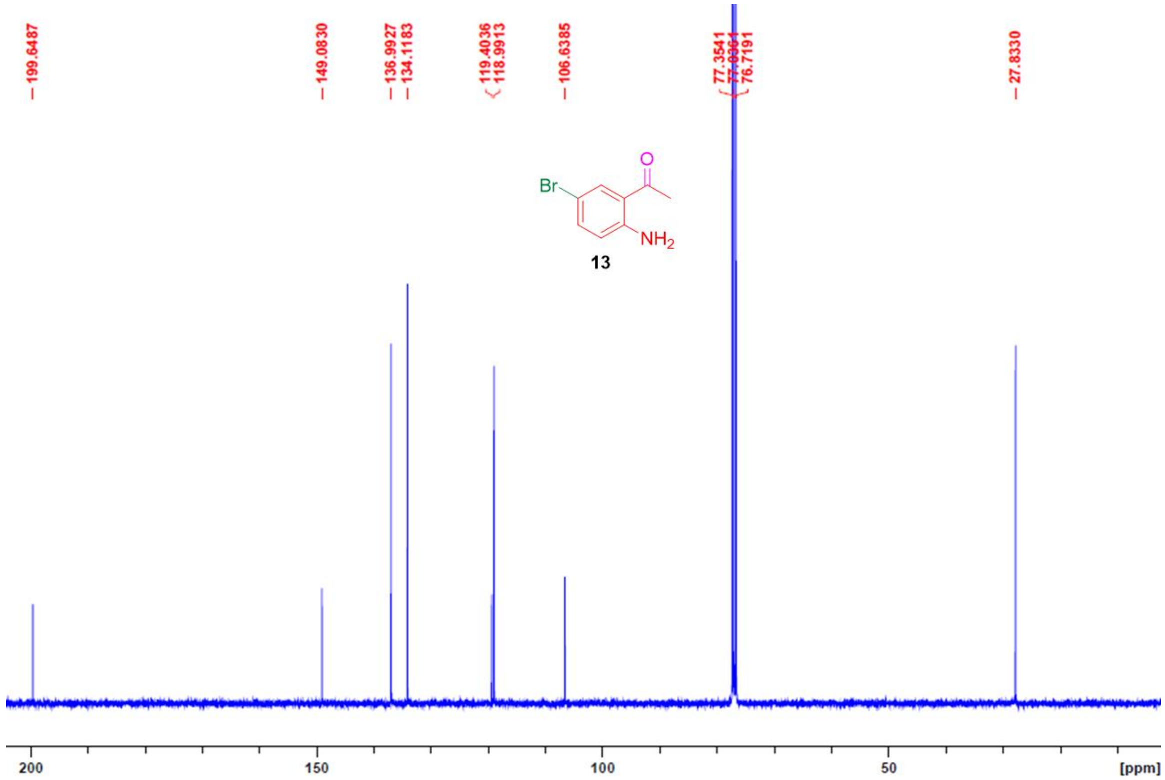

199.6487
149.0830
136.9927
134.1183
119.4036
118.9913
106.6385
77.3541
76.7191
27.8330
Br
O
NH2
13
200
150
100
50
[ppm]

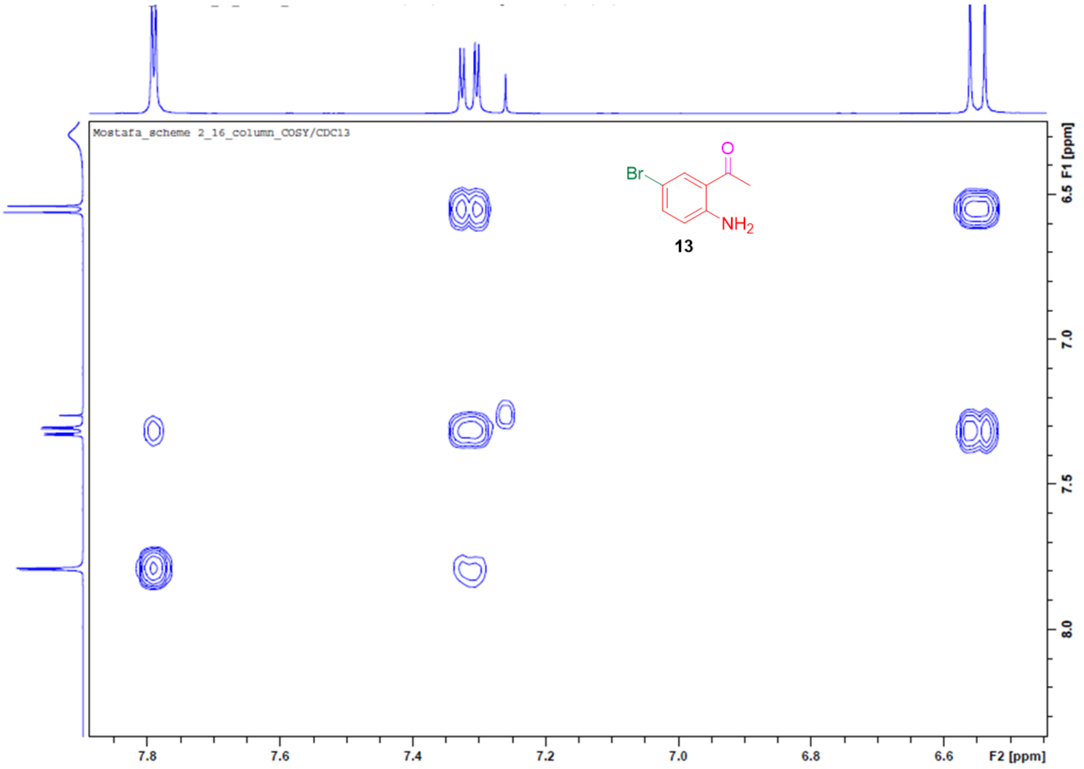

Mostafa_scheme 2_16_column_COSY/CDCl3
Br
O
NH2
13
7.8
7.6
7.4
7.2
7.0
6.8
6.6
F2 [ppm]
6.5 F1 [ppm]
7.0
7.5
8.0

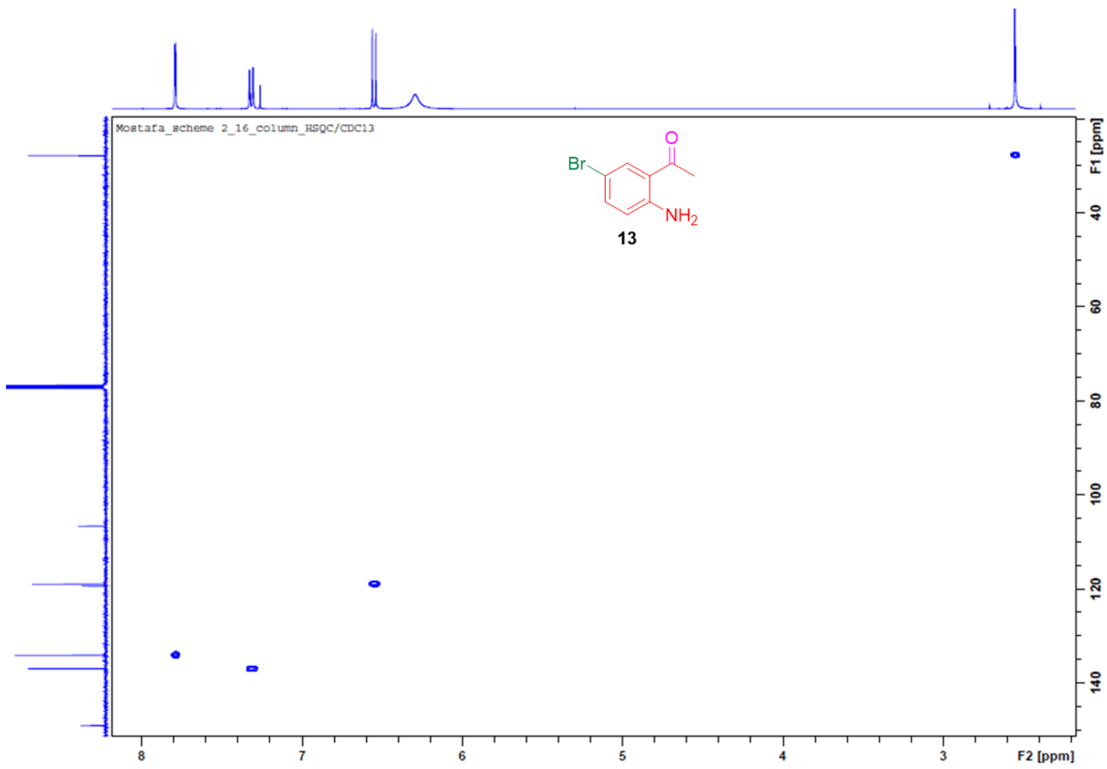
Mostafa_scheme 2_16_column_HSQC/CDCl3
Br
O
NH2
13
F1 [ppm]
F2 [ppm]

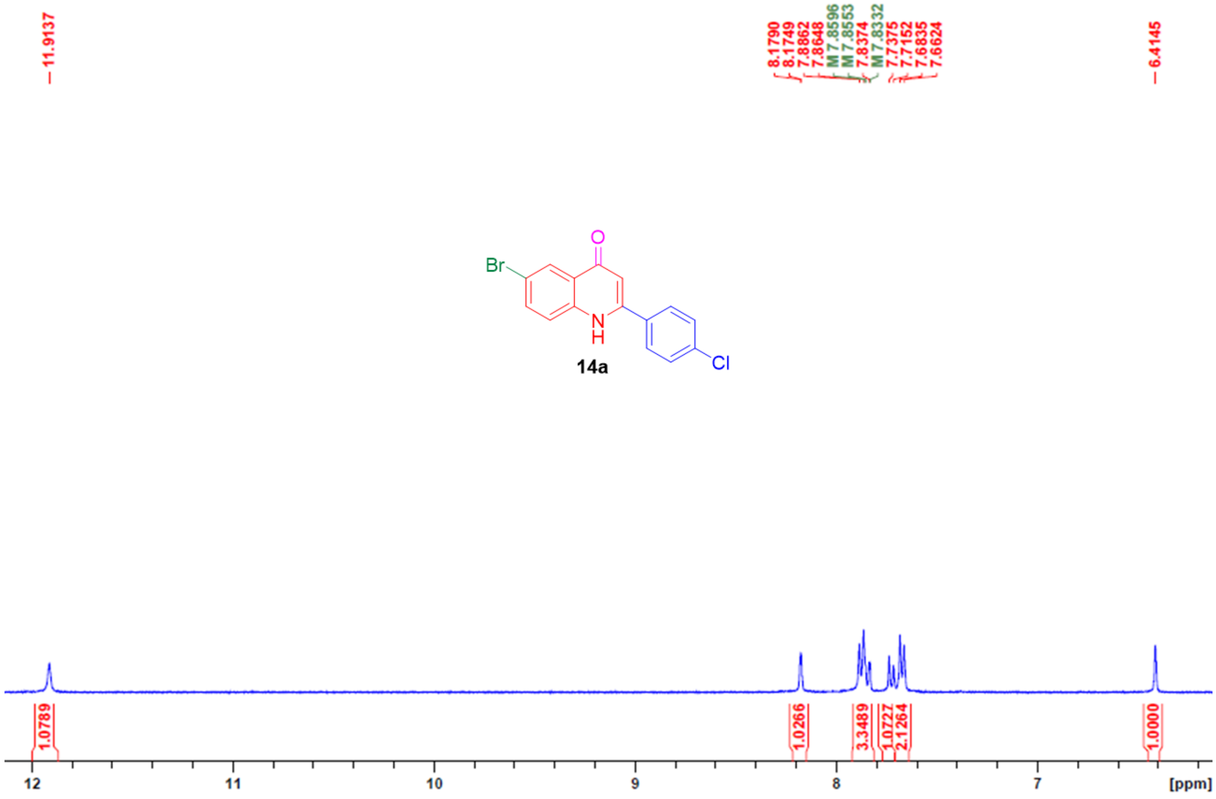
11.9137
8.1790
8.1749
7.8862
7.8648
M 7.8596
M 7.8553
7.8374
M 7.8332
7.7375
7.7152
7.6835
7.6624
6.4145
Br
O
N
H
Cl
14a
1.0789
1.0266
3.3489
1.0727
2.1264
1.0000
[ppm]

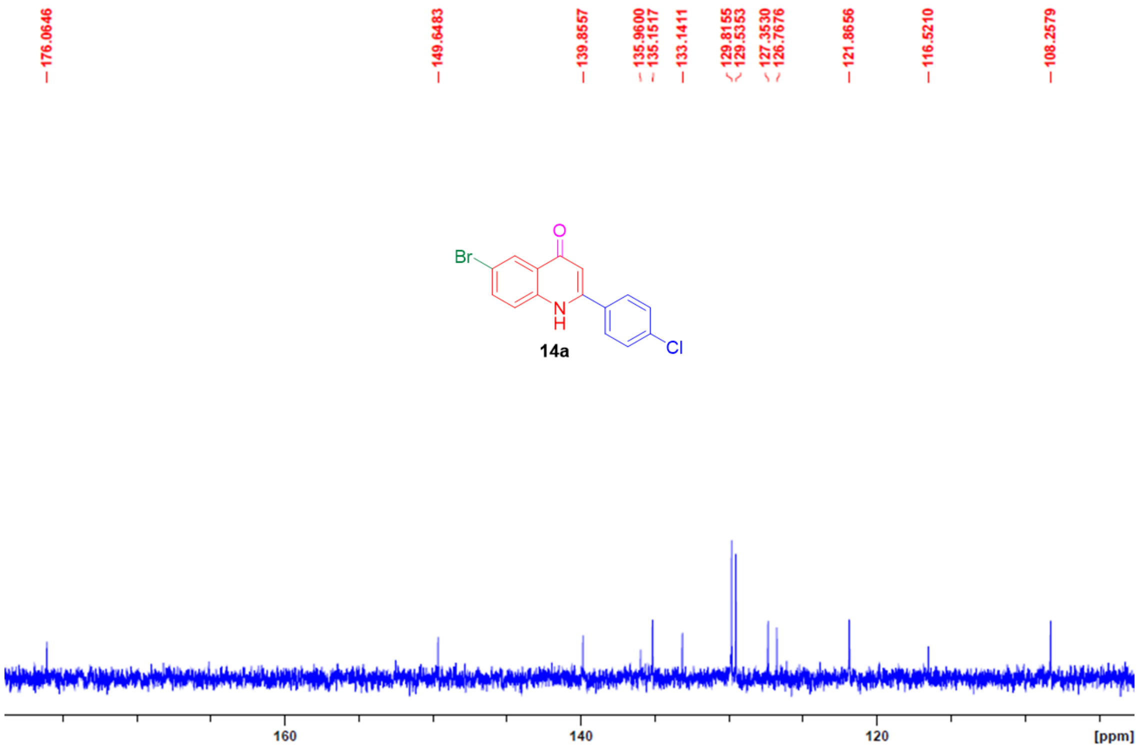
176.0646
149.6483
139.8557
135.9600
135.1517
133.1411
129.8155
129.5353
127.3530
126.7676
121.8656
116.5210
108.2579
Br
O
N
H
Cl
14a
160
140
120
[ppm]

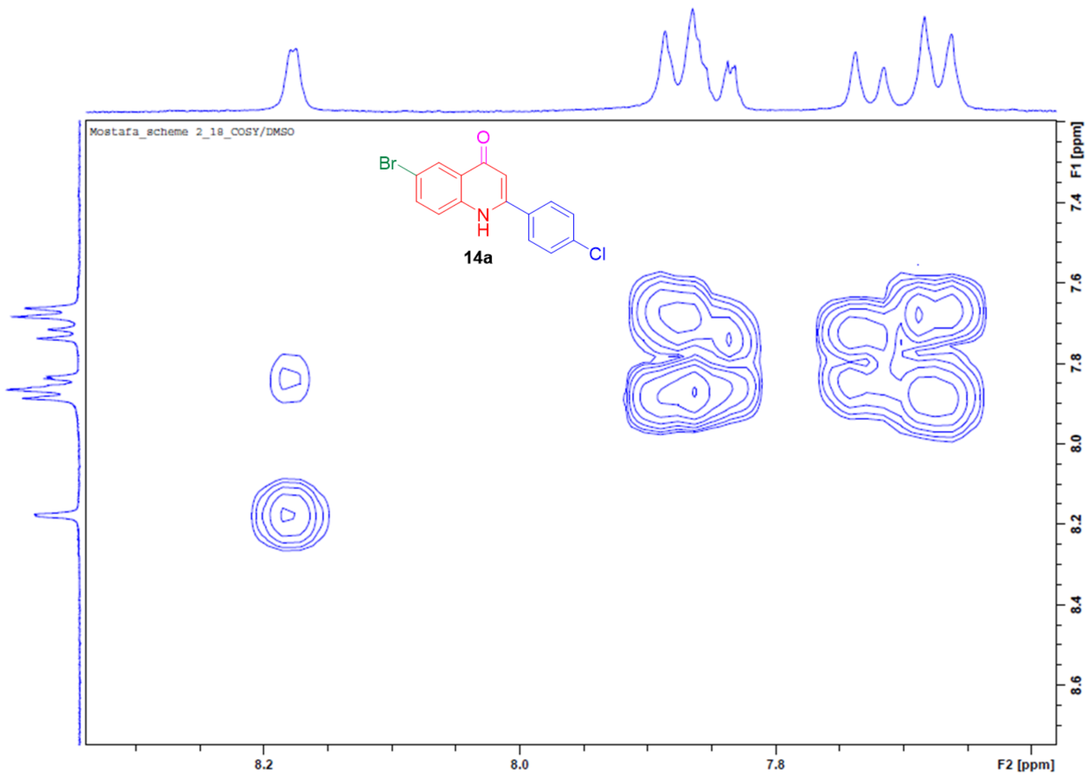
Mostafa_scheme 2_18_COSY/DMSO
Br
O
N
H
Cl
14a
F1 [ppm]
7.4
7.6
7.8
8.0
8.2
8.4
8.6
8.2
8.0
7.8
F2 [ppm]

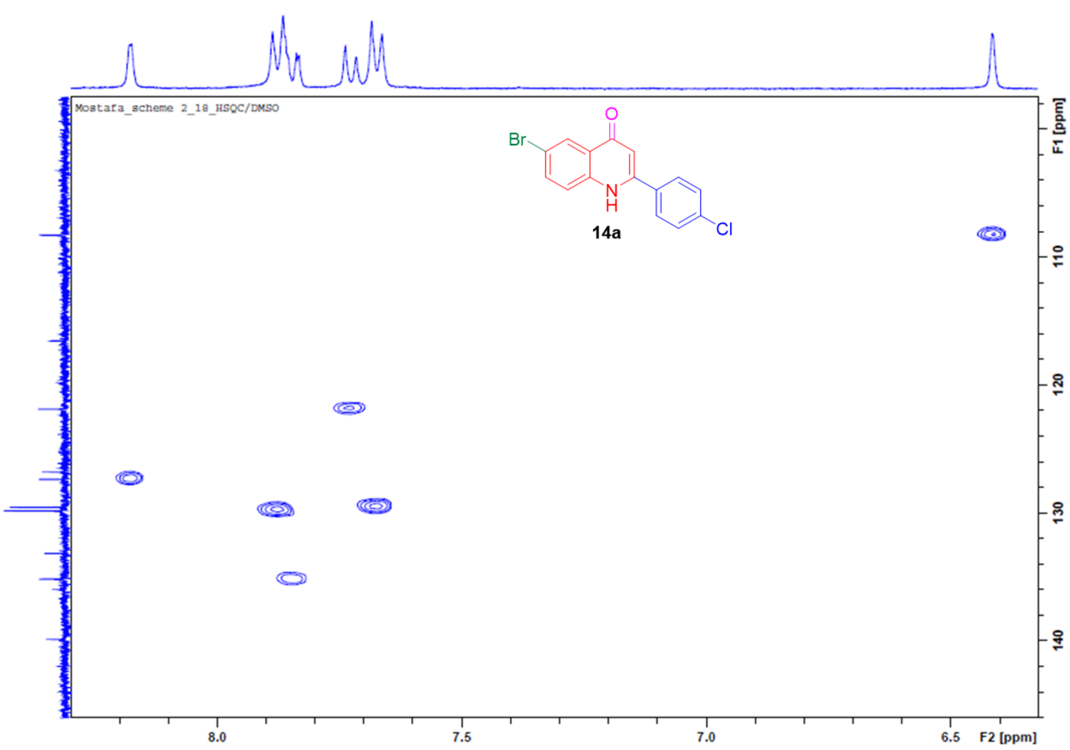
Mostafa_scheme 2_18_HSQC/DMSO
Br
O
N
H
Cl
14a
F1 [ppm]
110
120
130
140
8.0
7.5
7.0
6.5
F2 [ppm]

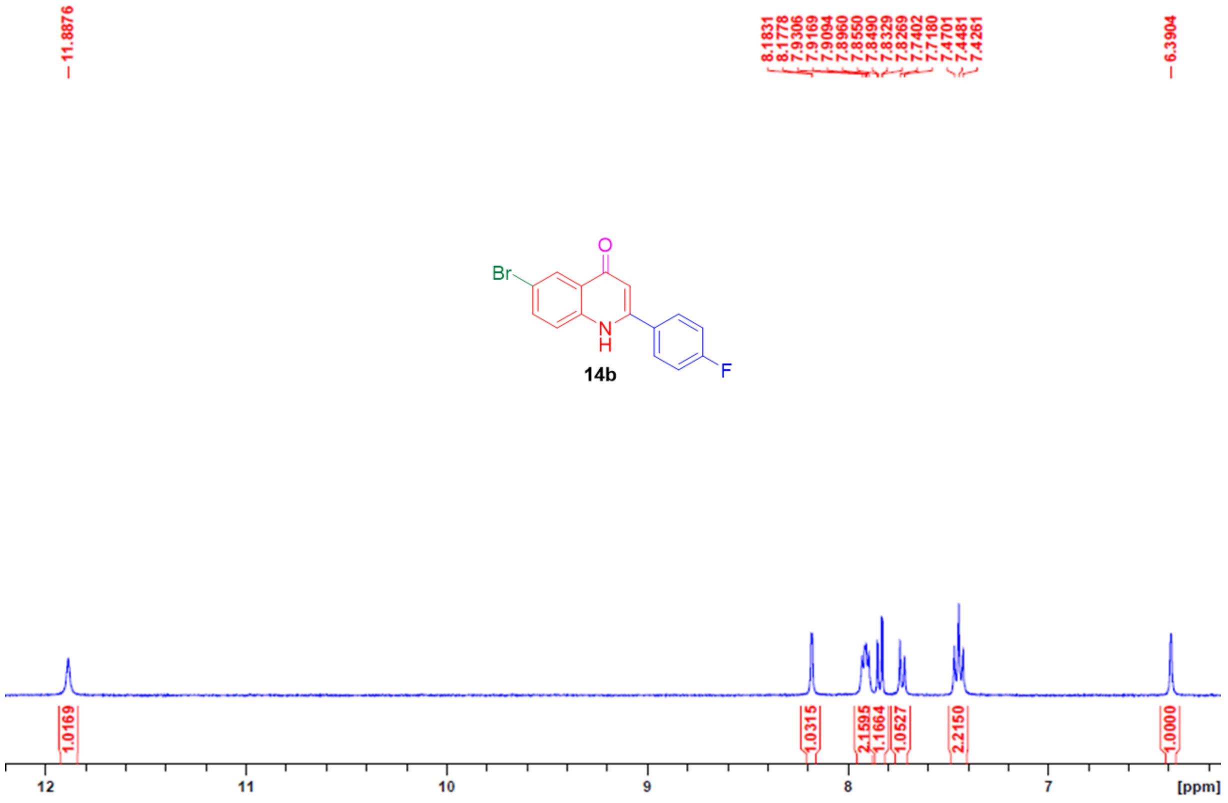
11.8876
8.1831
8.1778
7.9306
7.9169
7.9094
7.8960
7.8550
7.8490
7.8329
7.8269
7.7402
7.7180
7.4701
7.4481
7.4261
6.3904
Br
O
N
H
F
14b
1.0169
1.0315
2.1595
1.1664
1.0527
2.2150
1.0000
12
11
10
9
8
7
[ppm]

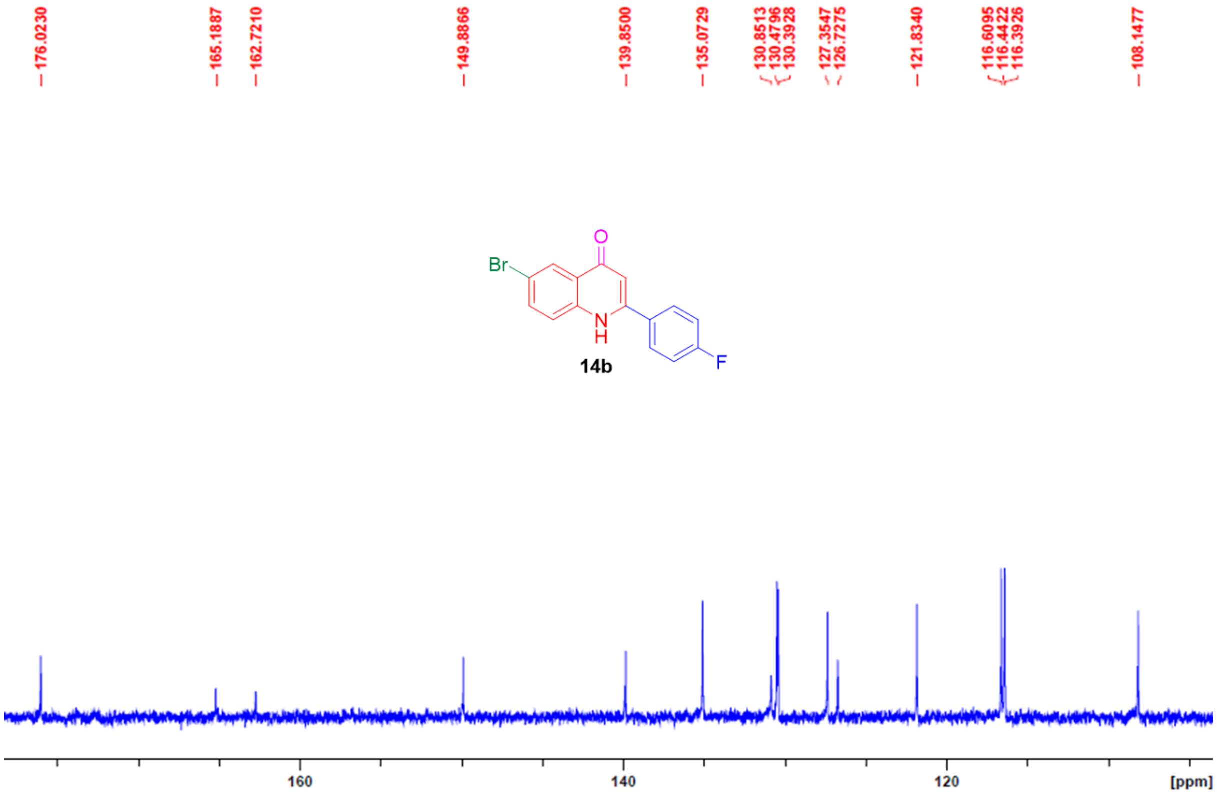
176.0230
165.1887
162.7210
149.8866
139.8500
135.0729
130.8513
130.4796
130.3928
127.3547
126.7275
121.8340
116.6095
116.4422
116.3926
108.1477
Br
O
N
H
F
14b
160
140
120
[ppm]

_19F/DMSO

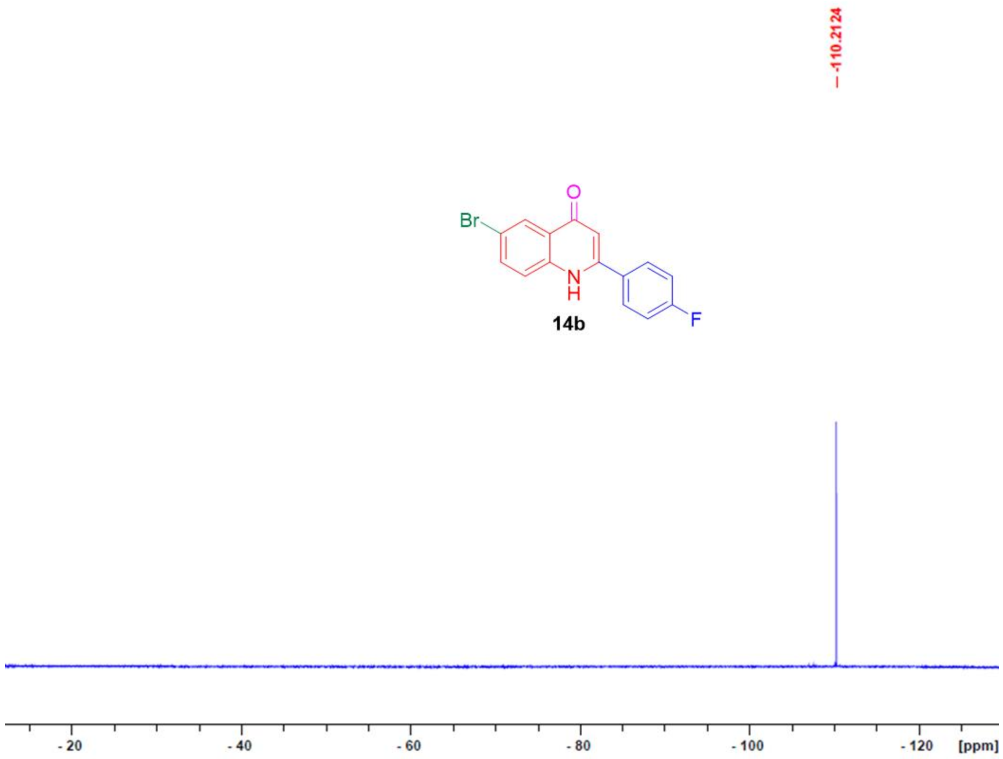
-110.2124
Br
O
N
H
F
14b
- 20
- 40
- 60
- 80
- 100
- 120
[ppm]

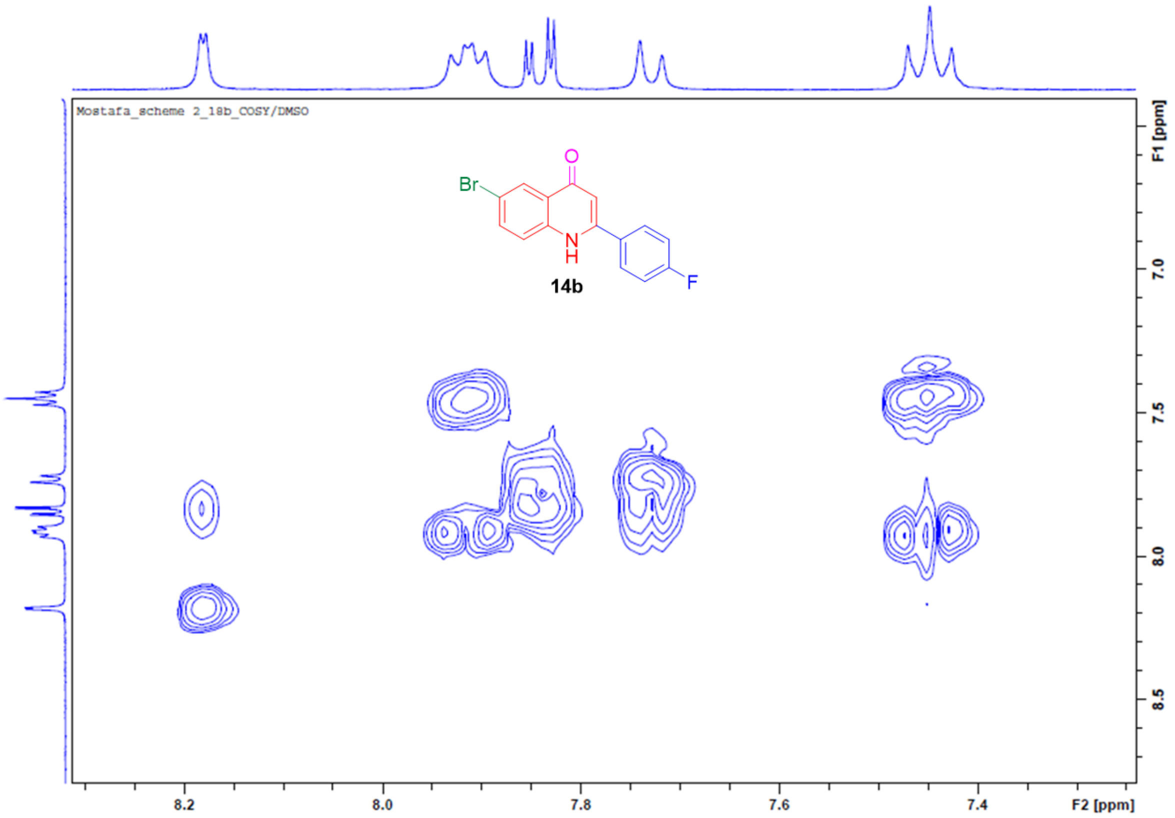
Mostafa_scheme 2_18b_COSY/DMSO
Br
O
N
H
F
14b
F1 [ppm]
7.0
7.5
8.0
8.5
8.2
8.0
7.8
7.6
7.4
F2 [ppm]

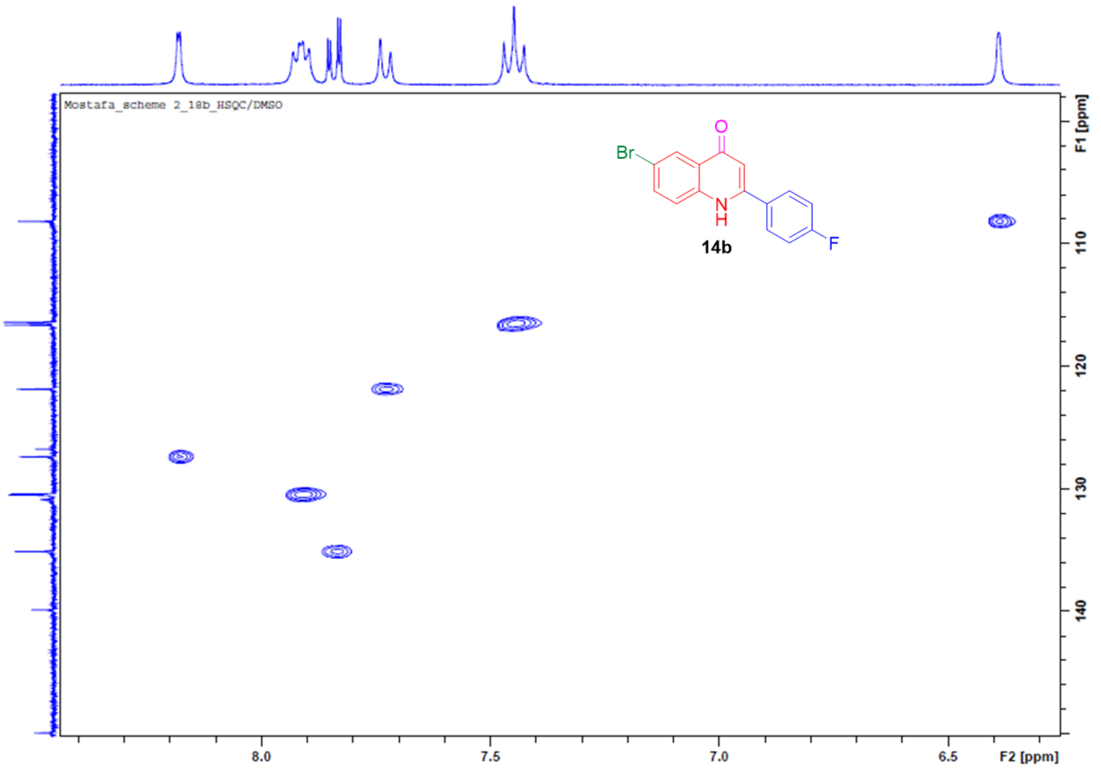
Mostafa_scheme 2_18b_HSQC/DMSO
Br
O
N
H
F
14b
F1 [ppm]
110
120
130
140
8.0
7.5
7.0
6.5
F2 [ppm]

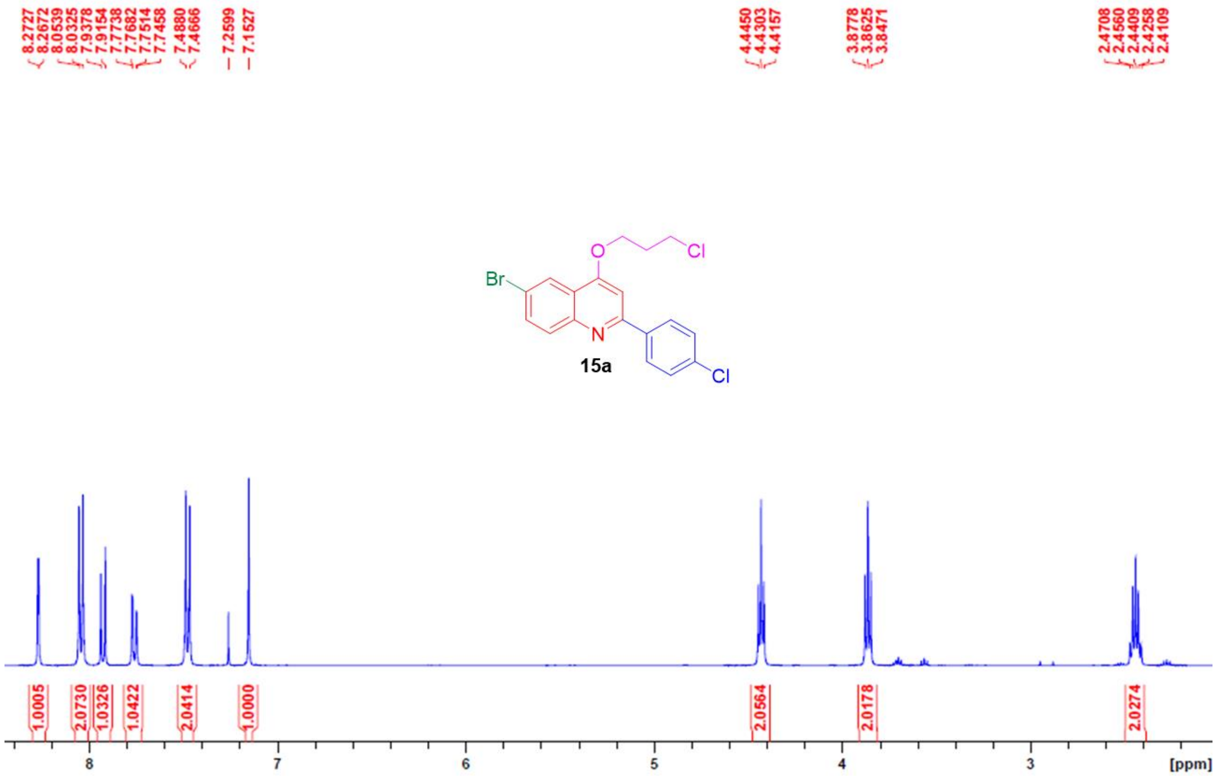
8.2727
8.2672
8.0539
8.0325
7.9378
7.9154
7.7738
7.7682
7.7514
7.7458
7.4880
7.4666
7.2599
7.1527
4.4450
4.4303
4.4157
3.8778
3.8625
3.8471
2.4708
2.4560
2.4409
2.4258
2.4109
Br
O
Cl
N
Cl
15a
1.0005
2.0730
1.0326
1.0422
2.0414
1.0000
2.0564
2.0178
2.0274
[ppm]

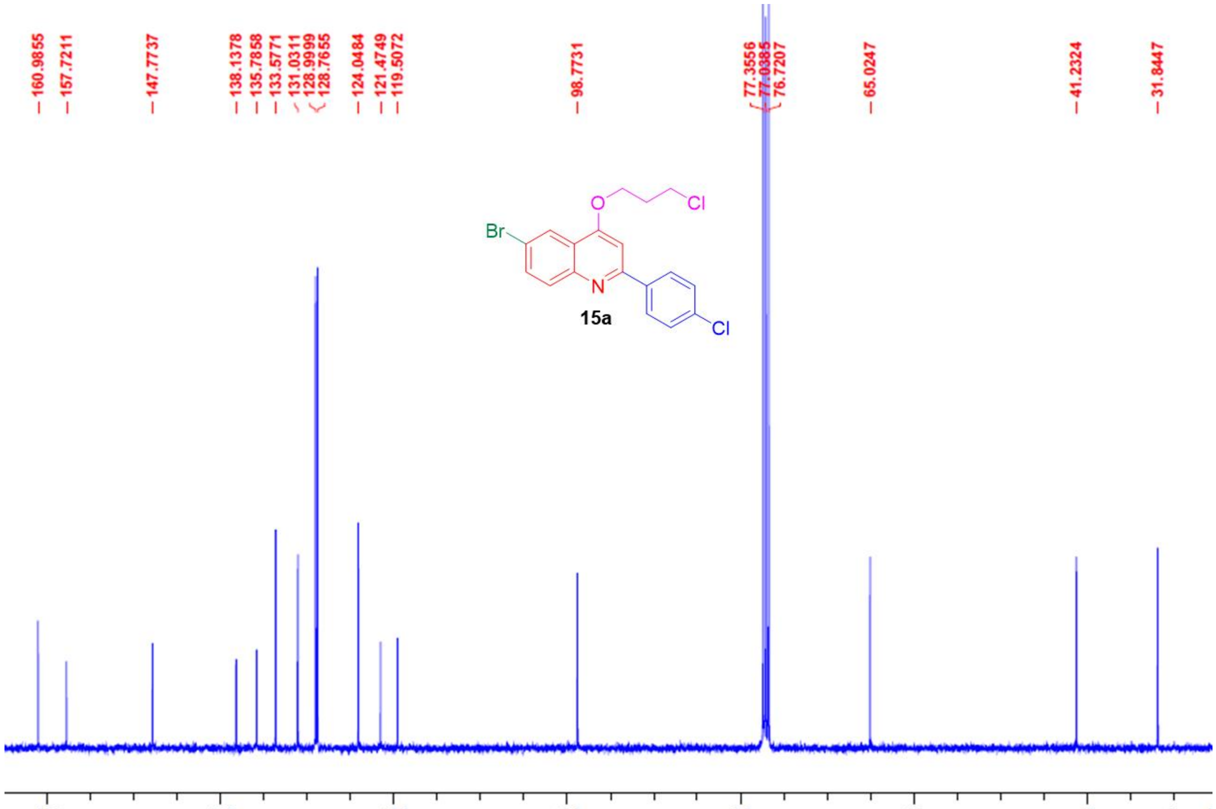
160.9855
157.7211
147.7737
138.1378
135.7858
133.5771
131.0311
128.9999
128.7655
124.0484
121.4749
119.5072
98.7731
77.3556
77.0385
76.7207
65.0247
41.2324
31.8447
Br
O
Cl
N
Cl
15a
[ppm]

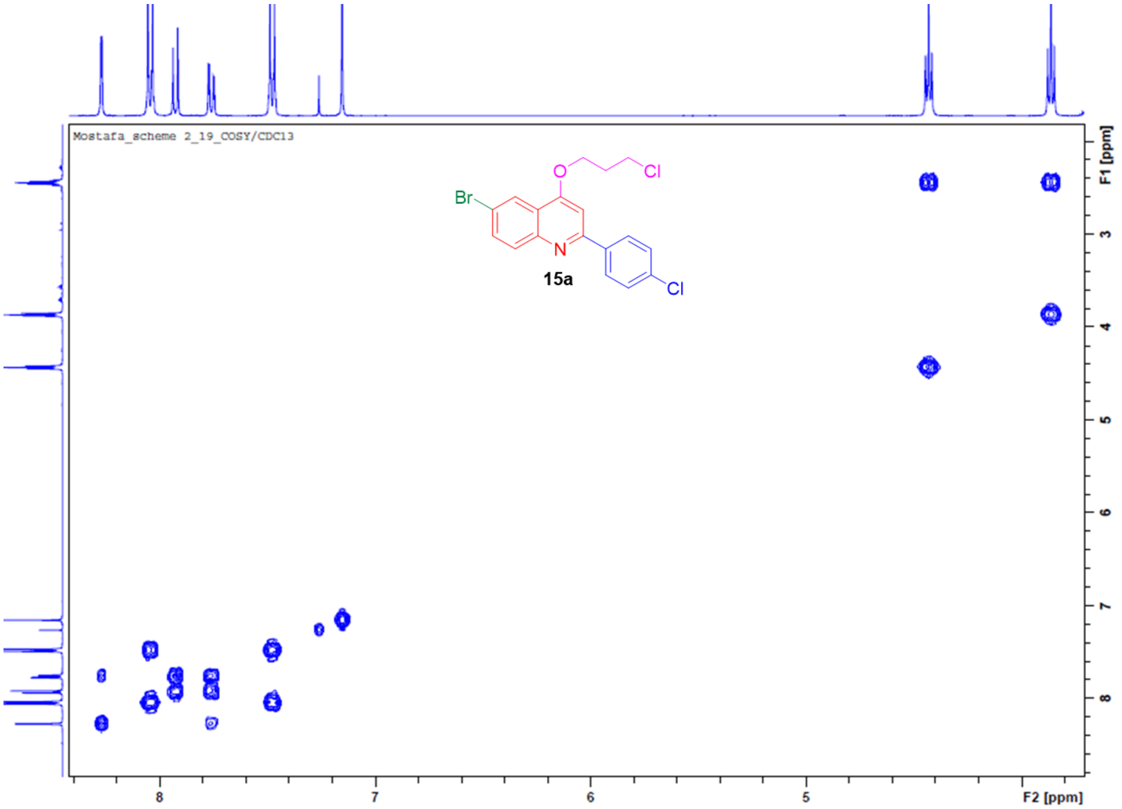
Mostafa_scheme 2_19_COSY/CDCl3
Br
O
Cl
N
Cl
15a
F1 [ppm]
F2 [ppm]

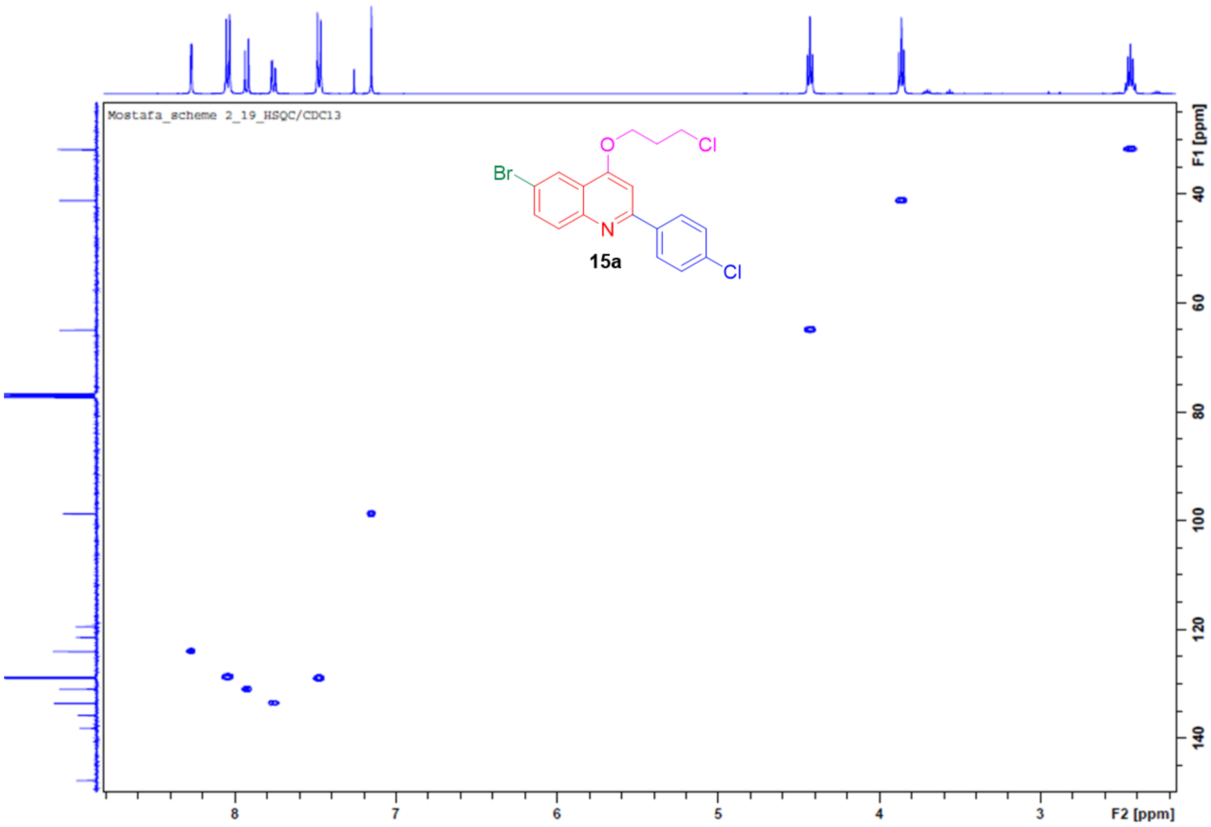
Mostafa_scheme 2_19_HSQC/CDCl3
Br
O
Cl
N
Cl
15a
F1 [ppm]
F2 [ppm]

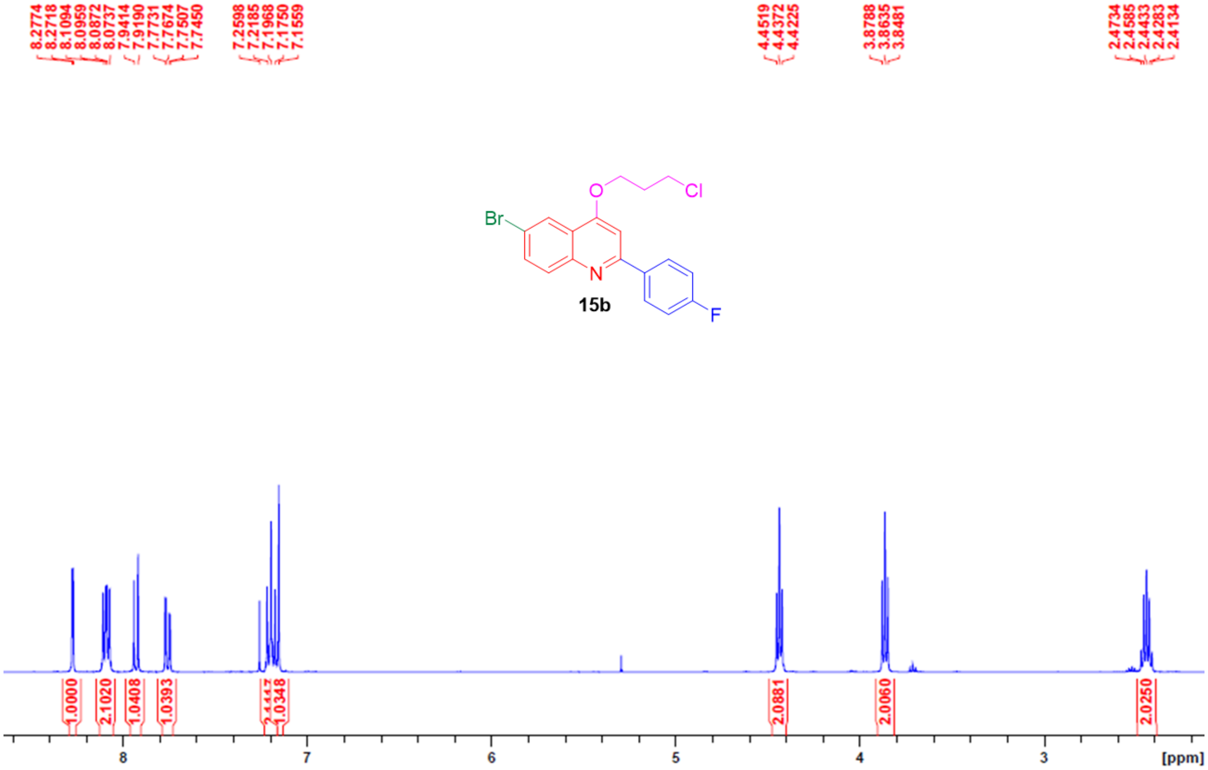
8.2774
8.2718
8.1094
8.0959
8.0872
8.0737
7.9414
7.9190
7.7731
7.7674
7.7507
7.7450
7.2598
7.2185
7.1968
7.1750
7.1559
4.4519
4.4372
4.4225
3.8788
3.8635
3.8481
2.4734
2.4585
2.4433
2.4283
2.4134
Br
O
Cl
N
F
15b
1.0000
2.1020
1.0408
1.0393
1.0348
2.0881
2.0060
2.0250
8
7
6
5
4
3
[ppm]

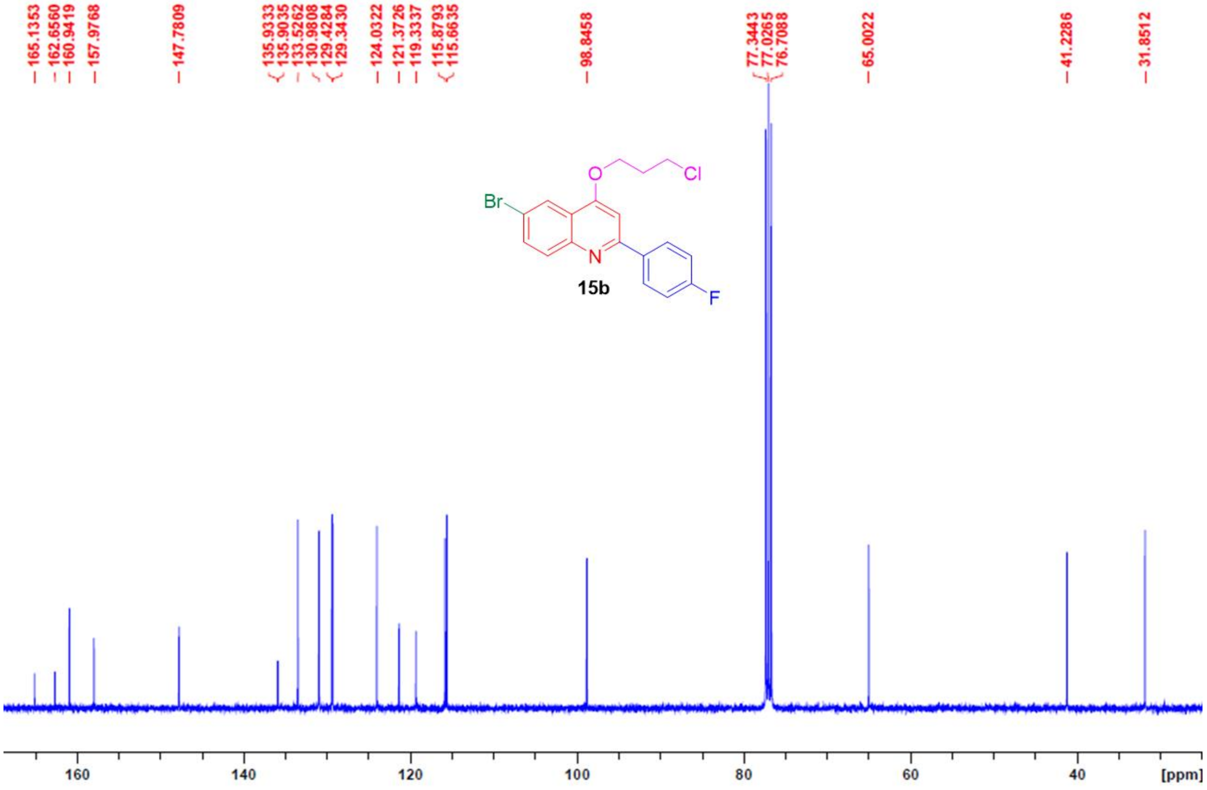
165.1353
162.6560
160.9419
157.9768
147.7809
135.9333
135.9035
133.5262
130.9808
129.4284
129.3430
124.0322
121.3726
119.3337
115.8793
115.6635
98.8458
77.3443
77.0265
76.7088
65.0022
41.2286
31.8512
Br
O
Cl
N
F
15b
160
140
120
100
80
60
40
[ppm]

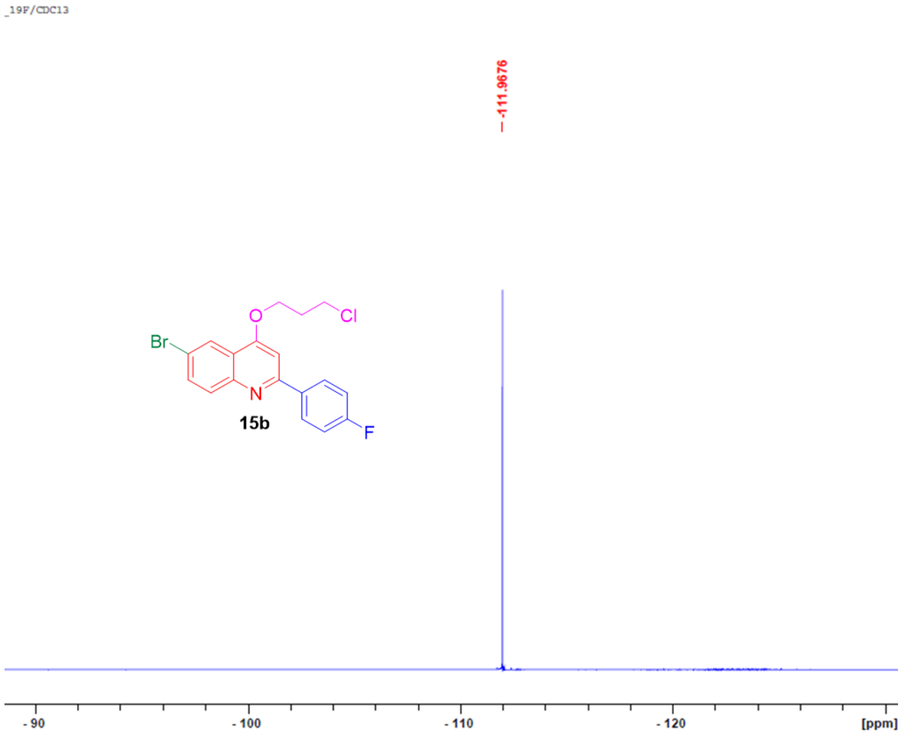
_19F/CDCl3
-111.9676
Br
O
Cl
N
F
15b
- 90
- 100
- 110
- 120
[ppm]

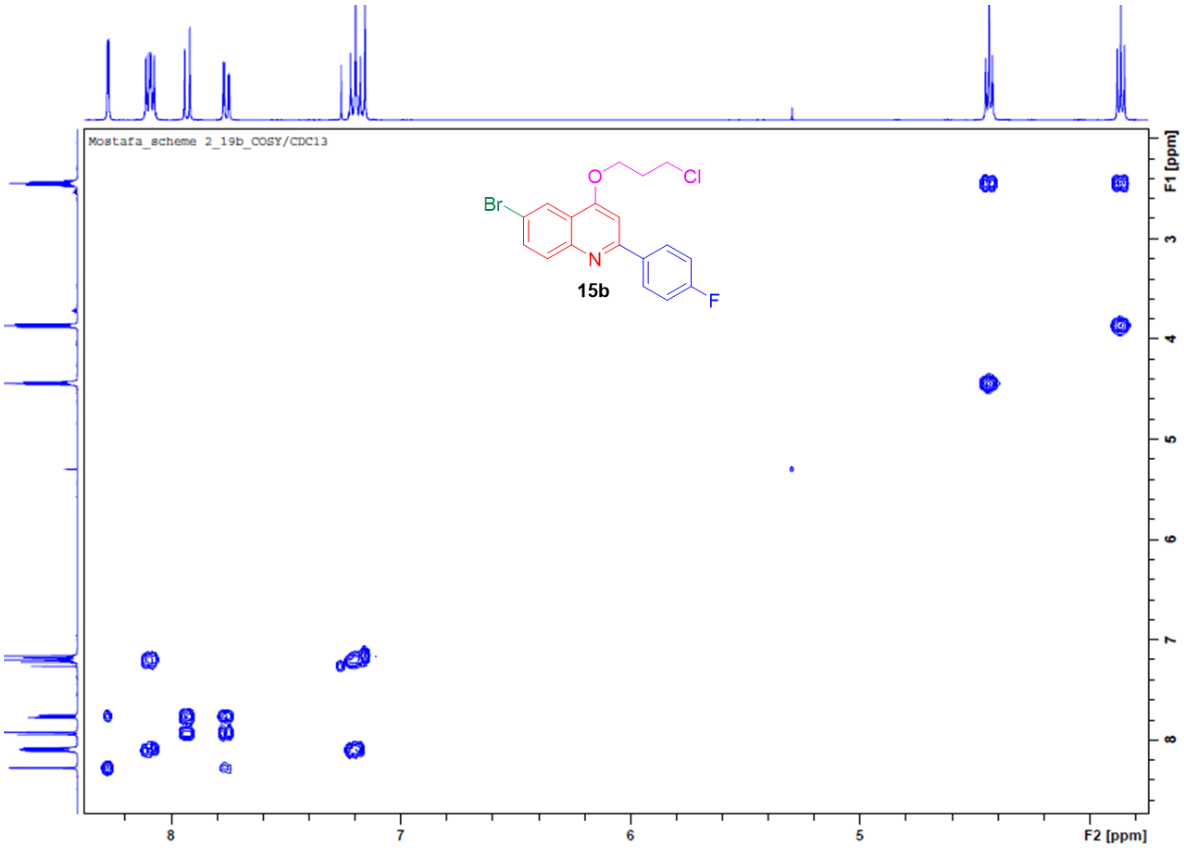
Mostafa_scheme 2_19b_COSY/CDCl3
Br
O
Cl
N
F
15b
F1 [ppm]
F2 [ppm]

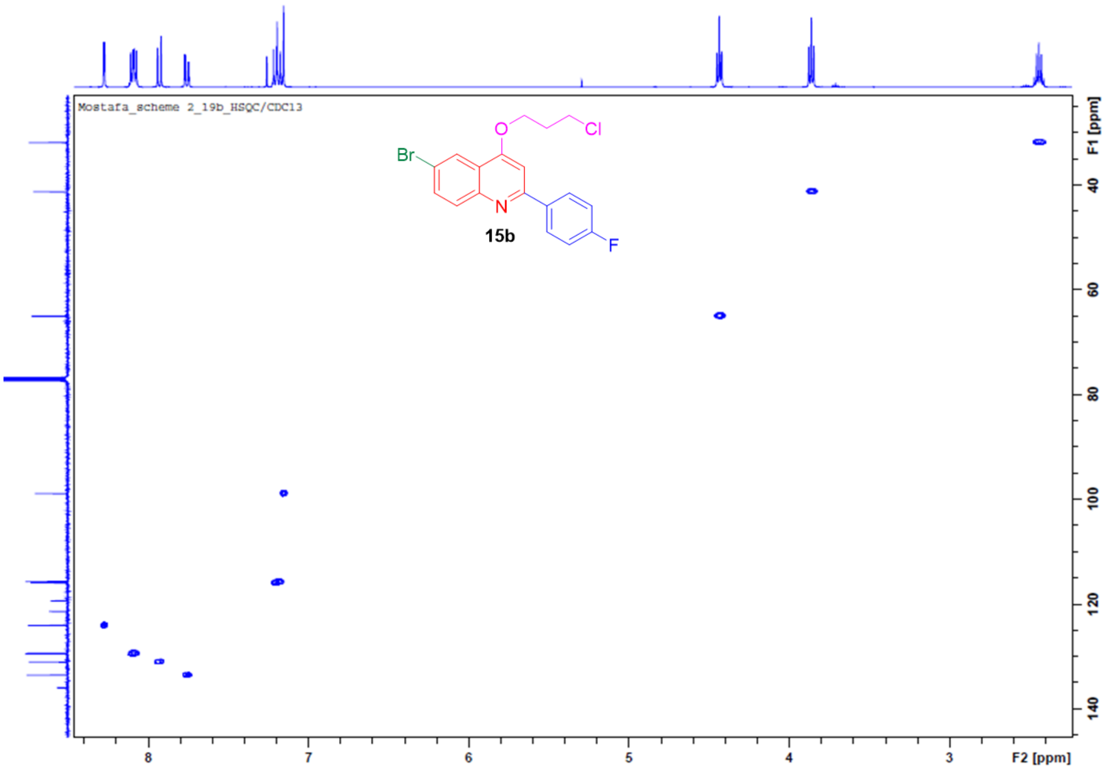
Mostafa_scheme 2_19b_HSQC/CDCl3
Br
O
Cl
N
F
15b
F1 [ppm]
F2 [ppm]

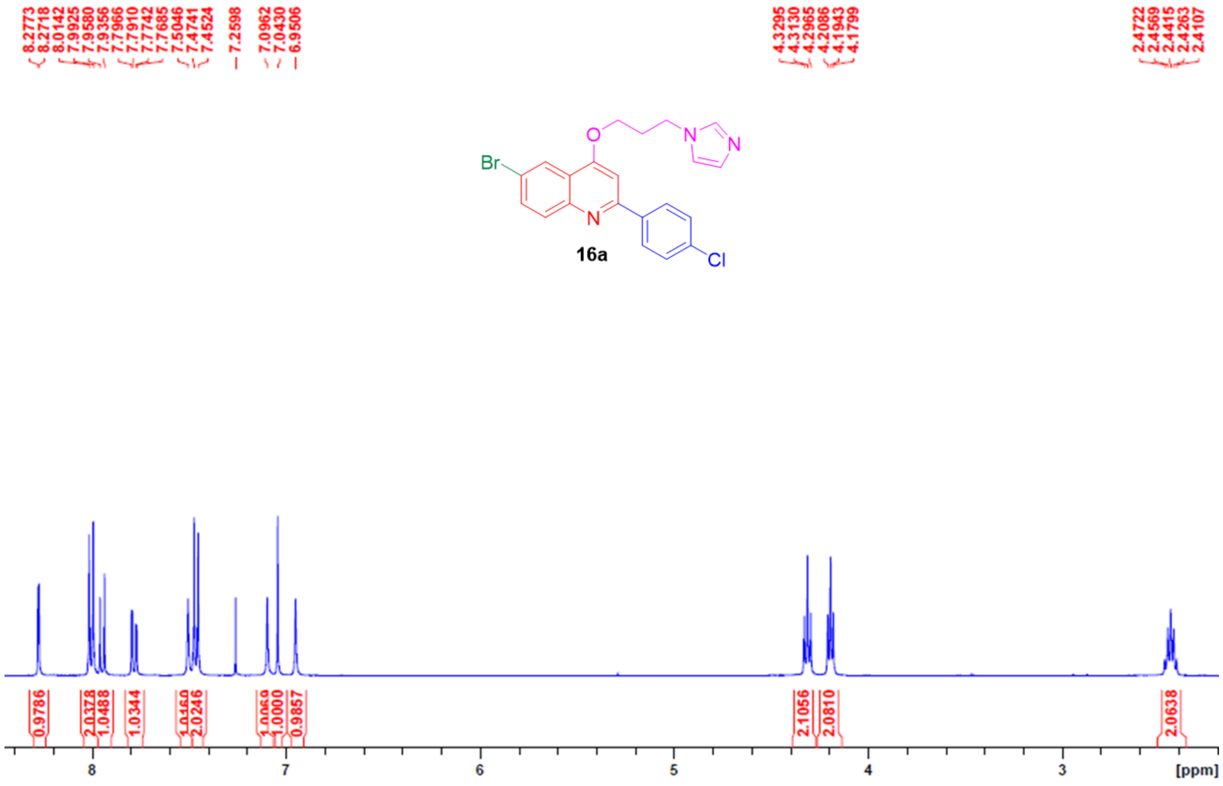
8.2773
8.2718
8.0142
7.9925
7.9580
7.9356
7.7966
7.7910
7.7742
7.7685
7.5046
7.4741
7.4524
7.2598
7.0962
7.0430
6.9506
4.3295
4.3130
4.2965
4.2086
4.1943
4.1799
2.4722
2.4569
2.4415
2.4263
2.4107
Br
O
N
N
N
Cl
16a
0.9786
2.0378
1.0488
1.0344
2.0246
1.0000
0.9857
2.1056
2.0810
2.0638
[ppm]

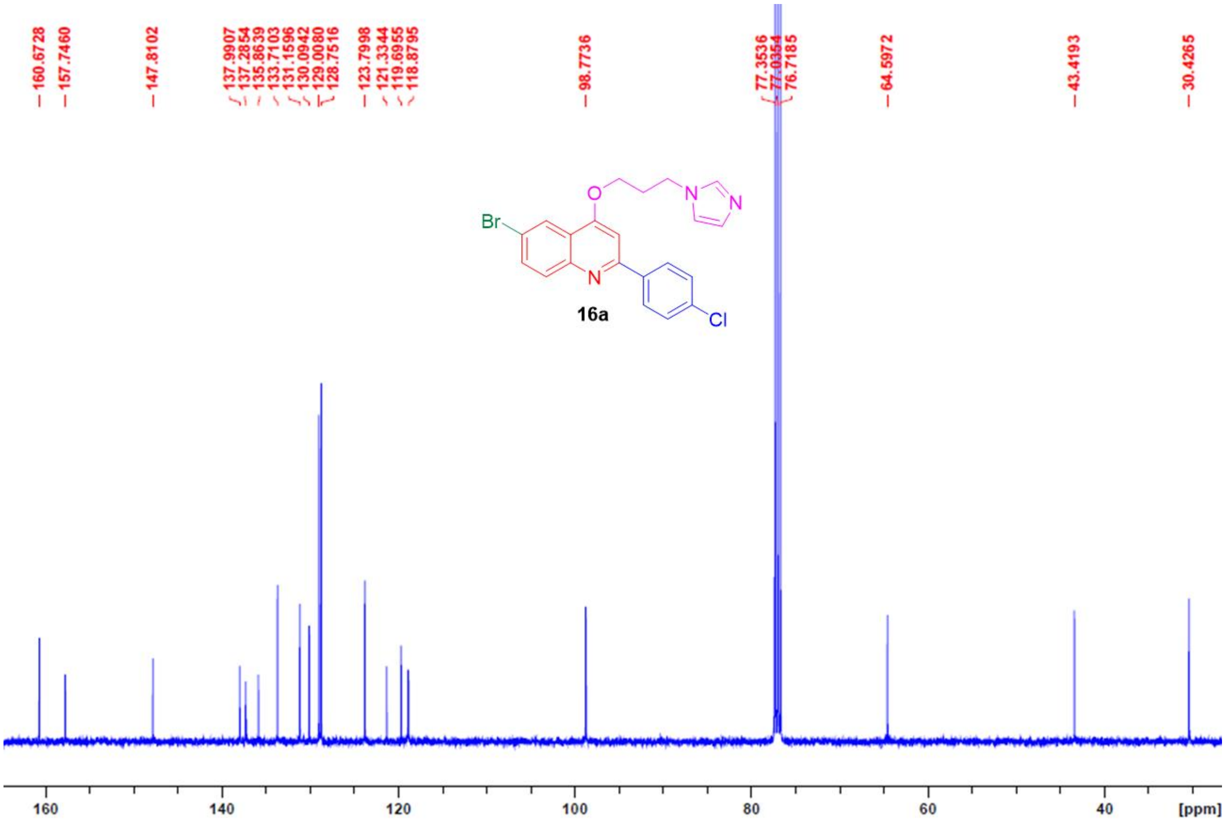
160.6728
157.7460
147.8102
137.9907
137.2854
135.8639
133.7103
131.1596
130.0942
129.0080
128.7516
123.7998
121.3344
119.6955
118.8795
98.7736
77.3536
77.0354
76.7185
64.5972
43.4193
30.4265
Br
O
N
N
N
Cl
16a
[ppm]

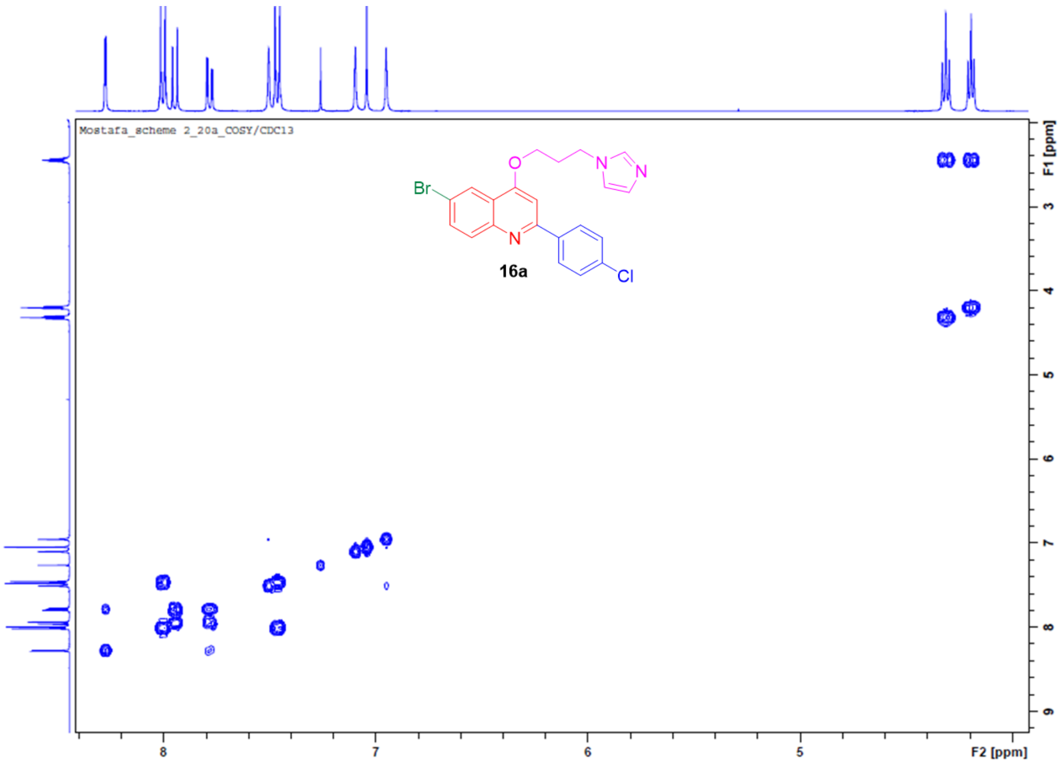
Mostafa_scheme 2_20a_COSY/CDCl3
Br
O
N
N
N
Cl
16a
F1 [ppm]
F2 [ppm]

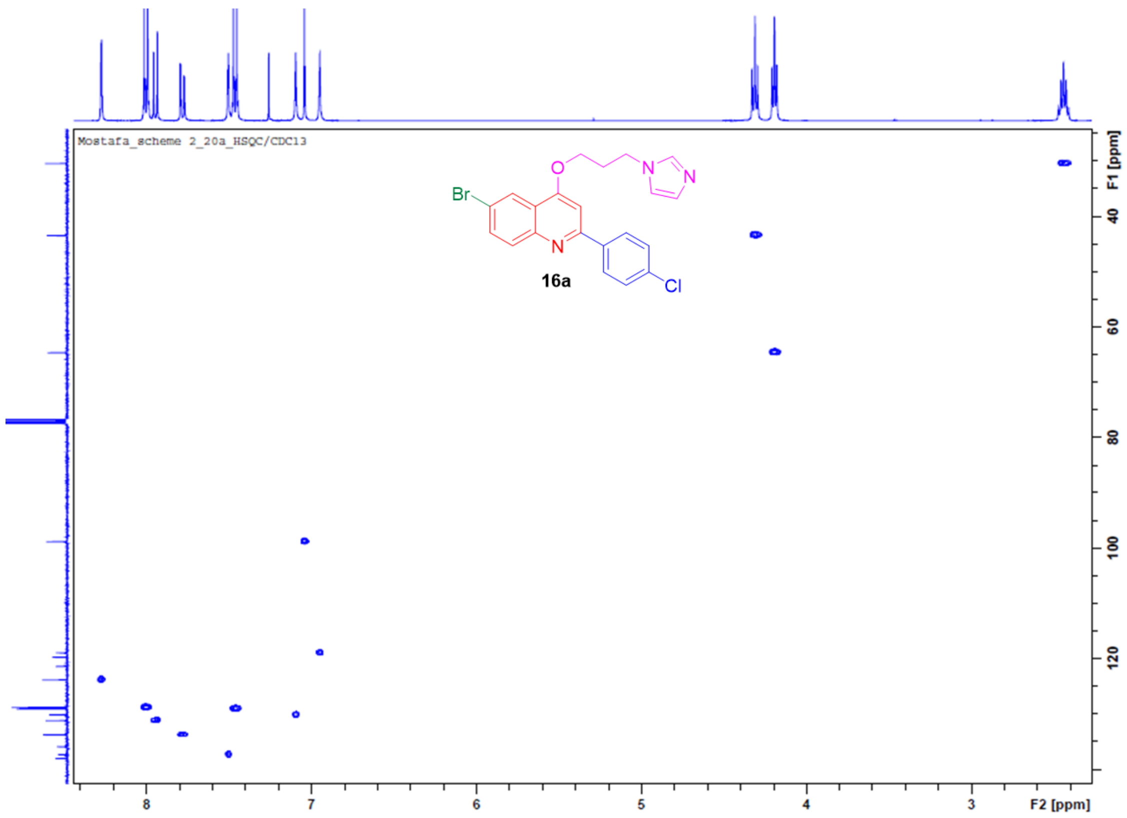
Mostafa_scheme 2_20a_HSQC/CDCl3
Br
O
N
N
N
Cl
16a
F1 [ppm]
F2 [ppm]

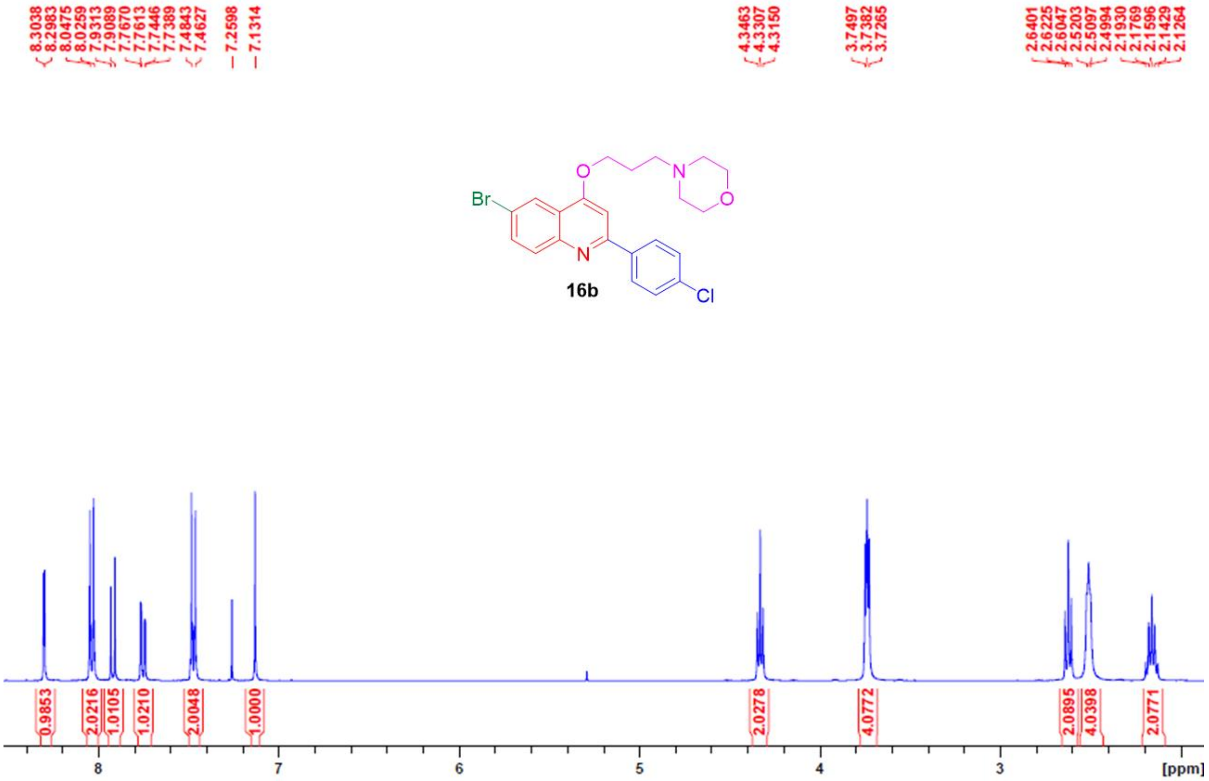
8.3038
8.2983
8.0475
8.0259
7.9313
7.9089
7.7670
7.7613
7.7446
7.7389
7.4843
7.4627
7.2598
7.1314
4.3463
4.3307
4.3150
3.7497
3.7382
3.7265
2.6401
2.6225
2.6047
2.5203
2.5097
2.4994
2.1930
2.1769
2.1596
2.1429
2.1264
Br
O
N
O
N
Cl
16b
0.9853
2.0216
1.0105
1.0210
2.0048
1.0000
2.0278
4.0772
2.0895
4.0398
2.0771
[ppm]

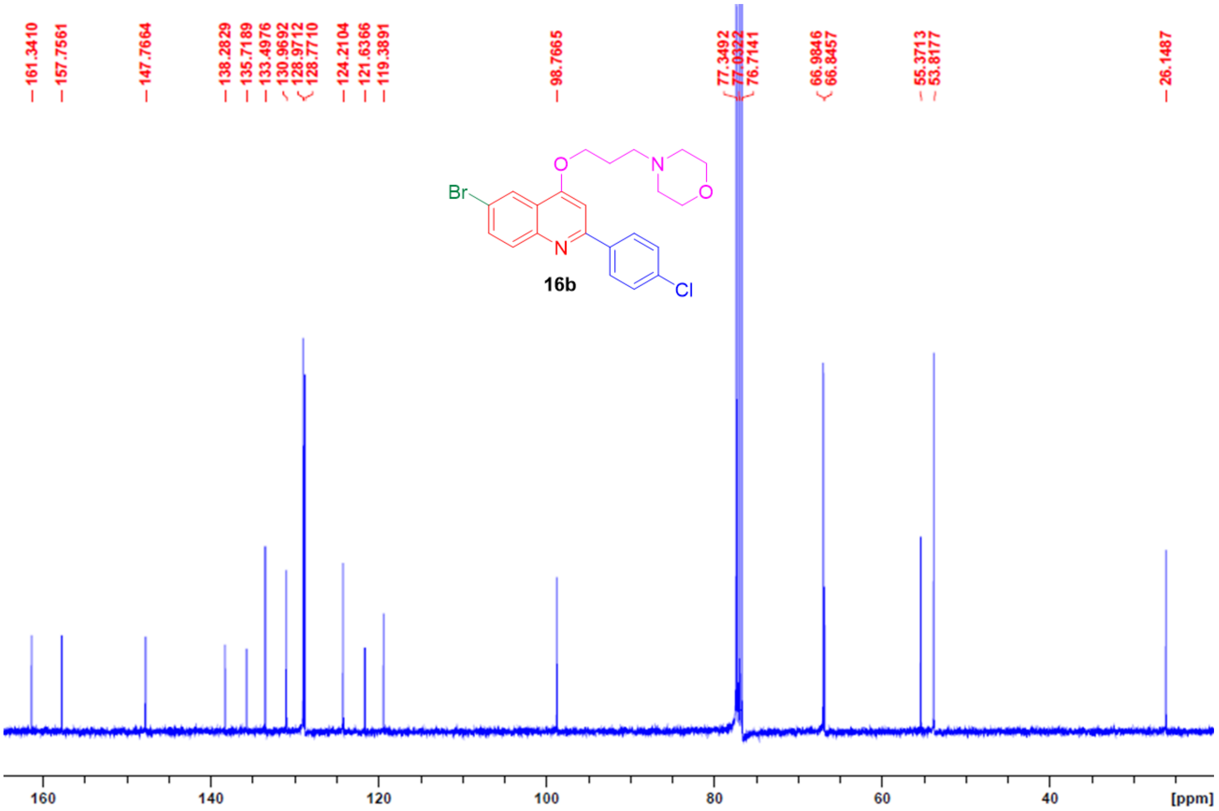
161.3410
157.7561
147.7664
138.2829
135.7189
133.4976
130.9692
128.9712
128.7710
124.2104
121.6366
119.3891
98.7665
77.3492
77.0322
76.7141
66.9846
66.8457
55.3713
53.8177
26.1487
Br
O
N
O
N
Cl
16b
[ppm]

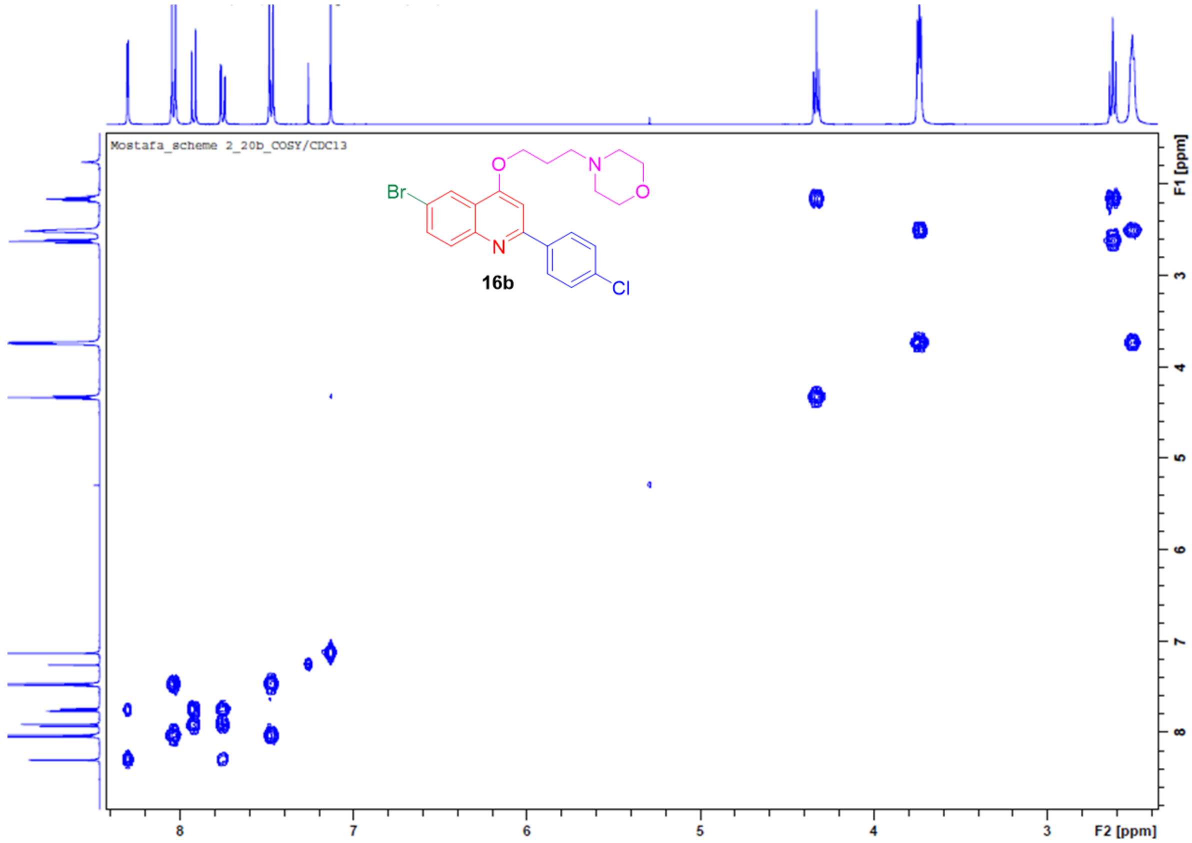
Mostafa_scheme 2_20b_COSY/CDCl3
Br
O
N
O
N
Cl
16b
F1 [ppm]
F2 [ppm]

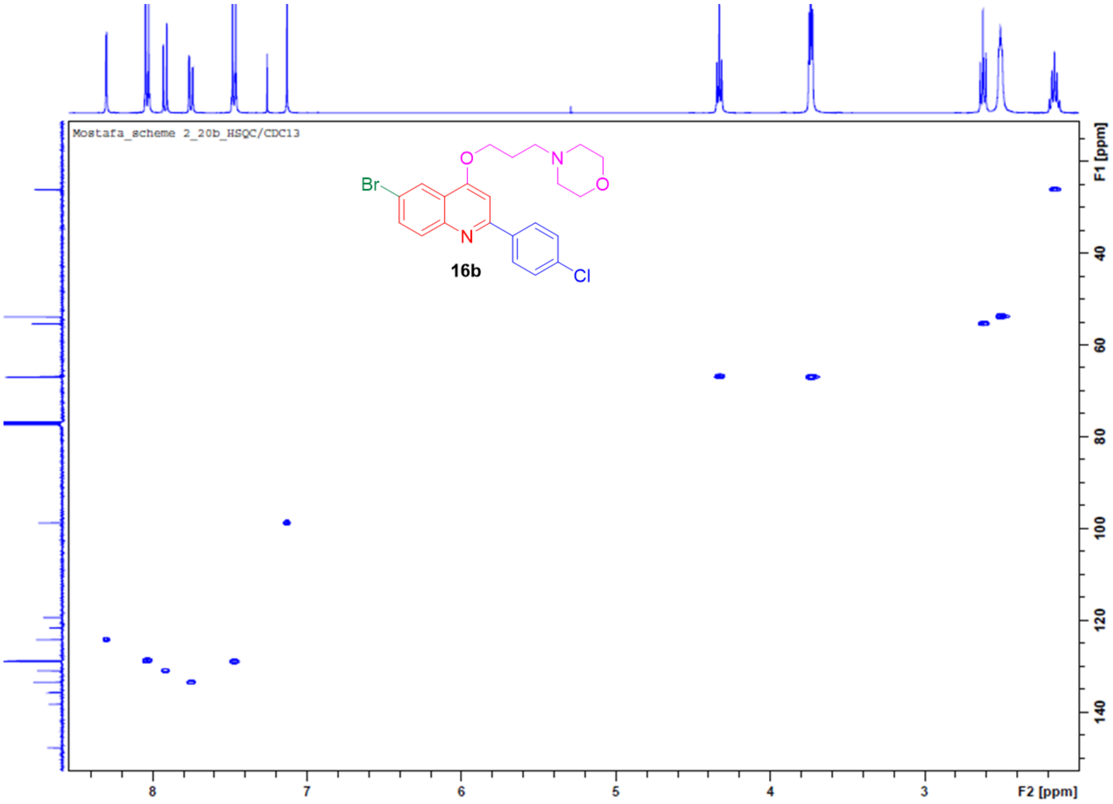
Mostafa_scheme 2_20b_HSQC/CDCl3
Br
O
N
O
N
Cl
16b
F1 [ppm]
40
60
80
100
120
140
8
7
6
5
4
3
F2 [ppm]

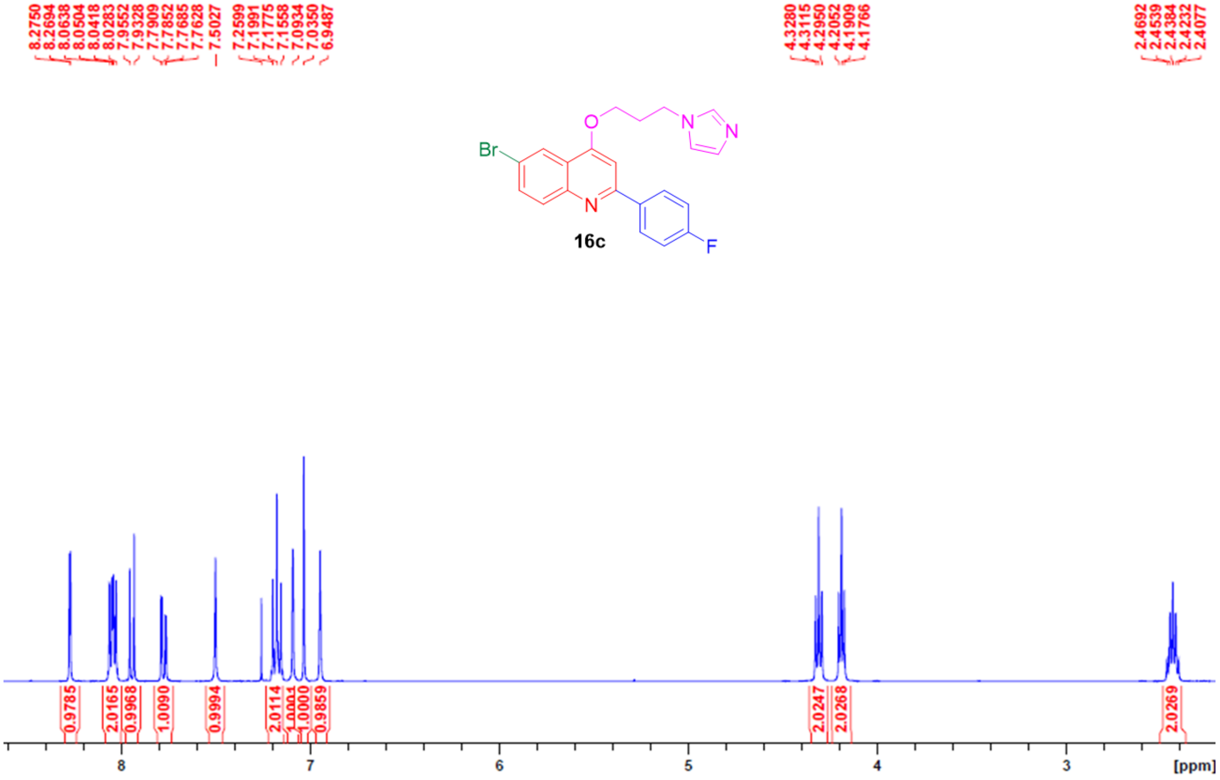
8.2750
8.2694
8.0638
8.0504
8.0418
8.0283
7.9552
7.9328
7.7909
7.7852
7.7685
7.7628
7.5027
7.2599
7.1991
7.1775
7.1558
7.0934
7.0350
6.9487
4.3280
4.3115
4.2950
4.2052
4.1909
4.1766
2.4692
2.4539
2.4384
2.4232
2.4077
Br
O
N
N
N
F
16c
0.9785
2.0165
0.9968
1.0090
0.9994
2.0114
1.0001
1.0000
0.9859
2.0247
2.0268
2.0269
8
7
6
5
4
3
[ppm]

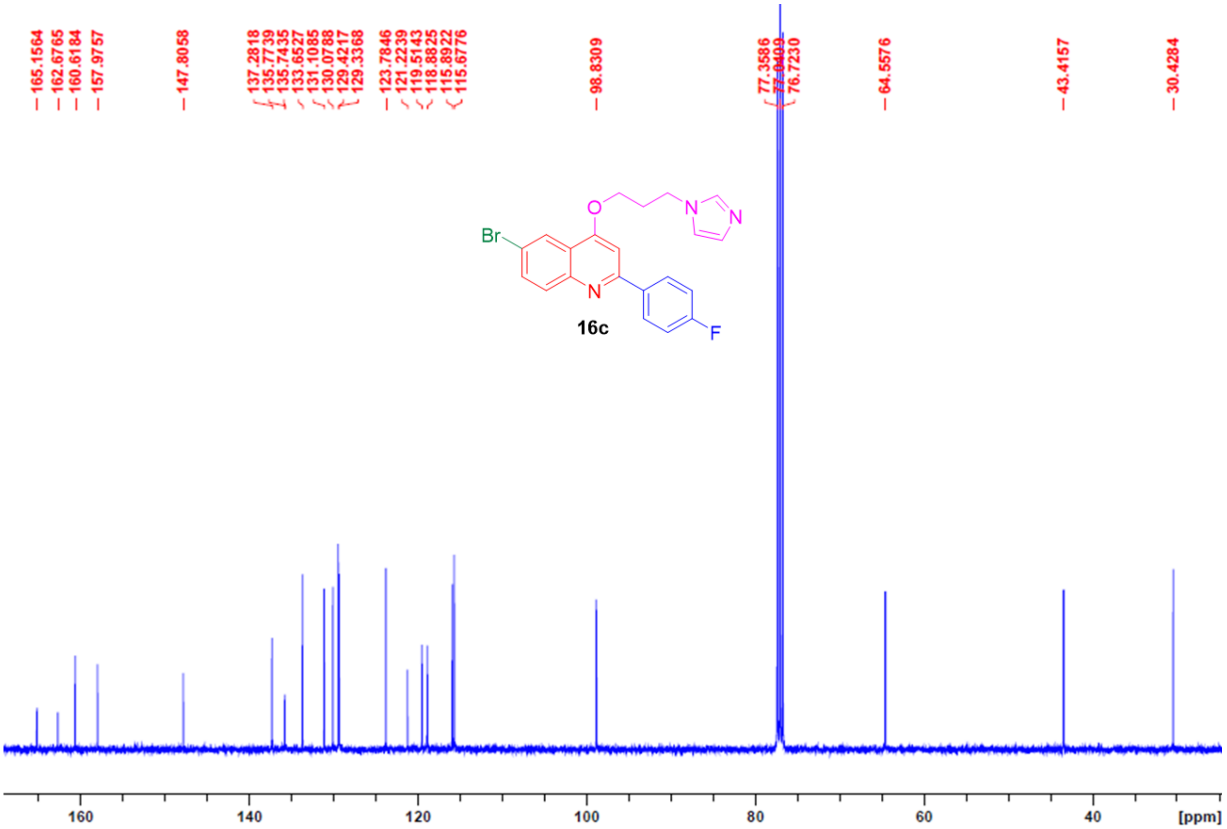

165.1564
162.6765
160.6184
157.9757
147.8058
137.2818
135.7739
135.7435
133.6527
131.1085
130.0788
129.4217
129.3368
123.7846
121.2239
119.5143
118.8825
115.8922
115.6776
98.8309
77.3586
76.7230
64.5576
43.4157
30.4284
Br
O
N
N
N
F
16c
160
140
120
100
80
60
40
[ppm]

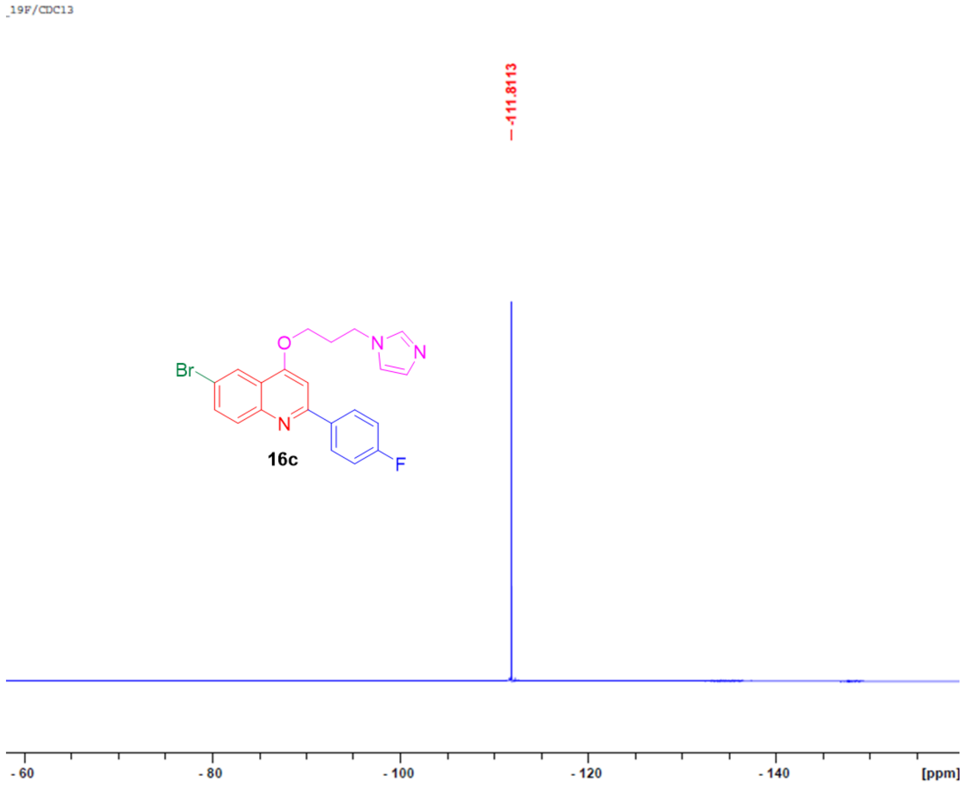

_19F/CDCl3
-111.8113
Br
O
N
N
N
F
16c
-60
-80
-100
-120
-140
[ppm]

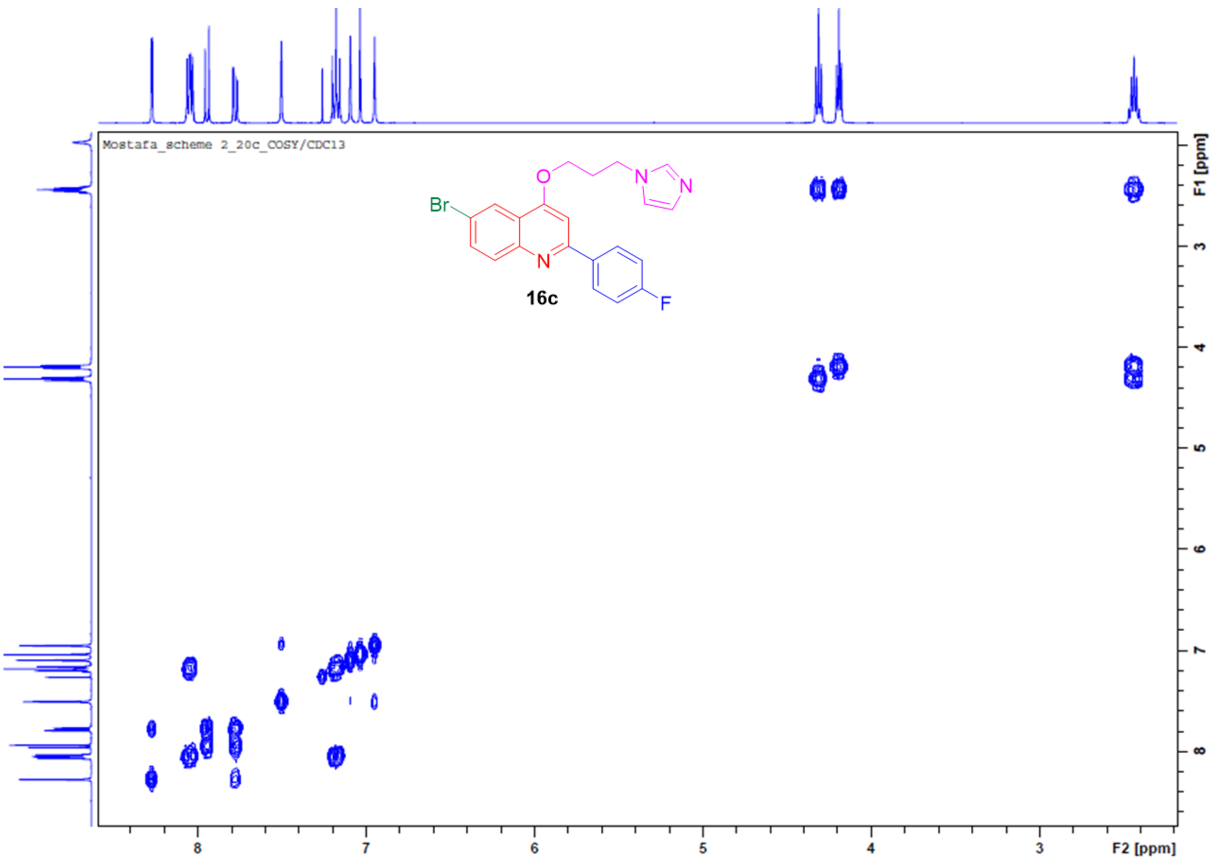
Mostafa_scheme 2_20c_COSY/CDCl3
Br
O
N
N
N
F
16c
F1 [ppm]
F2 [ppm]

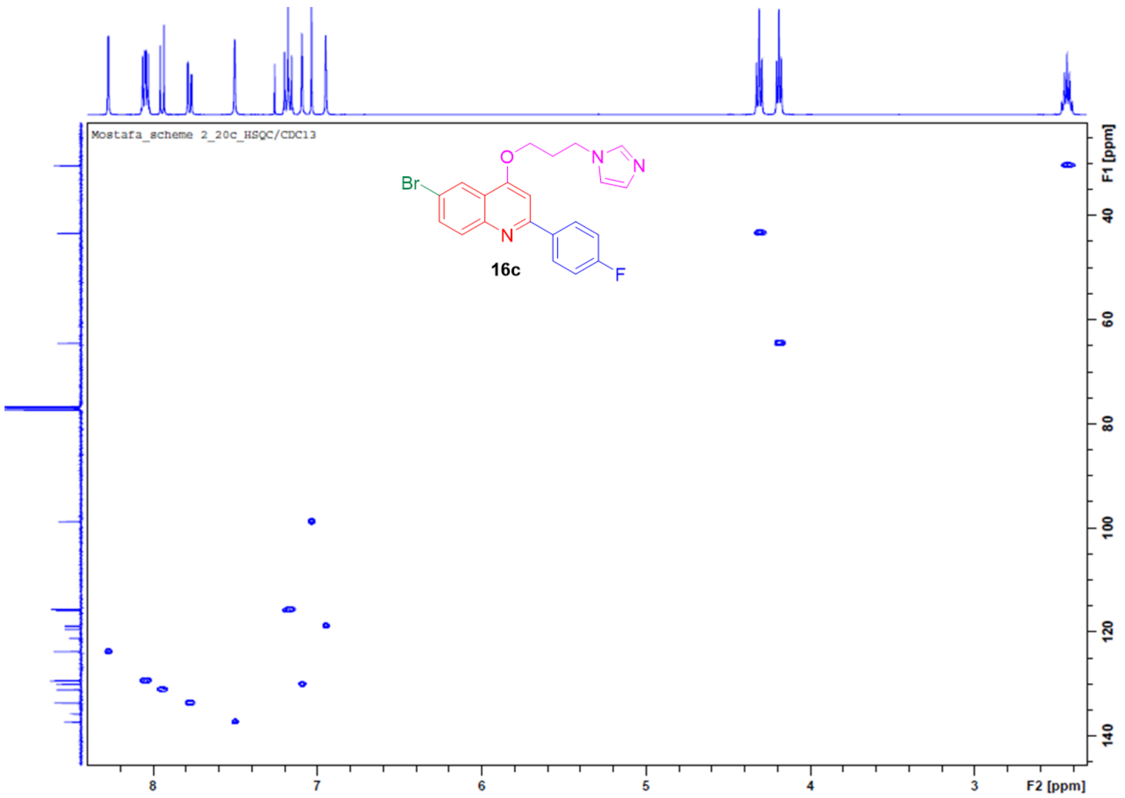
Mostafa_scheme 2_20c_HSQC/CDCl3
Br
O
N
N
N
F
16c
F1 [ppm]
F2 [ppm]

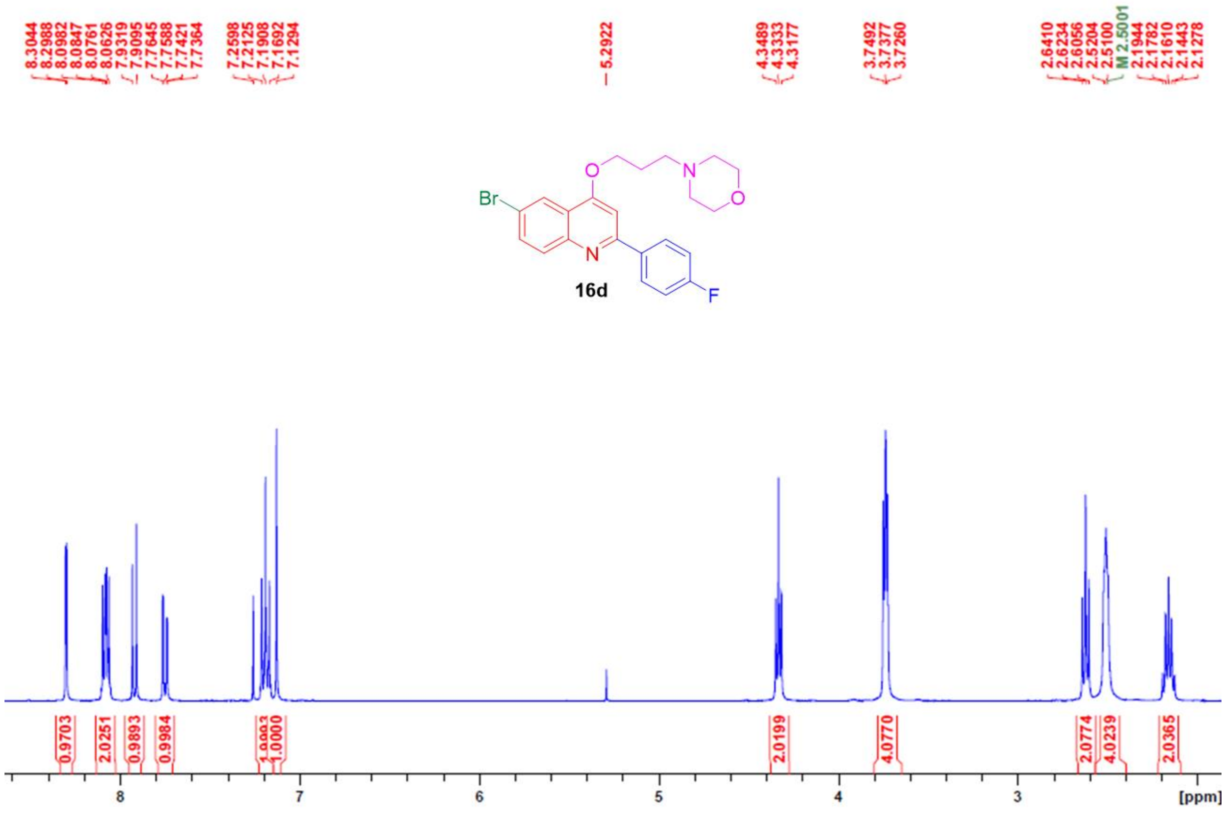
8.3044
8.2988
8.0982
8.0847
8.0761
8.0626
7.9319
7.9095
7.7645
7.7588
7.7421
7.7364
7.2598
7.2125
7.1908
7.1692
7.1294
5.2922
4.3489
4.3333
4.3177
3.7492
3.7377
3.7260
2.6410
2.6234
2.6056
2.5204
2.5100
M 2.5001
2.1944
2.1782
2.1610
2.1443
2.1278
Br
O
N
O
N
F
16d
0.9703
2.0251
0.9893
0.9984
1.0000
2.0199
4.0770
2.0774
4.0239
2.0365
[ppm]

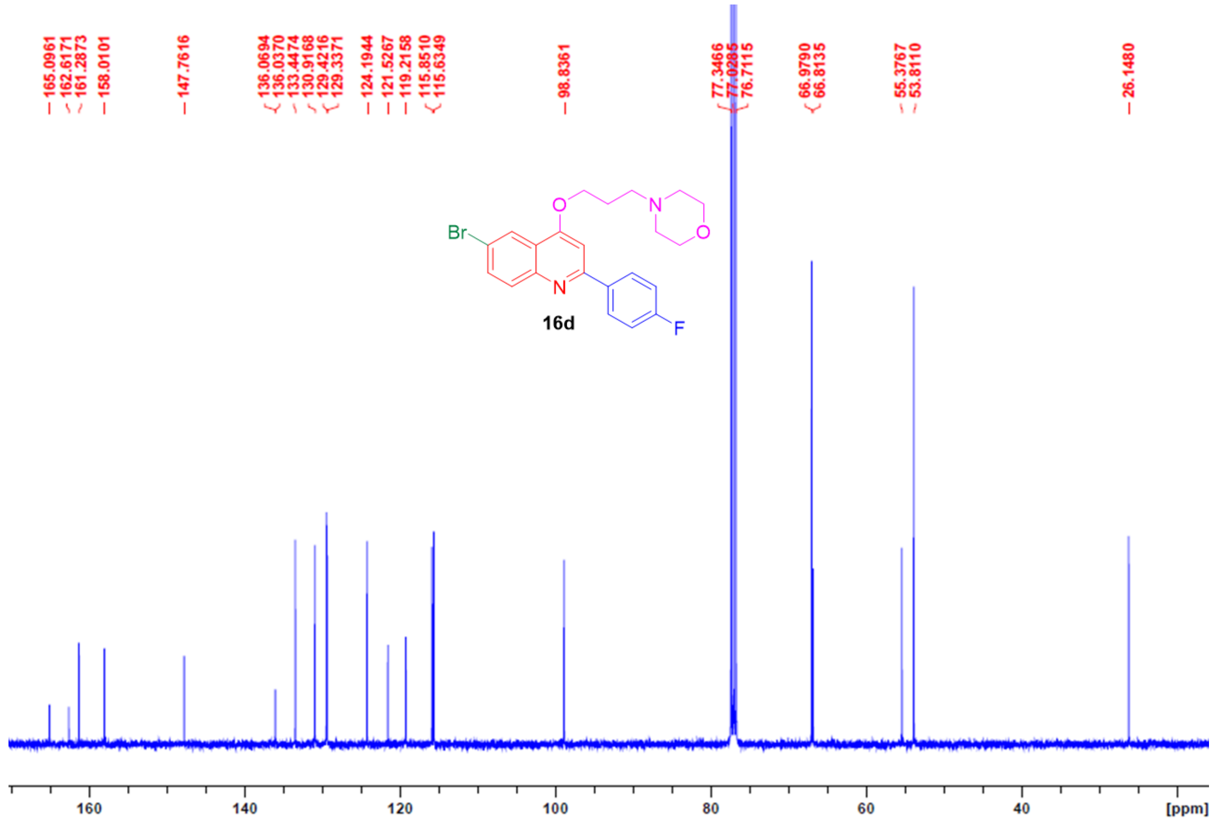
165.0961
162.6171
161.2873
158.0101
147.7616
136.0694
136.0370
133.4474
130.9168
129.4216
129.3371
124.1944
121.5267
119.2158
115.8510
115.6349
98.8361
77.3466
76.7115
66.9790
66.8135
55.3767
53.8110
26.1480
Br
O
N
O
N
F
16d
[ppm]

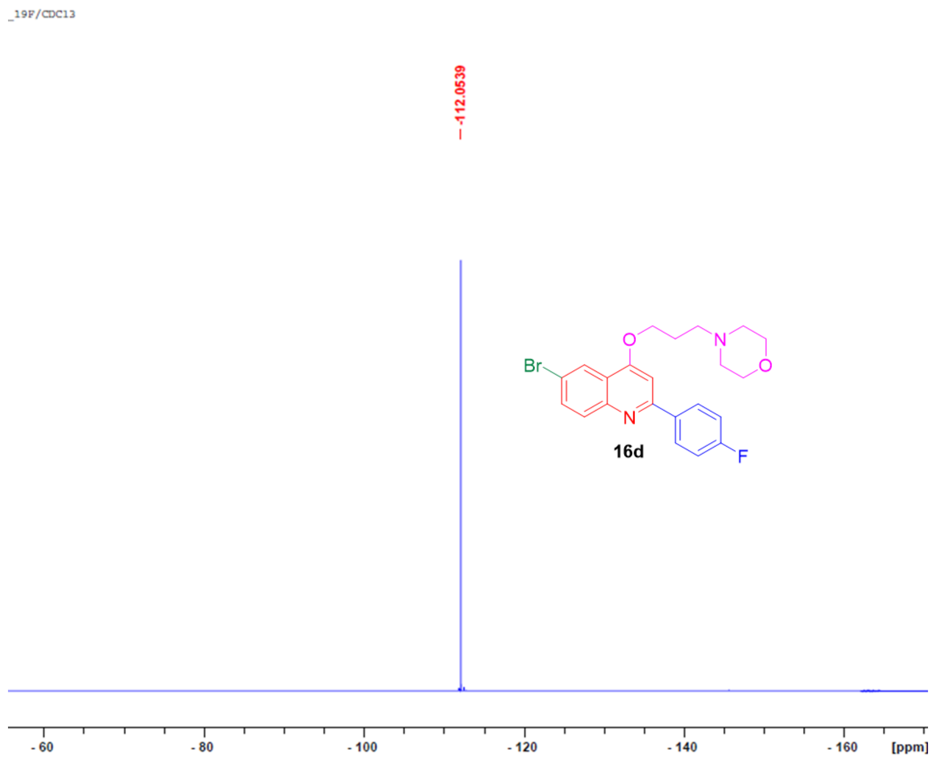
_19F/CDCl3
-112.0539
Br
O
N
O
N
16d
F
-60
-80
-100
-120
-140
-160
[ppm]

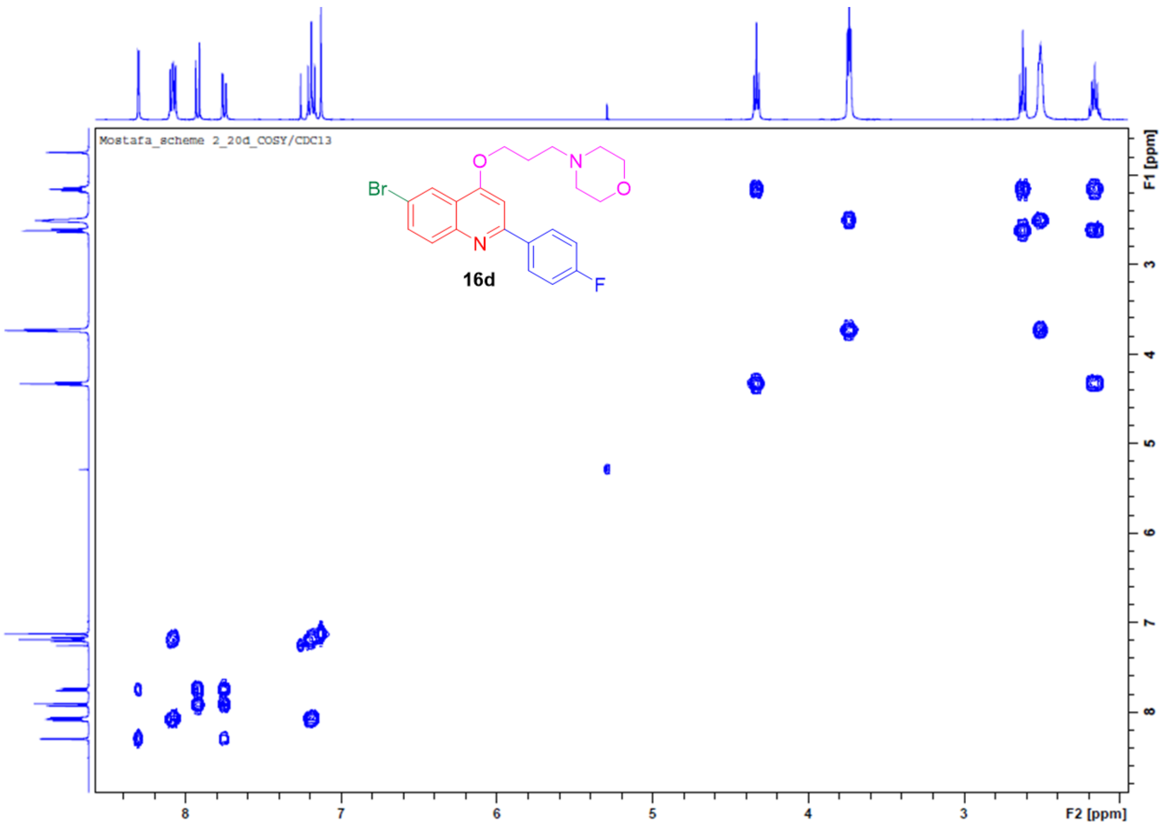
Mostafa_scheme 2_20d_COSY/CDCl3
Br
O
N
O
N
16d
F
F1 [ppm]
F2 [ppm]

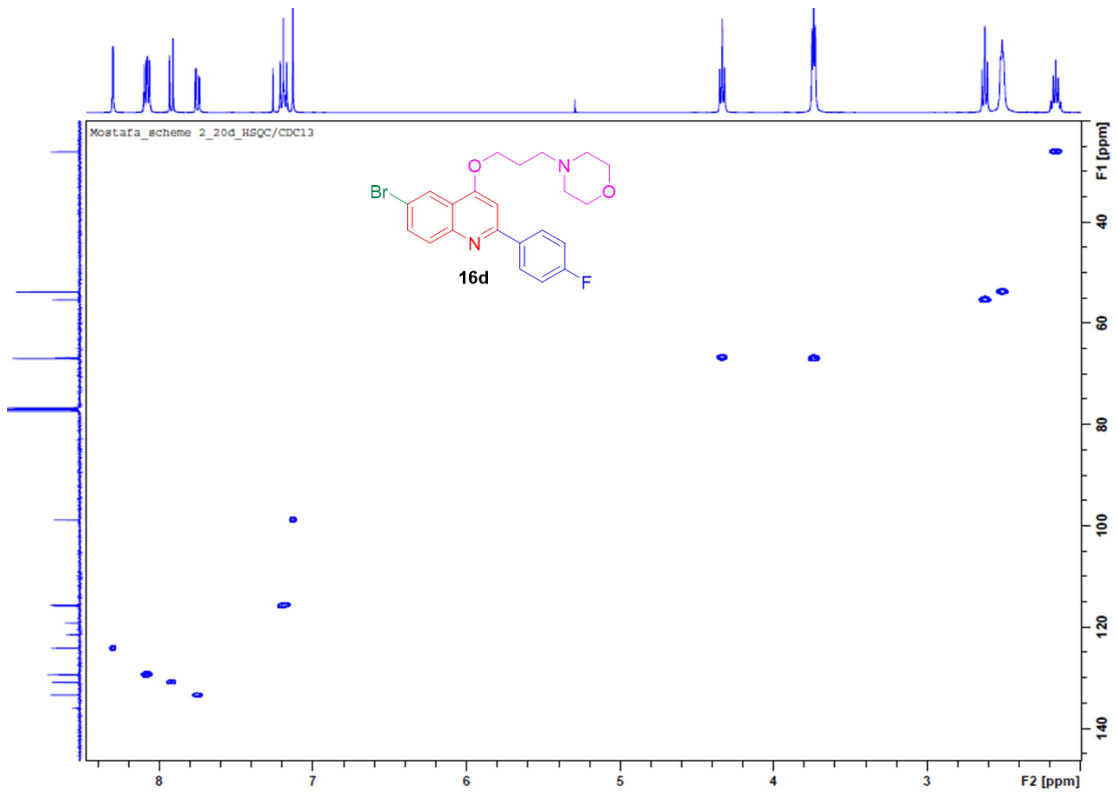
Mostafa_scheme 2_20d_HSQC/CDCl3
Br
O
N
O
N
F
16d
F1 [ppm]
F2 [ppm]

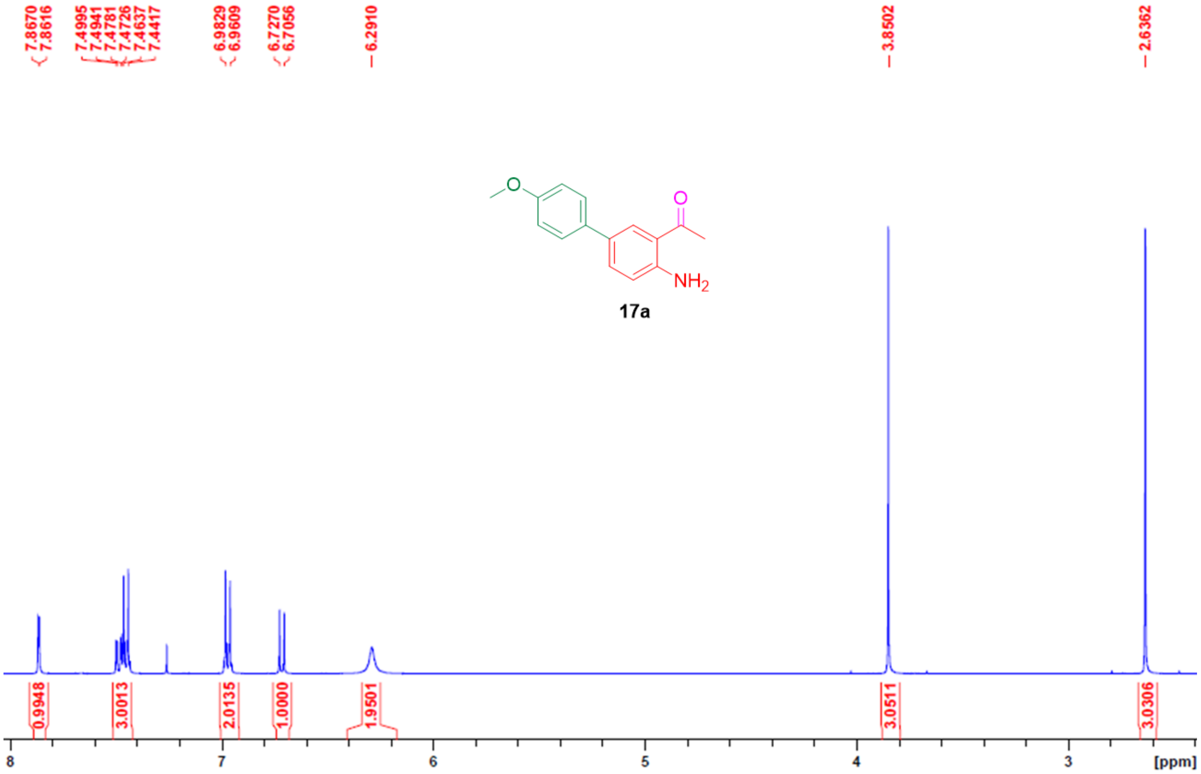
7.8670
7.8616
7.4995
7.4941
7.4781
7.4726
7.4637
7.4417
6.9829
6.9609
6.7270
6.7056
6.2910
3.8502
2.6362
O
O
NH2
17a
0.9948
3.0013
2.0135
1.0000
1.9501
3.0511
3.0306
[ppm]

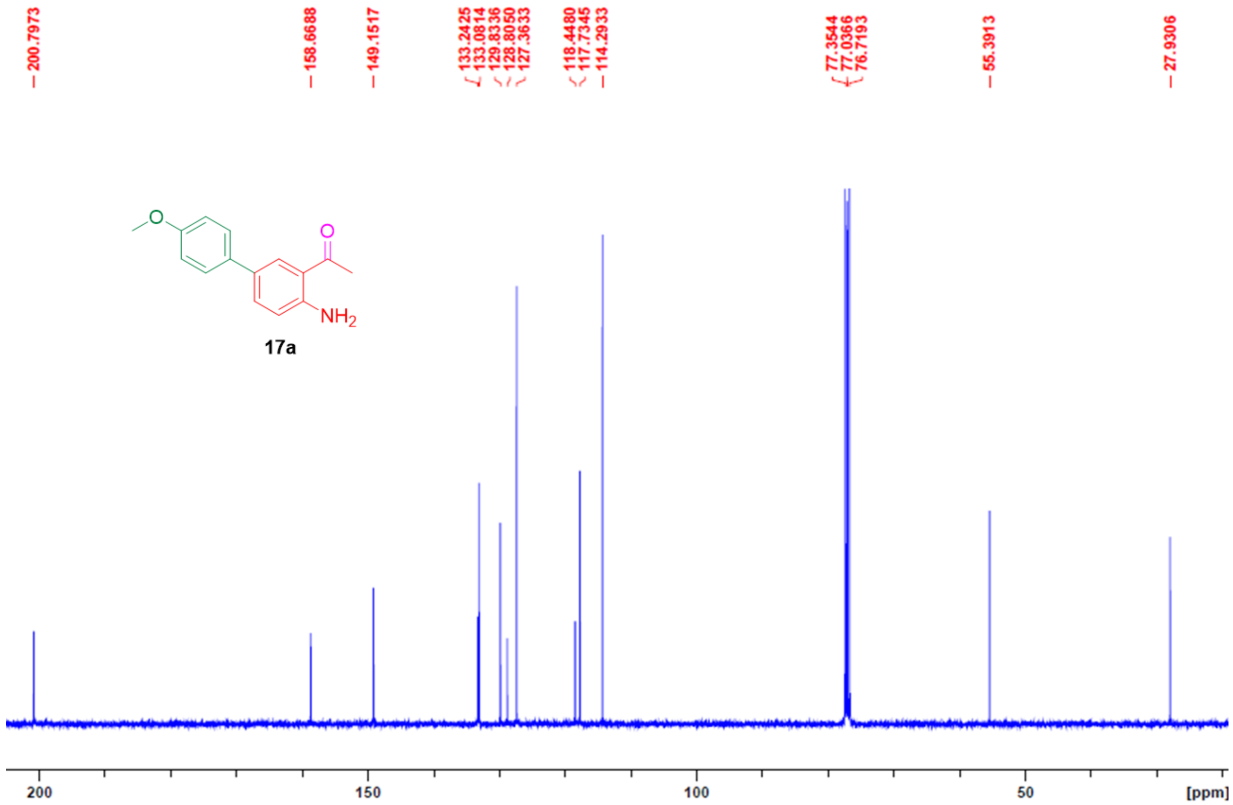
200.7973
158.6688
149.1517
133.2425
133.0814
129.8336
128.8050
127.3633
118.4480
117.7345
114.2933
77.3544
77.0366
76.7193
55.3913
27.9306
O
O
NH2
17a
200
150
100
50
[ppm]

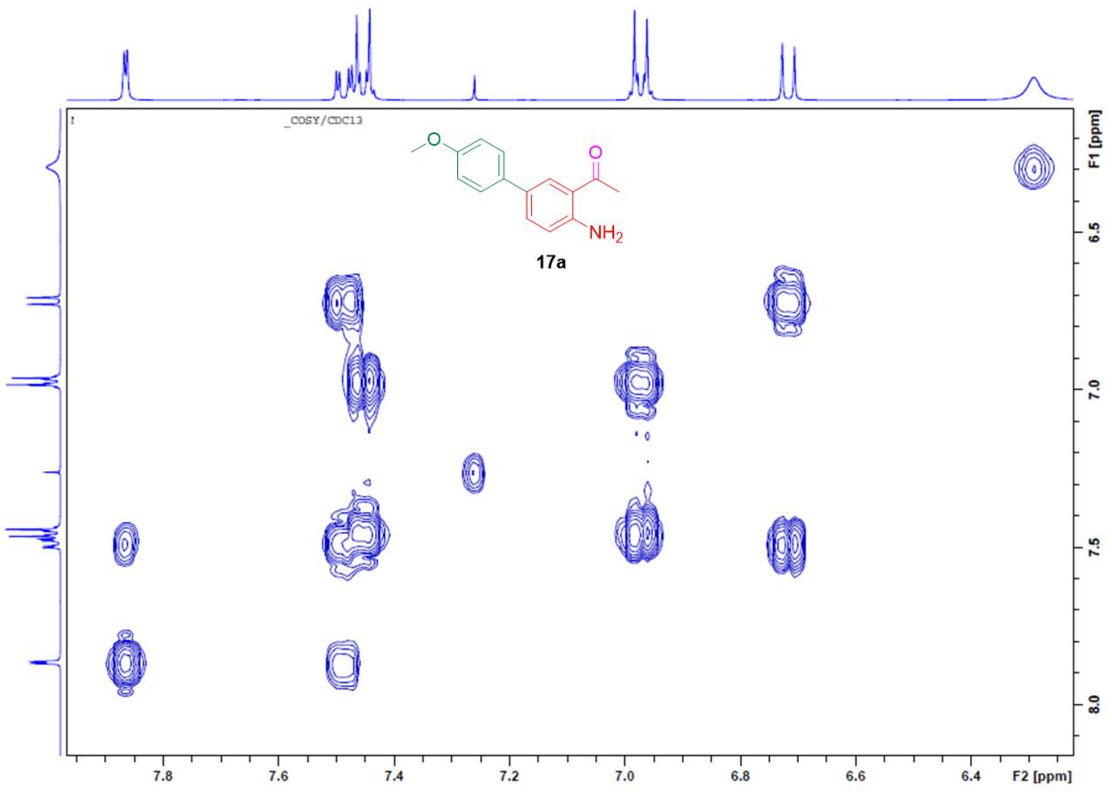
_COSY/CDCl3
O
O
NH2
17a
F1 [ppm]
6.5
7.0
7.5
8.0
7.8
7.6
7.4
7.2
7.0
6.8
6.6
6.4
F2 [ppm]

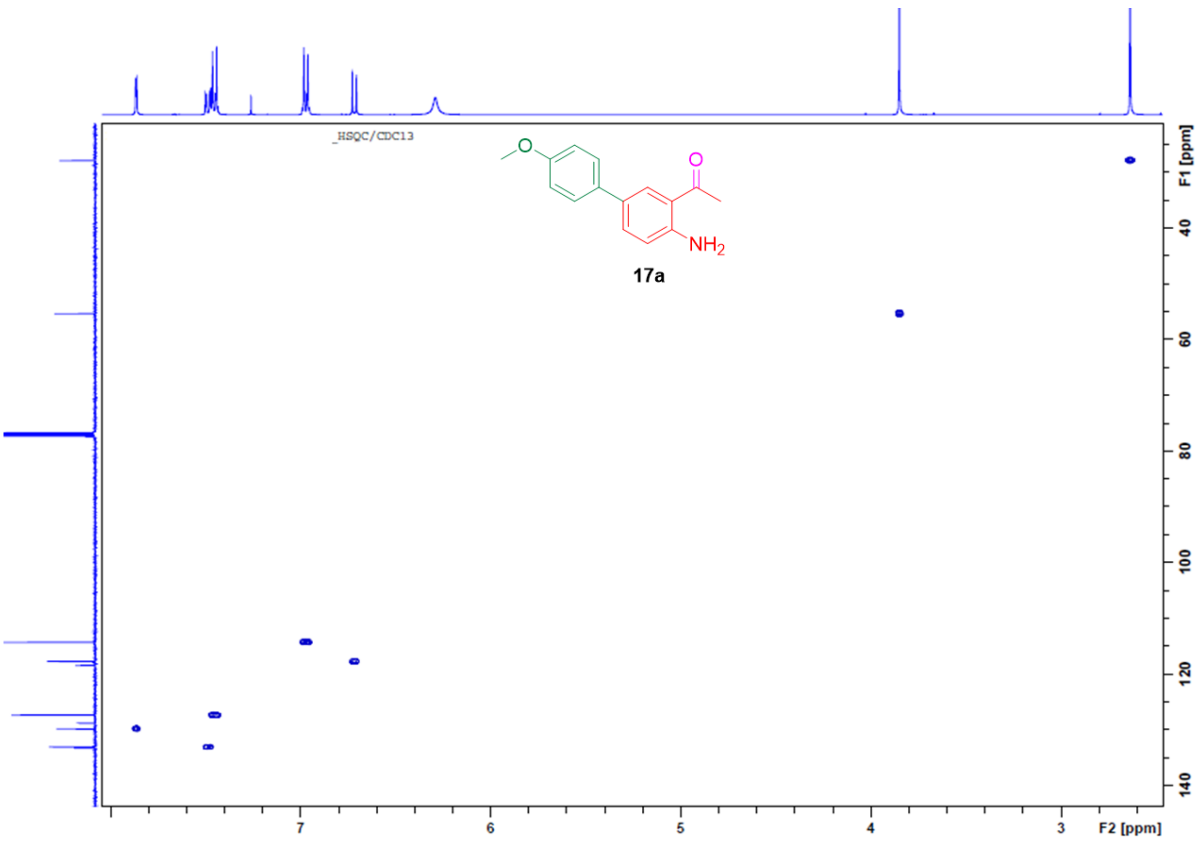

_HSQC/CDCl3
O
NH2
17a
F1 [ppm]
F2 [ppm]

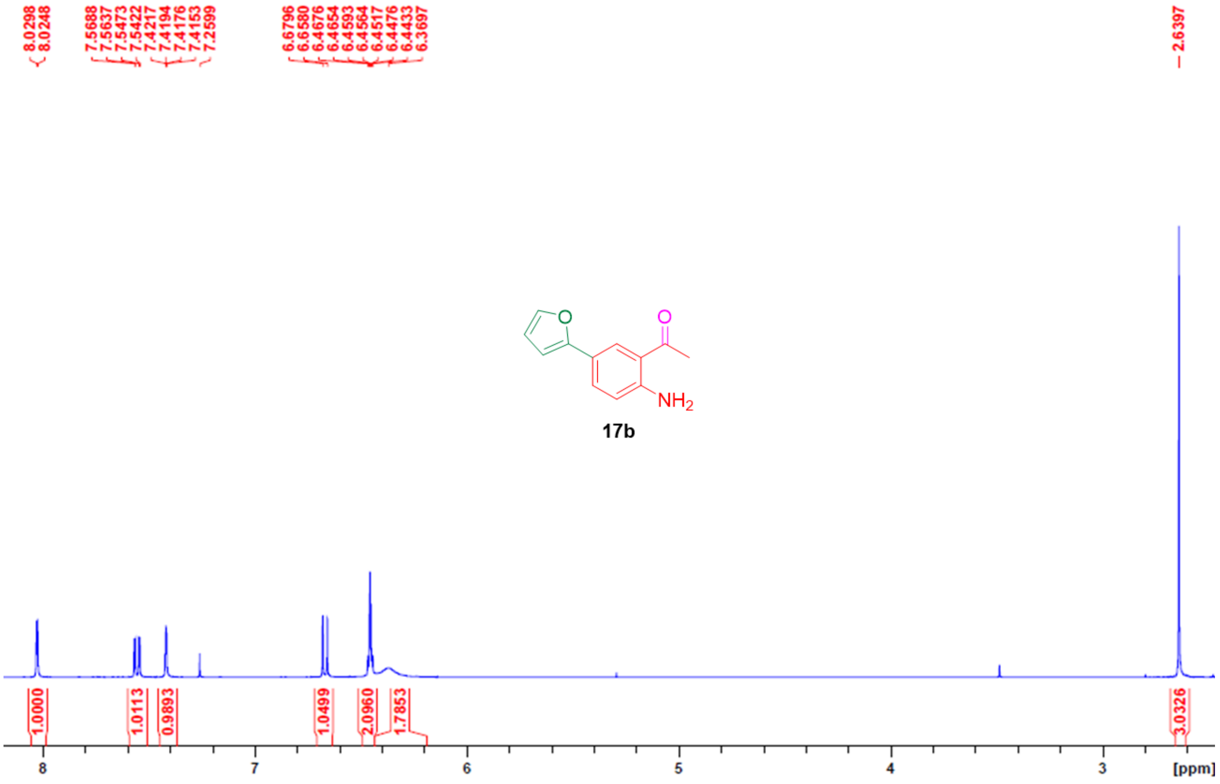

8.0298
8.0248
7.5688
7.5637
7.5473
7.5422
7.4217
7.4194
7.4176
7.4153
7.2599
6.6796
6.6580
6.4676
6.4654
6.4593
6.4564
6.4517
6.4476
6.4433
6.3697
2.6397
O
NH2
17b
1.0000
1.0113
0.9893
1.0499
2.0960
1.7853
3.0326
[ppm]

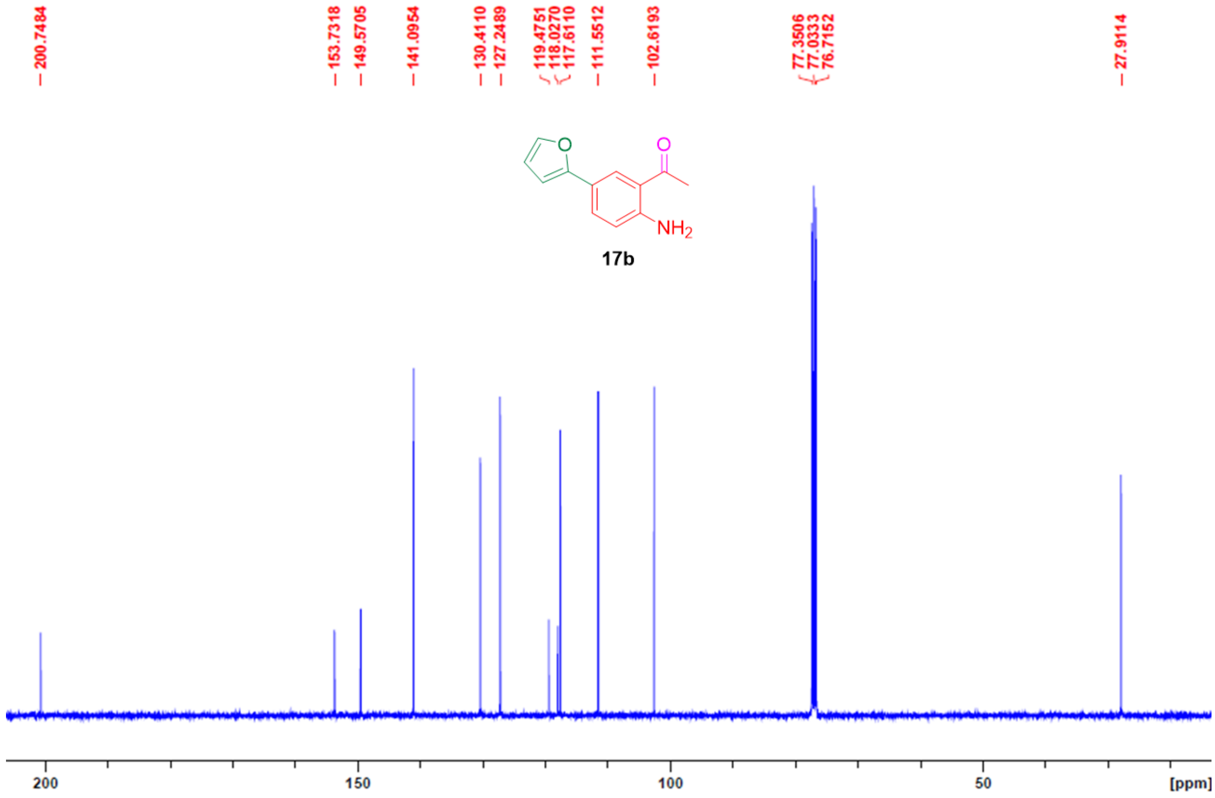
200.7484
153.7318
149.5705
141.0954
130.4110
127.2489
119.4751
118.0270
117.6110
111.5512
102.6193
77.3506
77.0333
76.7152
27.9114
O
NH2
17b
200
150
100
50
[ppm]

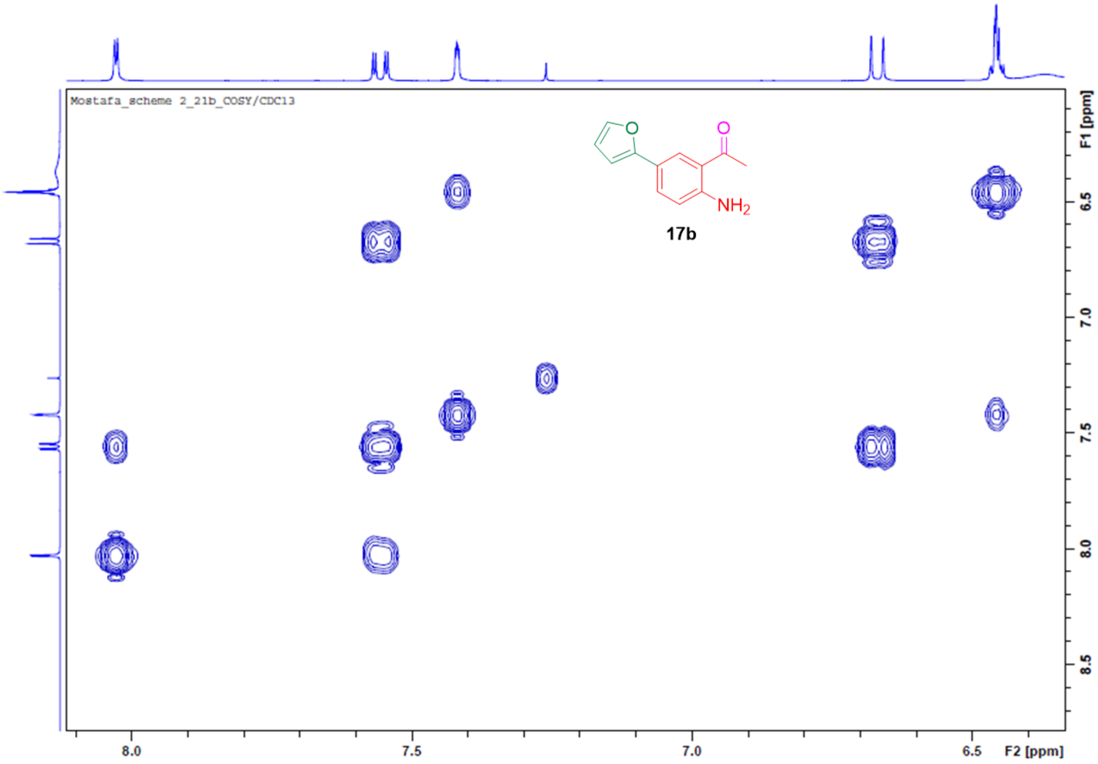
Mostafa_scheme 2_21b_COSY/CDCl3
O
NH2
17b
F1 [ppm]
6.5
7.0
7.5
8.0
8.5
8.0
7.5
7.0
6.5
F2 [ppm]

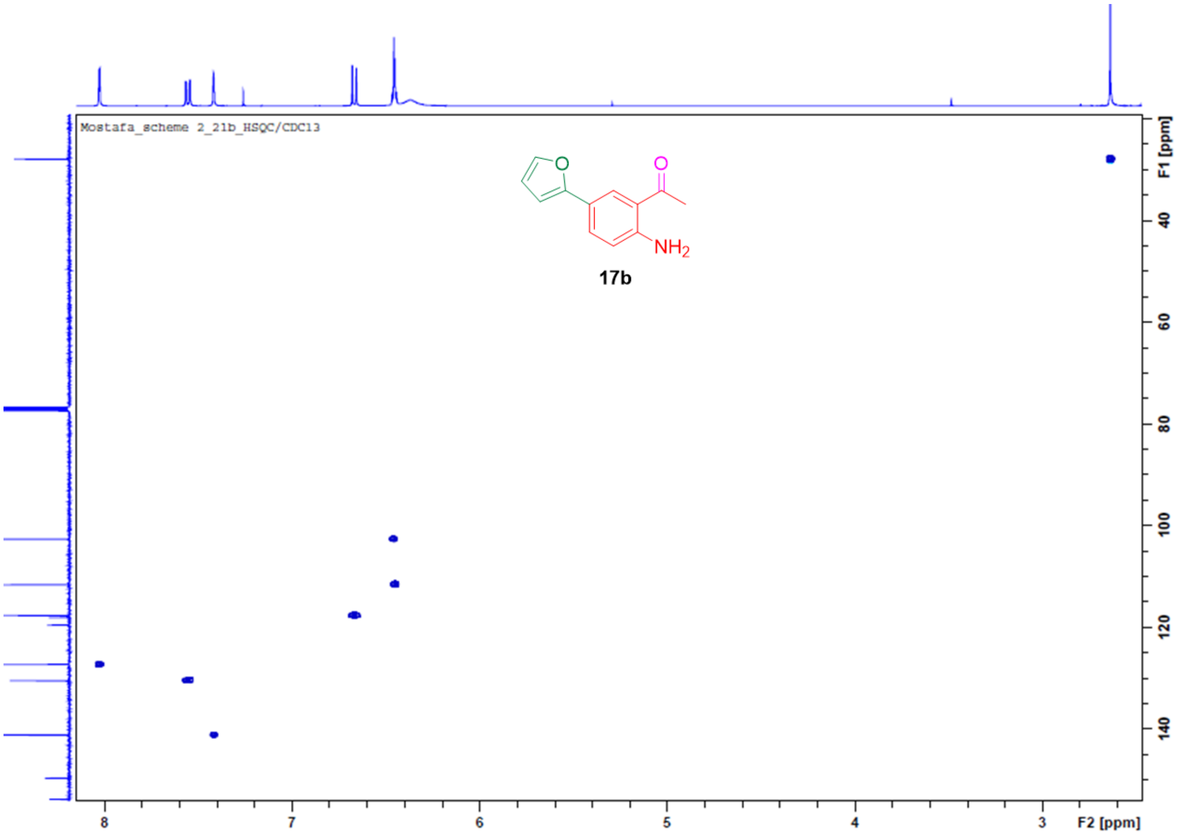
Mostafa_scheme 2_21b_HSQC/CDCl3
O
O
NH2
17b
F1 [ppm]
40
60
80
100
120
140
8
7
6
5
4
3
F2 [ppm]

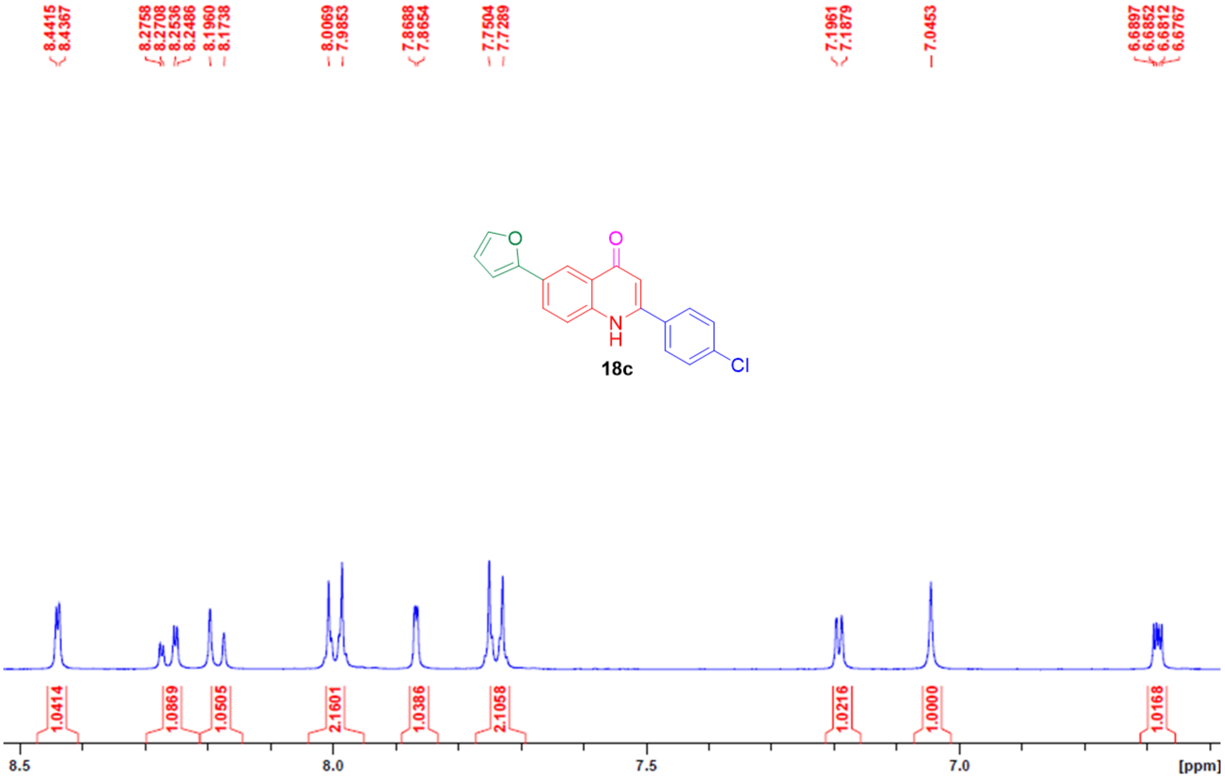
8.4415
8.4367
8.2758
8.2708
8.2536
8.2486
8.1960
8.1738
8.0069
7.9853
7.8688
7.8654
7.7504
7.7289
7.1961
7.1879
7.0453
6.6897
6.6852
6.6812
6.6767
O
O
N
H
Cl
18c
1.0414
1.0869
1.0505
2.1601
1.0386
2.1058
1.0216
1.0000
1.0168
8.5
8.0
7.5
7.0
[ppm]

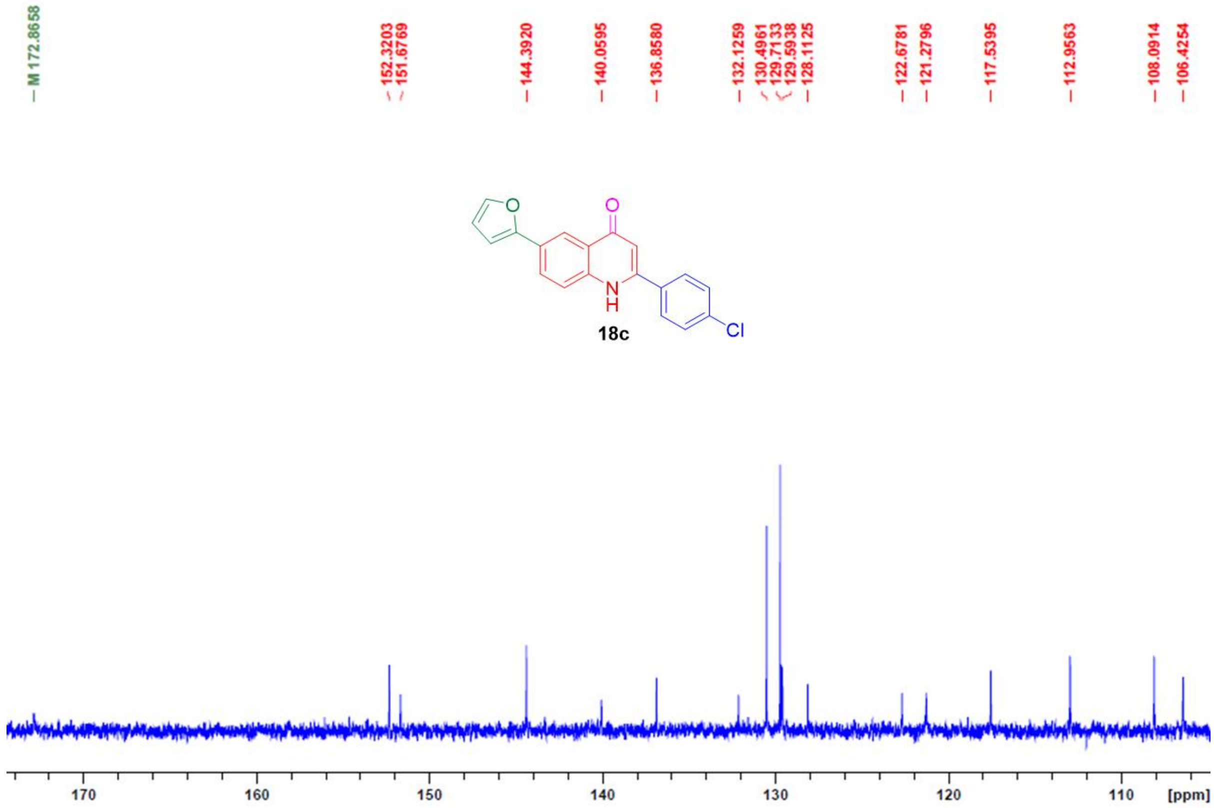
M 172.8658
152.3203
151.6769
144.3920
140.0595
136.8580
132.1259
130.4961
129.7133
129.5938
128.1125
122.6781
121.2796
117.5395
112.9563
108.0914
106.4254
O
N
H
Cl
18c
170
160
150
140
130
120
110
[ppm]

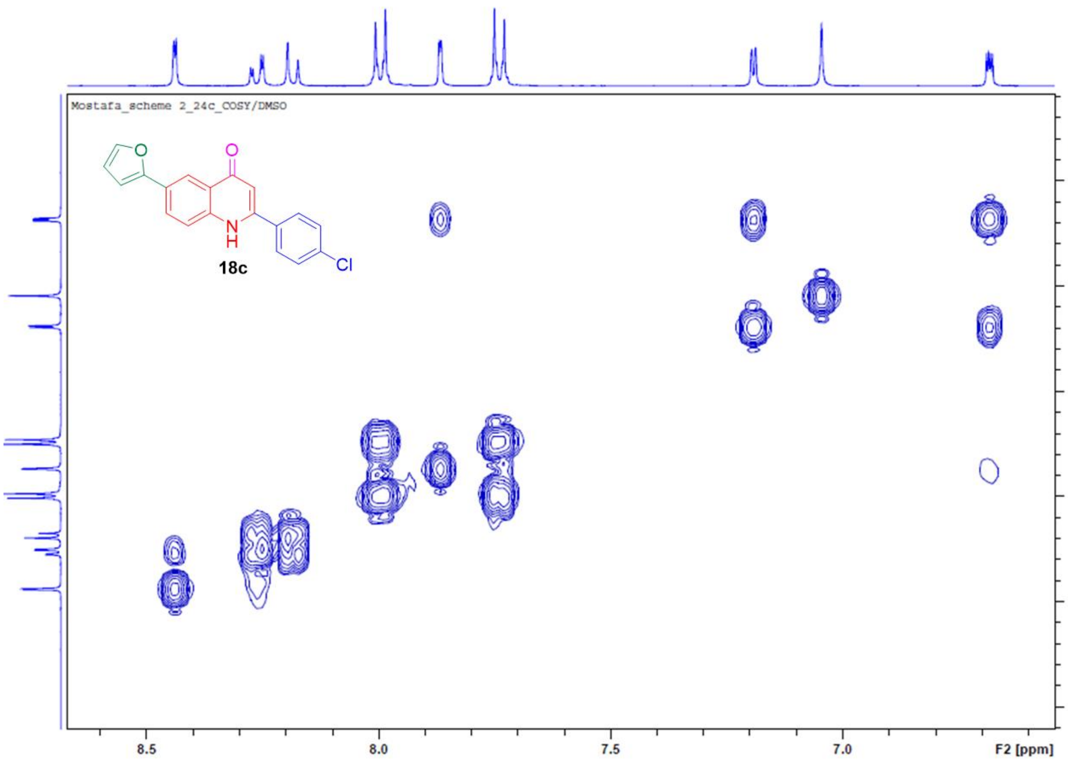
Mostafa_scheme 2_24c_COSY/DMSO
O
N
H
Cl
18c
F1 [ppm]
6.5
7.0
7.5
8.0
8.5
9.0
8.5
8.0
7.5
7.0
F2 [ppm]

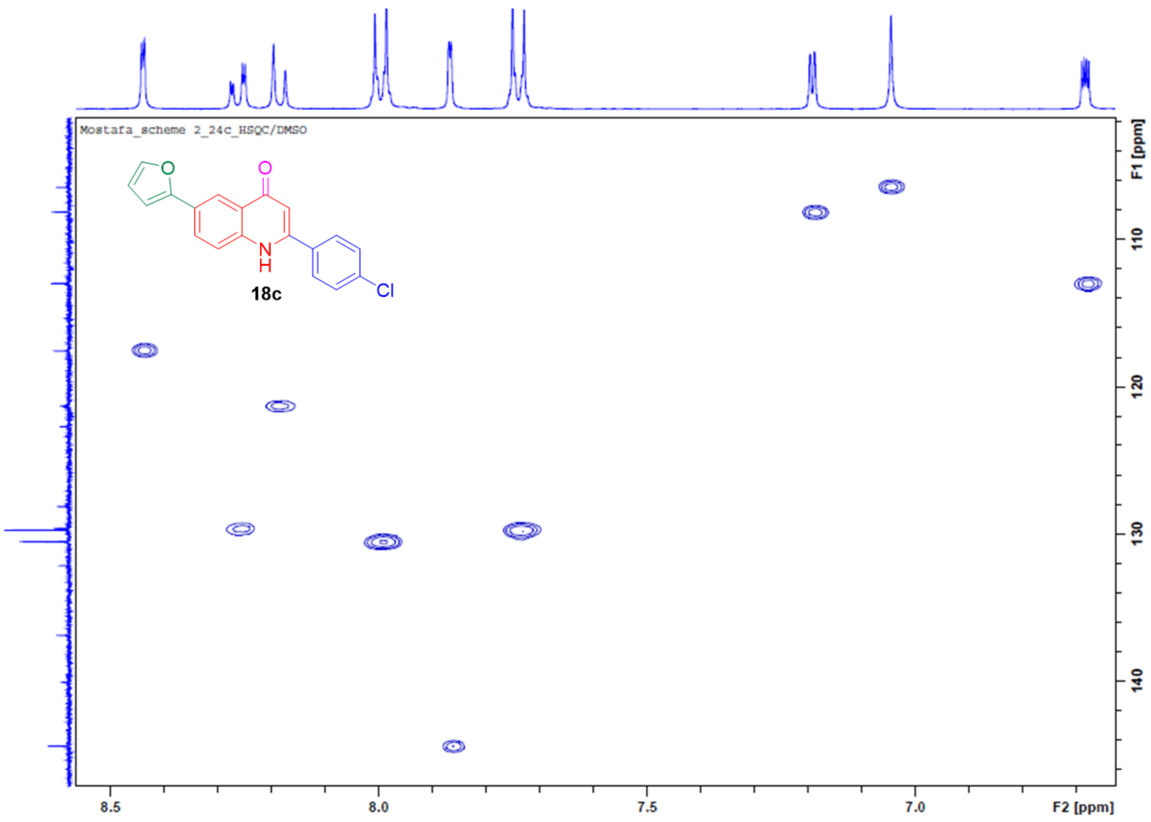
Mostafa_scheme 2_24c_HSQC/DMSO
18c
F1 [ppm]
F2 [ppm]

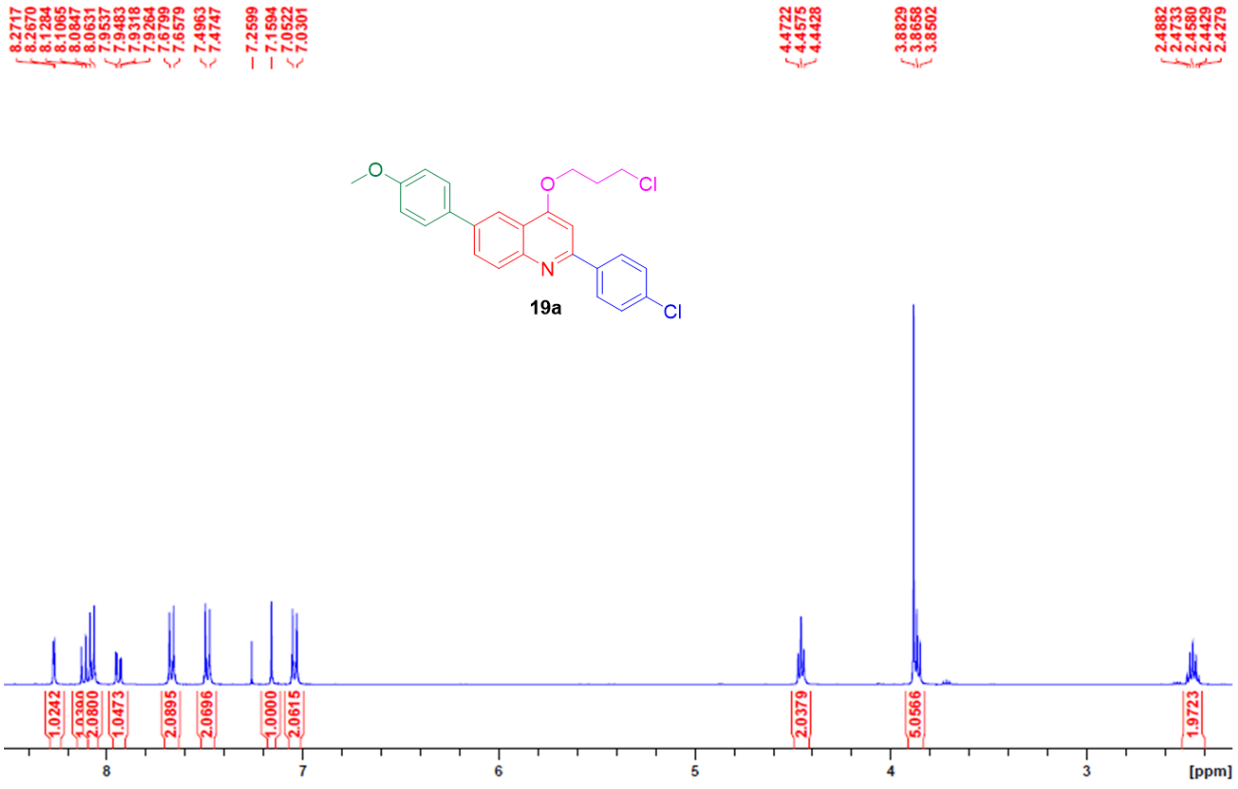
19a
[ppm]

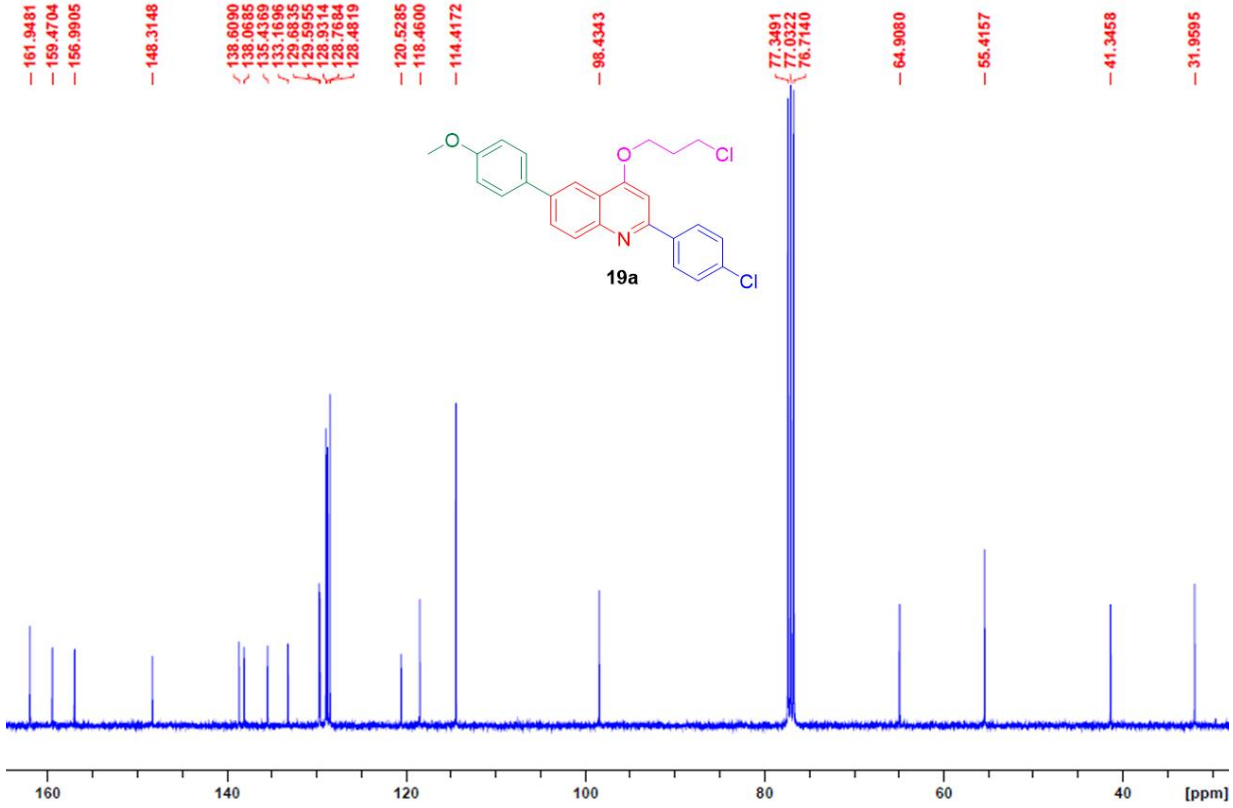
161.9481
159.4704
156.9905
148.3148
138.6090
138.0685
135.4369
133.1696
129.6835
129.5955
128.9314
128.7684
128.4819
120.5285
118.4600
114.4172
98.4343
77.3491
77.0322
76.7140
64.9080
55.4157
41.3458
31.9595
19a
[ppm]

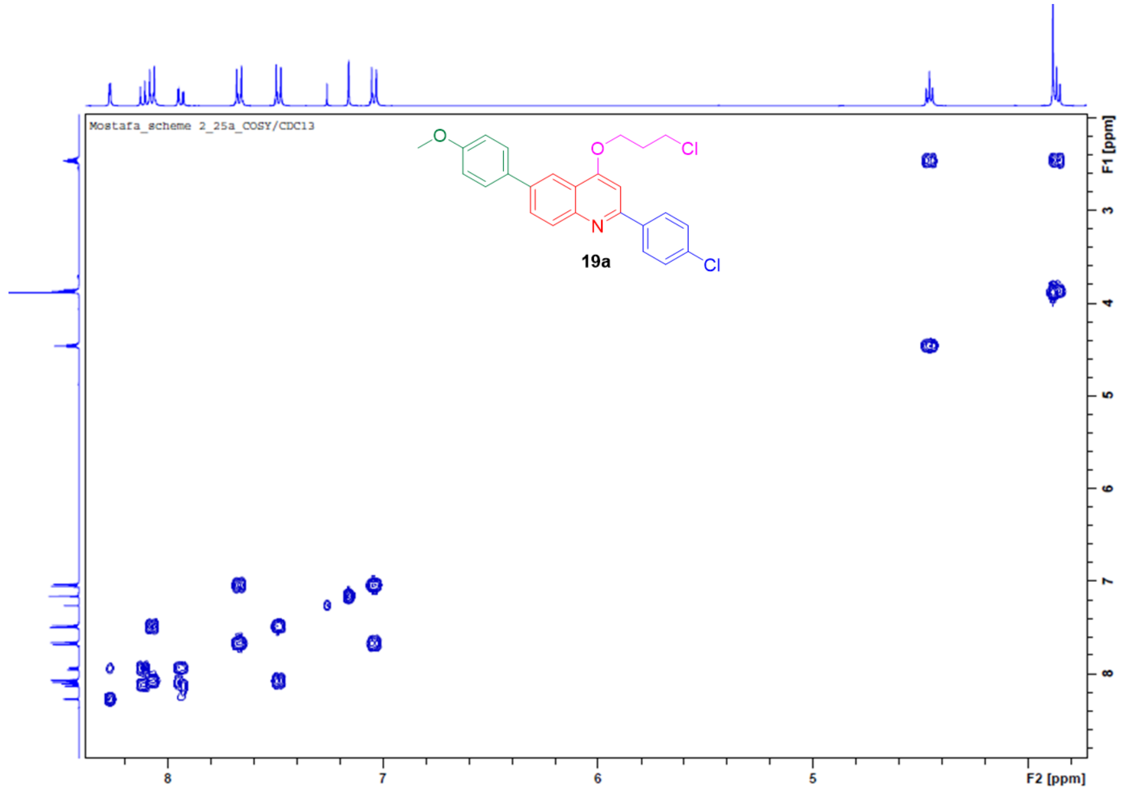
Mostafa_scheme 2_25a_COSY/CDCl3
19a
F1 [ppm]
F2 [ppm]

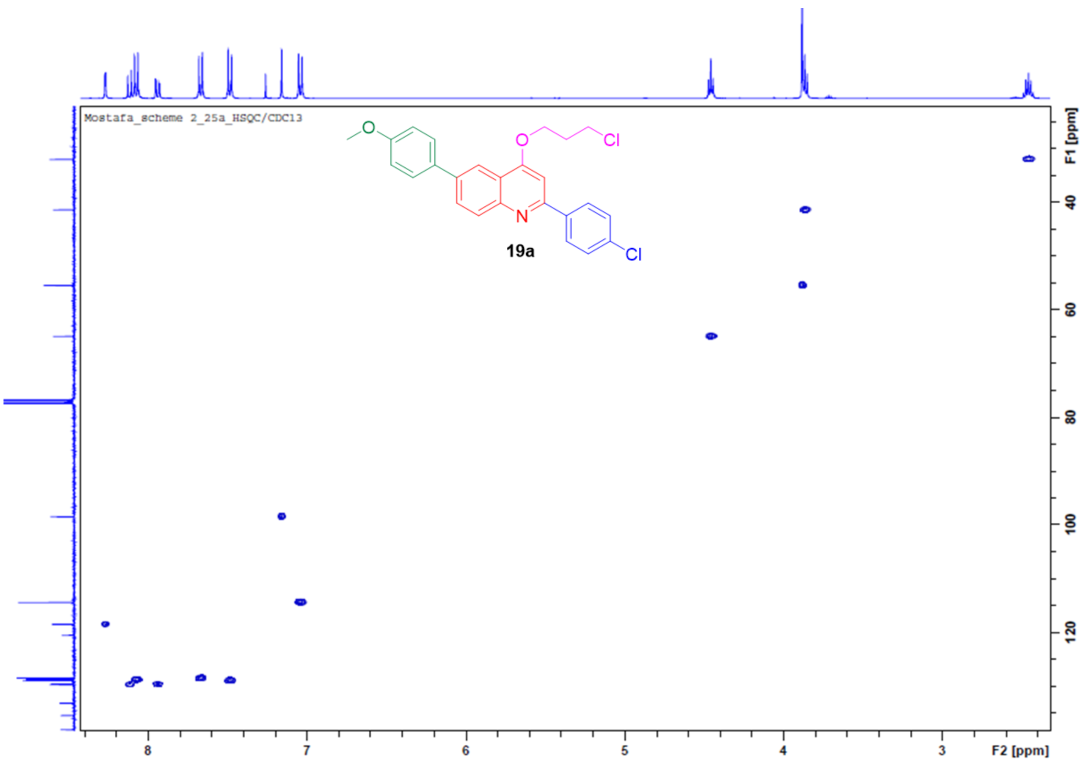
Mostafa_scheme 2_25a_HSQC/CDCl3
O
O
Cl
N
Cl
19a
F1 [ppm]
40
60
80
100
120
8
7
6
5
4
3
F2 [ppm]

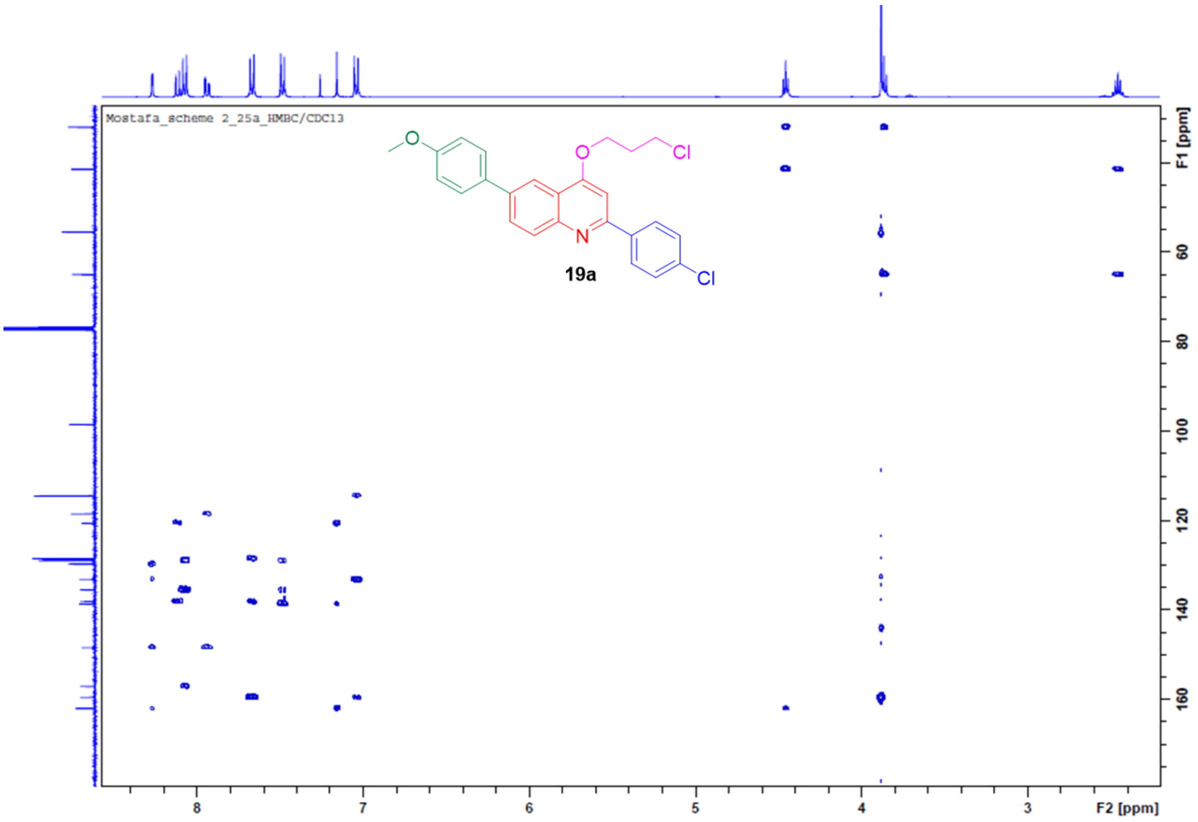
Mostafa_scheme 2_25a_HMBC/CDCl3
O
O
Cl
N
Cl
19a
F1 [ppm]
60
80
100
120
140
160
8
7
6
5
4
3
F2 [ppm]

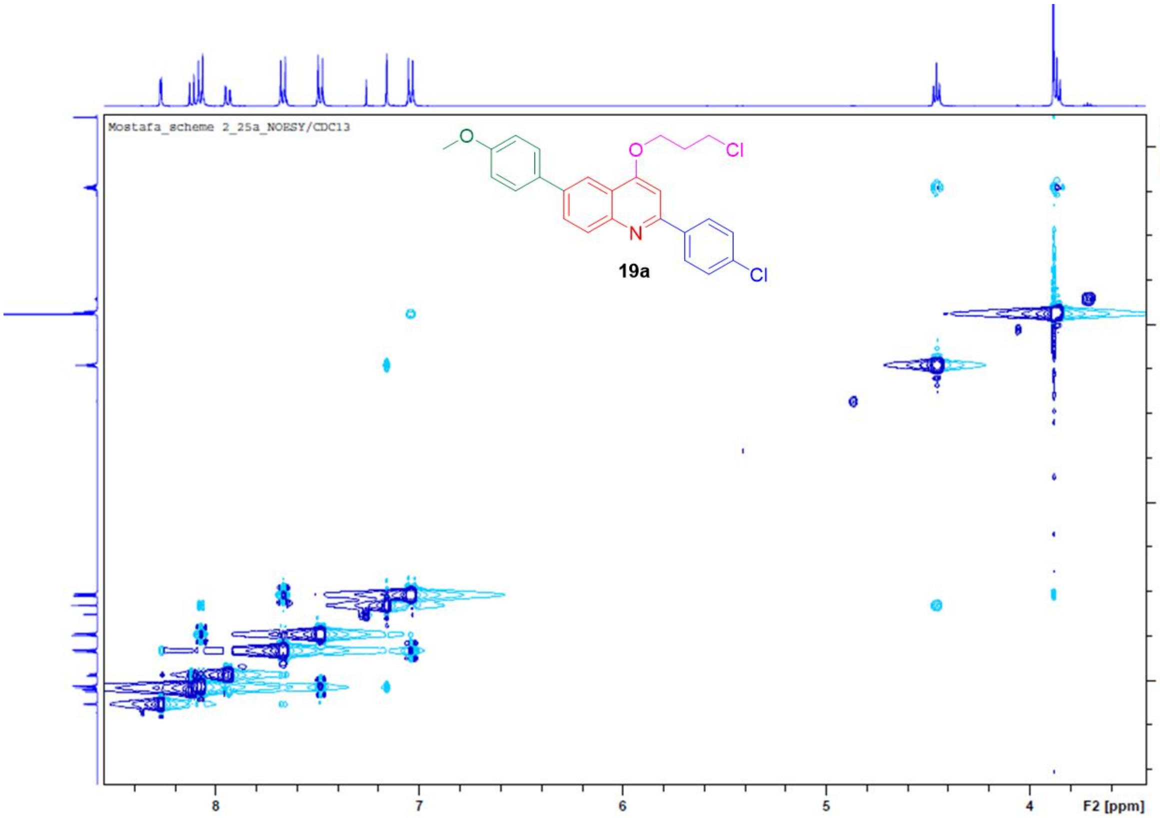
Mostafa_scheme 2_25a_NOESY/CDCl3
19a
F1 [ppm]
F2 [ppm]

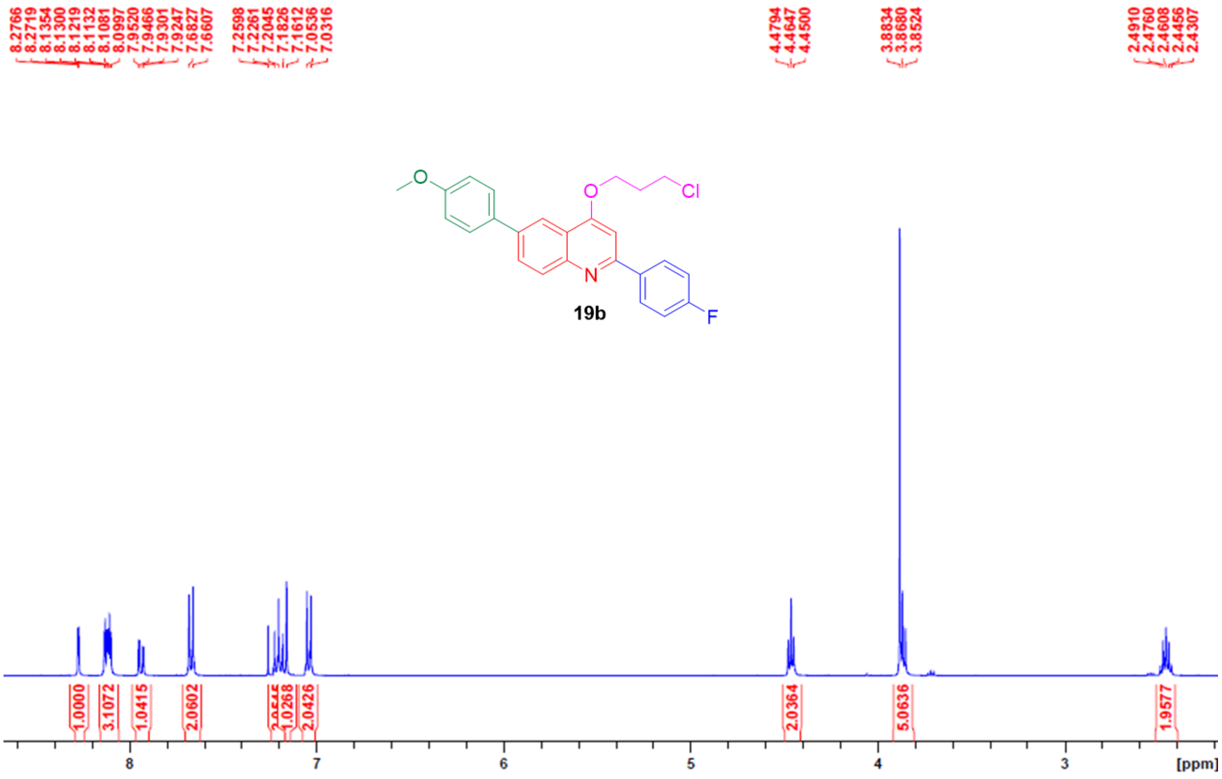
8.2766
8.2719
8.1354
8.1300
8.1219
8.1132
8.1081
8.0997
7.9520
7.9466
7.9301
7.9247
7.6827
7.6607
7.2598
7.2261
7.2045
7.1826
7.1612
7.0536
7.0316
4.4794
4.4647
4.4500
3.8834
3.8680
3.8524
2.4910
2.4760
2.4608
2.4456
2.4307
19b
1.0000
3.1072
1.0415
2.0602
1.0268
2.0426
2.0364
5.0636
1.9577
[ppm]

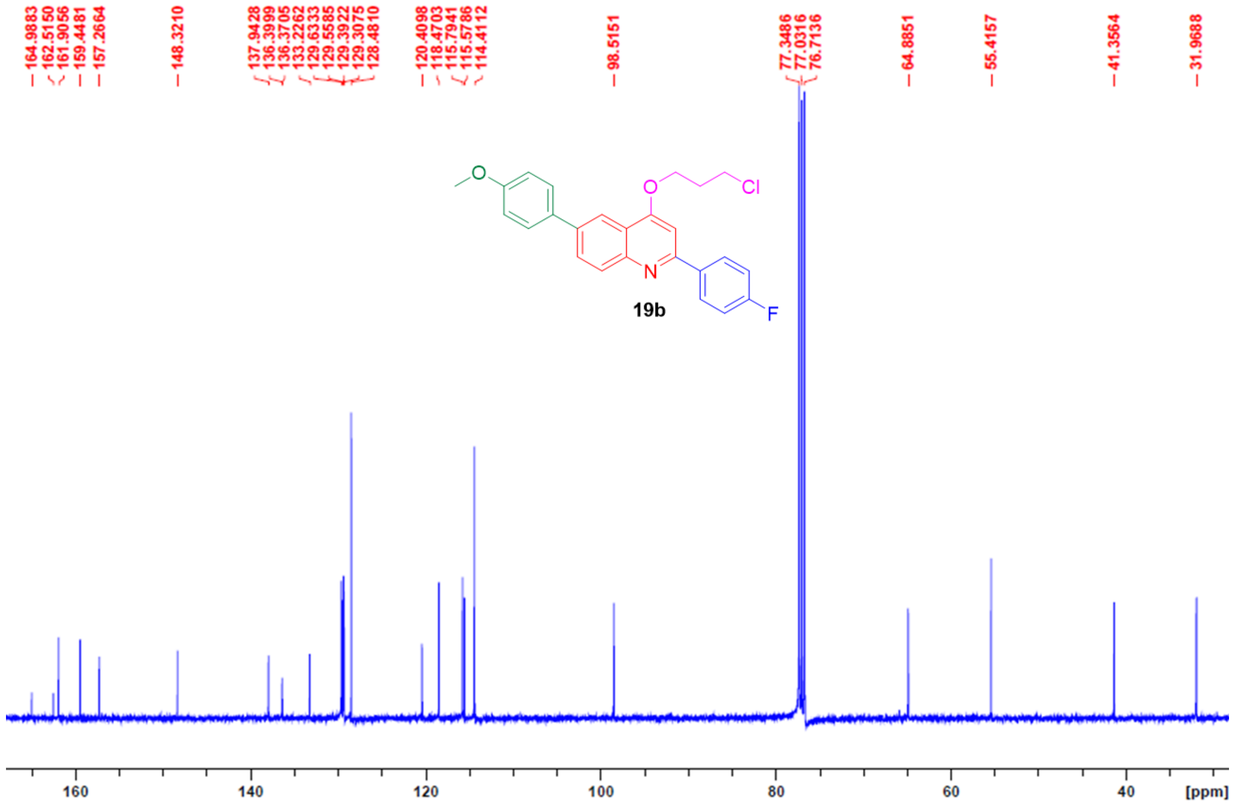
164.9883
162.5150
161.9056
159.4481
157.2664
148.3210
137.9428
136.3999
136.3705
133.2262
129.6333
129.5585
129.3922
129.3075
128.4810
120.4098
118.4703
115.7941
115.5786
114.4112
98.5151
77.3486
77.0316
76.7136
64.8851
55.4157
41.3564
31.9688
O
O
Cl
N
F
19b
160
140
120
100
80
60
40
[ppm]

_19F/CDCl3

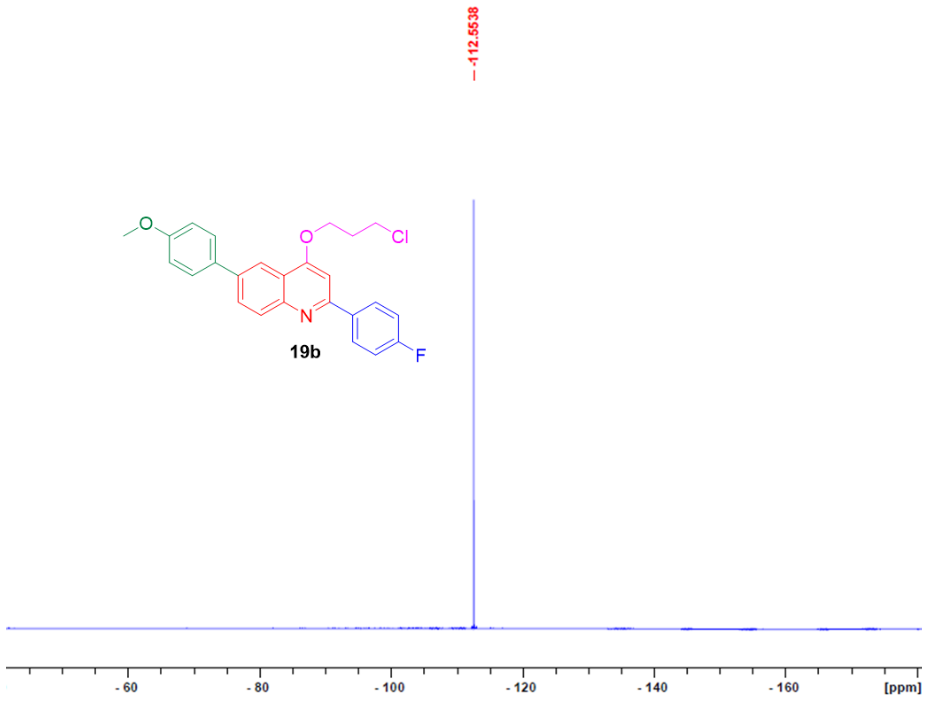
-112.5538
O
O
Cl
N
F
19b
-60
-80
-100
-120
-140
-160
[ppm]

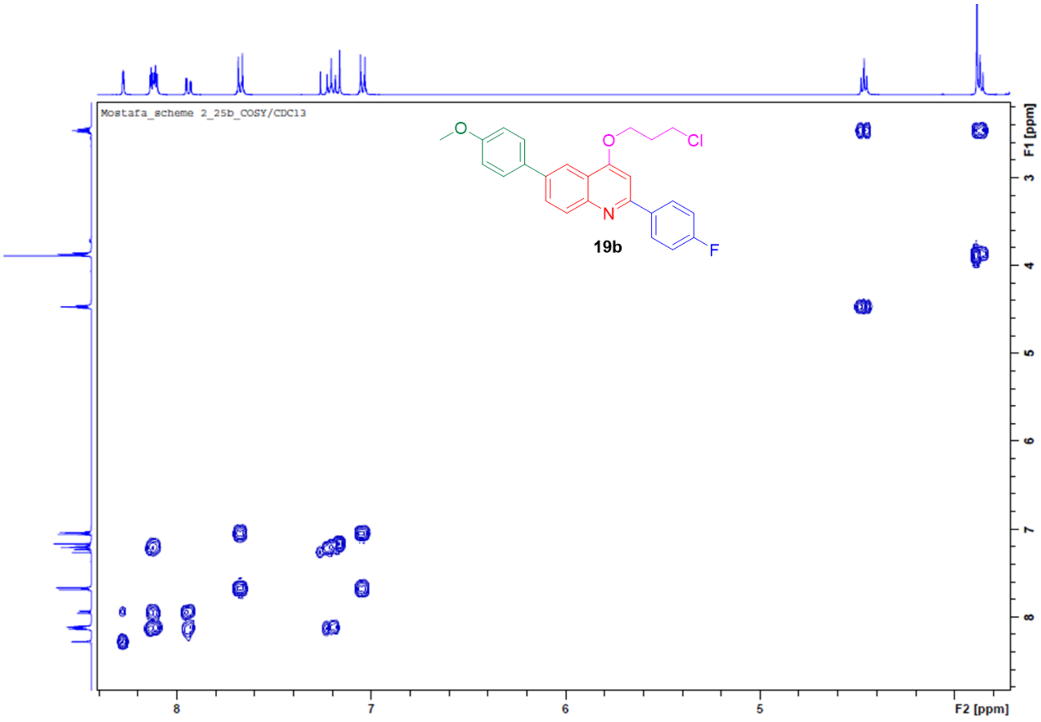
Mostafa_scheme 2_25b_COSY/CDCl3
O
O
Cl
N
F
19b
F1 [ppm]
F2 [ppm]
3
4
5
6
7
8
8
7
6
5

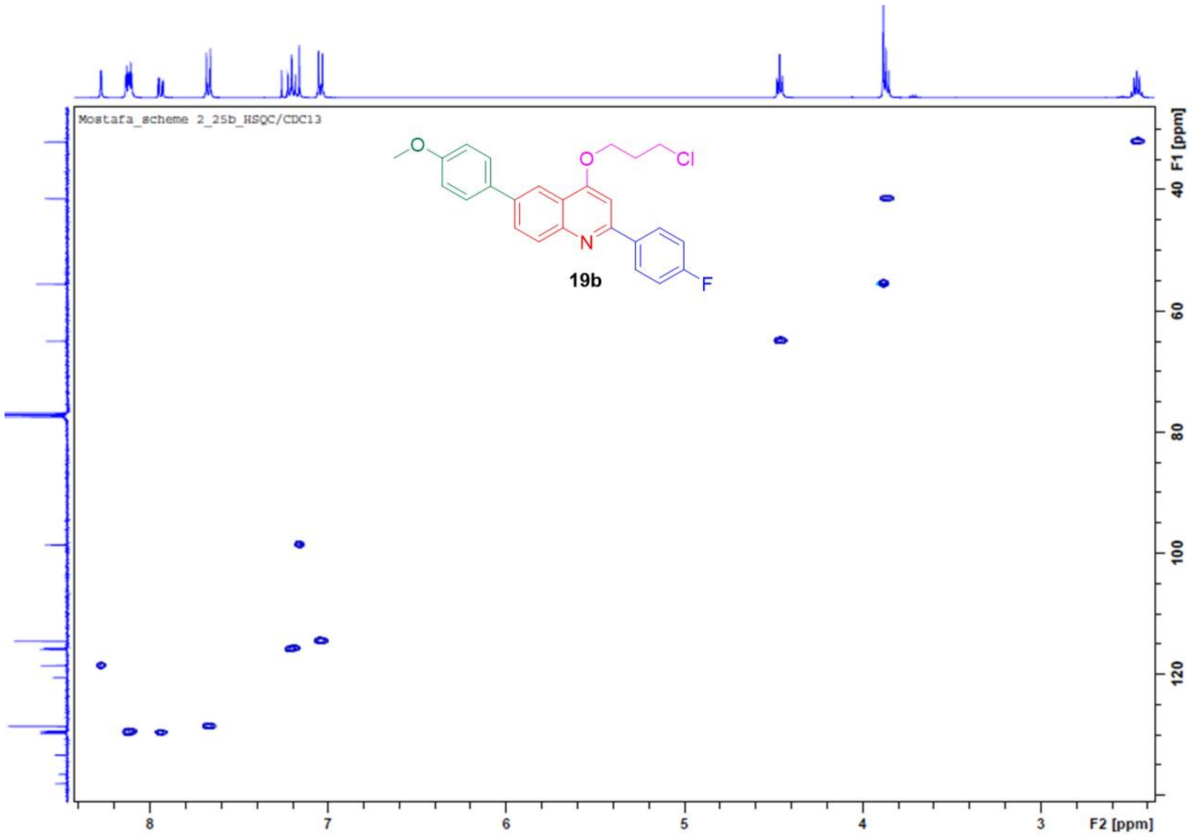
Mostafa_scheme 2_25b_HSQC/CDCl3
O
O
Cl
N
F
19b
F1 [ppm]
F2 [ppm]
40
60
80
100
120
8
7
6
5
4
3

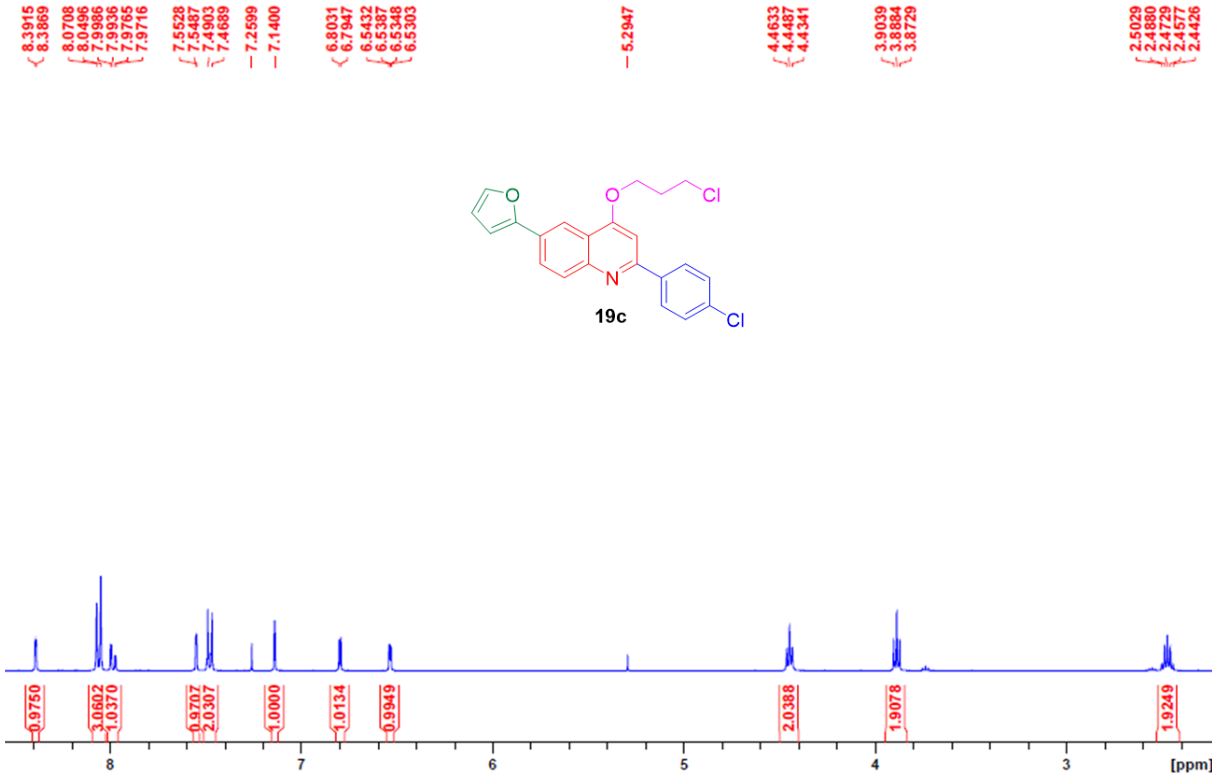
8.3915
8.3869
8.0708
8.0496
7.9986
7.9936
7.9765
7.9716
7.5528
7.5487
7.4903
7.4689
7.2599
7.1400
6.8031
6.7947
6.5432
6.5387
6.5348
6.5303
5.2947
4.4633
4.4487
4.4341
3.9039
3.8884
3.8729
2.5029
2.4880
2.4729
2.4577
2.4426
O
Cl
N
Cl
19c
0.9750
3.0602
1.0370
0.9707
2.0307
1.0000
1.0134
0.9949
2.0388
1.9078
1.9249
8
7
6
5
4
3
[ppm]

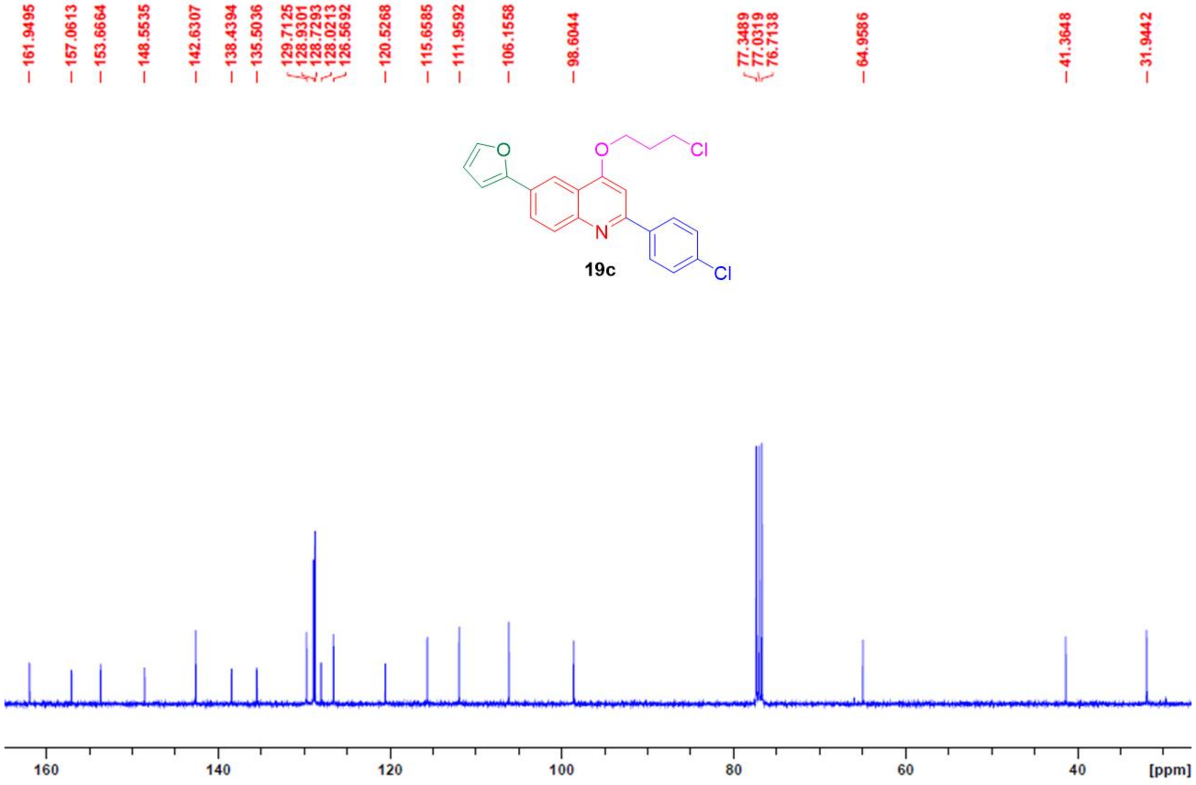
161.9495
157.0613
153.6664
148.5535
142.6307
138.4394
135.5036
129.7125
128.9301
128.7293
128.0213
126.5692
120.5268
115.6585
111.9592
106.1558
98.6044
77.3489
77.0319
76.7138
64.9586
41.3648
31.9442
O
Cl
N
Cl
19c
160
140
120
100
80
60
40
[ppm]

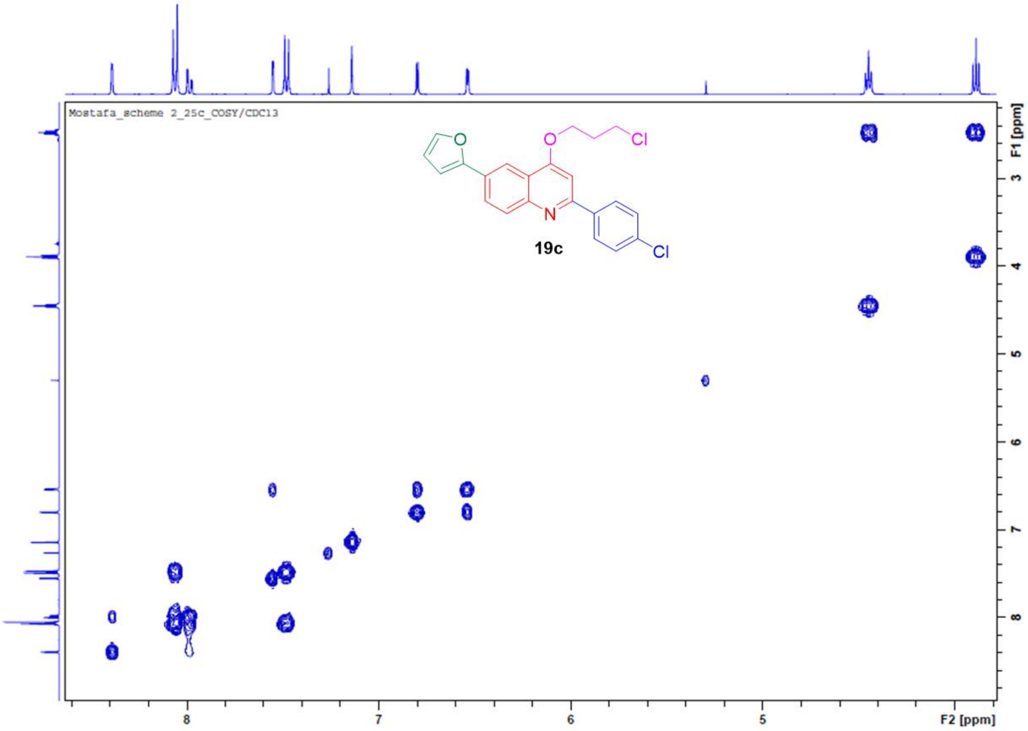
Mostafa_scheme 2_25c_COSY/CDCl3
19c
F1 [ppm]
F2 [ppm]

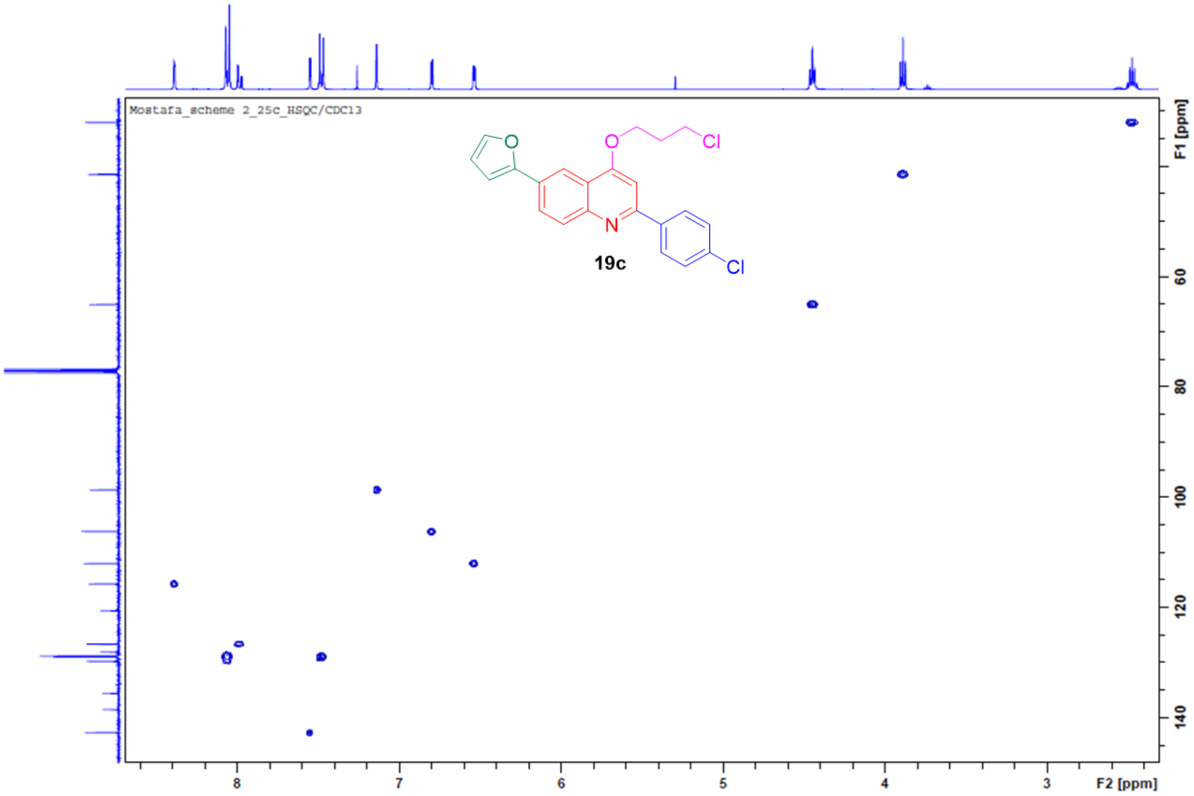
Mostafa_scheme 2_25c_HSQC/CDCl3
19c
F1 [ppm]
F2 [ppm]

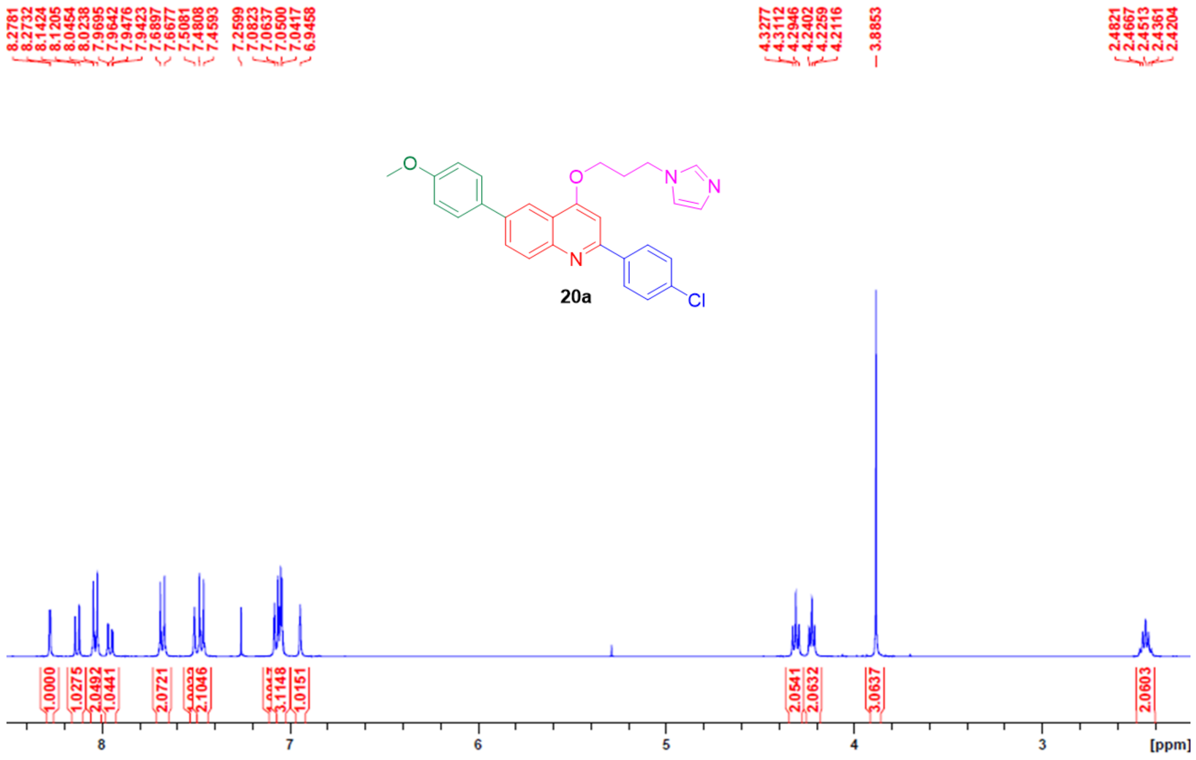

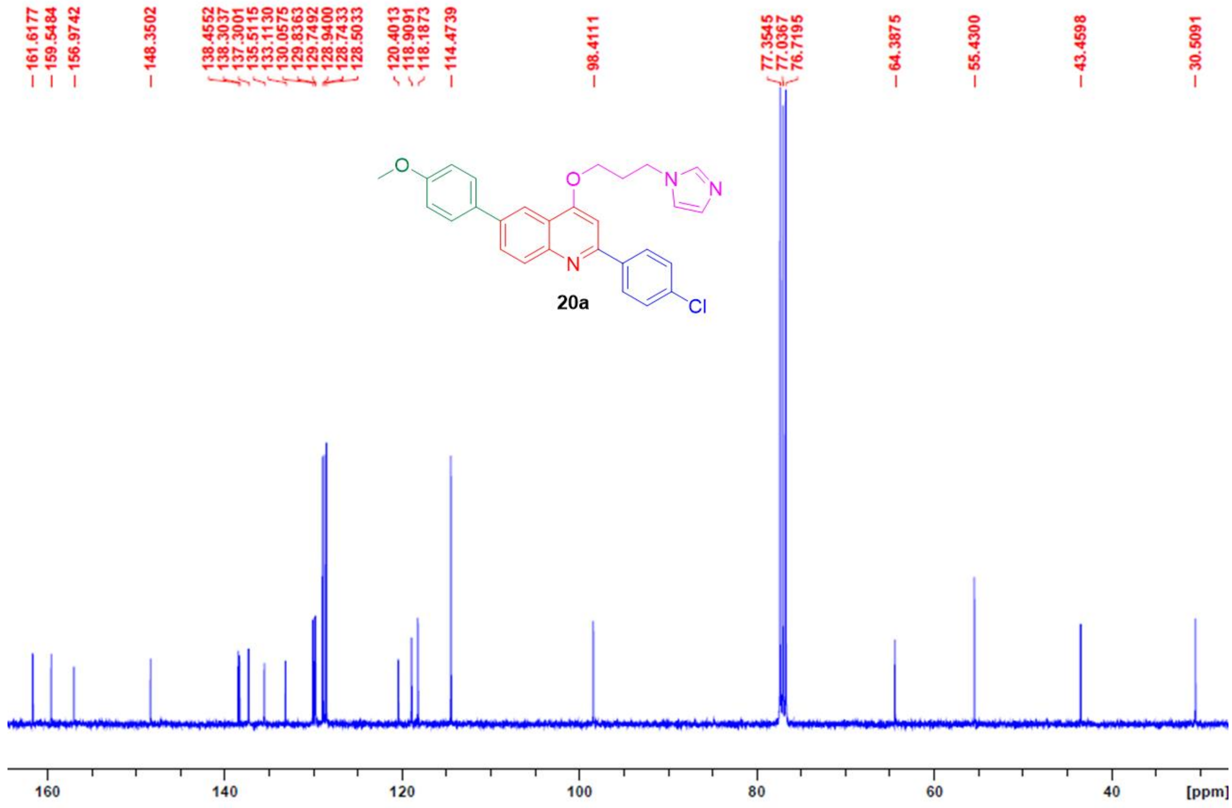

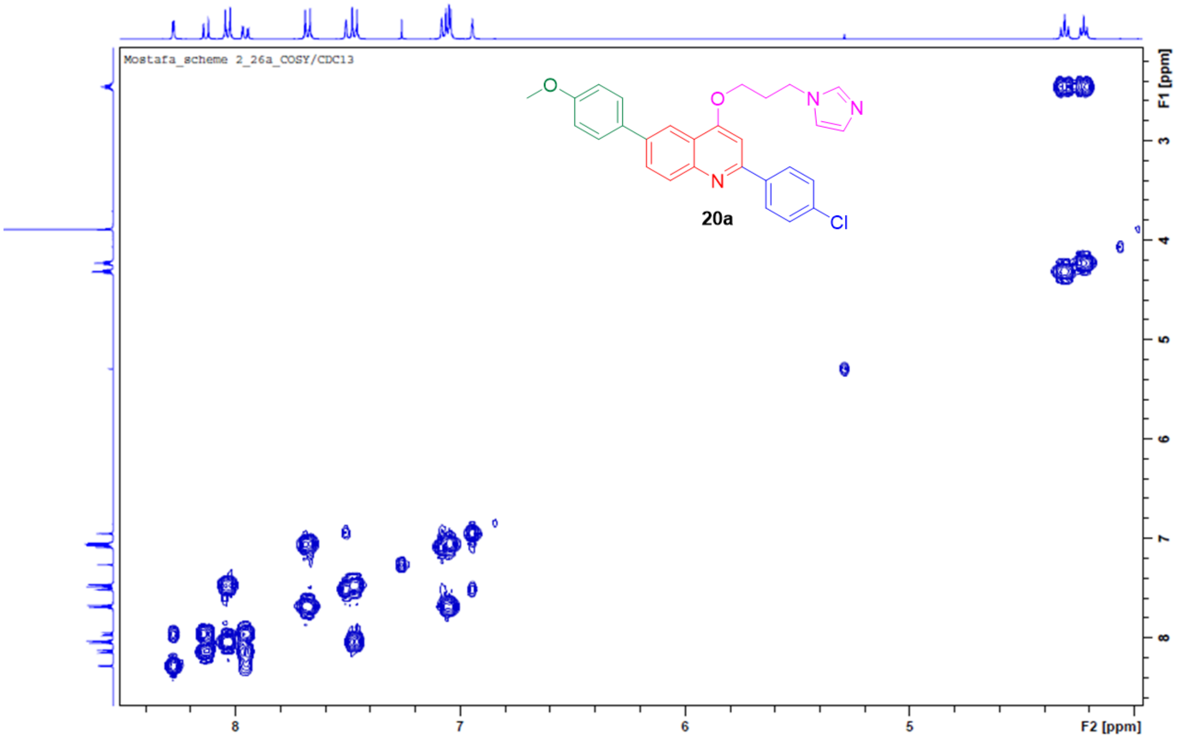

Mostafa_scheme 2_26a_COSY/CDCl3
20a
F1 [ppm]
F2 [ppm]

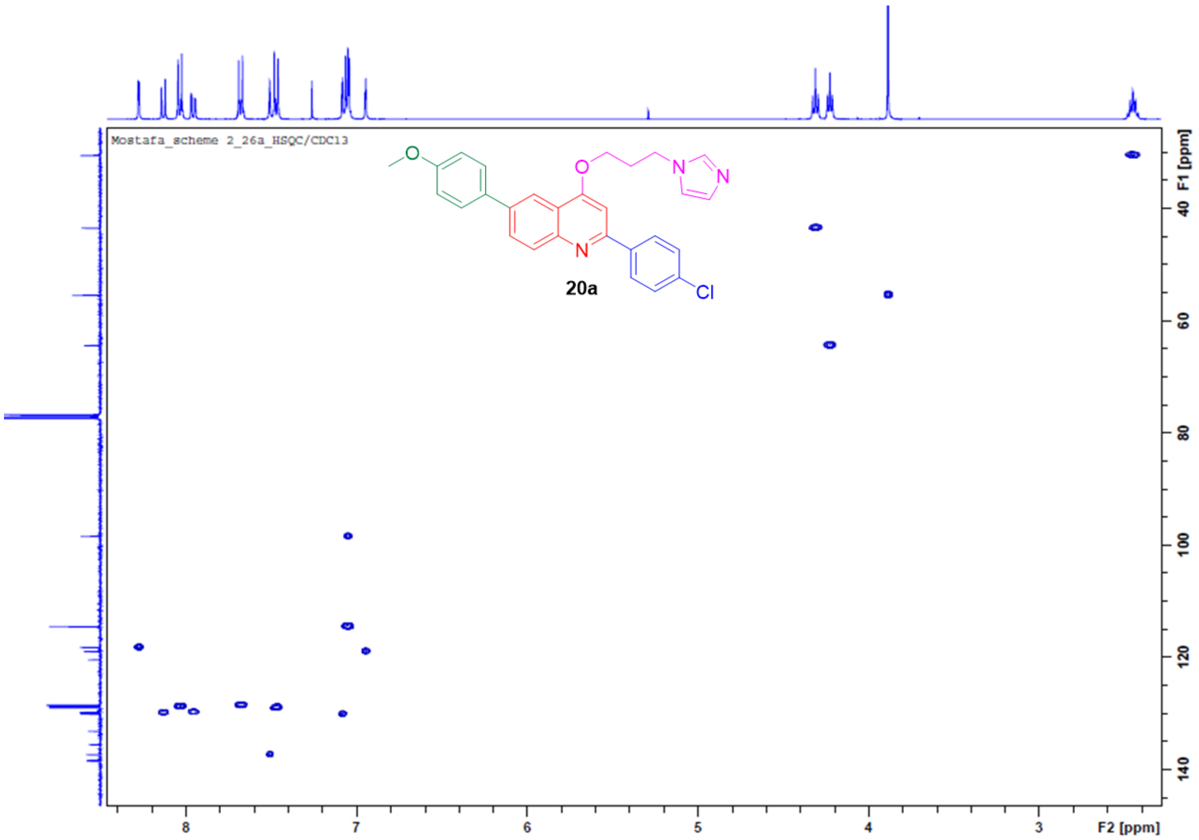

Mostafa_scheme 2_26a_HSQC/CDCl3
20a
F1 [ppm]
F2 [ppm]

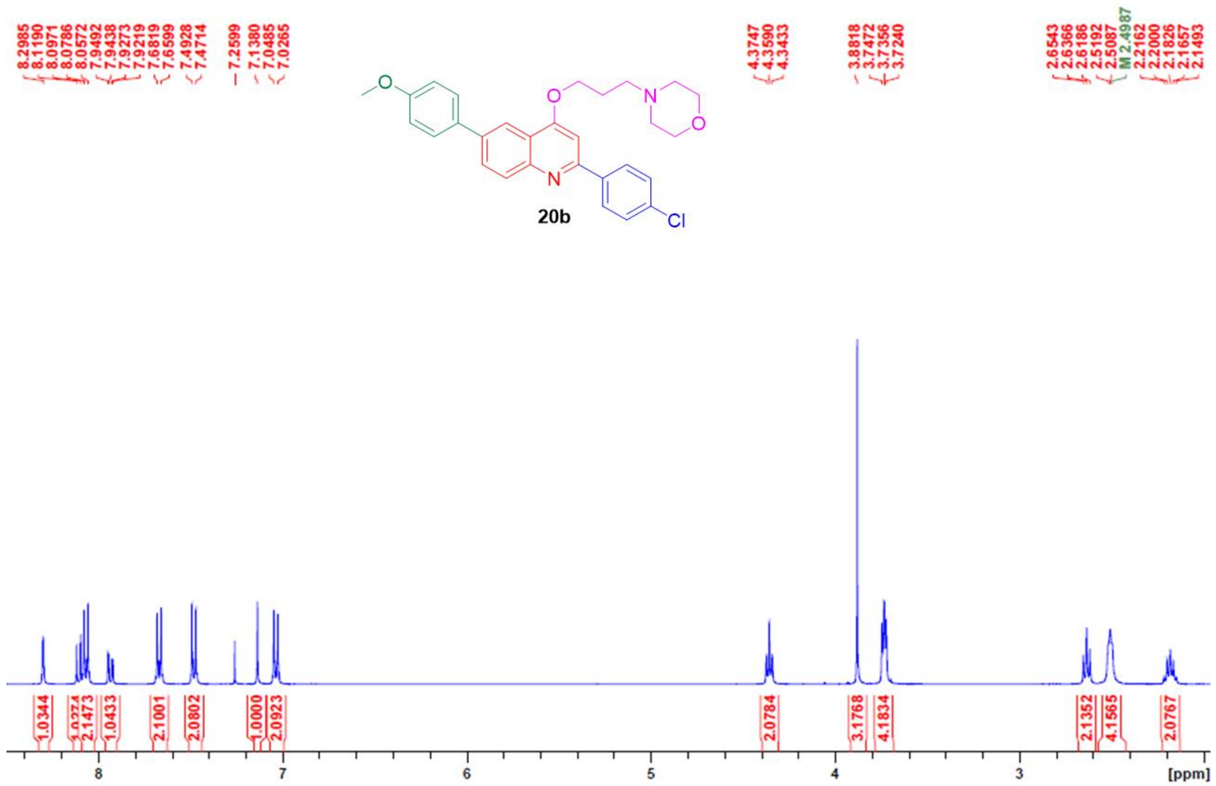
8.2985
8.1190
8.0971
8.0786
8.0572
7.9492
7.9438
7.9273
7.9219
7.6819
7.6599
7.4928
7.4714
7.2599
7.1380
7.0485
7.0265
4.3747
4.3590
4.3433
3.8818
3.7472
3.7356
3.7240
2.6543
2.6366
2.6186
2.5192
2.5087
M 2.4987
2.2162
2.2000
2.1826
2.1657
2.1493
20b
1.0344
1.0274
2.1473
1.0433
2.1001
2.0802
1.0000
2.0923
2.0784
3.1768
4.1834
2.1352
4.1565
2.0767
[ppm]

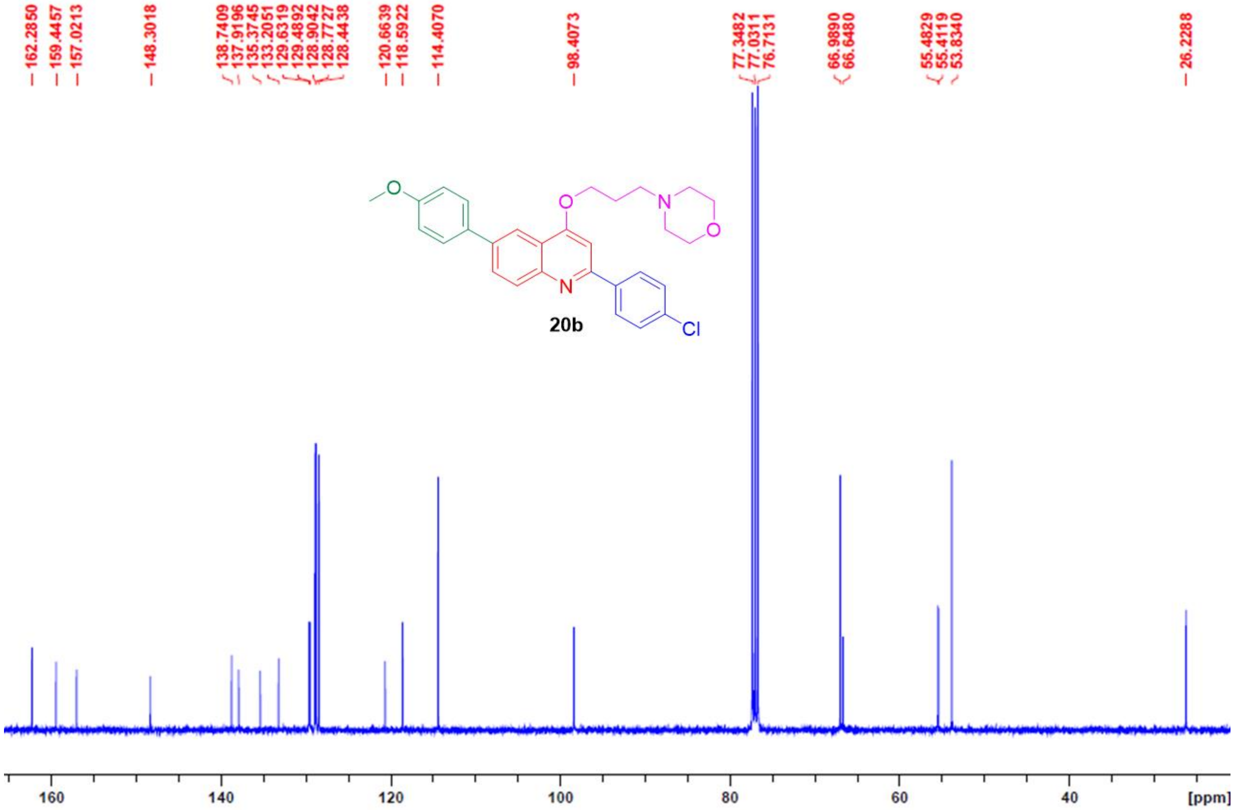
162.2850
159.4457
157.0213
148.3018
138.7409
137.9196
135.3745
133.2051
129.6319
129.4892
128.9042
128.7727
128.4438
120.6639
118.5922
114.4070
98.4073
77.3482
77.0311
76.7131
66.9890
66.6480
55.4829
55.4119
53.8340
26.2288
20b
[ppm]

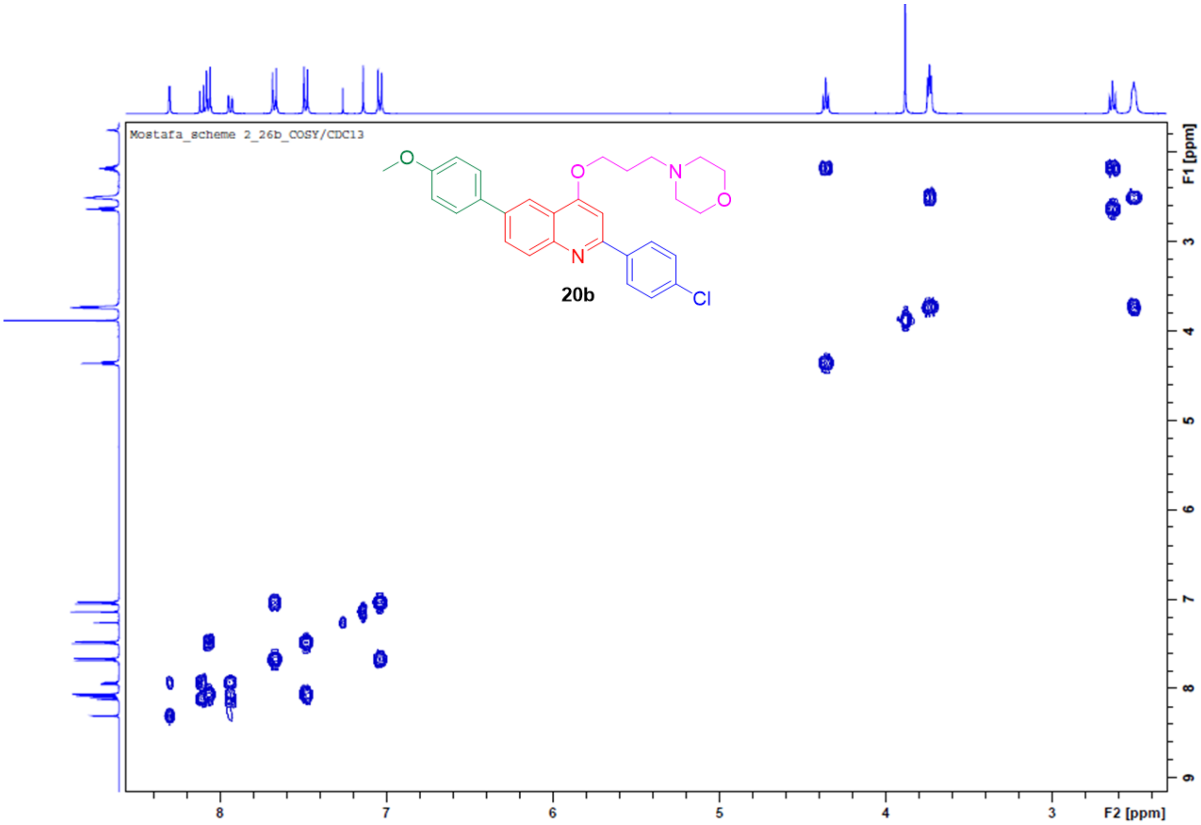
Mostafa_scheme 2_26b_COSY/CDCl3
20b
N
O
N
O
O
Cl
F1 [ppm]
F2 [ppm]

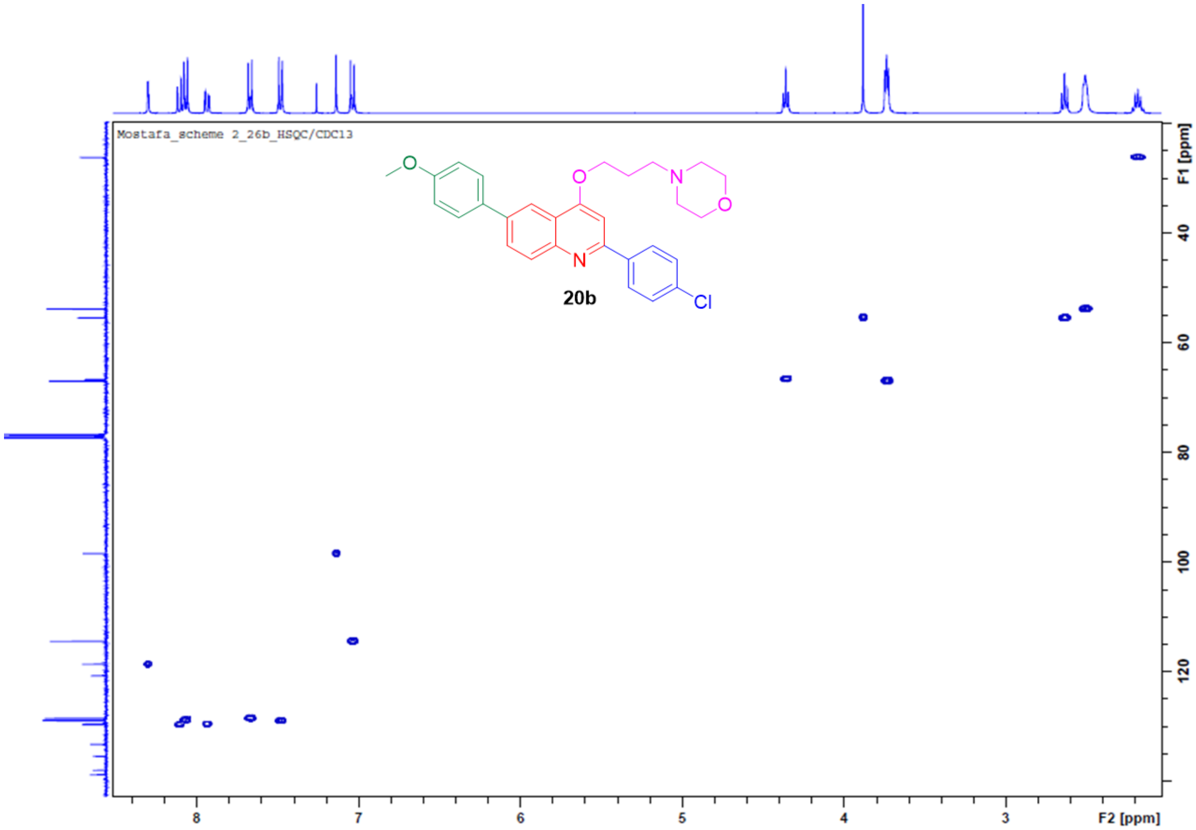
Mostafa_scheme 2_26b_HSQC/CDCl3
20b
N
O
N
O
O
Cl
F1 [ppm]
F2 [ppm]

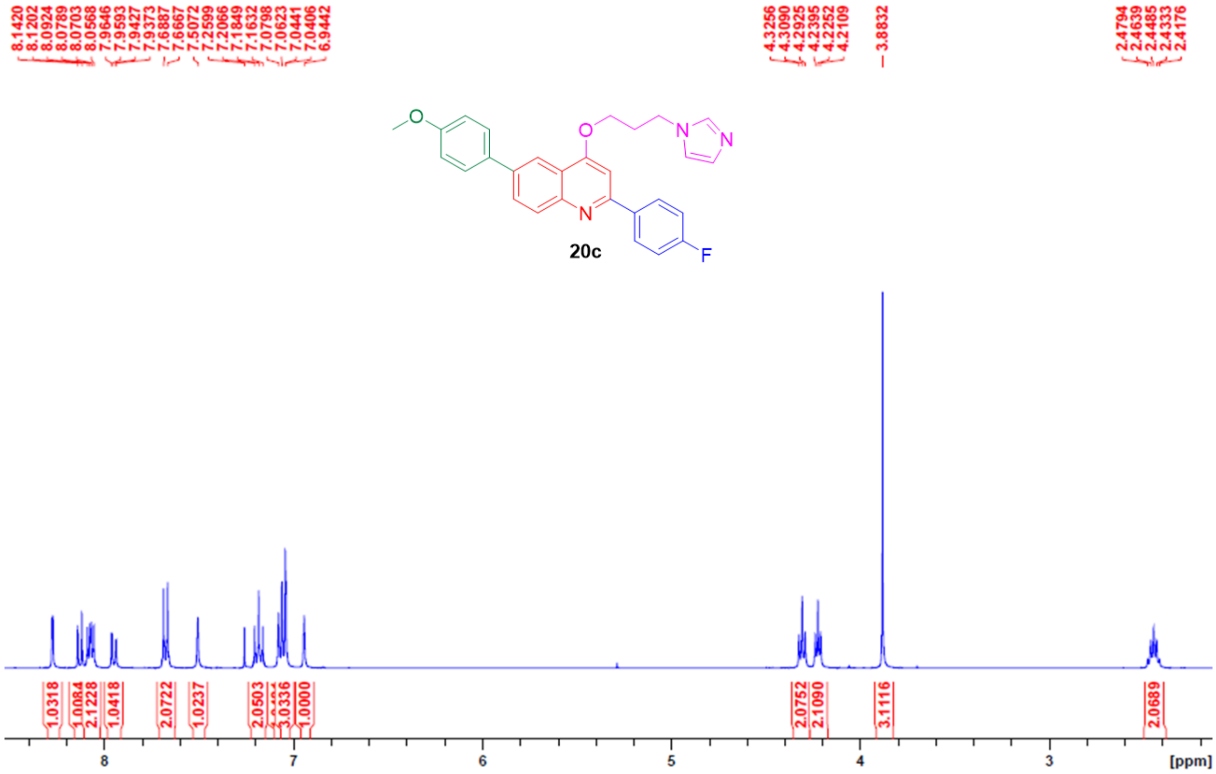

8.1420
8.1202
8.0924
8.0789
8.0703
8.0568
7.9646
7.9593
7.9427
7.9373
7.6887
7.6667
7.5072
7.2599
7.2066
7.1849
7.1632
7.0798
7.0623
7.0441
7.0406
6.9442
4.3256
4.3090
4.2925
4.2395
4.2252
4.2109
3.8832
2.4794
2.4639
2.4485
2.4333
2.4176
O
N
N
N
F
20c
1.0318
1.0084
2.1228
1.0418
2.0722
1.0237
2.0503
3.0336
1.0000
2.0752
2.1090
3.1116
2.0689
8
7
6
5
4
3
[ppm]

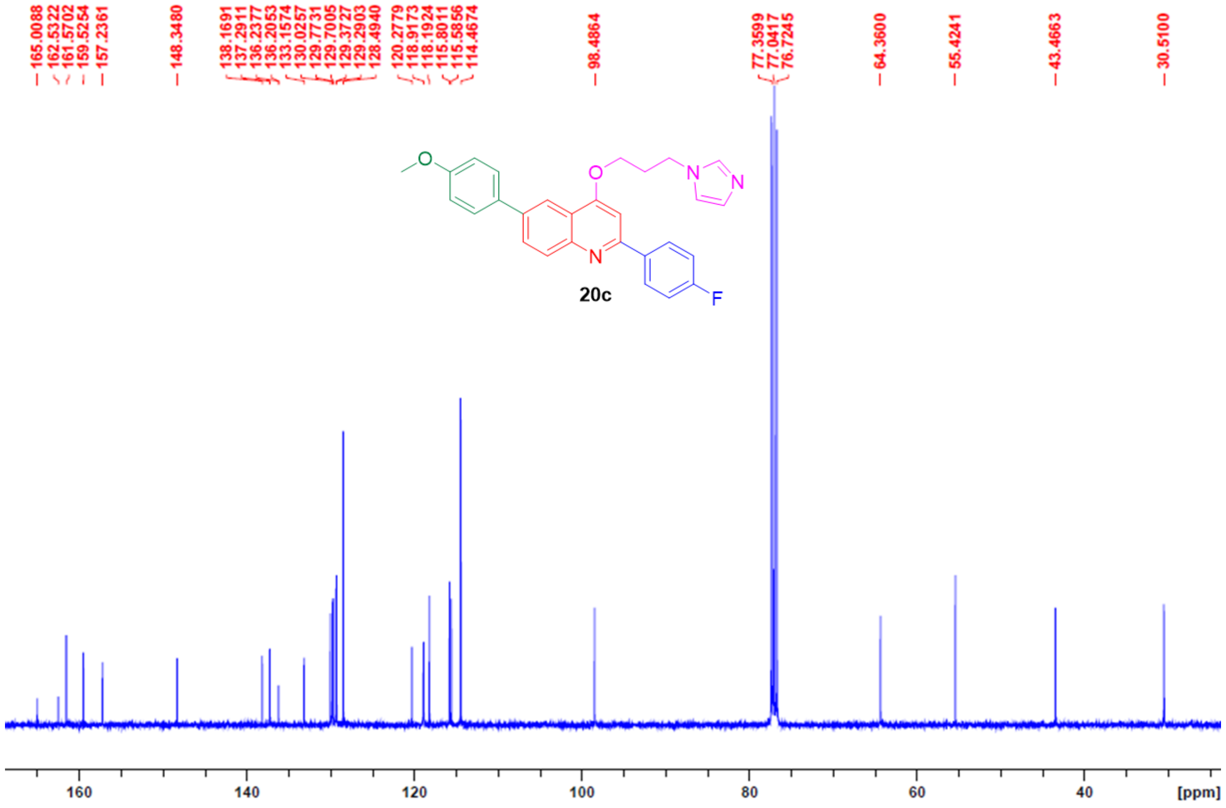

165.0088
162.5322
161.5702
159.5254
157.2361
148.3480
138.1691
137.2911
136.2377
136.2053
133.1574
130.0257
129.7731
129.7005
129.3727
129.2903
128.4940
120.2779
118.9173
118.1924
115.8011
115.5856
114.4674
98.4864
77.3599
77.0417
76.7245
64.3600
55.4241
43.4663
30.5100
O
N
N
N
F
20c
160
140
120
100
80
60
40
[ppm]

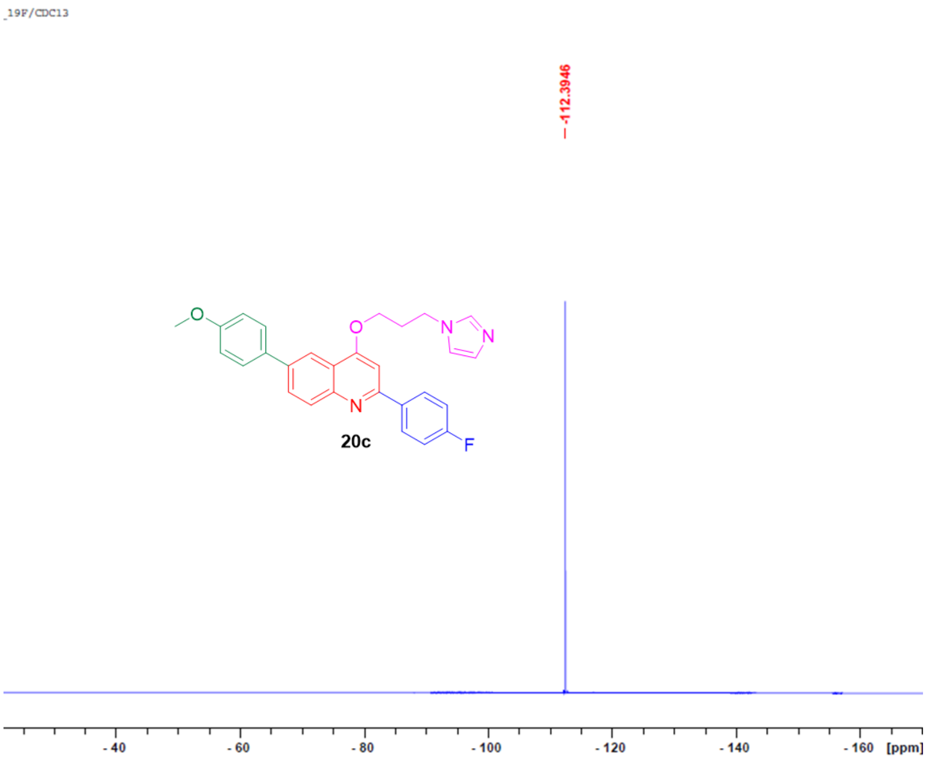

_19F/CDCl3
-112.3946
20c
F
-40
-60
-80
-100
-120
-140
-160
[ppm]

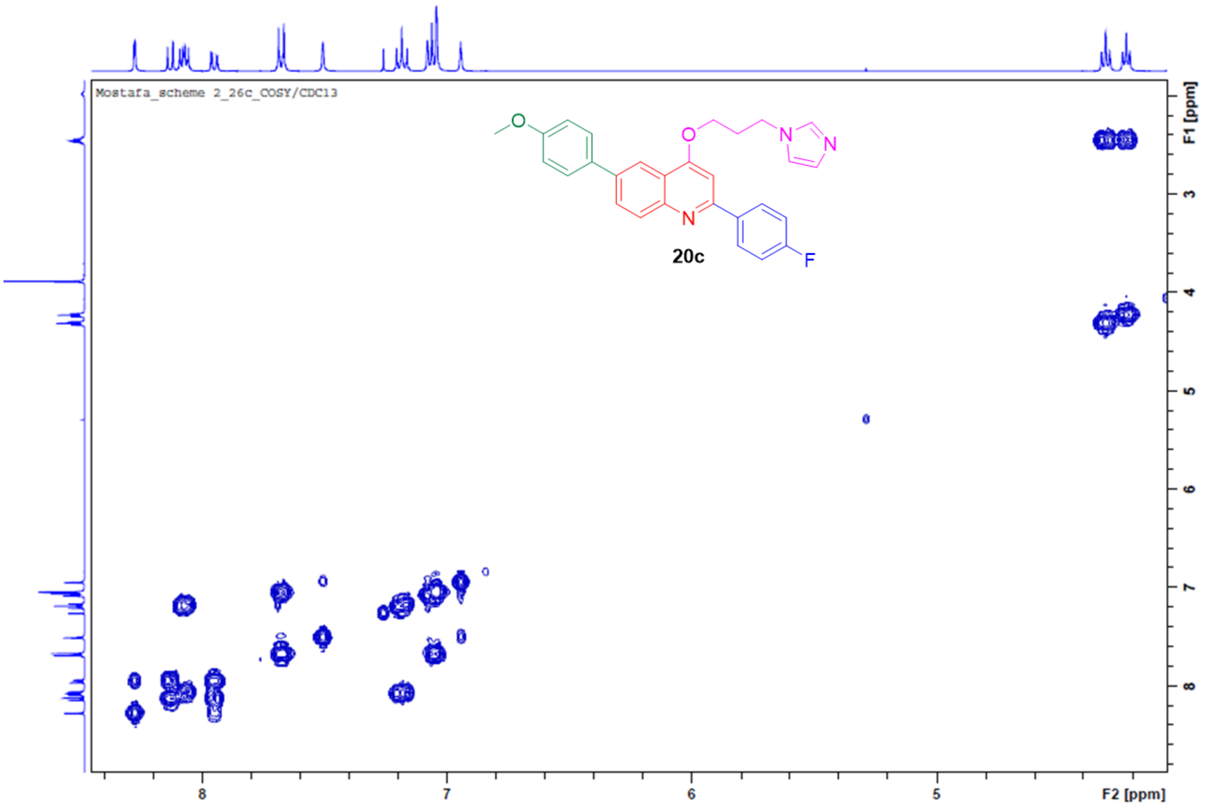

Mostafa_scheme 2_26c_COSY/CDCl3
20c
F
F1 [ppm]
F2 [ppm]

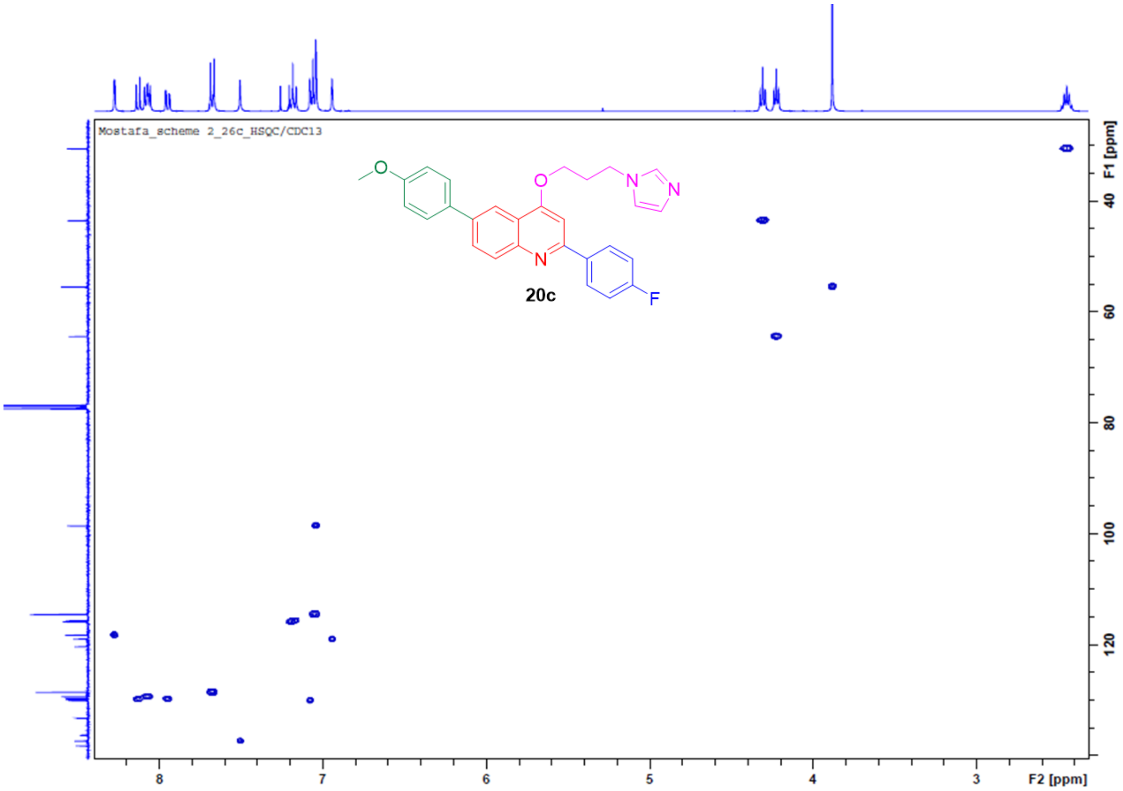
Mostafa_scheme 2_26c_HSQC/CDCl3
20c
F1 [ppm]
F2 [ppm]

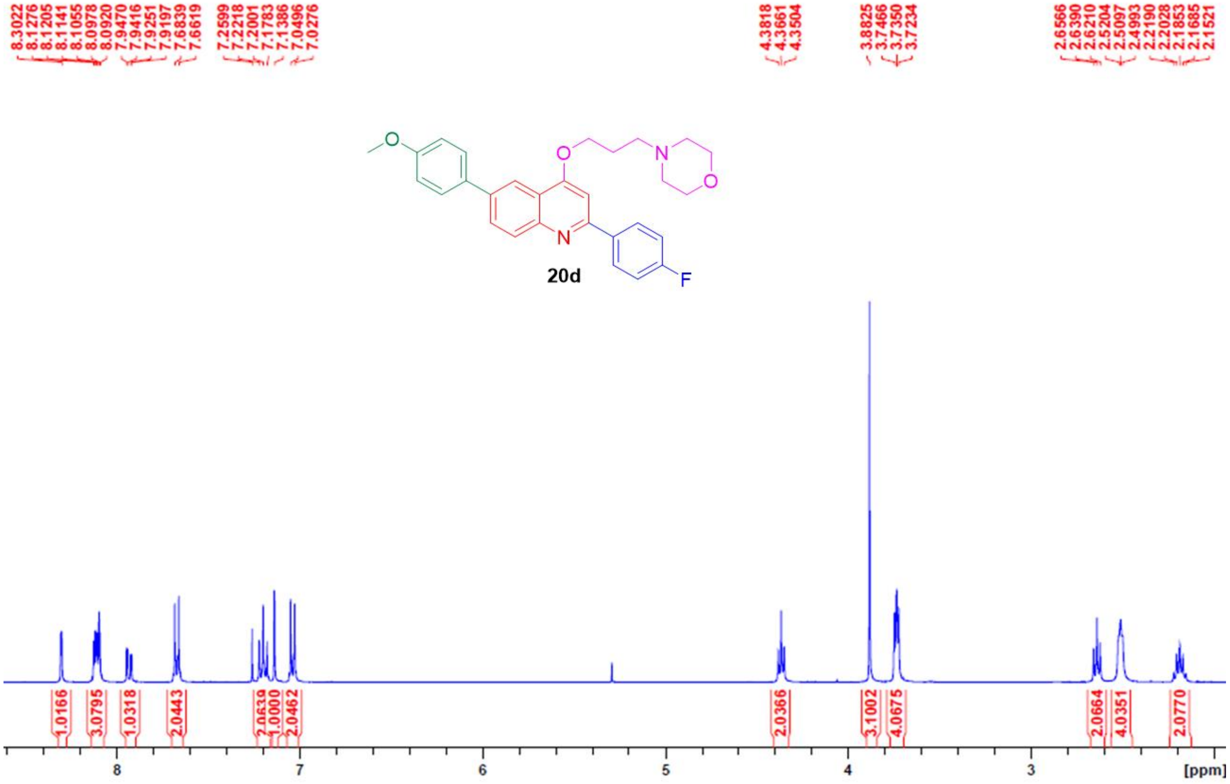
20d
[ppm]

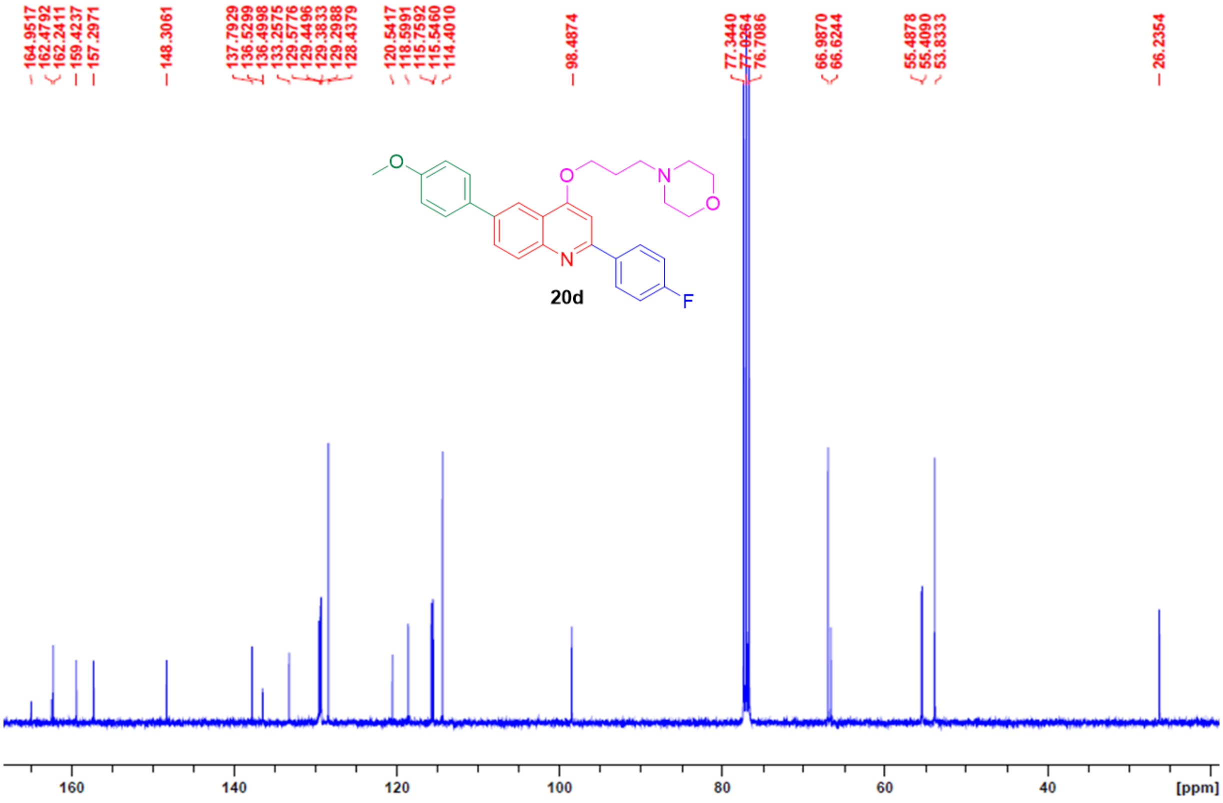
164.9517
162.4792
162.2411
159.4237
157.2971
148.3061
137.7929
136.5299
136.4998
133.2575
129.5776
129.4496
129.3833
129.2988
128.4379
120.5417
118.5991
115.7592
115.5460
114.4010
98.4874
77.3440
76.7086
66.9870
66.6244
55.4878
55.4090
53.8333
26.2354
20d
160
140
120
100
80
60
40
[ppm]

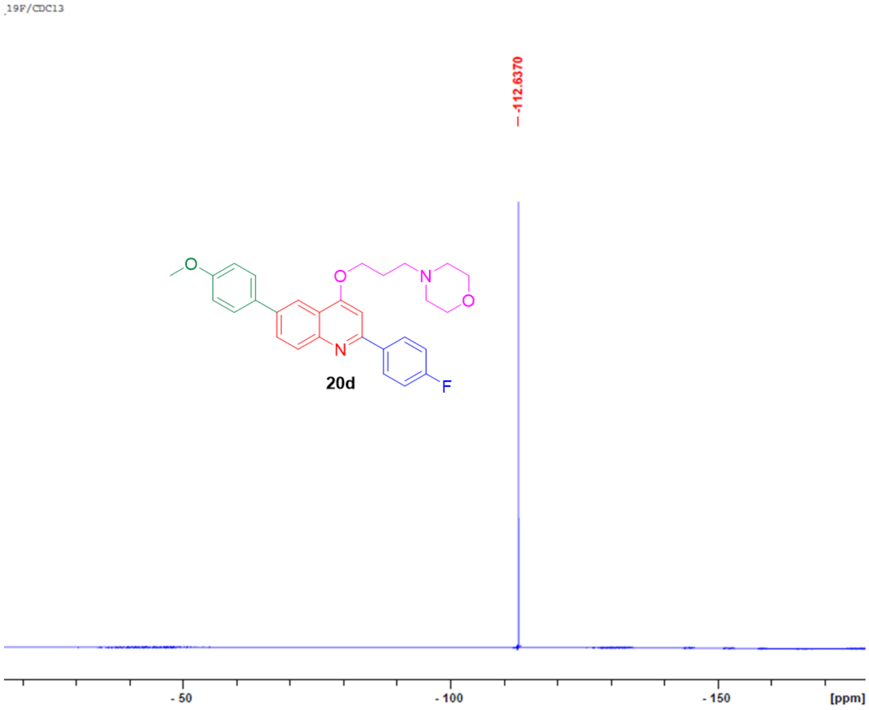
19F/CDC13
-112.6370
20d
- 50
- 100
- 150
[ppm]

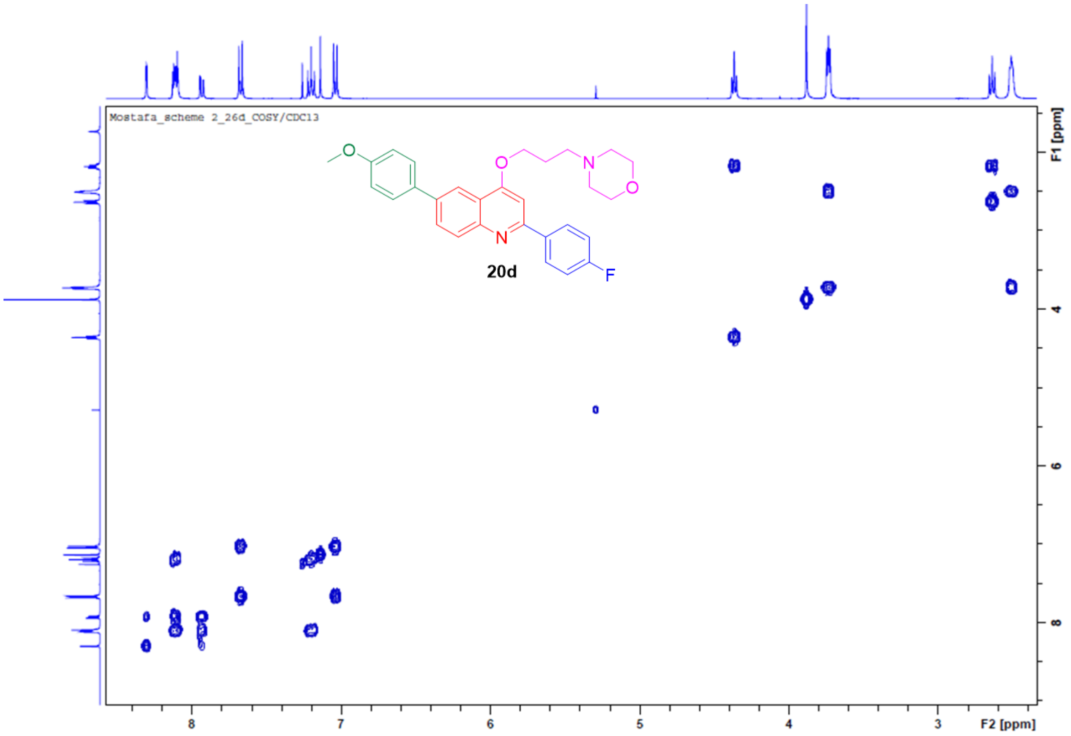
Mostafa_scheme 2_26d_COSY/CDCl3
O
N
O
N
F
20d
F1 [ppm]
F2 [ppm]

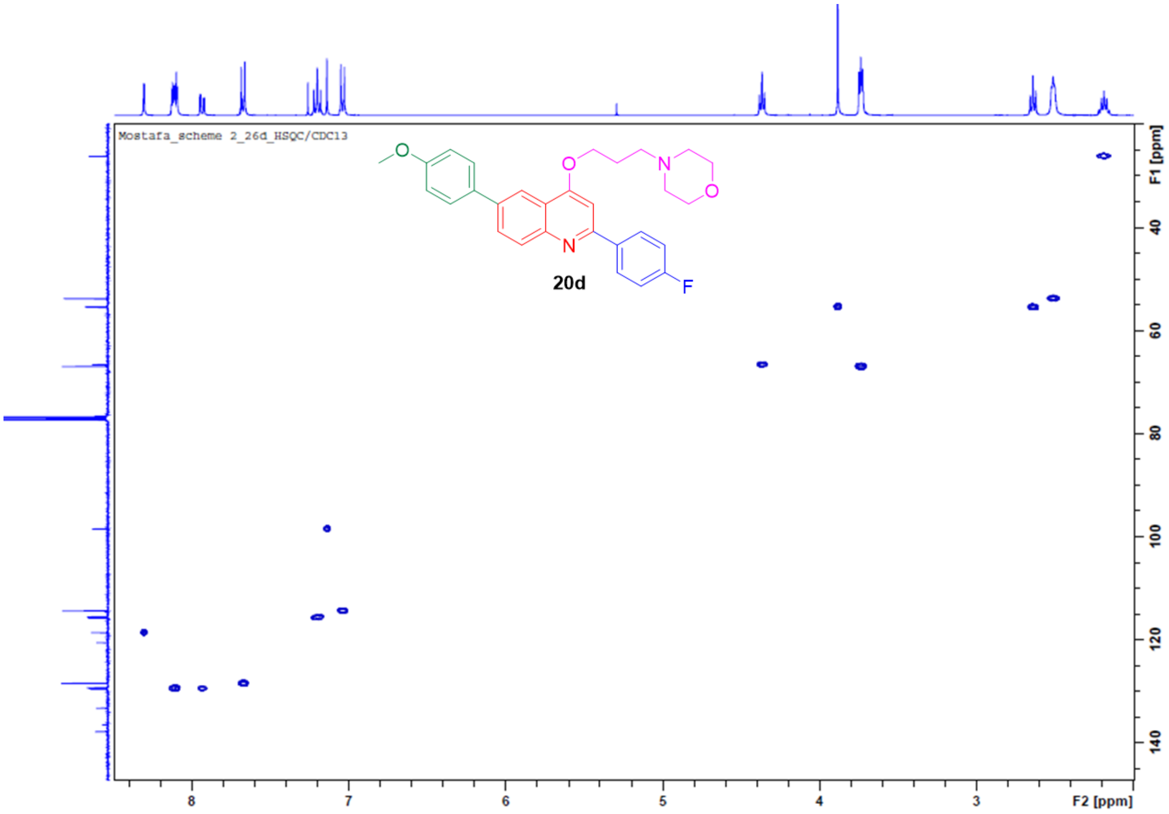
Mostafa_scheme 2_26d_HSQC/CDCl3
O
N
O
N
F
20d
F1 [ppm]
F2 [ppm]

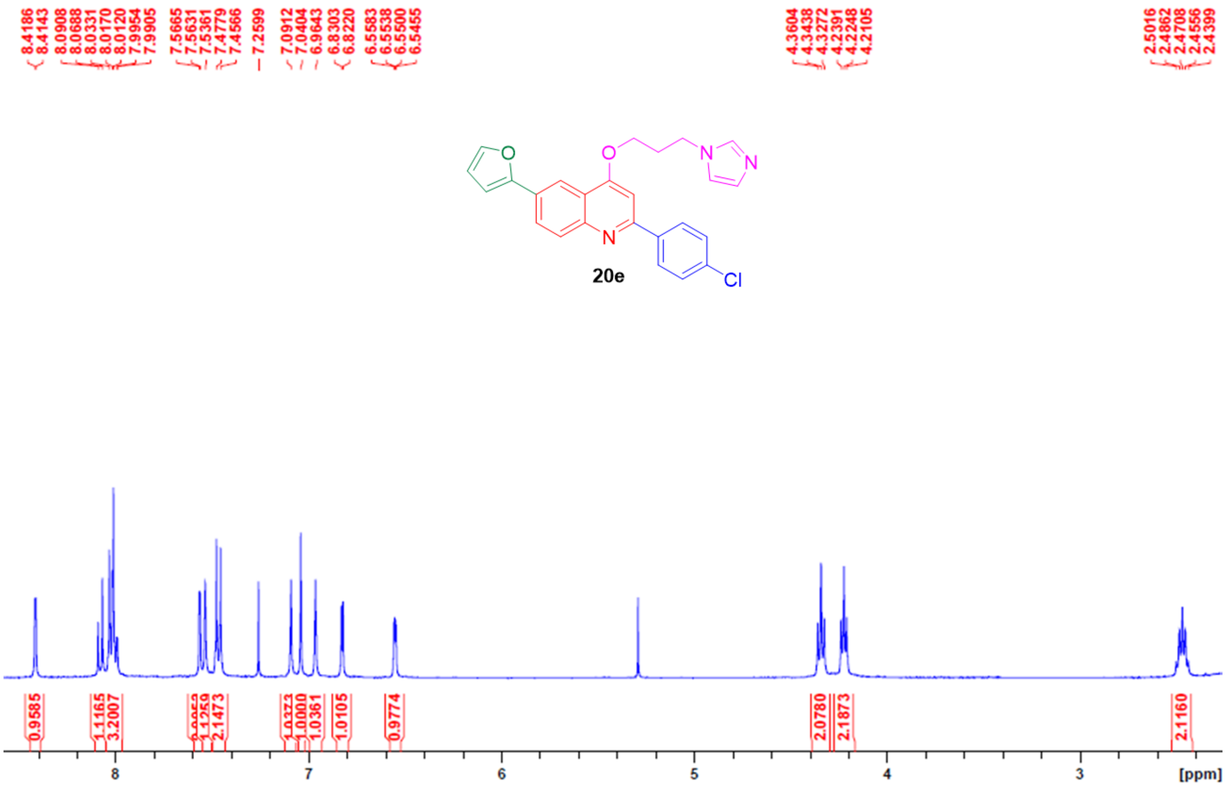
8.4186
8.4143
8.0908
8.0688
8.0331
8.0170
8.0120
7.9954
7.9905
7.5665
7.5631
7.5361
7.4779
7.4566
7.2599
7.0912
7.0404
6.9643
6.8303
6.8220
6.5583
6.5538
6.5500
6.5455
4.3604
4.3438
4.3272
4.2391
4.2248
4.2105
2.5016
2.4862
2.4708
2.4556
2.4399
20e
0.9585
1.1165
3.2007
2.1473
1.0361
1.0105
0.9774
2.0780
2.1873
2.1160
[ppm]

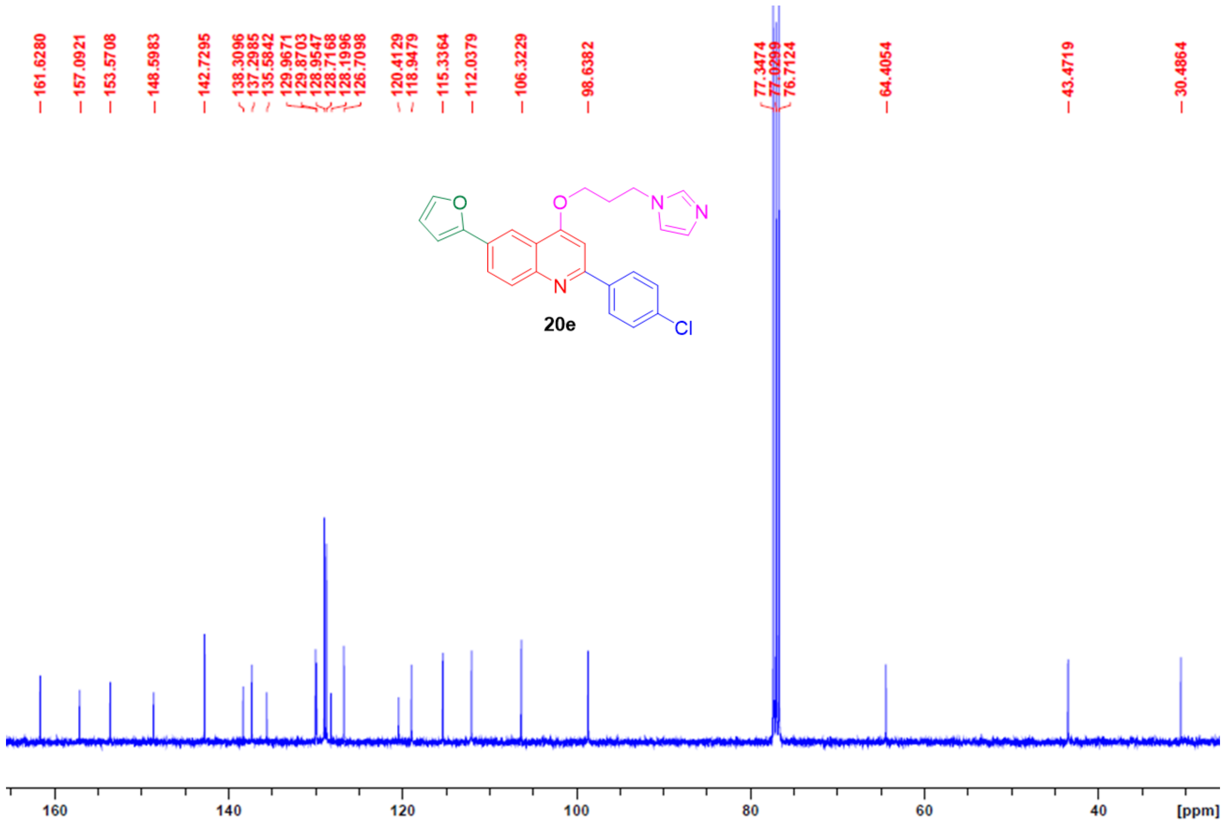
161.6280
157.0921
153.5708
148.5983
142.7295
138.3096
137.2985
135.5842
129.9671
129.8703
128.9547
128.7168
128.1996
126.7098
120.4129
118.9479
115.3364
112.0379
106.3229
98.6382
77.3474
77.0299
76.7124
64.4054
43.4719
30.4864
20e
[ppm]

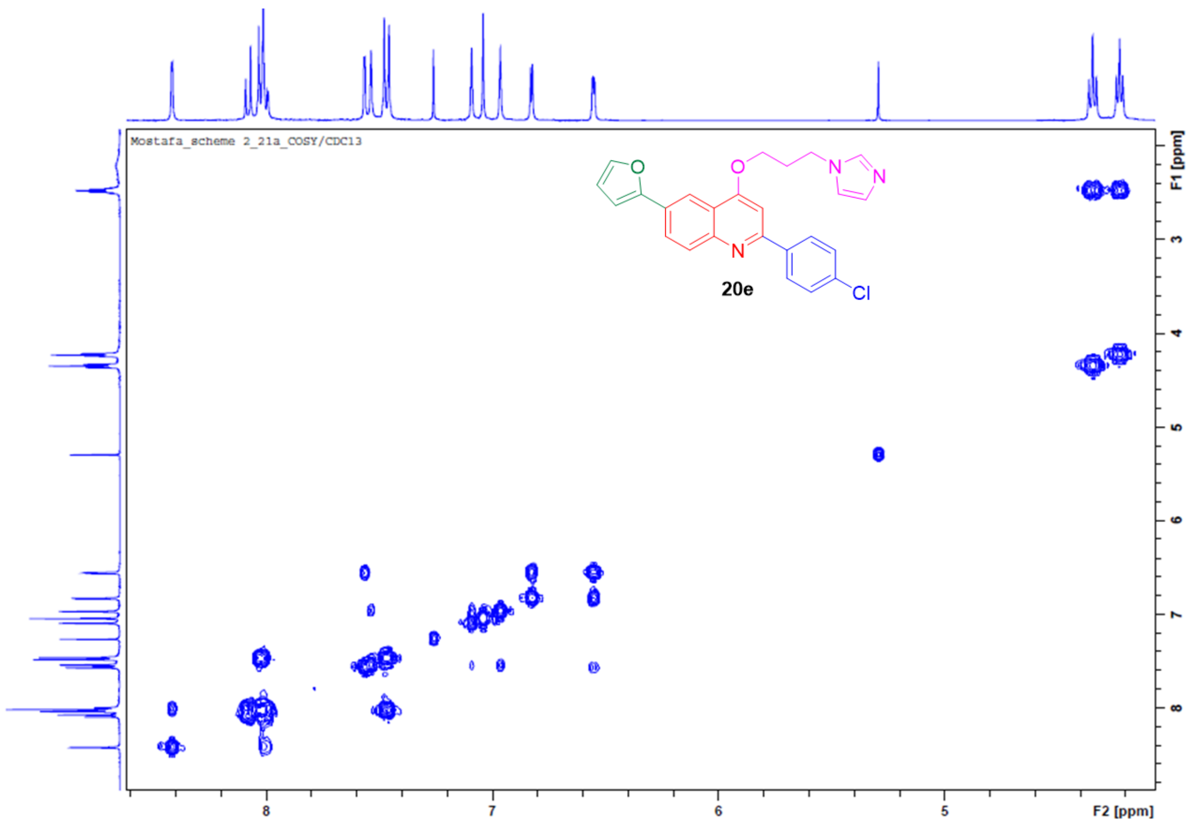
Mostafa_scheme 2_21a_COSY/CDCl3
20e
O
N
N
N
O
Cl
F1 [ppm]
F2 [ppm]

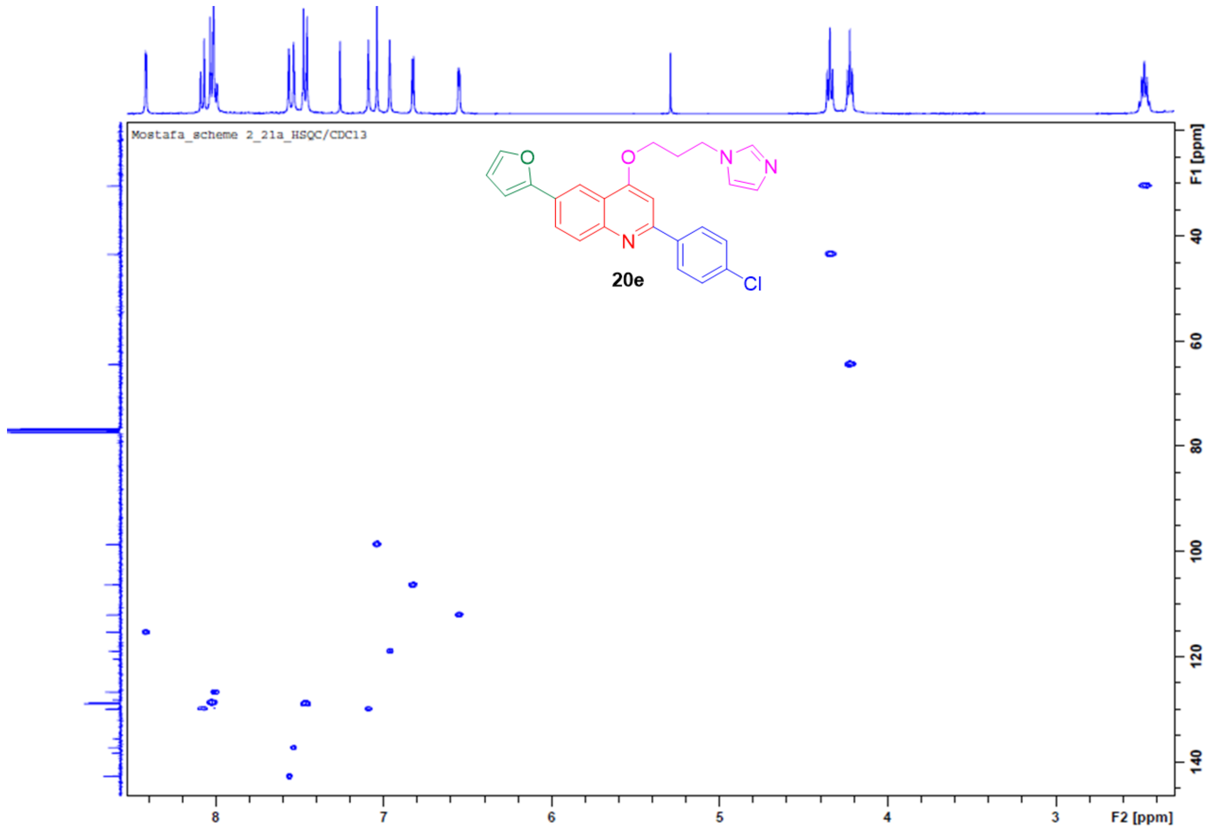
Mostafa_scheme 2_21a_HSQC/CDCl3
20e
O
N
N
N
O
Cl
F1 [ppm]
F2 [ppm]

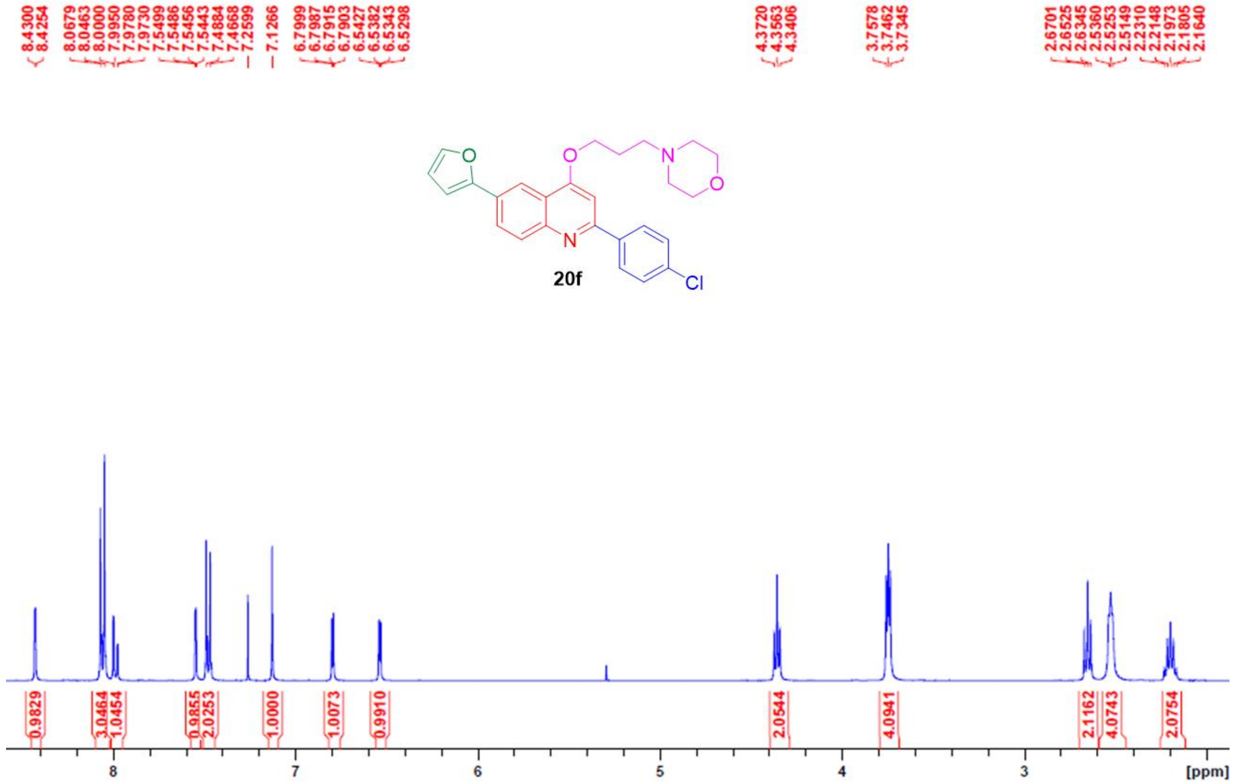
8.4300
8.4254
8.0679
8.0463
8.0000
7.9950
7.9780
7.9730
7.5499
7.5486
7.5456
7.5443
7.4884
7.4668
7.2599
7.1266
6.7999
6.7987
6.7915
6.7903
6.5427
6.5382
6.5343
6.5298
4.3720
4.3563
4.3406
3.7578
3.7462
3.7345
2.6701
2.6525
2.6345
2.5360
2.5253
2.5149
2.2310
2.2148
2.1973
2.1805
2.1640
O
N
O
N
Cl
20f
0.9829
3.0464
1.0454
0.9855
2.0253
1.0000
1.0073
0.9910
2.0544
4.0941
2.1162
4.0743
2.0754
8
7
6
5
4
3
[ppm]

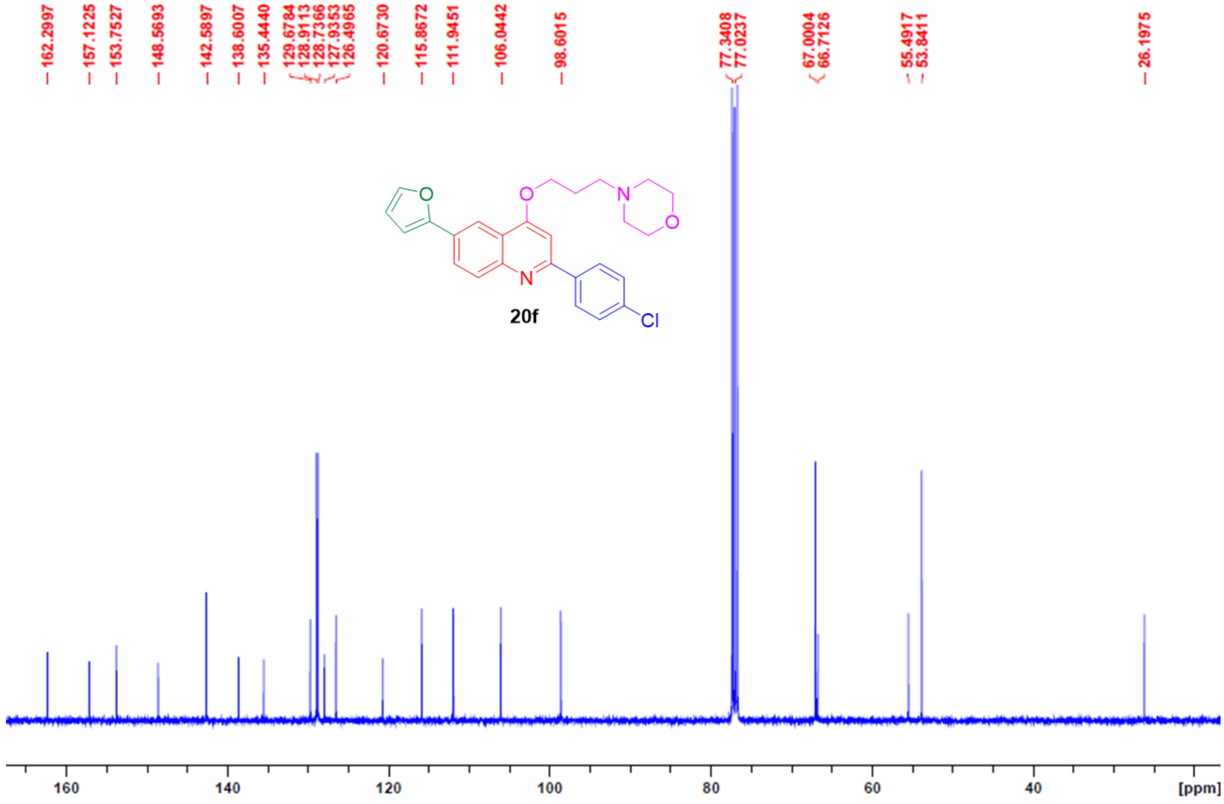
162.2997
157.1225
153.7527
148.5693
142.5897
138.6007
135.4440
129.6784
128.9113
128.7366
127.9353
126.4965
120.6730
115.8672
111.9451
106.0442
98.6015
77.3408
77.0237
67.0004
66.7126
55.4917
53.8411
26.1975
O
N
O
N
Cl
20f
160
140
120
100
80
60
40
[ppm]

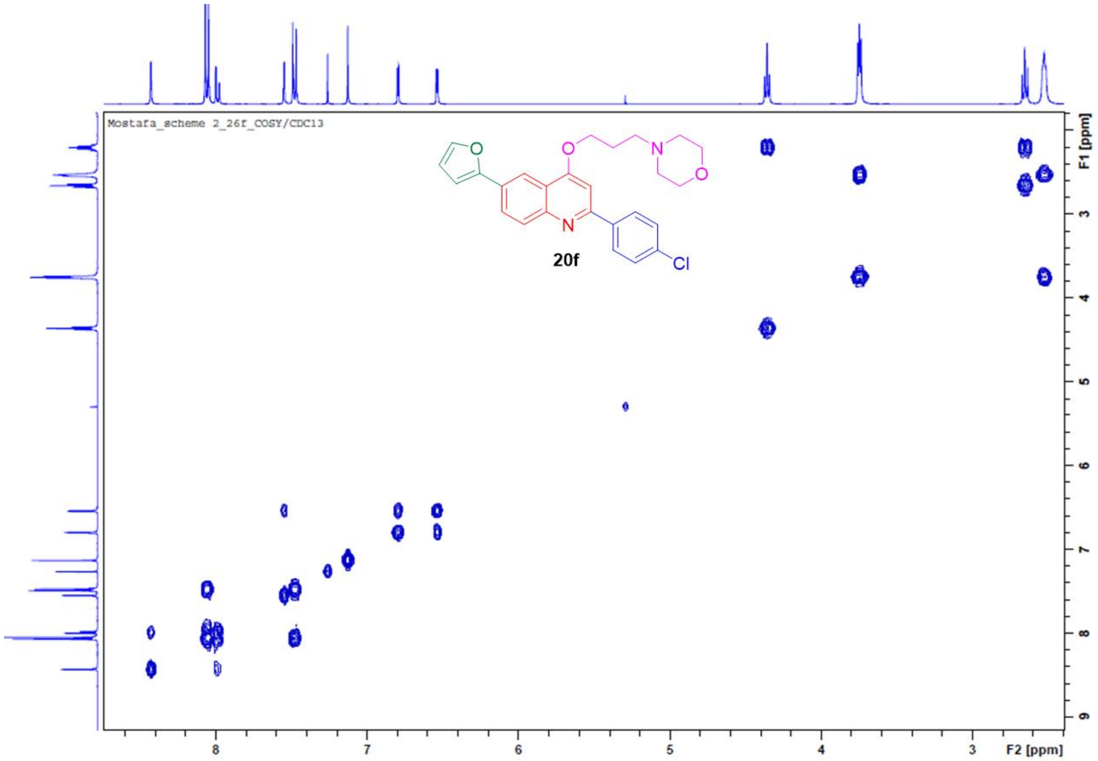
Mostafa_scheme 2_26f_COSY/CDCl3
20f
F1 [ppm]
F2 [ppm]

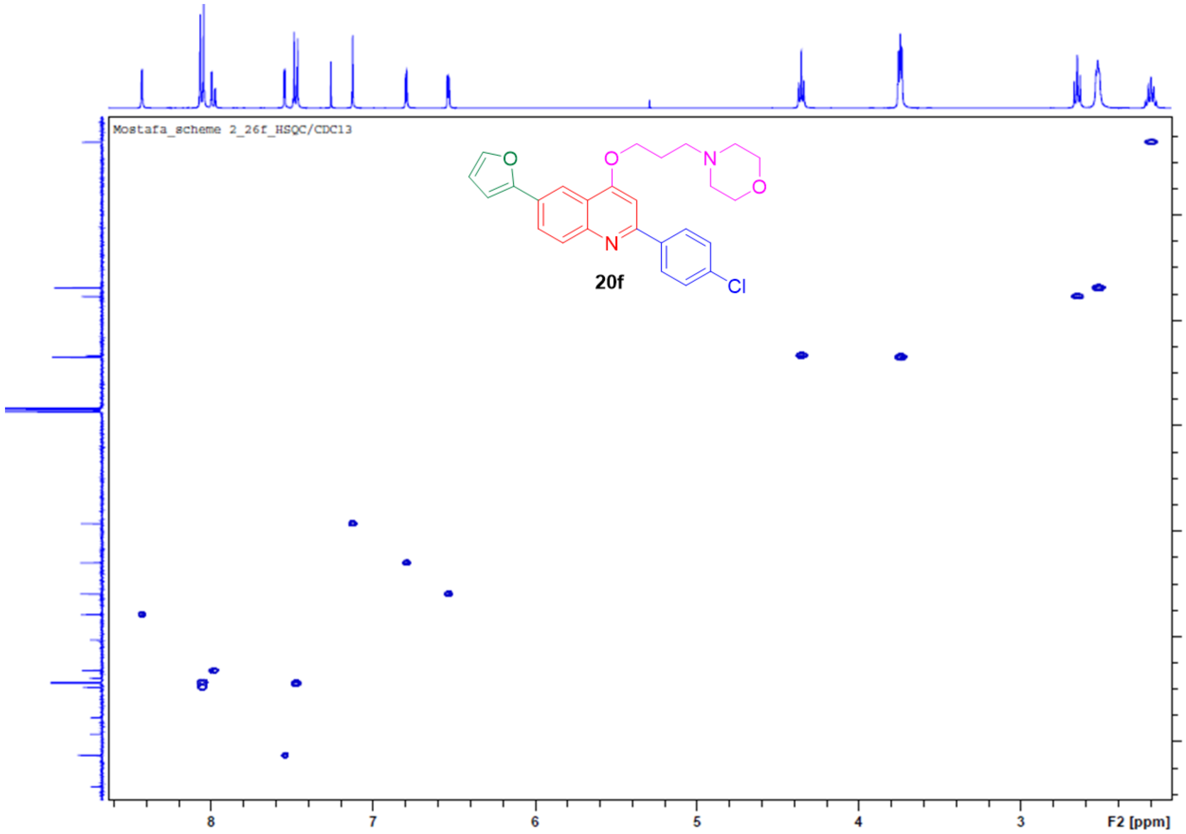
Mostafa_scheme 2_26f_HSQC/CDCl3
20f
F1 [ppm]
F2 [ppm]

Topoisomerase I-mediated DNA cleavage assay

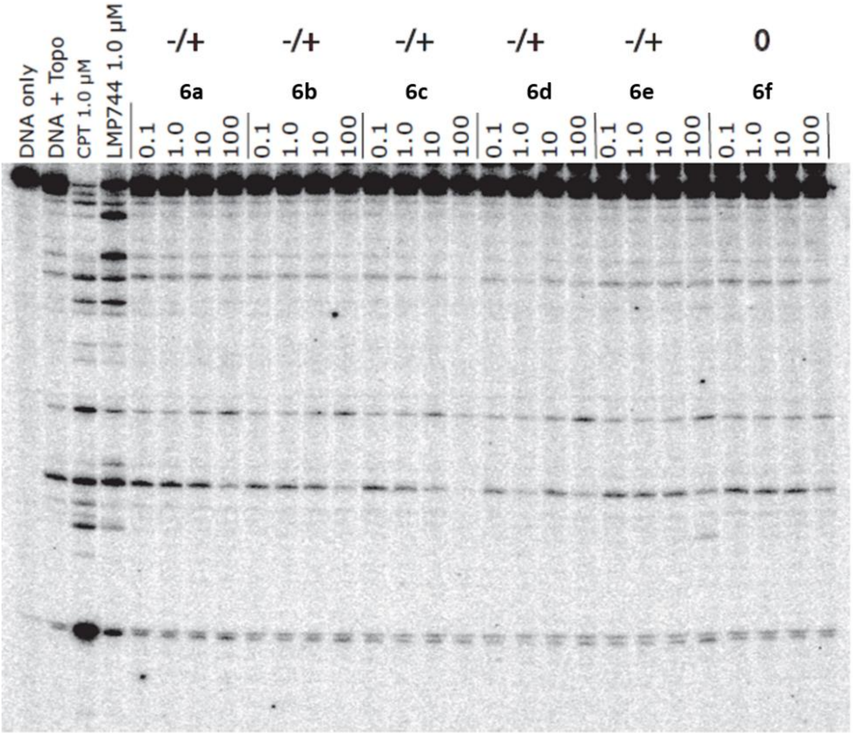

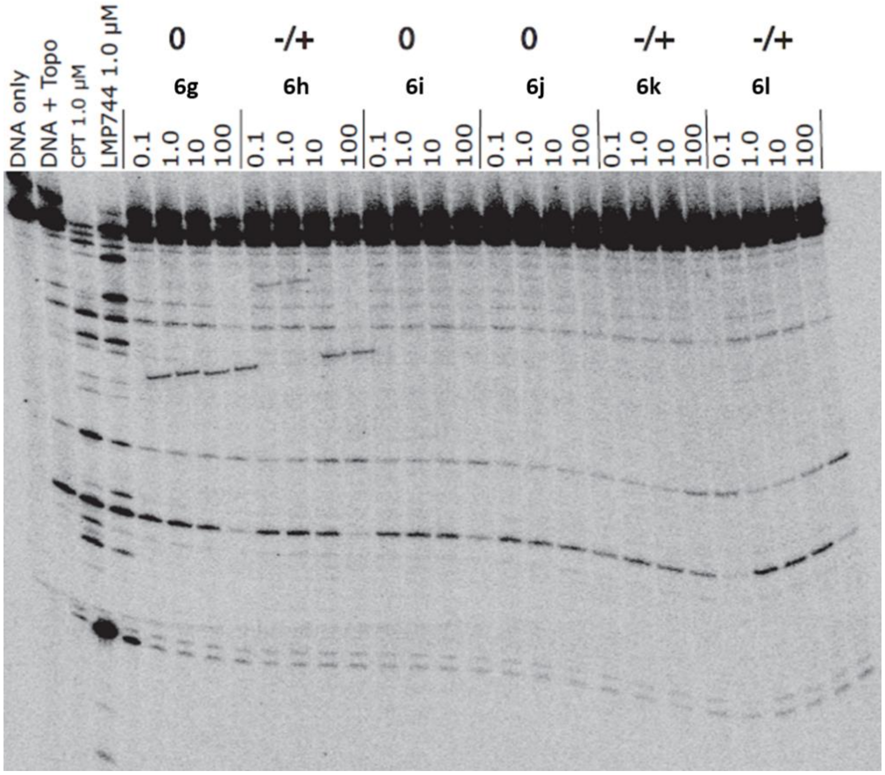

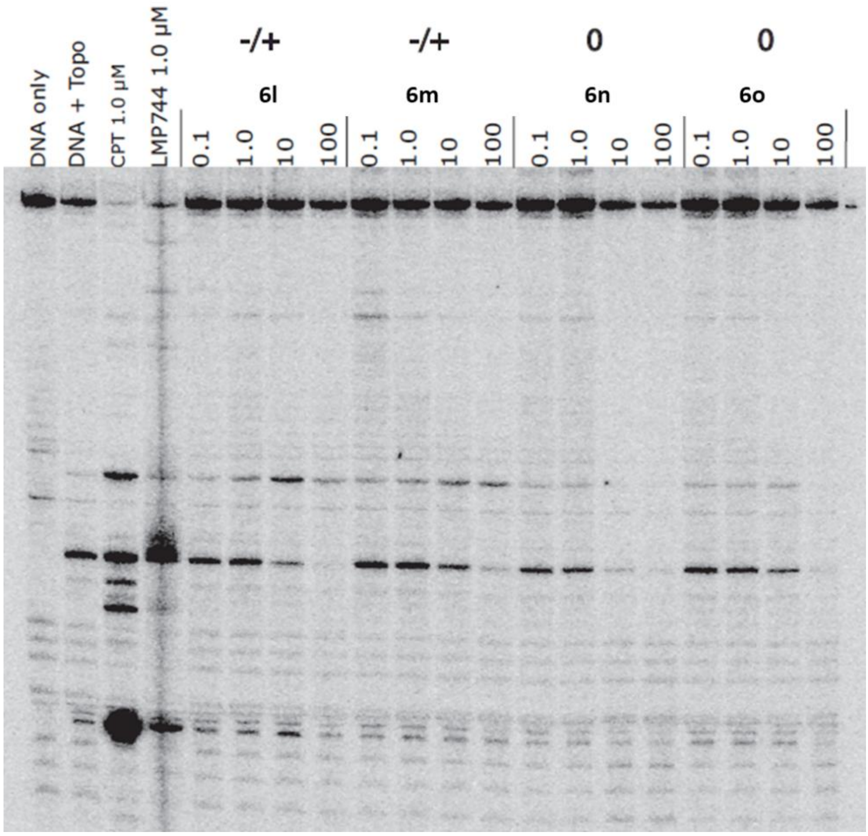

-/+
-/+
0
0
DNA only
DNA + Topo
CPT 1.0 μM
LMP744 1.0 μM
6l
6m
6n
6o
0.1
1.0
10
100

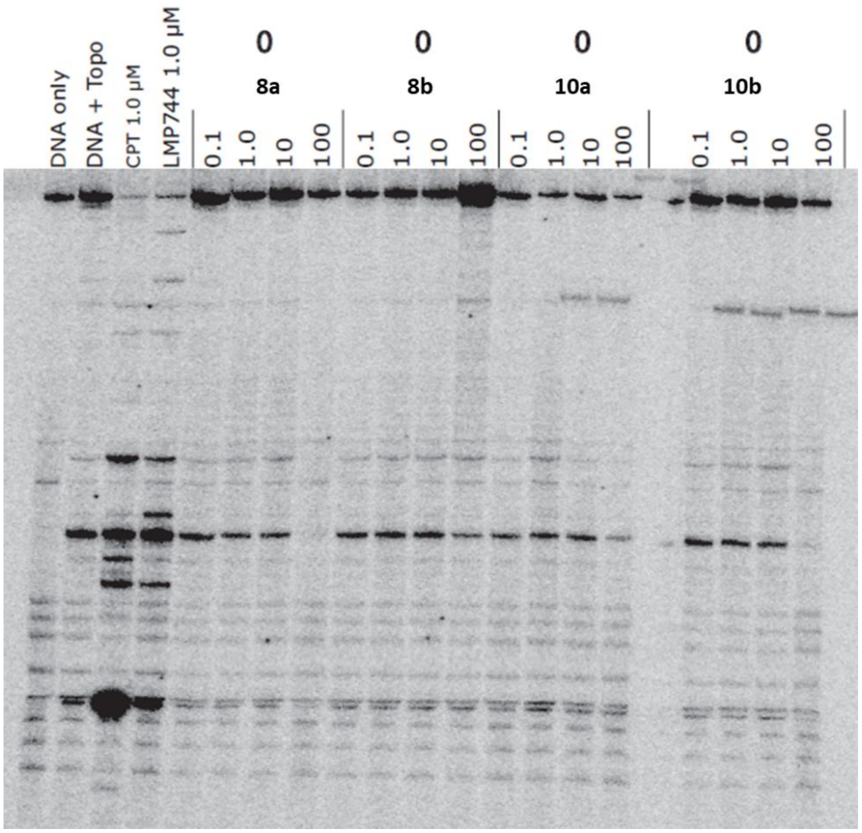

0
0
0
0
DNA only
DNA + Topo
CPT 1.0 μM
LMP744 1.0 μM
8a
8b
10a
10b
0.1
1.0
10
100

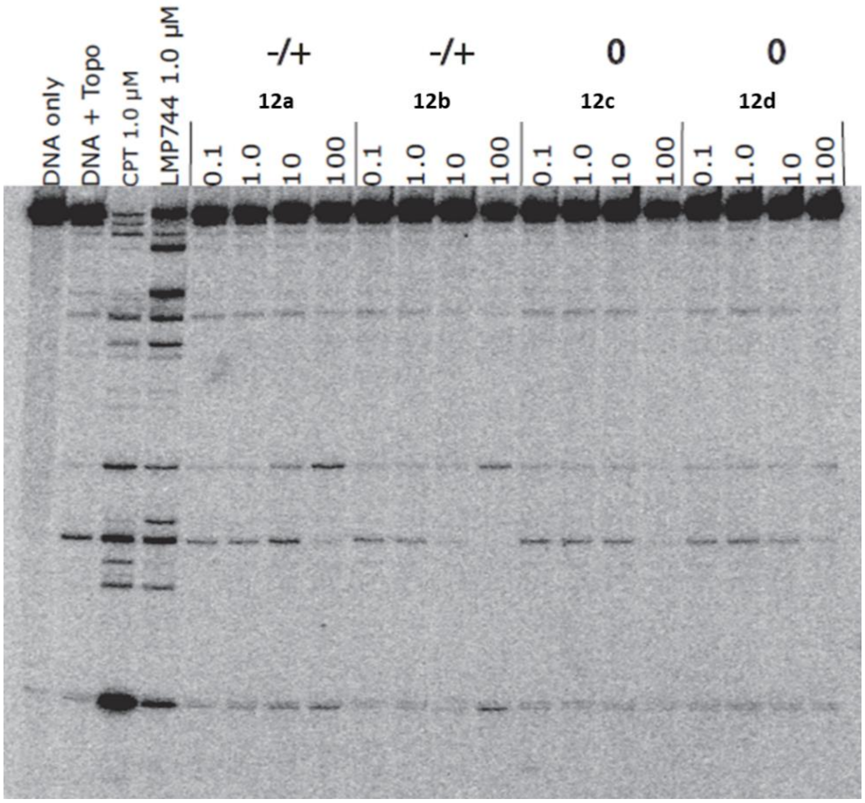

DNA only
DNA + Topo
CPT 1.0 µM
LMP744 1.0 µM
-/+
-/+
0
0
12a
12b
12c
12d
0.1
1.0
10
100
0.1
1.0
10
100
0.1
1.0
10
100
0.1
1.0
10
100

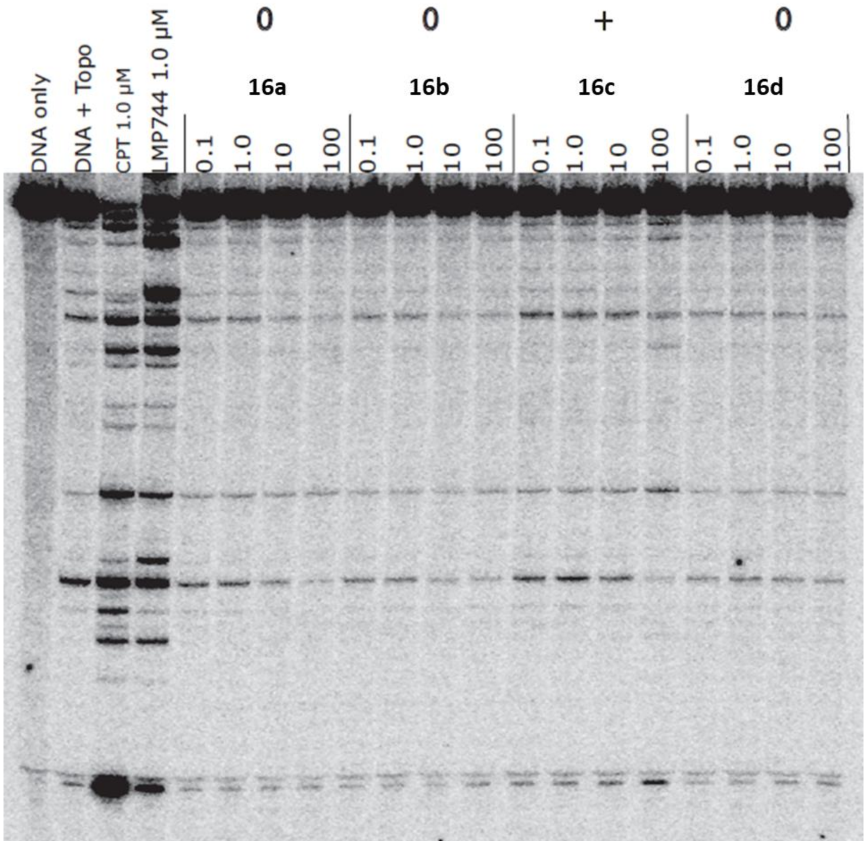

DNA only
DNA + Topo
CPT 1.0 µM
LMP744 1.0 µM
0
0
+
0
16a
16b
16c
16d
0.1
1.0
10
100
0.1
1.0
10
100
0.1
1.0
10
100
0.1
1.0
10
100

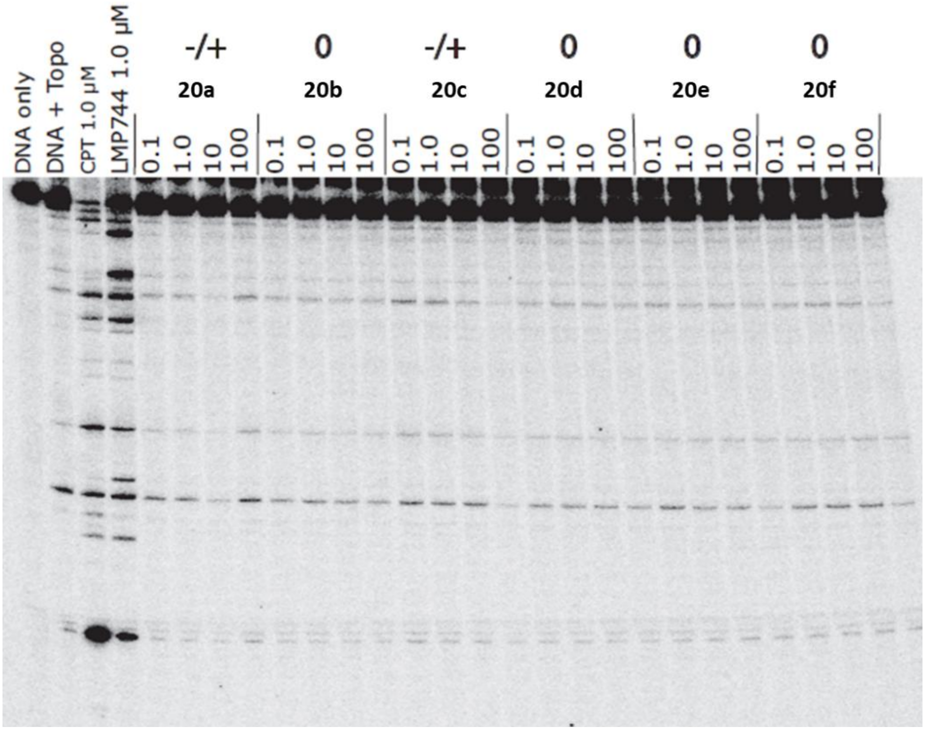

Figure S1. TOP1-mediated DNA cleavage induced by compounds **6a-o, 8a,b, 10a,b, 12a-d, 16a-d** and **20a-f**. From the left: lane 1, DNA only; lane 2, DNA + TOP1; lane 3, CPT (1μM); lane 4, LMP744 (1 μM); lanes from 5, tested compounds at 0.1, 1, 10, 100 μM.

Kinase profiling

Table S1. IC_{50} values of the target quinolines against different kinases.

Comp.	IC_{50} (nM)						
	FRK	IGF-1R	BTK	c-Src	VEGFR-1	VEGFR-2	HER-2
**6a**	825.45	1018.99	1585.09	783.22	95.30	268.29	1238.62
**6b**	9150.28	8062.93	8521.88	2497.77	3332.58	6740.59	123.00
**6c**	1559.15	2034.34	2250.63	1504.17	606.28	405.21	1380.30
**6d**	506.56	1592.03	1374.51	407.96	302.85	492.05	695.06
**6e**	8998.85	2163.20	4941.62	197.66	7329.54	3763.81	82.10
**6f**	5045.59	5345.29	8794.35	3940.04	1729.02	1940.12	638.82
**6g**	1279.54	1482.85	4756.76	2759.17	1109.74	997.07	454.75
**6h**	330.47	196.67	3477.02	3580.40	463.77	2454.98	1580.82
**6i**	1210.19	1711.01	7289.68	7013.46	800.64	8238.73	1380.98
**6j**	138.75	218.75	185.48	185.84	768.93	391.62	206.41
**6k**	3168.47	2395.70	2760.92	6448.99	5037.65	5959.68	1349.38
**6l**	743.12	1817.73	1608.41	1923.90	346.06	2397.87	756.48
**6m**	2763.16	2806.03	2458.95	980.12	864.57	2828.75	331.90
**6n**	2516.77	2808.17	819.15	82.69	555.65	1186.92	1391.69
**6o**	785.93	408.08	3828.83	3487.44	511.46	3618.59	1379.93
**8a**	845.56	1061.81	5597.33	4537.32	648.06	6367.57	989.01
**8b**	3921.86	3900.07	2916.17	7198.49	6959.50	8374.57	1068.67
**10a**	8910.00	197.92	4755.43	1025.83	4388.00	8547.43	1024.09
**10b**	4372.15	5742.42	2980.17	7775.37	8509.91	8683.43	597.68
**12a**	733.80	5366.45	195.55	55.26	1755.01	48.29	34.77
**12b**	1133.79	7156.88	213.36	42.30	5986.46	26.97	27.31
**12c**	4877.54	8995.21	99.53	118.70	7718.25	56.89	168.65
**12d**	6534.31	452.80	653.35	1452.31	2536.64	854.67	897.05
**16a**	6147.60	5232.65	4804.88	1347.51	1963.43	3408.44	2016.89
**16b**	3129.15	3491.20	2884.60	1324.72	1266.97	3231.53	2313.75
**16c**	6668.22	6690.88	5624.98	1733.09	2556.44	5095.37	1610.40
**16d**	1732.93	2229.33	2097.21	3897.24	1768.49	4692.09	357.58
**20a**	2616.37	2859.51	8668.64	2903.25	1351.78	1251.85	1012.65
**20b**	8713.05	8905.71	8976.83	4212.78	4737.61	2301.72	1650.64
**20c**	1436.19	1882.31	8667.93	9054.63	1751.70	8969.80	1231.21
**20d**	133.32	41.23	1776.06	250.84	711.77	294.23	1768.17
**20e**	216.42	3014.15	271.20	137.68	720.44	242.54	98.78
**20f**	631.98	3528.39	23.47	92.14	870.40	89.35	250.64
**TAE226**	321.76	125.79	133.94	864.05	224.58	246.37	390.08

Representative HPLC traces

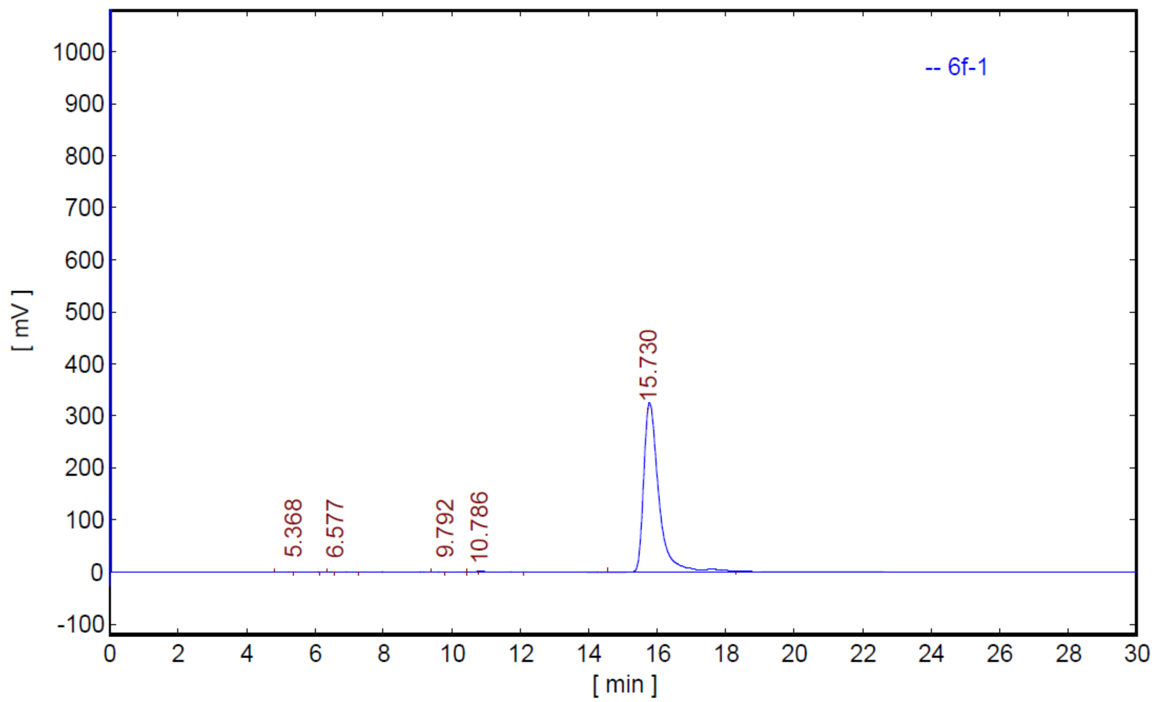

No.	Time	Area	Hight	% Conc.
**1**	5.36830	17078.85	669.71	0.17
**2**	6.57709	17783.21	791.42	0.18
**3**	9.79209	23857.56	1010.08	0.24
**4**	10.7856	49604.19	1990.82	0.49
**5**	15.7298	0025772.2	326459.65	98.93

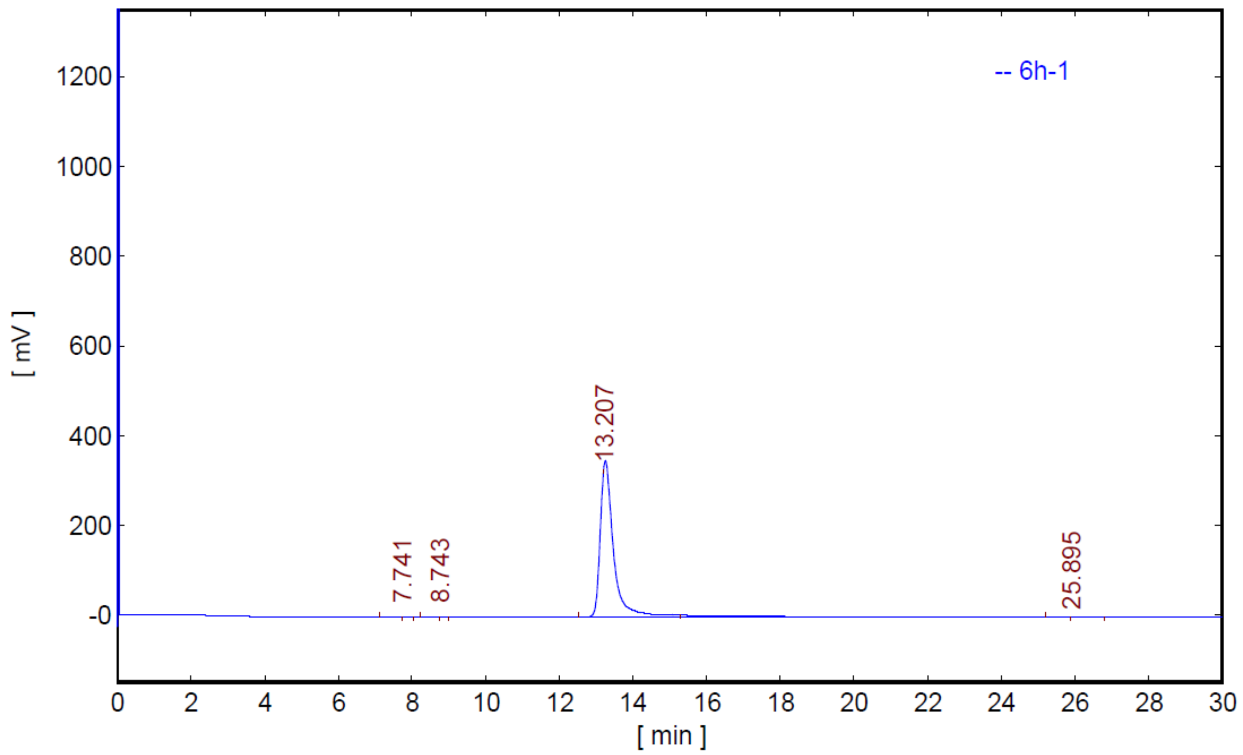

No.	Time	Area	Hight	% Conc.
1	7.74127	5586.25	154.61	0.07
2	8.74340	5377.98	254.05	0.06
3	13.2067	8523139.41	347128.01	99.75
4	25.8947	10550.28	238.14	0.12

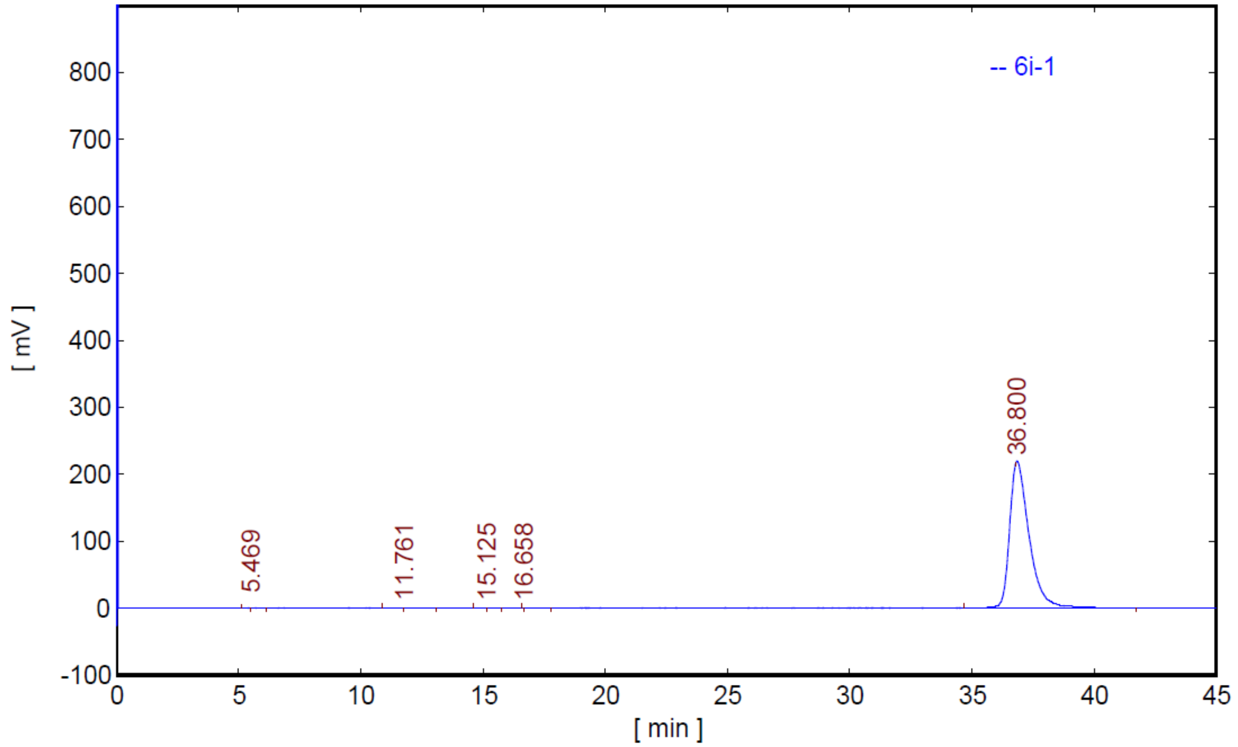

No.	Time	Area	Hight	% Conc.
1	5.46920	5196.88	200.89	0.04
2	11.7606	22951.78	390.46	0.19
3	15.1251	10567.63	460.29	0.09
4	16.6583	5907.20	45.29	0.05
5	36.8001	12063367.6	219255.17	99.63

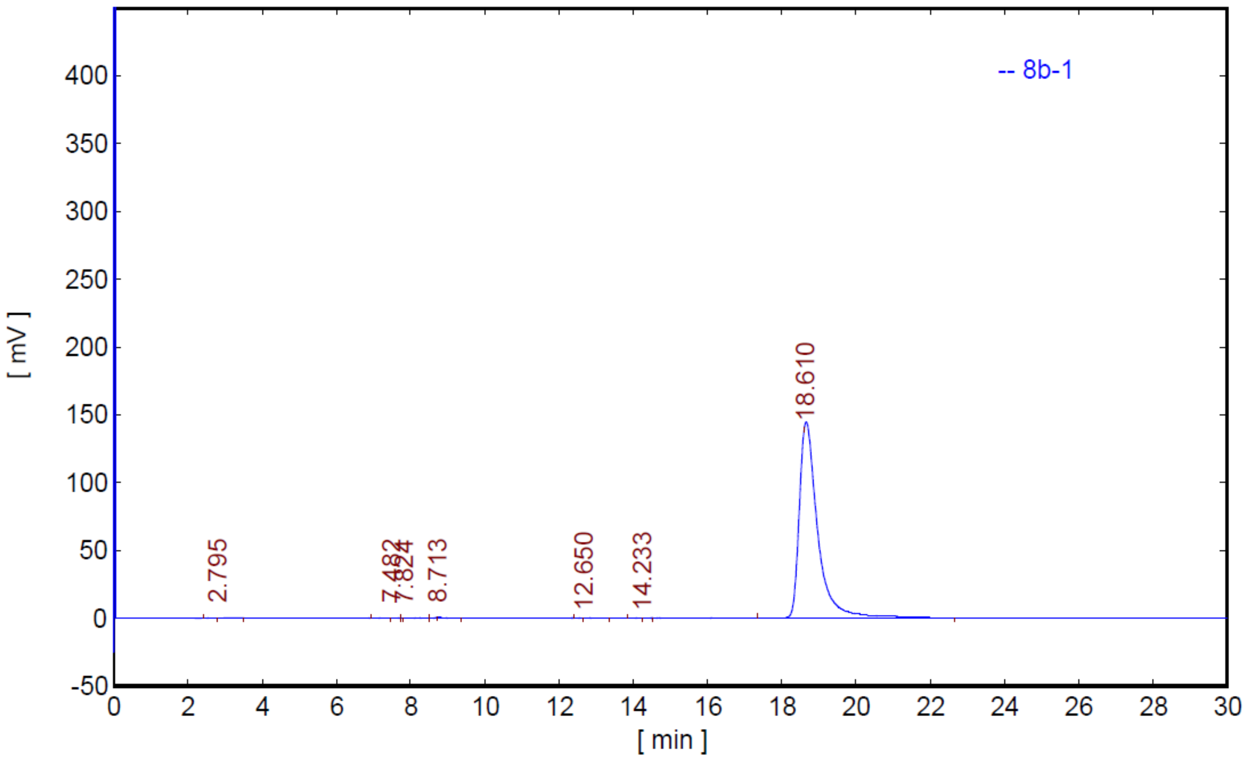

No.	Time	Area	Hight	% Conc.
**1**	2.79482	5410.71	229.53	0.11
**2**	7.48237	10709.48	369.94	0.22
**3**	7.82366	6247.08	151.18	0.13
**4**	8.71322	12992.29	1050.26	0.26
**5**	12.6497	9180.99	340.63	0.18
**6**	14.2325	7281.03	289.16	0.15
**7**	18.6099	4921757.34	144978.32	98.96

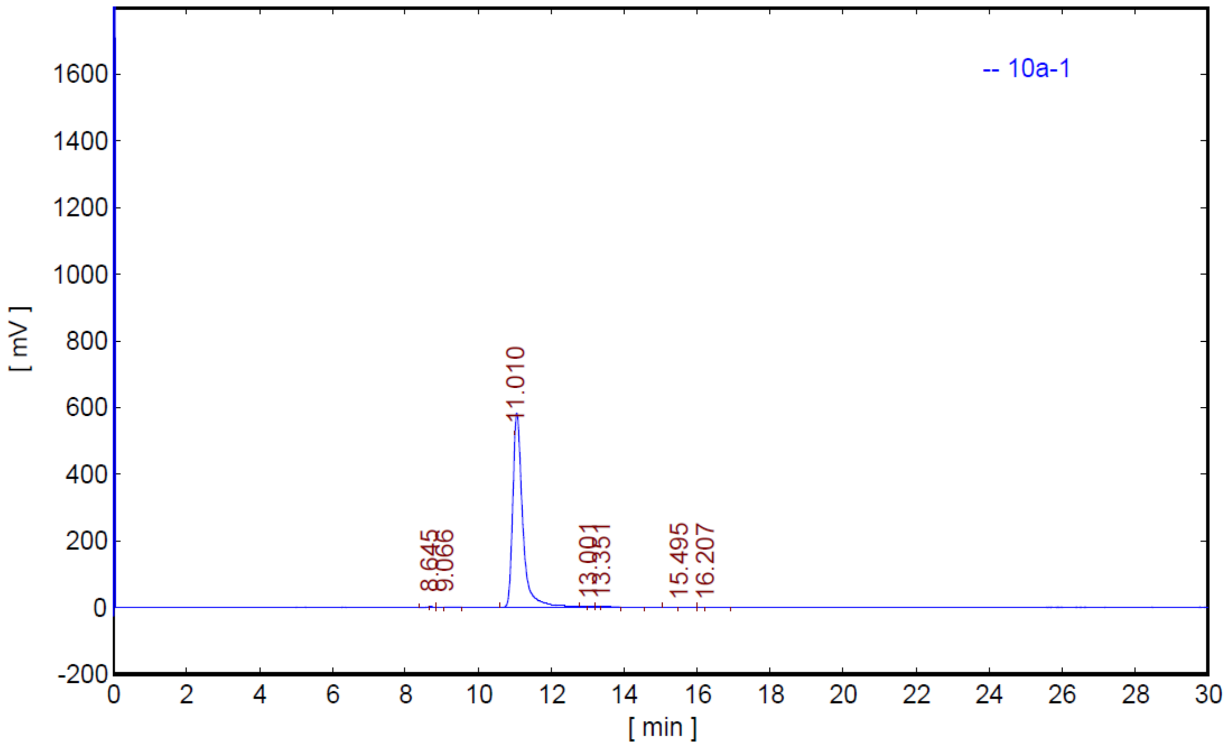

No.	Time	Area	Hight	% Conc.
**1**	8.64536	30346.51	3974.23	0.27
**2**	9.06580	17570.42	1073.60	0.16
**3**	11.0103	11136381.7	591386.75	98.63
**4**	13.0008	12307.09	743.23	0.11
**5**	13.3508	33758.24	1563.75	0.30
**6**	15.4949	43342.38	1189.39	0.38
**7**	16.2072	17803.62	686.46	0.16

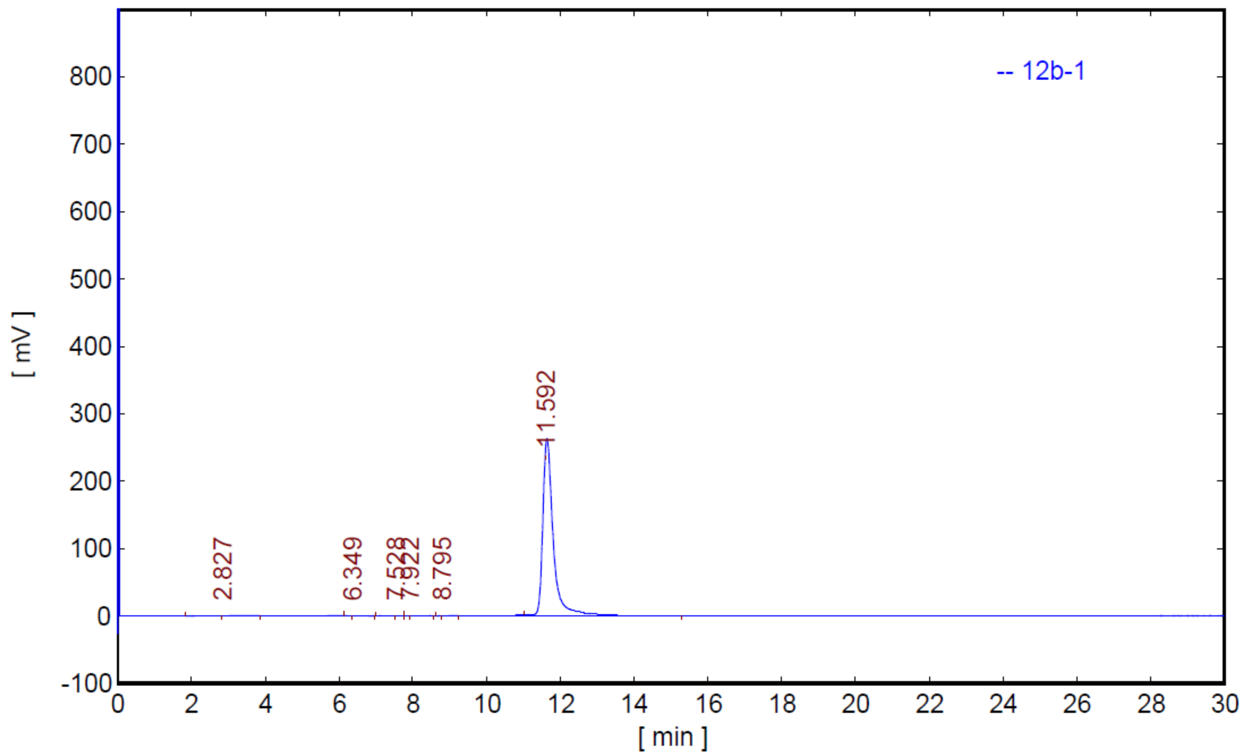

No.	Time	Area	Hight	% Conc.
1	2.82687	15783.72	275.01	0.30
2	6.34896	14799.62	380.91	0.28
3	7.52754	9120.26	322.91	0.17
4	7.92236	9995.42	217.15	0.19
5	8.79526	5692.54	443.95	0.11
6	11.5919	5177474.76	266303.41	98.94

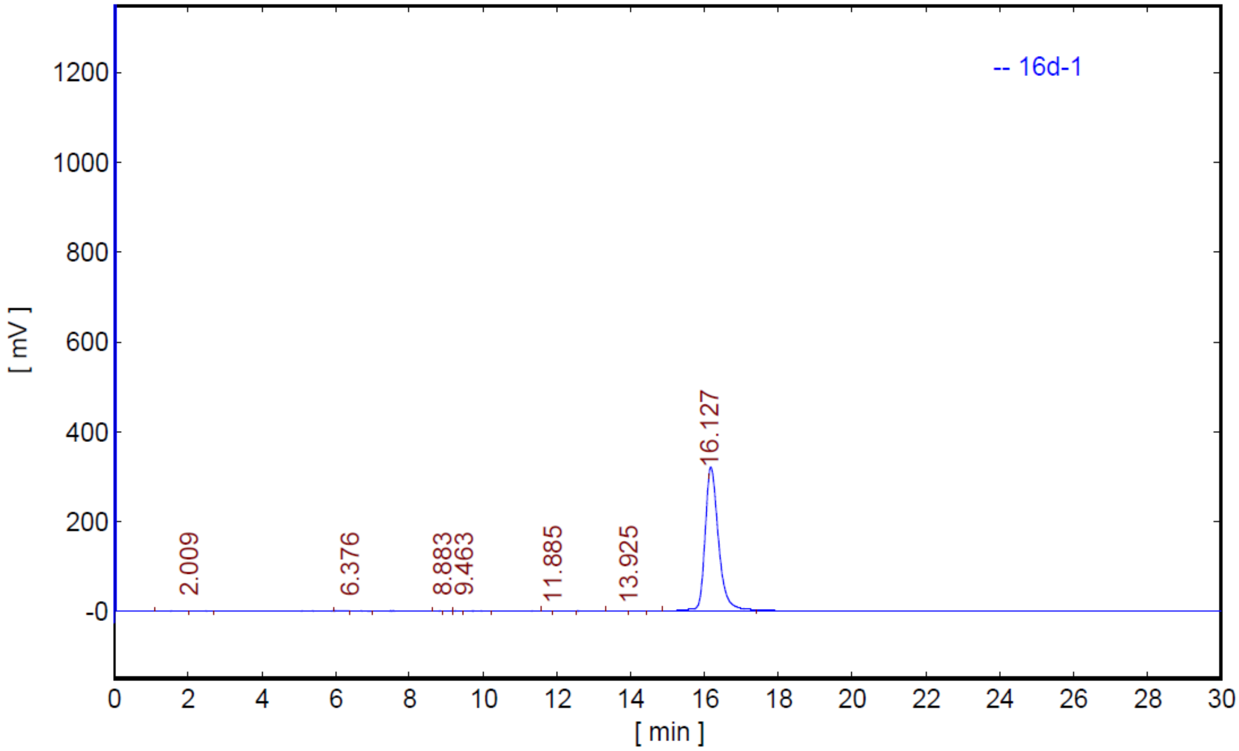

No.	Time	Area	Hight	% Conc.
1	2.00876	16147.84	382.58	0.20
2	6.37583	13388.29	433.04	0.16
3	8.88264	7523.51	480.73	0.09
4	9.46339	8706.04	267.45	0.11
5	11.8850	12436.89	503.58	0.15
6	13.9251	11058.41	345.71	0.14
7	16.1265	8114469.22	322679.85	99.15

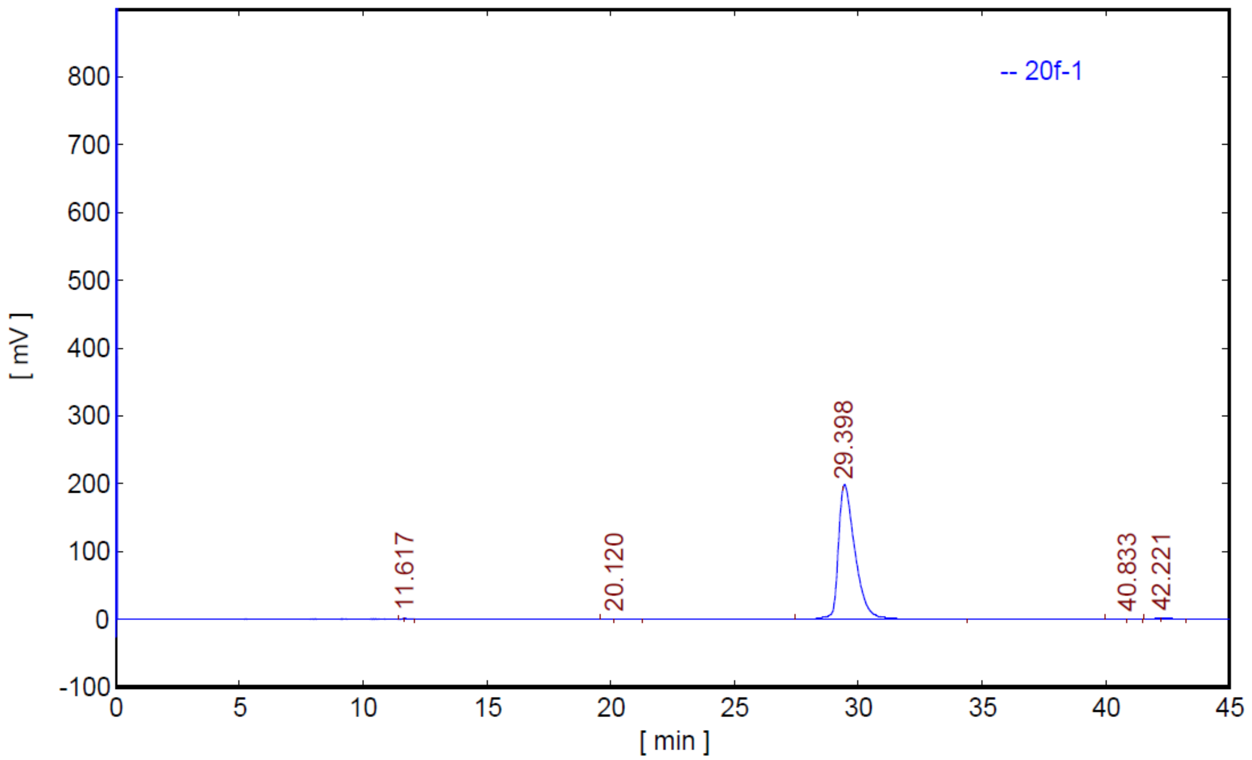

No.	Time	Area	Hight	% Conc.
1	11.6165	19836.34	1648.32	0.21
2	20.1197	15111.46	331.22	0.16
3	29.3984	9177196.25	198282.67	98.79
4	40.8329	17747.66	456.74	0.19
5	42.2212	59459.82	1529.21	0.64
